# Enhanced Control of Isoprene Polymerization with Trialkyl Rare Earth Metal Complexes through Neutral Donor Support

**DOI:** 10.1021/acs.inorgchem.3c03161

**Published:** 2023-12-08

**Authors:** Sophia C. Kosloski-Oh, Kai D. Knight, Megan E. Fieser

**Affiliations:** † Department of Chemistry, University of Southern California, Los Angeles, California 90089, United States; ‡ Wrigley Institute for Environment and Sustainability, University of Southern California, Los Angeles, California 90089, United States

## Abstract

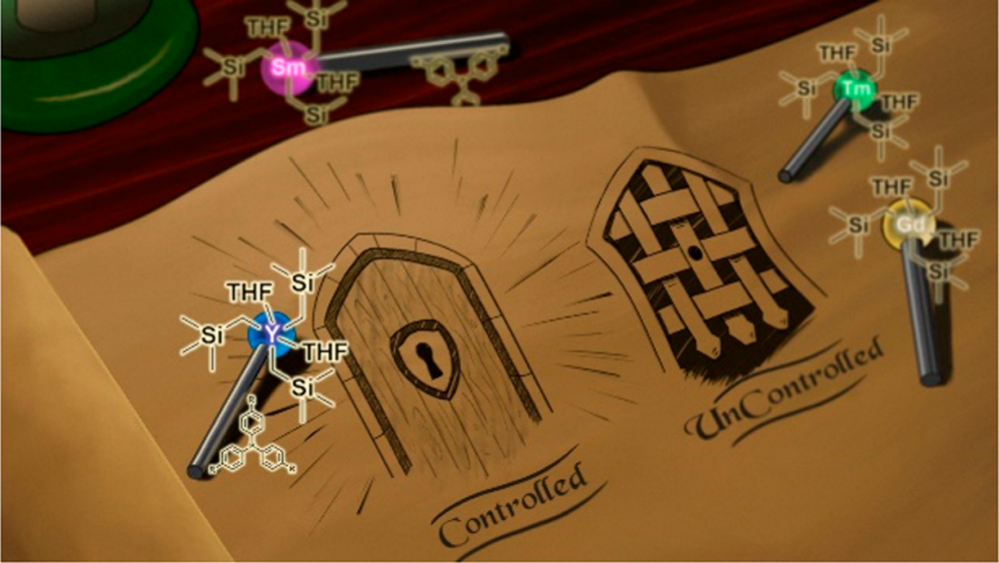

The development of catalysts for stereospecific polymerization of 1,3-dienes is an area of interest due to the robust nature of poly(1,3-diene)s’ physical and mechanical properties, as well as the material’s versatility in many applications. Dialkyl rare earth metal complexes supported by a diverse cast of ligand frameworks are selective for the polymerization of 1,3-dienes and are an exciting option for examination. However, development in this area has been hampered by the focus on complex catalyst systems that are costly to make. In this study, we synthesize a series of simple homoleptic trialkyl rare earth metal precatalysts and highlight their efficacy for isoprene polymerization using 1 or 2 equiv of [Ph_3_C][B(C_6_F_5_)_4_] activator. We investigated the addition of commercially available in situ donors, leading to the identification of triphenylphosphine as an ideal support to enhance the dispersity control and prevent loss of catalyst activity. We demonstrated how the activation and reaction conditions, including the order/time of reagent addition and donor electronics, had a major impact on the rate, control, and selectivity for the polymerization of 1,3-dienes. Further interrogation of the catalyst system signals the crucial role of triphenylphosphine in providing enhanced stability and control in this living catalyst system.

## Abstract 2

Four simple trialkyl rare earth metal complexes were synthesized and tested for their ability to polymerize isoprene. The stability and polymerization control of these complexes were influenced by the inclusion of inexpensive phosphine donors. This study investigated the impact of various factors on the rate and control of isoprene polymerization, such as reagent order, donor identity, and activation conditions.

## Introduction

Conjugated diolefin polymers, such as those made from isoprene (IP) and butadiene (BD) monomers, have been extensively studied for their high-value commercial application in the automotive and footwear industries.^1,2^ Selective and living polymerization of these monomers has led to polymers with enhanced thermal and mechanical properties. However, achieving such polymerization control often requires elaborate metal catalyst designs that are not always economically feasible for many commercial applications. For example, rare earth (RE) metal (scandium, yttrium, and the lanthanide series) dialkyl complexes have emerged as excellent but expensive catalysts for the stereoselective polymerization of 1,3-dienes.^3,4^ Many RE metal complexes have been strategically designed to achieve excellent stereoregularity and dispersity control by way of elaborate ancillary ligand supports, including pincer, β-diketiminate, cyclopentadienyl, amidinate, and many others.^5−10^ High *cis*-1,4, *trans*-1,4, or 3,4 selectivities can also be realized on demand by control of the size and Lewis acidity of the metal as well as the sterics and electronics of the supporting ancillary ligand. Supporting ligands not directly involved in the polymerization still play a vital catalytic role by providing stability, solubility, and control. Recently, we reported a family of yttrium β-diketiminate dialkyl complexes featuring only subtle variances in the ancillary ligand donor strength, which, in turn, displayed a dramatic difference in the rate and selectivity for IP polymerization.^11^ However, such designer ligands can be costly to synthesize and can require many intensive synthetic steps. Without these supporting ligands, bare metal alkyls can be used as rapid polymerization catalysts, but they often suffer from poor selectivity and poor dispersity control (vide infra). We questioned whether we could extend simple metal alkyl catalyst lifetimes and improve their control without lengthy ligand and catalyst syntheses.

**Scheme 1:**
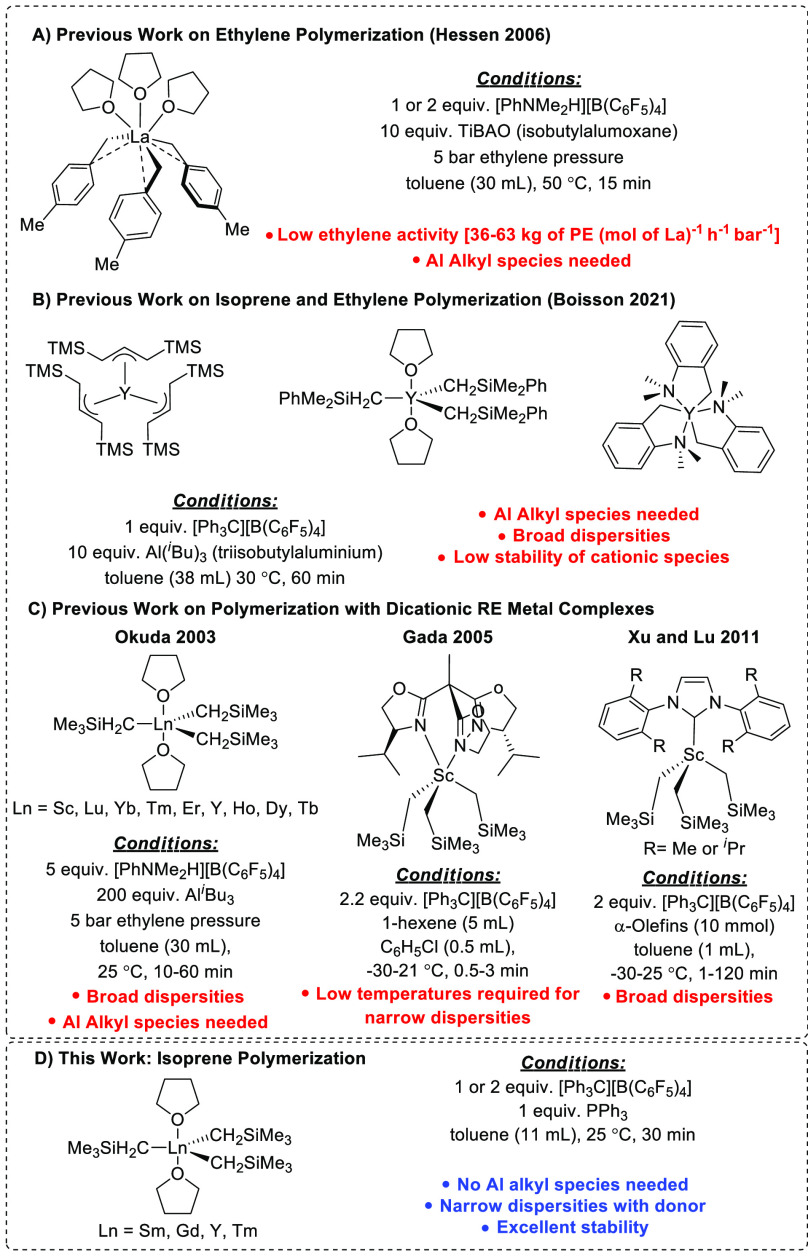
Trialkyl RE Metal Complexes for the Polymerization of Ethylene, α-Olefines, or Isoprene^16,17,25,28,29^

Several examples of simplified catalyst systems exist in both industry and academia. Some of these RE metals have already been employed by industry with excellent efficiency. For example, Goodyear Tire Company uses a ternary catalyst system composed of a Nd complex, a cocatalyst, and a halide donor (e.g., Nd carboxylates/triisobutylaluminum (Al^*i*^Bu_3_)/diethyl aluminum chloride (DEAC)) to prepare high molar mass poly(isoprene) (PIP) and copolymers of polyisoprene-*b*-polybutadiene.^12,13^ However, these systems are not living, and precise control over molar mass and dispersity has not been achieved.^14,15^ Another set of examples involves RE trialkyl complexes, which are often used as convenient metal precursors for the synthesis of many RE polymerization catalysts in the literature but are seldom treated as catalysts themselves. These examples include Hessen’s lanthanum tribenzyl, La(CH_2_Bn)_3_(THF)_3_, complex, which exhibited only mild activity toward ethylene polymerization (Scheme 1, part A), and Boisson’s yttrium triallyl and trialkyl complexes, Y[1,3-(SiMe_3_)_2_C_3_H_3_]_3_, Y(CH_2_SiMe_2_Ph)_3_(THF)_2_, and Y(CH_2_C_6_H_4_NMe_2_)_3_, and their ability to polymerize IP and ethylene (Scheme 1, part B).^16,17^ Boisson and co-workers found that the latter yttrium complexes needed activation with the cationizing agent [Ph_3_C][B(C_6_F_5_)_4_] and the addition of a Al^*i*^Bu_3_ cocatalyst for polymerization to occur. These simplified catalyst systems could achieve 70% *cis*-1,4 selectivity and demonstrated living character.

Despite having simplified catalyst systems, the aforementioned examples still require the use of an additional cocatalyst and lack stability and polymerization control. Trialkyl RE metal complexes RE(CH_2_SiMe_3_)_3_(THF)_*n*_ (RE = Sc, Lu, Tm, Yb, Y, Er, Ho, Dy, Tb, Gd, *n* = 2; RE = Sm, La, *n* = 3) are prevalent in the RE metal literature, with established synthetic routes readily available for many of the metals in the series, offering promising opportunities for studying the effects of Lewis acidity in catalysis while also being relatively temperature stable.^18^ These catalysts have even been used as neutral catalysts to polymerize several aromatic vinyl polar monomers. Xu and co-workers used RE(CH_2_SiMe_3_)_3_(THF)_2_ (RE = La, Sc, Y, Dy, Lu) and Lu(CH_2_SiMe_3_)_3_(pyridine)_2_ to polymerize 2,5-divinylpyridine at room temperature.^19^ They found that the use of a Lewis base such as THF or pyridine could change the stereoselectivity of the polymer. They noted that the use of a cocatalyst [B(C_6_F_5_)_3_] with Lu(CH_2_SiMe_3_)_3_(pyridine)_2_ led to the undesired formation of pyridine·B(C_6_F_5_)_3_, which prevented pyridine from aiding in the stereoselectivity of the polymerization as exemplified by the switch from syndiotactic to perfect isotacticity of the polymer with a broad dispersity of 3.21. In a similar study, Xu and co-workers also explored the coordination polymerization of 2-vinylpyridine using RE(CH_2_SiMe_3_)_3_(Lewis base)_2_ (RE = Y, Sc, Lu) (Lewis base = THF, pyridine) and showed that by changing the amount of Lewis base the tacticity could be modulated to achieve *mmmm* > 99% with moderate dispersity 1.22–1.69.^20^ Recently, Li and co-workers showed that these RE metal trialkyl complexes, upon activation with [Ph_3_C][B(C_6_F_5_)_4_], were effective catalysts for the polymerization of polar functionalized olefins such as isocyanides.^21^ The RE(CH_2_SiMe_3_)_3_(THF)_2_ (RE = Sc, Y, Lu) complexes particularly demonstrated exceptional tolerance to polar functionalities. Remarkably, the groups of Okuda and Gade identified the ability to abstract one or two alkyls from this precursor to form mono- and dicationic rare earth metal complexes, respectively.^22−24^ Okuda and co-workers activated several RE(CH_2_SiMe_3_)_3_(THF)_2_ complexes (RE = Tb, Dy, Ho, Y, Er, Tm, Yb, Lu, and Sc) with 5 equiv of [NMe_2_HPh][B(C_6_F_5_)_4_] activator in the presence of Al^*i*^Bu_3_ (Scheme 1, part C).^25^ These activated species were found to polymerize ethylene at room temperature with dispersities ranging from 1.7 to 5.3. In this case, larger metals showed faster conversion of ethylene, while smaller metals produced longer polymer chains. They hypothesized that a dicationic active catalyst ([M(CH_2_SiMe_3_)(solvent)_*x*_][B(C_6_F_5_)_4_]_2_) was responsible for the polymerization activity. Dicationic yttrium alkyl (with methyl and trimethylsilylmethyl alkyls) complexes have been crystallographically characterized, but only when [B(C_6_F_5_)_4_] was exchanged for the [BPh_4_] anion, and the complex was crystallized in the presence of THF and/or crown ethers.^25−27^ Gade and co-workers were able to coordinate a trisoxazoline ligand to Sc(CH_2_SiMe_3_)_3_(THF)_2_, from which two alkyl groups could be abstracted with [Ph_3_C][B(C_6_F_5_)_4_] (Scheme 1, part C).^28^ The resulting dicationic Sc species was found to be active for polymerizing 1-hexene at low temperatures. The fastest polymerization occurred at 21 °C, while the most controlled dispersity was achieved at −30 °C. Xu, Lu, and co-workers likewise used Sc(CH_2_SiMe_3_)_3_ precatalyst supported by *N*-heterocyclic carbenes to aid in the stabilization of the dicationic Sc species after activation with 2 equiv [Ph_3_C][B(C_6_F_5_)_4_] (Scheme 1, part C).^29^ They were able to achieve both a homo- and novel copolymerization of α-olefins with 1,5-hexadiene, however, dispersities were quite broad.

In a similar case, Okuda and co-workers found that Y[(μ-Me_2_)_2_-(AlMe_2_)]_3_ could be combined with [NEt_3_H][BPh_4_] to form a dicationic methyl complex, [YMe(THF)_6_][BPh_4_]_2_.^25^ When activated without the presence of THF, these complexes were active for isoprene and 1,3-butadiene polymerization, with dicationic species showing higher polymerization rates than the analogous monocationic species. The presence of trialkylaluminum reagents had a greater impact on the isoprene polymerization rate and selectivity.^30^ Once again, in these cases, dispersities ranged from 1.5 to 4.4. High selectivity for *cis*-1,4 polymerization of 1,3-dienes with a simple ligand system is promising, particularly if the dispersity can be controlled.

While these examples show that active cationic and dicationic complexes can be formed by abstracting alkyls from simple rare earth metal trialkyl species, limited information exists regarding their polymerization behavior. Many of the studies focus on different monomers or involve only the use of trialkylaluminum additives. Additionally, all the catalysts investigated have shown poor dispersity control. In this work, we report that RE(CH_2_SiMe_3_)_3_(THF)_*n*_ (RE = Sm, *n* = 3; RE = Gd, Y, Tm, *n* = 2) can be activated to achieve rapid polymerization of isoprene, with exceptional molar mass control and moderate selectivity for *cis*-1,4 polymerization, in the presence of a commercially available, soft, neutral donor (Scheme 1, part D). These results present an unexpected and attractive strategy for d^0^ metal catalysis, as the use of a soft donor to support the reactivity and control of rare earth metal ions is not typically considered due to the binding mismatch between the soft donor and hard metal ion. We also elucidate how the observed catalyst activity and polymerization control are dictated by the order and timing of addition, the stoichiometry of activating agent and donor, and the size of the metal ion.

## Results and Discussion

### Yttrium Precatalyst Screening

**Figure 1:**
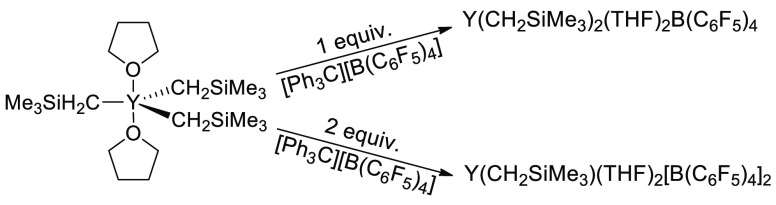
Proposed yttrium precatalyst activation with 1 or 2 equiv of [Ph_3_C][B(C_6_F_5_)_4_], assuming no interaction with toluene or the borate anion.

The yttrium trialkyl complex, Y(CH_2_SiMe_3_)_3_(THF)_2,_ was initially selected to test for polymerization activity, as it is a midsized metal, and the complex is diamagnetic. This complex was synthesized following literature procedure by reacting YCl_3_ with 2.9 equiv of (trimethylsilyl)methyllithium (LiCH_2_SiMe_3_) at reduced temperatures.^31^ Initially, the precatalyst showed no IP polymerization activity without the addition of [Ph_3_C][B(C_6_F_5_)_4_]. The yttrium precatalyst was subsequently screened with 1 or 2 equiv of [Ph_3_C][B(C_6_F_5_)_4_], leaving 2 or 1 alkyls to enact polymerizations, respectively (Figure 1). This activation step serves the dual purpose of increasing the Lewis acidity of the metal center and opening a metal binding pocket to allow enough physical space for the crucial binding of IP. As previously discussed, Boisson and co-workers activated Y(CH_2_SiMe_2_Ph)_3_(THF)_2_ with 1 equiv of [Ph_3_C][B(C_6_F_5_)_4_] in THF, which led to an isolable cationic dialkyl species with three THF molecules coordinated. This species was inactive for IP polymerization without Al^*i*^Bu_3_. When Okuda and co-workers generated dicationic monoalkyl species for polymerizations, they did so in toluene, presumably to avoid additional THF binding.^25,30^ In this report, activation is done in toluene to prevent THF from occupying the IP coordination site. While toluene can also bind to the metal center, past studies have shown that toluene does not impede polymerization in the same way THF does.

Reaction of Y(CH_2_SiMe_3_)_3_(THF)_2_ with 1 equiv of [Ph_3_C][B(C_6_F_5_)_4_] in C_7_D_8_ reveals indication of the formation of an monocationic species in the NMR. The ^1^H NMR shows two broad peaks (δ = −0.68 and −0.61). These peaks are in the region that had been previously assigned to analogous monocationic bisalkyl yttrium complexes (Figure S406 of the Supporting Information, SI).^27^ Upon addition of IP, the peaks resolved to a single doublet at −0.69 ppm giving clear indication of Y-CH_2_ bond (Figure S407). In this case, the ^19^F NMR shows one set of C_6_F_5_ resonances, which suggests the [B(C_6_F_5_)_4_] anion is either not coordinating to the Y or is fluxional (Figure S408). Reaction of Y(CH_2_SiMe_3_)_3_(THF)_2_ with 2 equiv of [Ph_3_C][B(C_6_F_5_)_4_] shows two broad Y-CH_2_ signals in the ^1^H NMR (δ = −1.10 and δ = −1.22). The chemical shift is consistent with other dicationic species (Figure S409), however, the fact that they are broad singlets suggests that the complex is highly fluxional.^26^ Indeed, contrary to the monocationic case, the ^19^F NMR shows two sets of C_6_F_5_ resonances with one being broad and having a lower intensity which suggests a minor species with coordination of the [B(C_6_F_5_)_4_] anion to the Y metal center (Figure S410). These interactions with the borate anion being fluxional have been identified for other complexes where the crystal structure shows coordination of the fluorine to a rare earth metal, while the NMR shows one set of ^19^F resonances.^32^

IP was chosen as the monomer to be studied because of its industrial value, and since it is well-established as a benchmark for general 1,3-diene polymerizations. IP polymerization using 1 or 2 equiv of [Ph_3_C][B(C_6_F_5_)_4_] was performed to determine the efficacy of the monocationic and dicationic active catalyst species (Table 1, entries 1 and 2). Polymerizations were conducted in toluene with the IP monomer added 10 min after the precatalyst was activated with [Ph_3_C][B(C_6_F_5_)_4_]. With 1 equiv of [Ph_3_C][B(C_6_F_5_)_4_], the polymerization only reached 20% conversion after 30 min, but with 2 equiv of [Ph_3_C][B(C_6_F_5_)_4_], full conversion was reached within the same time frame. These results are in accord with the increased Lewis acidity and reduced steric crowding around the yttrium metal center creating a more reactive catalyst species. There are two alkyls present when 1 equiv of [Ph_3_C][B(C_6_F_5_)_4_] is used. At the early conversion shown in entry 1 in Table 1 the experimental molar mass matches reasonably well for either one or two active alkyl initiators although it agrees more with one active alkyl initiator. This is likely due to slow initiation of the second alkyl initiator at the monocationic yttrium metal center. The molar mass is shown to better match the two alkyl initiators at higher IP conversion, discussed below. The GPC data is also consistent with this as the 30 min reaction in Table 1, entry 1 shows a monomodal distribution while a longer reaction shows a bimodal distribution in the light-scattering trace (Figures S304 and S334, respectively). This behavior was also shown for other yttrium trialkyl precatalysts when only one alkyl was abstracted.^16^

**Table 1:**
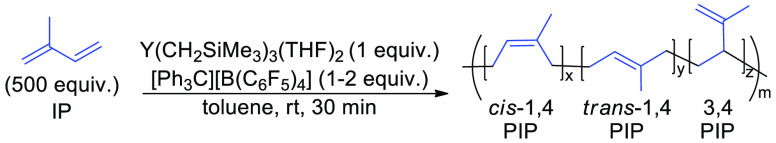
Polymerization of IP with Y(CH_2_SiMe_**3**_)_3_(THF)_2_ Precatalyst^a^

entry	borate (B) (equiv)	conv. (%)^b^	^Theor^*M*_n_ (kDa)^c^	^Exp^*M*_n_ (kDa)^d^	*Đ*^d^	microstructure^e^*cis*-1,4/*trans*-1,4/3,4
1	1	20(1)	7	5(1)	1.44(5)	41/41/18
2	2	>99	34	40(2)	2.04(2)	83/0/17

a Conditions: Y(CH_2_SiMe_3_)_3_(THF)_2_, 10 μmol; [Ph_3_C][B(C_6_F_5_)_4_] (B), 10–20 μmol; toluene, 10 mL; [IP]/**Y** = 50. All entries are done in duplicate and the error is denoted in parentheses.

b Determined by ^1^H NMR spectroscopy of crude reaction mixtures, comparing monomer peaks to polymer.

c Calculated based on one alkyl initiator, [IP mol/Y mol] × IP molar mass × Conversion.

d Determined by gel permeation chromatography (GPC) in THF using a Wyatt DAWN HELEOS II MALS detector.

e All selectivity data is an average of duplicate runs. 1,4 and 3,4 selectivity determined by ^1^H NMR. *Cis*-1,4 and *trans*-1,4 selectivity determined by ^13^C NMR.

The molar mass of the polymer from entry 2 in Table 1 matches the expected value for a single alkyl initiator on the proposed dicationic active catalyst. Similar to the trend seen with rate, the dispersity was much higher when 2 equiv of [Ph_3_C][B(C_6_F_5_)_4_] were used (*Đ* = 2.04) and narrower with only 1 equiv (*Đ* = 1.44), a further indication of the highly reactive nature of the former active catalyst. The high dispersity of the dicationic monoalkyl rare earth complex is consistent with prior literature.^25^

With 1 equiv of [Ph_3_C][B(C_6_F_5_)_4_] the microstructure shows *cis*-1,4 and *trans*-1,4 PIP, however, with 2 equiv of [Ph_3_C][B(C_6_F_5_)_4_] no *trans*-1,4 selectivity is observed. This is consistent with the trend of *cis* vs *trans* selectivity in BD polymerization for mono- vs dicationic RE initiators.^33,34^ Interestingly, when using a Y[(μ-Me_2_)_2_-(AlMe_2_)]_3_ precatalyst with 1 or 2 equiv of [PhNHMe_2_][B(C_6_F_5_)_4_] showed the same 3,4 selectivity, yet slightly different 1,4 isoprene selectivity.^30^ In that case, the cationic dialkyl species showed a higher *cis*-1,4 than this study, while the dicationic monoalkyl species showed *trans*-1,4 selectivity. It is unclear if these differences in selectivity are due to the precatalyst, activating agent, or differed reaction conditions.

### Neutral Donor Scope

While the yttrium precatalyst demonstrates a fast rate and excellent *cis*-1,4 versus *trans*-1,4 selectivity with 2 equiv of [Ph_3_C][B(C_6_F_5_)_4_], the poor dispersity exemplifies a need to modulate the catalysis. It was hypothesized that addition of a neutral donor in situ might provide an exciting avenue to give the catalyst support without intensive ligand synthesis. A previous study from Xu and co-workers showed how adding Lewis bases such as (THF, pyridine, styrene, triphenyl phosphine, triethyl phosphine, and trimethyl phosphine) to cationic RE alkyl complexes affected the stereoselectivity of 2-vinylpyridine polymerization.^35^ In these reactions, the Y(CH_2_SiMe_3_)_3_(THF)_2_ was first activated with 2 equiv of [Ph_3_C][B(C_6_F_5_)_4_] for 10 min, followed by reaction with neutral donor for another 10 min, prior to IP addition. Since most ancillary ligands for IP polymerization catalysts are multidentate, bidentate ligands with harder nitrogen donors, and softer phosphorus donors were tested (Table 2). In the cases of bidentate phosphines 1,2-bis(diphenylphosphino)ethane (dppe) and bis[(2-diphenylphosphino)phenyl] ether (DPEphos), polymerization activity was halted completely (Table 2, entries 1 and 2). Alternatively, when a harder bidentate nitrogen donor, bipyridine (bipy) was added, polymerization activity was significantly diminished but not halted (Table 2, entry 3). Given the harder nitrogen donors in bipy would lead to a comparatively weaker yttrium Lewis acid, its greater reactivity may be explained by a reduced steric crowding compared to the two phosphine donors.

**Table 2:**
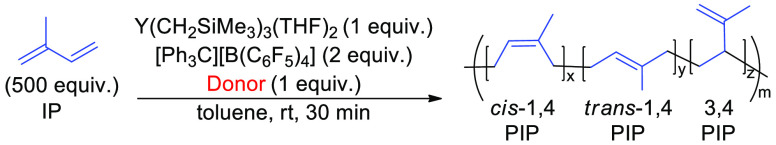
Neutral Donor Additives in the Homopolymerization of IP with **Y(CH**_**2**_**SiMe**_**3**_**)**_**3**_**(THF)**_**2**_^a^

entry	donor (D)	conv. (%)^b^	^Theor^*M*_n_ (kDa)^c^	^Exp^*M*_n_ (kDa)^d^	*Đ*^d^	microstructure^e^*cis*-1,4/*trans*-1,4/3,4
1	Dppe	0				
2	DPEphos	0				
3	Bipy	14(1)	5	30(3)	1.37(2)	66/8/26
4^f^	Pyridine	4(1)	1			
5	Acetonitrile	20(1)	7	35(5)	1.99(7)	84/0/16
6	P(*o*-tolyl)_3_	60(4)	20	37(5)	2.06(2)	83/0/17
7	PCy_3_	36(1)	12	7.9(1)	1.41(5)	66/8/26
8	PPh_3_	76(3)	26	33(2)	1.16(4)	61/13/26

a Conditions: Y(CH_2_SiMe_3_)_3_(THF)_2_, 10 μmol; [Ph_3_C][B(C_6_F_5_)_4_] (B), 20 μmol; toluene, 10 mL; 10 μmol, Donor; [IP]/**Y** = 500. All entries are done in duplicate and the error is denoted in parentheses.

b Determined by ^1^H NMR spectroscopy of crude reaction mixtures, comparing monomer peaks to polymer.

c Calculated based on one alkyl initiator, [IP mol/Y mol] × IP molar mass × Conversion.

d Determined by gel permeation chromatography (GPC) in THF using a Wyatt DAWN HELEOS II MALS detector.

e All selectivity data is an average of duplicate runs. 1,4 and 3,4 selectivity determined by ^1^H NMR. *cis*-1,4 and *trans*-1,4 selectivity determined by ^13^C NMR.

f Yield was insufficient for characterization.

Because of the poor activity with bidentate neutral donors, several monodentate nitrogen and phosphine donors were tested. Two monodentate nitrogen donors, pyridine, and acetonitrile, showed comparable conversions to those seen with bipy (Table 2, entries 4 and 5), suggesting the denticity of these donors was not crucial. Yttrium dicationic complexes have been known to activate C–H bonds of pyridine, although this is extremely slow at room temperature in neat pyridine, so it is unlikely to occur in this case with catalytic quantities.^36^ Although, it also may not be likely in this case as Okuda and co-workers used pyridine-*d*_5_ to characterize similar dicationic species.^27^ Next, three monodentate phosphine ligands, tri(ortho-tolyl)phosphine (P(*o*-tolyl)_3_), tricyclohexylphosphine (PCy_3_), and triphenylphosphine (PPh_3_), showed higher activity compared to all the nitrogen donors and the bidentate phosphine donors (Table 2, entries 6, 7, and 8, respectively). Gratifyingly, a much lower dispersity of 1.16 was achieved when PPh_3_ was used while not dramatically lowering the rate compared to the reactions without a neutral donor. In comparison, there was only a slight improvement to the polymerization rate when 1 equiv of PPh_3_ was added to the monocationic catalyst. When comparing the three monodentate phosphine donors, an important question arises about whether the excellent control demonstrated by the PPh_3_ is caused by a steric profile that may help deter aggregation of the dicationic catalyst, or by the electronics of the donor. In the case of P(*o*-tolyl)_3_, which would have a similar donor strength as PPh_3_, the steric profile appears to be too large (Tolman cone angle = 194°) for the donor to stabilize the catalyst, as evidenced by the fact that the dispersity and microstructure match the data where a donor was not added (Table 1, entry 2), and the discrepancy between the theoretical *M*_n_ and the experimental *M*_n_ is large.^37^ It was hypothesized that PPh_3_ was providing adequate steric/coordinative support to the catalyst without disrupting the Lewis acidity of the metal center or blocking access to the IP monomer. The Tolman cone angle of PCy_3_ (170°) represents a middle ground in size. The decrease in conversion and lowered dispersity indicates that PCy_3_ is directly impacting the catalyst activity. However, it is unclear if the lowered conversion, in comparison to PPh_3_, is due to the difference in steric crowding or donor strength of the ligands. Additionally, comparing the GPC traces of the three monodentate phosphine donors, when PPh_3_ is used, the light scattering trace appears more unimodal (Figure S310).

**Figure 2:**
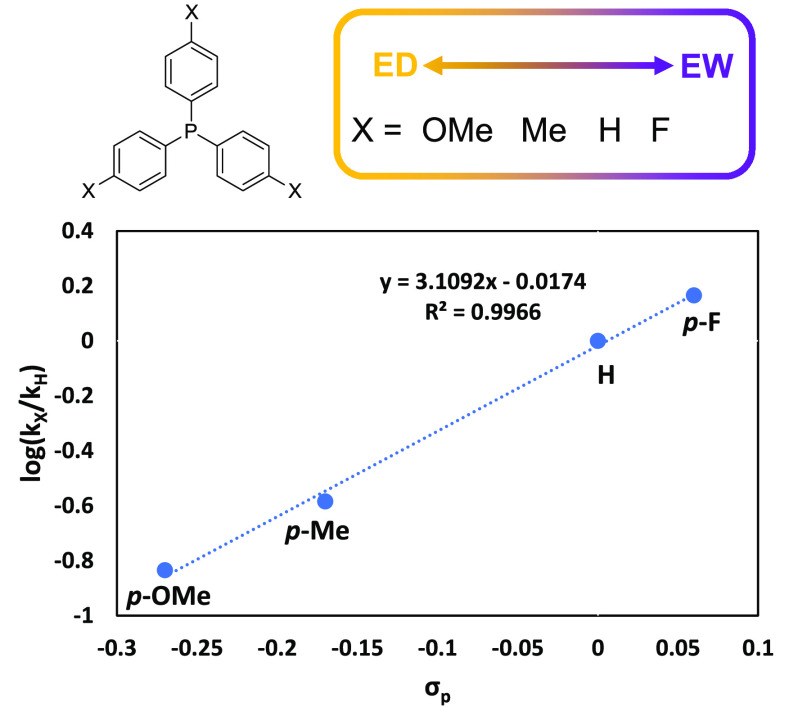
Hammett plot of log (*k*_*x*_/*k*_H_) versus the standard σ constants for the substituent. Reactions run analogous to those in Table 2.

**Figure 3:**
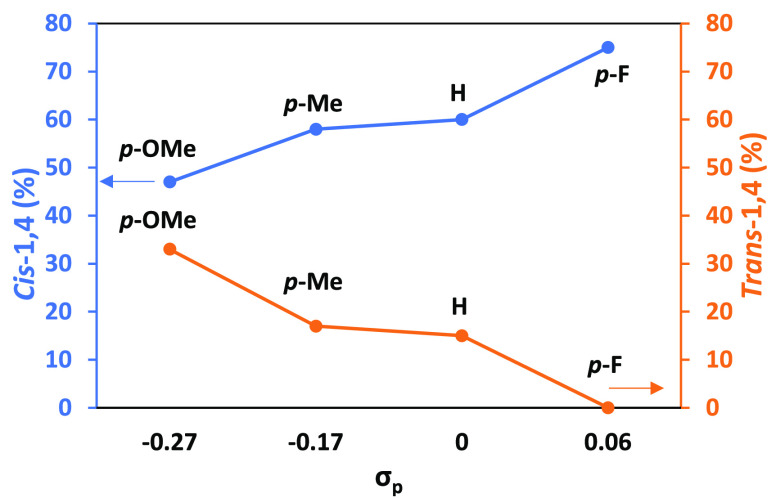
Comparison of *cis*-1,4 and *trans*-1,4 selectivity versus the standard σ constants for the different substituents. Reactions run analogous to those in Table 2.

In order to isolate the electronic effects of the PPh_3_ donor, a Hammett plot of log (*k*_X_/*k*_H_) versus the standard σ constants for para-substituents on the phenyl rings was constructed (Figure 2).^38^ Three commercially available para-X-substituted PPh_3_ (X = OMe, CH_3_, F) were tested for their efficacy as donor supports for the polymerization of IP with Y(CH_2_SiMe_3_)_3_(THF)_2_/2[Ph_3_C][B(C_6_F_5_)_4_] catalyst system, using reaction conditions identical to those used for Table 2. As hypothesized, electron-withdrawing groups enhanced the rate of polymerization as the weaker donors would be expected to not lower the Lewis acidity of the metal ion. A linear relationship was extrapolated from the Hammett plot with a positive slope of 3.11, indicating that the reaction is sensitive to electronic changes i.e., negative charge is built, or positive charge is lost during the reaction. Information on the conversion and number-average molar mass (*M*_n_) can be found in the SI (Table S1). The effect of the donor substituents on the selectivity of the PIP microstructure was also examined (Figure 3). It was found that the 3,4 selectivity remained unchanged for each donor, which is expected as 3,4 selectivity is generally dependent on the steric crowding around the metal center. However, higher *cis*-1,4 content was seen with more electron-withdrawing substituents where full *cis*-1,4 versus *trans*-1,4 selectivity was achieved with the para-fluorinated substituent. Importantly, while the *p*-F substituent enhanced both the rate and the *cis*-1,4 selectivity, PPh_3_ still demonstrated superior *M*_n_ and dispersity control (Table S1, entry 4 and Table 2, entry 8, respectively). This might be because the donor could have a weaker bond to the metal center, thus losing some of the stabilizing effect of the donor. This highlights the need to design ligand frameworks that are modulated to provide sufficient support to stabilize the complex without sacrificing the Lewis acidity of the metal center.

**Figure 4:**
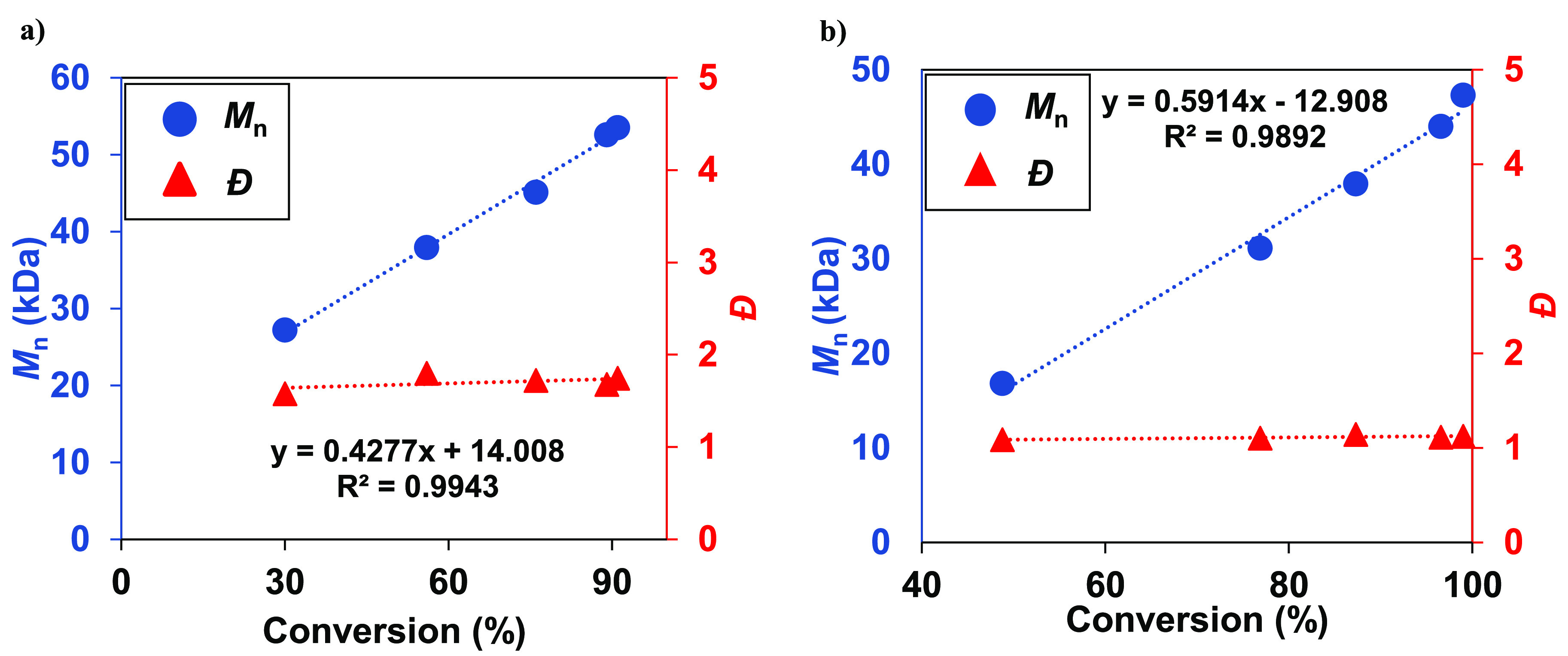
*M*_n_ vs conversion graphs for IP polymerization with Y(CH_2_SiMe_3_)_3_(THF)_2_ and 2 equiv [Ph_3_C][B(C_6_F_5_)_4_]; (a) without PPh_3_; (b) with 1 equiv PPh_3_.

In order to further examine the effect of the PPh_3_ on the polymerization of IP, a dynamic study of the Y(CH_2_SiMe_3_)_3_(THF)_2_ activated by 2 equiv [Ph_3_C][B(C_6_F_5_)_4_] was carried out both without PPh_3_ and with inclusion of PPh_3_ (Figure 4a and b, respectively). Gratifyingly, in the case where PPh_3_ was added the *M*_n_ increased linearly with conversion while the dispersity remained unchanged (*Đ* ≈ 1), indicating that the polymerization had living character. However, in the reaction without a donor, the dispersity was broad (>1.5) and increased early in the reaction. This further highlights the stability and control that the PPh_3_ adds to the polymerization. Information on the selectivity of the microstructure can be found in the SI (Tables S2 and S3).

We hypothesized that the phosphine is loosely binding to the metal center, which is causing the added dispersity control. Since the electron-withdrawing groups increases the polymerization rate, it appears that the loose binding is important to maintain catalytic activity. NMR studies were conducted to characterize the active catalyst with PPh_3_. Therefore, we activated Y(CH_2_SiMe_3_)_3_(THF)_2_ with 2 equiv of [Ph_3_C][B(C_6_F_5_)_4_] for 0, 10, and 30 min before adding 1 equiv of PPh_3_, and collected ^31^P NMR spectra over time. In all three cases, two ^31^P signals show up at 24.18 and 23.84. Over time the growth of a signal at −5.28 ppm is observed. As this solution is left over time, solids form and the signals around 24 ppm disappear (Figure S411). A control reaction of [Ph_3_C][B(C_6_F_5_)_4_] and PPh_3_ reveals the same two ^31^P signal around 24.18 and 23.84 as well as several small upfield peaks between (6.50–5.50). Over time a single peak around −6.48 is observed and the formation of solids in the NMR tube is seen (Figure S412). As the signal at −5.28 is very close to free PPh_3_ and the lack of a doublet suggests weak, fluxional, or no interaction with yttrium, we reasoned that by cooling the reaction we could resolve the peaks. Variable temperature (VT) NMR was conducted on Y(CH_2_SiMe_3_)_3_(THF)_2_ with 2 equiv of [Ph_3_C][B(C_6_F_5_)_4_] for 10 min before adding one equivalent of PPh_3_; however, even at a temperature as low as −80 °C, the spectrum only showed a singlet ^31^P signal (Figure S413). This might not be entirely surprising due to the mismatch in polarizability between the hard Lewis acid Y center and the soft Lewis base PPh_3_. Indeed, Anwander et al. crystallized the neutral complex Y(CH_2_SiMe_3_)_3_THF(dmpe) (dmpe = 1,2-bis(dimethylphosphino)ethane); however, they did not observe a doublet in the ^31^P NMR at room temperature or at −80 °C.^39^ Therefore, while PPh_3_ clearly impacts the polymerization by significantly lowering the dispersity, it cannot be conclusively confirmed to be binding to the metal ion. In another attempt to confirm if the PPh_3_ was bound to the yttrium metal center, DOSY NMR was taken before and after PPh_3_ addition. In both cases, a single diffusion coefficient is observed, indicating a single species in solution (Figures S414 and S415). This provides support that the PPh_3_ is interacting with the yttrium active species.

### Degree of Activation

We next investigated the effect of varying the equivalents of [Ph_3_C][B(C_6_F_5_)_4_] on IP polymerization, both with and without PPh_3_. First, with only 0.5 equiv of [Ph_3_C][B(C_6_F_5_)_4_], Y(CH_2_SiMe_3_)_3_(THF)_2_ was inactive toward IP polymerization. We then gradually increased the amount of [Ph_3_C][B(C_6_F_5_)_4_] in 0.5 equiv increments up to 3 equiv for separate IP polymerizations (Table 3). With 1 equiv of [Ph_3_C][B(C_6_F_5_)_4_], both with and without PPh_3_, we observed only about 70% conversion within 7 h, whereas higher equivalents reached full conversion within the same time frame. Additionally, with 1 equiv of [Ph_3_C][B(C_6_F_5_)_4_], we observed that the experimental *M*_n_ was consistent with two active alkyl initiators, now that higher conversion had been reached. As the amount of [Ph_3_C][B(C_6_F_5_)_4_] was increased, the molar mass increased relative to the theoretical molar mass values for either one or two active alkyl initiators. This is consistent with the idea that, with fewer alkyls to stabilize the metal center, some of the catalysts would begin to decompose before polymerization. The discrepancy between the experimental *M*_n_ and the theoretical *M*_n_ was decreased in the cases where PPh_3_ was added, indicating that PPh_3_ helps to stabilize the active catalyst. In addition to improving the molar mass, PPh_3_ significantly reduced the dispersity. As the amount of [Ph_3_C][B(C_6_F_5_)_4_] is increased, the more the light scattering GPC traces deviate from a unimodal distribution indicating multiple catalyst environments (Figure S336, S338, and S340). In each case, with the addition of PPh_3_ the light scattering GPC traces appear more unimodal.

**Table 3:**
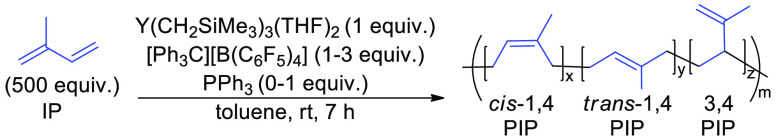
Varying Equivalents of **[Ph**_**3**_**C][B(C**_**6**_**F**_**5**_**)**_**4**_**]** in the Homopolymerization of IP with **Y(CH**_**2**_**SiMe**_**3**_**)**_**3**_**(THF)**_**2**_^a^

entry	PPh_3_ (P) (equiv)	borate (B) (equiv)	order of addition	conv. (%)^b^	^Theor^*M*_n_ (KDa) (2 Inr/1 Inr)^c^	^Exp^*M*_n_ (KDa)^d^	*Đ*^d^	microstructure^e^*cis*-1,4/ *trans*-1,4/3,4
1	0	1		72(2)	12/24	18(1)	1.29(7)	13/74/13
2	1	1	B → P	70(3)	12/24	15(4)	1.21(4)	12/75/13
3	0	1.5		>99	17/34	49(8)	1.6(10)	70/6/24
4	1	1.5	B → P	>99	17/34	25(5)	1.33(8)	66/7/27
5	0	2		>99	17/34	57(6)	1.98(6)	83/0/17
6	1	2	B → P	>99	17/34	46(5)	1.18(1)	61/13/26
7	0	2.5		>99	17/34	56(3)	1.96(7)	84/0/16
8	1	2.5	B → P	>99	17/34	44(2)	1.26(3)	64/11/25
9	0	3		>99	17/34	148(7)	3.89(10)	86/0/14
10	1	3	B → P	>99	17/34	67(4)	1.41(8)	82/0/18

a Conditions: Y(CH_2_SiMe_3_)_3_(THF)_2_, 10 μmol; [Ph_3_C][B(C_6_F_5_)_4_] (B), 10–30 μmol; toluene, 10 mL; 0–10 μmol PPh_3_ (P); [IP]/**Y** = 500. All entries are done in duplicate and the error is denoted in parentheses.

b Determined by ^1^H NMR spectroscopy of crude reaction mixtures, comparing monomer peaks to polymer.

c Calculated based on 1 or 2 alkyl initiators, [IP mol/RE mol] × IP molar mass × Conversion. Inr = alkyl initiator.

d Determined by gel permeation chromatography (GPC) in THF using a Wyatt DAWN HELEOS II MALS detector.

e All selectivity data is an average of duplicate runs. 1,4 and 3,4 selectivity determined by ^1^H NMR. *Cis*-1,4 and *trans*-1,4 selectivity determined by ^13^C NMR.

Surprisingly, we observe polymerization activity even with 3 equiv of [Ph_3_C][B(C_6_F_5_)_4_]. We have noted in a previous study that an impurity is present in the [Ph_3_C][B(C_6_F_5_)_4_] which could mean that we could be adding less [Ph_3_C][B(C_6_F_5_)_4_] than we are expecting.^11^ Additionally, we only react the [Ph_3_C][B(C_6_F_5_)_4_] with Y(CH_2_SiMe_3_)_3_(THF)_2_ for 10 min prior to IP addition, which could lead to incomplete activation. However, Okuda and co-workers were not able to abstract the third alkyl of Y(CH_2_SiMe_3_)_3_(THF)_2_, even with 5 equiv of [NMe_2_HPh][B(C_6_F_5_)_4_].^25^ In a later study, Okuda and co- workers were able to see NMR evidence of an alkyl-free complex by reacting Y(CH_2_SiMe_3_)_3_(12-crown-4), 3 equiv of [NMe_2_HPh][B(C_6_F_5_)_4_], and 1 equiv 12-crown-4 for 1 h.^27^ Even though in the present study it is difficult to discern if the third alkyl is being abstracted or initiator/precatalyst deactivation occurs before isoprene addition, the *M*_n_ of polyisoprene continues to increase as [Ph_3_C][B(C_6_F_5_)_4_] equivalence is increased (Table 3). An NMR scale reaction between Y(CH_2_SiMe_3_)_3_(THF)_2_ with 3 equiv of [Ph_3_C][B(C_6_F_5_)_4_] was conducted to identify if the third alkyl is being abstracted. The ^1^H spectra showed resonances due to Y-CH_2_ methylene groups (δ = −0.86, −1.05(d, *J*_YH_ = 3.4), −1.16). (Figure S419). Only one of the signals showed a clear doublet at a chemical shift value consistent with the Y-CH_2_ peak of the dicationic species. But it is worth noting that the neutral complex in toluene-*d*_8_ appears as a broad singlet, so the other two upfield signals might also be attributable to Y-CH_2_ interactions. This indicates that within the time scale of these polymerizations not all the alkyls are being abstracted.

### IP Polymerization with Ternary Systems

The addition of a cocatalyst is often used to promote better selectivity with RE metal alkyl catalysts. We tested the influence of three commonly used aluminum alkylating agents as cocatalysts for IP polymerization: AlMe_3_, AlEt_3_, and Al^*i*^Bu_3_ (Table 4). These reactions were carried out by combining Y(CH_2_SiMe_3_)_3_(THF)_2_ with 2 equiv of [Ph_3_C][B(C_6_F_5_)_4_] for 10 min, followed by the addition of the desired aluminum alkylating agent. After 10 min of mixing, 500 equiv of IP were added. The aluminum alkyls form varied steric environments and different conformations around the Y metal center, which will impact the rate and selectivity of isoprene polymerization. The bulkier alkylating agents allowed for faster IP polymerization rates (Al^*i*^Bu_3_ > AlEt_3_ > AlMe_3_). When Al^*i*^Bu_3_ was used, full conversion was reached within 30 min. The enhanced rate with the bulkier cocatalysts parallels other reports, where the aluminum cocatalysts with shorter alkyl chains can form heterobimetallic RE-Al alkyl species that slow down activity.^40,41^ As the ratio between the alkylating agent and catalyst increased, a drop in *M*_n_ and a significant broadening of the dispersity (Table 4, entries 7–9) were observed, indicating that Al^*i*^Bu_3_ was acting as a chain transfer agent. As the equivalents of alkylating agent increased, the conversion decreased, suggesting that chain transfer was competitive with polymer propagation. Dispersity also increased with higher alkylating equivalents, further indicating competition between chain transfer and propagation. After Al^*i*^Bu_3_ demonstrated the fastest rate, we wanted to optimize the reaction and speculated that adding PPh_3_ could aid in lowering the dispersity, just as it did without an alkylating agent. PPh_3_ was incorporated into the reaction after 10 min of activation of Y(CH_2_SiMe_3_)_3_(THF)_2_ with 2 equiv of [Ph_3_C][B(C_6_F_5_)_4_], and 5–15 equiv of Al^*i*^Bu_3_ were added after 10 min of stirring. With 5 and 10 equiv of Al^*i*^Bu_3_, the reaction reached full conversion, but at 15 equiv, the rate decreased. This could be due to the increased steric crowding around the metal center. Overall, reactions with PPh_3_ lowered the *M*_n_ and slightly decreased the dispersity (Table 4, entries 10–12). The *cis*-1,4 selectivity was enhanced by adding Al^*i*^Bu_3_, with the incorporation of PPh_3_ only slightly decreasing the *cis*-1,4 selectivity. In general, the use of a cocatalyst exhibited higher dispersities and, in the cases with AlEt_3_ and AlMe_3_, a loss in activity.

**Table 4:**
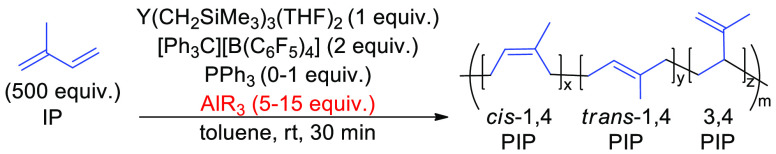
Aluminum Alkyl Additives in the Homopolymerization of IP with **Y(CH**_**2**_**SiMe**_**3**_**)**_**3**_**(THF)**_**2**_^a^

entry	PPh_3_ (P)	alkylating agent (AlR_3_)	AlR_3_ (equiv)	conv. (%)^b^	*M*_n_ (KDa)^c^	*Đ*^c^	microstructure^d^*cis*-1,4/ *trans*-1,4/3,4
1	0	AlMe_3_	5	68	68	1.49	54/42/4
2	0	AlMe_3_	10	78	43	1.49	56/37/7
3	0	AlMe_3_	15	49	39	1.81	58/37/5
4	0	AlEt_3_	5	90	25	3.31	64/19/17
5	0	AlEt_3_	10	84	26	4.78	63/21/16
6	0	AlEt_3_	15	80	22	6.41	65/21/14
7	0	Al^*i*^Bu_3_	5	>99	59	3.69	78/2/20
8	0	Al^*i*^Bu_3_	10	>99	46	4.69	84/3/13
9	0	Al^*i*^Bu_3_	15	>99	37	6.38	85/2/13
10	1	Al^*i*^Bu_3_	5	>99	42	3.07	73/4/23
11	1	Al^*i*^Bu_3_	10	>99	40	2.29	71/5/24
12	1	Al^*i*^Bu_3_	15	86	28	4.15	71/7/22

a Conditions: Y(CH_2_SiMe_3_)_3_(THF)_2_, 10 μmol; [Ph_3_C][B(C_6_F_5_)_4_] (B), 20 μmol; toluene, 10 mL; 0–10 μmol, PPh_3_; [IP]/**Y** = 500.

b Determined by ^1^H NMR spectroscopy of crude reaction mixtures, comparing monomer peaks to polymer.

c Determined by gel permeation chromatography (GPC) in THF using a Wyatt DAWN HELEOS II MALS detector.

d 1,4 and 3,4 selectivity determined by ^1^H NMR. *Cis*-1,4 and *trans*-1,4 selectivity determined by ^13^C NMR.

NMR studies were conducted to better understand the loss of activity with AlMe_3_ despite the better dispersity control it displayed. These were carried out by first reacting Y(CH_2_SiMe_3_)_3_(THF)_2_ with 2 equiv of [Ph_3_C][B(C_6_F_5_)_4_] for 10 min. Five equivalents of AlMe_3_ were then added and ^1^H and ^27^Al NMR spectra were taken at room temperature in toluene-*d*_*8*_ (Figures S420 and S421, respectively). The ^1^H NMR showed multiple peaks between −0.66 ppm to −1.13 ppm indicating multiple CH_3_ species. Similarly, Cui and co-workers observed the presence of multiple peaks in the yttrium alkyl region which they attributed to the asymmetric η^1^ binding of the tetramethylaluminum ligand.^42^ Interestingly, despite activation with two equivalents of [Ph_3_C][B(C_6_F_5_)_4_], a doublet around −0.67 ppm is observed consistent with the Y-CH_2_ monocationic species while the peak associated with the dicationic species at −1.08 ppm is present but at a lower intensity. So, the polymerization is hypothesized to proceed through a monocationic species or a lower concentration of a dicationic species, thus accounting for the reduced activity. The abundance of the monocationic species could be attributed to ligand rearrangement. Also, a previous report has shown that AlMe_3_ species do react with [Ph_3_C][B(C_6_F_5_)_4_] forming [AlR_2_]^+^ which will subsequently degrade the [B(C_6_F_5_)_4_]^−^ into AlMe_3–*x*_(C_6_F_5_)_*x*_ species.^43^ This is unlikely to occur with AlMe_3_ as elevated temperatures were required in that case; however, this could be taking place with the other AlR_3_ species containing β-hydrogens, which can decompose [Ph_3_C][B(C_6_F_5_)_4_] under ambient conditions. ^27^Al NMR shows two broad signals, one at 157 ppm which is consistent with a monocationic [AlR_4_]^−^ previously described by Okuda and co-workers.^44^ The second broad signal appears between 37 and 78 ppm which falls in between the 5-coordinate Al region (30–50 ppm) and the 4-coordinate Al region (60–70 ppm), which could indicate a high degree of dynamic binding behavior.^45^ These fast interactions are supported by what is observed in the polymerization experiments, where adding additional equivalents of AlMe_3_ (10 or 15 equiv) lead to the reduction in polymer molar mass giving indication of facile chain transfer. NMR studies were not conducted with either AlEt_3_ or Al^*i*^Bu_3_ as polymerizations with either showed exceptionally poor dispersities which increased with further additions of AlR_3_ (3.07–6.41). This could be due to the added steric bulk by the longer alkyl chains which block access to coordination sites. Indeed, the light scattering GPC trace with the addition of AlEt_3_ shows multimodal distribution indicating multiple different active sites. This is coupled with the fact that the experimental molar mass does not decrease with higher AlEt_3_ indicating that chain transfer is not facile. In the case with Al^*i*^Bu_3_, these ligands have an even larger steric profile, but the light scattering GPC traces show a mostly unimodal distribution especially with higher AlR_3_ equivalents. This is accounted for by the enhanced chain transfer ability of this Lewis acid. Upon inclusion of PPh_3_ into the reaction with Al^*i*^Bu_3_, chain transfer is seen but to a lesser extent. However, the light scattering GPC trace is multimodal indicating that the PPh_3_ could be preventing some chain transfer. The increased dispersity matches results found for the yttrium methyl dicationic species, which showed higher dispersity in the presence of Al^*i*^Bu_3_.^30^ This contrasts with the studies done by Boisson and co-workers where polymerization activity required the addition of Al cocatalysts.

### Extension to Other Rare Earth Metal Precatalysts

Traditionally, the faster catalysts for olefin and IP polymerization favor the smallest RE metals, Sc and Lu, which unfortunately are the most expensive and least abundant. In contrast, larger metals such as La are often inactive or show much slower diene polymerization compared to analogous complexes with small metals.^46^ With the proposed simple system, using accessible RE(CH_2_SiMe_3_)_3_(THF)_*n*_ (RE = Sc, Lu, Tm, Yb, Y, Er, Ho, Dy, Tb, Gd, *n* = 2; RE = Sm, La, *n* = 3), we sought to investigate whether neutral donors could support active and controlled catalysis for other RE metals, particularly the larger, less expensive ones. Therefore, polymerizations of IP were pursued for RE(CH_2_SiMe_3_)_3_(THF)_*n*_ with Sm, Gd, and Tm, representing large, medium, and small RE ions, respectively, and compared to those of Y (Table 5).^47,31^ Previous studies of RE(CH_2_SiMe_3_)_3_(THF)_*n*_ using 5 equiv of [NMe_2_HPh][B(C_6_F_5_)_4_] and 200 equiv of Al^*i*^Bu_3_ showed active catalysts for ethylene polymerization for a range of metals from Tb to Tm, while the smallest metals Sc, Lu, and Yb showed minimal polymerization activity.^25^ In this case, the larger metals showed faster polymerization activity, while the smaller active metals showed slower catalysis. Nevertheless, the trends swapped for molar masses, with the smaller metals allowing for larger polymer molar masses to be synthesized.

**Table 5:**
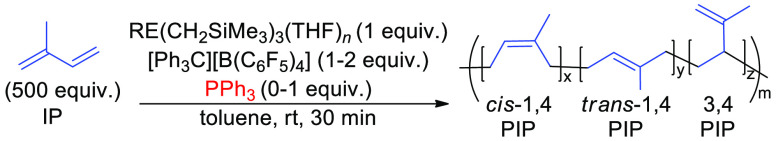
Polymerization of IP with RE(CH_2_SiMe_3_)_3_(THF*)*_*n*_ Precatalysts with and without PPh_3_^a^

entry	RE	borate (B) (equiv)	PPh_3_ (P) (equiv)	conv. (%)^b^	^Theor^*M*_n_ (kDa)^c^	^Exp^*M*_n_ (kDa)^d^	*Đ*^d^	microstructure^e^*cis*-1,4/*trans*-1,4/3,4
1	Sm	1	0	13(1)	4	8(2)	1.81(3)	23/67/10
2	Sm	2	0	30(5)	10	68(1)	1.58(2)	65/12/23
3	Gd	1	0	43(1)	15	32(2)	1.24(1)	58/17/25
4	Gd	2	0	63(5)	21	48(6)	2.23(28)	86/0/14
5	Y	1	0	20(1)	7	5(1)	1.44(5)	43/42/15
6	Y	2	0	>99	34	40(2)	2.04(2)	83/0/17
7	Tm	1	0	40(4)	14	20(1)	1.55(3)	39/46/15
8	Tm	2	0	47(2)	16	36(4)	2.67(10)	63/9/28
9	Sm	1	1	20(4)	7	10(2)	1.19(1)	23/67/10
10	Sm	2	1	45(1)	15	55(4)	1.33(2)	64/12/24
11	Gd	1	1	31(4)	11	15(1)	1.23(7)	28/56/16
12	Gd	2	1	88(1)	30	85(1)	1.10(4)	68/8/24
13	Y	1	1	15(1)	5	5(1)	1.27(4)	30/55/15
14	Y	2	1	76(3)	26	33(2)	1.16(6)	61/13/26
15	Tm	1	1	46(2)	16	22(6)	1.37(10)	39/46/15
16	Tm	2	1	67(2)	23	28(1)	1.29(1)	48/31/22

a Conditions: RE(CH_2_SiMe_3_)_3_(THF)*_n_* (RE = Sm, *n* = 3, RE = Gd, Y, Tm, *n* = 2), 10 μmol; [Ph_3_C][B(C_6_F_5_)_4_] (B),10–20 μmol; toluene, 10 mL; 0–10 μmol, PPh_3_; [IP]/RE = 500. All entries are done in duplicate and the error is denoted in parentheses.

b Determined by ^1^H NMR spectroscopy of crude reaction mixtures, comparing monomer peaks to polymer.

c Calculated based on one alkyl initiator, [IP mol/RE mol] × IP molar mass × Conversion.

d Determined by gel permeation chromatography (GPC) in THF using a Wyatt DAWN HELEOS II MALS detector.

e All selectivity data is an average of duplicate runs. 1,4 and 3,4 selectivity determined by ^1^H NMR. *Cis*-1,4 and *trans*-1,4 selectivity determined by ^13^C NMR.

The synthesized RE trialkyl precatalysts were activated with 1 or 2 equiv of [Ph_3_C][B(C_6_F_5_)_4_] 10 min prior to IP addition. IP polymerization was also carried out with the addition of the monodentate neutral donor, PPh_3_. In these reactions, 1 equiv of PPh_3_ was added to the monocationic or dicationic RE alkyl complex after 10 min activation time, and subsequent IP addition occurred 10 min after PPh_3_ was introduced to the reaction. The overall rate of polymerization was faster with 2 equiv of [Ph_3_C][B(C_6_F_5_)_4_] than with only 1 equiv for all precatalysts screened. The yttrium catalyst with 2 equiv of [Ph_3_C][B(C_6_F_5_)_4_] had the fastest rate, with complete IP conversion reached within 30 min.

Without the added support of a neutral donor, the dispersity remained high for all metals, regardless of how much [Ph_3_C][B(C_6_F_5_)_4_] was added. Additionally, with 1 equiv of [Ph_3_C][B(C_6_F_5_)_4_], the dispersity remained moderate (1.24–1.81) for all metals. Alternatively, with 2 equiv of [Ph_3_C][B(C_6_F_5_)_4_], the dispersity increased as the metal size decreased. This may be due to the more congested steric environment for the smaller metals, where IP coordination has an inhibitive effect on propagation. In most cases, the experimental molar mass was much higher than expected for one initiator per metal. This could be attributed to the possible decomposition of the catalyst during activation, but prior to IP addition, leaving fewer initiators than expected (vide infra).

When PPh_3_ is used, the dispersities of all polymerizations drop significantly, with no observable trends based on the metal size. Similar to the experiments without a donor, the rates of polymerization are faster when 2 equiv of [Ph_3_C][B(C_6_F_5_)_4_] are used instead of 1 equiv. When 2 equiv of [Ph_3_C][B(C_6_F_5_)_4_] are used with PPh_3_, the yttrium precatalyst shows consistent molar mass control similar to theoretical and also has a low dispersity of 1.16. The gadolinium catalyst shows the fastest rates and lower dispersity but shows poor molar mass control. The largest and smallest metals, Sm and Tm respectively, show the highest dispersities, which identifies the value in the RE metal series, allowing for subtle fine-tuning between metal size and Lewis acidity. This is contrary to what was seen previously for ethylene polymerization, as discussed above.^25^ However, the demonstrated polymerization of IP with large metals, such as Sm, with low dispersities near 1.3, opens opportunities to decrease the cost of selective and living diene polymerization, as the larger RE metals are known to be cheaper and more abundant.^48^

### Activation Conditions

When examining the IP polymerization using the Y(CH_2_SiMe_3_)_3_(THF)_2_/[Ph_3_C][B(C_6_F_5_)_4_]/donor system, there are many questions to be addressed. For instance, does the order in which the different components are added, or the time spaced between the addition of reagents affect the rate, selectivity, and molar mass control for the polymerization? In the previously discussed studies, the Y(CH_2_SiMe_3_)_3_(THF)_2_ was first activated with [Ph_3_C][B(C_6_F_5_)_4_], followed by addition of a neutral donor. Since polymer *M*_n_ was often slightly higher than theoretical, it was hypothesized that some catalyst might decompose prior to donor addition, leaving fewer initiators than expected. It was questioned whether donor presence prior to activation might prevent any decomposition of the proposed dicationic yttrium active catalyst. To test this hypothesis, experiments were carried out by first combining the precatalyst with PPh_3_ and then adjusting the time at which [Ph_3_C][B(C_6_F_5_)_4_] was added (Table 6). It was observed that for all precatalysts tested, insoluble materials appeared upon [Ph_3_C][B(C_6_F_5_)_4_] addition after PPh_3_, which was not present when [Ph_3_C][B(C_6_F_5_)_4_] was added first. This was attributed to a direct reaction between [Ph_3_C][B(C_6_F_5_)_4_] and PPh_3_, as discussed previously through NMR studies of catalyst activation.

**Table 6:**
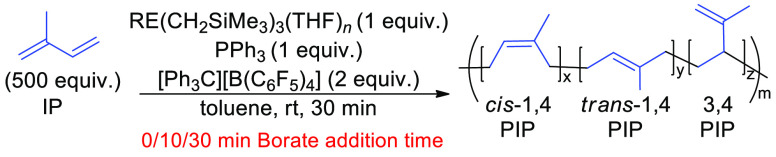
Addition Order in the Homopolymerization of IP with **RE(CH**_**2**_**SiMe**_**3**_**)**_**3**_**(THF)**_***n***_^a^

entry	RE	time before borate addition (min)	order of addition	conv. (%)^b^	^Theor^*M*_n_ (kDa)^c^	^Exp^*M*_n_ (kDa)^d^	*Đ*^d^	microstructure^e^*cis*-1,4/*trans*-1,4/3,4
1	Sm	0	PPh_3_→ B	23(5)	8	10(2)	1.24(8)	45/37/18
2	Sm	10	PPh_3_→ B	18(2)	6	13(4)	1.18(2)	53/28/19
3	Sm	30	PPh_3_→ B	18(1)	6	11(5)	1.21(5)	49/34/17
4	Gd	0	PPh_3_→ B	58(2)	20	25(6)	1.11(8)	63/16/21
5	Gd	10	PPh_3_→ B	64(5)	22	34(5)	1.09(3)	62/18/20
6	Gd	30	PPh_3_→ B	39(3)	13	23(3)	1.16(1)	55/24/21
7	Y	0	PPh_3_→ B	34(3)	11	7(3)	1.25(5)	38/43/19
8	Y	10	PPh_3_→ B	28(1)	10	8(2)	1.23(8)	41/40/19
9	Y	30	PPh_3_→ B	25(4)	9	7(3)	1.29(4)	38/43/19
10	Tm	0	PPh_3_→ B	36(2)	12	20(1)	1.29(6)	45/38/17
11	Tm	10	PPh_3_→ B	50(1)	17	24(3)	1.21(3)	47/35/18
12	Tm	30	PPh_3_→ B	43(3)	15	24(1)	1.25(7)	53/31/16

a Conditions: RE(CH_2_SiMe_3_)_3_(THF)*_n_* (RE = Sm, *n* = 3, RE = Gd, Y, Tm, *n* = 2), 10 μmol; 10 μmol, PPh_3_; [Ph_3_C][B(C_6_F_5_)_4_] (B), 20 μmol; toluene, 10 mL; [IP]/RE = 500. All entries are done in duplicate and the error is denoted in parentheses.

b Determined by ^1^H NMR spectroscopy of crude reaction mixtures, comparing monomer peaks to polymer.

c Calculated based on one alkyl initiator, [IP mol/RE mol] × IP molar mass × Conversion.

d Determined by gel permeation chromatography (GPC) in THF using a Wyatt DAWN HELEOS II MALS detector.

e All selectivity data is an average of duplicate runs. 1,4 and 3,4 selectivity determined by ^1^H NMR. *Cis*-1,4 and *trans*-1,4 selectivity determined by ^13^C NMR.

This suggested that there was likely less active catalyst in solution due to the interaction between PPh_3_ and the [Ph_3_C][B(C_6_F_5_)_4_] reagent which prevented the activation of the precatalysts. As expected for less catalyst, the conversion rates were severely diminished. However, the dispersity was not greatly affected by the change in addition order, which could suggest that the remaining active catalyst in solution is the same. In all cases, the conversion, *M*_n_, and dispersity seemed to closely resemble the data seen where only 1 equiv of [Ph_3_C][B(C_6_F_5_)_4_] was added prior to PPh_3_ (Table 5, entries 9, 11, 13, and 15, respectively). Changing the time between PPh_3_ addition and [Ph_3_C][B(C_6_F_5_)_4_] did not greatly impact the polymerization. Next, we adjusted the time between activation and addition of PPh_3_ (Table 7). For these trials, RE trialkyl precatalysts were activated with 2 equiv of [Ph_3_C][B(C_6_F_5_)_4_] and 1 equiv of PPh_3_ was added in three separate polymerizations at 0, 10, and 30 min after activation.

**Table 7:**
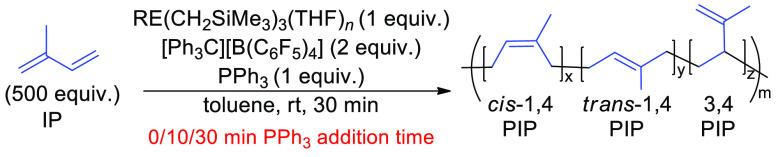
PPh_3_ Addition Time Variation in the Homopolymerization of IP with **RE(CH**_**2**_**SiMe**_**3**_**)**_**3**_**(THF*)*_*n*_**^a^

entry	RE	time before PPh_3_ addition (min)	total time before IP addition (min)	conv. (%)^b^	^Theor^*M*_n_ (kDa)^d^	^Exp^*M*_n_ (kDa)^d^	*Đ*^d^	microstructure^e^*cis*-1,4/*trans*-1,4/3,4
1	Sm	0	10	38(1)	13	22(4)	1.17(3)	53/27/20
2	Sm	10	20	45(1)	15	55(4)	1.33(2)	64/12/24
3	Sm	30	40	43(1)	15	64(3)	1.61(6)	31/7/24
4	Gd	0	10	74(1)	25	44(2)	1.18(5)	71/3/26
5	Gd	10	20	88(1)	30	85(1)	1.09(4)	71/7/23
6	Gd	30	40	90(1)	31	120(3)	1.09(1)	75/6/20
7	Y	0	10	31(1)	11	10(3)	1.20(3)	45/34/21
8	Y	10	20	76(3)	26	33(2)	1.16(6)	61/13/26
9	Y	30	40	>99	34	42(5)	1.18(1)	68/8/24
10	Tm	0	10	60(1)	20	21(2)	1.28(4)	46/33/21
11	Tm	10	20	67(2)	23	27(1)	1.2(6)	48/31/22
12	Tm	30	40	89(2)	30	32(3)	1.34(6)	54/21/25
13	Y		10	>99	34	40(2)	2.04(2)	83/0/17
14	Y		20	>99	34	55(5)	2.59(7)	74/1/25
15	Y		40	>99	34	100(7)	3.92(10)	77/2/21

a Conditions: RE(CH_2_SiMe_3_)_3_(THF)_*n*_ (RE = Sm, *n* = 3, RE = Gd, Y, Tm, *n* = 2), 10 μmol; [Ph_3_C][B(C_6_F_5_)_4_] (B), 20 μmol; 0–10 μmol PPh_3_; toluene, 10 mL; [IP]/RE = 500. All entries are done in duplicate and the error is denoted in parentheses.

b Determined by ^1^H NMR spectroscopy of crude reaction mixtures, comparing monomer peaks to polymer.

c Calculated based on one alkyl initiator, [IP mol/RE mol] × IP molar mass × Conversion.

d Determined by gel permeation chromatography (GPC) in THF using a Wyatt DAWN HELEOS II MALS detector.

e All selectivity data is an average of duplicate runs. 1,4 and 3,4 selectivity determined by ^1^H NMR. *Cis*-1,4 and *trans*-1,4 selectivity determined by ^13^C NMR.

In all cases, IP addition was kept constant by adding it to the reaction 10 min after PPh_3_ addition. For the smaller metals (Y and Tm), waiting a longer time before adding PPh_3_ increased the rate, while the corresponding conversions for Sm and Gd remained similarly stagnant. For the smaller metals, a longer activation time could allow the [Ph_3_C][B(C_6_F_5_)_4_] to fully react quantitatively to form the dicationic catalyst, leading to the enhanced rate. In contrast, the more coordinatively unstable dicationic congeners of Sm and Gd could decompose to a greater extent with a longer activation time frame in the absence of a neutral donor. Similarly, the experimental *M*_n_ increased relative to the theoretical *M*_n_ with increasing activation time, again more so for Sm and Gd than Y and Tm. Since this discrepancy in molar mass is characteristic of less active catalyst species than expected being present in solution, this trend is self-consistent with the decomposition of the dicationic complex without support from other ancillary ligands. However, since we have established living behavior, we hypothesize this decomposition occurs before IP addition. The discrepancy between the theoretical and the experimental *M*_n_ becomes more apparent as the ionic radius of the metal catalyst increases, which would be consistent with the fact that the larger dicationic species would be more unstable. To further demonstrate the role that PPh_3_ had in preventing the decomposition of the catalyst, analogous studies without the PPh_3_ were conducted (Table 7, entries 13–15). As the time before isoprene addition increased, the dispersity and the experimental *M*_n_ increased dramatically. This shows that the catalyst is decomposing as the time before IP addition is extended.

NMR experiments analogous to the conditions in Table 7 with the addition of PPh_3_ were conducted to better understand how the donor impacts the activation of the catalysts. Three experiments were carried out using Y(CH_2_SiMe_3_)_3_(THF)_2_ and 2 equiv of [Ph_3_C][B(C_6_F_5_)_4_]. PPh_3_ was then added at 0, 10, and 30 min after the addition of the [Ph_3_C][B(C_6_F_5_)_4_] with 0 min meaning that the donor, catalyst, and activator were added in at the same time. The different times of PPh_3_ addition showed an impact on the ^1^H NMR spectra. When adding the catalyst and the PPh_3_ together at the same time, the NMR shows a mixture of the monocationic and dicationic species (Figure S416). At the 10 min addition time, the ^1^H spectrum shows one distinct doublet at −1.10 ppm which was assigned to the Y-CH_2_ species which integrated to two relative to the THF signal (Figure S417). However, when waiting for 30 min before adding PPh_3_ the intensity of the Y-CH_2_ decreased relative to the THF peak and they appear as two singlets (Figure S418), indicating that waiting longer before addition of PPh_3_ led to less dicationic complex present in solution, thus demonstrating the stabilizing effect of PPh_3_. We also found experimentally that shorter activation times lead to slower polymerization rates. Also, an observation was made that at longer activation times, the molar mass increased dramatically for larger metals which might be due to the deactivation of the catalyst that were observed in the NMR spectra. One important thing to note is that the concentration of the catalyst is much higher in the NMR studies than in the polymerization conditions so any comparison may not be wholly representative. This is exemplified by the fact that in the NMR studies solids often appeared in the NMR tubes. Under standard catalytic conditions if full activation of the catalyst is achieved then no solids are seen.

### Stability of the Precatalyst

The stability of the Y(CH_2_SiMe_3_)_3_(THF)_2_/2[Ph_3_C][B(C_6_F_5_)_4_] system under catalysis conditions, both with and without addition of an in situ donor, was tested by sequential addition of IP (Table 8). Initially, 10 μmol Y(CH_2_SiMe_3_)_3_(THF)_2_ was stirred with 20 μmol [Ph_3_C][B(C_6_F_5_)_4_] for 10 min before adding 500 equiv of IP. In the case with the donor_,_ 10 μmol of PPh_3_ was first added, and the reaction was stirred for 10 min before adding IP. Reactions were stirred for 60 min and half the reaction was quenched and fully characterized with ^1^H, ^13^C NMR spectroscopy, and GPC analysis. A second portion of IP (250 equiv) was added and after 60 min of stirring, half of the reaction was again removed and characterized. The last step of the sequential polymerization was achieved by adding 125 equiv of IP, and then the entire reaction mixture was quenched and characterized after 60 min of stirring. Throughout the reaction with PPh_3_, the dispersity remains around 1.19 indicating no termination of chains during the sequence of polymerizations. The polymer molar mass also increased correspondingly, with excellent agreement between experimental and theoretical *M*_n_ values, and preservation of PIP microstructure. However, without a donor, both the dispersity and *M*_n_ increased significantly during the three separate IP additions, indicating a lack of catalyst stability between additions without the added support of the donor. While this by no means indicates that the catalyst could be isolated after polymerization, it does denote the stability of the catalyst under catalysis conditions, even when monomer is fully converted.

**Table 8:**
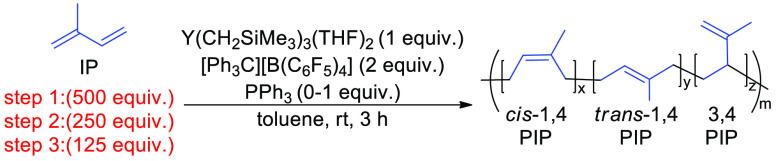
Sequential Polymerization of IP Using **Y(CH**_**2**_**SiMe**_**3**_**)**_**3**_**(THF)**_**2**_/**2[Ph**_**3**_**C][B(C**_**6**_**F**_**5**_**)**_**4**_**]** Both with and without PPh_3_^a^

entry	PPh_3_ (P) (equiv)	time (min)	IP addition (equiv)	conv. (%)^b^	^Theor^*M*_n_ (kDa)^c^	^Exp^*M*_n_ (KDa)^d^	*Đ*^d^	microstructure^e^*cis*-1,4/ *trans*-1,4/3,4
1	1	Step 1:60	500	>99	34	30	1.18	58/16/26
2		Step 2:60	250	>99	68	61	1.19	61/13/26
3		Step 3:60	125	>99	102	113	1.16	63/11/26
4	0	Step 1:60	500	>99	34	69	2.08	73/3/24
5		Step 2:60	250	>99	68	139	2.39	77/3/20
6		Step 3:60	125	>99	102	224	3.25	82/5/13

a Conditions: Y(CH_2_SiMe_3_)_3_(THF)_2_, 10 μmol; [Ph_3_C][B(C_6_F_5_)_4_] (B), 20 μmol; toluene, 10 mL; 0–10 μmol PPh_3_; [IP]/Y = 500 for each step.

b Determined by ^1^H NMR spectroscopy of crude reaction mixtures, comparing monomer peaks to polymer.

c Calculated based on one alkyl initiator, [IP mol/Y mol] × IP molar mass × Conversion.

d Determined by gel permeation chromatography (GPC) in THF using a Wyatt DAWN HELEOS II MALS detector.

e 1,4 and 3,4 selectivity determined by ^1^H NMR. *Cis*-1,4 and *trans*-1,4 selectivity determined by ^13^C NMR.

## Conclusions

**Figure 5:**
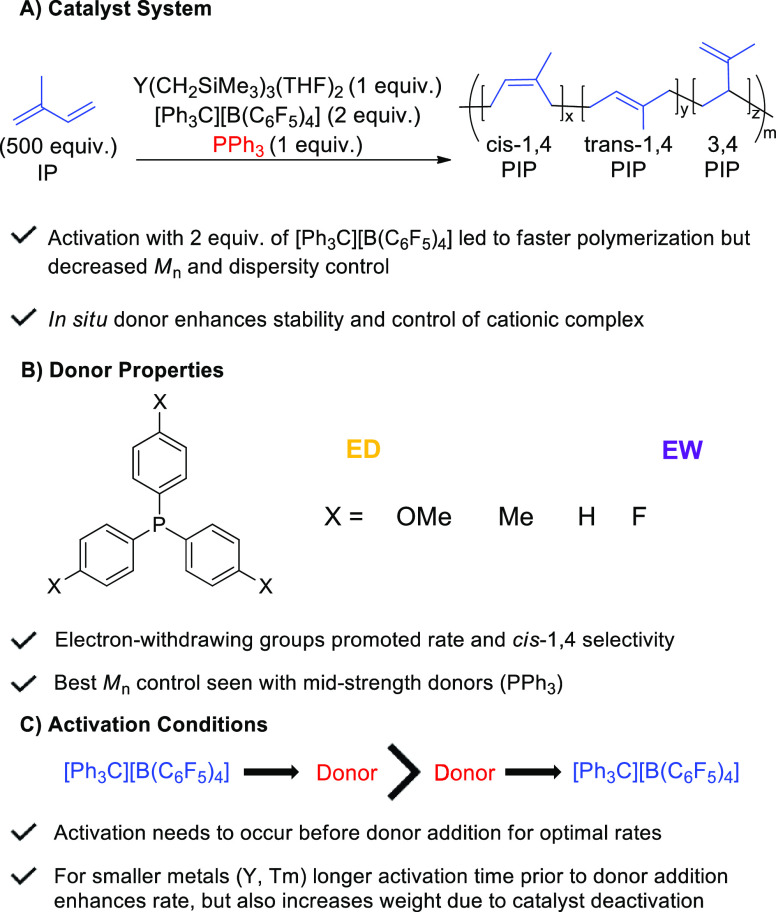
Summary of major findings of this work.

Herein, we have demonstrated that simple rare earth metal trialkyl complexes can be activated for rapid and living polymerization of isoprene when given the right activation conditions (Figure 5). Abstraction of two alkyls from the precatalysts, using [Ph_3_C][B(C_6_F_5_)_4_], followed by the addition of a weak field donor, PPh_3_, led to the best rate of polymerization and molar mass control (low dispersities and experimental *M*_n_ close to theoretical *M*_n_). While the yttrium precatalyst exhibited the best overall reactivity for polymerization, evidence suggests that polymerizations with larger metals (such as Gd and Sm), could lead to reduced catalyst cost and a more efficient transition to polar monomers, where large metals often excel.^49^ The rate and order of addition of all substrates greatly impacted the catalyst reactivity. Finally, in the presence of the donor, the catalyst demonstrates robust stability after polymerization is complete, as the addition of more IP showed continued polymerization with maintained low dispersity. These results suggest that proper reaction conditions can promote catalyst stability for the polymerization of isoprene, without the lengthy synthesis of designer catalysts. While these catalysts are only moderately selective for *cis*-1,4 polymerization of isoprene, these studies identify that the donor ligand can alter this selectivity. Future efforts will use strategies to adapt phosphine ligands to further improve this selectivity. Additionally, application of these methods to other monomers, including copolymerizations, is currently underway. Although soft, phosphine donors are used readily in late transition metal chemistry and catalysis, they are not commonly considered for use with hard early transition and rare earth metal ions. We hope these findings will add these soft donors as potential resources in early transition metal and rare earth metal catalysis.

## Supporting Information Available

All relevant data sets supporting this article including general considerations for chemicals, metal complexes, monomers and solvents, polymerization conditions and results, and characterization data (including ^1^H and ^13^C NMR spectra, FT-IR spectra, GPC spectra) (PDF)
Figures S1–S202, Nuclear magnetic resonance (NMR) characterization of isolated polymers (PDF)
Figure S202–S303, Fourier-transform infrared (FT-IR) spectroscopy characterization of isolated polymers (PDF)
Figures S304–S421, Gel permeation chromatography (GPC) characterization of isolated polymers (PDF)
