# Supplementary material for: Enhanced Control of Isoprene Polymerization with Trialkyl Rare Earth Metal Complexes through Neutral Donor Support
Source: Inorg Chem. 2023 Dec 8;63(21):9464–77. doi: 10.1021/acs.inorgchem.3c03161 (PMC11134520; doi:10.1021/acs.inorgchem.3c03161)
Supplement: Supplementary file 1 — ic3c03161_si_001.pdf [file ic3c03161_si_001.pdf]

Supporting Information

For

Enhanced Control of Isoprene Polymerization with Trialkyl Rare Earth Metal Complexes through Neutral Donor Support

Sophia C. Kosloski-Oh[a], Kai D. Knight[a] and Megan E. Fieser*[a,b]

[a]Department of Chemistry, University of Southern California, Los Angeles, California 90089

[b]Wrigley Institute for Environmental Studies, University of Southern California, Los Angeles, California 90089

Email: Fieser@usc.edu

*To whom correspondence should be addressed.

Table of Contents

**1.0**	**General Considerations**	**S25**
**1.1**	**Polymerization Methods**	**S25**
	Preparation of Stock Solutions for polymerizations	**S25**
	General procedure for the homopolymerization of isoprene	**S25**
**1.2**	**Characterization Methods**	**S26**
**1.3**	**Hammett plot**	**S27**
	**Table S1** IP polymerization with $\mathbf{Y(CH_2SiMe_3)_3(THF)_2}$, 2 equiv. $\mathbf{[Ph_3C][B(C_6F_5)_4]}$, and different para substituted donors.	**S27**
**1.4**	**Living Polymerization**	**S28**
	General procedure for time point studies	**S28**
	**Table S2** Living plot homopolymerization of IP with $\mathbf{Y(CH_2SiMe_3)_3(THF)_2}$.	**S28**
	**Table S3** Living plot homopolymerization of IP with $\mathbf{Y(CH_2SiMe_3)_3(THF)_2}$ and PPh_3.	**S29**
**1.5**	**Extended Reaction Times for IP Polymerization with RE Trialkyl Complexes**	**S30**
	**Table S4** Homopolymerization of IP with RE trialkyl pre-catalysts with and without PPh_3.	**S30**
**2.0**	**References**	**S31**
**3.0**	**Nuclear Magnetic Resonance (NMR) Characterization of Isolated Polymers**	**S32**
	**^{1}H NMR Spectroscopy**	**S32**
	**Fig. S1.** ^{1}H NMR spectrum of PIP 500 equivalents generated by $\mathbf{Y(CH_2SiMe_3)_3(THF)_2}$ and 1 equivalent $[Ph_3C][B(C_6F_5)_4]$ from **Table 1**, entry 1 in $CDCl_3$ at 298 K (30 min).	**S32**
	**Fig. S2.** ^{1}H NMR spectrum of PIP 500 equivalents generated by $\mathbf{Y(CH_2SiMe_3)_3(THF)_2}$ and 2 equivalents $[Ph_3C][B(C_6F_5)_4]$ from **Table 1**, entry 2 in $CDCl_3$ at 298 K (30 min).	**S32**
	**Fig. S3.** ^{1}H NMR spectrum of PIP 500 equivalents generated by $\mathbf{Y(CH_2SiMe_3)_3(THF)_2}$, 2 equivalents $[Ph_3C][B(C_6F_5)_4]$, and 1 equivalent Bipy from **Table 2**, entry 3 in $CDCl_3$ at 298 K (30 min).	**S33**
	**Fig. S4.** ^{1}H NMR spectrum of PIP 500 equivalents generated by $\mathbf{Y(CH_2SiMe_3)_3(THF)_2}$, 2 equivalents $[Ph_3C][B(C_6F_5)_4]$, and 1 equivalent MeCN from **Table 2**, entry 5 in $CDCl_3$ at 298 K (30 min).	**S33**
	**Fig. S5.** ^{1}H NMR spectrum of PIP 500 equivalents generated by $\mathbf{Y(CH_2SiMe_3)_3(THF)_2}$, 2 equivalents $[Ph_3C][B(C_6F_5)_4]$, and 1 equivalent P(*o*-tolyl)$_3$ from **Table 2**, entry 6 in $CDCl_3$ at 298 K (30 min).	**S34**
	**Fig. S6.** ^{1}H NMR spectrum of PIP 500 equivalents generated by $\mathbf{Y(CH_2SiMe_3)_3(THF)_2}$, 2 equivalents $[Ph_3C][B(C_6F_5)_4]$, and 1 equivalent PCy_3 from **Table 2**, entry 7 in $CDCl_3$ at 298 K (30 min).	**S34**

Fig. S7. ^{1}H NMR spectrum of PIP 500 equivalents generated by **$Y(CH_2SiMe_3)_3(THF)_2$**, 2 equivalents $[Ph_3C][B(C_6F_5)_4]$, and 1 equivalent PPh_3 from **Table 2**, entry 8 in $CDCl_3$ at 298 K (30 min). **S35**
Fig. S8. ^{1}H NMR spectrum of PIP 500 equivalents generated by **$Y(CH_2SiMe_3)_3(THF)_2$**, 2 equivalents $[Ph_3C][B(C_6F_5)_4]$, and 1 equivalent P(Ph-*p*-OMe)$_3$ from **Table S1**, entry 1 in $CDCl_3$ at 298 K (30 min). **S35**
Fig. S9. ^{1}H NMR spectrum of PIP 500 equivalents generated by **$Y(CH_2SiMe_3)_3(THF)_2$**, 2 equivalents $[Ph_3C][B(C_6F_5)_4]$, and 1 equivalent P(*p*-tolyl)$_3$ from **Table S1**, entry 2 in $CDCl_3$ at 298 K (30 min). **S36**
Fig. S10. ^{1}H NMR spectrum of PIP 500 equivalents generated by **$Y(CH_2SiMe_3)_3(THF)_2$**, 2 equivalents $[Ph_3C][B(C_6F_5)_4]$, and 1 equivalent P(Ph-*p*-F)$_3$ from **Table S1**, entry 4 in $CDCl_3$ at 298 K (30 min). **S36**
Fig. S11. ^{1}H NMR spectrum of PIP 500 equivalents generated by **$Y(CH_2SiMe_3)_3(THF)_2$** and 2 equivalents $[Ph_3C][B(C_6F_5)_4]$ from **Table S2**, entry 1 in $CDCl_3$ at 298 K (5 min). **S37**
Fig. S12. ^{1}H NMR spectrum of PIP 500 equivalents generated by **$Y(CH_2SiMe_3)_3(THF)_2$** and 2 equivalents $[Ph_3C][B(C_6F_5)_4]$ from **Table S2**, entry 2 in $CDCl_3$ at 298 K (12 min). **S37**
Fig. S13. ^{1}H NMR spectrum of PIP 500 equivalents generated by **$Y(CH_2SiMe_3)_3(THF)_2$** and 2 equivalents $[Ph_3C][B(C_6F_5)_4]$ from **Table S2**, entry 3 in $CDCl_3$ at 298 K (18 min). **S38**
Fig. S14. ^{1}H NMR spectrum of PIP 500 equivalents generated by **$Y(CH_2SiMe_3)_3(THF)_2$** and 2 equivalents $[Ph_3C][B(C_6F_5)_4]$ from **Table S2**, entry 4 in $CDCl_3$ at 298 K (24 min). **S38**
Fig. S15. ^{1}H NMR spectrum of PIP 500 equivalents generated by **$Y(CH_2SiMe_3)_3(THF)_2$** and 2 equivalents $[Ph_3C][B(C_6F_5)_4]$ from **Table S2**, entry 5 in $CDCl_3$ at 298 K (30 min). **S39**
Fig. S16. ^{1}H NMR spectrum of PIP 500 equivalents generated by **$Y(CH_2SiMe_3)_3(THF)_2$**, 2 equivalents $[Ph_3C][B(C_6F_5)_4]$, and 1 equivalent PPh_3 from **Table S3**, entry 1 in $CDCl_3$ at 298 K (10 min). **S39**
Fig. S17. ^{1}H NMR spectrum of PIP 500 equivalents generated by **$Y(CH_2SiMe_3)_3(THF)_2$**, 2 equivalents $[Ph_3C][B(C_6F_5)_4]$, and 1 equivalent PPh_3 from **Table S3**, entry 2 in $CDCl_3$ at 298 K (21 min). **S40**
Fig. S18. ^{1}H NMR spectrum of PIP 500 equivalents generated by **$Y(CH_2SiMe_3)_3(THF)_2$**, 2 equivalents $[Ph_3C][B(C_6F_5)_4]$, and 1 equivalent PPh_3 from **Table S3**, entry 3 in $CDCl_3$ at 298 K (31 min). **S40**
Fig. S19. ^{1}H NMR spectrum of PIP 500 equivalents generated by **$Y(CH_2SiMe_3)_3(THF)_2$**, 2 equivalents $[Ph_3C][B(C_6F_5)_4]$, and 1 equivalent PPh_3 from **Table S3**, entry 4 in $CDCl_3$ at 298 K (41 min). **S41**
Fig. S20. ^{1}H NMR spectrum of PIP 500 equivalents generated by **$Y(CH_2SiMe_3)_3(THF)_2$**, 2 equivalents $[Ph_3C][B(C_6F_5)_4]$, and 1 equivalent PPh_3 from **Table S3**, entry 5 in $CDCl_3$ at 298 K (51 min). **S41**
Fig. S21. ^{1}H NMR spectrum of PIP 500 equivalents generated by **$Sm(CH_2SiMe_3)_3(THF)_3$** and 1 equivalent $[Ph_3C][B(C_6F_5)_4]$ from **Table S4**, entry 1 in $CDCl_3$ at 298 K (7 h). **S42**
Fig. S22. ^{1}H NMR spectrum of PIP 500 equivalents generated by **$Gd(CH_2SiMe_3)_3(THF)_2$** and 1 equivalent $[Ph_3C][B(C_6F_5)_4]$ from **Table S4**, entry 2 in $CDCl_3$ at 298 K (7 h). **S42**
Fig. S23. ^{1}H NMR spectrum of PIP 500 equivalents generated by **$Gd(CH_2SiMe_3)_3(THF)_2$** and 2 equivalents $[Ph_3C][B(C_6F_5)_4]$ from **Table S4**, entry 3 in $CDCl_3$ at 298 K (7 h). **S43**
Fig. S24. ^{1}H NMR spectrum of PIP 500 equivalents generated by **$Tm(CH_2SiMe_3)_3(THF)_2$** and 1 equivalent $[Ph_3C][B(C_6F_5)_4]$ from **Table S4**, entry 4 in $CDCl_3$ at 298 K (7 h). **S43**
Fig. S25. ^{1}H NMR spectrum of PIP 500 equivalents generated by **$Tm(CH_2SiMe_3)_3(THF)_2$** and 2 equivalents $[Ph_3C][B(C_6F_5)_4]$ from **Table S4**, entry 5 in $CDCl_3$ at 298 K (7 h). **S44**
Fig. S26. ^{1}H NMR spectrum of PIP 500 equivalents generated by **$Sm(CH_2SiMe_3)_3(THF)_3$**, 1 equivalent $[Ph_3C][B(C_6F_5)_4]$, and 1 equivalent PPh_3 from **Table S4**, entry 6 in $CDCl_3$ at 298 K (7 h). **S44**

Fig. S27. ^{1}H NMR spectrum of PIP 500 equivalents generated by **$Gd(CH_2SiMe_3)_3(THF)_2$**, 1 equivalent $[Ph_3C][B(C_6F_5)_4]$, and 1 equivalent PPh_3 from **Table S4**, entry 7 in $CDCl_3$ at 298 K (7 h). **S45**
Fig. S28. ^{1}H NMR spectrum of PIP 500 equivalents generated by **$Gd(CH_2SiMe_3)_3(THF)_2$**, 2 equivalents $[Ph_3C][B(C_6F_5)_4]$, and 1 equivalent PPh_3 from **Table S4**, entry 8 in $CDCl_3$ at 298 K (7 h). **S45**
Fig. S29. ^{1}H NMR spectrum of PIP 500 equivalents generated by **$Tm(CH_2SiMe_3)_3(THF)_2$**, 1 equivalent $[Ph_3C][B(C_6F_5)_4]$, and 1 equivalent PPh_3 from **Table S4**, entry 9 in $CDCl_3$ at 298 K (7 h). **S46**
Fig. S30. ^{1}H NMR spectrum of PIP 500 equivalents generated by **$Tm(CH_2SiMe_3)_3(THF)_2$**, 2 equivalents $[Ph_3C][B(C_6F_5)_4]$, and 1 equivalent PPh_3 from **Table S4**, entry 10 in $CDCl_3$ at 298 K (7 h). **S46**
Fig. S31. ^{1}H NMR spectrum of PIP 500 equivalents generated by **$Y(CH_2SiMe_3)_3(THF)_2$** and 1 equivalent $[Ph_3C][B(C_6F_5)_4]$ from **Table 3**, entry 1 in $CDCl_3$ at 298 K (7 h). **S47**
Fig. S32. ^{1}H NMR spectrum of PIP 500 equivalents generated by **$Y(CH_2SiMe_3)_3(THF)_2$**, 1 equivalent $[Ph_3C][B(C_6F_5)_4]$, and 1 equivalent PPh_3 from **Table 3**, entry 2 in $CDCl_3$ at 298 K (7 h). **S47**
Fig. S33. ^{1}H NMR spectrum of PIP 500 equivalents generated by **$Y(CH_2SiMe_3)_3(THF)_2$** and 1.5 equivalents $[Ph_3C][B(C_6F_5)_4]$ from **Table 3**, entry 3 in $CDCl_3$ at 298 K (7 h). **S48**
Fig. S34. ^{1}H NMR spectrum of PIP 500 equivalents generated by **$Y(CH_2SiMe_3)_3(THF)_2$**, 1.5 equivalents $[Ph_3C][B(C_6F_5)_4]$, and 1 equivalent PPh_3 from **Table 3**, entry 4 in $CDCl_3$ at 298 K (7 h). **S48**
Fig. S35. ^{1}H NMR spectrum of PIP 500 equivalents generated by **$Y(CH_2SiMe_3)_3(THF)_2$** and 2 equivalents $[Ph_3C][B(C_6F_5)_4]$ from **Table 3**, entry 5 in $CDCl_3$ at 298 K (7 h). **S49**
Fig. S36. ^{1}H NMR spectrum of PIP 500 equivalents generated by **$Y(CH_2SiMe_3)_3(THF)_2$**, 2 equivalents $[Ph_3C][B(C_6F_5)_4]$, and 1 equivalent PPh_3 from **Table 3**, entry 6 in $CDCl_3$ at 298 K (7 h). **S49**
Fig. S37. ^{1}H NMR spectrum of PIP 500 equivalents generated by **$Y(CH_2SiMe_3)_3(THF)_2$** and 2.5 equivalents $[Ph_3C][B(C_6F_5)_4]$ from **Table 3**, entry 7 in $CDCl_3$ at 298 K (7 h). **S50**
Fig. S38. ^{1}H NMR spectrum of PIP 500 equivalents generated by **$Y(CH_2SiMe_3)_3(THF)_2$**, 2.5 equivalents $[Ph_3C][B(C_6F_5)_4]$, and 1 equivalent PPh_3 from **Table 3**, entry 8 in $CDCl_3$ at 298 K (7 h). **S50**
Fig. S39. ^{1}H NMR spectrum of PIP 500 equivalents generated by **$Y(CH_2SiMe_3)_3(THF)_2$** and 3 equivalents $[Ph_3C][B(C_6F_5)_4]$ from **Table 3**, entry 9 in $CDCl_3$ at 298 K (7 h). **S51**
Fig. S40. ^{1}H NMR spectrum of PIP 500 equivalents generated by **$Y(CH_2SiMe_3)_3(THF)_2$**, 3 equivalents $[Ph_3C][B(C_6F_5)_4]$, and 1 equivalent PPh_3 from **Table 3**, entry 10 in $CDCl_3$ at 298 K (7 h). **S51**
Fig. S41. ^{1}H NMR spectrum of PIP 500 equivalents generated by **$Y(CH_2SiMe_3)_3(THF)_2$**, 2 equivalents $[Ph_3C][B(C_6F_5)_4]$, and 5 equivalents $AlMe_3$ from **Table 4**, entry 1 in $CDCl_3$ at 298 K (30 min). **S52**
Fig. S42. ^{1}H NMR spectrum of PIP 500 equivalents generated by **$Y(CH_2SiMe_3)_3(THF)_2$**, 2 equivalents $[Ph_3C][B(C_6F_5)_4]$, and 10 equivalents $AlMe_3$ from **Table 4**, entry 2 in $CDCl_3$ at 298 K (30 min). **S52**
Fig. S43. ^{1}H NMR spectrum of PIP 500 equivalents generated by **$Y(CH_2SiMe_3)_3(THF)_2$**, 2 equivalents $[Ph_3C][B(C_6F_5)_4]$, and 15 equivalents $AlMe_3$ from **Table 4**, entry 3 in $CDCl_3$ at 298 K (30 min). **S53**
Fig. S44. ^{1}H NMR spectrum of PIP 500 equivalents generated by **$Y(CH_2SiMe_3)_3(THF)_2$**, 2 equivalents $[Ph_3C][B(C_6F_5)_4]$, and 5 equivalents $AlEt_3$ from **Table 4**, entry 4 in $CDCl_3$ at 298 K (30 min). **S53**

Fig. S45. ^{1}H NMR spectrum of PIP 500 equivalents generated by **$Y(CH_2SiMe_3)_3(THF)_2$**, 2 equivalents $[Ph_3C][B(C_6F_5)_4]$, and 10 equivalents $AlEt_3$ from **Table 4**, entry 5 in $CDCl_3$ at 298 K (30 min). S54
Fig. S46. ^{1}H NMR spectrum of PIP 500 equivalents generated by **$Y(CH_2SiMe_3)_3(THF)_2$**, 2 equivalents $[Ph_3C][B(C_6F_5)_4]$, and 15 equivalents $AlEt_3$ from **Table 4**, entry 6 in $CDCl_3$ at 298 K (30 min). S54
Fig. S47. ^{1}H NMR spectrum of PIP 500 equivalents generated by **$Y(CH_2SiMe_3)_3(THF)_2$**, 2 equivalents $[Ph_3C][B(C_6F_5)_4]$, and 5 equivalents Al^iBu_3 from **Table 4**, entry 7 in $CDCl_3$ at 298 K (30 min). S55
Fig. S48. ^{1}H NMR spectrum of PIP 500 equivalents generated by **$Y(CH_2SiMe_3)_3(THF)_2$**, 2 equivalents $[Ph_3C][B(C_6F_5)_4]$, and 10 equivalents Al^iBu_3 from **Table 4**, entry 8 in $CDCl_3$ at 298 K (30 min). S55
Fig. S49. ^{1}H NMR spectrum of PIP 500 equivalents generated by **$Y(CH_2SiMe_3)_3(THF)_2$**, 2 equivalents $[Ph_3C][B(C_6F_5)_4]$, and 15 equivalents Al^iBu_3 from **Table 4**, entry 9 in $CDCl_3$ at 298 K (30 min). S56
Fig. S50. ^{1}H NMR spectrum of PIP 500 equivalents generated by **$Y(CH_2SiMe_3)_3(THF)_2$**, 2 equivalents $[Ph_3C][B(C_6F_5)_4]$, 1 equivalent PPh_3, and 5 equivalents Al^iBu_3 from **Table 4**, entry 10 in $CDCl_3$ at 298 K (30 min). S56
Fig. S51. ^{1}H NMR spectrum of PIP 500 equivalents generated by **$Y(CH_2SiMe_3)_3(THF)_2$**, 2 equivalents $[Ph_3C][B(C_6F_5)_4]$, 1 equivalent PPh_3, and 10 equivalents Al^iBu_3 from **Table 4**, entry 11 in $CDCl_3$ at 298 K (30 min). S57
Fig. S52. ^{1}H NMR spectrum of PIP 500 equivalents generated by **$Y(CH_2SiMe_3)_3(THF)_2$**, 2 equivalents $[Ph_3C][B(C_6F_5)_4]$, 1 equivalent PPh_3, and 15 equivalents Al^iBu_3 from **Table 4**, entry 12 in $CDCl_3$ at 298 K (30 min). S57
Fig. S53. ^{1}H NMR spectrum of PIP 500 equivalents generated by **$Sm(CH_2SiMe_3)_3(THF)_3$**, 1 equivalent $[Ph_3C][B(C_6F_5)_4]$ from **Table 5**, entry 1 in $CDCl_3$ at 298 K (30 min). S58
Fig. S54. ^{1}H NMR spectrum of PIP 500 equivalents generated by **$Sm(CH_2SiMe_3)_3(THF)_3$**, 2 equivalents $[Ph_3C][B(C_6F_5)_4]$ from **Table 5**, entry 2 in $CDCl_3$ at 298 K (30 min). S58
Fig. S55. ^{1}H NMR spectrum of PIP 500 equivalents generated by **$Gd(CH_2SiMe_3)_3(THF)_2$**, 1 equivalent $[Ph_3C][B(C_6F_5)_4]$ from **Table 5**, entry 3 in $CDCl_3$ at 298 K (30 min). S59
Fig. S56. ^{1}H NMR spectrum of PIP 500 equivalents generated by **$Gd(CH_2SiMe_3)_3(THF)_2$**, 2 equivalents $[Ph_3C][B(C_6F_5)_4]$ from **Table 5**, entry 4 in $CDCl_3$ at 298 K (30 min). S59
Fig. S57. ^{1}H NMR spectrum of PIP 500 equivalents generated by **$Y(CH_2SiMe_3)_3(THF)_2$**, 1 equivalent $[Ph_3C][B(C_6F_5)_4]$ from **Table 5**, entry 5 in $CDCl_3$ at 298 K (30 min). S60
Fig. S58. ^{1}H NMR spectrum of PIP 500 equivalents generated by **$Y(CH_2SiMe_3)_3(THF)_2$**, 2 equivalents $[Ph_3C][B(C_6F_5)_4]$ from **Table 5**, entry 6 in $CDCl_3$ at 298 K (30 min). S60
Fig. S59. ^{1}H NMR spectrum of PIP 500 equivalents generated by **$Tm(CH_2SiMe_3)_3(THF)_2$**, 1 equivalent $[Ph_3C][B(C_6F_5)_4]$ from **Table 5**, entry 7 in $CDCl_3$ at 298 K (30 min). S61
Fig. S60. ^{1}H NMR spectrum of PIP 500 equivalents generated by **$Tm(CH_2SiMe_3)_3(THF)_2$**, 2 equivalents $[Ph_3C][B(C_6F_5)_4]$ from **Table 5**, entry 8 in $CDCl_3$ at 298 K (30 min). S61
Fig. S61. ^{1}H NMR spectrum of PIP 500 equivalents generated by **$Sm(CH_2SiMe_3)_3(THF)_3$**,1 equivalent $[Ph_3C][B(C_6F_5)_4]$, and 1 equivalent PPh_3 from **Table 5**, entry 9 in $CDCl_3$ at 298 K (30 min). S62
Fig. S62. ^{1}H NMR spectrum of PIP 500 equivalents generated by **$Sm(CH_2SiMe_3)_3(THF)_3$**, 2 equivalents $[Ph_3C][B(C_6F_5)_4]$, and 1 equivalent PPh_3 from **Table 5**, entry 10 in $CDCl_3$ at 298 K (30 min). S62
Fig. S63. ^{1}H NMR spectrum of PIP 500 equivalents generated by **$Gd(CH_2SiMe_3)_3(THF)_2$**, 1 equivalent $[Ph_3C][B(C_6F_5)_4]$, and 1 equivalent PPh_3 from **Table 5**, entry 11 in $CDCl_3$ at 298 K (30 min). S63

Fig. S64. ^{1}H NMR spectrum of PIP 500 equivalents generated by **$Gd(CH_2SiMe_3)_3(THF)_2$**, 2 equivalents $[Ph_3C][B(C_6F_5)_4]$, and 1 equivalent PPh_3 from **Table 5**, entry 12 in $CDCl_3$ at 298 K (30 min). **S63**

Fig. S65. ^{1}H NMR spectrum of PIP 500 equivalents generated by **$Y(CH_2SiMe_3)_3(THF)_2$**, 1 equivalent $[Ph_3C][B(C_6F_5)_4]$, and 1 equivalent PPh_3 from **Table 5**, entry 13 in $CDCl_3$ at 298 K (30 min). **S64**

Fig. S66. ^{1}H NMR spectrum of PIP 500 equivalents generated by **$Y(CH_2SiMe_3)_3(THF)_2$**, 2 equivalents $[Ph_3C][B(C_6F_5)_4]$, and 1 equivalent PPh_3 from **Table 5**, entry 14 in $CDCl_3$ at 298 K (30 min). **S64**

Fig. S67. ^{1}H NMR spectrum of PIP 500 equivalents generated by **$Tm(CH_2SiMe_3)_3(THF)_2$**, 1 equivalent $[Ph_3C][B(C_6F_5)_4]$, and 1 equivalent PPh_3 from **Table 5**, entry 15 in $CDCl_3$ at 298 K (30 min). **S65**

Fig. S68. ^{1}H NMR spectrum of PIP 500 equivalents generated by **$Tm(CH_2SiMe_3)_3(THF)_2$**, 2 equivalents $[Ph_3C][B(C_6F_5)_4]$, and 1 equivalent PPh_3 from **Table 5**, entry 16 in $CDCl_3$ at 298 K (30 min). **S65**

Fig. S69. ^{1}H NMR spectrum of PIP 500 equivalents generated by **$Sm(CH_2SiMe_3)_3(THF)_3$**, 1 equivalent PPh_3 and 2 equivalents $[Ph_3C][B(C_6F_5)_4]$ from **Table 6**, entry 1 in $CDCl_3$ at 298 K ($[Ph_3C][B(C_6F_5)_4]$ addition time 0 min). **S66**

Fig. S70. ^{1}H NMR spectrum of PIP 500 equivalents generated by **$Sm(CH_2SiMe_3)_3(THF)_3$**, 1 equivalent PPh_3, and 2 equivalents $[Ph_3C][B(C_6F_5)_4]$ from **Table 6**, entry 2 in $CDCl_3$ at 298 K ($[Ph_3C][B(C_6F_5)_4]$ addition time 10 min). **S66**

Fig. S71. ^{1}H NMR spectrum of PIP 500 equivalents generated by **$Sm(CH_2SiMe_3)_3(THF)_3$**, 1 equivalent PPh_3, and 2 equivalents $[Ph_3C][B(C_6F_5)_4]$ from **Table 6**, entry 3 in $CDCl_3$ at 298 K ($[Ph_3C][B(C_6F_5)_4]$ addition time 30 min). **S67**

Fig. S72. ^{1}H NMR spectrum of PIP 500 equivalents generated by **$Gd(CH_2SiMe_3)_3(THF)_2$**, 1 equivalent PPh_3 and 2 equivalents $[Ph_3C][B(C_6F_5)_4]$ from **Table 6**, entry 4 in $CDCl_3$ at 298 K ($[Ph_3C][B(C_6F_5)_4]$ addition time 0 min). **S67**

Fig. S73. ^{1}H NMR spectrum of PIP 500 equivalents generated by **$Gd(CH_2SiMe_3)_3(THF)_2$**, 1 equivalent PPh_3, and 2 equivalents $[Ph_3C][B(C_6F_5)_4]$ from **Table 6**, entry 5 in $CDCl_3$ at 298 K ($[Ph_3C][B(C_6F_5)_4]$ addition time 10 min). **S68**

Fig. S74. ^{1}H NMR spectrum of PIP 500 equivalents generated by **$Gd(CH_2SiMe_3)_3(THF)_2$**, 1 equivalent PPh_3, and 2 equivalents $[Ph_3C][B(C_6F_5)_4]$ from **Table 6**, entry 6 in $CDCl_3$ at 298 K ($[Ph_3C][B(C_6F_5)_4]$ addition time 30 min). **S68**

Fig. S75. ^{1}H NMR spectrum of PIP 500 equivalents generated by **$Y(CH_2SiMe_3)_3(THF)_2$**, 1 equivalent PPh_3, and 2 equivalents $[Ph_3C][B(C_6F_5)_4]$ from **Table 6**, entry 7 in $CDCl_3$ at 298 K ($[Ph_3C][B(C_6F_5)_4]$ addition time 0 min). **S69**

Fig. S76. ^{1}H NMR spectrum of PIP 500 equivalents generated by **$Y(CH_2SiMe_3)_3(THF)_2$**, 1 equivalent PPh_3, and 2 equivalents $[Ph_3C][B(C_6F_5)_4]$ from **Table 6**, entry 8 in $CDCl_3$ at 298 K ($[Ph_3C][B(C_6F_5)_4]$ addition time 10 min). **S69**

Fig. S77. ^{1}H NMR spectrum of PIP 500 equivalents generated by **$Y(CH_2SiMe_3)_3(THF)_2$**, 1 equivalent PPh_3, and 2 equivalents $[Ph_3C][B(C_6F_5)_4]$ from **Table 6**, entry 9 in $CDCl_3$ at 298 K ($[Ph_3C][B(C_6F_5)_4]$ addition time 30 min). **S70**

Fig. S78. ^{1}H NMR spectrum of PIP 500 equivalents generated by **$Tm(CH_2SiMe_3)_3(THF)_2$**, 1 equivalent PPh_3, and 2 equivalents $[Ph_3C][B(C_6F_5)_4]$ from **Table 6**, entry 10 in $CDCl_3$ at 298 K ($[Ph_3C][B(C_6F_5)_4]$ addition time 0 min). **S70**

Fig. S79. ^{1}H NMR spectrum of PIP 500 equivalents generated by **$Tm(CH_2SiMe_3)_3(THF)_2$**, 1 equivalent PPh_3, and 2 equivalents $[Ph_3C][B(C_6F_5)_4]$ from **Table 6**, entry 11 in $CDCl_3$ at 298 K ($[Ph_3C][B(C_6F_5)_4]$ addition time 10 min). **S71**

Fig. S80. ^{1}H NMR spectrum of PIP 500 equivalents generated by **$Tm(CH_2SiMe_3)_3(THF)_2$**, 1 equivalent PPh_3, and 2 equivalents $[Ph_3C][B(C_6F_5)_4]$ from **Table 6**, entry 12 in $CDCl_3$ at 298 K ($[Ph_3C][B(C_6F_5)_4]$ addition time 30 min). **S71**

Fig. S81. ^{1}H NMR spectrum of PIP 500 equivalents generated by **Sm(CH$_2$SiMe$_3$)$_3$(THF)$_3$**, 2 equivalents [Ph$_3$C][B(C$_6$F$_5$)$_4$], and 1 equivalent PPh$_3$ from **Table 7**, entry 1 in CDCl$_3$ at 298 K (PPh$_3$ addition time 0 min). **S72**
Fig. S82. ^{1}H NMR spectrum of PIP 500 equivalents generated by **Sm(CH$_2$SiMe$_3$)$_3$(THF)$_3$**, 2 equivalents [Ph$_3$C][B(C$_6$F$_5$)$_4$], and 1 equivalent PPh$_3$ from **Table 7**, entry 2 in CDCl$_3$ at 298 K (PPh$_3$ addition time 10 min). **S72**
Fig. S83. ^{1}H NMR spectrum of PIP 500 equivalents generated by **Sm(CH$_2$SiMe$_3$)$_3$(THF)$_3$**, 2 equivalents [Ph$_3$C][B(C$_6$F$_5$)$_4$], and 1 equivalent PPh$_3$ from **Table 7**, entry 3 in CDCl$_3$ at 298 K (PPh$_3$ addition time 30 min). **S73**
Fig. S84. ^{1}H NMR spectrum of PIP 500 equivalents generated by **Gd(CH$_2$SiMe$_3$)$_3$(THF)$_2$**, 2 equivalents [Ph$_3$C][B(C$_6$F$_5$)$_4$], and 1 equivalent PPh$_3$ from **Table 7**, entry 4 in CDCl$_3$ at 298 K (PPh$_3$ addition time 0 min). **S73**
Fig. S85. ^{1}H NMR spectrum of PIP 500 equivalents generated by **Gd(CH$_2$SiMe$_3$)$_3$(THF)$_2$**, 2 equivalents [Ph$_3$C][B(C$_6$F$_5$)$_4$], and 1 equivalent PPh$_3$ from **Table 7**, entry 5 in CDCl$_3$ at 298 K (PPh$_3$ addition time 10 min). **S74**
Fig. S86. ^{1}H NMR spectrum of PIP 500 equivalents generated by **Gd(CH$_2$SiMe$_3$)$_3$(THF)$_2$**, 2 equivalents [Ph$_3$C][B(C$_6$F$_5$)$_4$], and 1 equivalent PPh$_3$ from **Table 7**, entry 6 in CDCl$_3$ at 298 K (PPh$_3$ addition time 30 min). **S74**
Fig. S87. ^{1}H NMR spectrum of PIP 500 equivalents generated by **Y(CH$_2$SiMe$_3$)$_3$(THF)$_2$**, 2 equivalents [Ph$_3$C][B(C$_6$F$_5$)$_4$], and 1 equivalent PPh$_3$ from **Table 7**, entry 7 in CDCl$_3$ at 298 K (PPh$_3$ addition time 0 min). **S75**
Fig. S88. ^{1}H NMR spectrum of PIP 500 equivalents generated by **Y(CH$_2$SiMe$_3$)$_3$(THF)$_2$**, 2 equivalents [Ph$_3$C][B(C$_6$F$_5$)$_4$], and 1 equivalent PPh$_3$ from **Table 7**, entry 8 in CDCl$_3$ at 298 K (PPh$_3$ addition time 10 min). **S75**
Fig. S89. ^{1}H NMR spectrum of PIP 500 equivalents generated by **Y(CH$_2$SiMe$_3$)$_3$(THF)$_2$**, 2 equivalents [Ph$_3$C][B(C$_6$F$_5$)$_4$], and 1 equivalent PPh$_3$ from **Table 7**, entry 9 in CDCl$_3$ at 298 K (PPh$_3$ addition time 30 min). **S76**
Fig. S90. ^{1}H NMR spectrum of PIP 500 equivalents generated by **Tm(CH$_2$SiMe$_3$)$_3$(THF)$_2$**, 2 equivalents [Ph$_3$C][B(C$_6$F$_5$)$_4$], and 1 equivalent PPh$_3$ from **Table 7**, entry 10 in CDCl$_3$ at 298 K (PPh$_3$ addition time 0 min). **S76**
Fig. S91. ^{1}H NMR spectrum of PIP 500 equivalents generated by **Tm(CH$_2$SiMe$_3$)$_3$(THF)$_2$**-, 2 equivalents [Ph$_3$C][B(C$_6$F$_5$)$_4$], and 1 equivalent PPh$_3$ from **Table 7**, entry 11 in CDCl$_3$ at 298 K (PPh$_3$ addition time 10 min). **S77**
Fig. S92. ^{1}H NMR spectrum of PIP 500 equivalents generated by **Tm(CH$_2$SiMe$_3$)$_3$(THF)$_2$**-, 2 equivalents [Ph$_3$C][B(C$_6$F$_5$)$_4$], and 1 equivalent PPh$_3$ from **Table 7**, entry 12 in CDCl$_3$ at 298 K (PPh$_3$ addition time 30 min). **S77**
Fig. S93. ^{1}H NMR spectrum of PIP 500 equivalents generated by **Y(CH$_2$SiMe$_3$)$_3$(THF)$_2$** and 2 equivalents [Ph$_3$C][B(C$_6$F$_5$)$_4$] from **Table 7**, entry 13 in CDCl$_3$ at 298 K (IP addition time 10 min). **S78**
Fig. S94. ^{1}H NMR spectrum of PIP 500 equivalents generated by **Y(CH$_2$SiMe$_3$)$_3$(THF)$_2$** and 2 equivalents [Ph$_3$C][B(C$_6$F$_5$)$_4$] from **Table 7**, entry 14 in CDCl$_3$ at 298 K (IP addition time 20 min). **S78**
Fig. S95. ^{1}H NMR spectrum of PIP 500 equivalents generated by **Y(CH$_2$SiMe$_3$)$_3$(THF)$_2$** and 2 equivalents [Ph$_3$C][B(C$_6$F$_5$)$_4$] from **Table 7**, entry 15 in CDCl$_3$ at 298 K (IP addition time 40 min). **S79**
Fig. S96. ^{1}H NMR spectrum of PIP 500 equivalents generated by **Y(CH$_2$SiMe$_3$)$_3$(THF)$_2$**, 2 equivalents [Ph$_3$C][B(C$_6$F$_5$)$_4$], and 1 equivalent PPh$_3$ from **Table 8**, entry 1 (Step 1). **S79**
Fig. S97. ^{1}H NMR spectrum of PIP 500 equivalents generated by **Y(CH$_2$SiMe$_3$)$_3$(THF)$_2$**, 2 equivalents [Ph$_3$C][B(C$_6$F$_5$)$_4$], and 1 equivalent PPh$_3$ from **Table 8**, entry 2 (Step 2). **S80**
Fig. S98. ^{1}H NMR spectrum of PIP 500 equivalents generated by **Y(CH$_2$SiMe$_3$)$_3$(THF)$_2$**, 2 equivalents [Ph$_3$C][B(C$_6$F$_5$)$_4$], and 1 equivalent PPh$_3$ from **Table 8**, entry 3 (Step 3). **S80**

Fig. S99. ^{1}H NMR spectrum of PIP 500 equivalents generated by **$Y(CH_2SiMe_3)_3(THF)_2$** and 2 equivalents $[Ph_3C][B(C_6F_5)_4]$ from **Table 8**, entry 4 (Step 1). **S81**
Fig. S100. ^{1}H NMR spectrum of PIP 500 equivalents generated by **$Y(CH_2SiMe_3)_3(THF)_2$** and 2 equivalents $[Ph_3C][B(C_6F_5)_4]$ from **Table 8**, entry 5 (Step 2). **S81**
Fig. S101. ^{1}H NMR spectrum of PIP 500 equivalents generated by **$Y(CH_2SiMe_3)_3(THF)_2$** and 2 equivalents $[Ph_3C][B(C_6F_5)_4]$ from **Table 8**, entry 6 (Step 3). **S82**

^{13}C NMR Spectroscopy

Fig. S102. ^{13}C NMR spectrum of PIP 500 equivalents generated by **$Y(CH_2SiMe_3)_3(THF)_2$** 1 equivalent $[Ph_3C][B(C_6F_5)_4]$ from **Table 1**, entry 1 in $CDCl_3$ at 298 K (30 min). **S82**
Fig. S103. ^{13}C NMR spectrum of PIP 500 equivalents generated by **$Y(CH_2SiMe_3)_3(THF)_2$** and 2 equivalents $[Ph_3C][B(C_6F_5)_4]$ from **Table 1**, entry 2 in $CDCl_3$ at 298 K (30 min). **S83**
Fig. S104. ^{13}C NMR spectrum of PIP 500 equivalents generated by **$Y(CH_2SiMe_3)_3(THF)_2$**, 2 equivalents $[Ph_3C][B(C_6F_5)_4]$, and 1 equivalent Bipy from **Table 2**, entry 3 in $CDCl_3$ at 298 K (30 min). **S83**
Fig. S105. ^{13}C NMR spectrum of PIP 500 equivalents generated by **$Y(CH_2SiMe_3)_3(THF)_2$**, 2 equivalents $[Ph_3C][B(C_6F_5)_4]$, and 1 equivalent MeCN from **Table 2**, entry 5 in $CDCl_3$ at 298 K (30 min). **S84**
Fig. S106. ^{13}C NMR spectrum of PIP 500 equivalents generated by **$Y(CH_2SiMe_3)_3(THF)_2$**, 2 equivalents $[Ph_3C][B(C_6F_5)_4]$, and 1 equivalent P(*o*-tolyl)$_3$ from **Table 2**, entry 6 in $CDCl_3$ at 298 K (30 min). **S84**
Fig. S107. ^{13}C NMR spectrum of PIP 500 equivalents generated by **$Y(CH_2SiMe_3)_3(THF)_2$**, 2 equivalents $[Ph_3C][B(C_6F_5)_4]$, and 1 equivalent PCy_3 from **Table 2**, entry 7 in $CDCl_3$ at 298 K (30 min). **S85**
Fig. S108. ^{13}C NMR spectrum of PIP 500 equivalents generated by **$Y(CH_2SiMe_3)_3(THF)_2$**, 2 equivalents $[Ph_3C][B(C_6F_5)_4]$, and 1 PPh_3 from **Table 2**, entry 8 in $CDCl_3$ at 298 K (30 min). **S85**
Fig. S109. ^{13}C NMR spectrum of PIP 500 equivalents generated by **$Y(CH_2SiMe_3)_3(THF)_2$**, 2 equivalents $[Ph_3C][B(C_6F_5)_4]$, and 1 equivalent P(Ph-*p*-OMe)$_3$ from **Table S1**, entry 1 in $CDCl_3$ at 298 K (30 min). **S86**
Fig. S110. ^{13}C NMR spectrum of PIP 500 equivalents generated by **$Y(CH_2SiMe_3)_3(THF)_2$**, 2 equivalents $[Ph_3C][B(C_6F_5)_4]$, and 1 equivalent P(*p*-tolyl)$_3$ from **Table S1**, entry 2 in $CDCl_3$ at 298 K (30 min). **S86**
Fig. S111. ^{13}C NMR spectrum of PIP 500 equivalents generated by **$Y(CH_2SiMe_3)_3(THF)_2$**, 2 equivalents $[Ph_3C][B(C_6F_5)_4]$, and 1 equivalent P(Ph-*p*-F)$_3$ from **Table S1**, entry 4 in $CDCl_3$ at 298 K (10 min). **S87**
Fig. S112. ^{13}C NMR spectrum of PIP 500 equivalents generated by **$Y(CH_2SiMe_3)_3(THF)_2$** and 2 equivalents $[Ph_3C][B(C_6F_5)_4]$ from **Table S2**, entry 1 in $CDCl_3$ at 298 K (5 min). **S87**
Fig. S113. ^{13}C NMR spectrum of PIP 500 equivalents generated by **$Y(CH_2SiMe_3)_3(THF)_2$** and 2 equivalents $[Ph_3C][B(C_6F_5)_4]$ from **Table S2**, entry 2 in $CDCl_3$ at 298 K (12 min). **S88**
Fig. S114. ^{13}C NMR spectrum of PIP 500 equivalents generated by **$Y(CH_2SiMe_3)_3(THF)_2$** and 2 equivalents $[Ph_3C][B(C_6F_5)_4]$ from **Table S2**, entry 3 in $CDCl_3$ at 298 K (18 min). **S88**
Fig. S115. ^{13}C NMR spectrum of PIP 500 equivalents generated by **$Y(CH_2SiMe_3)_3(THF)_2$** and 2 equivalents $[Ph_3C][B(C_6F_5)_4]$ from **Table S2**, entry 4 in $CDCl_3$ at 298 K (24 min). **S89**
Fig. S116. ^{13}C NMR spectrum of PIP 500 equivalents generated by **$Y(CH_2SiMe_3)_3(THF)_2$** and 2 equivalents $[Ph_3C][B(C_6F_5)_4]$ from **Table S2**, entry 5 in $CDCl_3$ at 298 K (30 min). **S89**
Fig. S117. ^{13}C NMR spectrum of PIP 500 equivalents generated by **$Y(CH_2SiMe_3)_3(THF)_2$**, 2 equivalents $[Ph_3C][B(C_6F_5)_4]$, and 1 equivalent PPh_3 from **Table S3**, entry 1 in $CDCl_3$ at 298 K (10 min). **S90**

Fig. S118. ^{13}C NMR spectrum of PIP 500 equivalents generated by **$Y(CH_2SiMe_3)_3(THF)_2$**, 2 equivalents $[Ph_3C][B(C_6F_5)_4]$, and 1 equivalent PPh_3 from **Table S3**, entry 2 in $CDCl_3$ at 298 K (21 min). **S90**
Fig. S119. ^{13}C NMR spectrum of PIP 500 equivalents generated by **$Y(CH_2SiMe_3)_3(THF)_2$**, 2 equivalents $[Ph_3C][B(C_6F_5)_4]$, and 1 equivalent PPh_3 from **Table S3**, entry 3 in $CDCl_3$ at 298 K (31 min). **S91**
Fig. S120. ^{13}C NMR spectrum of PIP 500 equivalents generated by **$Y(CH_2SiMe_3)_3(THF)_2$**, 2 equivalents $[Ph_3C][B(C_6F_5)_4]$, and 1 equivalent PPh_3 from **Table S3**, entry 4 in $CDCl_3$ at 298 K (41 min). **S91**
Fig. S121. ^{13}C NMR spectrum of PIP 500 equivalents generated by **$Y(CH_2SiMe_3)_3(THF)_2$**, 2 equivalents $[Ph_3C][B(C_6F_5)_4]$, and 1 equivalent PPh_3 from **Table S3**, entry 5 in $CDCl_3$ at 298 K (51 min). **S92**
Fig. S122. ^{13}C NMR spectrum of PIP 500 equivalents generated by **$Sm(CH_2SiMe_3)_3(THF)_3$** and 1 equivalent $[Ph_3C][B(C_6F_5)_4]$ from **Table S4**, entry 1 in $CDCl_3$ at 298 K (7 h). **S92**
Fig. S123. ^{13}C NMR spectrum of PIP 500 equivalents generated by **$Gd(CH_2SiMe_3)_3(THF)_2$** and 1 equivalent $[Ph_3C][B(C_6F_5)_4]$ from **Table S4**, entry 2 in $CDCl_3$ at 298 K (7 h). **S93**
Fig. S124. ^{13}C NMR spectrum of PIP 500 equivalents generated by **$Gd(CH_2SiMe_3)_3(THF)_2$** and 2 equivalents $[Ph_3C][B(C_6F_5)_4]$ from **Table S4**, entry 3 in $CDCl_3$ at 298 K (7 h). **S93**
Fig. S125. ^{13}C NMR spectrum of PIP 500 equivalents generated by **$Tm(CH_2SiMe_3)_3(THF)_2$** and 1 equivalent $[Ph_3C][B(C_6F_5)_4]$ from **Table S4**, entry 4 in $CDCl_3$ at 298 K (7 h). **S94**
Fig. S126. ^{13}C NMR spectrum of PIP 500 equivalents generated by **$Tm(CH_2SiMe_3)_3(THF)_2$** and 2 equivalents $[Ph_3C][B(C_6F_5)_4]$ from **Table S4**, entry 5 in $CDCl_3$ at 298 K (7 h). **S94**
Fig. S127. ^{13}C NMR spectrum of PIP 500 equivalents generated by **$Sm(CH_2SiMe_3)_3(THF)_3$**, 1 equivalent $[Ph_3C][B(C_6F_5)_4]$, and 1 equivalent PPh_3 from **Table S4**, entry 6 in $CDCl_3$ at 298 K (7 h). **S95**
Fig. S128. ^{13}C NMR spectrum of PIP 500 equivalents generated by **$Gd(CH_2SiMe_3)_3(THF)_2$**, 1 equivalent $[Ph_3C][B(C_6F_5)_4]$, and 1 equivalent PPh_3 from **Table S4**, entry 7 in $CDCl_3$ at 298 K (7 h). **S95**
Fig. S129. ^{13}C NMR spectrum of PIP 500 equivalents generated by **$Gd(CH_2SiMe_3)_3(THF)_2$**, 2 equivalents $[Ph_3C][B(C_6F_5)_4]$, and 1 equivalent PPh_3 from **Table S4**, entry 8 in $CDCl_3$ at 298 K (7 h). **S96**
Fig. S130. ^{13}C NMR spectrum of PIP 500 equivalents generated by **$Tm(CH_2SiMe_3)_3(THF)_2$**, 1 equivalent $[Ph_3C][B(C_6F_5)_4]$, and 1 equivalent PPh_3 from **Table S4**, entry 9 in $CDCl_3$ at 298 K (7 h). **S96**
Fig. S131. ^{13}C NMR spectrum of PIP 500 equivalents generated by **$Tm(CH_2SiMe_3)_3(THF)_2$**, 2 equivalents $[Ph_3C][B(C_6F_5)_4]$, and 1 equivalent PPh_3 from **Table S4**, entry 10 in $CDCl_3$ at 298 K (7 h). **S97**
Fig. S132. ^{13}C NMR spectrum of PIP 500 equivalents generated by **$Y(CH_2SiMe_3)_3(THF)_2$** and 1 equivalent $[Ph_3C][B(C_6F_5)_4]$ from **Table 3**, entry 1 in $CDCl_3$ at 298 K (7 h). **S97**
Fig. S133. ^{13}C NMR spectrum of PIP 500 equivalents generated by **$Y(CH_2SiMe_3)_3(THF)_2$**, 1 equivalent $[Ph_3C][B(C_6F_5)_4]$, and 1 equivalent PPh_3 from **Table 3**, entry 2 in $CDCl_3$ at 298 K (7 h). **S98**
Fig. S134. ^{13}C NMR spectrum of PIP 500 equivalents generated by **$Y(CH_2SiMe_3)_3(THF)_2$**, 1.5 equivalents $[Ph_3C][B(C_6F_5)_4]$ from **Table 3**, entry 3 in $CDCl_3$ at 298 K (7 h). **S98**

Fig. S135. ^{13}C NMR spectrum of PIP 500 equivalents generated by **$Y(CH_2SiMe_3)_3(THF)_2$**, 1.5 equivalents $[Ph_3C][B(C_6F_5)_4]$, and 1 equivalent PPh_3 from **Table 3**, entry 4 in $CDCl_3$ at 298 K (7 h). **S99**
Fig. S136. ^{13}C NMR spectrum of PIP 500 equivalents generated by **$Y(CH_2SiMe_3)_3(THF)_2$** and 2 equivalents $[Ph_3C][B(C_6F_5)_4]$ from **Table 3**, entry 5 in $CDCl_3$ at 298 K (7 h). **S99**
Fig. S137. ^{13}C NMR spectrum of PIP 500 equivalents generated by **$Y(CH_2SiMe_3)_3(THF)_2$**, 2 equivalents $[Ph_3C][B(C_6F_5)_4]$, and 1 equivalent PPh_3 from **Table 3**, entry 6 in $CDCl_3$ at 298 K (7 h). **S100**
Fig. S138. ^{13}C NMR spectrum of PIP 500 equivalents generated by **$Y(CH_2SiMe_3)_3(THF)_2$** and 2.5 equivalents $[Ph_3C][B(C_6F_5)_4]$ from **Table 3**, entry 7 in $CDCl_3$ at 298 K (7 h). **S100**
Fig. S139. ^{13}C NMR spectrum of PIP 500 equivalents generated by **$Y(CH_2SiMe_3)_3(THF)_2$**, 2.5 equivalents $[Ph_3C][B(C_6F_5)_4]$, and 1 equivalent PPh_3 from **Table 3**, entry 8 in $CDCl_3$ at 298 K (7 h). **S101**
Fig. S140. ^{13}C NMR spectrum of PIP 500 equivalents generated by **$Y(CH_2SiMe_3)_3(THF)_2$** and 3 equivalents $[Ph_3C][B(C_6F_5)_4]$ from **Table 3**, entry 9 in $CDCl_3$ at 298 K (7 h). **S101**
Fig. S141. ^{13}C NMR spectrum of PIP 500 equivalents generated by **$Y(CH_2SiMe_3)_3(THF)_2$**, 3 equivalents $[Ph_3C][B(C_6F_5)_4]$, and 1 equivalent PPh_3 from **Table 3**, entry 10 in $CDCl_3$ at 298 K (7 h). **S102**
Fig. S142. ^{13}C NMR spectrum of PIP 500 equivalents generated by **$Y(CH_2SiMe_3)_3(THF)_2$**, 2 equivalents $[Ph_3C][B(C_6F_5)_4]$, and 5 equivalents $AlMe_3$ from **Table 4**, entry 1 in $CDCl_3$ at 298 K (30 min). **S102**
Fig. S143. ^{13}C NMR spectrum of PIP 500 equivalents generated by **$Y(CH_2SiMe_3)_3(THF)_2$**, 2 equivalents $[Ph_3C][B(C_6F_5)_4]$, and 10 equivalents $AlMe_3$ from **Table 4**, entry 2 in $CDCl_3$ at 298 K (30 min). **S103**
Fig. S144. ^{13}C NMR spectrum of PIP 500 equivalents generated by **$Y(CH_2SiMe_3)_3(THF)_2$**, 2 equivalents $[Ph_3C][B(C_6F_5)_4]$, and 15 equivalents $AlMe_3$ from **Table 4**, entry 3 in $CDCl_3$ at 298 K (30 min). **S103**
Fig. S145. ^{13}C NMR spectrum of PIP 500 equivalents generated by **$Y(CH_2SiMe_3)_3(THF)_2$**, 2 equivalents $[Ph_3C][B(C_6F_5)_4]$, and 5 equivalents $AlEt_3$ from **Table 4**, entry 4 in $CDCl_3$ at 298 K (30 min). **S104**
Fig. S146. ^{13}C NMR spectrum of PIP 500 equivalents generated by **$Y(CH_2SiMe_3)_3(THF)_2$**, 2 equivalents $[Ph_3C][B(C_6F_5)_4]$, and 10 equivalents $AlEt_3$ from **Table 4**, entry 5 in $CDCl_3$ at 298 K (30 min). **S104**
Fig. S147. ^{13}C NMR spectrum of PIP 500 equivalents generated by **$Y(CH_2SiMe_3)_3(THF)_2$**, 2 equivalents $[Ph_3C][B(C_6F_5)_4]$, and 15 equivalents $AlEt_3$ from **Table 4**, entry 6 in $CDCl_3$ at 298 K (30 min). **S105**
Fig. S148. ^{13}C NMR spectrum of PIP 500 equivalents generated by **$Y(CH_2SiMe_3)_3(THF)_2$**, 2 equivalents $[Ph_3C][B(C_6F_5)_4]$, and 5 equivalents $Al^{i}Bu_3$ from **Table 4**, entry 7 in $CDCl_3$ at 298 K (30 min). **S105**
Fig. S149. ^{13}C NMR spectrum of PIP 500 equivalents generated by **$Y(CH_2SiMe_3)_3(THF)_2$**, 2 equivalents $[Ph_3C][B(C_6F_5)_4]$, and 10 equivalents $Al^{i}Bu_3$ from **Table 4**, entry 8 in $CDCl_3$ at 298 K (30 min). **S106**
Fig. S150. ^{13}C NMR spectrum of PIP 500 equivalents generated by **$Y(CH_2SiMe_3)_3(THF)_2$**, 2 equivalents $[Ph_3C][B(C_6F_5)_4]$, and 15 equivalents $Al^{i}Bu_3$ from **Table 4**, entry 9 in $CDCl_3$ at 298 K (30 min). **S106**
Fig. S151. ^{13}C NMR spectrum of PIP 500 equivalents generated by **$Y(CH_2SiMe_3)_3(THF)_2$**, 2 equivalents $[Ph_3C][B(C_6F_5)_4]$, 1 equivalent PPh_3, and 5 equivalents $Al^{i}Bu_3$ from **Table 4**, entry 10 in $CDCl_3$ at 298 K (30 min). **S107**
Fig. S152. ^{13}C NMR spectrum of PIP 500 equivalents generated by **$Y(CH_2SiMe_3)_3(THF)_2$**, 2 equivalents $[Ph_3C][B(C_6F_5)_4]$, 1 equivalent PPh_3, and 10 equivalents $Al^{i}Bu_3$ from **Table 4**, entry 11 in $CDCl_3$ at 298 K (30 min). **S107**

Fig. S153. ^{13}C NMR spectrum of PIP 500 equivalents generated by **$Y(CH_2SiMe_3)_3(THF)_2$**, 2 equivalents $[Ph_3C][B(C_6F_5)_4]$, 1 equivalent PPh_3, and 15 equivalents Al^iBu_3 from **Table 4**, entry 12 in $CDCl_3$ at 298 K (30 min). **S108**
Fig. S154. ^{13}C NMR spectrum of PIP 500 equivalents generated by **$Sm(CH_2SiMe_3)_3(THF)_3$** and 1 equivalent $[Ph_3C][B(C_6F_5)_4]$ from **Table 5**, entry 1 in $CDCl_3$ at 298 K (30 min). **S108**
Fig. S155. ^{13}C NMR spectrum of PIP 500 equivalents generated by **$Sm(CH_2SiMe_3)_3(THF)_3$** and 2 equivalents $[Ph_3C][B(C_6F_5)_4]$ from **Table 5**, entry 2 in $CDCl_3$ at 298 K (30 min). **S109**
Fig. S156. ^{13}C NMR spectrum of PIP 500 equivalents generated by **$Gd(CH_2SiMe_3)_3(THF)_2$** and 1 equivalent $[Ph_3C][B(C_6F_5)_4]$ from **Table 5**, entry 3 in $CDCl_3$ at 298 K (30 min). **S109**
Fig. S157. ^{13}C NMR spectrum of PIP 500 equivalents generated by **$Gd(CH_2SiMe_3)_3(THF)_2$** and 2 equivalents $[Ph_3C][B(C_6F_5)_4]$ from **Table 5**, entry 4 in $CDCl_3$ at 298 K (30 min). **S110**
Fig. S158. ^{13}C NMR spectrum of PIP 500 equivalents generated by **$Y(CH_2SiMe_3)_3(THF)_2$** and 1 equivalent $[Ph_3C][B(C_6F_5)_4]$ from **Table 5**, entry 5 in $CDCl_3$ at 298 K (30 min). **S110**
Fig. S159. ^{13}C NMR spectrum of PIP 500 equivalents generated by **$Y(CH_2SiMe_3)_3(THF)_2$** and 2 equivalents $[Ph_3C][B(C_6F_5)_4]$ from **Table 5**, entry 6 in $CDCl_3$ at 298 K (30 min). **S111**
Fig. S160. ^{13}C NMR spectrum of PIP 500 equivalents generated by **$Tm(CH_2SiMe_3)_3(THF)_2$** and 1 equivalent $[Ph_3C][B(C_6F_5)_4]$ from **Table 5**, entry 7 in $CDCl_3$ at 298 K (30 min). **S111**
Fig. S161. ^{13}C NMR spectrum of PIP 500 equivalents generated by **$Tm(CH_2SiMe_3)_3(THF)_2$** and 2 equivalents $[Ph_3C][B(C_6F_5)_4]$ from **Table 5**, entry 8 in $CDCl_3$ at 298 K (30 min). **S112**
Fig. S162. ^{13}C NMR spectrum of PIP 500 equivalents generated by **$Sm(CH_2SiMe_3)_3(THF)_3$**, 1 equivalent $[Ph_3C][B(C_6F_5)_4]$, and 1 equivalent PPh_3 from **Table 5**, entry 9 in $CDCl_3$ at 298 K (30 min). **S112**
Fig. S163. ^{13}C NMR spectrum of PIP 500 equivalents generated by **$Sm(CH_2SiMe_3)_3(THF)_3$**, 2 equivalents $[Ph_3C][B(C_6F_5)_4]$, and 1 equivalent PPh_3 from **Table 5**, entry 10 in $CDCl_3$ at 298 K (30 min). **S113**
Fig. S164. ^{13}C NMR spectrum of PIP 500 equivalents generated by **$Gd(CH_2SiMe_3)_3(THF)_2$**, 1 equivalent $[Ph_3C][B(C_6F_5)_4]$, and 1 equivalent PPh_3 from **Table 5**, entry 11 in $CDCl_3$ at 298 K (30 min). **S113**
Fig. S165. ^{13}C NMR spectrum of PIP 500 equivalents generated by **$Gd(CH_2SiMe_3)_3(THF)_2$**, 2 equivalents $[Ph_3C][B(C_6F_5)_4]$, and 1 equivalent PPh_3 from **Table 5**, entry 12 in $CDCl_3$ at 298 K (30 min). **S114**
Fig. S166. ^{13}C NMR spectrum of PIP 500 equivalents generated by **$Y(CH_2SiMe_3)_3(THF)_2$**, 1 equivalent $[Ph_3C][B(C_6F_5)_4]$, and 1 equivalent PPh_3 from **Table 5**, entry 13 in $CDCl_3$ at 298 K (30 min). **S114**
Fig. S167. ^{13}C NMR spectrum of PIP 500 equivalents generated by **$Y(CH_2SiMe_3)_3(THF)_2$**, 2 equivalents $[Ph_3C][B(C_6F_5)_4]$, and 1 equivalent PPh_3 from **Table 5**, entry 14 in $CDCl_3$ at 298 K (30 min). **S115**
Fig. S168. ^{13}C NMR spectrum of PIP 500 equivalents generated by **$Tm(CH_2SiMe_3)_3(THF)_2$**, 1 equivalent $[Ph_3C][B(C_6F_5)_4]$, and 1 equivalent PPh_3 from **Table 5**, entry 15 in $CDCl_3$ at 298 K (30 min). **S115**
Fig. S169. ^{13}C NMR spectrum of PIP 500 equivalents generated by **$Tm(CH_2SiMe_3)_3(THF)_2$**, 2 equivalents $[Ph_3C][B(C_6F_5)_4]$, and 1 equivalent PPh_3 from **Table 5**, entry 16 in $CDCl_3$ at 298 K (30 min). **S116**

Fig. S170. ^{13}C NMR spectrum of PIP 500 equivalents generated by **$Sm(CH_2SiMe_3)_3(THF)_3$**, 1 equivalent PPh_3, and 2 equivalents $[Ph_3C][B(C_6F_5)_4]$ and from **Table 6**, entry 1 in $CDCl_3$ at 298 K ($[Ph_3C][B(C_6F_5)_4]$ addition time 0 min). **S116**
Fig. S171. ^{13}C NMR spectrum of PIP 500 equivalents generated by **$Sm(CH_2SiMe_3)_3(THF)_3$**, 1 equivalent PPh_3, and 2 equivalents $[Ph_3C][B(C_6F_5)_4]$ and from **Table 6**, entry 2 in $CDCl_3$ at 298 K ($[Ph_3C][B(C_6F_5)_4]$ addition time 10 min). **S117**
Fig. S172. ^{13}C NMR spectrum of PIP 500 equivalents generated by **$Sm(CH_2SiMe_3)_3(THF)_3$**, 1 equivalent PPh_3, and 2 equivalents $[Ph_3C][B(C_6F_5)_4]$ from **Table 6**, entry 3 in $CDCl_3$ at 298 K ($[Ph_3C][B(C_6F_5)_4]$ addition time 30 min). **S117**
Fig. S173. ^{13}C NMR spectrum of PIP 500 equivalents generated by **$Gd(CH_2SiMe_3)_3(THF)_2$**, 1 equivalent PPh_3, and 2 equivalents $[Ph_3C][B(C_6F_5)_4]$ from **Table 6**, entry 4 in $CDCl_3$ at 298 K ($[Ph_3C][B(C_6F_5)_4]$ addition time 0 min). **S118**
Fig. S174. ^{13}C NMR spectrum of PIP 500 equivalents generated by **$Gd(CH_2SiMe_3)_3(THF)_2$**, 1 equivalent PPh_3, and 2 equivalents $[Ph_3C][B(C_6F_5)_4]$ from **Table 6**, entry 5 in $CDCl_3$ at 298 K ($[Ph_3C][B(C_6F_5)_4]$ addition time 10 min). **S118**
Fig. S175. ^{13}C NMR spectrum of PIP 500 equivalents generated by **$Gd(CH_2SiMe_3)_3(THF)_2$**, 1 equivalent PPh_3, and 2 equivalents $[Ph_3C][B(C_6F_5)_4]$ from **Table 6**, entry 6 in $CDCl_3$ at 298 K ($[Ph_3C][B(C_6F_5)_4]$ addition time 30 min). **S119**
Fig. S176. ^{13}C NMR spectrum of PIP 500 equivalents generated by **$Y(CH_2SiMe_3)_3(THF)_2$**, 1 equivalent PPh_3, and 2 equivalents $[Ph_3C][B(C_6F_5)_4]$ from **Table 6**, entry 7 in $CDCl_3$ at 298 K ($[Ph_3C][B(C_6F_5)_4]$ addition time 0 min). **S119**
Fig. S177. ^{13}C NMR spectrum of PIP 500 equivalents generated by **$Y(CH_2SiMe_3)_3(THF)_2$**, 1 equivalent PPh_3, and 2 equivalents $[Ph_3C][B(C_6F_5)_4]$ from **Table 6**, entry 8 in $CDCl_3$ at 298 K ($[Ph_3C][B(C_6F_5)_4]$ addition time 10 min). **S120**
Fig. S178. ^{13}C NMR spectrum of PIP 500 equivalents generated by **$Y(CH_2SiMe_3)_3(THF)_2$**, 1 equivalent PPh_3, and 2 equivalents $[Ph_3C][B(C_6F_5)_4]$ from **Table 6**, entry 9 in $CDCl_3$ at 298 K ($[Ph_3C][B(C_6F_5)_4]$ addition time 30 min). **S120**
Fig. S179. ^{13}C NMR spectrum of PIP 500 equivalents generated by **$Tm(CH_2SiMe_3)_3(THF)_2$**, 1 equivalent PPh_3, and 2 equivalents $[Ph_3C][B(C_6F_5)_4]$ from **Table 6**, entry 10 in $CDCl_3$ at 298 K ($[Ph_3C][B(C_6F_5)_4]$ addition time 0 min). **S121**
Fig. S180. ^{13}C NMR spectrum of PIP 500 equivalents generated by **$Tm(CH_2SiMe_3)_3(THF)_2$**, 1 equivalent PPh_3, and 2 equivalents $[Ph_3C][B(C_6F_5)_4]$ from **Table 6**, entry 11 in $CDCl_3$ at 298 K ($[Ph_3C][B(C_6F_5)_4]$ addition time 10 min). **S121**
Fig. S181. ^{13}C NMR spectrum of PIP 500 equivalents generated by **$Tm(CH_2SiMe_3)_3(THF)_2$**, 1 equivalent PPh_3, and 2 equivalents $[Ph_3C][B(C_6F_5)_4]$ from **Table 6**, entry 12 in $CDCl_3$ at 298 K ($[Ph_3C][B(C_6F_5)_4]$ addition time 30 min). **S122**
Fig. S182. ^{13}C NMR spectrum of PIP 500 equivalents generated by **$Sm(CH_2SiMe_3)_3(THF)_3$**, 2 equivalents $[Ph_3C][B(C_6F_5)_4]$, and 1 equivalent PPh_3 from **Table 7**, entry 1 in $CDCl_3$ at 298 K (PPh_3 addition time 0 min). **S122**
Fig. S183. ^{13}C NMR spectrum of PIP 500 equivalents generated by **$Sm(CH_2SiMe_3)_3(THF)_3$**, 2 equivalents $[Ph_3C][B(C_6F_5)_4]$, and 1 equivalent PPh_3 from **Table 7**, entry 2 in $CDCl_3$ at 298 K (PPh_3 addition time 10 min). **S123**
Fig. S184. ^{13}C NMR spectrum of PIP 500 equivalents generated by **$Sm(CH_2SiMe_3)_3(THF)_3$**, 2 equivalents $[Ph_3C][B(C_6F_5)_4]$, and 1 equivalent PPh_3 from **Table 7**, entry 3 in $CDCl_3$ at 298 K (PPh_3 addition time 30 min). **S123**
Fig. S185. ^{13}C NMR spectrum of PIP 500 equivalents generated by **$Gd(CH_2SiMe_3)_3(THF)_2$**, 2 equivalents $[Ph_3C][B(C_6F_5)_4]$, and 1 equivalent PPh_3 from **Table 7**, entry 4 in $CDCl_3$ at 298 K (PPh_3 addition time 0 min). **S124**
Fig. S186. ^{13}C NMR spectrum of PIP 500 equivalents generated by **$Gd(CH_2SiMe_3)_3(THF)_2$**, 2 equivalents $[Ph_3C][B(C_6F_5)_4]$, and 1 equivalent PPh_3 from **Table 7**, entry 5 in $CDCl_3$ at 298 K (PPh_3 addition time 10 min). **S124**

Fig. S187. ^{13}C NMR spectrum of PIP 500 equivalents generated by **$Gd(CH_2SiMe_3)_3(THF)_2$**, 2 equivalents $[Ph_3C][B(C_6F_5)_4]$, and 1 equivalent PPh_3 from **Table 7**, entry 6 in $CDCl_3$ at 298 K (PPh_3 addition time 30 min). **S125**
Fig. S188. ^{13}C NMR spectrum of PIP 500 equivalents generated by **$Y(CH_2SiMe_3)_3(THF)_2$**, 2 equivalents $[Ph_3C][B(C_6F_5)_4]$, and 1 equivalent PPh_3 from **Table 7**, entry 7 in $CDCl_3$ at 298 K (PPh_3 addition time 0 min). **S125**
Fig. S189. ^{13}C NMR spectrum of PIP 500 equivalents generated by **$Y(CH_2SiMe_3)_3(THF)_2$**, 2 equivalents $[Ph_3C][B(C_6F_5)_4]$, and 1 equivalent PPh_3 from **Table 7**, entry 8 in $CDCl_3$ at 298 K (PPh_3 addition time 10 min). **S126**
Fig. S190. ^{13}C NMR spectrum of PIP 500 equivalents generated by **$Y(CH_2SiMe_3)_3(THF)_2$**, 2 equivalents $[Ph_3C][B(C_6F_5)_4]$, and 1 equivalent PPh_3 from **Table 7**, entry 9 in $CDCl_3$ at 298 K (PPh_3 addition time 30 min). **S126**
Fig. S191. ^{13}C NMR spectrum of PIP 500 equivalents generated by **$Tm(CH_2SiMe_3)_3(THF)_2$**, 2 equivalents $[Ph_3C][B(C_6F_5)_4]$, and 1 equivalent PPh_3 from **Table 7**, entry 10 in $CDCl_3$ at 298 K (PPh_3 addition time 0 min). **S127**
Fig. S192. ^{13}C NMR spectrum of PIP 500 equivalents generated by **$Tm(CH_2SiMe_3)_3(THF)_2$**, 2 equivalents $[Ph_3C][B(C_6F_5)_4]$, and 1 equivalent PPh_3 from **Table 7**, entry 11 in $CDCl_3$ at 298 K (PPh_3 addition time 10 min). **S127**
Fig. S193. ^{13}C NMR spectrum of PIP 500 equivalents generated by **$Tm(CH_2SiMe_3)_3(THF)_2$**, 2 equivalents $[Ph_3C][B(C_6F_5)_4]$, and 1 equivalent PPh_3 from **Table 7**, entry 12 in $CDCl_3$ at 298 K (PPh_3 addition time 30 min). **S128**
Fig. S194. ^{13}C NMR spectrum of PIP 500 equivalents generated by **$Y(CH_2SiMe_3)_3(THF)_2$** and 2 equivalents $[Ph_3C][B(C_6F_5)_4]$ from **Table 7**, entry 13 in $CDCl_3$ at 298 K (IP addition time 10 min). **S128**
Fig. S195. ^{13}C NMR spectrum of PIP 500 equivalents generated by **$Y(CH_2SiMe_3)_3(THF)_2$** and 2 equivalents $[Ph_3C][B(C_6F_5)_4]$ from **Table 7**, entry 14 in $CDCl_3$ at 298 K (IP addition time 20 min). **S129**
Fig. S196. ^{13}C NMR spectrum of PIP 500 equivalents generated by **$Y(CH_2SiMe_3)_3(THF)_2$** and 2 equivalents $[Ph_3C][B(C_6F_5)_4]$ from **Table 7**, entry 15 in $CDCl_3$ at 298 K (IP addition time 40 min). **S129**
Fig. S197. ^{13}C NMR spectrum of PIP 500 equivalents generated by **$Y(CH_2SiMe_3)_3(THF)_2$**, and 2 equivalents $[Ph_3C][B(C_6F_5)_4]$, and 1 equivalent PPh_3 from **Table 8**, entry 1 (Step 1). **S130**
Fig. S198. ^{13}C NMR spectrum of PIP 500 equivalents generated by **$Y(CH_2SiMe_3)_3(THF)_2$**, and 2 equivalents $[Ph_3C][B(C_6F_5)_4]$, and 1 equivalent PPh_3 from **Table 8**, entry 2 (Step 2). **S130**
Fig. S199. ^{13}C NMR spectrum of PIP 500 equivalents generated by **$Y(CH_2SiMe_3)_3(THF)_2$**, and 2 equivalents $[Ph_3C][B(C_6F_5)_4]$, and 1 equivalent PPh_3 from **Table 8**, entry 3 (Step 3). **S131**
Fig. S200. ^{13}C NMR spectrum of PIP 500 equivalents generated by **$Y(CH_2SiMe_3)_3(THF)_2$** and 2 equivalents $[Ph_3C][B(C_6F_5)_4]$ from **Table 8**, entry 4 (Step 1). **S131**
Fig. S201. ^{13}C NMR spectrum of PIP 500 equivalents generated by **$Y(CH_2SiMe_3)_3(THF)_2$** and 2 equivalents $[Ph_3C][B(C_6F_5)_4]$ from **Table 8**, entry 5 (Step 2). **S132**
Fig. S202. ^{13}C NMR spectrum of PIP 500 equivalents generated by **$Y(CH_2SiMe_3)_3(THF)_2$** and 2 equivalents $[Ph_3C][B(C_6F_5)_4]$ from **Table 8**, entry 6 (Step 3). **S132**
4.0 Fourier-Transform Infrared (FT-IR) Spectroscopy Characterization of Isolated Polymers **S133**
Fig. S203. FT-IR spectrum of PIP 500 equivalents generated by **$Y(CH_2SiMe_3)_3(THF)_2$** and 1 equivalent $[Ph_3C][B(C_6F_5)_4]$ from **Table 1**, entry 1 (30 min). **S133**
Fig. S204. FT-IR spectrum of PIP 500 equivalents generated by **$Y(CH_2SiMe_3)_3(THF)_2$** and 2 equivalents $[Ph_3C][B(C_6F_5)_4]$ from **Table 1**, entry 2 (30 min). **S133**
Fig. S205. FT-IR spectrum of PIP 500 equivalents generated by **$Y(CH_2SiMe_3)_3(THF)_2$**, 2 equivalents $[Ph_3C][B(C_6F_5)_4]$, and 1 equivalent Bipy from **Table 2**, entry 3 (30 min). **S134**

Fig. S206. FT-IR spectrum of PIP 500 equivalents generated by **$Y(CH_2SiMe_3)_3(THF)_2$**, 2 equivalents $[Ph_3C][B(C_6F_5)_4]$, and 1 equivalent MeCN from **Table 2**, entry 5 (30 min). **S134**
Fig. S207. FT-IR spectrum of PIP 500 equivalents generated by **$Y(CH_2SiMe_3)_3(THF)_2$**, 2 equivalents $[Ph_3C][B(C_6F_5)_4]$, and 1 equivalent P(*o*-tolyl)$_3$ from **Table 2**, entry 6 (30 min). **S135**
Fig. S208. FT-IR spectrum of PIP 500 equivalents generated by **$Y(CH_2SiMe_3)_3(THF)_2$**, 2 equivalents $[Ph_3C][B(C_6F_5)_4]$, and 1 equivalent PCy_3 from **Table 2**, entry 7 (30 min). **S135**
Fig. S209. FT-IR spectrum of PIP 500 equivalents generated by **$Y(CH_2SiMe_3)_3(THF)_2$**, 2 equivalents $[Ph_3C][B(C_6F_5)_4]$, and 1 equivalent PPh_3 from **Table 2**, entry 8 (30 min). **S136**
Fig. S210. FT-IR spectrum of PIP 500 equivalents generated by **$Y(CH_2SiMe_3)_3(THF)_2$**, 2 equivalents $[Ph_3C][B(C_6F_5)_4]$, and 1 equivalent P(Ph-*p*-OMe)$_3$ from **Table S1**, entry 1 (30 min). **S136**
Fig. S211. FT-IR spectrum of PIP 500 equivalents generated by **$Y(CH_2SiMe_3)_3(THF)_2$**, 2 equivalents $[Ph_3C][B(C_6F_5)_4]$, and 1 equivalent P(*p*-tolyl)$_3$ from **Table S1**, entry 2 (30 min). **S137**
Fig. S212. FT-IR spectrum of PIP 500 equivalents generated by **$Y(CH_2SiMe_3)_3(THF)_2$**, 2 equivalents $[Ph_3C][B(C_6F_5)_4]$, and 1 equivalent P(Ph-*p*-F)$_3$ from **Table S1**, entry 4 (30 min). **S137**
Fig. S213. FT-IR spectrum of PIP 500 equivalents generated by **$Y(CH_2SiMe_3)_3(THF)_2$** and 2 equivalents $[Ph_3C][B(C_6F_5)_4]$ from **Table S2**, entry 1 (5 min). **S138**
Fig. S214. FT-IR spectrum of PIP 500 equivalents generated by **$Y(CH_2SiMe_3)_3(THF)_2$** and 2 equivalents $[Ph_3C][B(C_6F_5)_4]$ from **Table S2**, entry 2 (12 min). **S138**
Fig. S215. FT-IR spectrum of PIP 500 equivalents generated by **$Y(CH_2SiMe_3)_3(THF)_2$** and 2 equivalents $[Ph_3C][B(C_6F_5)_4]$ from **Table S2**, entry 3 (18 min). **S139**
Fig. S216. FT-IR spectrum of PIP 500 equivalents generated by **$Y(CH_2SiMe_3)_3(THF)_2$** and 2 equivalents $[Ph_3C][B(C_6F_5)_4]$ from **Table S2**, entry 4 (24 min). **S139**
Fig. S217. FT-IR spectrum of PIP 500 equivalents generated by **$Y(CH_2SiMe_3)_3(THF)_2$** and 2 equivalents $[Ph_3C][B(C_6F_5)_4]$ from **Table S2**, entry 5 (30 min). **S140**
Fig. S218. FT-IR spectrum of PIP 500 equivalents generated by **$Y(CH_2SiMe_3)_3(THF)_2$**, 2 equivalents $[Ph_3C][B(C_6F_5)_4]$, and 1 equivalent PPh_3 from **Table S3**, entry 1 (10 min). **S140**
Fig. S219. FT-IR spectrum of PIP 500 equivalents generated by **$Y(CH_2SiMe_3)_3(THF)_2$**, 2 equivalents $[Ph_3C][B(C_6F_5)_4]$, and 1 equivalent PPh_3 from **Table S3**, entry 2 (21 min). **S141**
Fig. S220. FT-IR spectrum of PIP 500 equivalents generated by **$Y(CH_2SiMe_3)_3(THF)_2$**, 2 equivalents $[Ph_3C][B(C_6F_5)_4]$, and 1 equivalent PPh_3 from **Table S3**, entry 3 (31 min). **S141**
Fig. S221. FT-IR spectrum of PIP 500 equivalents generated by **$Y(CH_2SiMe_3)_3(THF)_2$**, 2 equivalents $[Ph_3C][B(C_6F_5)_4]$, and 1 equivalent PPh_3 from **Table S3**, entry 4 (41 min). **S142**
Fig. S222. FT-IR spectrum of PIP 500 equivalents generated by **$Y(CH_2SiMe_3)_3(THF)_2$**, 2 equivalents $[Ph_3C][B(C_6F_5)_4]$, and 1 equivalent PPh_3 from **Table S3**, entry 5 (51 min). **S142**
Fig. S223. FT-IR spectrum of PIP 500 equivalents generated by **$Sm(CH_2SiMe_3)_3(THF)_3$** and 1 equivalent $[Ph_3C][B(C_6F_5)_4]$ from **Table S4**, entry 1 (7 h). **S143**
Fig. S224. FT-IR spectrum of PIP 500 equivalents generated by **$Gd(CH_2SiMe_3)_3(THF)_2$** and 1 equivalent $[Ph_3C][B(C_6F_5)_4]$ from **Table S4**, entry 2 (7 h). **S143**
Fig. S225. FT-IR spectrum of PIP 500 equivalents generated by **$Gd(CH_2SiMe_3)_3(THF)_2$** and 2 equivalents $[Ph_3C][B(C_6F_5)_4]$ from **Table S4**, entry 3 (7 h). **S144**
Fig. S226. FT-IR spectrum of PIP 500 equivalents generated by **$Tm(CH_2SiMe_3)_3(THF)_2$** and 1 equivalent $[Ph_3C][B(C_6F_5)_4]$ from **Table S4**, entry 4 (7 h). **S144**
Fig. S227. FT-IR spectrum of PIP 500 equivalents generated by **$Tm(CH_2SiMe_3)_3(THF)_2$** and 2 equivalents $[Ph_3C][B(C_6F_5)_4]$ from **Table S4**, entry 5 (7 h). **S145**
Fig. S228. FT-IR spectrum of PIP 500 equivalents generated by **$Sm(CH_2SiMe_3)_3(THF)_3$**, 1 equivalent $[Ph_3C][B(C_6F_5)_4]$, and 1 equivalent PPh_3 from **Table S4**, entry 6 (7 h). **S145**
Fig. S229. FT-IR spectrum of PIP 500 equivalents generated by **$Gd(CH_2SiMe_3)_3(THF)_2$**, 1 equivalent $[Ph_3C][B(C_6F_5)_4]$, and 1 equivalent PPh_3 from **Table S4**, entry 7 (7 h). **S146**

Fig. S230. FT-IR spectrum of PIP 500 equivalents generated by **$Gd(CH_2SiMe_3)_3(THF)_2$**, 2 equivalents $[Ph_3C][B(C_6F_5)_4]$, and 1 equivalent PPh_3 from **Table S4**, entry 8 (7 h). **S146**
Fig. S231. FT-IR spectrum of PIP 500 equivalents generated by **$Tm(CH_2SiMe_3)_3(THF)_2$**, 1 equivalent $[Ph_3C][B(C_6F_5)_4]$, and 1 equivalent PPh_3 from **Table S4**, entry 9 (7 h). **S147**
Fig. S232. FT-IR spectrum of PIP 500 equivalents generated by **$Tm(CH_2SiMe_3)_3(THF)_2$**, 2 equivalents $[Ph_3C][B(C_6F_5)_4]$, and 1 equivalent PPh_3 from **Table S4**, entry 10 (7 h). **S147**
Fig. S233. FT-IR spectrum of PIP 500 equivalents generated by **$Y(CH_2SiMe_3)_3(THF)_2$** and 1 equivalent $[Ph_3C][B(C_6F_5)_4]$ from **Table 3**, entry 1 (7 h). **S148**
Fig. S234. FT-IR spectrum of PIP 500 equivalents generated by **$Y(CH_2SiMe_3)_3(THF)_2$**, 1 equivalent $[Ph_3C][B(C_6F_5)_4]$, and 1 equivalent PPh_3 from **Table 3**, entry 2 (7 h). **S148**
Fig. S235. FT-IR spectrum of PIP 500 equivalents generated by **$Y(CH_2SiMe_3)_3(THF)_2$** and 1.5 equivalents $[Ph_3C][B(C_6F_5)_4]$ from **Table 3**, entry 3 (7 h). **S149**
Fig. S236. FT-IR spectrum of PIP 500 equivalents generated by **$Y(CH_2SiMe_3)_3(THF)_2$**, 1.5 equivalents $[Ph_3C][B(C_6F_5)_4]$, and 1 equivalent PPh_3 from **Table 3**, entry 4 (7 h). **S149**
Fig. S237. FT-IR spectrum of PIP 500 equivalents generated by **$Y(CH_2SiMe_3)_3(THF)_2$** and 2 equivalents $[Ph_3C][B(C_6F_5)_4]$ from **Table 3**, entry 5 (7 h). **S150**
Fig. S238. FT-IR spectrum of PIP 500 equivalents generated by **$Y(CH_2SiMe_3)_3(THF)_2$**, 2 equivalents $[Ph_3C][B(C_6F_5)_4]$, and 1 equivalent PPh_3 from **Table 3**, entry 6 (7 h). **S150**
Fig. S239. FT-IR spectrum of PIP 500 equivalents generated by **$Y(CH_2SiMe_3)_3(THF)_2$** and 2.5 equivalents $[Ph_3C][B(C_6F_5)_4]$ from **Table 3**, entry 7 (7 h). **S151**
Fig. S240. FT-IR spectrum of PIP 500 equivalents generated by **$Y(CH_2SiMe_3)_3(THF)_2$**, 2.5 equivalents $[Ph_3C][B(C_6F_5)_4]$, and 1 equivalent PPh_3 from **Table 3**, entry 8 (7 h). **S151**
Fig. S241. FT-IR spectrum of PIP 500 equivalents generated by **$Y(CH_2SiMe_3)_3(THF)_2$** and 3 equivalents $[Ph_3C][B(C_6F_5)_4]$ from **Table 3**, entry 9 (7 h). **S152**
Fig. S242. FT-IR spectrum of PIP 500 equivalents generated by **$Y(CH_2SiMe_3)_3(THF)_2$**, 3 equivalents $[Ph_3C][B(C_6F_5)_4]$, and 1 equivalent PPh_3 from **Table 3**, entry 10 (7 h). **S152**
Fig. S243. FT-IR spectrum of PIP 500 equivalents generated by **$Y(CH_2SiMe_3)_3(THF)_2$**, 2 equivalents $[Ph_3C][B(C_6F_5)_4]$, and 5 equivalents $AlMe_3$ from **Table 4**, entry 1 (30 min). **S153**
Fig. S244. FT-IR spectrum of PIP 500 equivalents generated by **$Y(CH_2SiMe_3)_3(THF)_2$**, 2 equivalents $[Ph_3C][B(C_6F_5)_4]$, and 10 equivalents $AlMe_3$ from **Table 4**, entry 2 (30 min). **S153**
Fig. S245. FT-IR spectrum of PIP 500 equivalents generated by **$Y(CH_2SiMe_3)_3(THF)_2$**, 2 equivalents $[Ph_3C][B(C_6F_5)_4]$, and 15 equivalents $AlMe_3$ from **Table 4**, entry 3 (30 min). **S154**
Fig. S246. FT-IR spectrum of PIP 500 equivalents generated by **$Y(CH_2SiMe_3)_3(THF)_2$**, 2 equivalents $[Ph_3C][B(C_6F_5)_4]$, and 5 equivalents $AlEt_3$ from **Table 4**, entry 4 (30 min). **S154**
Fig. S247. FT-IR spectrum of PIP 500 equivalents generated by **$Y(CH_2SiMe_3)_3(THF)_2$**, 2 equivalents $[Ph_3C][B(C_6F_5)_4]$, and 10 equivalents $AlEt_3$ from **Table 4**, entry 5 (30 min). **S155**
Fig. S248. FT-IR spectrum of PIP 500 equivalents generated by **$Y(CH_2SiMe_3)_3(THF)_2$**, 2 equivalents $[Ph_3C][B(C_6F_5)_4]$, and 15 equivalents $AlEt_3$ from **Table 4**, entry 6 (30 min). **S155**
Fig. S249. FT-IR spectrum of PIP 500 equivalents generated by **$Y(CH_2SiMe_3)_3(THF)_2$**, 2 equivalents $[Ph_3C][B(C_6F_5)_4]$, and 5 equivalents Al^iBu_3 from **Table 4**, entry 7 (30 min). **S156**
Fig. S250. FT-IR spectrum of PIP 500 equivalents generated by **$Y(CH_2SiMe_3)_3(THF)_2$**, 2 equivalents $[Ph_3C][B(C_6F_5)_4]$, and 10 equivalents Al^iBu_3 from **Table 4**, entry 8 (30 min). **S156**
Fig. S251. FT-IR spectrum of PIP 500 equivalents generated by **$Y(CH_2SiMe_3)_3(THF)_2$**, 2 equivalents $[Ph_3C][B(C_6F_5)_4]$, and 15 equivalents Al^iBu_3 from **Table 4**, entry 9 (30 min). **S157**
Fig. S252. FT-IR spectrum of PIP 500 equivalents generated by **$Y(CH_2SiMe_3)_3(THF)_2$**, 2 equivalents $[Ph_3C][B(C_6F_5)_4]$, 1 equivalent PPh_3, and 5 equivalents Al^iBu_3 from **Table 4**, entry 10 (30 min). **S157**
Fig. S253. FT-IR spectrum of PIP 500 equivalents generated by **$Y(CH_2SiMe_3)_3(THF)_2$**, 2 equivalents $[Ph_3C][B(C_6F_5)_4]$, 1 equivalent PPh_3, and 10 equivalents Al^iBu_3 from **Table 4**, entry 11 (30 min). **S158**

Fig. S254. FT-IR spectrum of PIP 500 equivalents generated by **$Y(CH_2SiMe_3)_3(THF)_2$**, 2 equivalents $[Ph_3C][B(C_6F_5)_4]$, 1 equivalent PPh_3, and 15 equivalents Al^iBu_3 from **Table 4**, entry 12 (30 min). **S158**
Fig. S255. FT-IR spectrum of PIP 500 equivalents generated by **$Sm(CH_2SiMe_3)_3(THF)_3$** and 1 equivalent $[Ph_3C][B(C_6F_5)_4]$ from **Table 5**, entry 1 (30 min). **S159**
Fig. S256. FT-IR spectrum of PIP 500 equivalents generated by **$Sm(CH_2SiMe_3)_3(THF)_3$** and 2 equivalents $[Ph_3C][B(C_6F_5)_4]$ from **Table 5**, entry 2 (30 min). **S159**
Fig. S257. FT-IR spectrum of PIP 500 equivalents generated by **$Gd(CH_2SiMe_3)_3(THF)_2$** and 1 equivalent $[Ph_3C][B(C_6F_5)_4]$ from **Table 5**, entry 3 (30 min). **S160**
Fig. S258. FT-IR spectrum of PIP 500 equivalents generated by **$Gd(CH_2SiMe_3)_3(THF)_2$** and 2 equivalents $[Ph_3C][B(C_6F_5)_4]$ from **Table 5**, entry 4 (30 min). **S160**
Fig. S259. FT-IR spectrum of PIP 500 equivalents generated by **$Y(CH_2SiMe_3)_3(THF)_2$** and 1 equivalent $[Ph_3C][B(C_6F_5)_4]$ from **Table 5**, entry 5 (30 min). **S161**
Fig. S260. FT-IR spectrum of PIP 500 equivalents generated by **$Y(CH_2SiMe_3)_3(THF)_2$** and 2 equivalents $[Ph_3C][B(C_6F_5)_4]$ from **Table 5**, entry 6 (30 min). **S161**
Fig. S261. FT-IR spectrum of PIP 500 equivalents generated by **$Tm(CH_2SiMe_3)_3(THF)_2$** and 1 equivalent $[Ph_3C][B(C_6F_5)_4]$ from **Table 5**, entry 7 (30 min). **S162**
Fig. S262. FT-IR spectrum of PIP 500 equivalents generated by **$Tm(CH_2SiMe_3)_3(THF)_2$** and 2 equivalents $[Ph_3C][B(C_6F_5)_4]$ from **Table 5**, entry 8 (30 min). **S162**
Fig. S263. FT-IR spectrum of PIP 500 equivalents generated by **$Sm(CH_2SiMe_3)_3(THF)_3$**, 1 equivalent $[Ph_3C][B(C_6F_5)_4]$, and 1 equivalent PPh_3 from **Table 5**, entry 9 (30 min). **S163**
Fig. S264. FT-IR spectrum of PIP 500 equivalents generated by **$Sm(CH_2SiMe_3)_3(THF)_3$**, 2 equivalents $[Ph_3C][B(C_6F_5)_4]$, and 1 equivalent PPh_3 from **Table 5**, entry 10 (30 min). **S163**
Fig. S265. FT-IR spectrum of PIP 500 equivalents generated by **$Gd(CH_2SiMe_3)_3(THF)_2$**, 1 equivalent $[Ph_3C][B(C_6F_5)_4]$, and 1 equivalent PPh_3 from **Table 5**, entry 11 (30 min). **S164**
Fig. S266. FT-IR spectrum of PIP 500 equivalents generated by **$Gd(CH_2SiMe_3)_3(THF)_2$**, 2 equivalents $[Ph_3C][B(C_6F_5)_4]$, and 1 equivalent PPh_3 from **Table 5**, entry 12 (30 min). **S164**
Fig. S267. FT-IR spectrum of PIP 500 equivalents generated by **$Y(CH_2SiMe_3)_3(THF)_2$**, 1 equivalent $[Ph_3C][B(C_6F_5)_4]$, and 1 equivalent PPh_3 from **Table 5**, entry 13 (30 min). **S165**
Fig. S268. FT-IR spectrum of PIP 500 equivalents generated by **$Y(CH_2SiMe_3)_3(THF)_2$**, 2 equivalents $[Ph_3C][B(C_6F_5)_4]$, and 1 equivalent PPh_3 from **Table 5**, entry 14 (30 min). **S165**
Fig. S269. FT-IR spectrum of PIP 500 equivalents generated by **$Tm(CH_2SiMe_3)_3(THF)_2$**, 1 equivalent $[Ph_3C][B(C_6F_5)_4]$, and 1 equivalent PPh_3 from **Table 5**, entry 15 (30 min). **S166**
Fig. S270. FT-IR spectrum of PIP 500 equivalents generated by **$Tm(CH_2SiMe_3)_3(THF)_2$**, 2 equivalents $[Ph_3C][B(C_6F_5)_4]$, and 1 equivalent PPh_3 from **Table 5**, entry 16 (30 min). **S166**
Fig. S271. FT-IR spectrum of PIP 500 equivalents generated by **$Sm(CH_2SiMe_3)_3(THF)_3$,** 1 equivalent PPh_3, and 2 equivalents $[Ph_3C][B(C_6F_5)_4]$ from **Table 6**, entry 1 ($[Ph_3C][B(C_6F_5)_4]$ addition time 0 min). **S167**
Fig. S272. FT-IR spectrum of PIP 500 equivalents generated by **$Sm(CH_2SiMe_3)_3(THF)_3$**, 1 equivalent PPh_3, and 2 equivalents $[Ph_3C][B(C_6F_5)_4]$ from **Table 6**, entry 2 ($[Ph_3C][B(C_6F_5)_4]$ addition time 10 min). **S167**
Fig. S273. FT-IR spectrum of PIP 500 equivalents generated by **$Sm(CH_2SiMe_3)_3(THF)_3$**, 1 equivalent PPh_3, and 2 equivalents $[Ph_3C][B(C_6F_5)_4]$ from **Table 6**, entry 3 ($[Ph_3C][B(C_6F_5)_4]$ addition time 30 min). **S168**
Fig. S274. FT-IR spectrum of PIP 500 equivalents generated by **$Gd(CH_2SiMe_3)_3(THF)_2$**, 1 equivalent PPh_3, and 2 equivalents $[Ph_3C][B(C_6F_5)_4]$ from **Table 6**, entry 4 ($[Ph_3C][B(C_6F_5)_4]$ addition time 0 min). **S168**
Fig. S275. FT-IR spectrum of PIP 500 equivalents generated by **$Gd(CH_2SiMe_3)_3(THF)_2$**, 1 equivalent PPh_3, and 2 equivalents $[Ph_3C][B(C_6F_5)_4]$ from **Table 6**, entry 5 ($[Ph_3C][B(C_6F_5)_4]$ addition time 10 min). **S169**

Fig. S276. FT-IR spectrum of PIP 500 equivalents generated by **$Gd(CH_2SiMe_3)_3(THF)_2$**, 1 equivalent PPh_3, and 2 equivalents $[Ph_3C][B(C_6F_5)_4]$ from **Table 6**, entry 6 ($[Ph_3C][B(C_6F_5)_4]$ addition time 30 min). **S169**
Fig. S277. FT-IR spectrum of PIP 500 equivalents generated by **$Y(CH_2SiMe_3)_3(THF)_2$**, 1 equivalent PPh_3, and 2 equivalents $[Ph_3C][B(C_6F_5)_4]$ from **Table 6**, entry 7 ($[Ph_3C][B(C_6F_5)_4]$ addition time 0 min). **S170**
Fig. S278. FT-IR spectrum of PIP 500 equivalents generated by **$Y(CH_2SiMe_3)_3(THF)_2$**, 1 equivalent PPh_3, and 2 equivalents $[Ph_3C][B(C_6F_5)_4]$ from **Table 6**, entry 8 ($[Ph_3C][B(C_6F_5)_4]$ addition time 10 min). **S170**
Fig. S279. FT-IR spectrum of PIP 500 equivalents generated by **$Y(CH_2SiMe_3)_3(THF)_2$**, 1 equivalent PPh_3, and 2 equivalents $[Ph_3C][B(C_6F_5)_4]$ from **Table 6**, entry 9 ($[Ph_3C][B(C_6F_5)_4]$ addition time 30 min). **S171**
Fig. S280. FT-IR spectrum of PIP 500 equivalents generated by **$Tm(CH_2SiMe_3)_3(THF)_2$**, 1 equivalent PPh_3, and 2 equivalents $[Ph_3C][B(C_6F_5)_4]$ from **Table 6**, entry 10 ($[Ph_3C][B(C_6F_5)_4]$ addition time 0 min). **S171**
Fig. S281. FT-IR spectrum of PIP 500 equivalents generated by **$Tm(CH_2SiMe_3)_3(THF)_2$**, 1 equivalent PPh_3, and 2 equivalents $[Ph_3C][B(C_6F_5)_4]$ from **Table 6**, entry 11 ($[Ph_3C][B(C_6F_5)_4]$ addition time 10 min). **S172**
Fig. S282. FT-IR spectrum of PIP 500 equivalents generated by **$Tm(CH_2SiMe_3)_3(THF)_2$**, 1 equivalent PPh_3, and 2 equivalents $[Ph_3C][B(C_6F_5)_4]$ from **Table 6**, entry 12 ($[Ph_3C][B(C_6F_5)_4]$ addition time 30 min). **S172**
Fig. S283. FT-IR spectrum of PIP 500 equivalents generated by **$Sm(CH_2SiMe_3)_3(THF)_3$**, 2 equivalents $[Ph_3C][B(C_6F_5)_4]$, and 1 equivalent PPh_3 from **Table 7**, entry 1 (PPh_3 addition time 0 min). **S173**
Fig. S284. FT-IR spectrum of PIP 500 equivalents generated by **$Sm(CH_2SiMe_3)_3(THF)_3$**, 2 equivalents $[Ph_3C][B(C_6F_5)_4]$, and 1 equivalent PPh_3 from **Table 7**, entry 2 (PPh_3 addition time 10 min). **S173**
Fig. S285. FT-IR spectrum of PIP 500 equivalents generated by **$Sm(CH_2SiMe_3)_3(THF)_3$**, 2 equivalents $[Ph_3C][B(C_6F_5)_4]$, and 1 equivalent PPh_3 from **Table 7**, entry 3 (PPh_3 addition time 30 min). **S174**
Fig. S286. FT-IR spectrum of PIP 500 equivalents generated by **$Gd(CH_2SiMe_3)_3(THF)_2$**, 2 equivalents $[Ph_3C][B(C_6F_5)_4]$, and 1 equivalent PPh_3 from **Table 7**, entry 4 (PPh_3 addition time 0 min). **S174**
Fig. S287. FT-IR spectrum of PIP 500 equivalents generated by **$Gd(CH_2SiMe_3)_3(THF)_2$**, 2 equivalents $[Ph_3C][B(C_6F_5)_4]$, and 1 equivalent PPh_3 from **Table 7**, entry 5 (PPh_3 addition time 10 min). **S175**
Fig. S288. FT-IR spectrum of PIP 500 equivalents generated by **$Gd(CH_2SiMe_3)_3(THF)_2$**, 2 equivalents $[Ph_3C][B(C_6F_5)_4]$, and 1 equivalent PPh_3 from **Table 7**, entry 6 (PPh_3 addition time 30 min). **S175**
Fig. S289. FT-IR spectrum of PIP 500 equivalents generated by **$Y(CH_2SiMe_3)_3(THF)_2$**, 2 equivalents $[Ph_3C][B(C_6F_5)_4]$, and 1 equivalent PPh_3 from **Table 7**, entry 7 (PPh_3 addition time 0 min). **S176**
Fig. S290. FT-IR spectrum of PIP 500 equivalents generated by **$Y(CH_2SiMe_3)_3(THF)_2$**, 2 equivalents $[Ph_3C][B(C_6F_5)_4]$, and 1 equivalent PPh_3 from **Table 7**, entry 8 (PPh_3 addition time 10 min). **S176**
Fig. S291. FT-IR spectrum of PIP 500 equivalents generated by **$Y(CH_2SiMe_3)_3(THF)_2$**, 2 equivalents $[Ph_3C][B(C_6F_5)_4]$, and 1 equivalent PPh_3 from **Table 7**, entry 9 (PPh_3 addition time 30 min). **S177**
Fig. S292. FT-IR spectrum of PIP 500 equivalents generated by **$Tm(CH_2SiMe_3)_3(THF)_2$**, 2 equivalents $[Ph_3C][B(C_6F_5)_4]$, and 1 equivalent PPh_3 from **Table 7**, entry 10 (PPh_3 addition time 0 min). **S177**

Fig. S293. FT-IR spectrum of PIP 500 equivalents generated by **$Tm(CH_2SiMe_3)_3(THF)_2$**, 2 equivalents $[Ph_3C][B(C_6F_5)_4]$, and 1 equivalent PPh_3 from **Table 7**, entry 11 (PPh_3 addition time 10 min). S178
Fig. S294. FT-IR spectrum of PIP 500 equivalents generated by **$Tm(CH_2SiMe_3)_3(THF)_2$**, 2 equivalents $[Ph_3C][B(C_6F_5)_4]$, and 1 equivalent PPh_3 from **Table 7**, entry 12 (PPh_3 addition time 30 min). S178
Fig. S295. FT-IR spectrum of PIP 500 equivalents generated by **$Y(CH_2SiMe_3)_3(THF)_2$** and 2 equivalents $[Ph_3C][B(C_6F_5)_4]$ from **Table 7**, entry 13 (IP addition time 10 min). S179
Fig. S296. FT-IR spectrum of PIP 500 equivalents generated by **$Y(CH_2SiMe_3)_3(THF)_2$** and 2 equivalents $[Ph_3C][B(C_6F_5)_4]$ from **Table 6**, entry 14 (IP addition time 20 min). S179
Fig. S297. FT-IR spectrum of PIP 500 equivalents generated by **$Y(CH_2SiMe_3)_3(THF)_2$** and 2 equivalents $[Ph_3C][B(C_6F_5)_4]$ from **Table 7**, entry 15 (IP addition time 40 min). S180
Fig. S298. FT-IR spectrum of PIP 500 equivalents generated by **$Y(CH_2SiMe_3)_3(THF)_2$**, 2 equivalents $[Ph_3C][B(C_6F_5)_4]$, and 1 equivalent PPh_3 from **Table 8**, entry 1 (Step 1). S180
Fig. S299. FT-IR spectrum of PIP 250 equivalents generated by **$Y(CH_2SiMe_3)_3(THF)_2$**, 2 equivalents $[Ph_3C][B(C_6F_5)_4]$, and 1 equivalent PPh_3 from **Table 8**, entry 2 (Step 2). S181
Fig. S300. FT-IR spectrum of PIP 125 equivalents generated by **$Y(CH_2SiMe_3)_3(THF)_2$**, 2 equivalents $[Ph_3C][B(C_6F_5)_4]$, and 1 equivalent PPh_3 from **Table 8**, entry 3 (Step 3). S181
Fig. S301. FT-IR spectrum of PIP 500 equivalents generated by **$Y(CH_2SiMe_3)_3(THF)_2$** and 2 equivalents $[Ph_3C][B(C_6F_5)_4]$ from **Table 8**, entry 4 (Step 1). S182
Fig. S302. FT-IR spectrum of PIP 250 equivalents generated by **$Y(CH_2SiMe_3)_3(THF)_2$** and 2 equivalents $[Ph_3C][B(C_6F_5)_4]$ from **Table 8**, entry 5 (Step 2). S182
Fig. S303. FT-IR spectrum of PIP 125 equivalents generated by **$Y(CH_2SiMe_3)_3(THF)_2$** and 2 equivalents $[Ph_3C][B(C_6F_5)_4]$ from **Table 8**, entry 6 (Step 3). S183
5.0 Gel Permeation Chromatography (GPC) Characterization of Isolated Polymers **S184**
Fig. S304. GPC spectrum of PIP 500 equivalents generated by **$Y(CH_2SiMe_3)_3(THF)_2$** and 1 equivalent $[Ph_3C][B(C_6F_5)_4]$ from **Table 1**, entry 1 (30 min): (left) LS; (right) RI. S184
Fig. S305. GPC spectrum of PIP 500 equivalents generated by **$Y(CH_2SiMe_3)_3(THF)_2$** and 2 equivalents $[Ph_3C][B(C_6F_5)_4]$ from **Table 1**, entry 2 (30 min): (left) LS; (right) RI. S184
Fig. S306. GPC spectrum of PIP 500 equivalents generated by **$Y(CH_2SiMe_3)_3(THF)_2$**, 2 equivalents $[Ph_3C][B(C_6F_5)_4]$, and 1 equivalent Bipy from **Table 2**, entry 3 (30 min): (left) LS; (right) RI. S184
Fig. S307. GPC spectrum of PIP 500 equivalents generated by **$Y(CH_2SiMe_3)_3(THF)_2$**, 2 equivalents $[Ph_3C][B(C_6F_5)_4]$, and 1 equivalent MeCN from **Table 2**, entry 5 (30 min): (left) LS; (right) RI. S185
Fig. S308. GPC spectrum of PIP 500 equivalents generated by **$Y(CH_2SiMe_3)_3(THF)_2$**, 2 equivalents $[Ph_3C][B(C_6F_5)_4]$, and 1 equivalent P(*o*-tolyl)$_3$ from **Table 2**, entry 6 (30 min): (left) LS; (right) RI. S185
Fig. S309. GPC spectrum of PIP 500 equivalents generated by **$Y(CH_2SiMe_3)_3(THF)_2$**, 2 equivalents $[Ph_3C][B(C_6F_5)_4]$, and 1 equivalent PCy_3 from **Table 2**, entry 7 (30 min): (left) LS; (right) RI. S185
Fig. S310. GPC spectrum of PIP 500 equivalents generated by **$Y(CH_2SiMe_3)_3(THF)_2$**, 2 equivalents $[Ph_3C][B(C_6F_5)_4]$, and 1 equivalent PPh_3 from **Table 2**, entry 8 (30 min): (left) LS; (right) RI. S186
Fig. S311. GPC spectrum of PIP 500 equivalents generated by **$Y(CH_2SiMe_3)_3(THF)_2$**, 2 equivalents $[Ph_3C][B(C_6F_5)_4]$, and 1 equivalent P(Ph-*p*-OMe)$_3$ from **Table S1**, entry 1 (30 min): (left) LS; (right) RI. S186
Fig. S312. GPC spectrum of PIP 500 equivalents generated by **$Y(CH_2SiMe_3)_3(THF)_2$**, 2 equivalents $[Ph_3C][B(C_6F_5)_4]$, and 1 equivalent P(*p*-tolyl)$_3$ from **Table S1**, entry 2 (30 min): (left) LS; (right) RI. S186

Fig. S313. GPC spectrum of PIP 500 equivalents generated by **$Y(CH_2SiMe_3)_3(THF)_2$**, 2 equivalents $[Ph_3C][B(C_6F_5)_4]$, and 1 equivalent $P(Ph\text{-}p\text{-}F)_3$ from **Table S1**, entry 4 (30 min): (left) LS; (right) RI. **S187**
Fig. S314. GPC spectrum of PIP 500 equivalents generated by **$Y(CH_2SiMe_3)_3(THF)_2$** and 2 equivalents $[Ph_3C][B(C_6F_5)_4]$ from **Table S2**, entry 1 (5 min): (left) LS; (right) RI. **S187**
Fig. S315. GPC spectrum of PIP 500 equivalents generated by **$Y(CH_2SiMe_3)_3(THF)_2$** and 2 equivalents $[Ph_3C][B(C_6F_5)_4]$ from **Table S2**, entry 2 (12 min): (left) LS; (right) RI. **S187**
Fig. S316. GPC spectrum of PIP 500 equivalents generated by **$Y(CH_2SiMe_3)_3(THF)_2$** and 2 equivalents $[Ph_3C][B(C_6F_5)_4]$ from **Table S2**, entry 3 (18 min): (left) LS; (right) RI. **S188**
Fig. S317. GPC spectrum of PIP 500 equivalents generated by **$Y(CH_2SiMe_3)_3(THF)_2$** and 2 equivalents $[Ph_3C][B(C_6F_5)_4]$ from **Table S2**, entry 4 (24 min): (left) LS; (right) RI. **S188**
Fig. S318. GPC spectrum of PIP 500 equivalents generated by **$Y(CH_2SiMe_3)_3(THF)_2$** and 2 equivalents $[Ph_3C][B(C_6F_5)_4]$ from **Table S2**, entry 5 (30 min): (left) LS; (right) RI. **S188**
Fig. S319. GPC spectrum of PIP 500 equivalents generated by **$Y(CH_2SiMe_3)_3(THF)_2$**, 2 equivalents $[Ph_3C][B(C_6F_5)_4]$, and 1 equivalent PPh_3 from **Table S3**, entry 1 (10 min): (left) LS; (right) RI. **S189**
Fig. S320. GPC spectrum of PIP 500 equivalents generated by **$Y(CH_2SiMe_3)_3(THF)_2$**, 2 equivalents $[Ph_3C][B(C_6F_5)_4]$, and 1 equivalent PPh_3 from **Table S3**, entry 2 (21 min): (left) LS; (right) RI. **S189**
Fig. S321. GPC spectrum of PIP 500 equivalents generated by **$Y(CH_2SiMe_3)_3(THF)_2$**, 2 equivalents $[Ph_3C][B(C_6F_5)_4]$, and 1 equivalent PPh_3 from **Table S3**, entry 3 (31 min): (left) LS; (right) RI. **S189**
Fig. S322. GPC spectrum of PIP 500 equivalents generated by **$Y(CH_2SiMe_3)_3(THF)_2$**, 2 equivalents $[Ph_3C][B(C_6F_5)_4]$, and 1 equivalent PPh_3 from **Table S3**, entry 4 (41 min): (left) LS; (right) RI. **S190**
Fig. S323. GPC spectrum of PIP 500 equivalents generated by **$Y(CH_2SiMe_3)_3(THF)_2$**, 2 equivalents $[Ph_3C][B(C_6F_5)_4]$, and 1 equivalent PPh_3 from **Table S3**, entry 5 (51 min): (left) LS; (right) RI. **S190**
Fig. S324. GPC spectrum of PIP 500 equivalents generated by **$Sm(CH_2SiMe_3)_3(THF)_3$** and 1 equivalent $[Ph_3C][B(C_6F_5)_4]$ from **Table S4**, entry 1 (7 h): (left) LS; (right) RI. **S190**
Fig. S325. GPC spectrum of PIP 500 equivalents generated by **$Gd(CH_2SiMe_3)_3(THF)_2$** and 1 equivalent $[Ph_3C][B(C_6F_5)_4]$ from **Table S4**, entry 2 (7 h): (left) LS; (right) RI. **S191**
Fig. S326. GPC spectrum of PIP 500 equivalents generated by **$Gd(CH_2SiMe_3)_3(THF)_2$** and 2 equivalents $[Ph_3C][B(C_6F_5)_4]$ from **Table S4**, entry 3 (7 h): (left) LS; (right) RI. **S191**
Fig. S327. GPC spectrum of PIP 500 equivalents generated by **$Tm(CH_2SiMe_3)_3(THF)_2$** and 1 equivalent $[Ph_3C][B(C_6F_5)_4]$ from **Table S4**, entry 4 (7 h): (left) LS; (right) RI. **S191**
Fig. S328. GPC spectrum of PIP 500 equivalents generated by **$Tm(CH_2SiMe_3)_3(THF)_2$** and 2 equivalents $[Ph_3C][B(C_6F_5)_4]$ from **Table S4**, entry 5 (7 h): (left) LS; (right) RI. **S192**
Fig. S329. GPC spectrum of PIP 500 equivalents generated by **$Sm(CH_2SiMe_3)_3(THF)_3$**, 1 equivalent $[Ph_3C][B(C_6F_5)_4]$, and 1 equivalent PPh_3 from **Table S4**, entry 6 (7 h): (left) LS; (right) RI. **S192**
Fig. S330. GPC spectrum of PIP 500 equivalents generated by **$Gd(CH_2SiMe_3)_3(THF)_2$**, 1 equivalent $[Ph_3C][B(C_6F_5)_4]$, and 1 equivalent PPh_3 from **Table S4**, entry 7 (7 h): (left) LS; (right) RI. **S192**
Fig. S331. GPC spectrum of PIP 500 equivalents generated by **$Gd(CH_2SiMe_3)_3(THF)_2$**, 2 equivalents $[Ph_3C][B(C_6F_5)_4]$, and 1 equivalent PPh_3 from **Table S4**, entry 8 (7 h): (left) LS; (right) RI. **S193**
Fig. S332. GPC spectrum of PIP 500 equivalents generated by **$Tm(CH_2SiMe_3)_3(THF)_2$**, 1 equivalent $[Ph_3C][B(C_6F_5)_4]$, and 1 equivalent PPh_3 from **Table S4**, entry 9 (7 h): (left) LS; (right) RI. **S193**

Fig. S333. GPC spectrum of PIP 500 equivalents generated by **$Tm(CH_2SiMe_3)_3(THF)_2$**, 2 equivalents $[Ph_3C][B(C_6F_5)_4]$, and 1 equivalent PPh_3 from **Table S4**, entry 10 (7 h): (left) LS; (right) RI. **S193**
Fig. S334. GPC spectrum of PIP 500 equivalents generated by **$Y(CH_2SiMe_3)_3(THF)_2$** and 1 equivalent $[Ph_3C][B(C_6F_5)_4]$ from **Table 3**, entry 1 (7 h): (left) LS; (right) RI. **S194**
Fig. S335. GPC spectrum of PIP 500 equivalents generated by **$Y(CH_2SiMe_3)_3(THF)_2$**, 1 equivalent $[Ph_3C][B(C_6F_5)_4]$, and 1 equivalent PPh_3 from **Table 3**, entry 2 (7 h): (left) LS; (right) RI. **S194**
Fig. S336. GPC spectrum of PIP 500 equivalents generated by **$Y(CH_2SiMe_3)_3(THF)_2$** and 1.5 equivalents $[Ph_3C][B(C_6F_5)_4]$ from **Table 3**, entry 3 (7 h): (left) LS; (right) RI. **S194**
Fig. S337. GPC spectrum of PIP 500 equivalents generated by **$Y(CH_2SiMe_3)_3(THF)_2$**, 1.5 equivalents $[Ph_3C][B(C_6F_5)_4]$, and 1 equivalent PPh_3 from **Table 3**, entry 4 (7 h): (left) LS; (right) RI. **S195**
Fig. S338. GPC spectrum of PIP 500 equivalents generated by **$Y(CH_2SiMe_3)_3(THF)_2$** and 2 equivalents $[Ph_3C][B(C_6F_5)_4]$ from **Table 3**, entry 5 (7 h): (left) LS; (right) RI. **S195**
Fig. S339. GPC spectrum of PIP 500 equivalents generated by **$Y(CH_2SiMe_3)_3(THF)_2$**, 2 equivalents $[Ph_3C][B(C_6F_5)_4]$, and 1 equivalent PPh_3 from **Table 3**, entry 6 (7 h): (left) LS; (right) RI. **S195**
Fig. S340. GPC spectrum of PIP 500 equivalents generated by **$Y(CH_2SiMe_3)_3(THF)_2$** and 2.5 equivalents $[Ph_3C][B(C_6F_5)_4]$ from **Table 3**, entry 7 (7 h): (left) LS; (right) RI. **S196**
Fig. S341. GPC spectrum of PIP 500 equivalents generated by **$Y(CH_2SiMe_3)_3(THF)_2$**, 2.5 equivalents $[Ph_3C][B(C_6F_5)_4]$, and 1 equivalent PPh_3 from **Table 3**, entry 8 (7 h): (left) LS; (right) RI. **S196**
Fig. S342. GPC spectrum of PIP 500 equivalents generated by **$Y(CH_2SiMe_3)_3(THF)_2$** and 3 equivalents $[Ph_3C][B(C_6F_5)_4]$ from **Table 3**, entry 9 (7 h): (left) LS; (right) RI. **S196**
Fig. S343. GPC spectrum of PIP 500 equivalents generated by **$Y(CH_2SiMe_3)_3(THF)_2$**, 3 equivalents $[Ph_3C][B(C_6F_5)_4]$, and 1 equivalent PPh_3 from **Table 3**, entry 10 (7 h): (left) LS; (right) RI. **S197**
Fig. S344. GPC spectrum of PIP 500 equivalents generated by **$Y(CH_2SiMe_3)_3(THF)_2$**, 2 equivalents $[Ph_3C][B(C_6F_5)_4]$, and 5 equivalents $AlMe_3$ from **Table 4**, entry 1 (30 min): (left) LS; (right) RI. **S197**
Fig. S345. GPC spectrum of PIP 500 equivalents generated by **$Y(CH_2SiMe_3)_3(THF)_2$**, 2 equivalents $[Ph_3C][B(C_6F_5)_4]$, and 10 equivalents $AlMe_3$ from **Table 4**, entry 2 (30 min): (left) LS; (right) RI. **S197**
Fig. S346. GPC spectrum of PIP 500 equivalents generated by **$Y(CH_2SiMe_3)_3(THF)_2$**, 2 equivalents $[Ph_3C][B(C_6F_5)_4]$, and 15 equivalents $AlMe_3$ from **Table 4**, entry 3 (30 min): (left) LS; (right) RI. **S198**
Fig. S347. GPC spectrum of PIP 500 equivalents generated by **$Y(CH_2SiMe_3)_3(THF)_2$**, 2 equivalents $[Ph_3C][B(C_6F_5)_4]$, and 5 equivalents $AlEt_3$ from **Table 4**, entry 4 (30 min): (left) LS; (right) RI. **S198**
Fig. S348. GPC spectrum of PIP 500 equivalents generated by **$Y(CH_2SiMe_3)_3(THF)_2$**, 2 equivalents $[Ph_3C][B(C_6F_5)_4]$, and 10 equivalents $AlEt_3$ from **Table 4**, entry 5 (30 min): (left) LS; (right) RI. **S198**
Fig. S349. GPC spectrum of PIP 500 equivalents generated by **$Y(CH_2SiMe_3)_3(THF)_2$**, 2 equivalents $[Ph_3C][B(C_6F_5)_4]$, and 15 equivalents $AlEt_3$ from **Table 4**, entry 6 (30 min): (left) LS; (right) RI. **S199**
Fig. S350. GPC spectrum of PIP 500 equivalents generated by **$Y(CH_2SiMe_3)_3(THF)_2$**, 2 equivalents $[Ph_3C][B(C_6F_5)_4]$, and 5 equivalents Al^iBu_3 from **Table 4**, entry 7 (30 min): (left) LS; (right) RI. **S199**

Fig. S351. GPC spectrum of PIP 500 equivalents generated by **$Y(CH_2SiMe_3)_3(THF)_2$**, 2 equivalents $[Ph_3C][B(C_6F_5)_4]$, and 10 equivalents Al^iBu_3 from **Table 4**, entry 8 (30 min): (left) LS; (right) RI. **S199**
Fig. S352. GPC spectrum of PIP 500 equivalents generated by **$Y(CH_2SiMe_3)_3(THF)_2$**, 2 equivalents $[Ph_3C][B(C_6F_5)_4]$, and 15 equivalents Al^iBu_3 from **Table 4**, entry 9 (30 min): (left) LS; (right) RI. **S200**
Fig. S353. GPC spectrum of PIP 500 equivalents generated by **$Y(CH_2SiMe_3)_3(THF)_2$**, 2 equivalents $[Ph_3C][B(C_6F_5)_4]$, 1 equivalent PPh_3, and 5 equivalents Al^iBu_3 from **Table 4**, entry 10 (30 min): (left) LS; (right) RI. **S200**
Fig. S354. GPC spectrum of PIP 500 equivalents generated by **$Y(CH_2SiMe_3)_3(THF)_2$**, 2 equivalents $[Ph_3C][B(C_6F_5)_4]$, 1 equivalent PPh_3, and 10 equivalents Al^iBu_3 from **Table 4**, entry 11 (30 min): (left) LS; (right) RI. **S200**
Fig. S355. GPC spectrum of PIP 500 equivalents generated by **$Y(CH_2SiMe_3)_3(THF)_2$**, 2 equivalents $[Ph_3C][B(C_6F_5)_4]$, 1 equivalent PPh_3, and 15 equivalents Al^iBu_3 from **Table 4**, entry 12 (30 min): (left) LS; (right) RI. **S201**
Fig. S356. GPC spectrum of PIP 500 equivalents generated by **$Sm(CH_2SiMe_3)_3(THF)_3$** and 1 equivalent $[Ph_3C][B(C_6F_5)_4]$ from **Table 5**, entry 1 (30 min): (left) LS; (right) RI. **S201**
Fig. S357. GPC spectrum of PIP 500 equivalents generated by **$Sm(CH_2SiMe_3)_3(THF)_3$** and 2 equivalents $[Ph_3C][B(C_6F_5)_4]$ from **Table 5**, entry 2 (30 min): (left) LS; (right) RI. **S201**
Fig. S358. GPC spectrum of PIP 500 equivalents generated by **$Gd(CH_2SiMe_3)_3(THF)_2$** and 1 equivalent $[Ph_3C][B(C_6F_5)_4]$ from **Table 5**, entry 3 (30 min): (left) LS; (right) RI. **S202**
Fig. S359. GPC spectrum of PIP 500 equivalents generated by **$Gd(CH_2SiMe_3)_3(THF)_2$** and 2 equivalents $[Ph_3C][B(C_6F_5)_4]$ from **Table 5**, entry 4 (30 min): (left) LS; (right) RI. **S202**
Fig. S360. GPC spectrum of PIP 500 equivalents generated by **$Y(CH_2SiMe_3)_3(THF)_2$** and 1 equivalent $[Ph_3C][B(C_6F_5)_4]$ from **Table 5**, entry 5 (30 min): (left) LS; (right) RI. **S202**
Fig. S361. GPC spectrum of PIP 500 equivalents generated by **$Y(CH_2SiMe_3)_3(THF)_2$** and 2 equivalents $[Ph_3C][B(C_6F_5)_4]$ from **Table 5**, entry 6 (30 min): (left) LS; (right) RI. **S203**
Fig. S362. GPC spectrum of PIP 500 equivalents generated by **$Tm(CH_2SiMe_3)_3(THF)_2$** and 1 equivalent $[Ph_3C][B(C_6F_5)_4]$ from **Table 5**, entry 7 (30 min): (left) LS; (right) RI. **S203**
Fig. S363. GPC spectrum of PIP 500 equivalents generated by **$Tm(CH_2SiMe_3)_3(THF)_2$** and 2 equivalents $[Ph_3C][B(C_6F_5)_4]$ from **Table 5**, entry 8 (30 min): (left) LS; (right) RI. **S203**
Fig. S364. GPC spectrum of PIP 500 equivalents generated by **$Sm(CH_2SiMe_3)_3(THF)_3$**, 1 equivalent $[Ph_3C][B(C_6F_5)_4]$, and 1 equivalent PPh_3 from **Table 5**, entry 9 (30 min): (left) LS; (right) RI. **S204**
Fig. S365. GPC spectrum of PIP 500 equivalents generated by **$Sm(CH_2SiMe_3)_3(THF)_3$**, 2 equivalents $[Ph_3C][B(C_6F_5)_4]$, and 1 equivalent PPh_3 from **Table 5**, entry 10 (30 min): (left) LS; (right) RI. **S204**
Fig. S366. GPC spectrum of PIP 500 equivalents generated by **$Gd(CH_2SiMe_3)_3(THF)_2$**, 1 equivalent $[Ph_3C][B(C_6F_5)_4]$, and 1 equivalent PPh_3 from **Table 5**, entry 11 (30 min): (left) LS; (right) RI. **S204**
Fig. S367. GPC spectrum of PIP 500 equivalents generated by **$Gd(CH_2SiMe_3)_3(THF)_2$**, 2 equivalents $[Ph_3C][B(C_6F_5)_4]$, and 1 equivalent PPh_3 from **Table 5**, entry 12 (30 min): (left) LS; (right) RI. **S205**
Fig. S368. GPC spectrum of PIP 500 equivalents generated by **$Y(CH_2SiMe_3)_3(THF)_2$**, 1 equivalent $[Ph_3C][B(C_6F_5)_4]$, and 1 equivalent PPh_3 from **Table 5**, entry 13 (30 min): (left) LS; (right) RI. **S205**
Fig. S369. GPC spectrum of PIP 500 equivalents generated by **$Y(CH_2SiMe_3)_3(THF)_2$**, 2 equivalents $[Ph_3C][B(C_6F_5)_4]$, and 1 equivalent PPh_3 from **Table 5**, entry 14 (30 min): (left) LS; (right) RI. **S205**

Fig. S370. GPC spectrum of PIP 500 equivalents generated by **$Tm(CH_2SiMe_3)_3(THF)_2$**, 1 equivalent $[Ph_3C][B(C_6F_5)_4]$, and 1 equivalent PPh_3 from **Table 5**, entry 15 (30 min): (left) LS; (right) RI. **S206**
Fig. S371. GPC spectrum of PIP 500 equivalents generated by **$Tm(CH_2SiMe_3)_3(THF)_2$**, 2 equivalents $[Ph_3C][B(C_6F_5)_4]$, and 1 equivalent PPh_3 from **Table 5**, entry 16 (30 min): (left) LS; (right) RI. **S206**
Fig. S372. GPC spectrum of PIP 500 equivalents generated by **$Sm(CH_2SiMe_3)_3(THF)_3$,** 1 equivalent PPh_3, and 2 equivalents $[Ph_3C][B(C_6F_5)_4]$ from **Table 6**, entry 1 ($[Ph_3C][B(C_6F_5)_4]$ addition time 0 min): (left) LS; (right) RI. **S206**
Fig. S373. GPC spectrum of PIP 500 equivalents generated by **$Sm(CH_2SiMe_3)_3(THF)_3$**, 1 equivalent PPh_3, and 2 equivalents $[Ph_3C][B(C_6F_5)_4]$ from **Table 6**, entry 2 ($[Ph_3C][B(C_6F_5)_4]$ addition time 10 min): (left) LS; (right) RI. **S207**
Fig. S374. GPC spectrum of PIP 500 equivalents generated by **$Sm(CH_2SiMe_3)_3(THF)_3$**, 1 equivalent PPh_3 and 2 equivalents $[Ph_3C][B(C_6F_5)_4]$ from **Table 6**, entry 3 ($[Ph_3C][B(C_6F_5)_4]$ addition time 30 min): (left) LS; (right) RI. **S207**
Fig. S375. GPC spectrum of PIP 500 equivalents generated by **$Gd(CH_2SiMe_3)_3(THF)_2$**, 1 equivalent PPh_3, and 2 equivalents $[Ph_3C][B(C_6F_5)_4]$ from **Table 6**, entry 4 ($[Ph_3C][B(C_6F_5)_4]$ addition time 0 min): (left) LS; (right) RI. **S207**
Fig. S376. GPC spectrum of PIP 500 equivalents generated by **$Gd(CH_2SiMe_3)_3(THF)_2$**, 1 equivalent PPh_3, and 2 equivalents $[Ph_3C][B(C_6F_5)_4]$ from **Table 6**, entry 5 ($[Ph_3C][B(C_6F_5)_4]$ addition time 10 min): (left) LS; (right) RI. **S208**
Fig. S377. GPC spectrum of PIP 500 equivalents generated by **$Gd(CH_2SiMe_3)_3(THF)_2$**, 1 equivalent PPh_3, and 2 equivalents $[Ph_3C][B(C_6F_5)_4]$ from **Table 6**, entry 6 ($[Ph_3C][B(C_6F_5)_4]$ addition time 30 min): (left) LS; (right) RI. **S208**
Fig. S378. GPC spectrum of PIP 500 equivalents generated by **$Y(CH_2SiMe_3)_3(THF)_2$**, 1 equivalent PPh_3, and 2 equivalents $[Ph_3C][B(C_6F_5)_4]$ from **Table 6**, entry 7 ($[Ph_3C][B(C_6F_5)_4]$ addition time 0 min): (left) LS; (right) RI. **S208**
Fig. S379. GPC spectrum of PIP 500 equivalents generated by **$Y(CH_2SiMe_3)_3(THF)_2$**, 1 equivalent PPh_3, and 2 equivalents $[Ph_3C][B(C_6F_5)_4]$ from **Table 6**, entry 8 ($[Ph_3C][B(C_6F_5)_4]$ addition time 10 min): (left) LS; (right) RI. **S209**
Fig. S380. GPC spectrum of PIP 500 equivalents generated by **$Y(CH_2SiMe_3)_3(THF)_2$**, 1 equivalent PPh_3, and 2 equivalents $[Ph_3C][B(C_6F_5)_4]$ from **Table 6**, entry 9 ($[Ph_3C][B(C_6F_5)_4]$ addition time 30 min): (left) LS; (right) RI. **S209**
Fig. S381. GPC spectrum of PIP 500 equivalents generated by **$Tm(CH_2SiMe_3)_3(THF)_2$**, 1 equivalent PPh_3, and 2 equivalents $[Ph_3C][B(C_6F_5)_4]$ from **Table 6**, entry 10 ($[Ph_3C][B(C_6F_5)_4]$ addition time 0 min): (left) LS; (right) RI. **S209**
Fig. S382. GPC spectrum of PIP 500 equivalents generated by **$Tm(CH_2SiMe_3)_3(THF)_2$**, 1 equivalent PPh_3, and 2 equivalents $[Ph_3C][B(C_6F_5)_4]$ from **Table 6**, entry 11 ($[Ph_3C][B(C_6F_5)_4]$ addition time 10 min): (left) LS; (right) RI. **S210**
Fig. S383. GPC spectrum of PIP 500 equivalents generated by **$Tm(CH_2SiMe_3)_3(THF)_2$**, 1 equivalent PPh_3 and 2 equivalents $[Ph_3C][B(C_6F_5)_4]$ from **Table 6**, entry 12 ($[Ph_3C][B(C_6F_5)_4]$ addition time 30 min): (left) LS; (right) RI. **S210**
Fig. S384. GPC spectrum of PIP 500 equivalents generated by **$Sm(CH_2SiMe_3)_3(THF)_3$**, 2 equivalents $[Ph_3C][B(C_6F_5)_4]$, and 1 equivalent PPh_3 from **Table 7**, entry 1 (PPh_3 addition time 0 min): (left) LS; (right) RI. **S210**
Fig. S385. GPC spectrum of PIP 500 equivalents generated by **$Sm(CH_2SiMe_3)_3(THF)_3$**, 2 equivalents $[Ph_3C][B(C_6F_5)_4]$, and 1 equivalent PPh_3 from **Table 7**, entry 2 (PPh_3 addition time 10 min): (left) LS; (right) RI. **S211**
Fig. S386. GPC spectrum of PIP 500 equivalents generated by **$Sm(CH_2SiMe_3)_3(THF)_3$**, 2 equivalents $[Ph_3C][B(C_6F_5)_4]$, and 1 equivalent PPh_3 from **Table 7**, entry 3 (PPh_3 addition time 30 min): (left) LS; (right) RI. **S211**

Fig. S387. GPC spectrum of PIP 500 equivalents generated by **$Gd(CH_2SiMe_3)_3(THF)_2$**, 2 equivalents $[Ph_3C][B(C_6F_5)_4]$, and 1 equivalent PPh_3 from **Table 7**, entry 4 (PPh_3 addition time 0 min): (left) LS; (right) RI. **S211**
Fig. S388. GPC spectrum of PIP 500 equivalents generated by **$Gd(CH_2SiMe_3)_3(THF)_2$**, 2 equivalents $[Ph_3C][B(C_6F_5)_4]$, and 1 equivalent PPh_3 from **Table 7**, entry 5 (PPh_3 addition time 10 min): (left) LS; (right) RI. **S212**
Fig. S389. GPC spectrum of PIP 500 equivalents generated by **$Gd(CH_2SiMe_3)_3(THF)_2$**, 2 equivalents $[Ph_3C][B(C_6F_5)_4]$, and 1 equivalent PPh_3 from **Table 7**, entry 6 (PPh_3 addition time 30 min): (left) LS; (right) RI. **S212**
Fig. S390. GPC spectrum of PIP 500 equivalents generated by **$Y(CH_2SiMe_3)_3(THF)_2$**, 2 equivalents $[Ph_3C][B(C_6F_5)_4]$, and 1 equivalent PPh_3 from **Table 7**, entry 7 (PPh_3 addition time 0 min): (left) LS; (right) RI. **S212**
Fig. S391. GPC spectrum of PIP 500 equivalents generated by **$Y(CH_2SiMe_3)_3(THF)_2$**, 2 equivalents $[Ph_3C][B(C_6F_5)_4]$, and 1 equivalent PPh_3 from **Table 7**, entry 8 (PPh_3 addition time 10 min): (left) LS; (right) RI. **S213**
Fig. S392. GPC spectrum of PIP 500 equivalents generated by **$Y(CH_2SiMe_3)_3(THF)_2$**, 2 equivalents $[Ph_3C][B(C_6F_5)_4]$, and 1 equivalent PPh_3 from **Table 7**, entry 9 (PPh_3 addition time 30 min): (left) LS; (right) RI. **S213**
Fig. S393. GPC spectrum of PIP 500 equivalents generated by **$Tm(CH_2SiMe_3)_3(THF)_2$**, 2 equivalents $[Ph_3C][B(C_6F_5)_4]$, and 1 equivalent PPh_3 from **Table 7**, entry 10 (PPh_3 addition time 0 min): (left) LS; (right) RI. **S213**
Fig. S394. GPC spectrum of PIP 500 equivalents generated by **$Tm(CH_2SiMe_3)_3(THF)_2$**, 2 equivalents $[Ph_3C][B(C_6F_5)_4]$, and 1 equivalent PPh_3 from **Table 7**, entry 11 (PPh_3 addition time 10 min): (left) LS; (right) RI. **S214**
Fig. S395. GPC spectrum of PIP 500 equivalents generated by **$Tm(CH_2SiMe_3)_3(THF)_2$**, 2 equivalents $[Ph_3C][B(C_6F_5)_4]$, and 1 equivalent PPh_3 from **Table 7**, entry 12 (PPh_3 addition time 30 min): (left) LS; (right) RI. **S214**
Fig. S396. GPC spectrum of PIP 500 equivalents generated by **$Y(CH_2SiMe_3)_3(THF)_2$**, and 2 equivalents $[Ph_3C][B(C_6F_5)_4]$ from **Table 7**, entry 13 (IP addition time 10 min): (left) LS; (right) RI. **S214**
Fig. S397. GPC spectrum of PIP 500 equivalents generated by **$Y(CH_2SiMe_3)_3(THF)_2$**, and 2 equivalents $[Ph_3C][B(C_6F_5)_4]$ from **Table 7**, entry 14 (IP addition time 20 min): (left) LS; (right) RI. **S215**
Fig. S398. GPC spectrum of PIP 500 equivalents generated by **$Y(CH_2SiMe_3)_3(THF)_2$**, and 2 equivalents $[Ph_3C][B(C_6F_5)_4]$ from **Table 7**, entry 15 (IP addition time 40 min): (left) LS; (right) RI. **S215**
Fig. S399. GPC spectrum of PIP 500 equivalents generated by **$Y(CH_2SiMe_3)_3(THF)_2$**, 2 equivalents $[Ph_3C][B(C_6F_5)_4]$, and 1 equivalent PPh_3 from **Table 8**, entry 1 (Step 1): (left) LS; (right) RI. **S215**
Fig. S400. GPC spectrum of PIP 250 equivalents generated by **$Y(CH_2SiMe_3)_3(THF)_2$**, 2 equivalents $[Ph_3C][B(C_6F_5)_4]$, and 1 equivalent PPh_3 from **Table 8**, entry 2 (Step 2): (left) LS; (right) RI. **S216**
Fig. S401. GPC spectrum of PIP 125 equivalents generated by **$Y(CH_2SiMe_3)_3(THF)_2$**, 2 equivalents $[Ph_3C][B(C_6F_5)_4]$, and 1 equivalent PPh_3 from **Table 8**, entry 3 (Step 3): (left) LS; (right) RI. **S216**
Fig. S402. GPC spectrum of PIP 500 equivalents generated by **$Y(CH_2SiMe_3)_3(THF)_2$** and 2 equivalents $[Ph_3C][B(C_6F_5)_4]$ from **Table 8**, entry 4 (Step 1): (left) LS; (right) RI. **S216**
Fig. S403. GPC spectrum of PIP 250 equivalents generated by **$Y(CH_2SiMe_3)_3(THF)_2$** and 2 equivalents $[Ph_3C][B(C_6F_5)_4]$ from **Table 8**, entry 5 (Step 2): (left) LS; (right) RI. **S217**
Fig. S404. GPC spectrum of PIP 125 equivalents generated by **$Y(CH_2SiMe_3)_3(THF)_2$** and 2 equivalents $[Ph_3C][B(C_6F_5)_4]$ from **Table 8**, entry 6 (Step 3): (left) LS; (right) RI. **S217**

6.0 **Thermogravimetric Analysis (TGA) of $Y(CH_2SiMe_3)_3(THF)_2$** **S218**
Fig. S405. TGA curve of $Y(CH_2SiMe_3)_3(THF)_2$. **S218**
7.0 ***In situ* NMR Studies** **S219**
7.1 ***In situ* synthesis of $[Y(CH_2SiMe_3)_2(THF)_2]^+[B(C_6F_5)_4]^-$ and $[Y(CH_2SiMe_3)(THF)_2]^{2+}[B(C_6F_5)_4]^{2-}$** **S219**
Fig. S406. *In situ* ^1H NMR spectrum of the monocationic active species $[Y(CH_2SiMe_3)_2(THF)_2]^+[B(C_6F_5)_4]^-$ from the reaction of complex $Y(CH_2SiMe_3)_3(THF)_2$ with 1 equiv. $[Ph_3C][B(C_6F_5)_4]$ in toluene-d_8 at room temperature. **S219**
Fig. S407. *In situ* ^1H NMR spectrum of the monocationic active species $[Y(CH_2SiMe_3)_2(THF)_2]^+[B(C_6F_5)_4]^-$ from the reaction of complex $Y(CH_2SiMe_3)_3(THF)_2$ with 1 equiv. $[Ph_3C][B(C_6F_5)_4]$ after IP addition in toluene-d_8 at room temperature. **S220**
Fig. S408. *In situ* ^{19}F NMR spectrum of the monocationic active species $[Y(CH_2SiMe_3)_2(THF)_2]^+[B(C_6F_5)_4]^-$ from the reaction of complex $Y(CH_2SiMe_3)_3(THF)_2$ with 1 equiv. $[Ph_3C][B(C_6F_5)_4]$ in toluene-d_8 at room temperature. **S220**
Fig. S409. *In situ* ^1H NMR spectrum of the dicationic active species $[Y(CH_2SiMe_3)(THF)_2]^{2+}[B(C_6F_5)_4]^{2-}$ from the reaction of complex $Y(CH_2SiMe_3)_3(THF)_2$ with 2 equiv. $[Ph_3C][B(C_6F_5)_4]$ in toluene-d_8 at room temperature. **S221**
Fig. S410. *In situ* ^{19}F NMR spectrum of the dicationic active species $[Y(CH_2SiMe_3)(THF)_2]^{2+}[B(C_6F_5)_4]^{2-}$ from the reaction of complex $Y(CH_2SiMe_3)_3(THF)_2$ with 1 equiv. $[Ph_3C][B(C_6F_5)_4]$ in toluene-d_8 at room temperature. **S221**
7.2 ***In situ* NMR Studies with PPh_3** **S222**
Fig. S411. *In situ* ^{31}P NMR spectrum of the dicationic active species $[Y(CH_2SiMe_3)(THF)_2]^{2+}[B(C_6F_5)_4]^{2-}$ from the reaction of complex $Y(CH_2SiMe_3)_3(THF)_2$ with 2 equiv. $[Ph_3C][B(C_6F_5)_4]$ and 1 equiv. PPh_3 added 10 min after activation in toluene-d_8 at room temperature. **S222**
Fig. S412. *In situ* ^{31}P NMR spectrum of 1 equiv. PPh_3 with 2 equiv. $[Ph_3C][B(C_6F_5)_4]$ in toluene-d_8 at room temperature. **S223**
Fig. S413. *In situ* ^{31}P NMR spectrum of the dicationic active species $[Y(CH_2SiMe_3)(THF)_2]^{2+}[B(C_6F_5)_4]^{2-}$ from the reaction of complex $Y(CH_2SiMe_3)_3(THF)_2$ with 2 equiv. $[Ph_3C][B(C_6F_5)_4]$ and 1 equiv. PPh_3 in toluene-d_8 at -80 ^oC. **S224**
Fig. S414. DOSY NMR spectrum of the dicationic active species $[Y(CH_2SiMe_3)(THF)_2]^{2+}[B(C_6F_5)_4]^{2-}$ from the reaction of complex $Y(CH_2SiMe_3)_3(THF)_2$ with 2 equiv. $[Ph_3C][B(C_6F_5)_4]$ in toluene-d_8 at room temperature. **S225**
Fig. S415. DOSY NMR spectrum of the dicationic active species $[Y(CH_2SiMe_3)(THF)_2]^{2+}[B(C_6F_5)_4]^{2-}$ from the reaction of complex $Y(CH_2SiMe_3)_3(THF)_2$ with 2 equiv. $[Ph_3C][B(C_6F_5)_4]$ and 1 equiv. PPh_3 in toluene-d_8 at room temperature. **S226**
Fig. S416. *In situ* ^1H NMR spectrum of the dicationic active species $[Y(CH_2SiMe_3)(THF)_2]^{2+}[B(C_6F_5)_4]^{2-}$ from the reaction of complex $Y(CH_2SiMe_3)_3(THF)_2$ with 2 equiv. $[Ph_3C][B(C_6F_5)_4]$ and PPh_3 added at time 0 min in toluene-d_8 at room temperature. **S227**
Fig. S417. *In situ* ^1H NMR spectrum of the dicationic active species $[Y(CH_2SiMe_3)(THF)_2]^{2+}[B(C_6F_5)_4]^{2-}$ from the reaction of complex $Y(CH_2SiMe_3)_3(THF)_2$ with 2 equiv. $[Ph_3C][B(C_6F_5)_4]$ and PPh_3 added at time 10 min in toluene-d_8 at room temperature. **S228**
Fig. S418. *In situ* ^1H NMR spectrum of the dicationic active species $[Y(CH_2SiMe_3)(THF)_2]^{2+}[B(C_6F_5)_4]^{2-}$from the reaction of complex $Y(CH_2SiMe_3)_3(THF)_2$ with 2 equiv. $[Ph_3C][B(C_6F_5)_4]$ and PPh_3 added at time 30 min in toluene-d_8 at room temperature. **S229**
7.3 ***In situ* reaction of $Y(CH_2SiMe_3)_3(THF)_2$ with 3 equiv. $[Ph_3C][B(C_6F_5)_4]$**. **S230**
Fig. S419. *In situ* ^1H NMR spectrum of the reaction of complex $Y(CH_2SiMe_3)_3(THF)_2$ with 3 equiv. $[Ph_3C][B(C_6F_5)_4]$ in toluene-d_8 at room temperature. **S230**

7.4 ***In situ* reaction of $Y(CH_2SiMe_3)_3(THF)_2$ with 2 equiv. $[Ph_3C][B(C_6F_5)_4]$ and 5 equiv. $AlMe_3$.** **S231**
Fig. S420. *In situ* ^1H NMR spectrum of the reaction of complex $Y(CH_2SiMe_3)_3(THF)_2$ with 2 equiv. $[Ph_3C][B(C_6F_5)_4]$ and 5 equiv. $AlMe_3$ in toluene-d_8 at room temperature. **S231**
Fig. S421. *In situ* ^{27}Al NMR spectrum of the reaction of complex $Y(CH_2SiMe_3)_3(THF)_2$ with 2 equiv. $[Ph_3C][B(C_6F_5)_4]$ and 5 equiv. $AlMe_3$ in toluene-d_8 at room temperature. **S231**

1.0 General Considerations

All reactions involving air and moisture sensitive compounds were carried out using Schlenk line techniques or in a Vacuum Atmospheres OMNI-LAB glovebox under an oxygen free, N_2 atmosphere. Solvents used in air free reactions (toluene, hexane, pentane, diethyl ether, and tetrahydrofuran) were purchased from Fisher, sparged under ultrahigh purity (UHP) grade argon and passed through two columns of drying agent in a JCMeyer solvent purification system and dispensed directly into the glovebox. All other solvents were used without further purification. Deuterated NMR solvents, C_6D_6 and $CDCl_3$, were purchased from Cambridge Isotope Laboratories and were used as received. $CDCl_3$ and C_6D_6 suitable for air sensitive compounds were dried using the following methods. C_6D_6 was dried by stirring over Na/benzophenone for two days, followed by three freeze-pump-thaw cycles and vacuum transferred into a flame-dried Straus flask and stored in a glovebox under a N_2 atmosphere. $CDCl_3$ was dried by stirring over 4Å molecular sieves for 3 days, followed by three freeze-pump-thaw cycles and vacuum transferred into a flame-dried Straus flask and stored in a glovebox under a N_2 atmosphere. The four rare earth metal pre-catalysts, $RE(CH_2SiMe_3)_3(THF)_n$ (RE= Tm, Y, Gd, n=2; RE= Sm, n=3), were synthesized following literature procedure.[1,2] Isoprene, purchased from Sigma-Aldrich, was dried over 4Å molecular sieves for 7 days, followed by three freeze-pump-thaw cycles and a vacuum transfer into a flame-dried Straus flask and stored in a glovebox at –35 ºC under a N_2 atmosphere. Bipyridine was purchased from Sigma-Aldrich and sublimed 3 times before being transferred and stored in a glovebox under a N_2 atmosphere. All other reagents and chemicals were obtained from commercial vendors (Sigma-Aldrich, TCI, Alfa Aesar, and VWR) and were used without further purification.

1.1 Polymerization Methods

Preparation of Stock Solutions for polymerizations

In a glovebox, RE trialkyl pre-catalysts was crystallized immediately following synthesis and stored at -35 ºC and used within two weeks. Stock solutions of trityl (tetrakis(pentafluorophenyl)borate $[Ph_3C][B(C_6F_5)_4]$ (20 μmol, 10 mL of a 2 M stock solution), RE trialkyl pre-catalyst (10 μmol, 200 μL of a 0.5 M stock solution) and donor (10 μmol, 600 μL of a 16 μM stock solution) were prepared using volumetric flasks and used within 12 h. Stock solutions were stored at -35 ºC when not in use. Stock solutions were warmed to rt prior to catalysis.

General procedure for homopolymerization of isoprene

In a glovebox, trityl (tetrakis(pentafluorophenyl)borate $[Ph_3C][B(C_6F_5)_4]$ (20 μmol, 10 mL of a 2 M stock solution) was placed in a stir bar charged 20 mL vial. RE trialkyl pre-catalyst (10 μmol, 200 μL of a 0.5 M stock solution) was added to the vial and the mixture was stirred for 10 min. In the cases where a donor was used, donor (10 μmol, 600 μL of a 16 μM stock solution) was then added by micro syringe and the reaction was stirred for an additional 10 min. Isoprene (500 equiv.) was added by micro syringe in one portion, and the polymerization was carried out for the designated time with constant stirring. The reaction mixture was removed from a glovebox and poured into a large quantity of ethanol (100 mL) to give colorless polymer as a precipitant. Collected polymer was redissolved in minimum chloroform and was washed with acetone to remove impurities and subsequently dried in a vacuum oven at 40 ºC for 12 h to a constant weight.

Aliquots were taken prior to quenching to determine the % conversion of the polymer. A 200 μL aliquot was placed in a dry NMR tube and quenched with $CDCl_3$ (500 μL). ^1H NMR was taken immediately following quenching.

The % conversion was calculated based on the following equation:

$$\% \, conversion = \frac{normalised \; integration \; for \; polymer \; (1{,}4 \; content \; peak + 3{,}4 \; content \; peak)}{normalised \; integration \; for \; polymer + \; normalised \; integration \; for \; monomer}$$

$$= \frac{\int(5.14 - 5.10 \; ppm) + \frac{\int(4.72 - 4.67 \; ppm)}{2}}{\int(5.14 - 5.10 \; ppm) + \frac{\int(4.72 - 4.67 \; ppm)}{2} + \int(6.5 - 6.42 \; ppm)} \quad (1)$$

1.2 Characterization Methods

Nuclear Magnetic Resonance Spectroscopy (NMR). ^1H and ^{13}C NMR spectra were recorded using a Varian Mercury 400 MHz, Varian 500 MHz, or Varian 600 MHz spectrometers. Chemical shifts are referenced to residual protons in the deuterated solvent or the deuterated solvent itself for ^1H (7.26 ppm for $CDCl_3$, 2.08 ppm for toluene-d_8) or ^{13}C (77.16 ppm for $CDCl_3$) NMR spectra. Description about the temperature and the deuterated solvent for each sample are recorded in the figure description. One ^1H NMR and one ^{13}C NMR for each experiment are shown as a representative. Isoprene selectivities are an average of duplicate runs. 1,4 and 3,4 selectivity are determined by ^1H NMR. The relevant signals corresponding to 1,4 and 3,4 content were found to be between 5.10-5.14 ppm and 4.67-4.72 ppm, respectively. These peaks were integrated, and the following equations were used to calculate the microstructure content:[3]

$$\% \; 1{,}4 \; content = \frac{\int(5.14 - 5.10 \; ppm)}{\int(5.14 - 5.10 \; ppm) + \frac{\int(4.72 - 4.67 \; ppm)}{2}} \quad (2)$$

$$\% \; 3{,}4 \; content = \frac{\frac{\int(4.72 - 4.67 \; ppm)}{2}}{\int(5.14 - 5.10 \; ppm) + \frac{\int(4.72 - 4.67 \; ppm)}{2}} \quad (3)$$

Cis-1,4 and *trans*-1,4 selectivity is determined by ^{13}C NMR with no NOE (1D sequence with inverse-gated ^1H-decoupling). The methyl carbons of *cis*-1,4 and *trans*-1,4 polyisoprene were found to be at 23.8 ppm and 16.2 ppm, respectively and were used to determine the microstructure content by the following equations:[4]

$$\% \; trans - 1{,}4 \; content = \frac{\int(16.2 \; ppm)}{\int(16.2 \; ppm) + \int(23.8 \; ppm)} \quad (4)$$

$$\% \; cis - 1{,}4 \; content = \frac{\int(23.8 \; ppm)}{\int(16.2 \; ppm) + \int(23.8 \; ppm)} \quad (5)$$

Fourier Transform Infrared Spectroscopy (FT-IR). FT-IR spectra were recorded using an Agilent Cary 630 FT-IR equipped with a Diamond ATR sampling accessory. Accompanying MicroLab FT-IR software was used to acquire 72 scans at 4 cm^{-1} resolution with a spectral range of 400-4000 cm^{-1}.

Gel Permeation Chromatography (GPC). GPC analyses were conducted using an Agilent 1260 Infinity II GPC System equipped with a Wyatt DAWN HELEOS-II and a Wyatt Optilab T-rEX as well as an Agilent 1260 Infinity autosampler and UV-detector. The GPC system was equipped with two Agilent PolyPore columns (5 micron, 4.6 mmID) which were calibrated using monodisperse polystyrene standards, eluted with THF at 30 °C at 0.3 mL/min. The number average molar mass and dispersity values were determined from multi-angle light scattering (MALS) using *dn/dc* values calculated by 100% mass recovery method from the refractive index (RI) signal.

1.3 Hammett plot

Following the already described *General procedure for homopolymerization of isoprene*, different substituted phosphine donors were added to a mixture of $[Ph_3C][B(C_6F_5)_4]$ (20 µmol, 10 mL of a 2 M stock solution) and $Y(CH_2SiMe_3)_3(THF)_2$ (10 µmol, 200 µL of a 0.5 M stock solution) and mixed for 10 minutes prior to the addition of isoprene (500 equiv.). Reactions were stirred for the specified time at rt before quenching in EtOH. Aliquot was taken and analyzed by ^{1}H NMR, to provide conversion data.

Table S1 IP polymerization with $\mathbf{Y(CH_2SiMe_3)_3(THF)_2}$, 2 equiv. $\mathbf{[Ph_3C][B(C_6F_5)_4]}$, and different para substituted donors.[a]

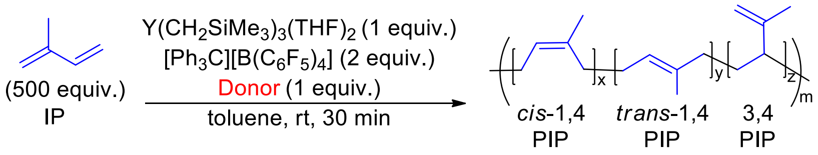

Entry	Donor	Time (min)	Conv. (%)[b]	$^{Theor}M_n$ (kDa)[c]	$^{Exp}M_n$ (kDa)[d]	Đ[d]	Microstructure[e] *Cis*-1,4/ *Trans*-1,4/3,4
**1**	P(Ph-*p*-OMe)$_3$	30	20(1)	7	12(2)	1.85(8)	48/31/21
**2**	P(*p*-tolyl)$_3$	30	36(2)	12	11(1)	1.78(15)	58/17/25
**3**	PPh$_3$	30	76(3)	26	33(2)	1.16(4)	61/13/26
**4**	P(Ph-*p*-F)$_3$	10	65(2)	22	67(5)	1.69	75/0/25

[a]Conditions: $\mathbf{Y(CH_2SiMe_3)_3(THF)_2}$, 10 µmol; $[Ph_3C][B(C_6F_5)_4]$(B), 20 µmol; toluene, 10 mL; 10 µmol Donor; [IP]/**Y**= 500. All entries are done in duplicate, and the error is denoted in parentheses. [b]Determined by ^{1}H NMR spectroscopy of crude reaction mixtures, comparing monomer peaks to polymer. [c]Calculated for one alkyl initiator, [IP mol/Y mol] x IP molecular weight x Conversion. [d]Determined by gel permeation chromatography (GPC) in THF using a Wyatt DAWN HELEOS II MALS detector. [e]All selectivity data is an average of duplicate runs. 1,4 and 3,4 selectivity determined by ^{1}H NMR. *Cis*-1,4 and *trans*-1,4 selectivity determined by ^{13}C NMR.

Log(k_x/k_H) was calculated using the following 1st order equation:

$$t_{1/2} = \frac{0.693}{k} \quad (6)$$

Donor	$T_{1/2}$ (min)	k_x/k_H	$Log(k_x/k_H)$
P(Ph-*p*-OMe)$_3$	75	0.146	-8.34
P(*p*-tolyl)$_3$	42	0.256	-0.584
PPh$_3$	11	1	0
P(Ph-*p*-F)$_3$	7.5	1.46	0.166

1.4 Living Polymerization

General procedure for time point studies

In a glovebox, $[Ph_3C][B(C_6F_5)_4]$ (47.2 μmol, 10 mL of a 2 M stock solution) was placed in a stir bar charged 20 mL Teflon capped Schlenk flask. RE trialkyl pre-catalyst (23.6 μmol, 200 μL of a 0.5 M stock solution) was add to the Schlenk flask and the mixture was stirred for 10 min. In the cases where a donor was used, donor (23.6 μmol, 600 μL of a 16 μM stock solution) was then added by micro syringe and the reaction was stirred for an additional 10 min. Isoprene (500 equiv.) was added by micro syringe in one portion, and the polymerization was stirred at 800 RPM. Aliquots were removed from the reaction at intervals throughout the polymerization and quenched with isopropanol. All quenched aliquots were removed from a glovebox and poured into a large quantity of ethanol (100 mL) to give colorless polymer precipitants. Collected polymer was redissolved in minimum chloroform and was washed with acetone to remove impurities and subsequently dried in a vacuum oven at 40 °C for 12 h to a constant weight.

Table S2 Living plot homopolymerization of IP with $\mathbf{Y(CH_2SiMe_3)_3(THF)_2}$.[a]

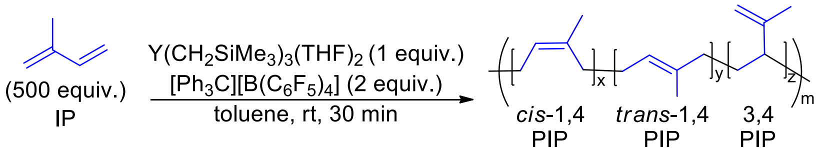

Entry	RE	Time (min)	Conv. (%)[b]	M_n (KDa)[c]	Đ[c]	Microstructure[d] *Cis*-1,4/ *Trans*-1,4/3,4
**1**	Y	5	30	27	1.58	79/0/21
**2**	Y	12	56	38	1.79	79/0/21
**3**	Y	18	76	44	1.72	82/0/18
**4**	Y	24	89	53	1.68	81/0/19
**5**	Y	30	91	54	1.74	81/0/19

[a]Conditions: $\mathbf{Y(CH_2SiMe_3)_3(THF)_2}$, 24 μmol; $[Ph_3C][B(C_6F_5)_4]$, 47 μmol; toluene, 26 mL; [IP]/**Y**=500. [b]Determined by ^1H NMR spectroscopy of crude reaction mixtures, comparing monomer peaks to polymer. [c]Determined by gel permeation chromatography (GPC) in THF using a Wyatt DAWN HELEOS II MALS detector. [d]1,4 and 3,4 selectivity determined by ^1H NMR. *Cis*-1,4 and *trans*-1,4 selectivity determined by ^{13}C NMR.

Table S3 Living plot homopolymerization of IP with **$Y(CH_2SiMe_3)_3(THF)_2$** and PPh_3.[a]

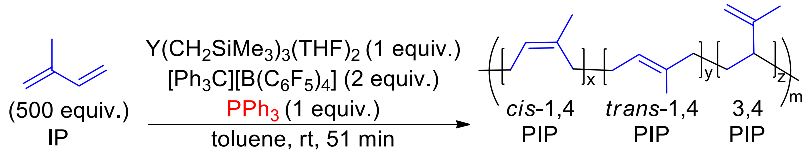

Entry	RE	Time (min)	Conv. (%)[b]	M_n (KDa)[c]	Đ[c]	Microstructure[d] *Cis*-1,4/ *Trans*-1,4/3,4
**1**	Y	10	49	17	1.1	71/5/24
**2**	Y	21	76	31	1.1	71/5/24
**3**	Y	31	87	38	1.15	64/7/29
**4**	Y	41	97	44	1.11	63/8/29
**5**	Y	51	99	47	1.12	63/9/28

[a]Conditions: **$Y(CH_2SiMe_3)_3(THF)_2$**, 24 μmol; $[Ph_3C][B(C_6F_5)_4]$, 47 μmol; toluene, 26 mL; [IP]/**Y**= 500; 24 μmol PPh_3. [b]Determined by ^1H NMR spectroscopy of crude reaction mixtures, comparing monomer peaks to polymer. [c]Determined by gel permeation chromatography (GPC) in THF using a Wyatt DAWN HELEOS II MALS detector. [d]1,4 and 3,4 selectivity determined by ^1H NMR. *Cis*-1,4 and *trans*-1,4 selectivity determined by ^{13}C NMR.

1.5 Extended Reaction Times for IP Polymerization with RE Trialkyl Complexes

Table S4 Homopolymerization of IP with RE trialkyl pre-catalysts both with and without PPh_3.[a]

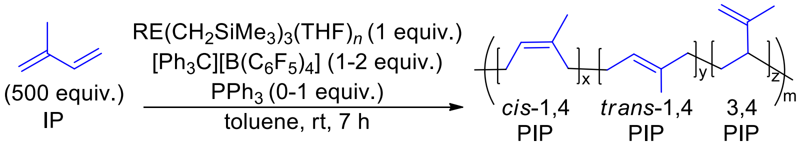

Entry	RE	Borate (B) (equiv.)	PPh_3 (P) (equiv.)	Conv. (%)[b]	${}^{Theor}M_n$ (kDa)[c]	${}^{Exp}M_n$ (kDa)[d]	Đ[d]	Microstructure[e] *Cis*-1,4/*Trans*-1,4/3,4
**1**	Sm	1	—	51(3)	17	48(2)	1.72(10)	58/30/20
**2**	Gd	1	—	85(8)	29	44(7)	1.22(11)	16/73/11
**3**	Gd	2	—	>99	34	89(7)	1.61(9)	69/10/21
**4**	Tm	1	—	74(1)	25	25(3)	1.36(5)	24/61/15
**5**	Tm	2	—	>99	34	54(1)	2.03(5)	61/10/29
**6**	Sm	1	1	54(4)	18	50(3)	1.42(6)	28/60/12
**7**	Gd	1	1	73(3)	25	51(6)	1.21(2)	17/70/13
**8**	Gd	2	1	>99	34	102(11)	1.21(7)	69/9/22
**9**	Tm	1	1	74(1)	25	21(2)	1.36(1)	28/57/15
**10**	Tm	2	1	>99	34	31(4)	1.39(14)	51/25/24

[a]Conditions: **$RE(CH_2SiMe_3)_3(THF)_n$** (RE= Sm, *n*=3, RE= Gd, Y, Tm, *n*=2), 10 μmol; $[Ph_3C][B(C_6F_5)_4]$(B), 10-20 μmol; toluene, 10 mL; [IP]/**RE**=500. All entries are done in duplicate, and the error is denoted in parentheses. [b]Determined by ^1H NMR spectroscopy of crude reaction mixtures, comparing monomer peaks to polymer. [c]Calculated for one alkyl initiator, [IP mol/RE mol] x IP molecular weight x Conversion. [d]Determined by gel permeation chromatography (GPC) in THF using a Wyatt DAWN HELEOS II MALS detector. [e]All selectivity data is an average of duplicate runs. 1,4 and 3,4 selectivity determined by ^1H NMR. *Cis*-1,4 and *trans*-1,4 selectivity determined by ^{13}C NMR.

2.0 References

[1] Schumann, H.; Freckmann, D. M. M.; Dechert, S. The Molecular Structure of Tris(trimethylsilylmethyl)samarium, -erbium, -ytterbium, and -lutetium. *Z. Anorg. Allg. Chem.* **2002**, *628*, 2422-2426.

[2] Chen, S-M.; Zhang, Y-Q.; Xiong, J.; Wang, B-W.; Gao, S. Adducts of Tris(alkyl) Holmium(III) Showing Magnetic Relaxation. *Inorg. Chem.* **2020**, *59*, 5835-5844.

[3] Beebe, D. H. Structure of 3,4-(Cis-1,4-)Trans-1,4-Polyisoprene by NMR. *Polymer* **1978**, *19*, 231-233.

[4] Tanaka, Y.; Sato, H.; Seimiya, T. ^{13}C-NMR of Polyisoprenes: Sequence Distribution of Cis-1,4 and Trans-1,4 Units. *Polymer J.* **1975**, *7*, 264-266.
