# Supplementary material for: Enhanced Control of Isoprene Polymerization with Trialkyl Rare Earth Metal Complexes through Neutral Donor Support
Source: Inorg Chem. 2023 Dec 8;63(21):9464–77. doi: 10.1021/acs.inorgchem.3c03161 (PMC11134520; doi:10.1021/acs.inorgchem.3c03161)
Supplement: Supplementary file 2 — ic3c03161_si_002.pdf [file ic3c03161_si_002.pdf]

3.0 Nuclear Magnetic Resonance (NMR) Characterization of Isolated Polymers

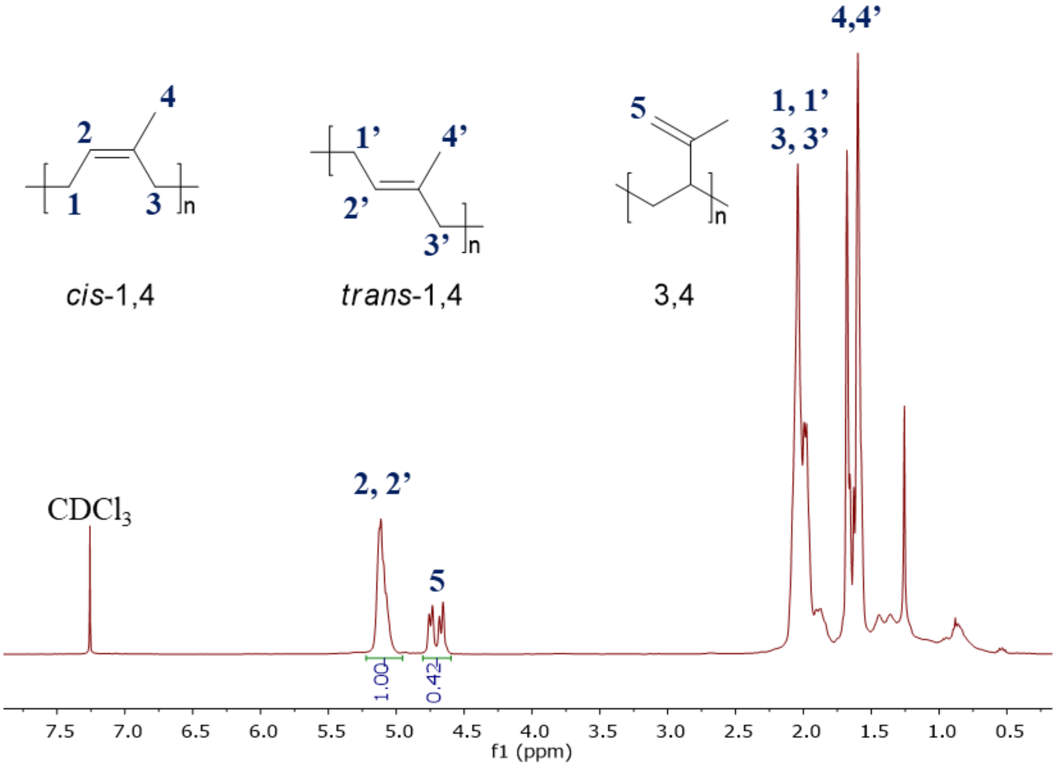

Fig. S1 ^{1}H NMR spectrum of PIP 500 equivalents generated by **$Y(CH_2SiMe_3)_3(THF)_2$** and 1 equivalent $[Ph_3C][B(C_6F_5)_4]$ from **Table 1**, entry 1 in $CDCl_3$ at 298 K (30 min). Representative peak assignment for PIP. Peak 5 used to calculate 3,4 content and peak 2, 2' used to calculate 1,4 content.

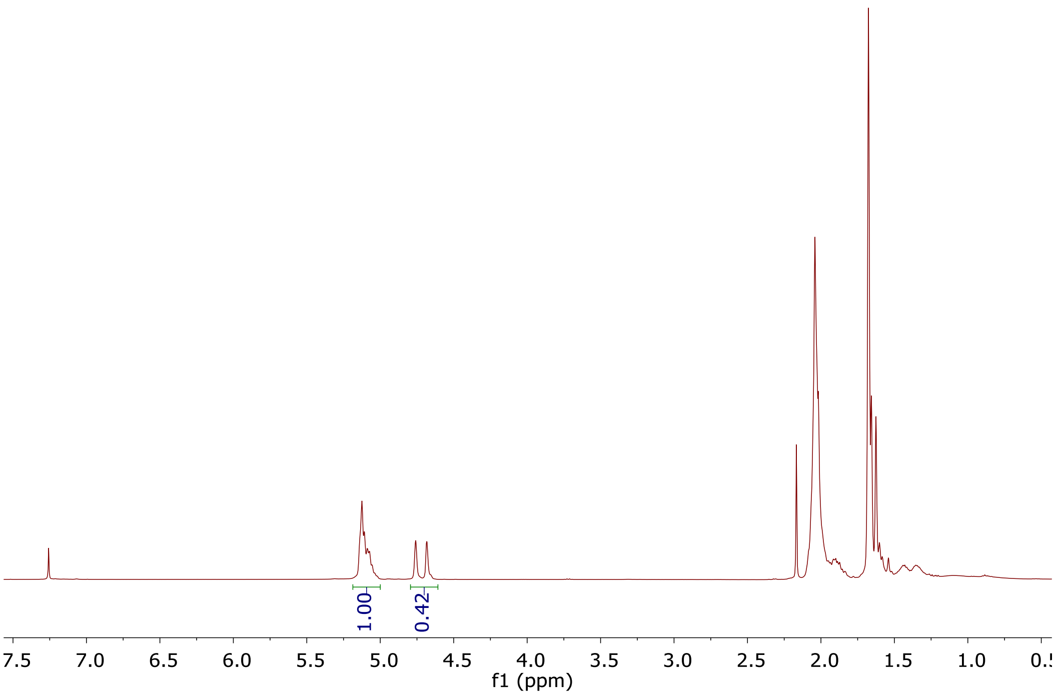

Fig. S2 ^{1}H NMR spectrum of PIP 500 equivalents generated by **$Y(CH_2SiMe_3)_3(THF)_2$** and 2 equivalents $[Ph_3C][B(C_6F_5)_4]$ from **Table 1**, entry 2 in $CDCl_3$ at 298 K (30 min).

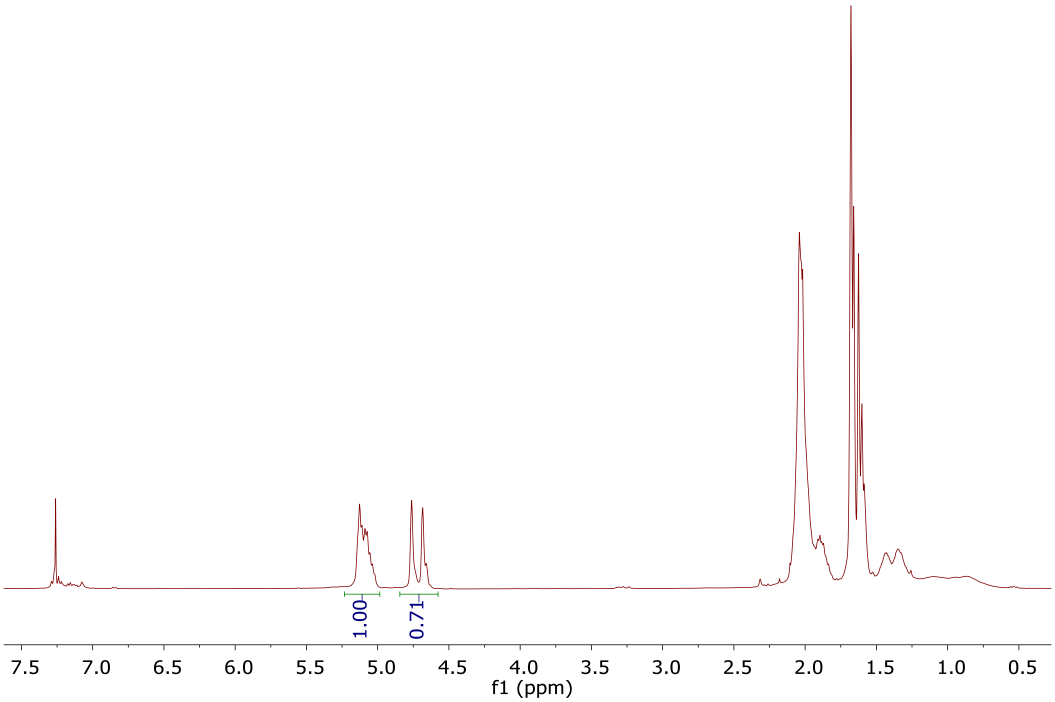

Fig. S3 ^{1}H NMR spectrum of PIP 500 equivalents generated by **Y(CH$_2$SiMe$_3$)$_3$(THF)$_2$**, 2 equivalents [Ph$_3$C][B(C$_6$F$_5$)$_4$], and 1 equivalent Bipy from **Table 2**, entry 3 in CDCl$_3$ at 298 K (30 min).

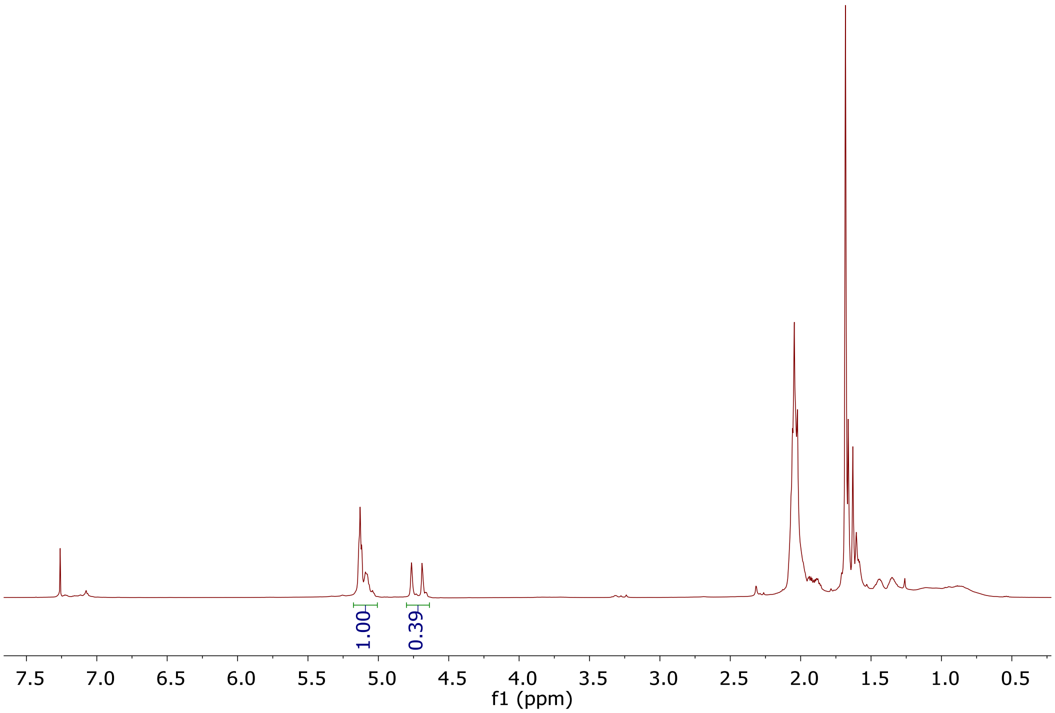

Fig. S4 ^{1}H NMR spectrum of PIP 500 equivalents generated by **Y(CH$_2$SiMe$_3$)$_3$(THF)$_2$**, 2 equivalents [Ph$_3$C][B(C$_6$F$_5$)$_4$], and 1 equivalent MeCN from **Table 2**, entry 5 in CDCl$_3$ at 298 K (30 min)

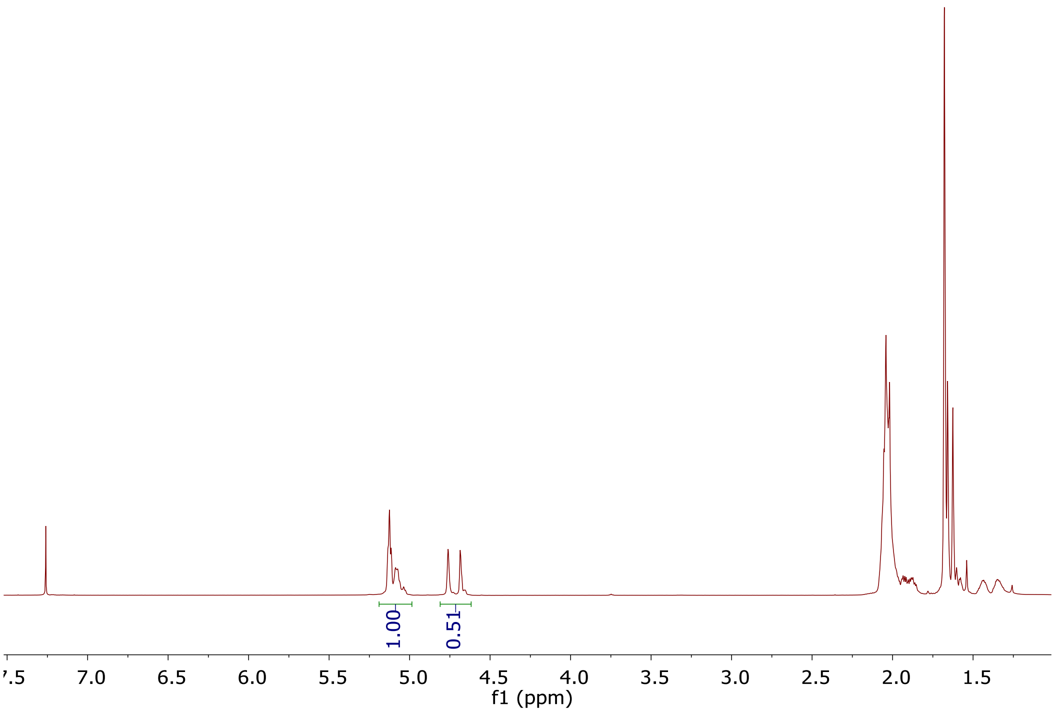

Fig. S5 ^{1}H NMR spectrum of PIP 500 equivalents generated by **Y(CH$_2$SiMe$_3$)$_3$(THF)$_2$**, 2 equivalents [Ph$_3$C][B(C$_6$F$_5$)$_4$], and 1 equivalent P(*o*-tolyl)$_3$ from **Table 2**, entry 6 in CDCl$_3$ at 298 K (30 min).

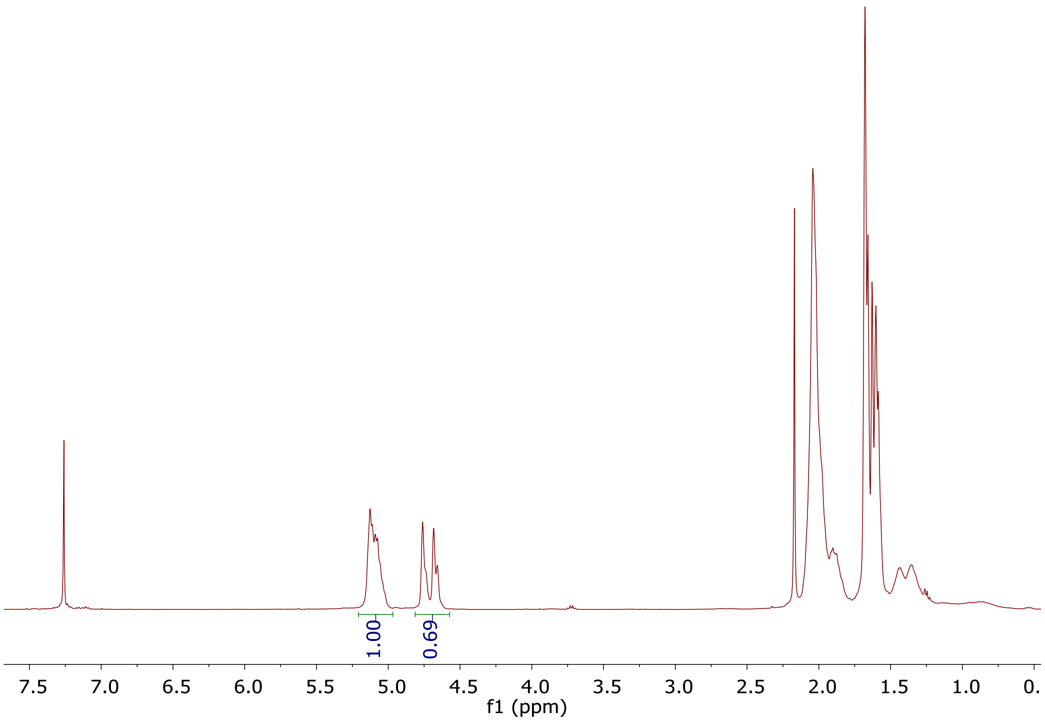

Fig. S6 ^{1}H NMR spectrum of PIP 500 equivalents generated by **Y(CH$_2$SiMe$_3$)$_3$(THF)$_2$**, 2 equivalents [Ph$_3$C][B(C$_6$F$_5$)$_4$], and 1 equivalent PCy$_3$ from **Table 2**, entry 7 in CDCl$_3$ at 298 K (30 min).

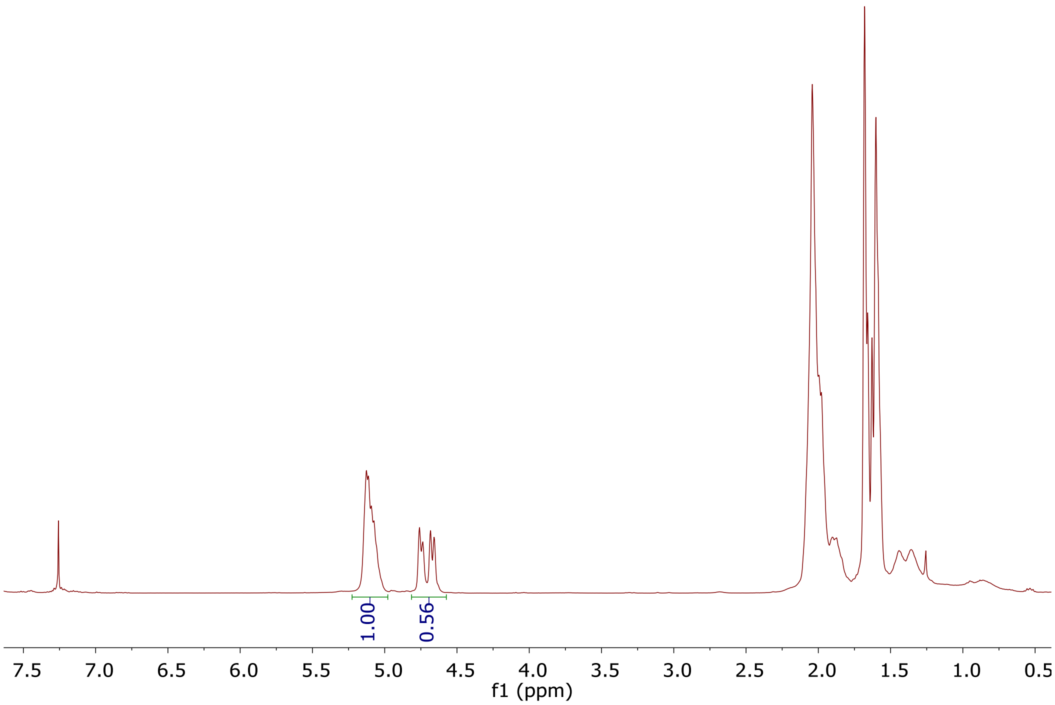

Fig. S7 ^{1}H NMR spectrum of PIP 500 equivalents generated by **$Y(CH_2SiMe_3)_3(THF)_2$**, 2 equivalents $[Ph_3C][B(C_6F_5)_4]$, and 1 equivalent PPh_3 from **Table 2**, entry 8 in $CDCl_3$ at 298 K (30 min).

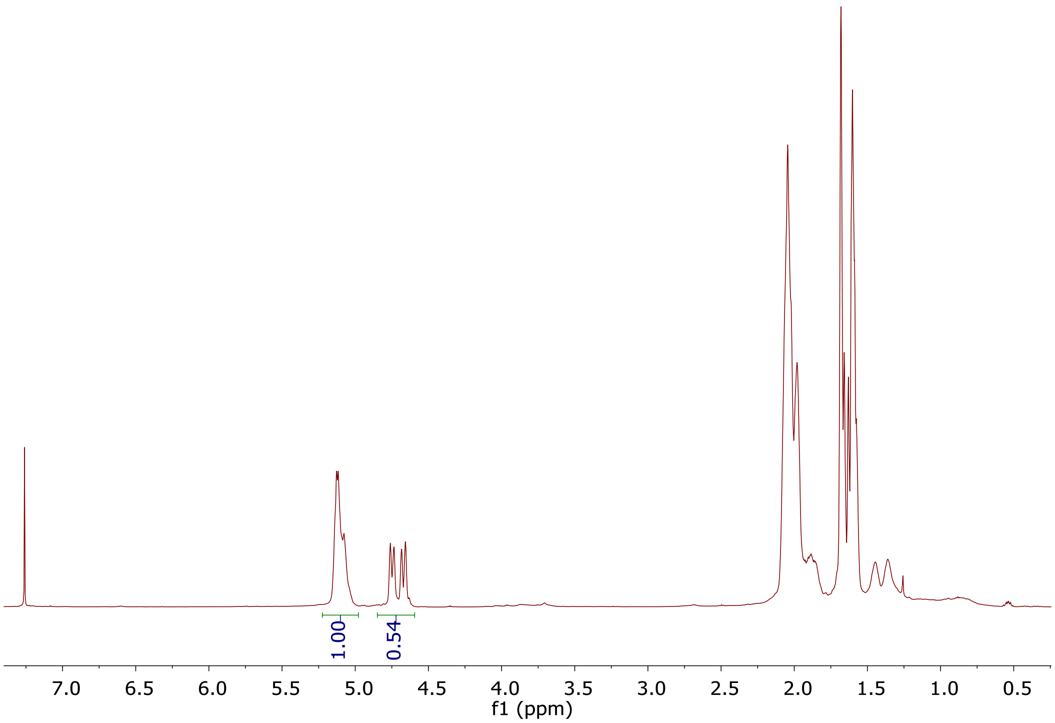

Fig. S8 ^{1}H NMR spectrum of PIP 500 equivalents generated by **$Y(CH_2SiMe_3)_3(THF)_2$**, 2 equivalents $[Ph_3C][B(C_6F_5)_4]$, and 1 equivalent P(Ph-*p*-OMe)$_3$ from **Table S1**, entry 1 in $CDCl_3$ at 298 K (30 min).

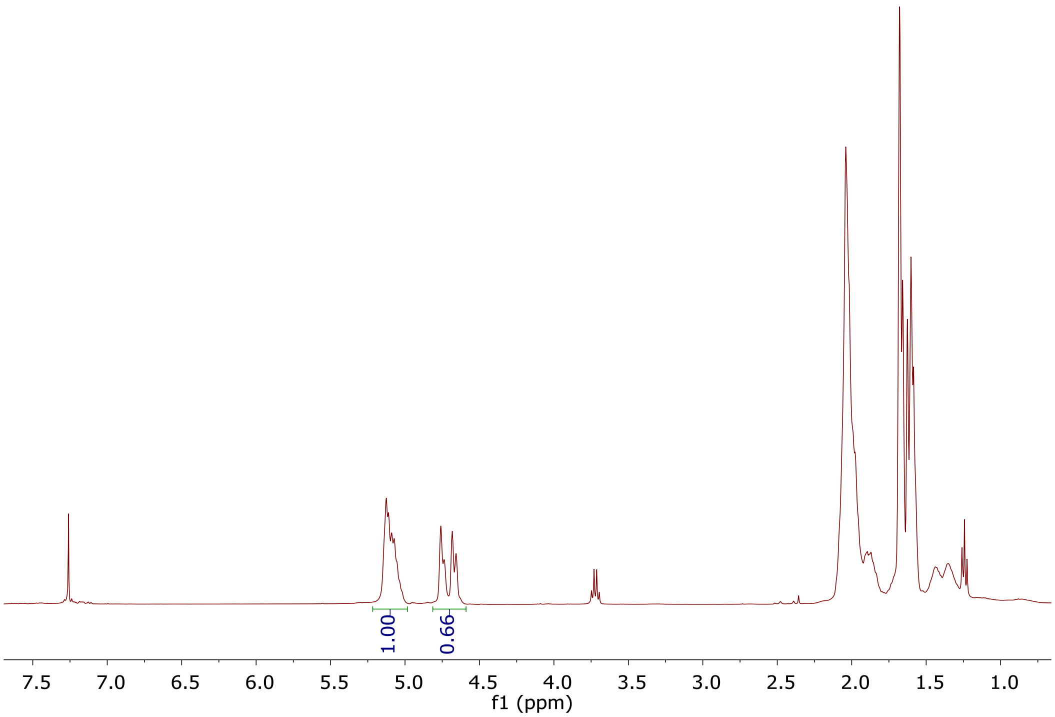

Fig. S9 ^{1}H NMR spectrum of PIP 500 equivalents generated by **Y(CH$_2$SiMe$_3$)$_3$(THF)$_2$**, 2 equivalents [Ph$_3$C][B(C$_6$F$_5$)$_4$], and 1 equivalent P(*p*-tolyl)$_3$ from **Table S1**, entry 2 in CDCl$_3$ at 298 K (30 min).

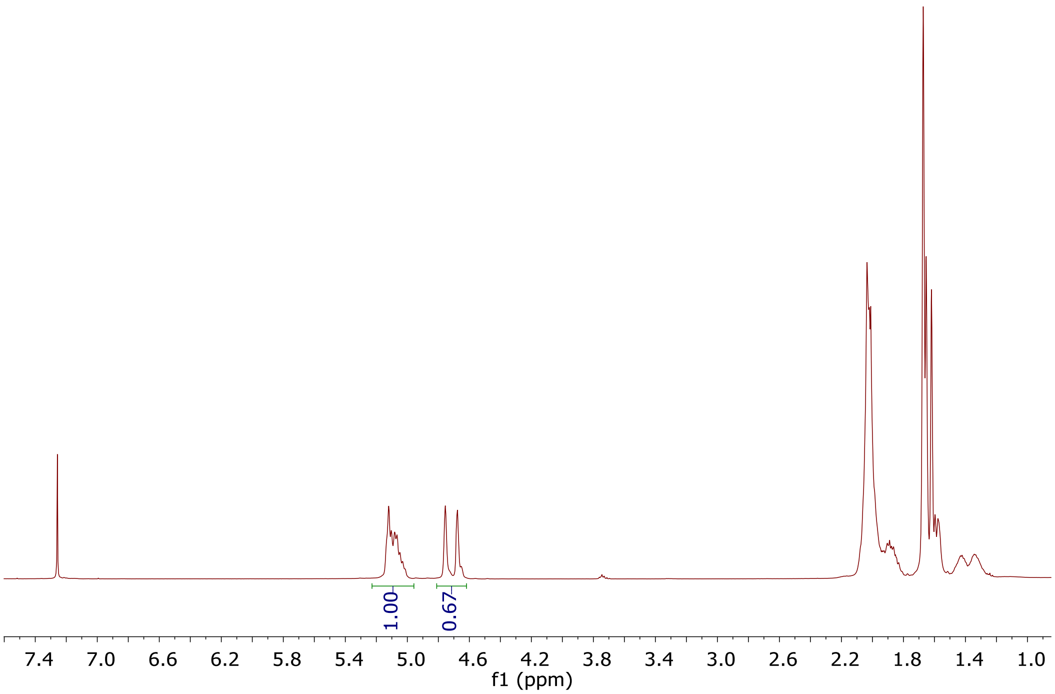

Fig. S10 ^{1}H NMR spectrum of PIP 500 equivalents generated by **Y(CH$_2$SiMe$_3$)$_3$(THF)$_2$**, 2 equivalents [Ph$_3$C][B(C$_6$F$_5$)$_4$], and 1 equivalent P(Ph-*p*-F)$_3$ from **Table S1**, entry 4 in CDCl$_3$ at 298 K (30 min).

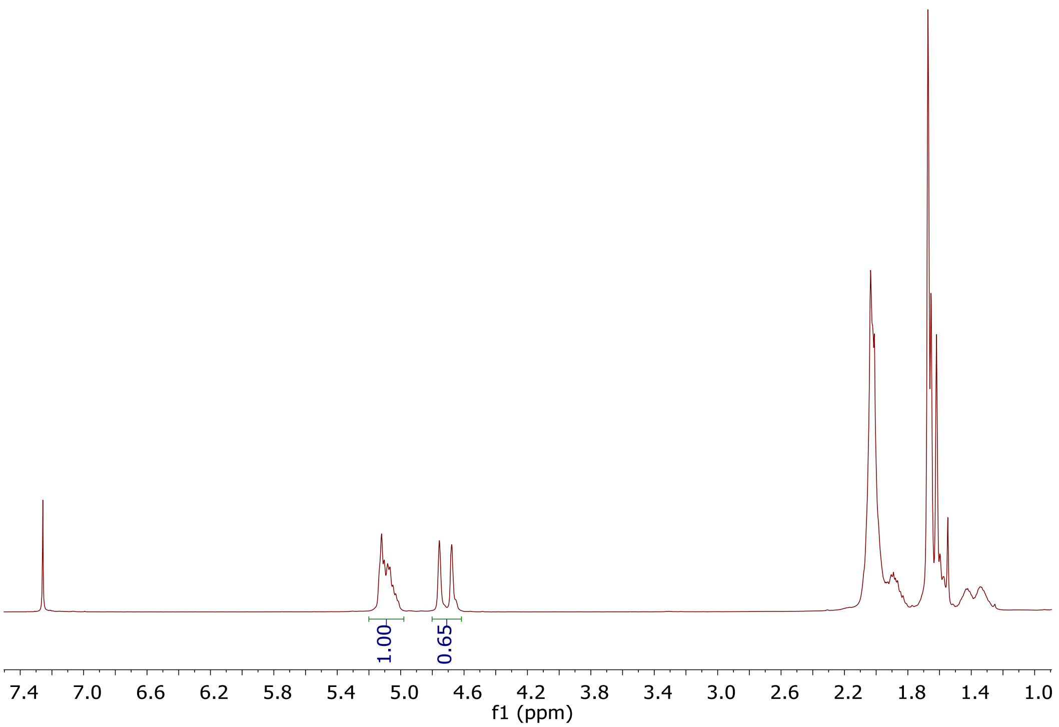

Fig. S11 ^{1}H NMR spectrum of PIP 500 equivalents generated by **$Y(CH_2SiMe_3)_3(THF)_2$** and 2 equivalents $[Ph_3C][B(C_6F_5)_4]$ from **Table S2**, entry 1 in $CDCl_3$ at 298 K (5 min).

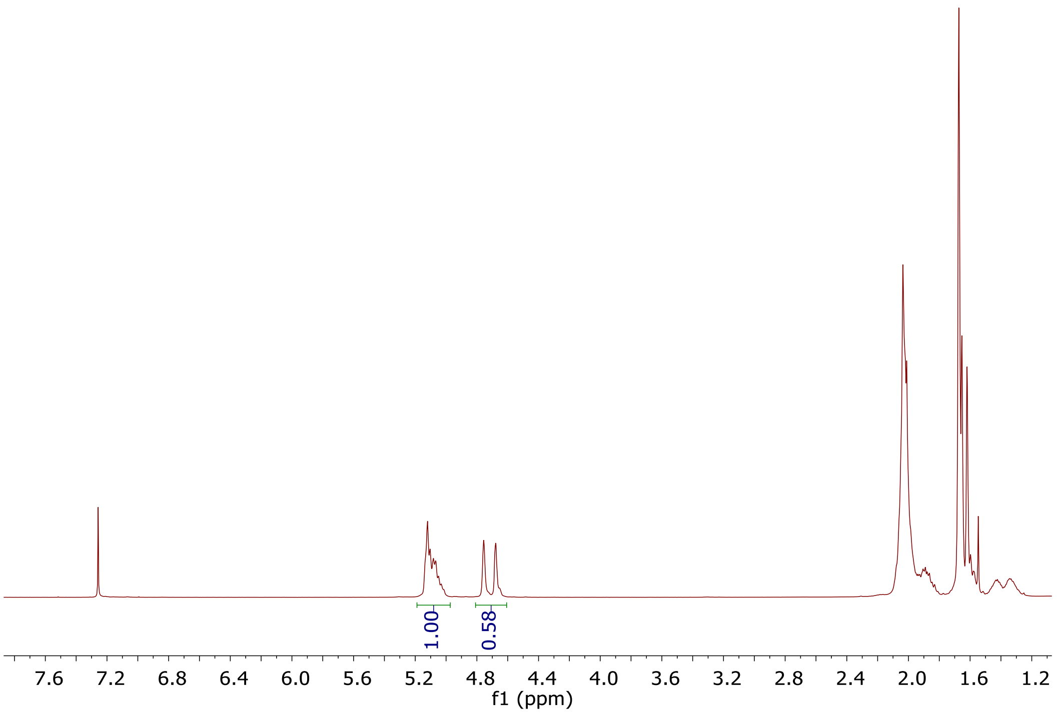

Fig. S12 ^{1}H NMR spectrum of PIP 500 equivalents generated by **$Y(CH_2SiMe_3)_3(THF)_2$** and 2 equivalents $[Ph_3C][B(C_6F_5)_4]$ from **Table S2**, entry 2 in $CDCl_3$ at 298 K (12 min).

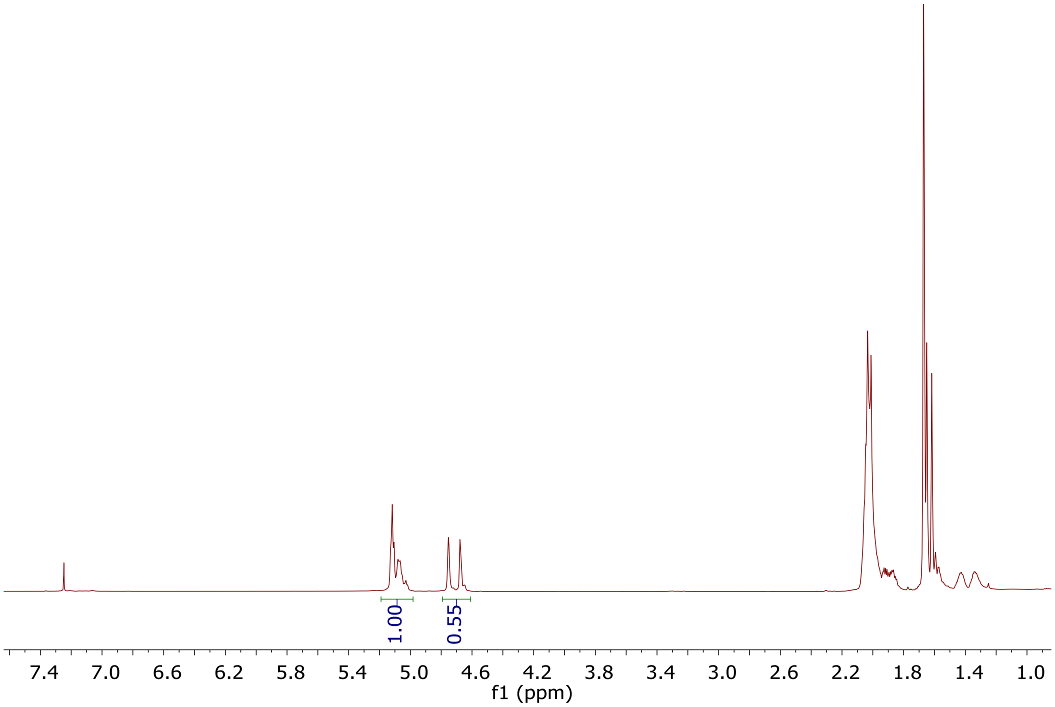

Fig. S13 ^{1}H NMR spectrum of PIP 500 equivalents generated by **$Y(CH_2SiMe_3)_3(THF)_2$** and 2 equivalents $[Ph_3C][B(C_6F_5)_4]$ from **Table S2**, entry 3 in $CDCl_3$ at 298 K (18 min).

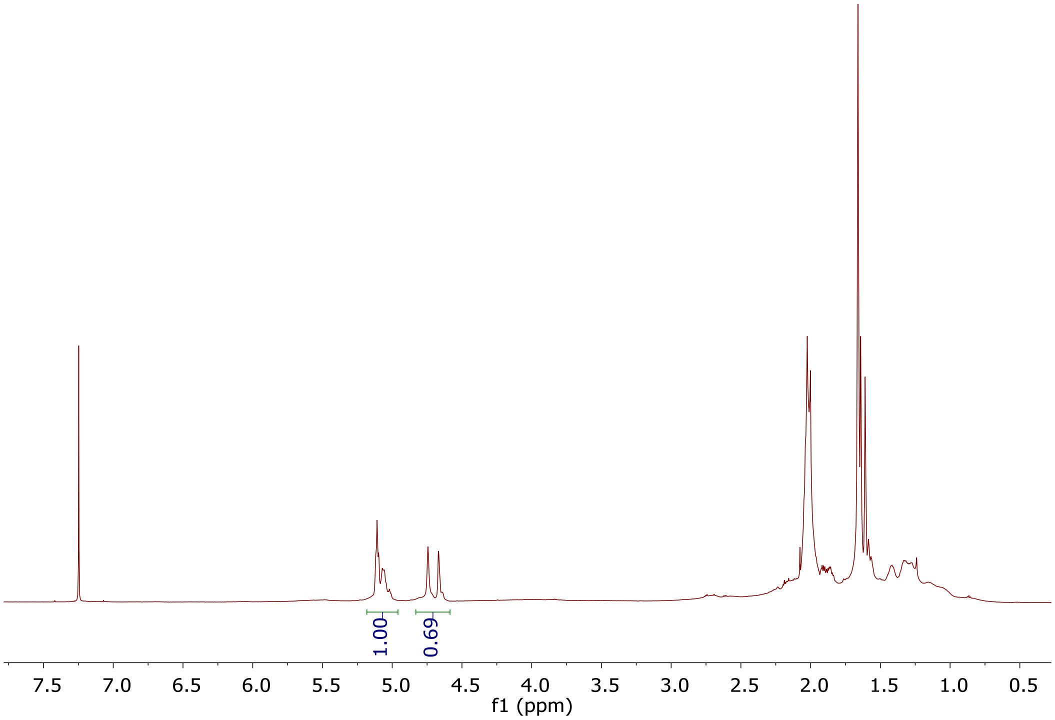

Fig. S14 ^{1}H NMR spectrum of PIP 500 equivalents generated by **$Y(CH_2SiMe_3)_3(THF)_2$** and 2 equivalents $[Ph_3C][B(C_6F_5)_4]$ from **Table S2**, entry 4 in $CDCl_3$ at 298 K (24 min).

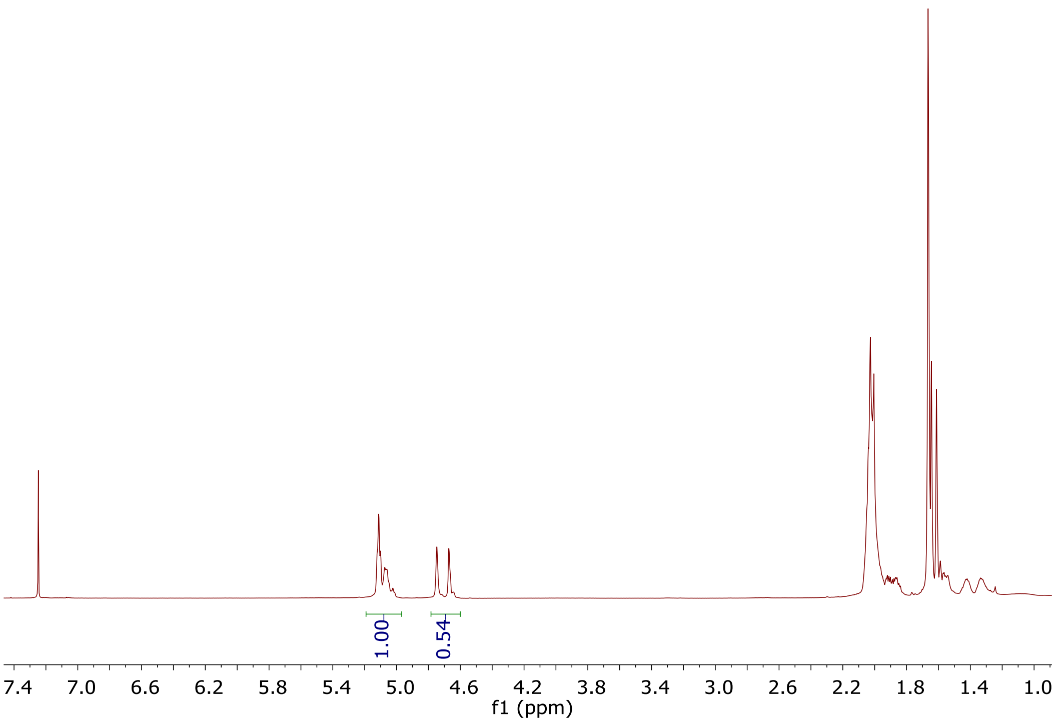

Fig. S15 ^{1}H NMR spectrum of PIP 500 equivalents generated by **$Y(CH_2SiMe_3)_3(THF)_2$** and 2 equivalents $[Ph_3C][B(C_6F_5)_4]$ from **Table S2**, entry 5 in $CDCl_3$ at 298 K (30 min).

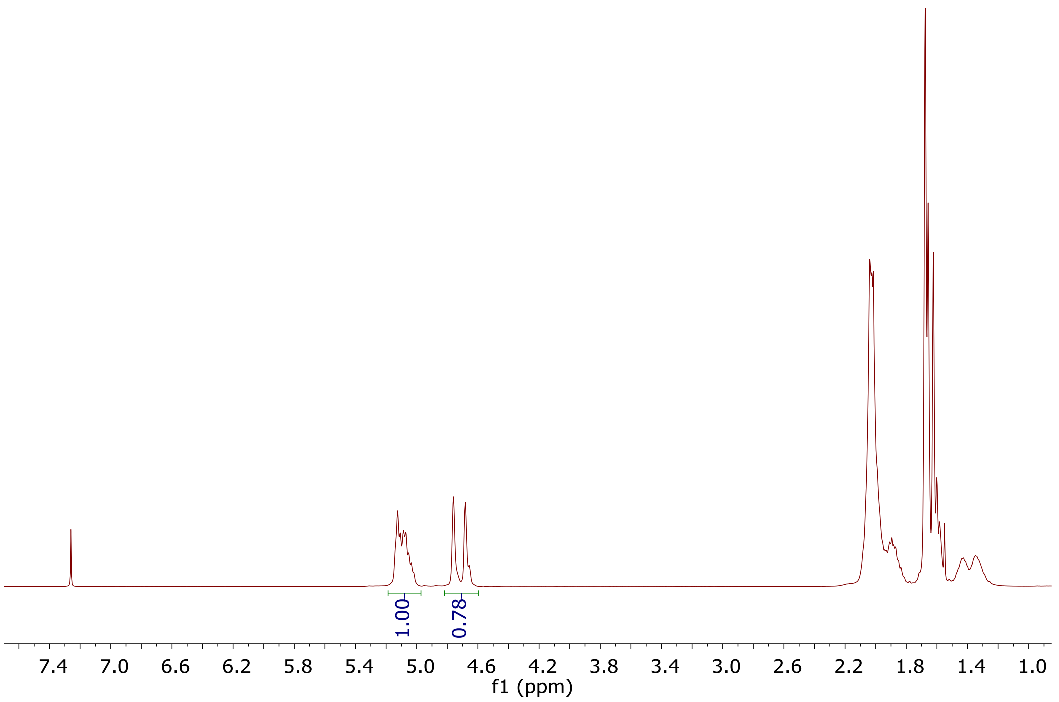

Fig. S16 ^{1}H NMR spectrum of PIP 500 equivalents generated by **$Y(CH_2SiMe_3)_3(THF)_2$**, 2 equivalents $[Ph_3C][B(C_6F_5)_4]$, and 1 equivalent PPh_3 from **Table S3**, entry 1 in $CDCl_3$ at 298 K (10 min).

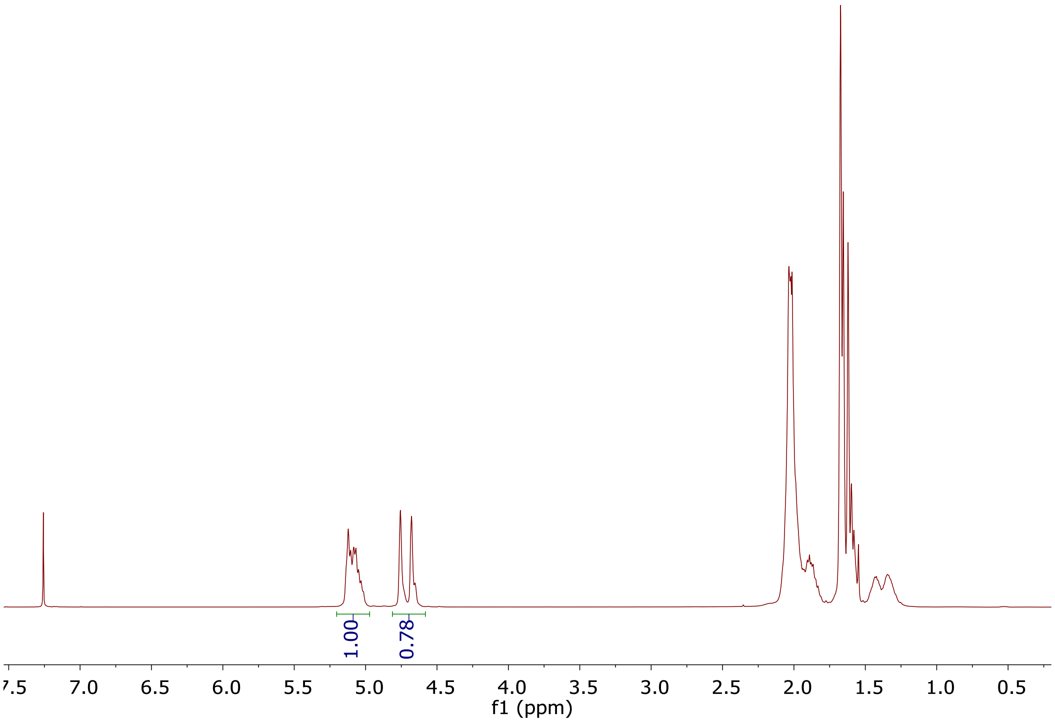

Fig. S17 ^{1}H NMR spectrum of PIP 500 equivalents generated by **Y(CH$_2$SiMe$_3$)$_3$(THF)$_2$**, 2 equivalents [Ph$_3$C][B(C$_6$F$_5$)$_4$], and 1 equivalent PPh$_3$ from **Table S3**, entry 2 in CDCl$_3$ at 298 K (21 min).

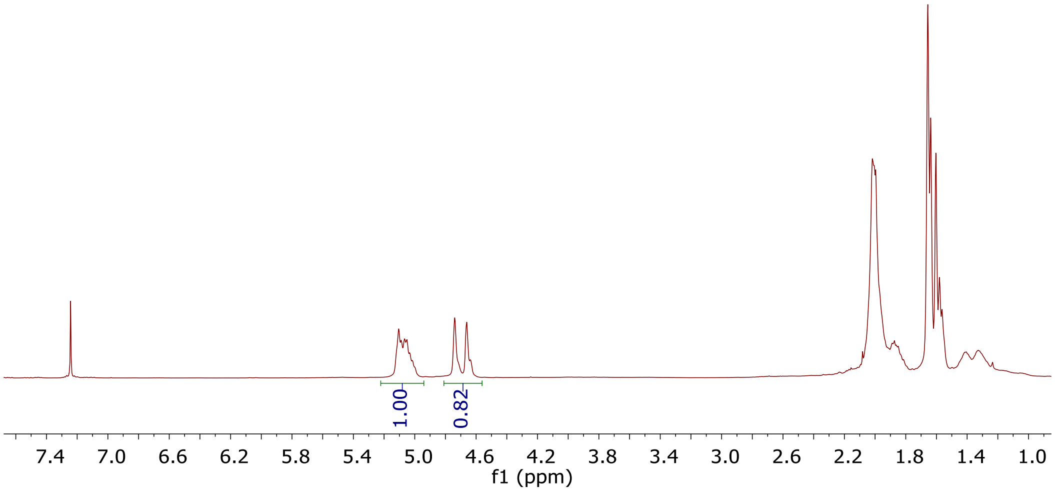

Fig. S18 ^{1}H NMR spectrum of PIP 500 equivalents generated by **Y(CH$_2$SiMe$_3$)$_3$(THF)$_2$**, 2 equivalents [Ph$_3$C][B(C$_6$F$_5$)$_4$], and 1 equivalent PPh$_3$ from **Table S3**, entry 3 in CDCl$_3$ at 298 K (31 min).

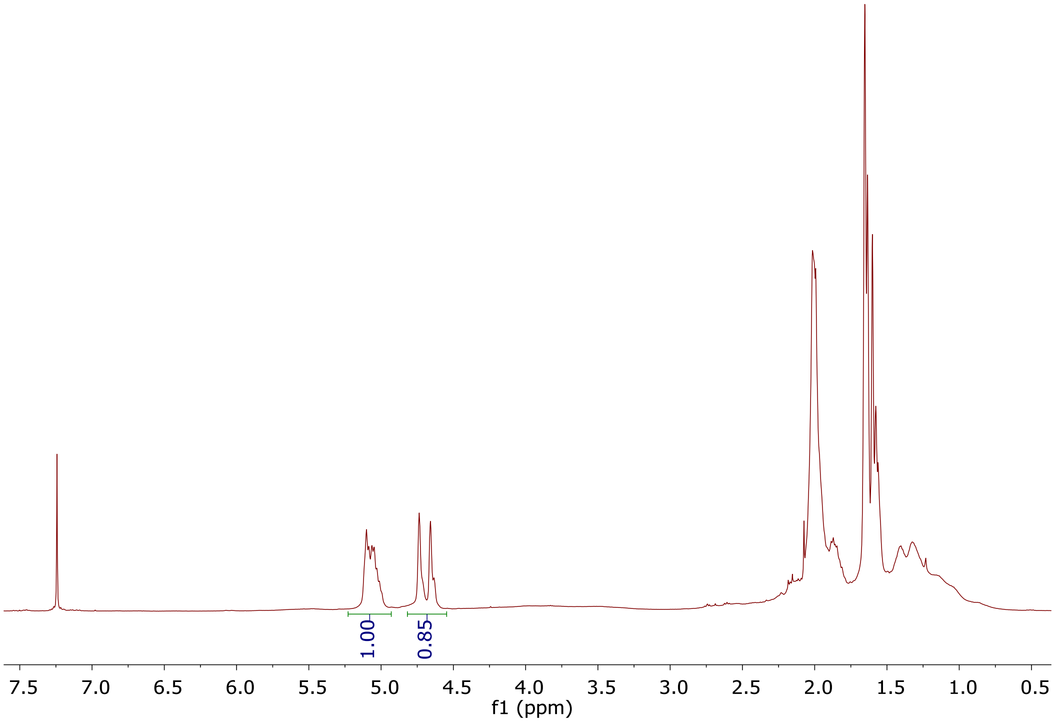

Fig. S19 ^{1}H NMR spectrum of PIP 500 equivalents generated by **$Y(CH_2SiMe_3)_3(THF)_2$**, 2 equivalents $[Ph_3C][B(C_6F_5)_4]$, and 1 equivalent PPh_3 from **Table S3**, entry 4 in $CDCl_3$ at 298 K (41 min).

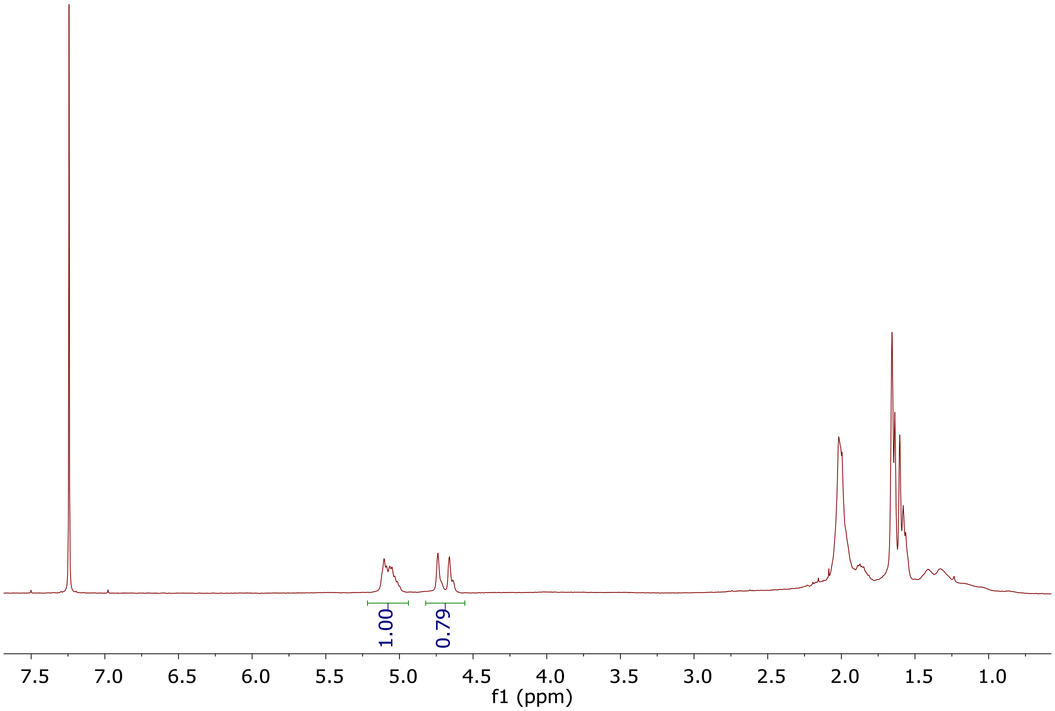

Fig. S20 ^{1}H NMR spectrum of PIP 500 equivalents generated by **$Y(CH_2SiMe_3)_3(THF)_2$**, 2 equivalents $[Ph_3C][B(C_6F_5)_4]$, and 1 equivalent PPh_3 from **Table S3**, entry 5 in $CDCl_3$ at 298 K (51 min).

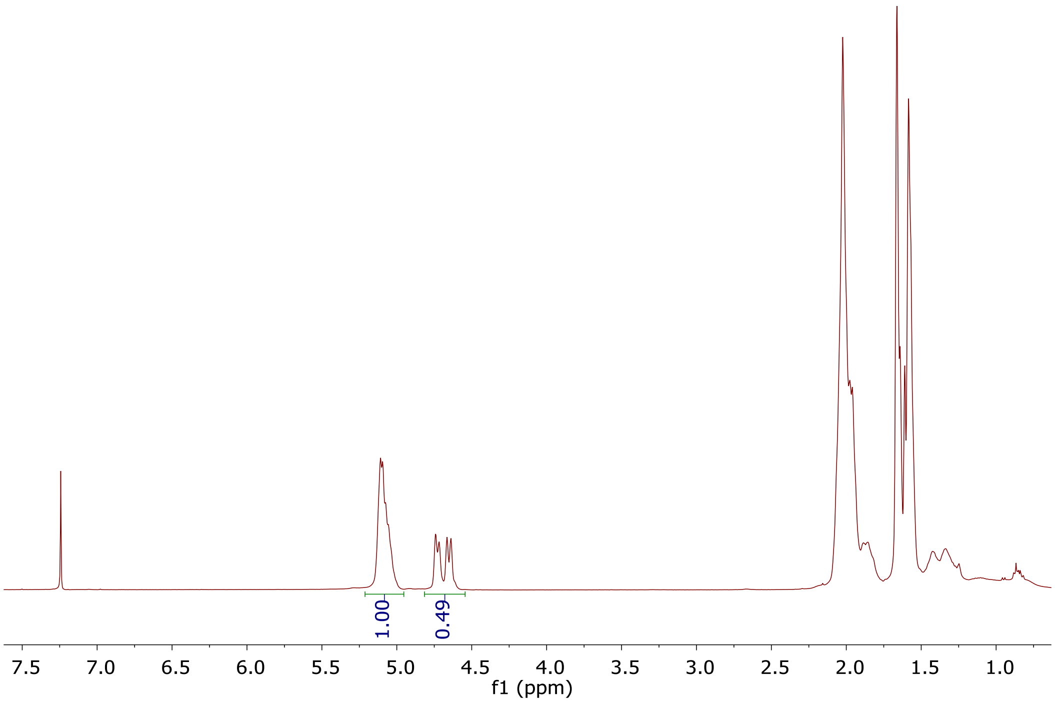

Fig. S21 ^{1}H NMR spectrum of PIP 500 equivalents generated by **Sm(CH_2SiMe_3)$_3$(THF)$_3$** and 1 equivalent [Ph_3C][B(C_6F_5)$_4$] from **Table S4**, entry 1 in $CDCl_3$ at 298 K (7 h).

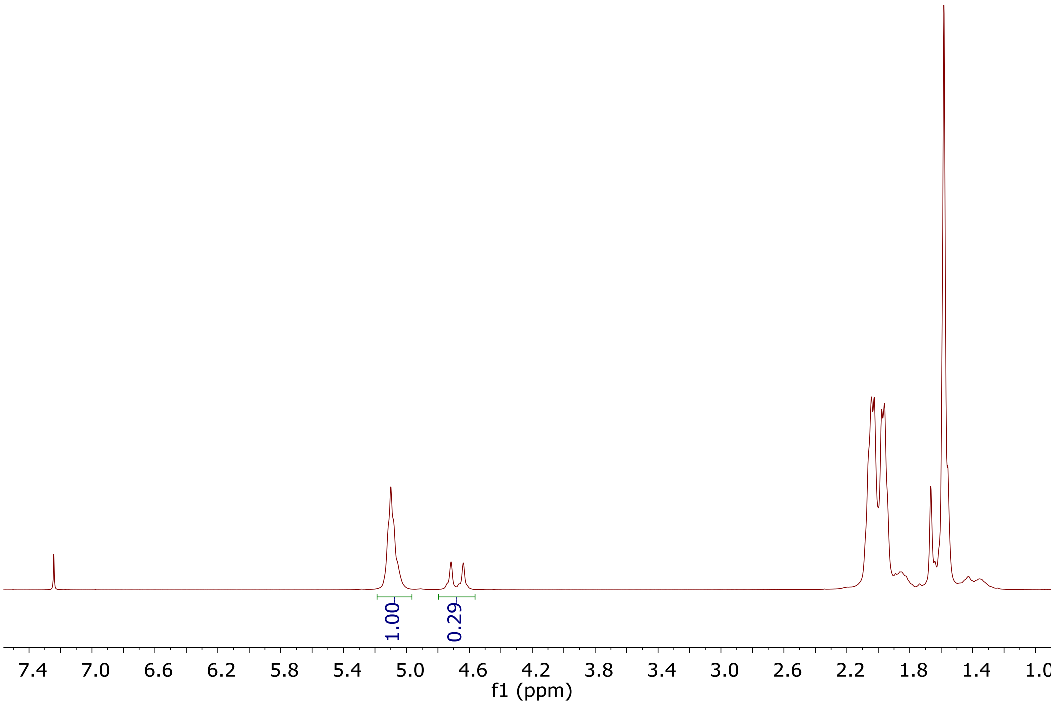

Fig. S22 ^{1}H NMR spectrum of PIP 500 equivalents generated by **Gd(CH_2SiMe_3)$_3$(THF)$_2$** and 1 equivalent [Ph_3C][B(C_6F_5)$_4$] from **Table S4**, entry 2 in $CDCl_3$ at 298 K (7 h).

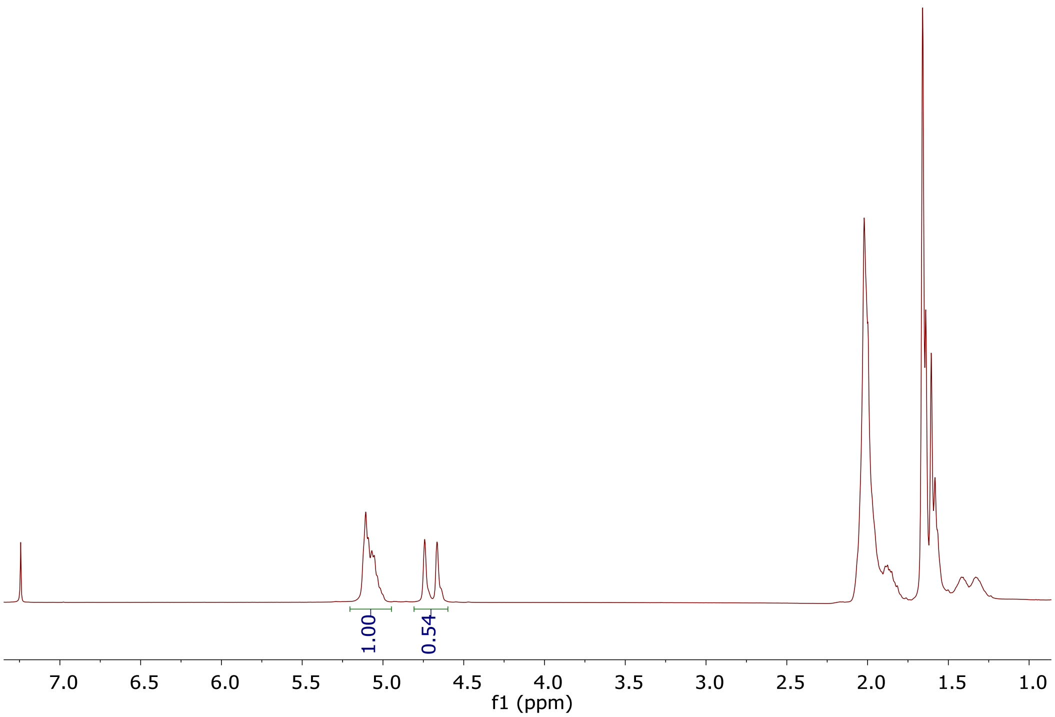

Fig. S23 ^{1}H NMR spectrum of PIP 500 equivalents generated by **Gd(CH$_2$SiMe$_3$)$_3$(THF)$_2$** and 2 equivalents [Ph$_3$C][B(C$_6$F$_5$)$_4$] from **Table S4**, entry 3 in CDCl$_3$ at 298 K (7 h).

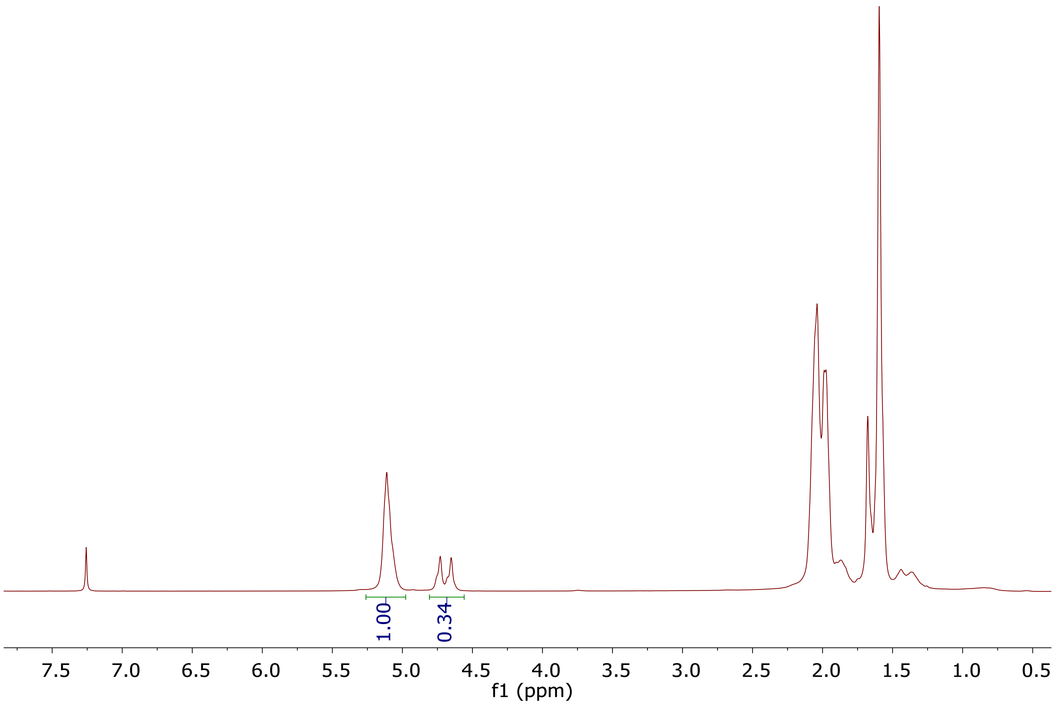

Fig. S24 ^{1}H NMR spectrum of PIP 500 equivalents generated by **Tm(CH$_2$SiMe$_3$)$_3$(THF)$_2$** and 1 equivalent [Ph$_3$C][B(C$_6$F$_5$)$_4$] from **Table S4**, entry 4 in CDCl$_3$ at 298 K (7 h).

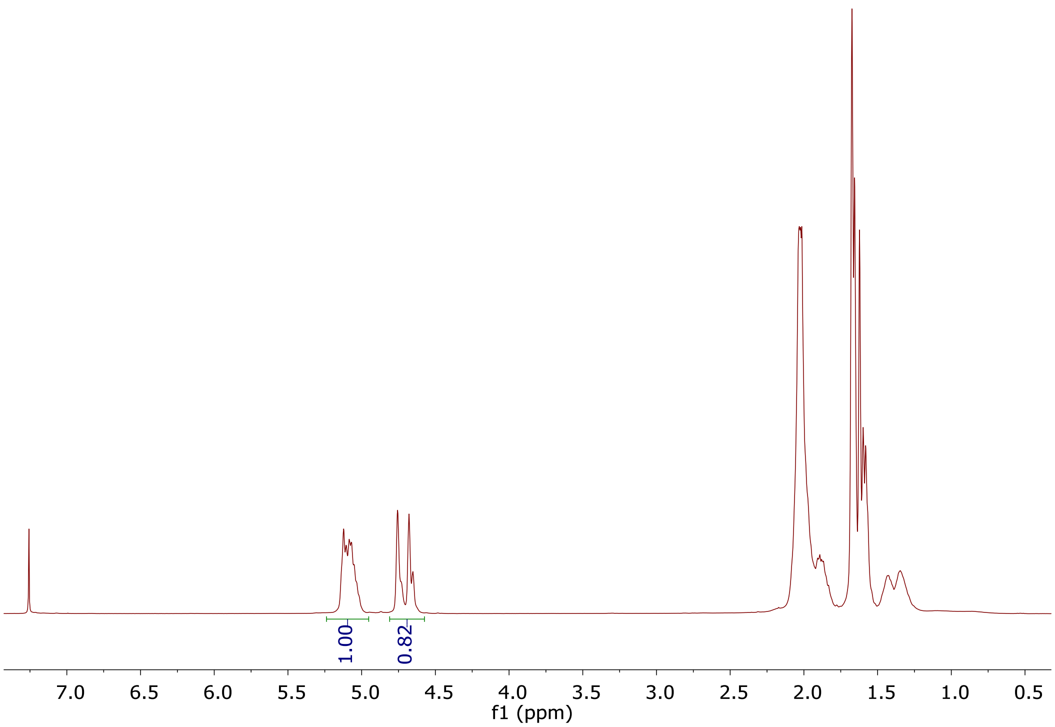

Fig. S25 ^{1}H NMR spectrum of PIP 500 equivalents generated by **$Tm(CH_2SiMe_3)_3(THF)_2$** and 2 equivalents $[Ph_3C][B(C_6F_5)_4]$ from **Table S4**, entry 5 in $CDCl_3$ at 298 K (7 h).

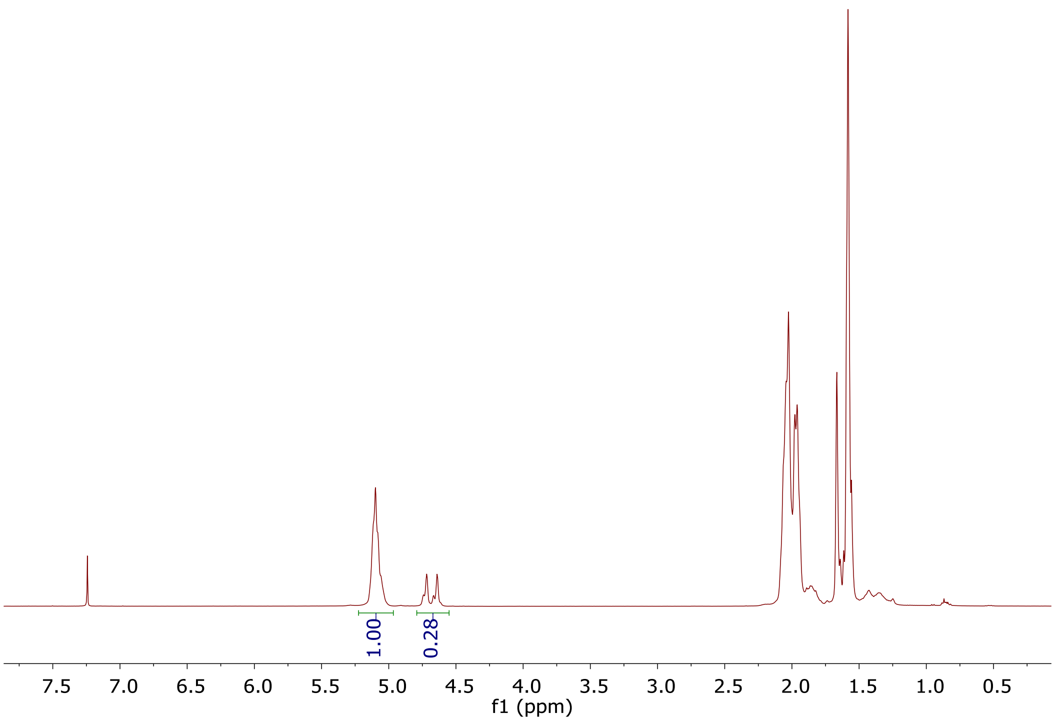

Fig. S26 ^{1}H NMR spectrum of PIP 500 equivalents generated by **$Sm(CH_2SiMe_3)_3(THF)_3$**, 1 equivalent $[Ph_3C][B(C_6F_5)_4]$, and 1 equivalent PPh_3 from **Table S4**, entry 6 in $CDCl_3$ at 298 K (7 h).

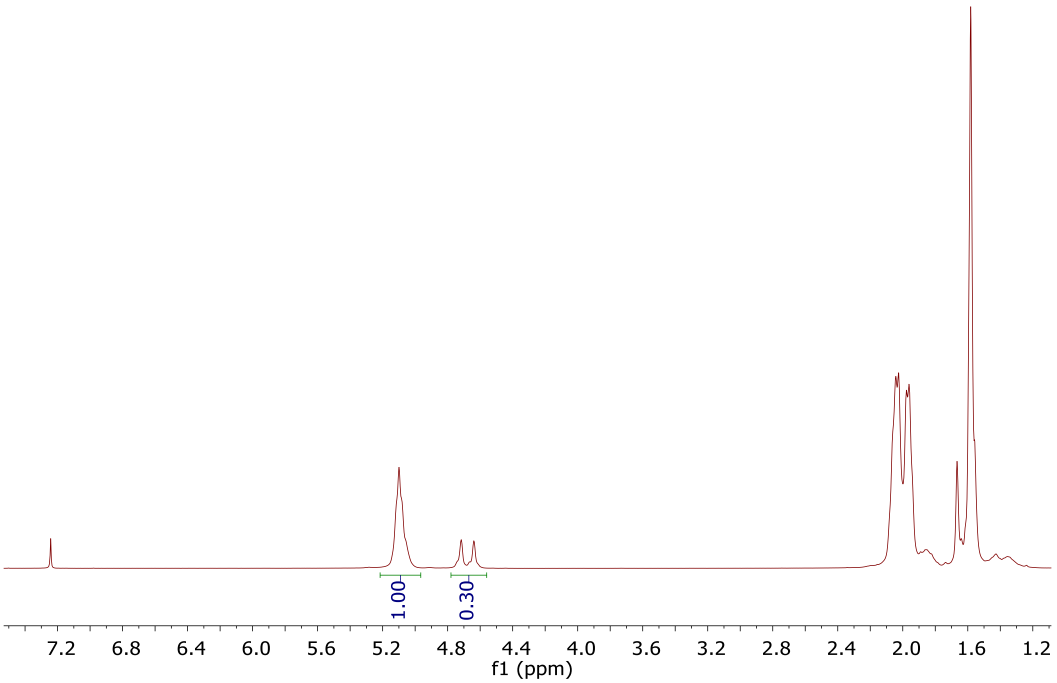

Fig. S27 ^{1}H NMR spectrum of PIP 500 equivalents generated by **$Gd(CH_2SiMe_3)_3(THF)_2$**, 1 equivalent $[Ph_3C][B(C_6F_5)_4]$, and 1 equivalent PPh_3 from **Table S4**, entry 7 in $CDCl_3$ at 298 K (7 h).

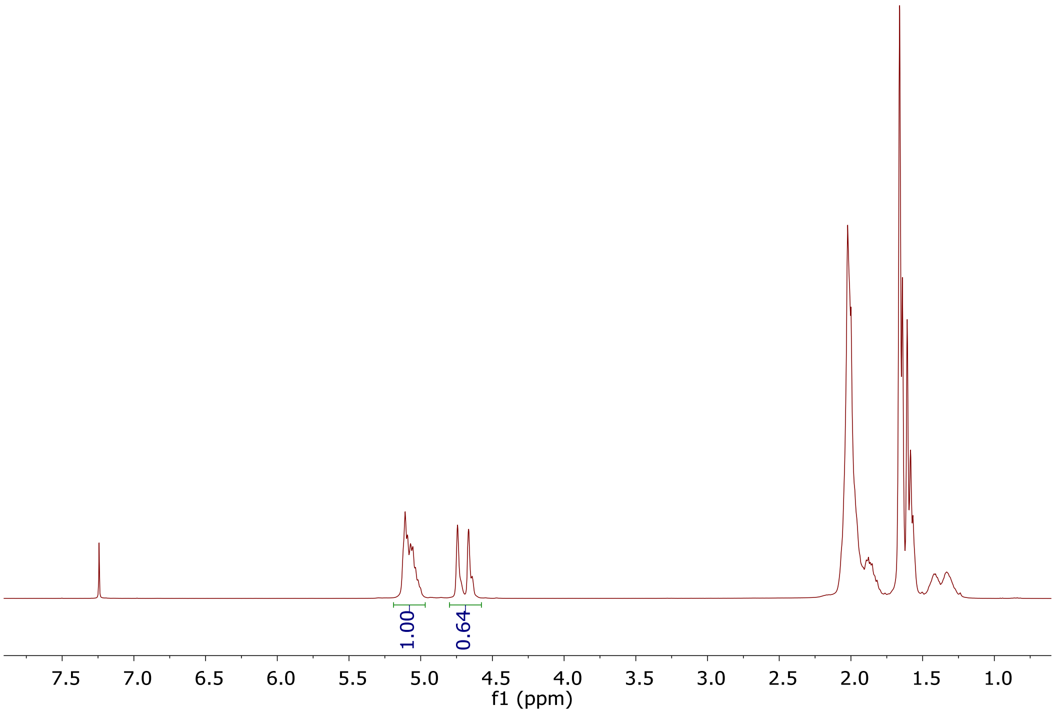

Fig. S28 ^{1}H NMR spectrum of PIP 500 equivalents generated by **$Gd(CH_2SiMe_3)_3(THF)_2$**, 2 equivalents $[Ph_3C][B(C_6F_5)_4]$, and 1 equivalent PPh_3 from **Table S4**, entry 8 in $CDCl_3$ at 298 K (7 h).

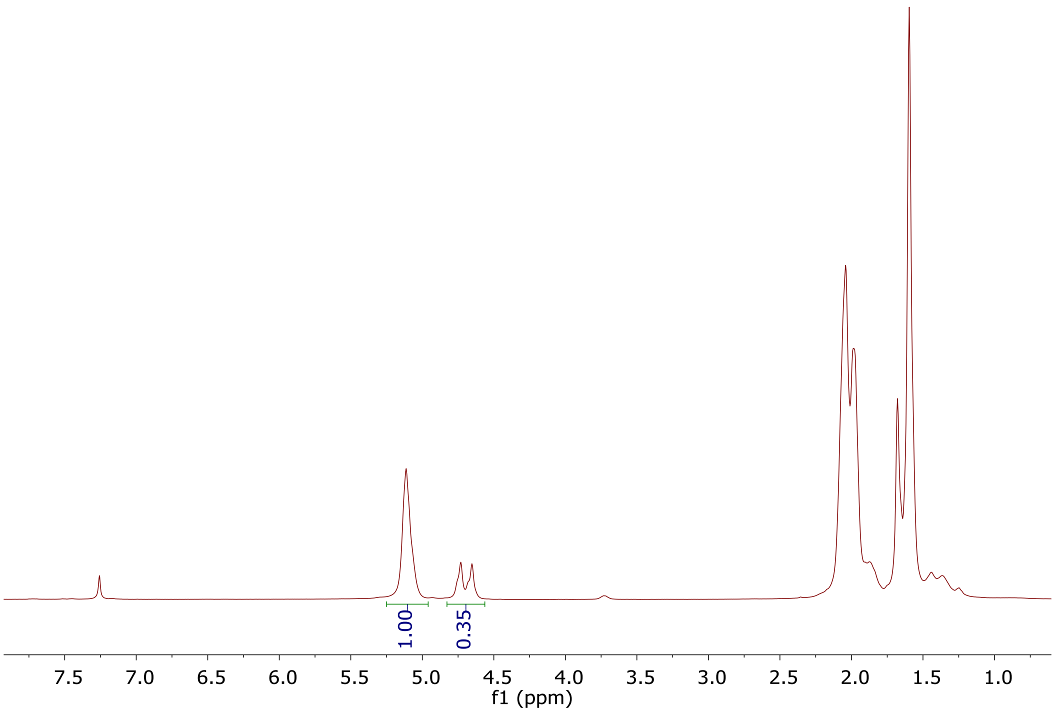

Fig. S29 ^{1}H NMR spectrum of PIP 500 equivalents generated by **Tm(CH$_2$SiMe$_3$)$_3$(THF)$_2$**, 1 equivalent [Ph$_3$C][B(C$_6$F$_5$)$_4$], and 1 equivalent PPh$_3$ from **Table S4**, entry 9 in CDCl$_3$ at 298 K (7 h).

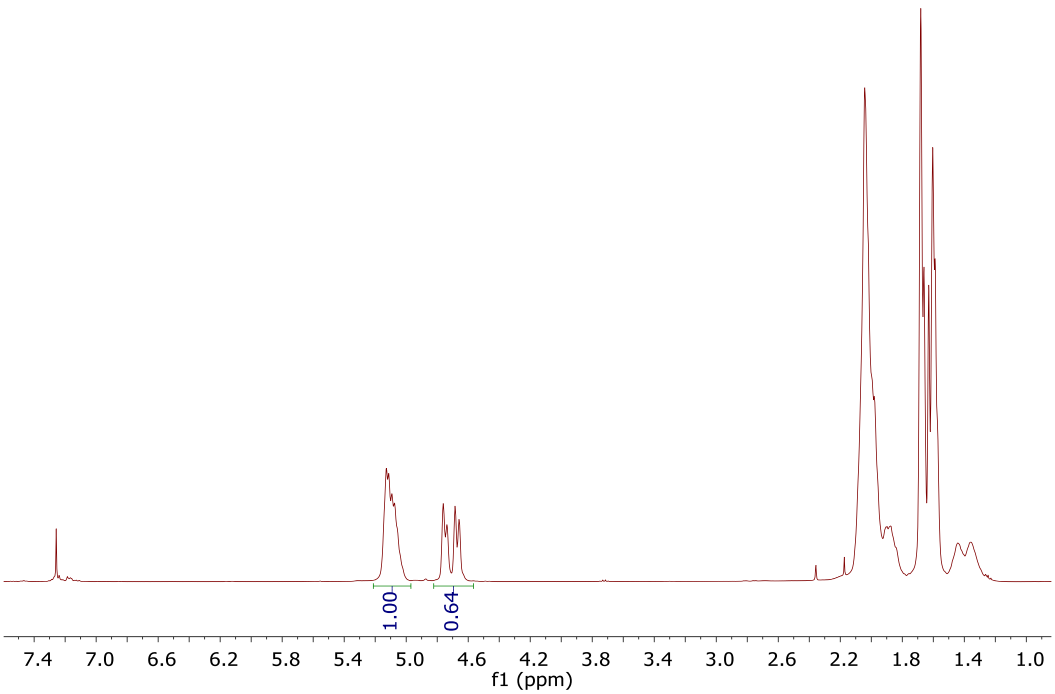

Fig. S30 ^{1}H NMR spectrum of PIP 500 equivalents generated by **Tm(CH$_2$SiMe$_3$)$_3$(THF)$_2$**, 2 equivalents [Ph$_3$C][B(C$_6$F$_5$)$_4$], and 1 equivalent PPh$_3$ from **Table S4**, entry 10 in CDCl$_3$ at 298 K (7 h).

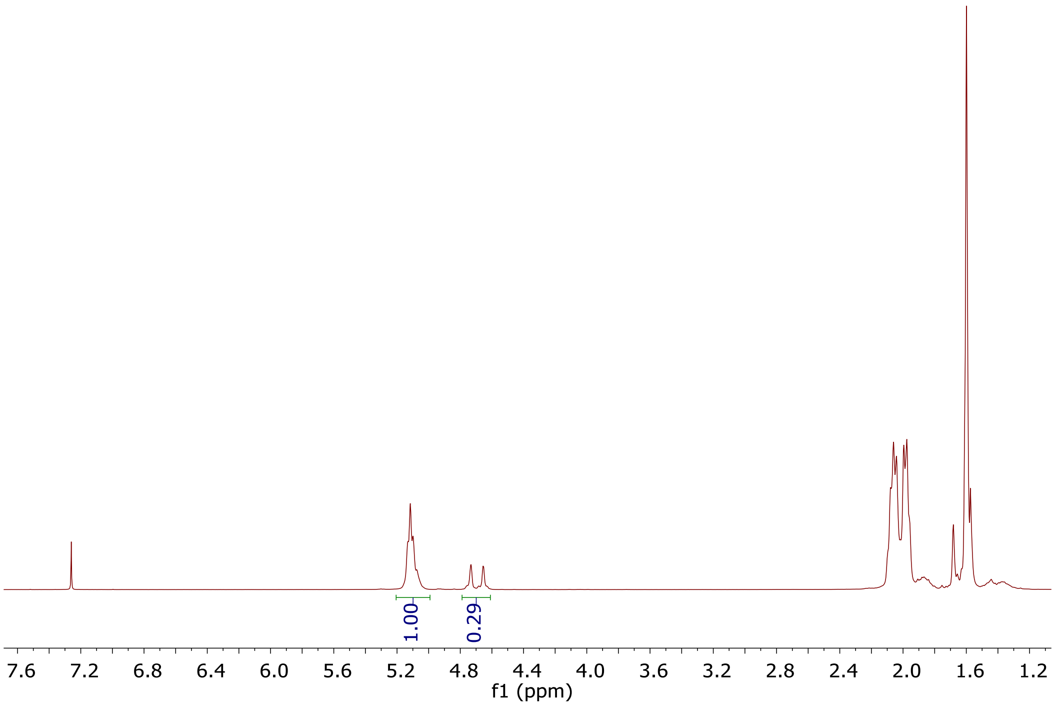

Fig. S31 ^{1}H NMR spectrum of PIP 500 equivalents generated by **$Y(CH_2SiMe_3)_3(THF)_2$** and 1 equivalent $[Ph_3C][B(C_6F_5)_4]$ from **Table 3**, entry 1 in $CDCl_3$ at 298 K (7 h).

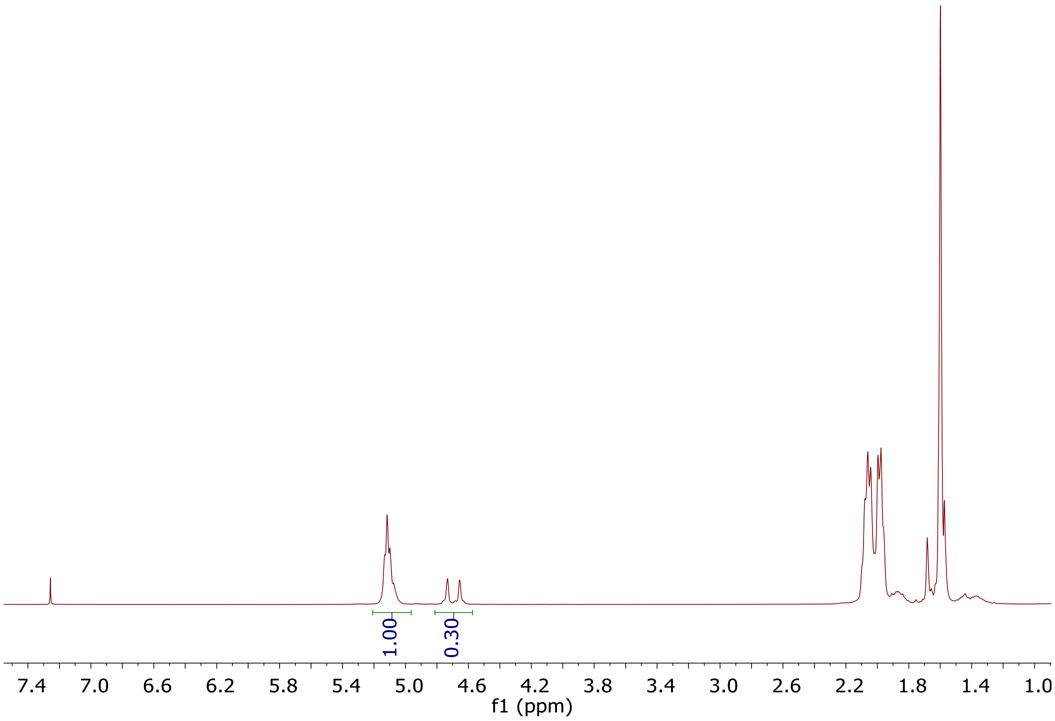

Fig. S32 ^{1}H NMR spectrum of PIP 500 equivalents generated by **$Y(CH_2SiMe_3)_3(THF)_2$**, 1 equivalent $[Ph_3C][B(C_6F_5)_4]$, and 1 equivalent PPh_3 from **Table 3**, entry 2 in $CDCl_3$ at 298 K (7 h).

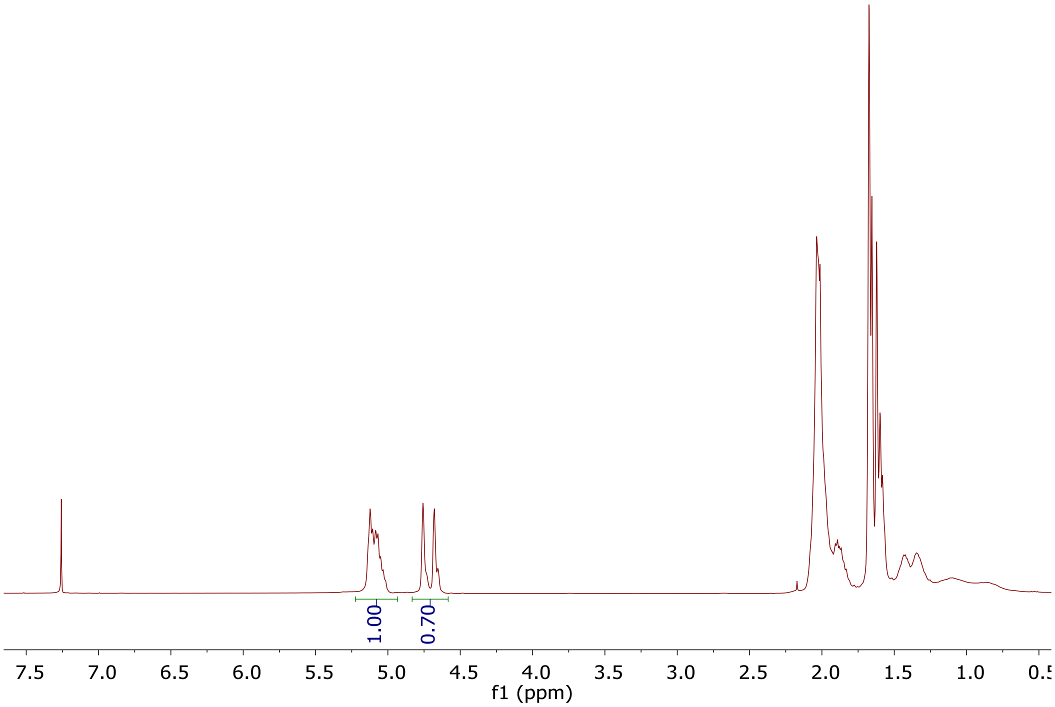

Fig. S33 ^1H NMR spectrum of PIP 500 equivalents generated by $\mathbf{Y(CH_2SiMe_3)_3(THF)_2}$ and 1.5 equivalents $[Ph_3C][B(C_6F_5)_4]$ from **Table 3**, entry 3 in $CDCl_3$ at 298 K (7 h).

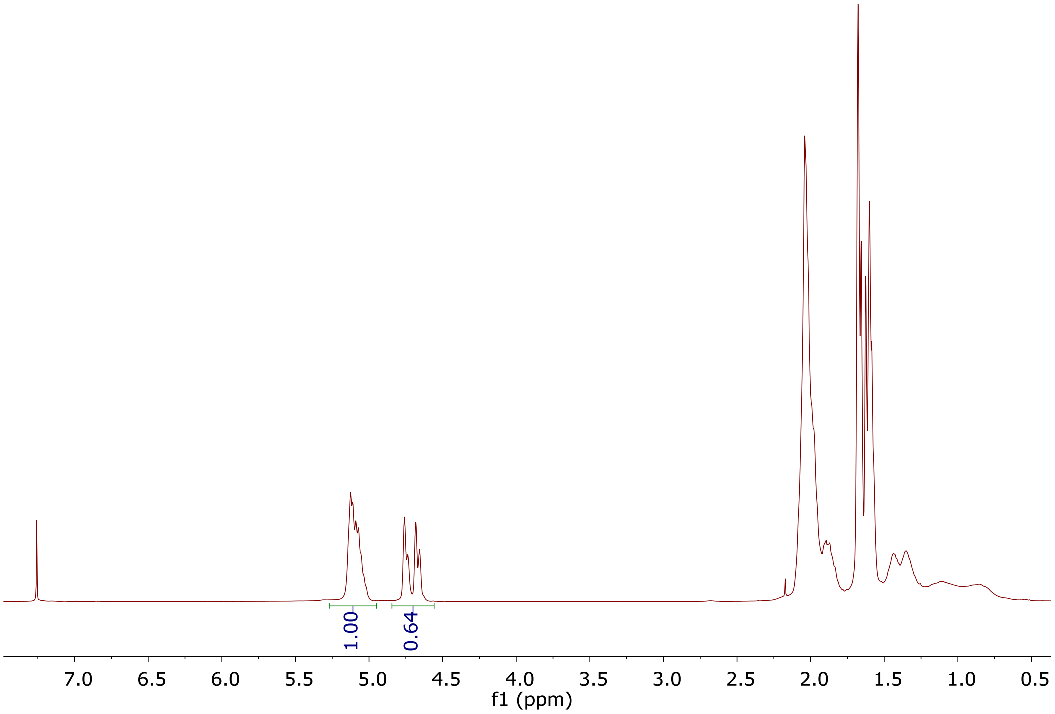

Fig. S34 ^1H NMR spectrum of PIP 500 equivalents generated by $\mathbf{Y(CH_2SiMe_3)_3(THF)_2}$, 1.5 equivalents $[Ph_3C][B(C_6F_5)_4]$, and 1 equivalent PPh_3 from **Table 3**, entry 4 in $CDCl_3$ at 298 K (7 h).

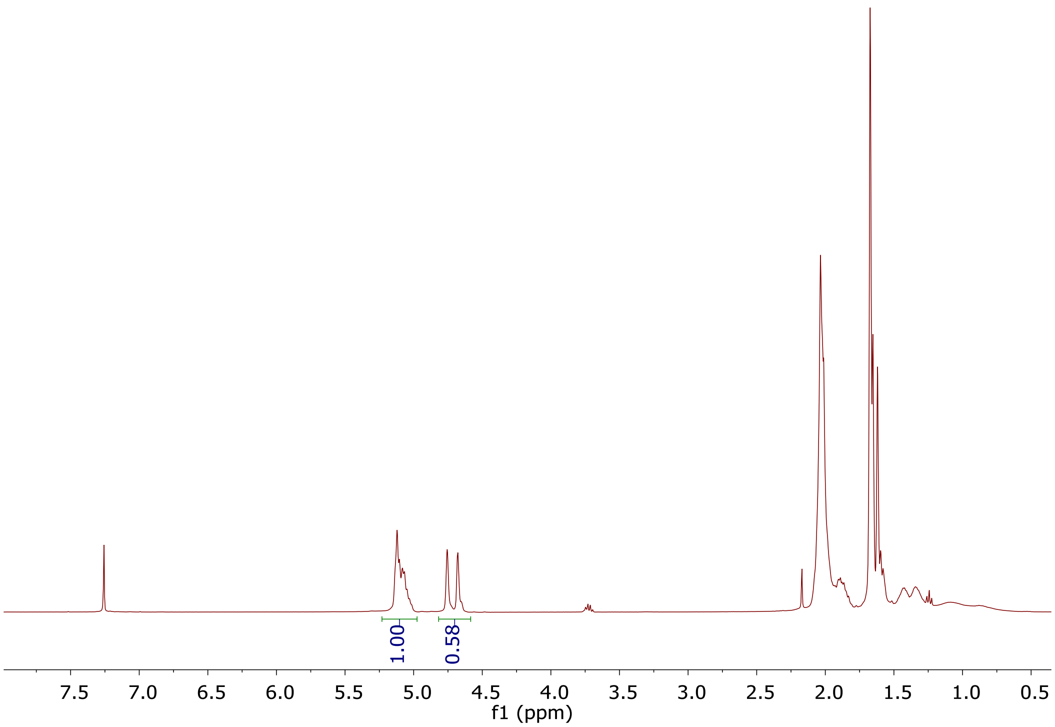

Fig. S35 ^{1}H NMR spectrum of PIP 500 equivalents generated by **$Y(CH_2SiMe_3)_3(THF)_2$** and 2 equivalents $[Ph_3C][B(C_6F_5)_4]$ from **Table 3**, entry 5 in $CDCl_3$ at 298 K (7 h).

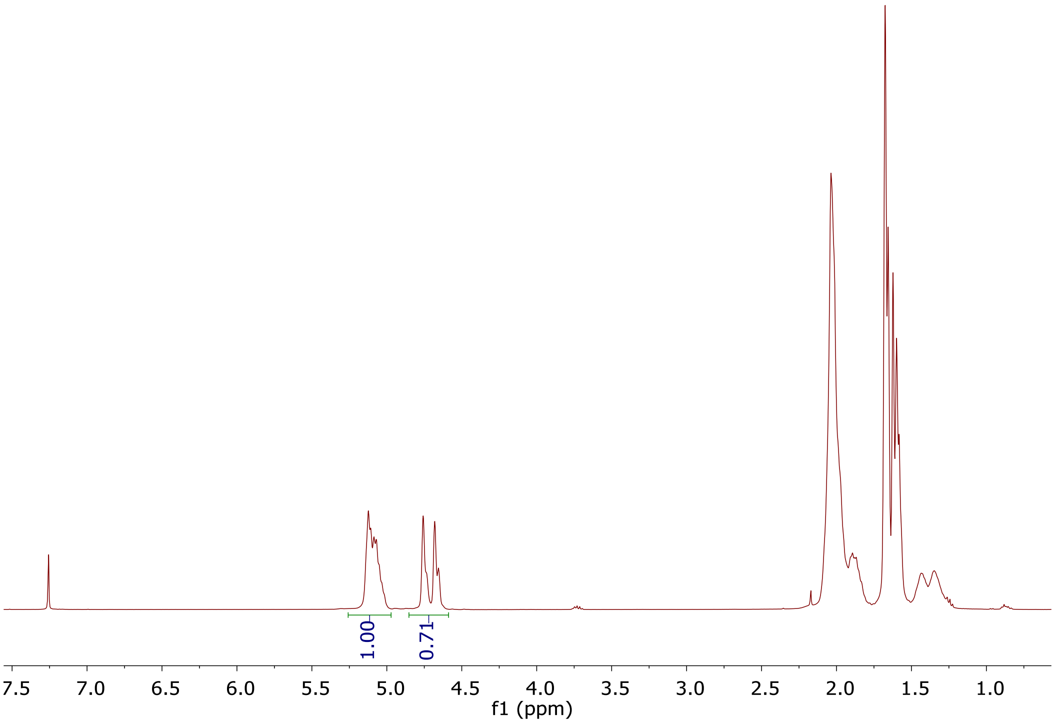

Fig. S36 ^{1}H NMR spectrum of PIP 500 equivalents generated by **$Y(CH_2SiMe_3)_3(THF)_2$**, 2 equivalents $[Ph_3C][B(C_6F_5)_4]$, and 1 equivalent PPh_3 from **Table 3**, entry 6 in $CDCl_3$ at 298 K (7 h).

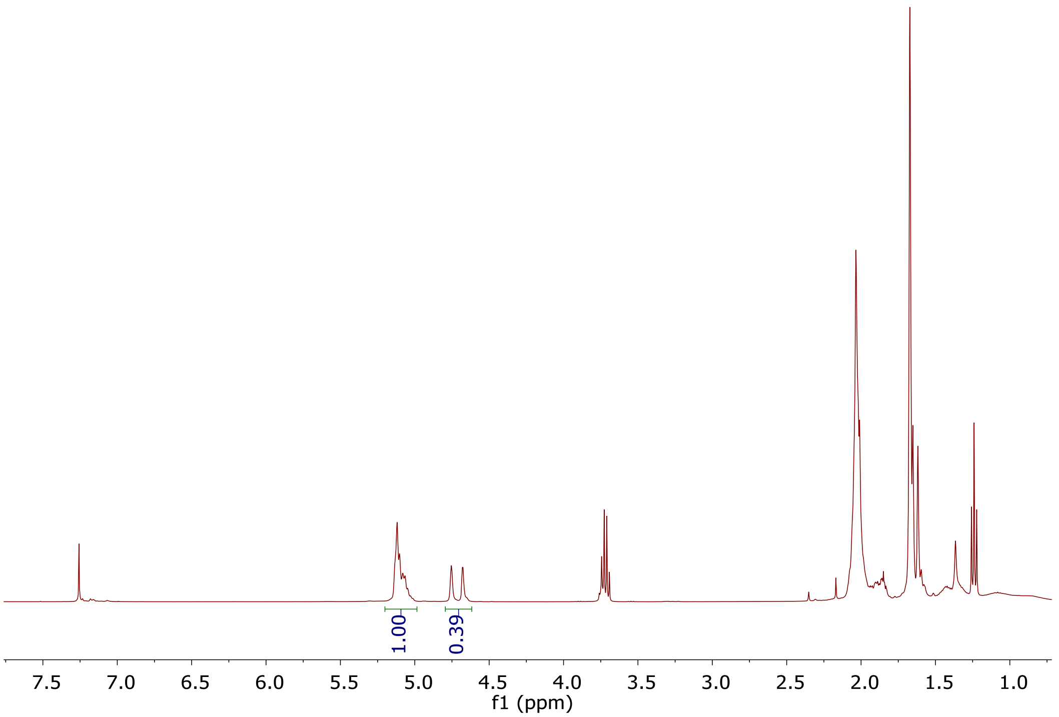

Fig. S37 ^{1}H NMR spectrum of PIP 500 equivalents generated by **$Y(CH_2SiMe_3)_3(THF)_2$** and 2.5 equivalents $[Ph_3C][B(C_6F_5)_4]$ from **Table 3**, entry 7 in $CDCl_3$ at 298 K (7 h).

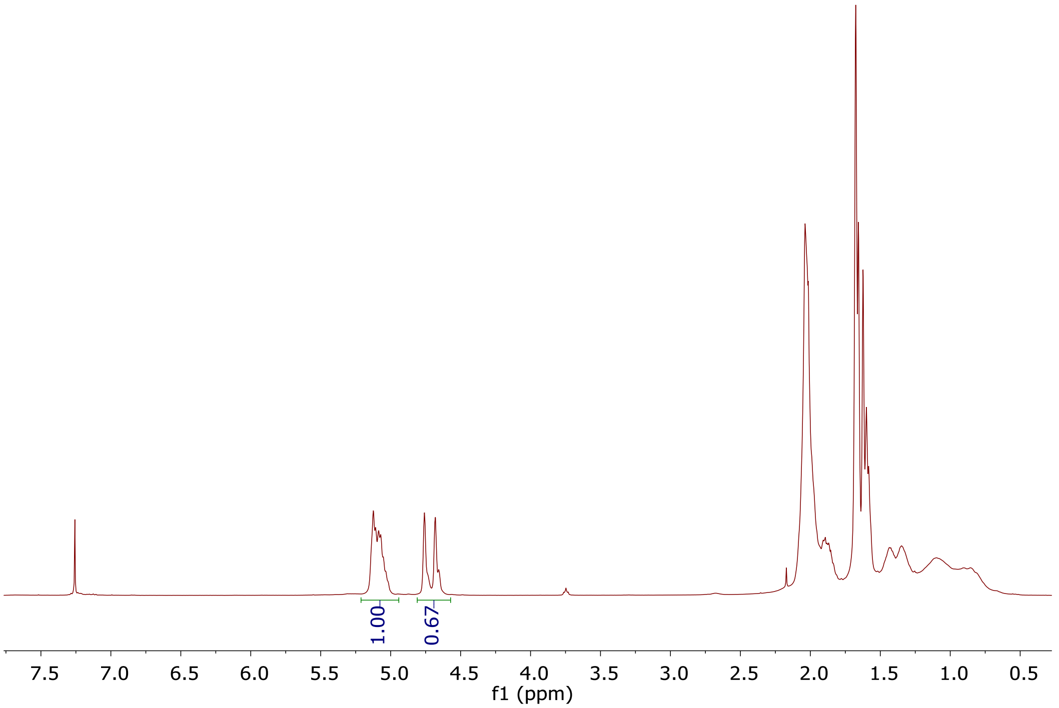

Fig. S38 ^{1}H NMR spectrum of PIP 500 equivalents generated by **$Y(CH_2SiMe_3)_3(THF)_2$**, 2.5 equivalents $[Ph_3C][B(C_6F_5)_4]$, and 1 equivalent PPh_3 from **Table 3**, entry 8 in $CDCl_3$ at 298 K (7 h).

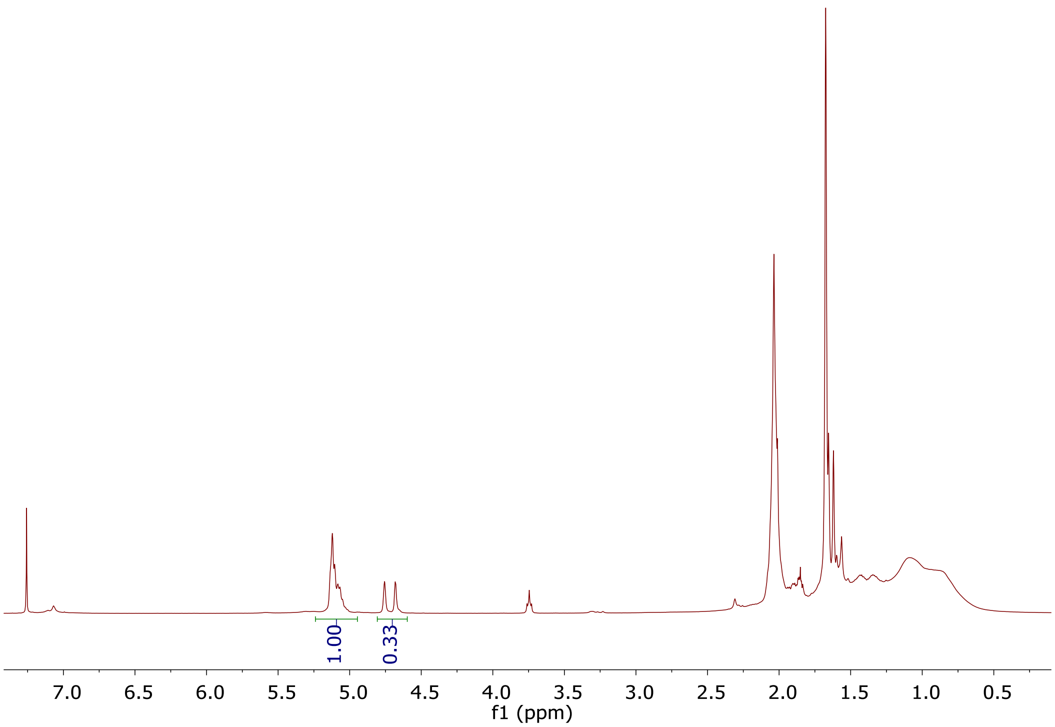

Fig. S39 ^{1}H NMR spectrum of PIP 500 equivalents generated by **Y(CH$_2$SiMe$_3$)$_3$(THF)$_2$** and 3 equivalents [Ph$_3$C][B(C$_6$F$_5$)$_4$] from **Table 3**, entry 9 in CDCl$_3$ at 298 K (7 h).

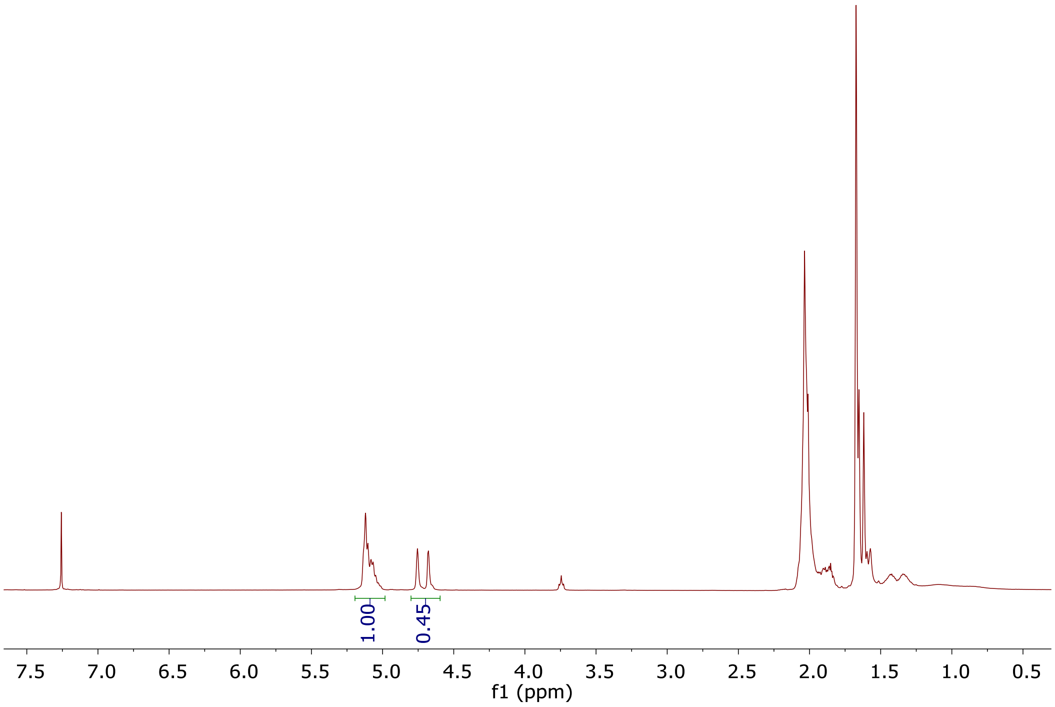

Fig. S40 ^{1}H NMR spectrum of PIP 500 equivalents generated by **Y(CH$_2$SiMe$_3$)$_3$(THF)$_2$**, 3 equivalents [Ph$_3$C][B(C$_6$F$_5$)$_4$], and 1 equivalent PPh$_3$ from **Table 3**, entry 10 in CDCl$_3$ at 298 K (7 h).

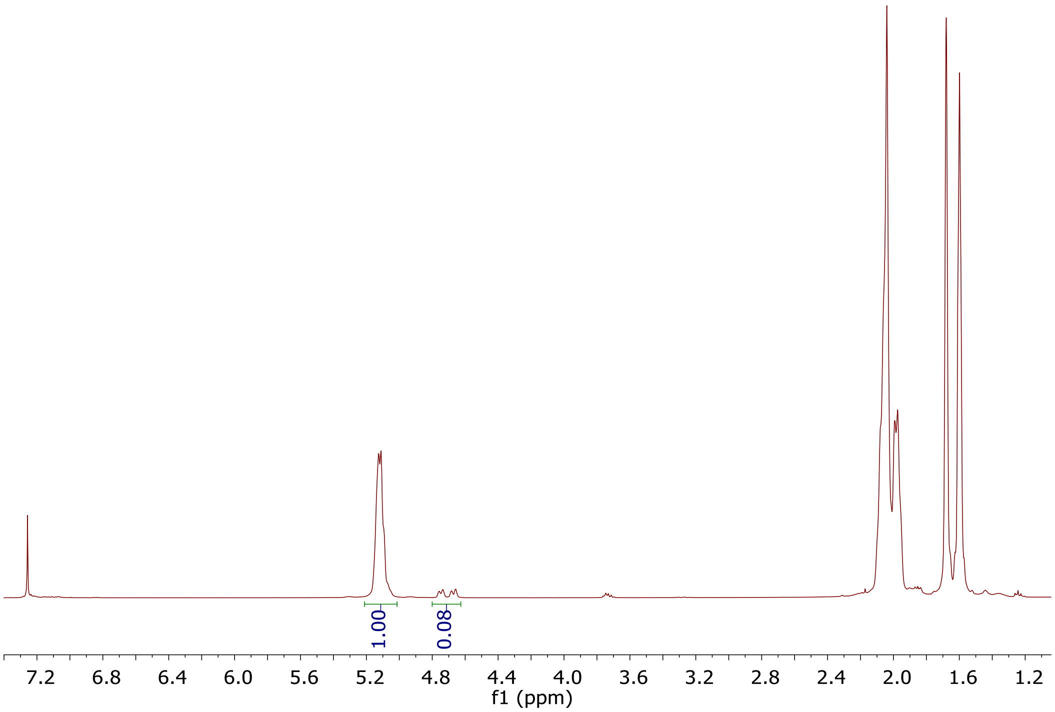

Fig. S41 ^{1}H NMR spectrum of PIP 500 equivalents generated by **$Y(CH_2SiMe_3)_3(THF)_2$**, 2 equivalents $[Ph_3C][B(C_6F_5)_4]$, and 5 equivalents $AlMe_3$ from **Table 4**, entry 1 in $CDCl_3$ at 298 K (30 min).

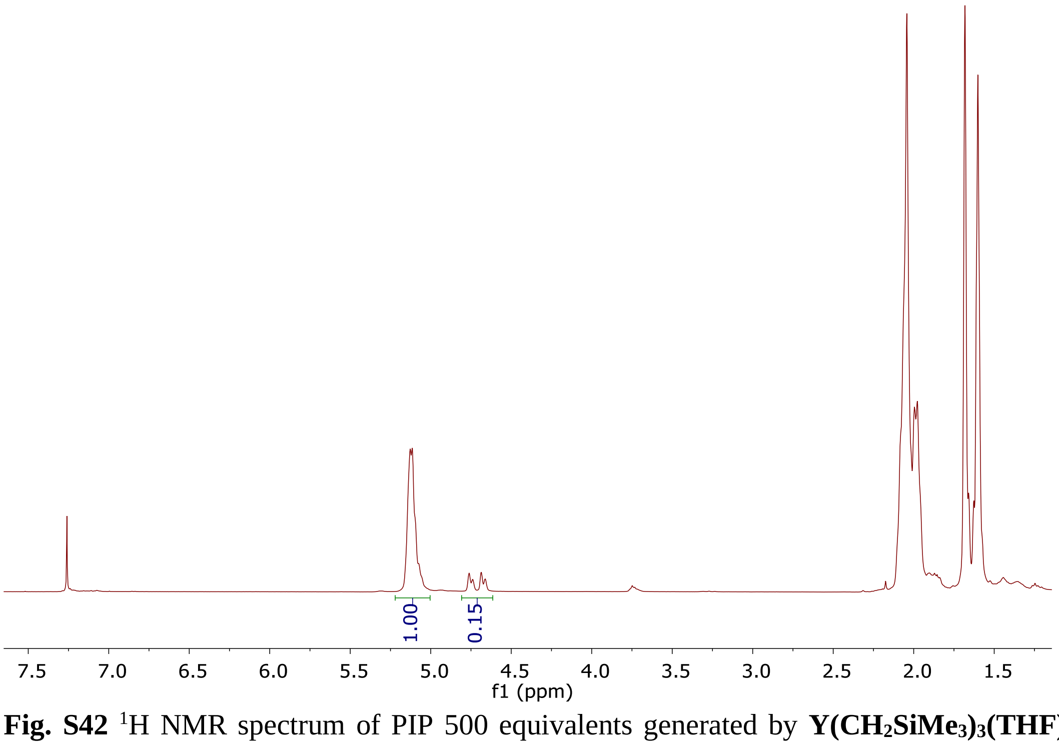

Fig. S42 ^{1}H NMR spectrum of PIP 500 equivalents generated by **$Y(CH_2SiMe_3)_3(THF)_2$**, 2 equivalents $[Ph_3C][B(C_6F_5)_4]$, and 10 equivalents $AlMe_3$ from **Table 4**, entry 2 in $CDCl_3$ at 298 K (30 min).

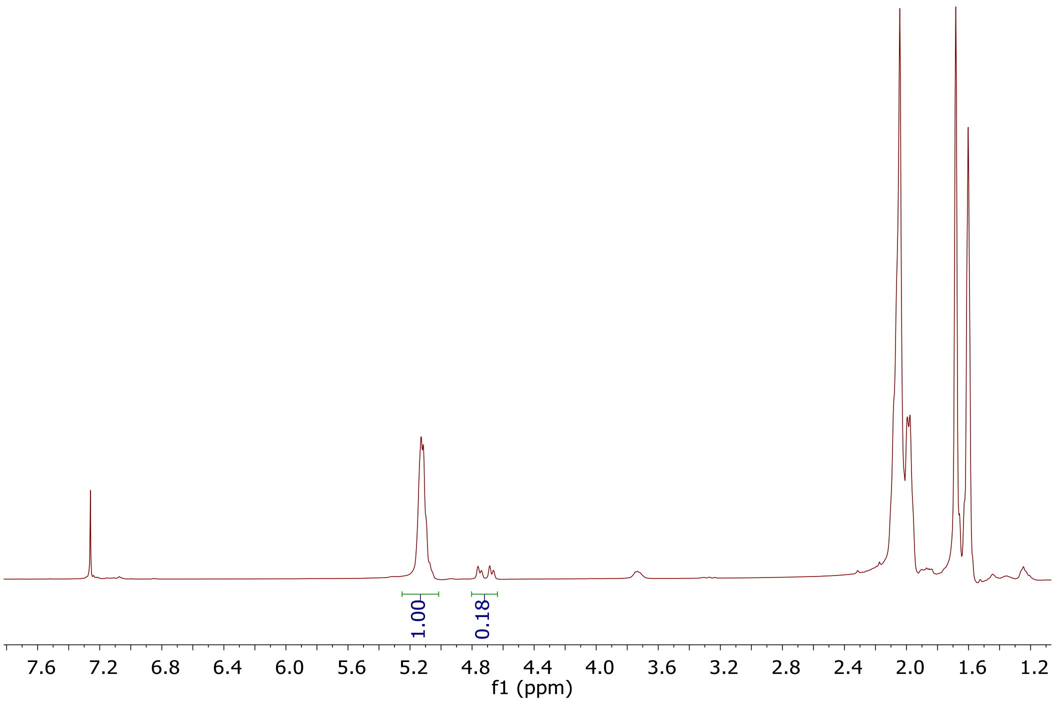

Fig. S43 ^{1}H NMR spectrum of PIP 500 equivalents generated by **$Y(CH_2SiMe_3)_3(THF)_2$**, 2 equivalents $[Ph_3C][B(C_6F_5)_4]$, and 15 equivalents $AlMe_3$ from **Table 4**, entry 3 in $CDCl_3$ at 298 K (30 min).

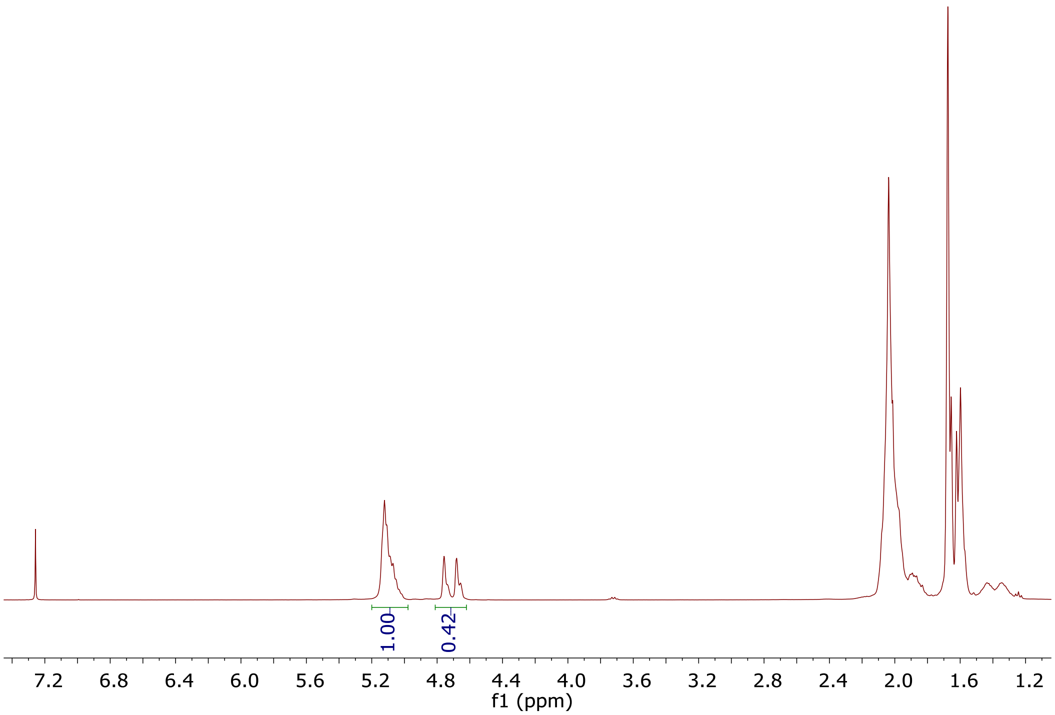

Fig. S44 ^{1}H NMR spectrum of PIP 500 equivalents generated by **$Y(CH_2SiMe_3)_3(THF)_2$**, 2 equivalents $[Ph_3C][B(C_6F_5)_4]$, and 5 equivalents $AlEt_3$ from **Table 4**, entry 4 in $CDCl_3$ at 298 K (30 min).

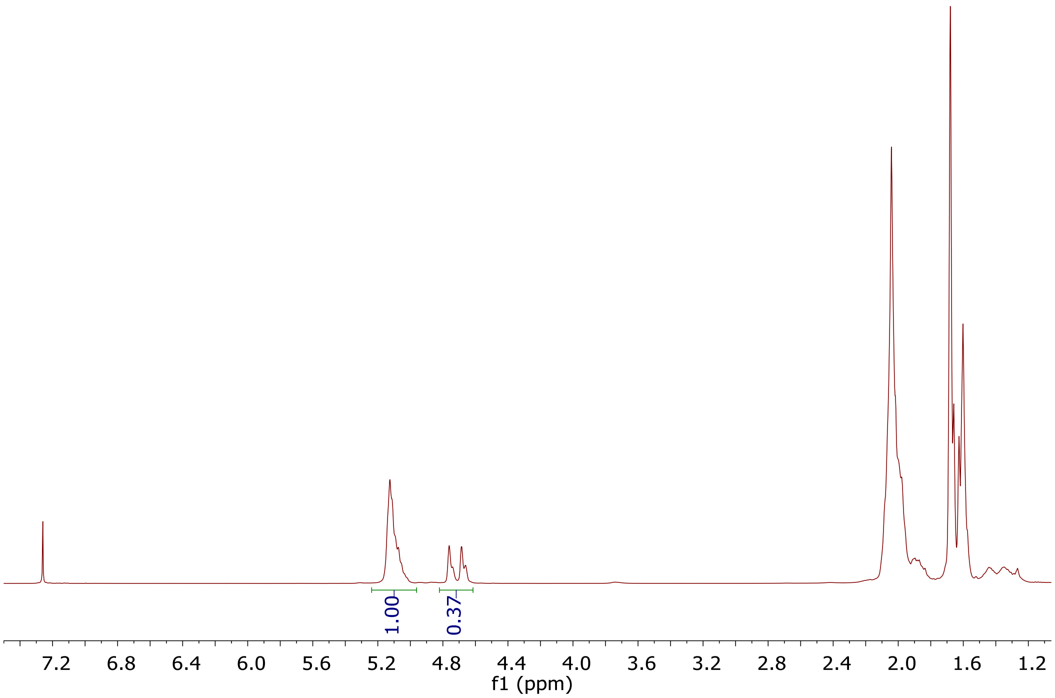

Fig. S45 ^{1}H NMR spectrum of PIP 500 equivalents generated by **$Y(CH_2SiMe_3)_3(THF)_2$**, 2 equivalents $[Ph_3C][B(C_6F_5)_4]$, and 10 equivalents $AlEt_3$ from **Table 4**, entry 5 in $CDCl_3$ at 298 K (30 min).

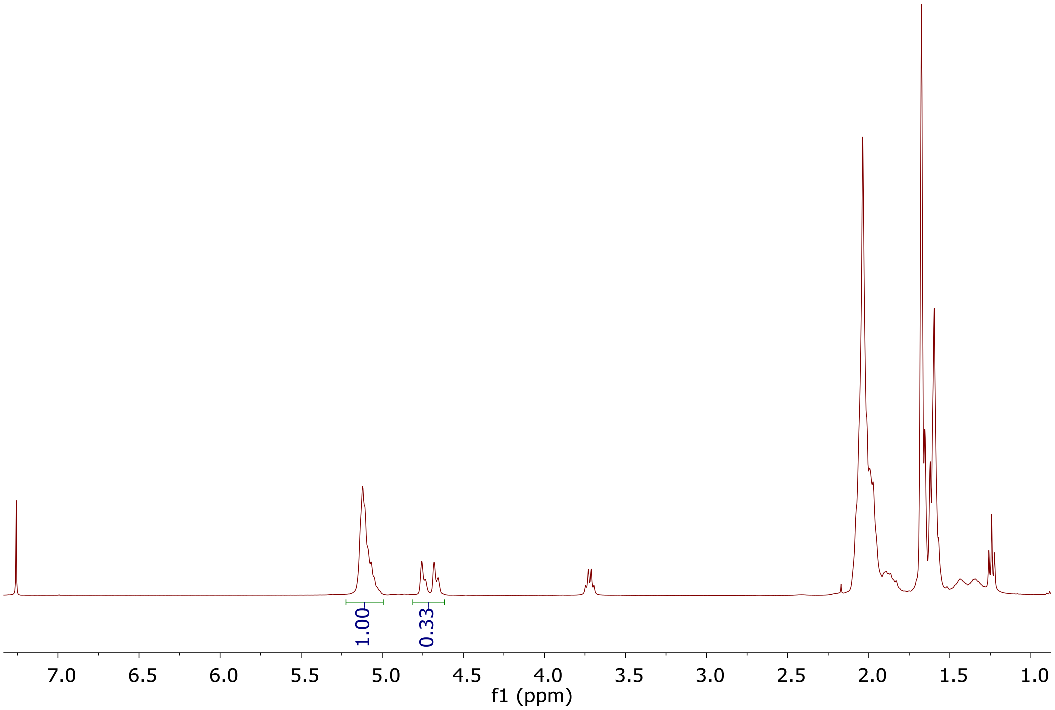

Fig. S46 ^{1}H NMR spectrum of PIP 500 equivalents generated by **$Y(CH_2SiMe_3)_3(THF)_2$**, 2 equivalents $[Ph_3C][B(C_6F_5)_4]$, and 15 equivalents $AlEt_3$ from **Table 4**, entry 6 in $CDCl_3$ at 298 K (30 min).

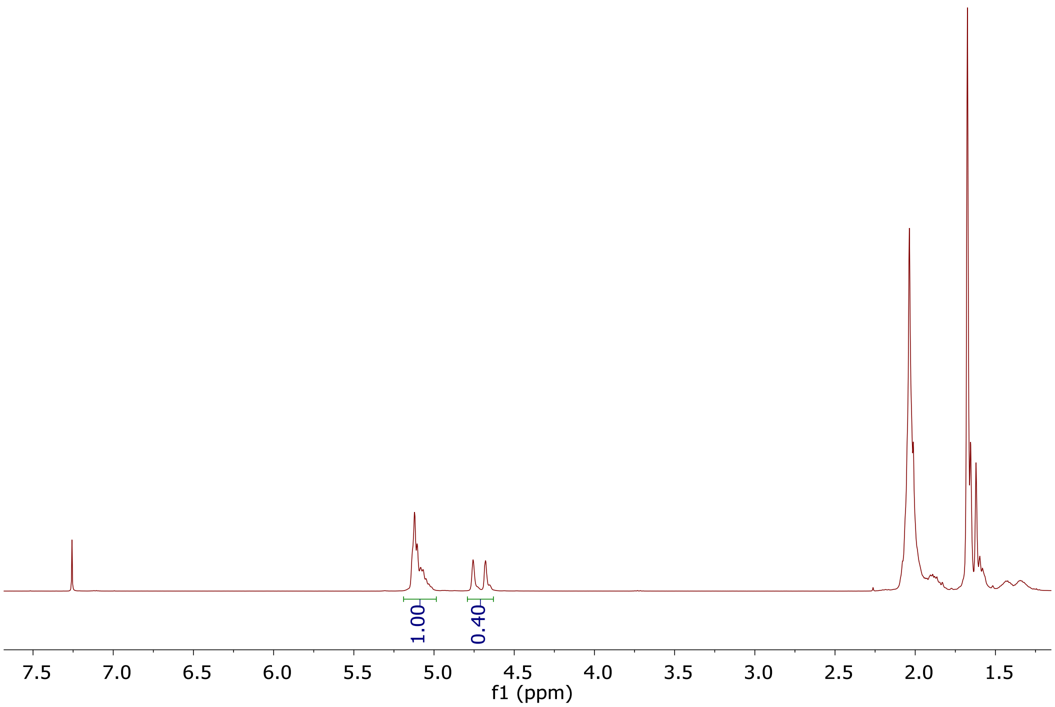

Fig. S47 ^{1}H NMR spectrum of PIP 500 equivalents generated by **Y(CH$_2$SiMe$_3$)$_3$(THF)$_2$**, 2 equivalents [Ph$_3$C][B(C$_6$F$_5$)$_4$], and 5 equivalents Al ^{i}Bu$_3$ from **Table 4**, entry 7 in CDCl$_3$ at 298 K (30 min).

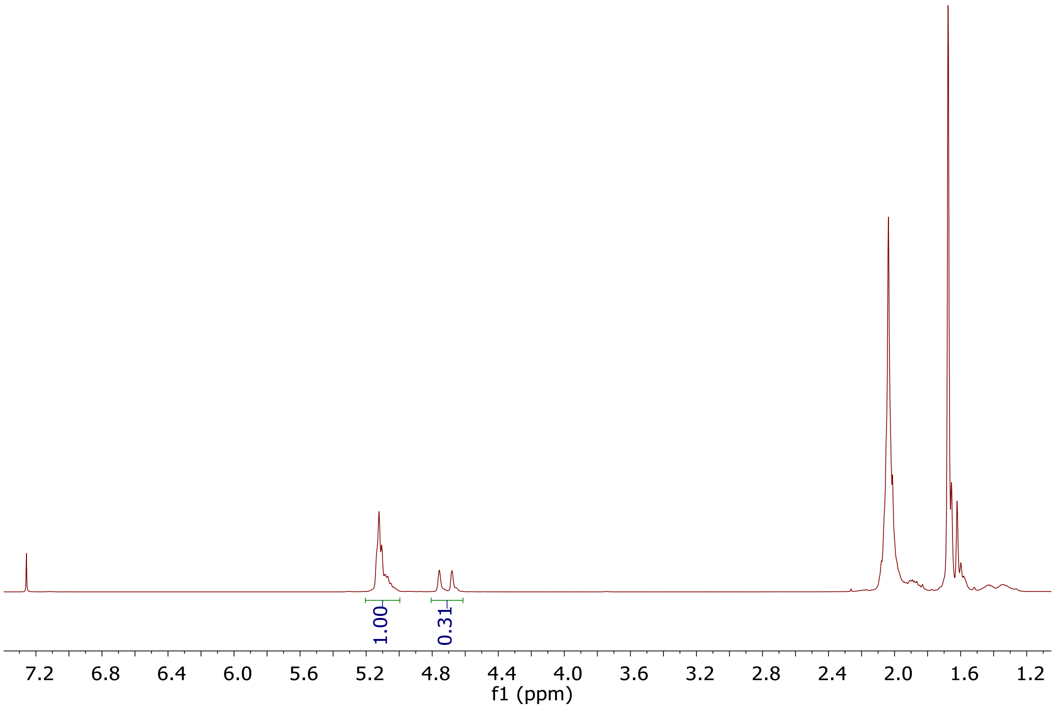

Fig. S48 ^{1}H NMR spectrum of PIP 500 equivalents generated by **Y(CH$_2$SiMe$_3$)$_3$(THF)$_2$**, 2 equivalents [Ph$_3$C][B(C$_6$F$_5$)$_4$], and 10 equivalents Al ^{i}Bu$_3$ from **Table 4**, entry 8 in CDCl$_3$ at 298 K (30 min).

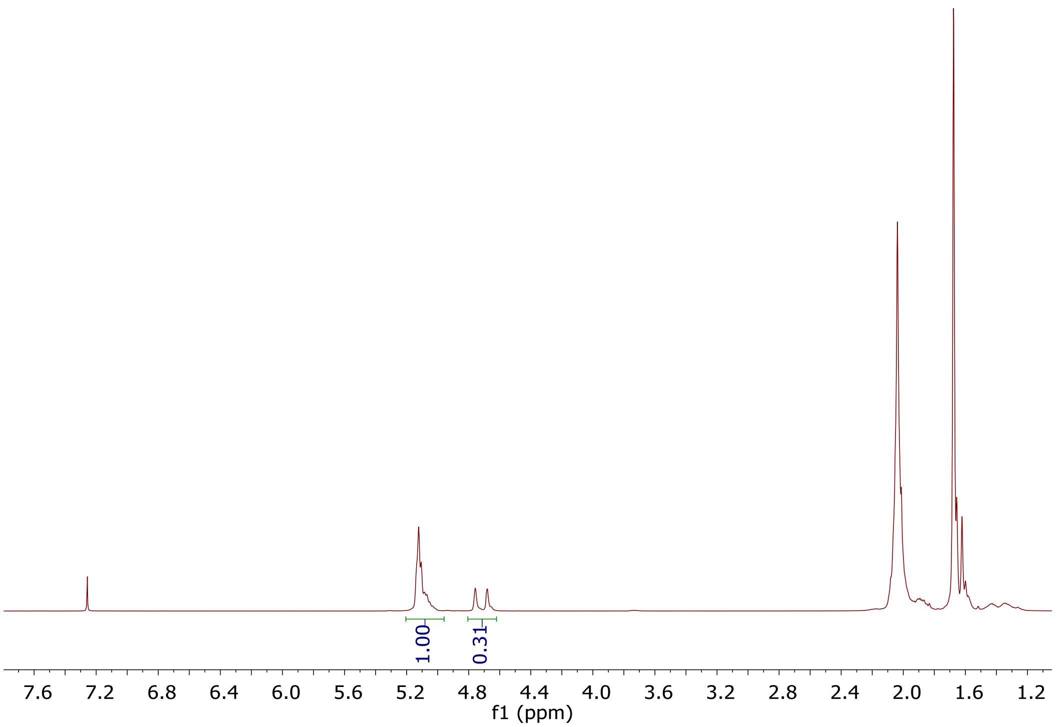

Fig. S49 ^{1}H NMR spectrum of PIP 500 equivalents generated by **Y(CH$_2$SiMe$_3$)$_3$(THF)$_2$**, 2 equivalents [Ph$_3$C][B(C$_6$F$_5$)$_4$], and 15 equivalents Al ^{i}Bu$_3$ from **Table 4**, entry 9 in CDCl$_3$ at 298 K (30 min).

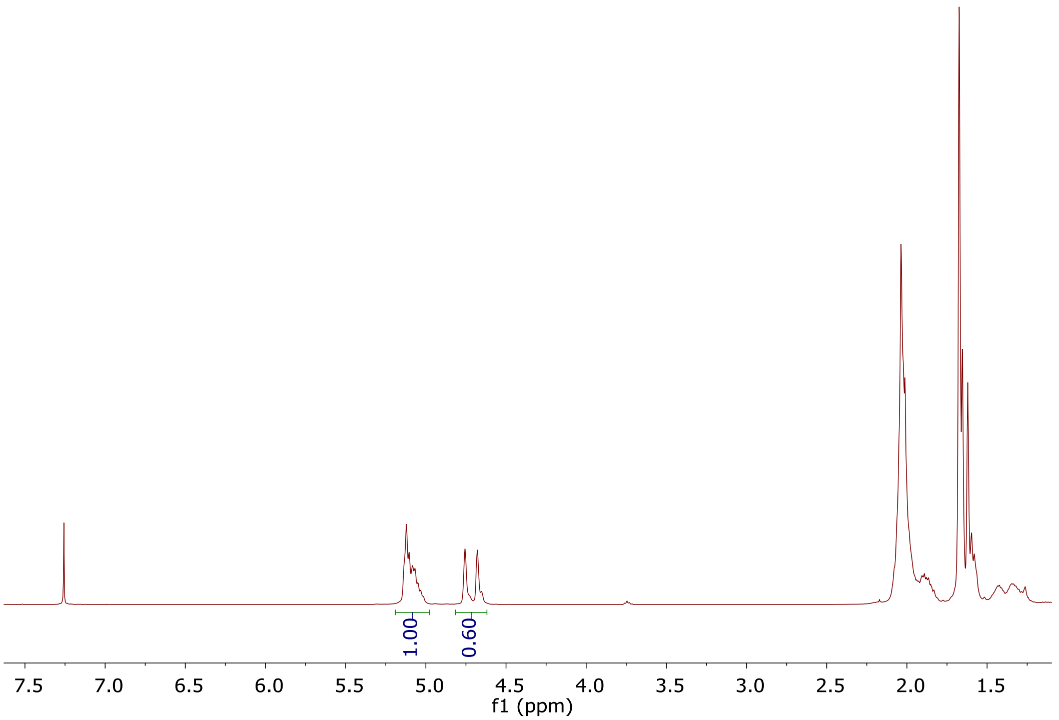

Fig. S50 ^{1}H NMR spectrum of PIP 500 equivalents generated by **Y(CH$_2$SiMe$_3$)$_3$(THF)$_2$**, 2 equivalents [Ph$_3$C][B(C$_6$F$_5$)$_4$], 1 equivalent PPh$_3$, and 5 equivalents Al ^{i}Bu$_3$ from **Table 4**, entry 10 in CDCl$_3$ at 298 K (30 min).

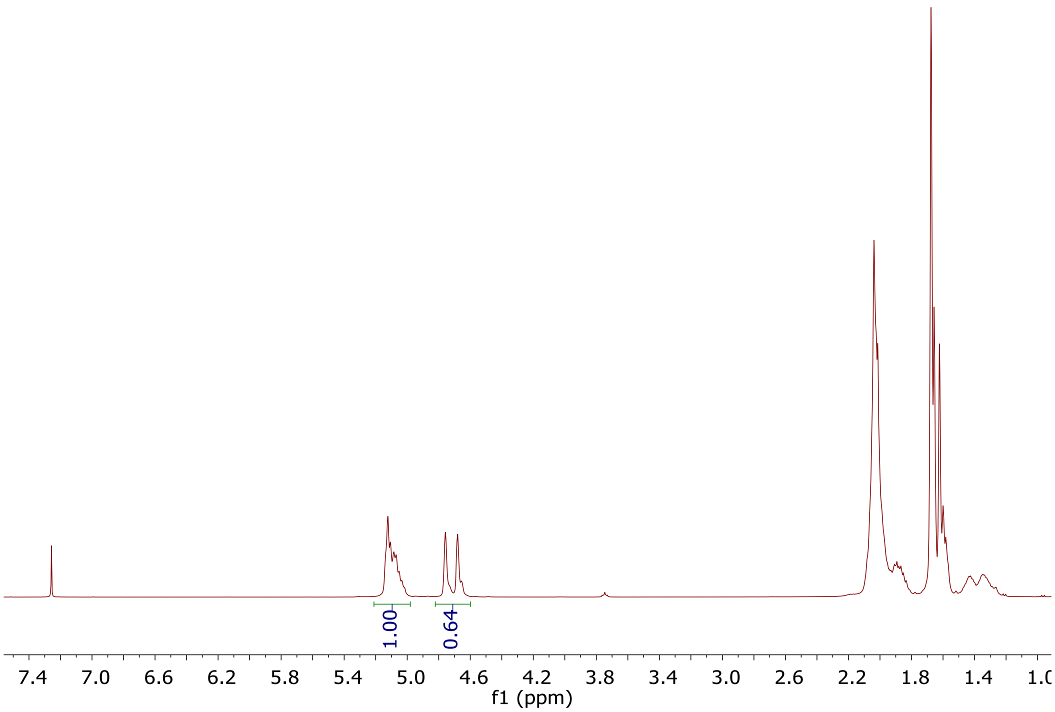

Fig. S51 ^{1}H NMR spectrum of PIP 500 equivalents generated by **$Y(CH_2SiMe_3)_3(THF)_2$**, 2 equivalents $[Ph_3C][B(C_6F_5)_4]$, 1 equivalent PPh_3, and 10 equivalents Al ^{i}Bu_3 from **Table 4**, entry 11 in $CDCl_3$ at 298 K (30 min).

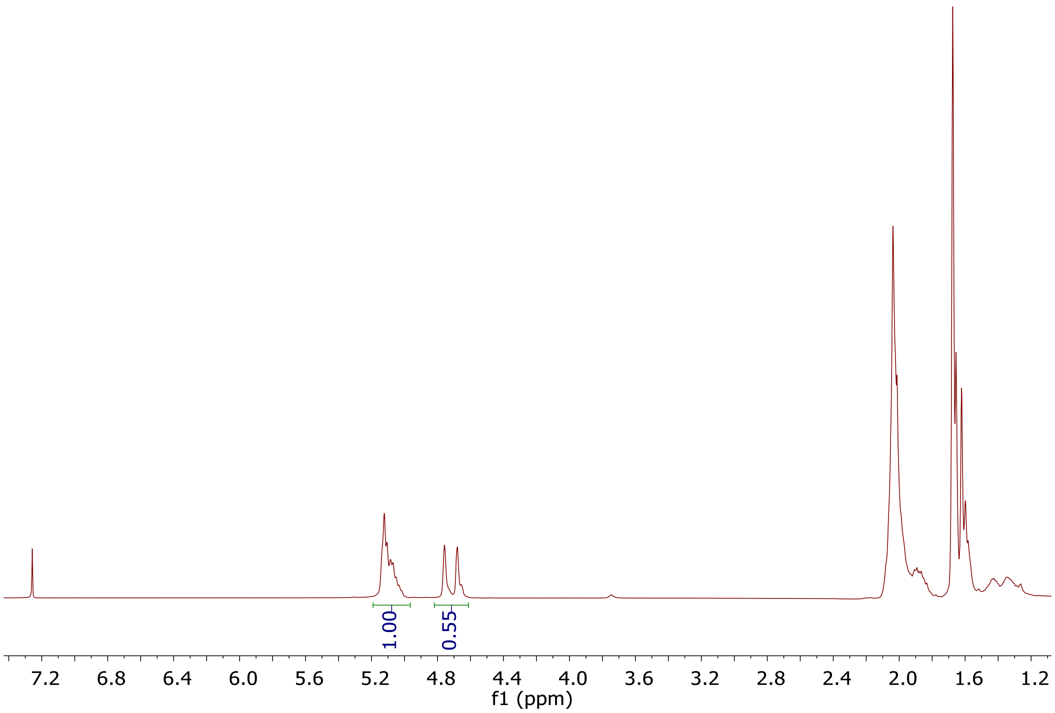

Fig. S52 ^{1}H NMR spectrum of PIP 500 equivalents generated by **$Y(CH_2SiMe_3)_3(THF)_2$**, 2 equivalents $[Ph_3C][B(C_6F_5)_4]$, 1 equivalent PPh_3, and 15 equivalents Al ^{i}Bu_3 from **Table 4**, entry 12 in $CDCl_3$ at 298 K (30 min).

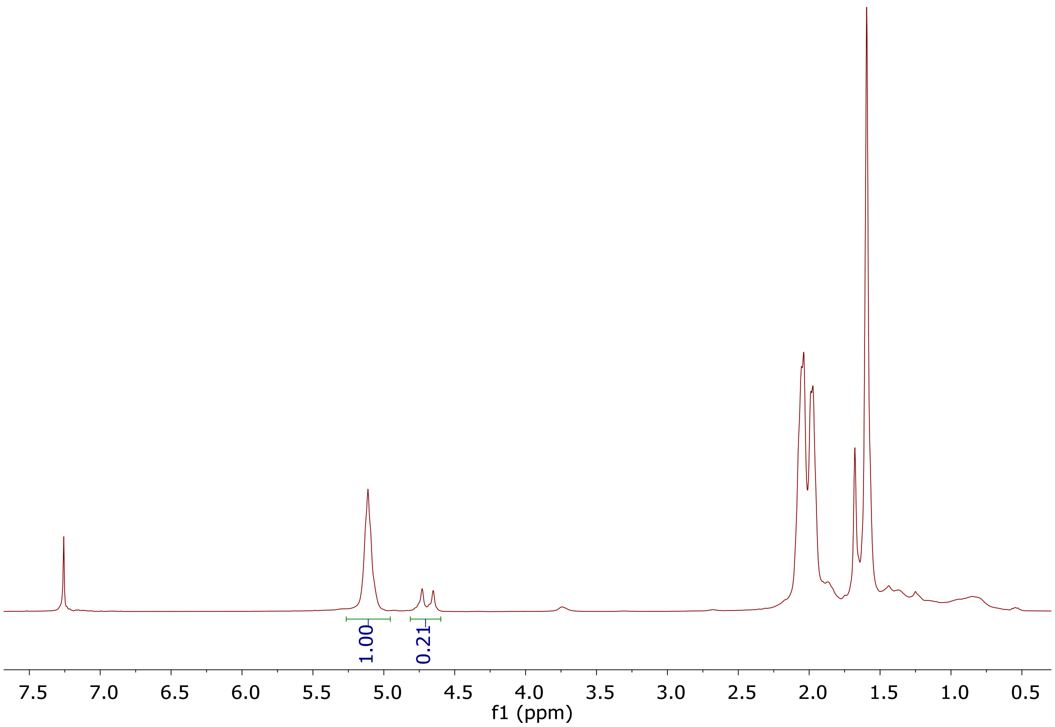

Fig. S53 ^1H NMR spectrum of PIP 500 equivalents generated by **Sm(CH$_2$SiMe$_3$)$_3$(THF)$_3$**, 1 equivalent [Ph$_3$C][B(C$_6$F$_5$)$_4$] from **Table 5**, entry 1 in CDCl$_3$ at 298 K (30 min).

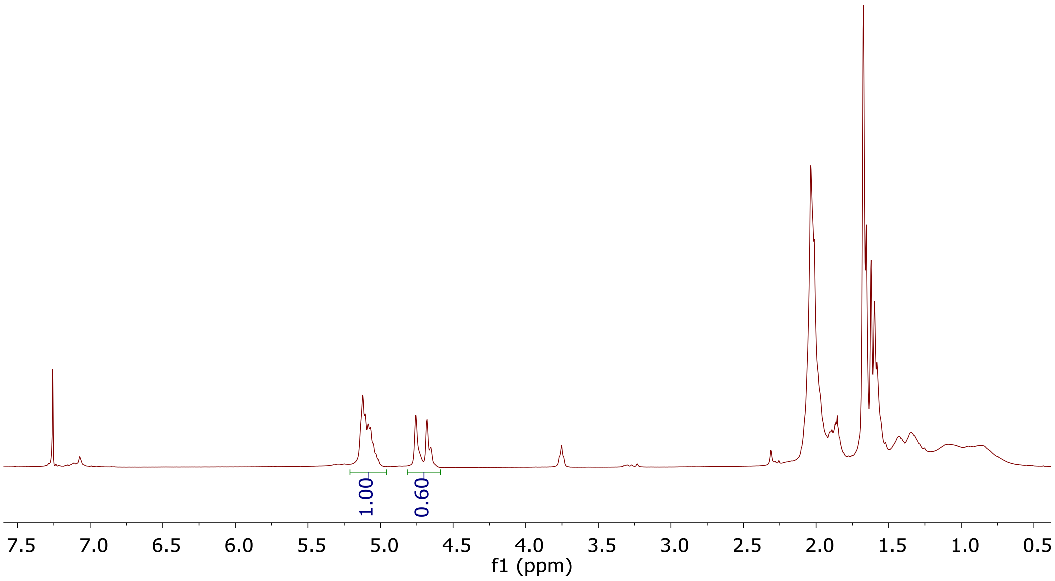

Fig. S54 ^1H NMR spectrum of PIP 500 equivalents generated by **Sm(CH$_2$SiMe$_3$)$_3$(THF)$_3$**, 2 equivalents [Ph$_3$C][B(C$_6$F$_5$)$_4$] from **Table 5**, entry 2 in CDCl$_3$ at 298 K (30 min).

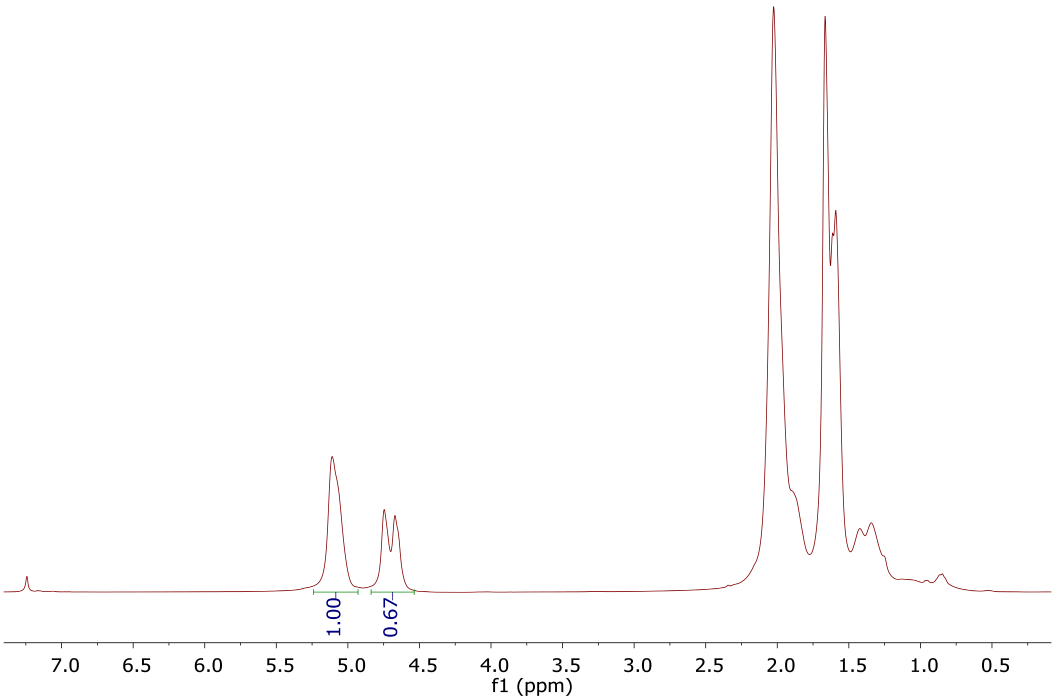

Fig. S55 ^{1}H NMR spectrum of PIP 500 equivalents generated by **Gd(CH_2SiMe_3)$_3$(THF)$_2$**, 1 equivalent [Ph_3C][B(C_6F_5)$_4$] from **Table 5**, entry 3 in $CDCl_3$ at 298 K (30 min).

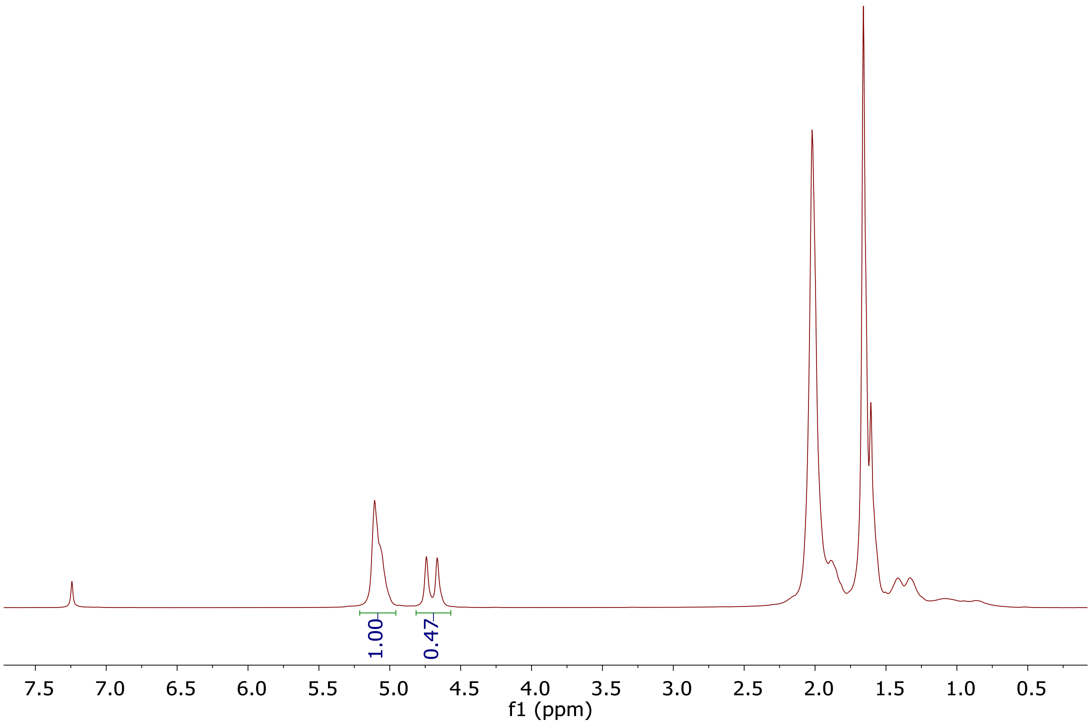

Fig. S56 ^{1}H NMR spectrum of PIP 500 equivalents generated by **Gd(CH_2SiMe_3)$_3$(THF)$_2$**, 2 equivalents [Ph_3C][B(C_6F_5)$_4$] from **Table 5**, entry 4 in $CDCl_3$ at 298 K (30 min).

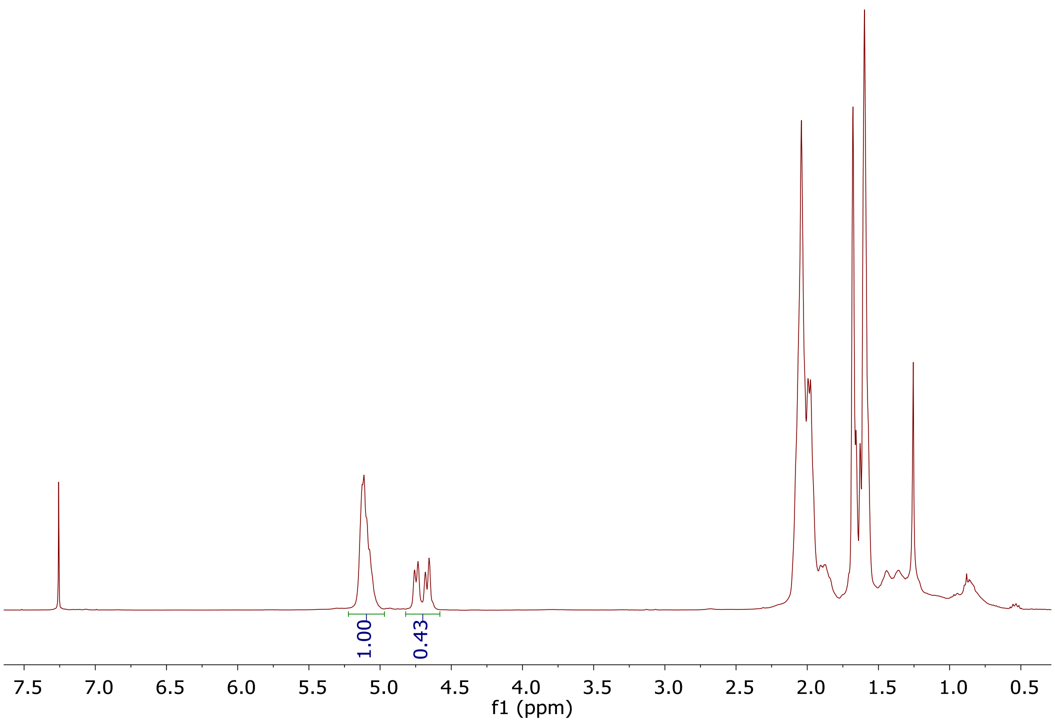

Fig. S57 ^{1}H NMR spectrum of PIP 500 equivalents generated by **$Y(CH_2SiMe_3)_3(THF)_2$**, 1 equivalent $[Ph_3C][B(C_6F_5)_4]$ from **Table 5**, entry 5 in $CDCl_3$ at 298 K (30 min).

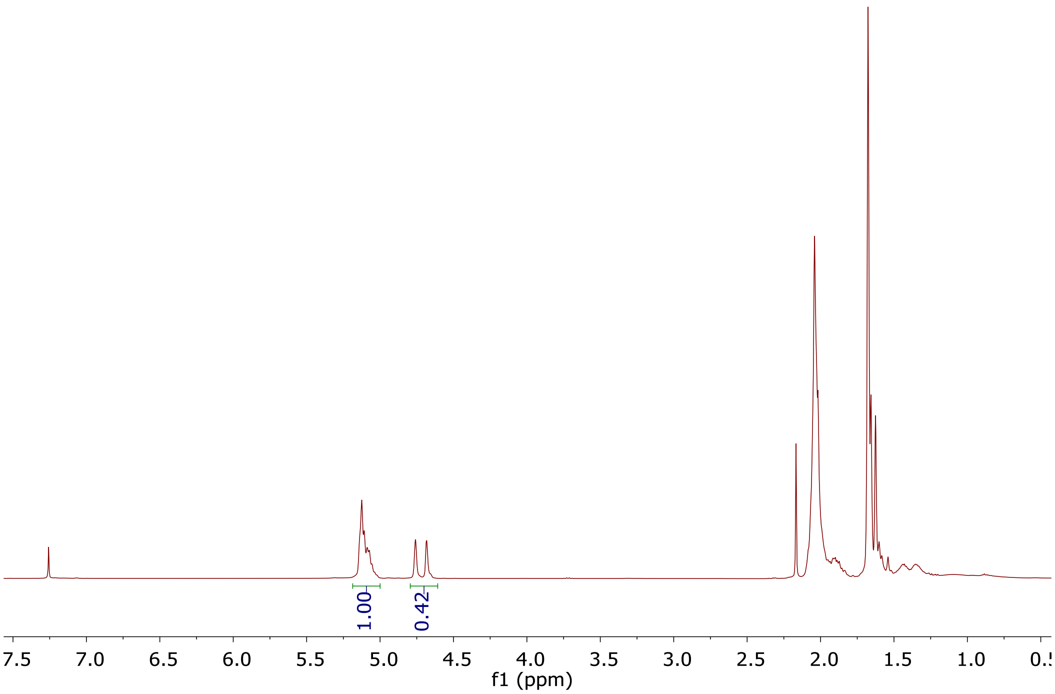

Fig. S58 ^{1}H NMR spectrum of PIP 500 equivalents generated by **$Y(CH_2SiMe_3)_3(THF)_2$**, 2 equivalents $[Ph_3C][B(C_6F_5)_4]$ from **Table 5**, entry 6 in $CDCl_3$ at 298 K (30 min).

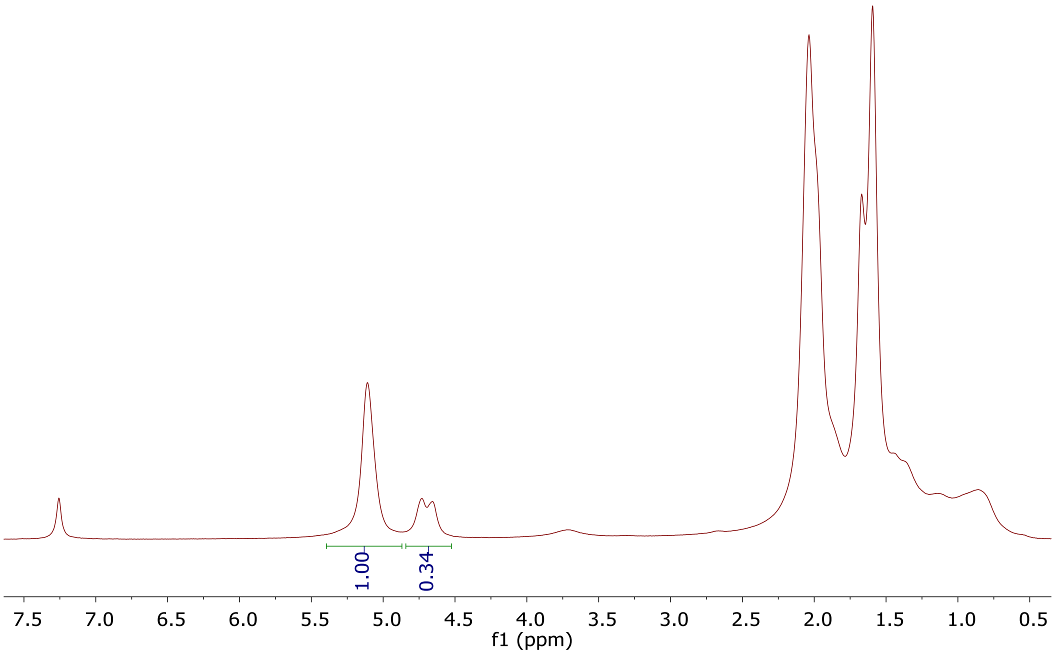

Fig. S59 ^{1}H NMR spectrum of PIP 500 equivalents generated by **Tm(CH$_2$SiMe$_3$)$_3$(THF)$_2$**, 1 equivalent [Ph$_3$C][B(C$_6$F$_5$)$_4$] from **Table 5**, entry 7 in CDCl$_3$ at 298 K (30 min).

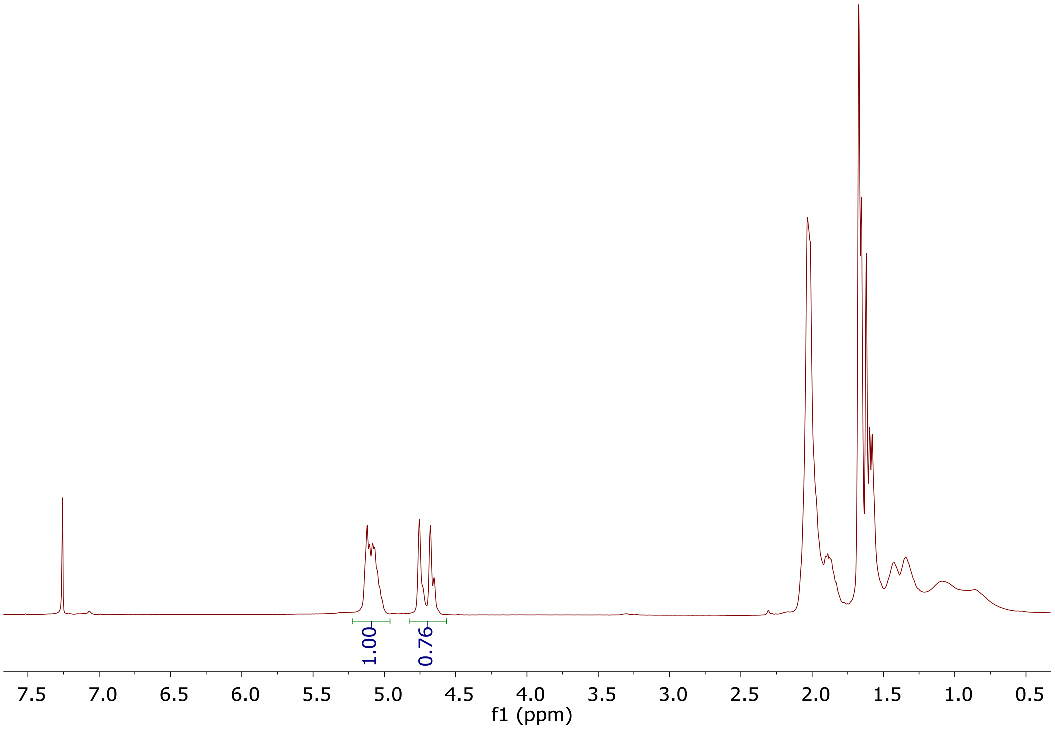

Fig. S60 ^{1}H NMR spectrum of PIP 500 equivalents generated by **Tm(CH$_2$SiMe$_3$)$_3$(THF)$_2$**, 2 equivalents [Ph$_3$C][B(C$_6$F$_5$)$_4$] from **Table 5**, entry 8 in CDCl$_3$ at 298 K (30 min).

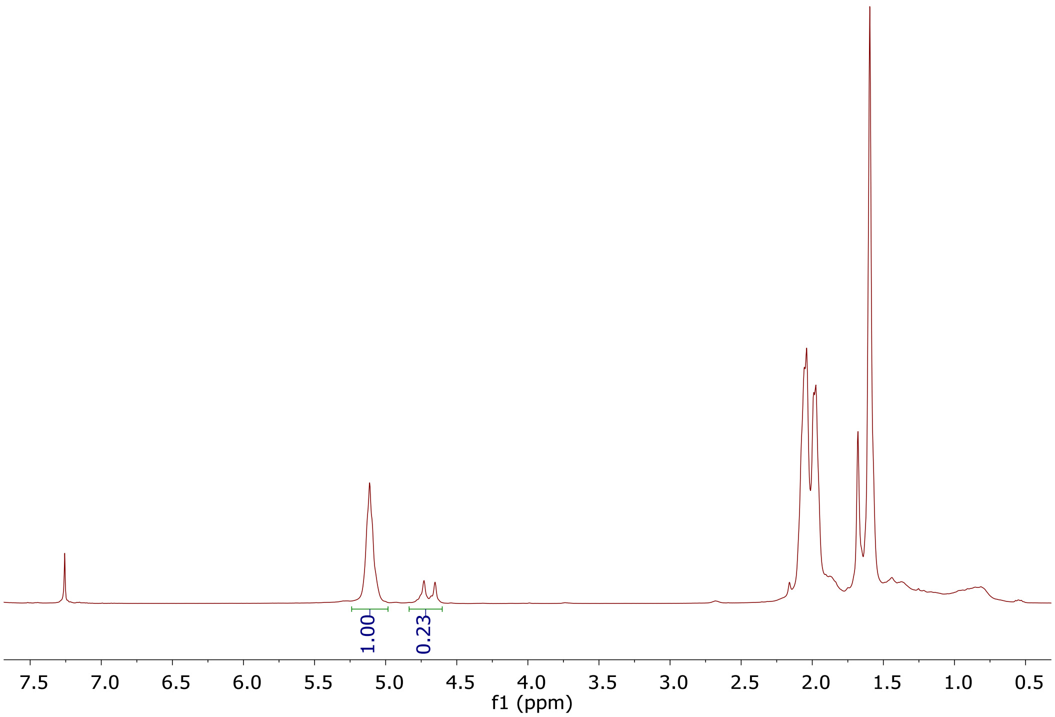

Fig. S61 ^{1}H NMR spectrum of PIP 500 equivalents generated by **Sm(CH$_2$SiMe$_3$)$_3$(THF)$_3$**,1 equivalent [Ph$_3$C][B(C$_6$F$_5$)$_4$], and 1 equivalent PPh$_3$ from **Table 5**, entry 9 in CDCl$_3$ at 298 K (30 min).

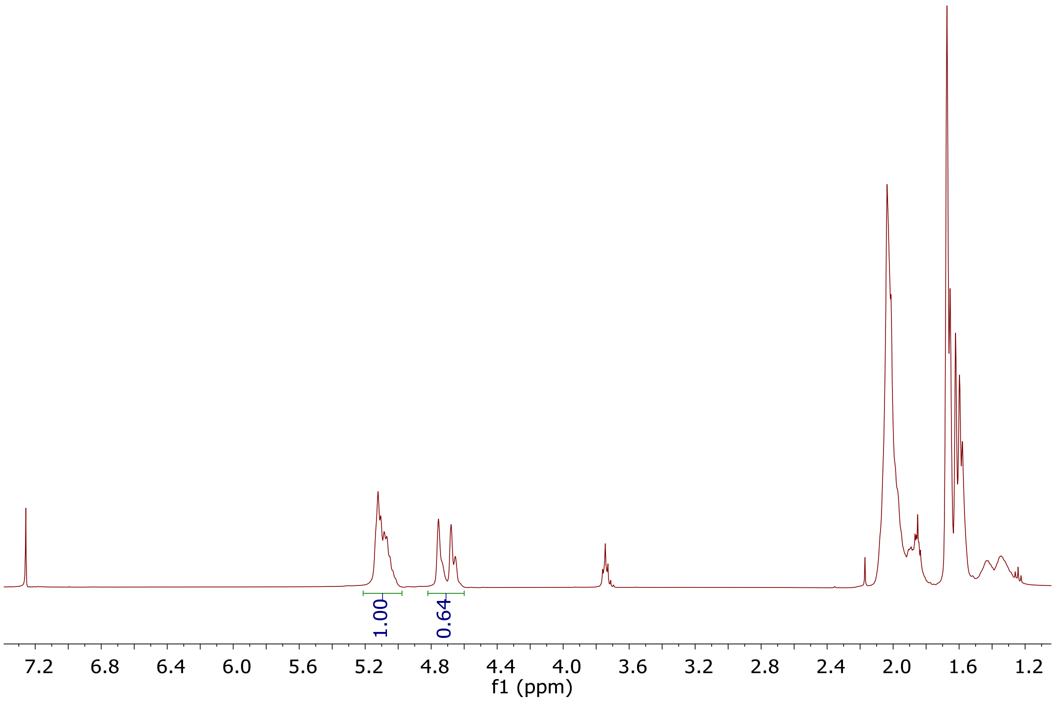

Fig. S62 ^{1}H NMR spectrum of PIP 500 equivalents generated by **Sm(CH$_2$SiMe$_3$)$_3$(THF)$_3$**, 2 equivalents [Ph$_3$C][B(C$_6$F$_5$)$_4$], and 1 equivalent PPh$_3$ from **Table 5**, entry 10 in CDCl$_3$ at 298 K (30 min).

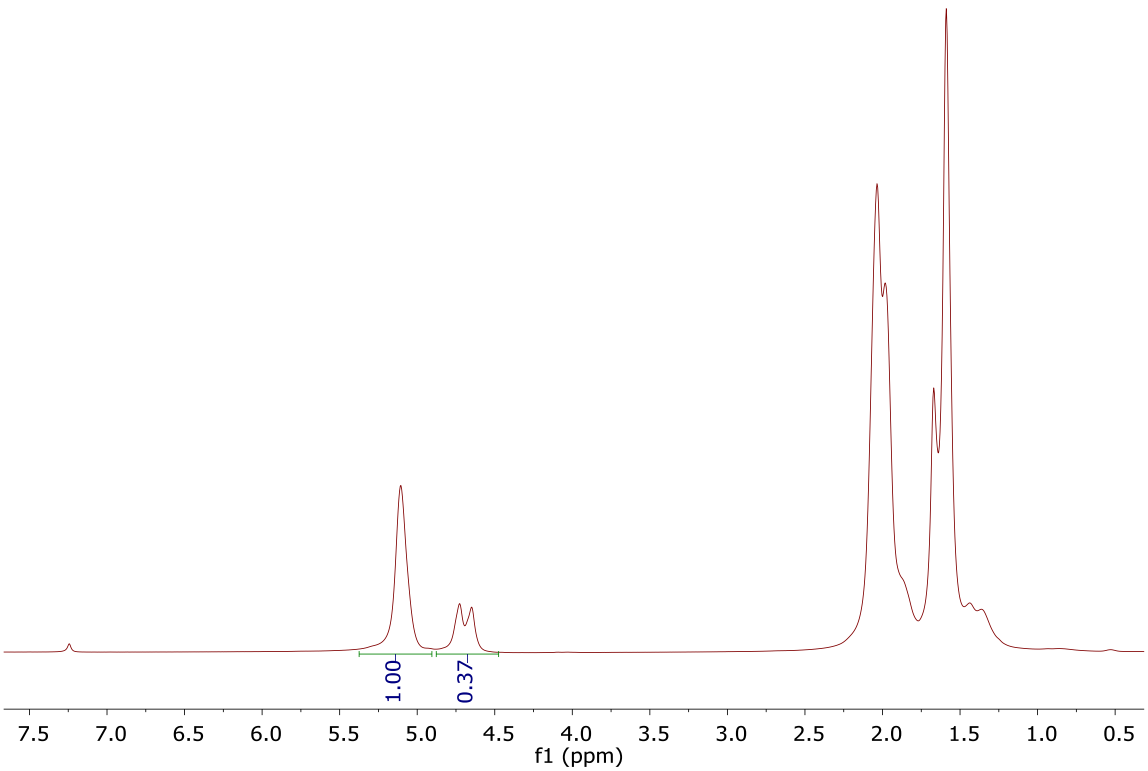

Fig. S63 ^{1}H NMR spectrum of PIP 500 equivalents generated by **Gd(CH$_2$SiMe$_3$)$_3$(THF)$_2$**, 1 equivalent [Ph$_3$C][B(C$_6$F$_5$)$_4$], and 1 equivalent PPh$_3$ from **Table 5**, entry 11 in CDCl$_3$ at 298 K (30 min).

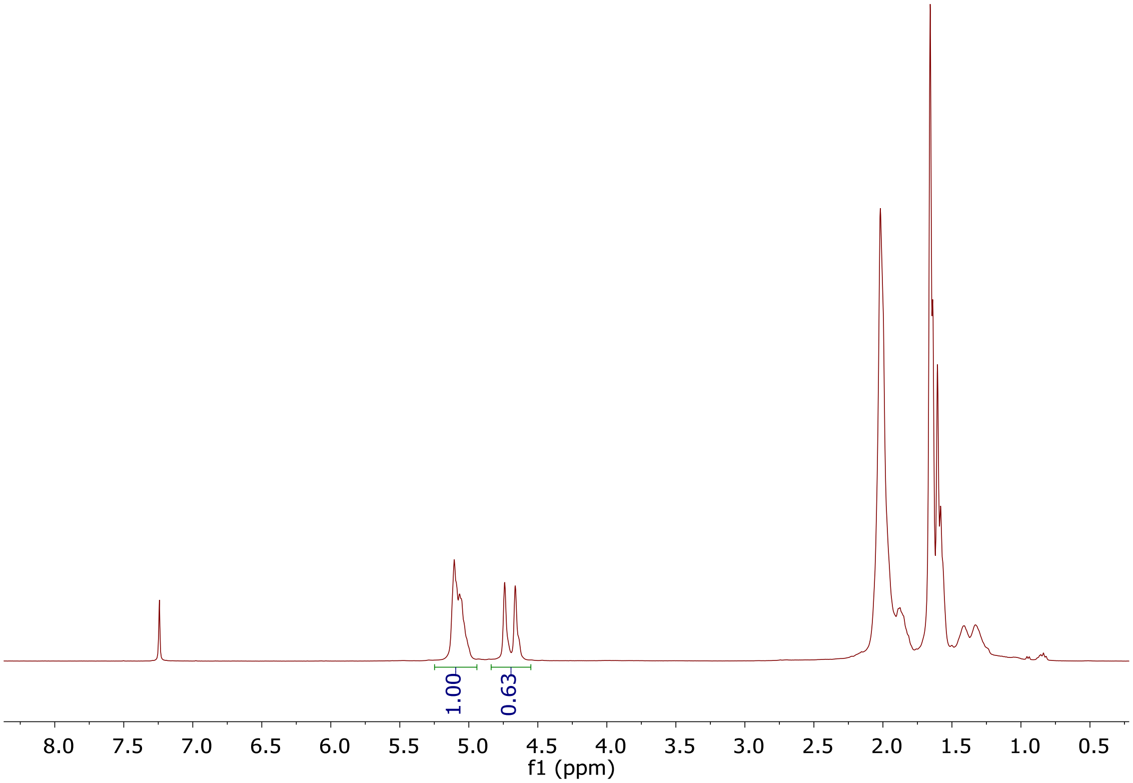

Fig. S64 ^{1}H NMR spectrum of PIP 500 equivalents generated by **Gd(CH$_2$SiMe$_3$)$_3$(THF)$_2$**, 2 equivalents [Ph$_3$C][B(C$_6$F$_5$)$_4$], and 1 equivalent PPh$_3$ from **Table 5**, entry 12 in CDCl$_3$ at 298 K (30 min).

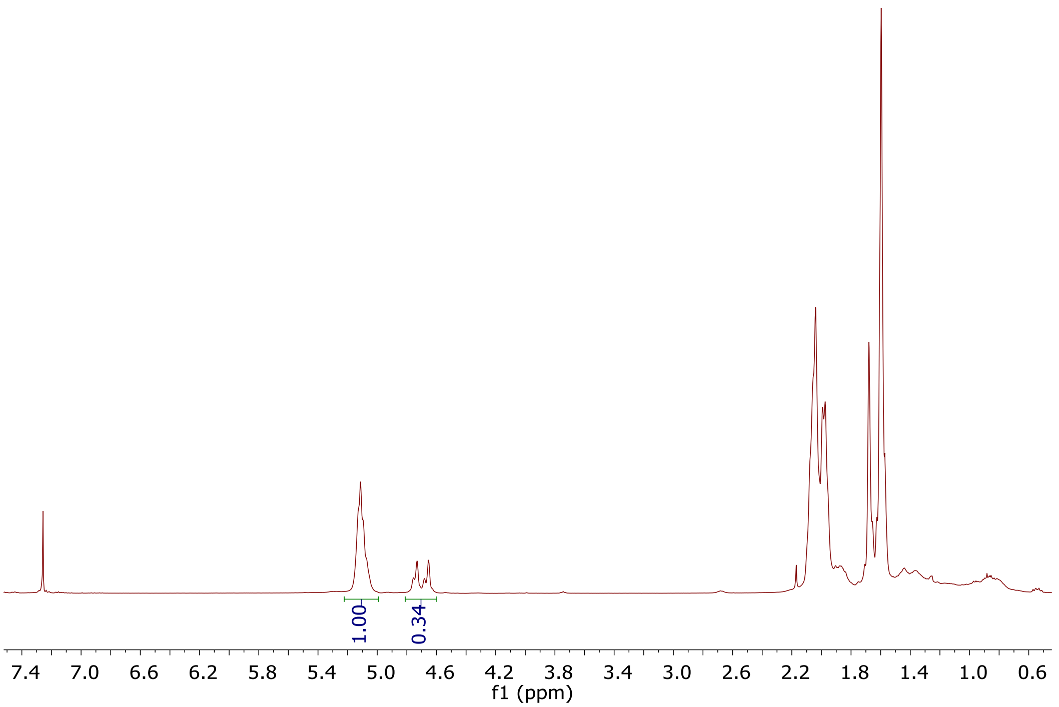

Fig. S65 ^{1}H NMR spectrum of PIP 500 equivalents generated by **$Y(CH_2SiMe_3)_3(THF)_2$**, 1 equivalent $[Ph_3C][B(C_6F_5)_4]$, and 1 equivalent PPh_3 from **Table 5**, entry 13 in $CDCl_3$ at 298 K (30 min).

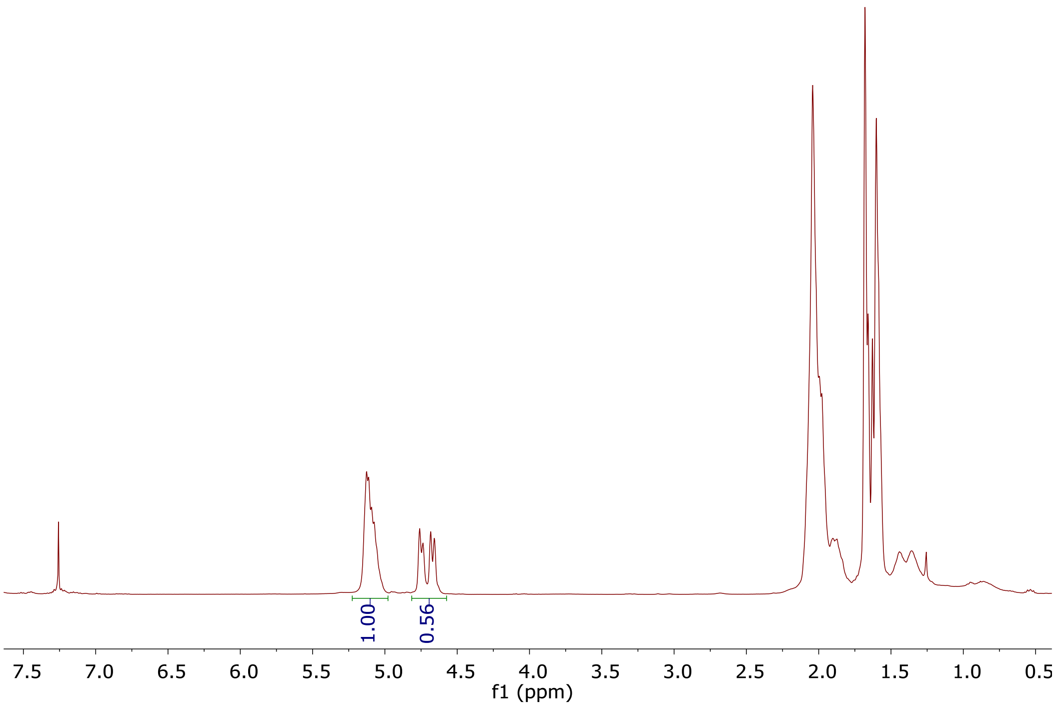

Fig. S66 ^{1}H NMR spectrum of PIP 500 equivalents generated by **$Y(CH_2SiMe_3)_3(THF)_2$**, 2 equivalents $[Ph_3C][B(C_6F_5)_4]$, and 1 equivalent PPh_3 from **Table 5**, entry 14 in $CDCl_3$ at 298 K (30 min).

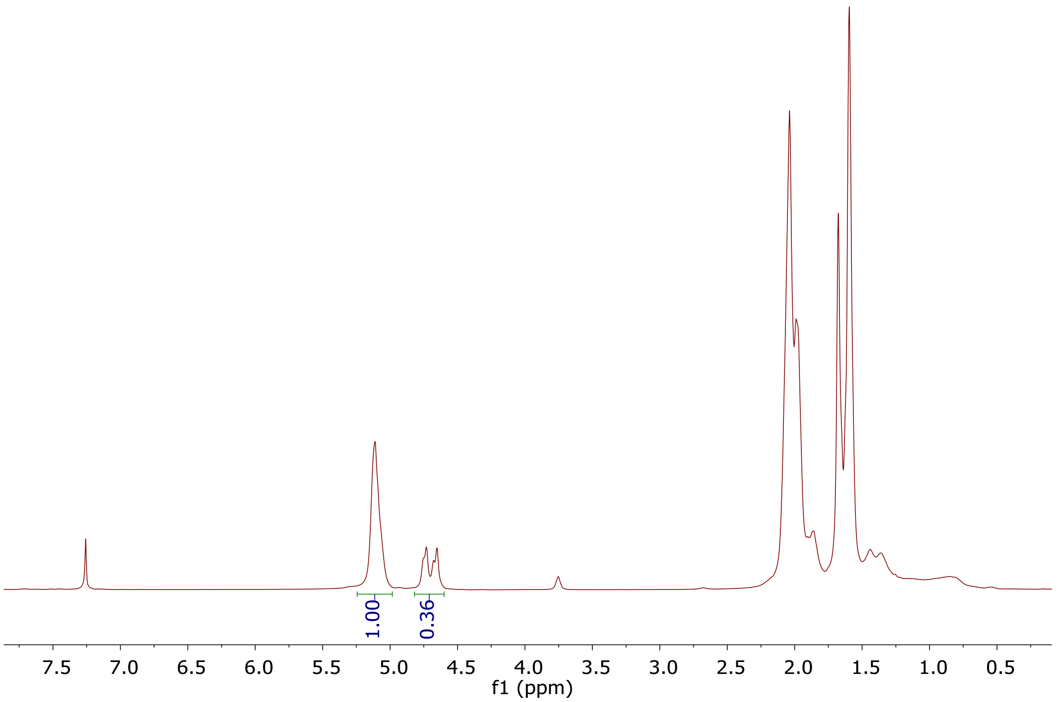

Fig. S67 ^{1}H NMR spectrum of PIP 500 equivalents generated by **Tm(CH$_2$SiMe$_3$)$_3$(THF)$_2$**, 1 equivalent [Ph$_3$C][B(C$_6$F$_5$)$_4$], and 1 equivalent PPh$_3$ from **Table 5**, entry 15 in CDCl$_3$ at 298 K (30 min).

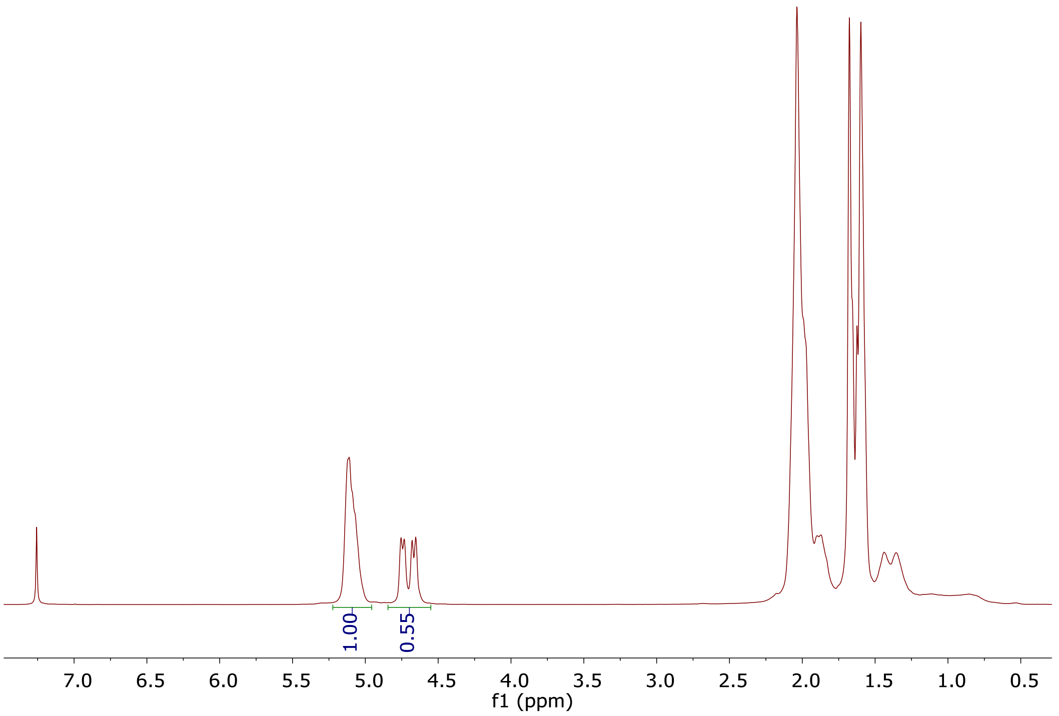

Fig. S68 ^{1}H NMR spectrum of PIP 500 equivalents generated by **Tm(CH$_2$SiMe$_3$)$_3$(THF)$_2$**, 2 equivalents [Ph$_3$C][B(C$_6$F$_5$)$_4$], and 1 equivalent PPh$_3$ from **Table 5**, entry 16 in CDCl$_3$ at 298 K (30 min).

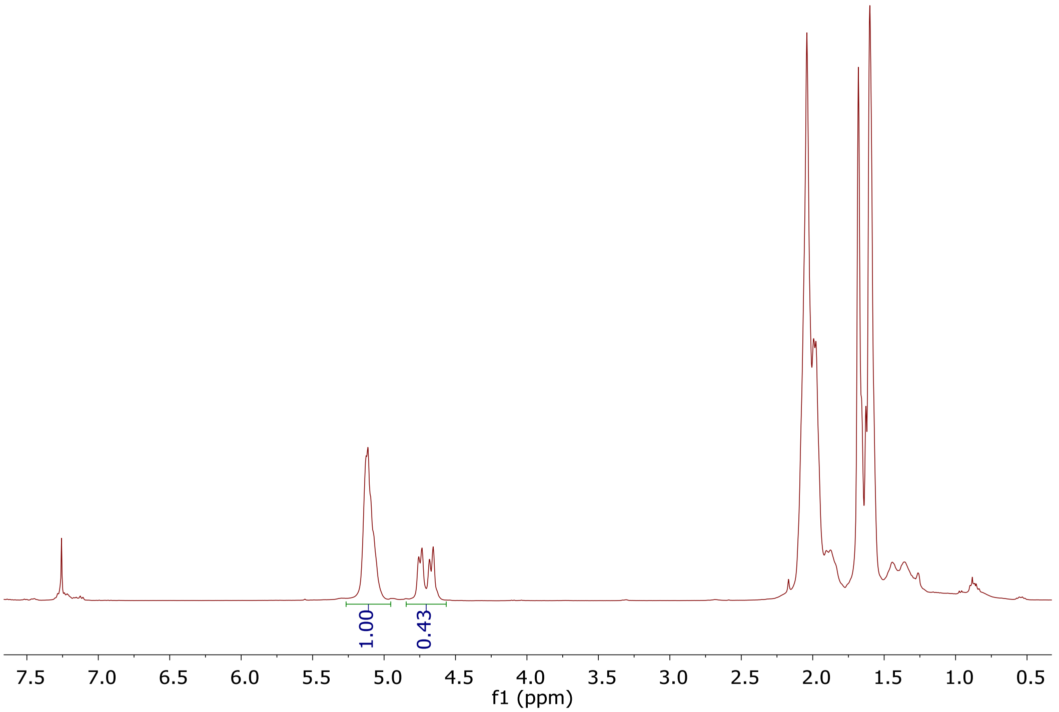

Fig. S69 ^{1}H NMR spectrum of PIP 500 equivalents generated by **Sm(CH$_2$SiMe$_3$)$_3$(THF)$_3$**, 1 equivalent PPh$_3$ and 2 equivalents [Ph$_3$C][B(C$_6$F$_5$)$_4$] from **Table 6**, entry 1 in CDCl$_3$ at 298 K ([Ph$_3$C][B(C$_6$F$_5$)$_4$] addition time 0 min).

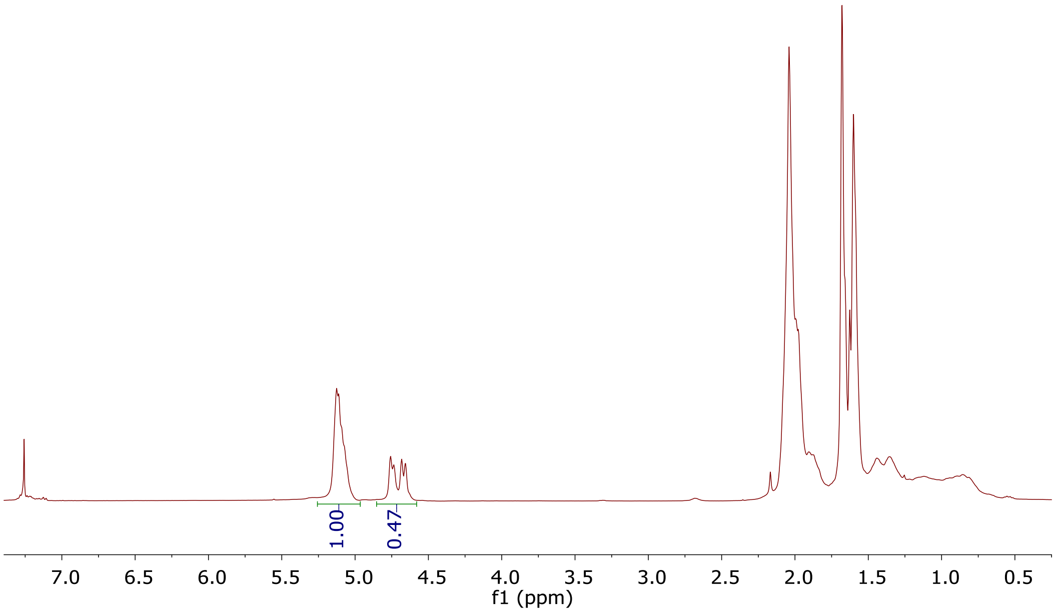

Fig. S70 ^{1}H NMR spectrum of PIP 500 equivalents generated by **Sm(CH$_2$SiMe$_3$)$_3$(THF)$_3$**, 1 equivalent PPh$_3$, and 2 equivalents [Ph$_3$C][B(C$_6$F$_5$)$_4$] from **Table 6**, entry 2 in CDCl$_3$ at 298 K ([Ph$_3$C][B(C$_6$F$_5$)$_4$] addition time 10 min).

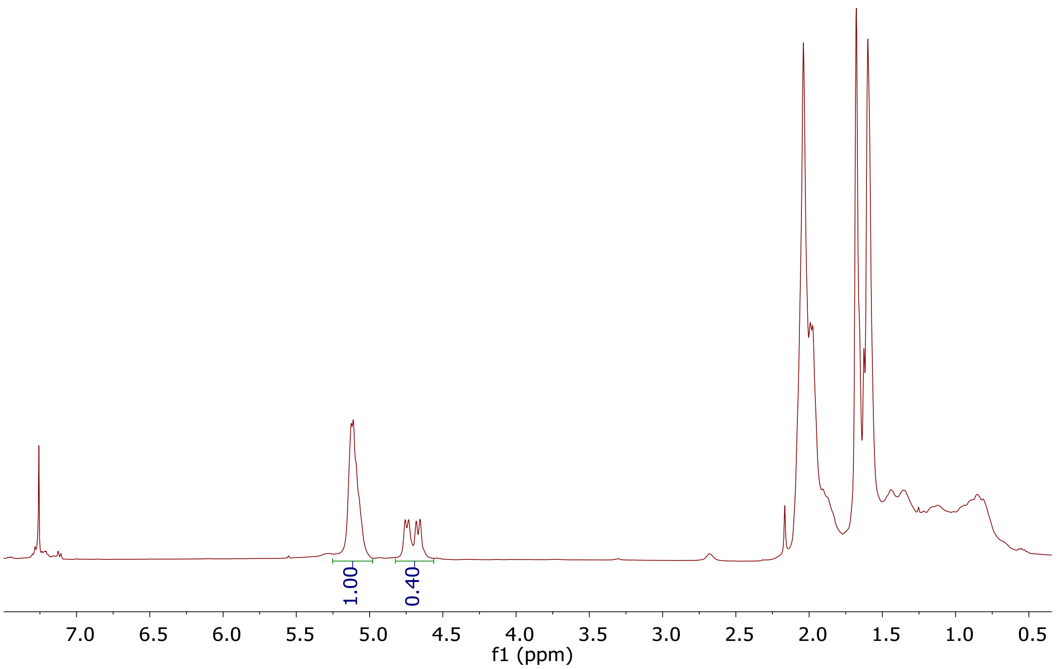

Fig. S71 ^{1}H NMR spectrum of PIP 500 equivalents generated by **$Sm(CH_2SiMe_3)_3(THF)_3$**, 1 equivalent PPh_3, and 2 equivalents $[Ph_3C][B(C_6F_5)_4]$ from **Table 6**, entry 3 in $CDCl_3$ at 298 K ($[Ph_3C][B(C_6F_5)_4]$ addition time 30 min).

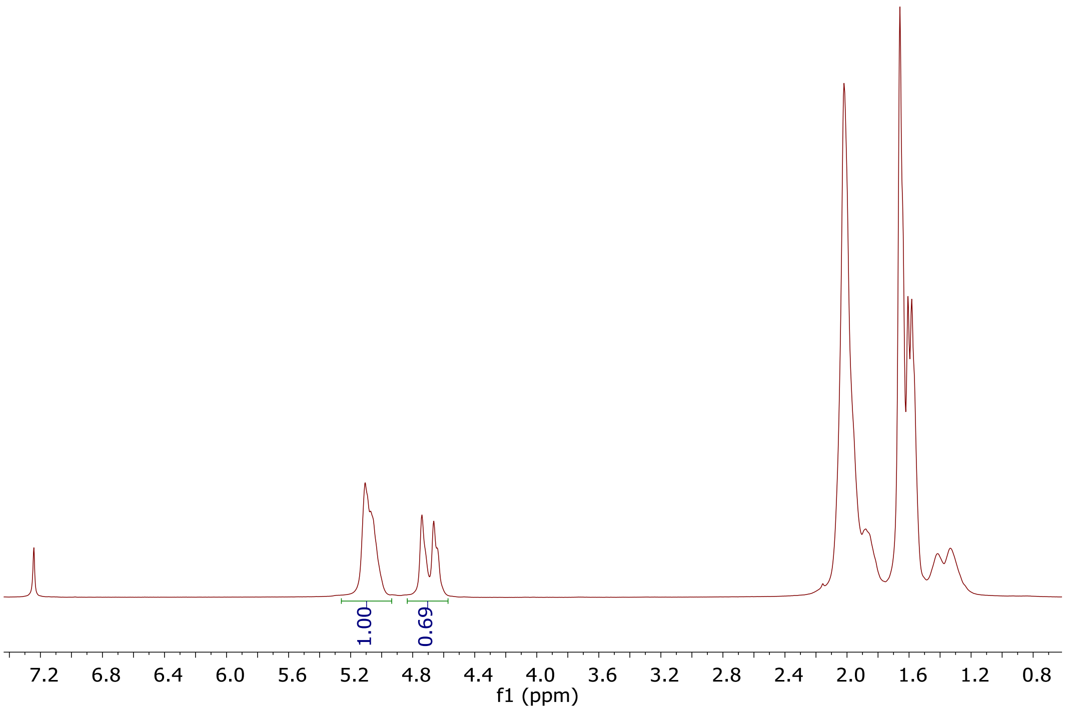

Fig. S72 ^{1}H NMR spectrum of PIP 500 equivalents generated by **$Gd(CH_2SiMe_3)_3(THF)_2$**, 1 equivalent PPh_3 and 2 equivalents $[Ph_3C][B(C_6F_5)_4]$ from **Table 6**, entry 4 in $CDCl_3$ at 298 K ($[Ph_3C][B(C_6F_5)_4]$ addition time 0 min).

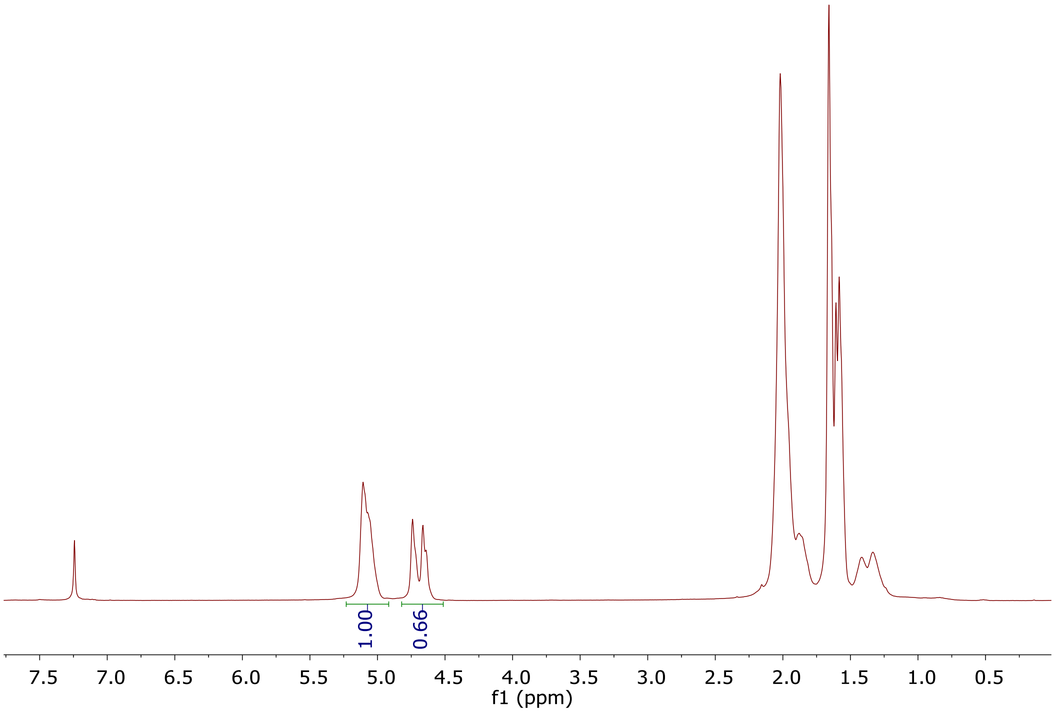

Fig. S73 ^{1}H NMR spectrum of PIP 500 equivalents generated by **Gd(CH$_2$SiMe$_3$)$_3$(THF)$_2$**, 1 equivalent PPh$_3$, and 2 equivalents [Ph$_3$C][B(C$_6$F$_5$)$_4$] from **Table 6**, entry 5 in CDCl$_3$ at 298 K ([Ph$_3$C][B(C$_6$F$_5$)$_4$] addition time 10 min).

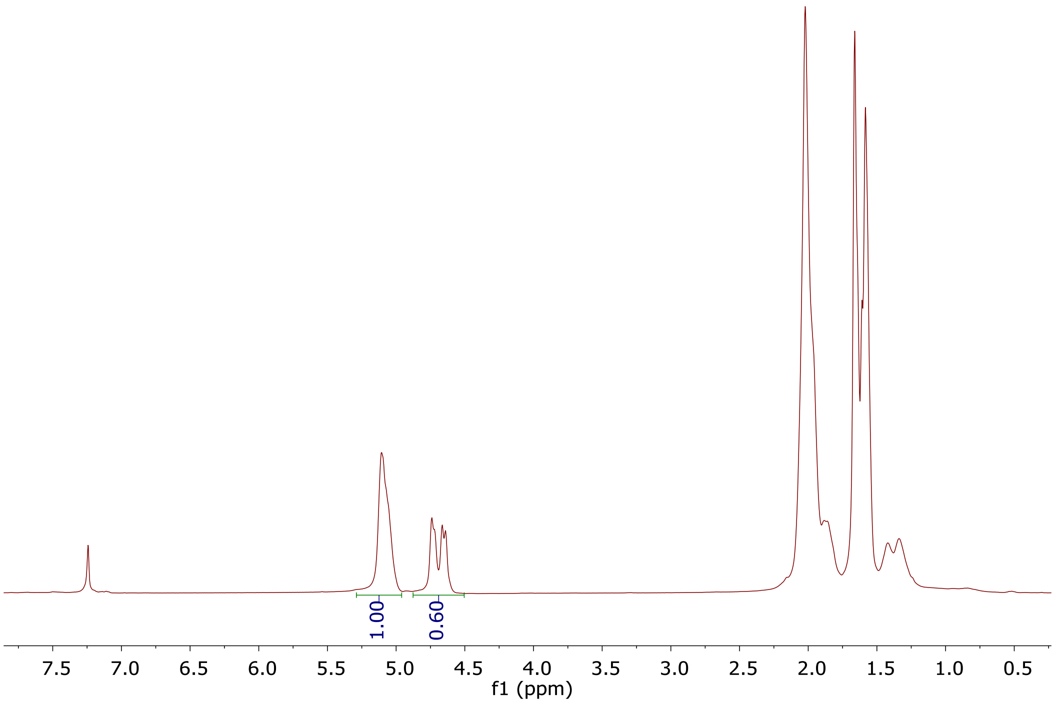

Fig. S74 ^{1}H NMR spectrum of PIP 500 equivalents generated by **Gd(CH$_2$SiMe$_3$)$_3$(THF)$_2$**, 1 equivalent PPh$_3$, and 2 equivalents [Ph$_3$C][B(C$_6$F$_5$)$_4$] from **Table 6**, entry 6 in CDCl$_3$ at 298 K ([Ph$_3$C][B(C$_6$F$_5$)$_4$] addition time 30 min).

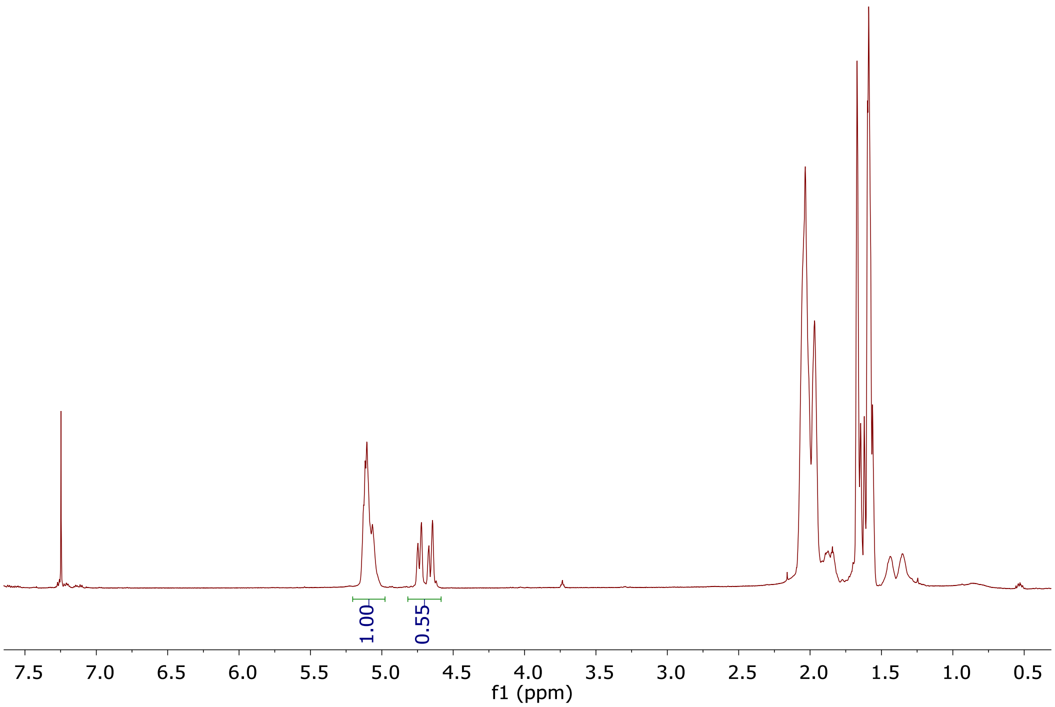

Fig. S75 ^{1}H NMR spectrum of PIP 500 equivalents generated by **$Y(CH_2SiMe_3)_3(THF)_2$**, 1 equivalent PPh_3, and 2 equivalents $[Ph_3C][B(C_6F_5)_4]$ from **Table 6**, entry 7 in $CDCl_3$ at 298 K ($[Ph_3C][B(C_6F_5)_4]$ addition time 0 min).

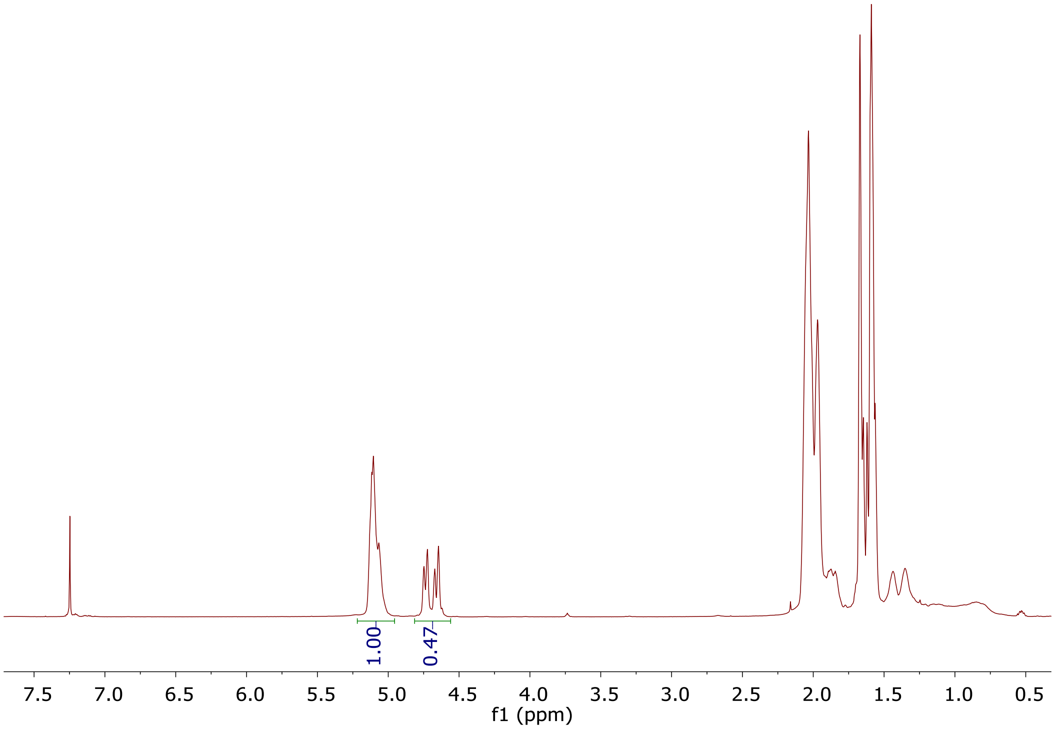

Fig. S76 ^{1}H NMR spectrum of PIP 500 equivalents generated by **$Y(CH_2SiMe_3)_3(THF)_2$**, 1 equivalent PPh_3, and 2 equivalents $[Ph_3C][B(C_6F_5)_4]$ from **Table 6**, entry 8 in $CDCl_3$ at 298 K ($[Ph_3C][B(C_6F_5)_4]$ addition time 10 min).

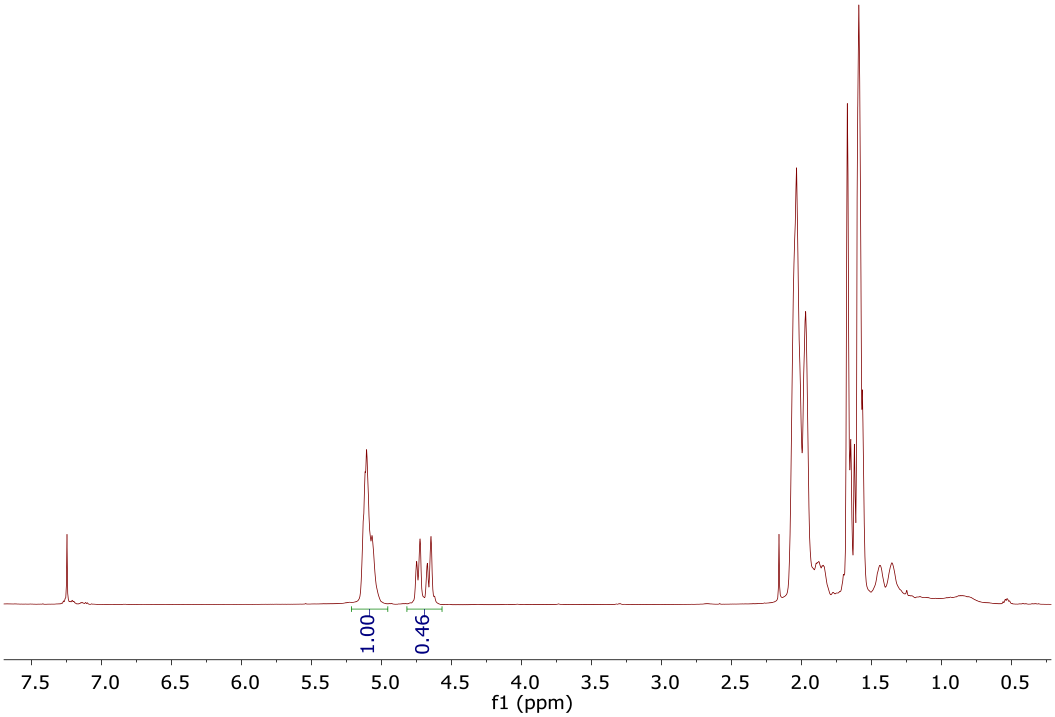

Fig. S77 ^{1}H NMR spectrum of PIP 500 equivalents generated by **$Y(CH_2SiMe_3)_3(THF)_2$**, 1 equivalent PPh_3, and 2 equivalents $[Ph_3C][B(C_6F_5)_4]$ from **Table 6**, entry 9 in $CDCl_3$ at 298 K ($[Ph_3C][B(C_6F_5)_4]$ addition time 30 min).

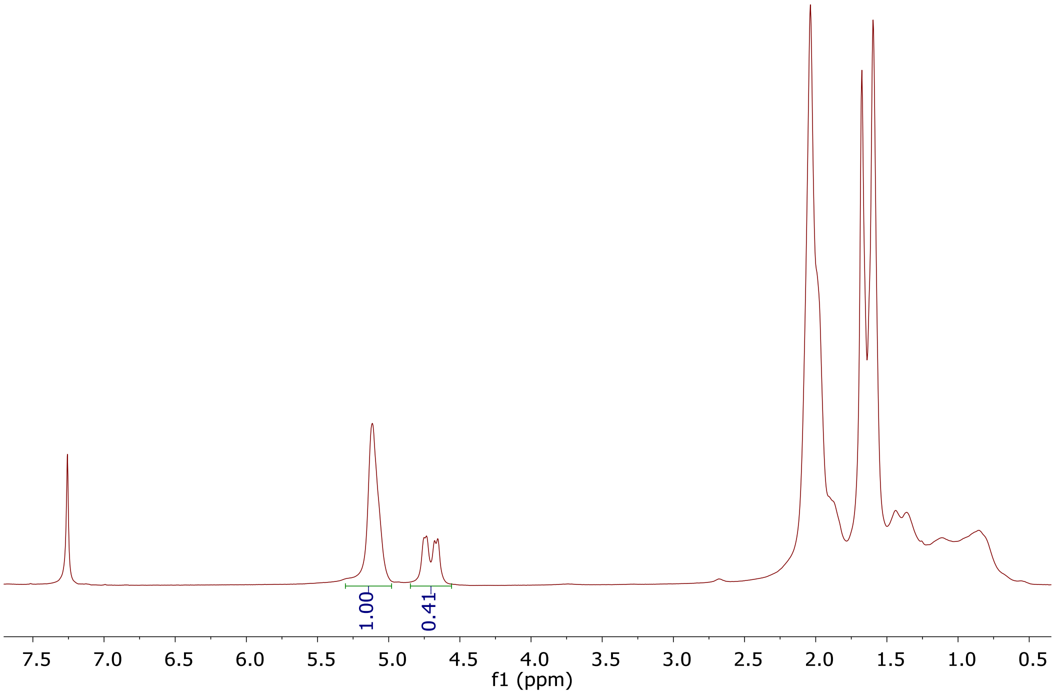

Fig. S78 ^{1}H NMR spectrum of PIP 500 equivalents generated by **$Tm(CH_2SiMe_3)_3(THF)_2$**, 1 equivalent PPh_3, and 2 equivalents $[Ph_3C][B(C_6F_5)_4]$ from **Table 6**, entry 10 in $CDCl_3$ at 298 K ($[Ph_3C][B(C_6F_5)_4]$ addition time 0 min).

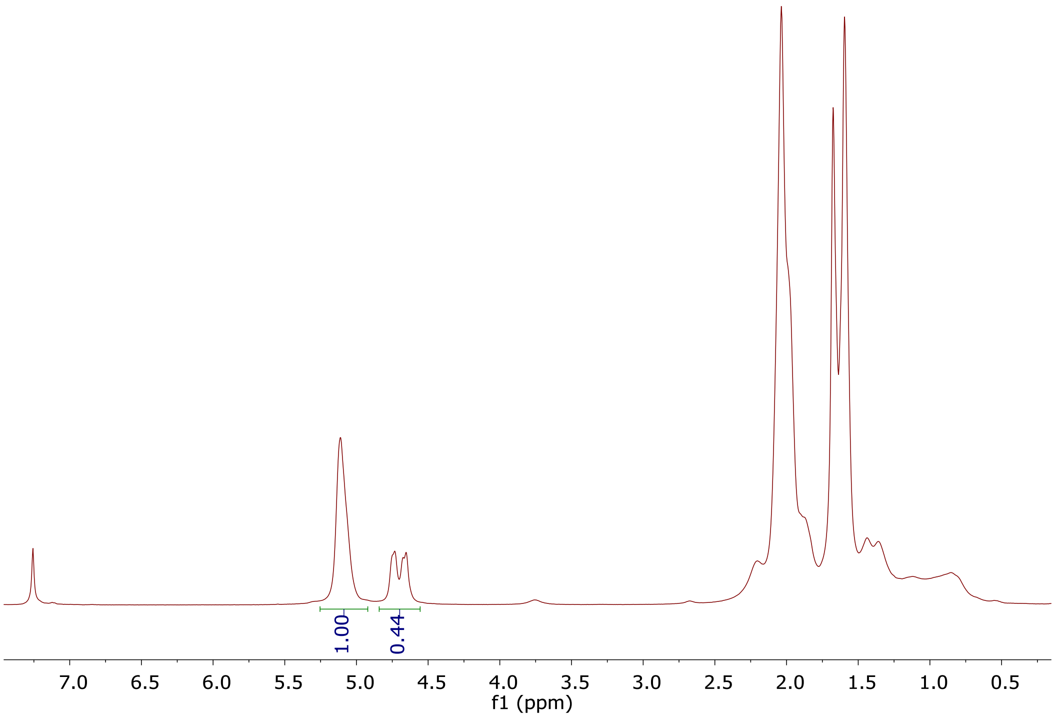

Fig. S79 ^{1}H NMR spectrum of PIP 500 equivalents generated by **$Tm(CH_2SiMe_3)_3(THF)_2$**, 1 equivalent PPh_3, and 2 equivalents $[Ph_3C][B(C_6F_5)_4]$ from **Table 6**, entry 11 in $CDCl_3$ at 298 K ($[Ph_3C][B(C_6F_5)_4]$ addition time 10 min).

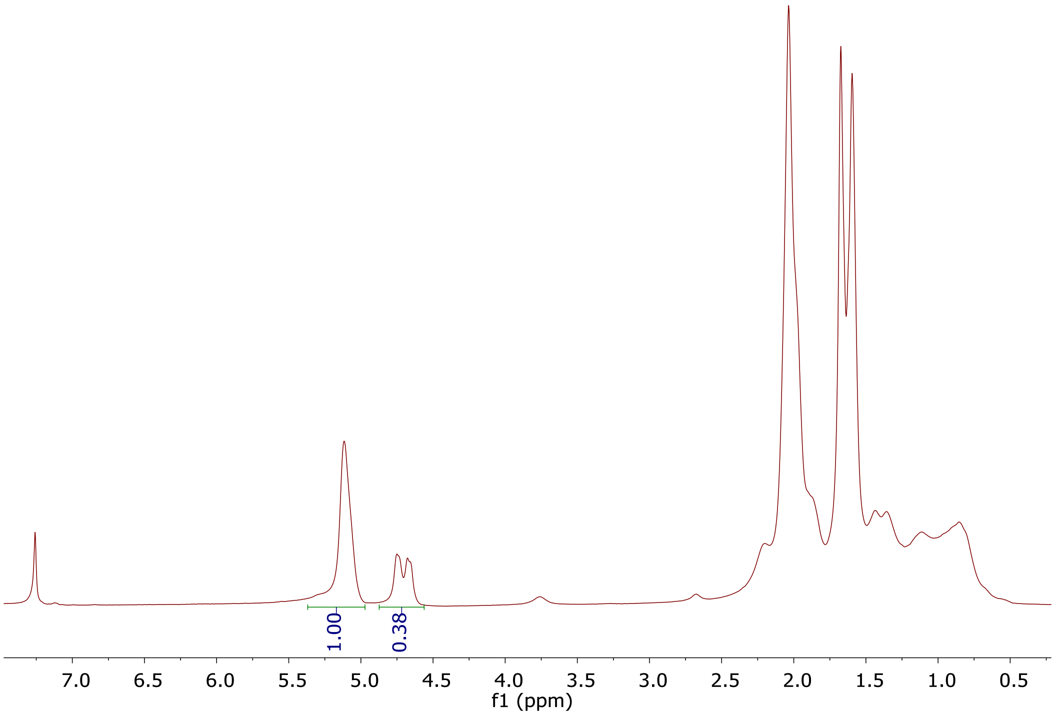

Fig. S80 ^{1}H NMR spectrum of PIP 500 equivalents generated by **$Tm(CH_2SiMe_3)_3(THF)_2$**, 1 equivalent PPh_3, and 2 equivalents $[Ph_3C][B(C_6F_5)_4]$ from **Table 6**, entry 12 in $CDCl_3$ at 298 K ($[Ph_3C][B(C_6F_5)_4]$ addition time 30 min).

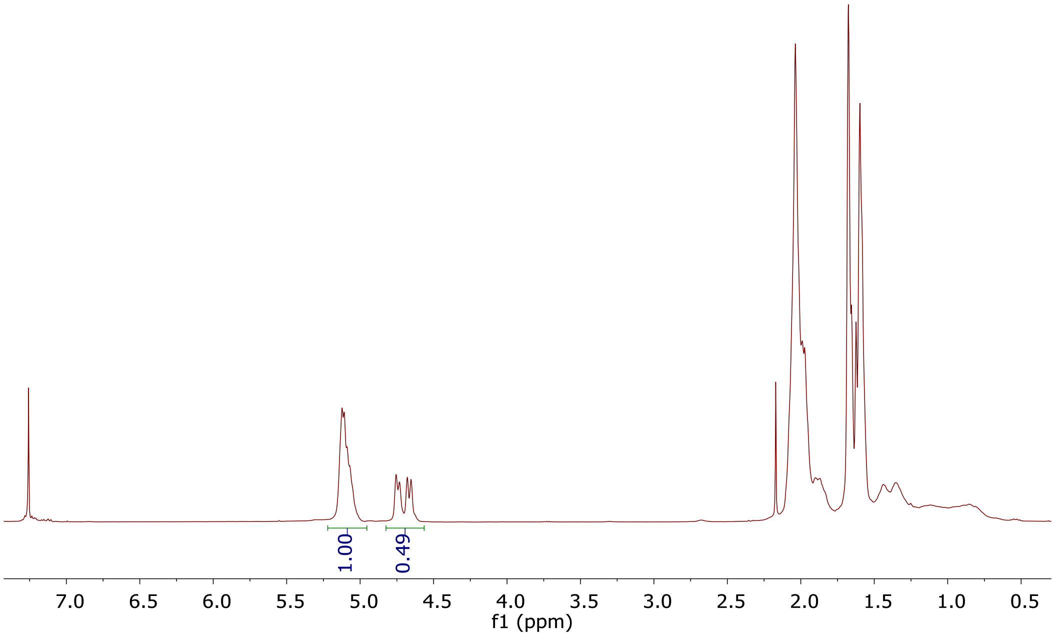

Fig. S81 ^{1}H NMR spectrum of PIP 500 equivalents generated by **$Sm(CH_2SiMe_3)_3(THF)_3$**, 2 equivalents $[Ph_3C][B(C_6F_5)_4]$, and 1 equivalent PPh_3 from **Table 7**, entry 1 in $CDCl_3$ at 298 K (PPh_3 addition time 0 min).

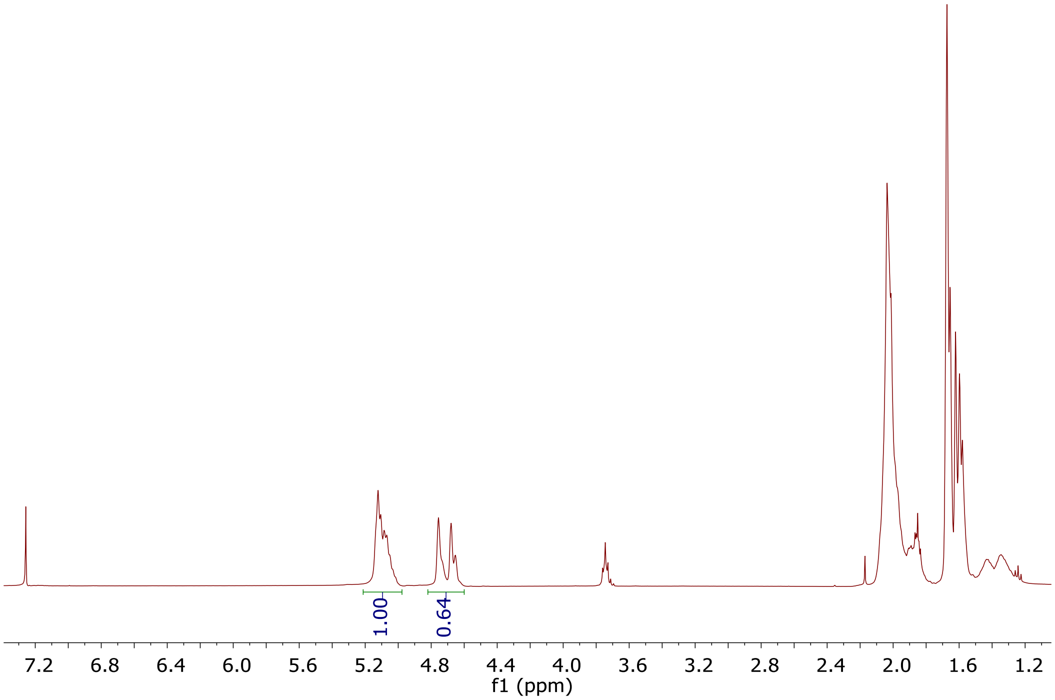

Fig. S82 ^{1}H NMR spectrum of PIP 500 equivalents generated by **$Sm(CH_2SiMe_3)_3(THF)_3$**, 2 equivalents $[Ph_3C][B(C_6F_5)_4]$, and 1 equivalent PPh_3 from **Table 7**, entry 2 in $CDCl_3$ at 298 K (PPh_3 addition time 10 min).

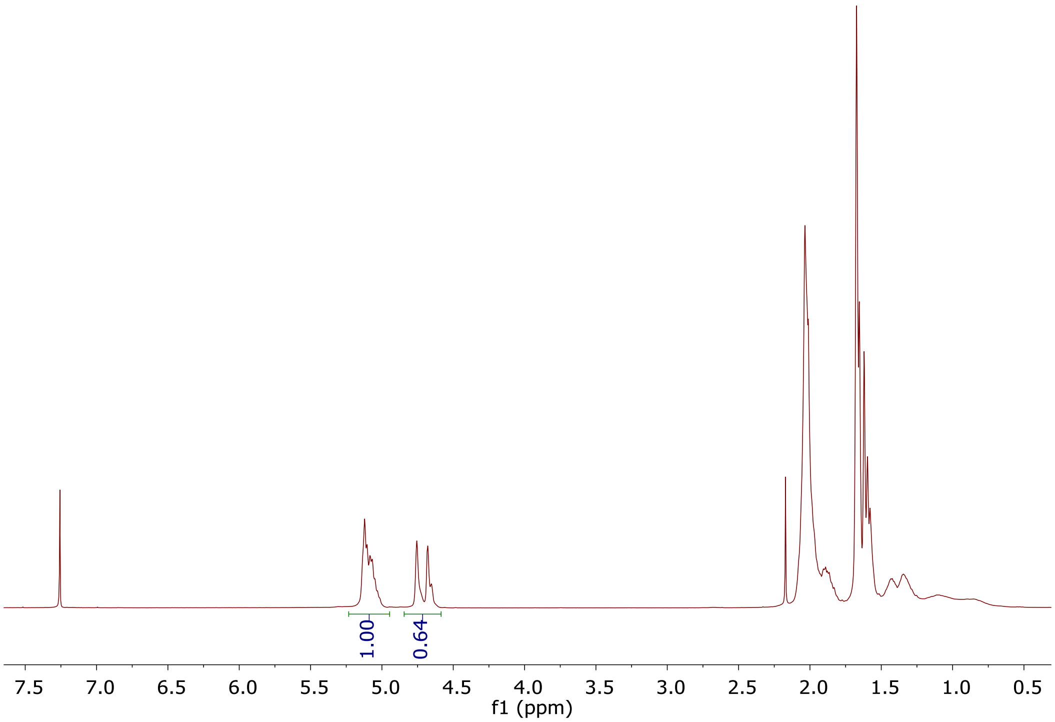

Fig. S83 ^{1}H NMR spectrum of PIP 500 equivalents generated by **$Sm(CH_2SiMe_3)_3(THF)_3$**, 2 equivalents $[Ph_3C][B(C_6F_5)_4]$, and 1 equivalent PPh_3 from **Table 7**, entry 3 in $CDCl_3$ at 298 K (PPh_3 addition time 30 min).

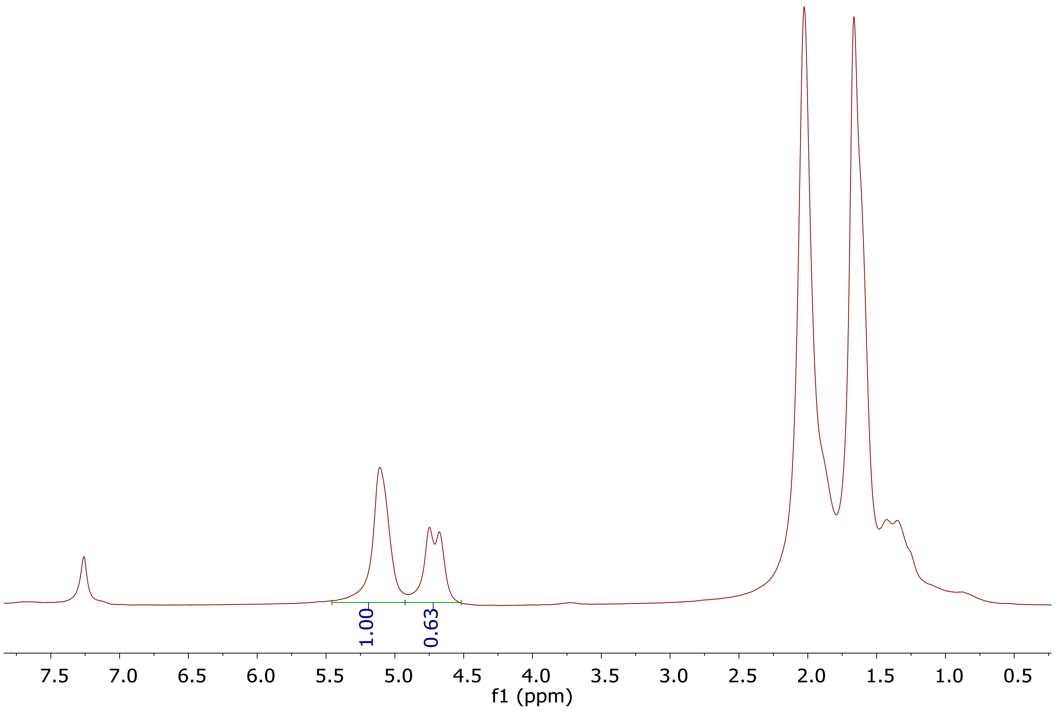

Fig. S84 ^{1}H NMR spectrum of PIP 500 equivalents generated by **$Gd(CH_2SiMe_3)_3(THF)_2$**, 2 equivalents $[Ph_3C][B(C_6F_5)_4]$, and 1 equivalent PPh_3 from **Table 7**, entry 4 in $CDCl_3$ at 298 K (PPh_3 addition time 0 min).

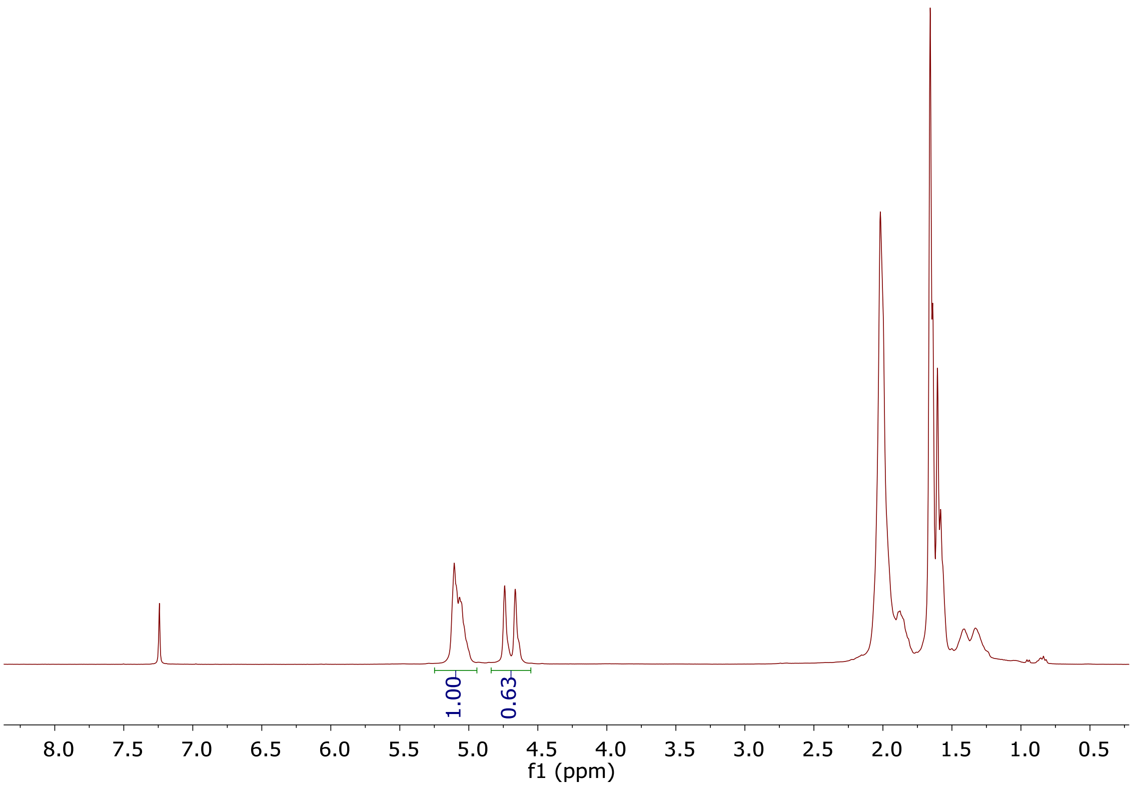

Fig. S85 ^{1}H NMR spectrum of PIP 500 equivalents generated by **Gd(CH$_2$SiMe$_3$)$_3$(THF)$_2$**, 2 equivalents [Ph$_3$C][B(C$_6$F$_5$)$_4$], and 1 equivalent PPh$_3$ from **Table 7**, entry 5 in CDCl$_3$ at 298 K (PPh$_3$ addition time 10 min).

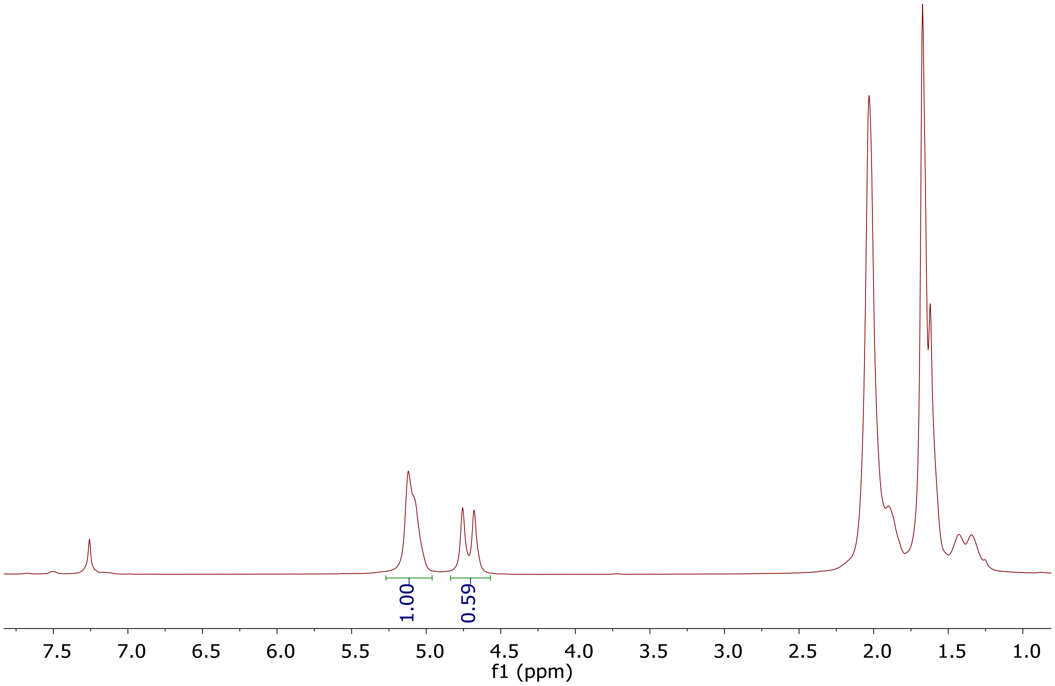

Fig. S86 ^{1}H NMR spectrum of PIP 500 equivalents generated by **Gd(CH$_2$SiMe$_3$)$_3$(THF)$_2$**, 2 equivalents [Ph$_3$C][B(C$_6$F$_5$)$_4$], and 1 equivalent PPh$_3$ from **Table 7**, entry 6 in CDCl$_3$ at 298 K (PPh$_3$ addition time 30 min).

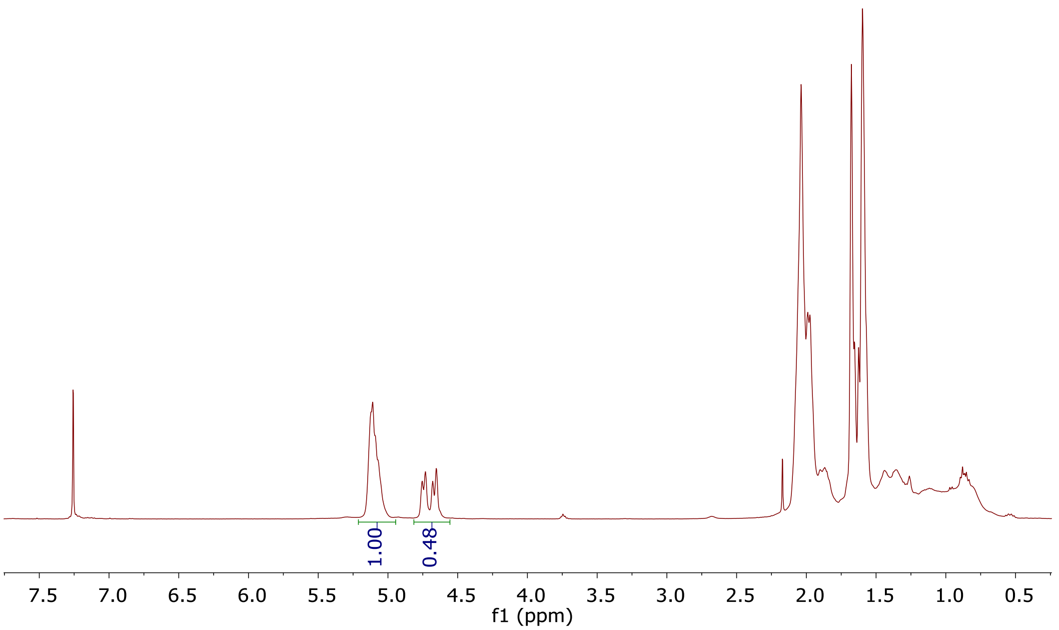

Fig. S87 ^{1}H NMR spectrum of PIP 500 equivalents generated by **$Y(CH_2SiMe_3)_3(THF)_2$**, 2 equivalents $[Ph_3C][B(C_6F_5)_4]$, and 1 equivalent PPh_3 from **Table 7**, entry 7 in $CDCl_3$ at 298 K (PPh_3 addition time 0 min).

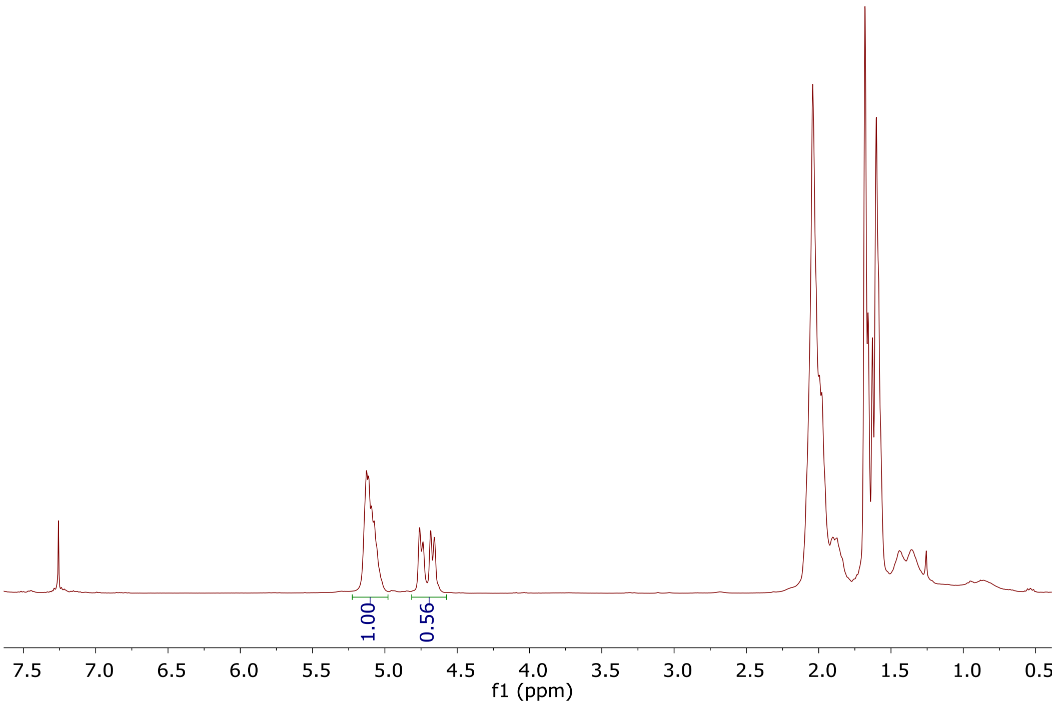

Fig. S88 ^{1}H NMR spectrum of PIP 500 equivalents generated by **$Y(CH_2SiMe_3)_3(THF)_2$**, 2 equivalents $[Ph_3C][B(C_6F_5)_4]$, and 1 equivalent PPh_3 from **Table 7**, entry 8 in $CDCl_3$ at 298 K (PPh_3 addition time 10 min).

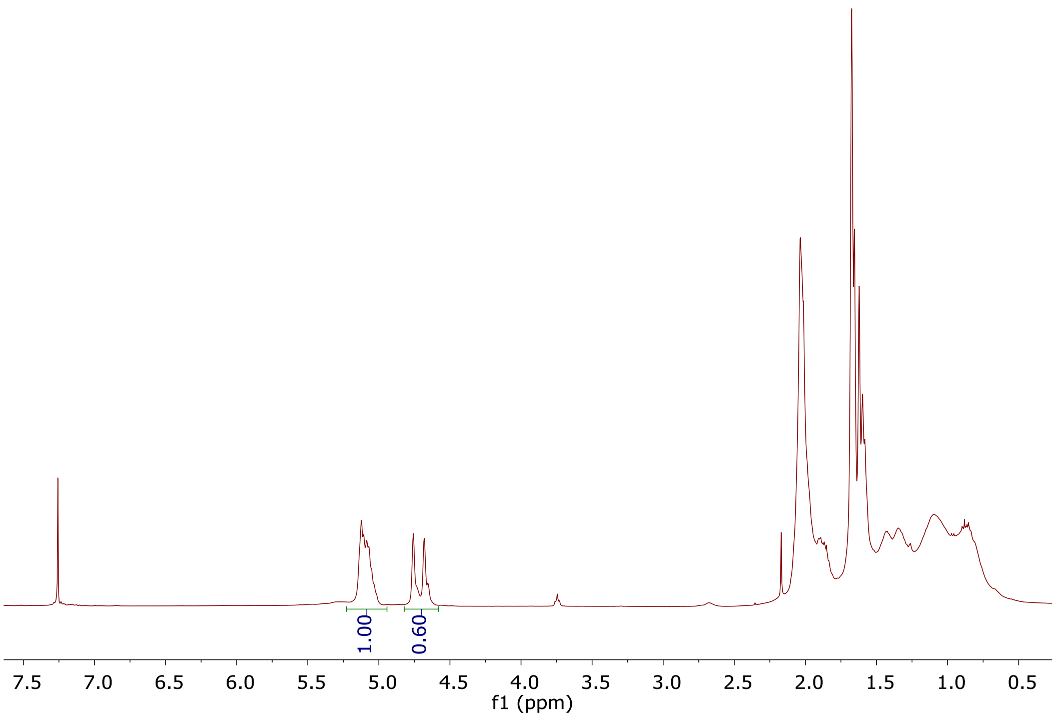

Fig. S89 ^{1}H NMR spectrum of PIP 500 equivalents generated by **$Y(CH_2SiMe_3)_3(THF)_2$**, 2 equivalents $[Ph_3C][B(C_6F_5)_4]$, and 1 equivalent PPh_3 from **Table 7**, entry 9 in $CDCl_3$ at 298 K (PPh_3 addition time 30 min).

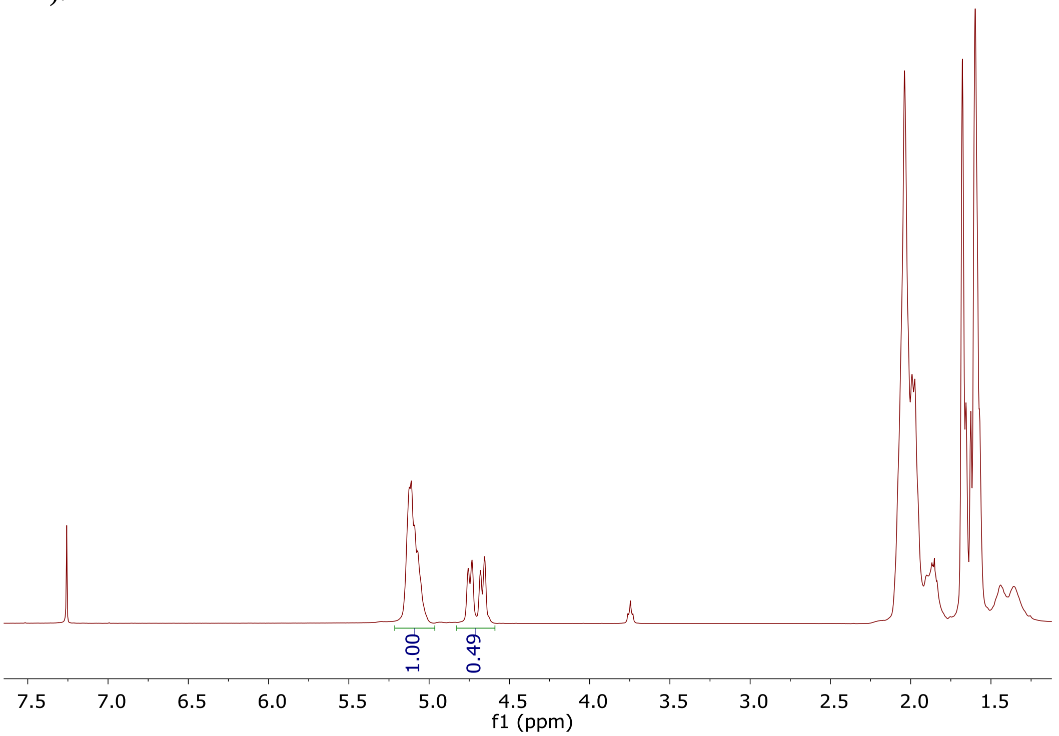

Fig. S90 ^{1}H NMR spectrum of PIP 500 equivalents generated by **$Tm(CH_2SiMe_3)_3(THF)_2$**, 2 equivalents $[Ph_3C][B(C_6F_5)_4]$, and 1 equivalent PPh_3 from **Table 7**, entry 10 in $CDCl_3$ at 298 K (PPh_3 addition time 0 min).

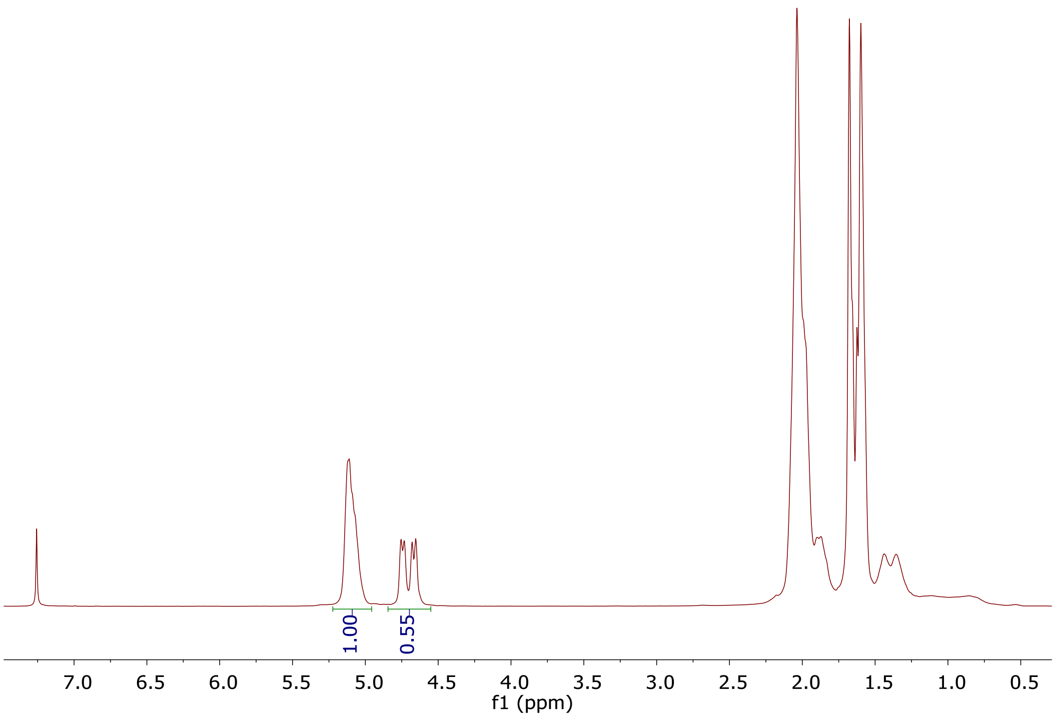

Fig. S91 ^{1}H NMR spectrum of PIP 500 equivalents generated by **$Tm(CH_2SiMe_3)_3(THF)_2$**, 2 equivalents $[Ph_3C][B(C_6F_5)_4]$, and 1 equivalent PPh_3 from **Table 7**, entry 11 in $CDCl_3$ at 298 K (PPh_3 addition time 10 min).

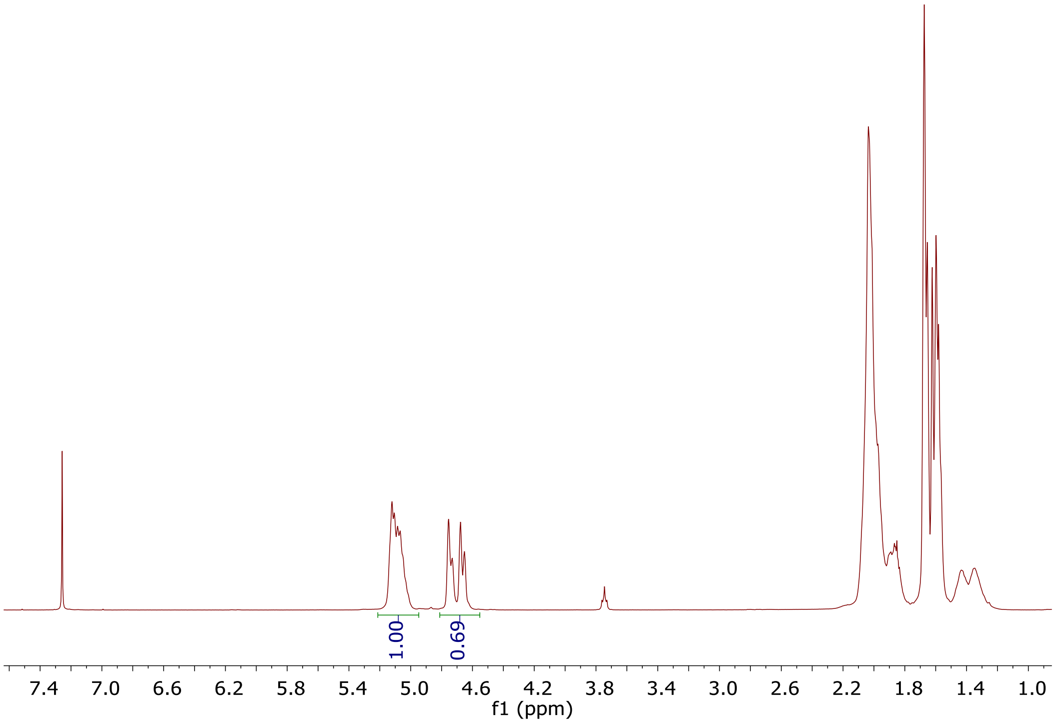

Fig. S92 ^{1}H NMR spectrum of PIP 500 equivalents generated by **$Tm(CH_2SiMe_3)_3(THF)_2$**, 2 equivalents $[Ph_3C][B(C_6F_5)_4]$, and 1 equivalent PPh_3 from **Table 7**, entry 12 in $CDCl_3$ at 298 K (PPh_3 addition time 30 min).

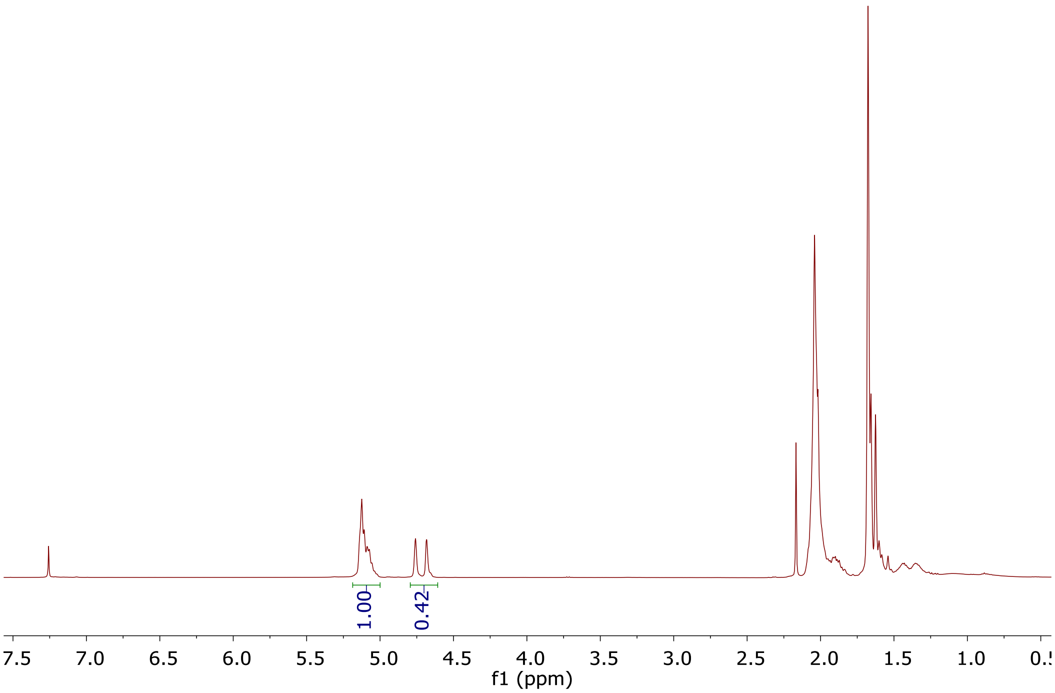

Fig. S93 ^{1}H NMR spectrum of PIP 500 equivalents generated by **$Y(CH_2SiMe_3)_3(THF)_2$** and 2 equivalents $[Ph_3C][B(C_6F_5)_4]$ from **Table 7**, entry 13 in $CDCl_3$ at 298 K (IP addition time 10 min).

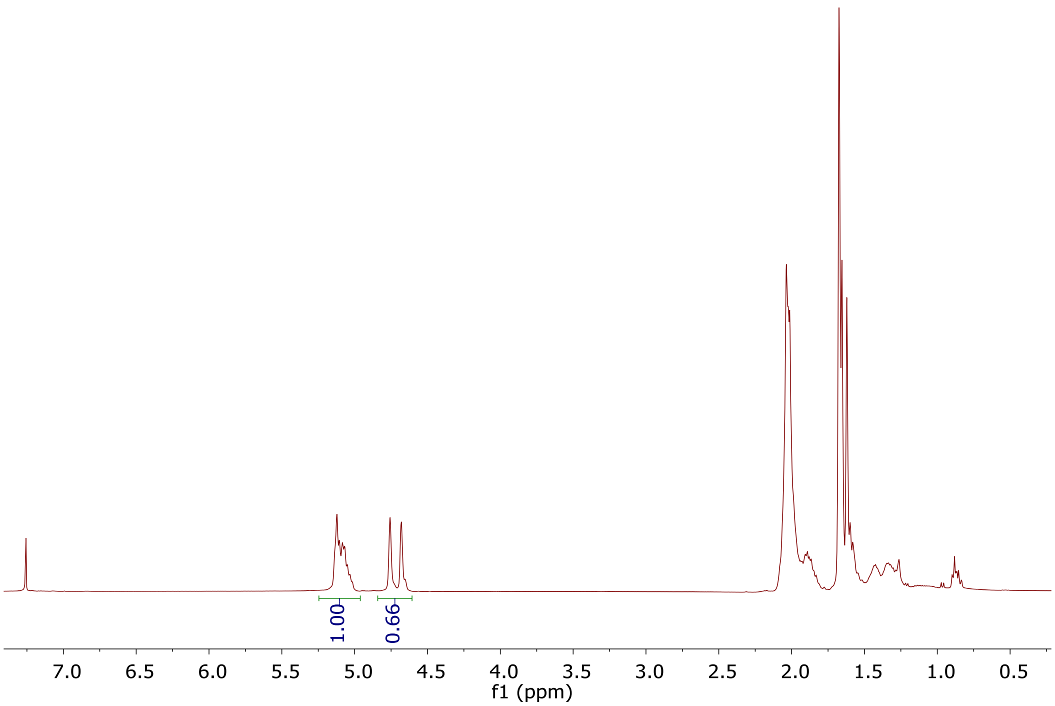

Fig. S94 ^{1}H NMR spectrum of PIP 500 equivalents generated by **$Y(CH_2SiMe_3)_3(THF)_2$** and 2 equivalents $[Ph_3C][B(C_6F_5)_4]$ from **Table 7**, entry 14 in $CDCl_3$ at 298 K (IP addition time 20 min).

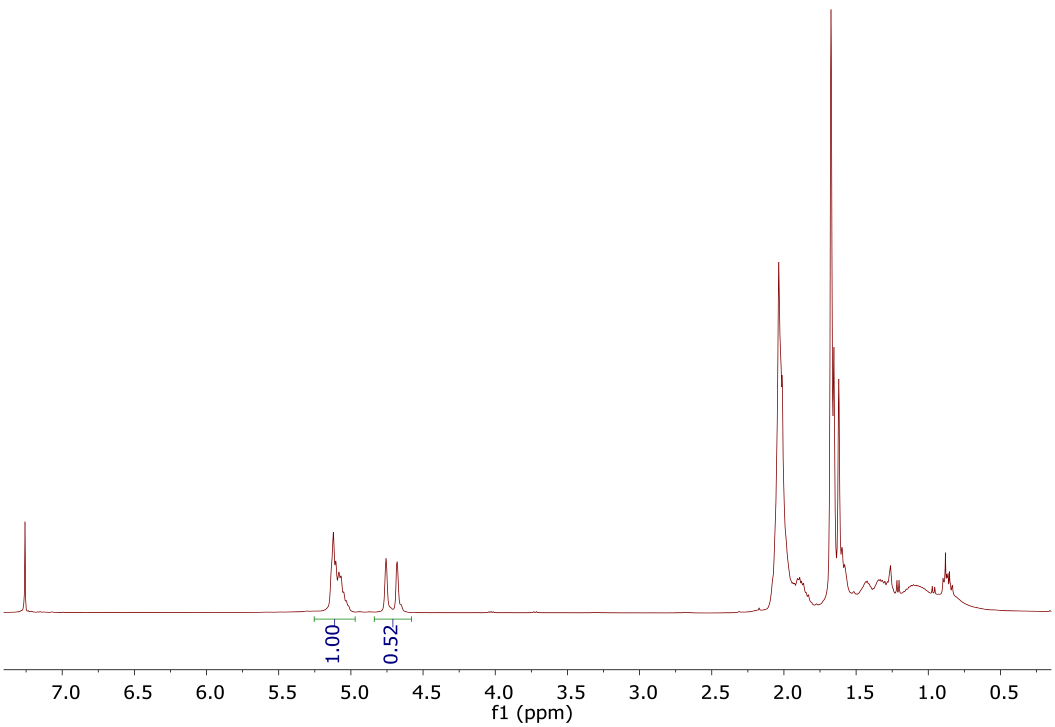

Fig. S95 ^{1}H NMR spectrum of PIP 500 equivalents generated by **$Y(CH_2SiMe_3)_3(THF)_2$** and 2 equivalents $[Ph_3C][B(C_6F_5)_4]$ from **Table 7**, entry 15 in $CDCl_3$ at 298 K (IP addition time 40 min).

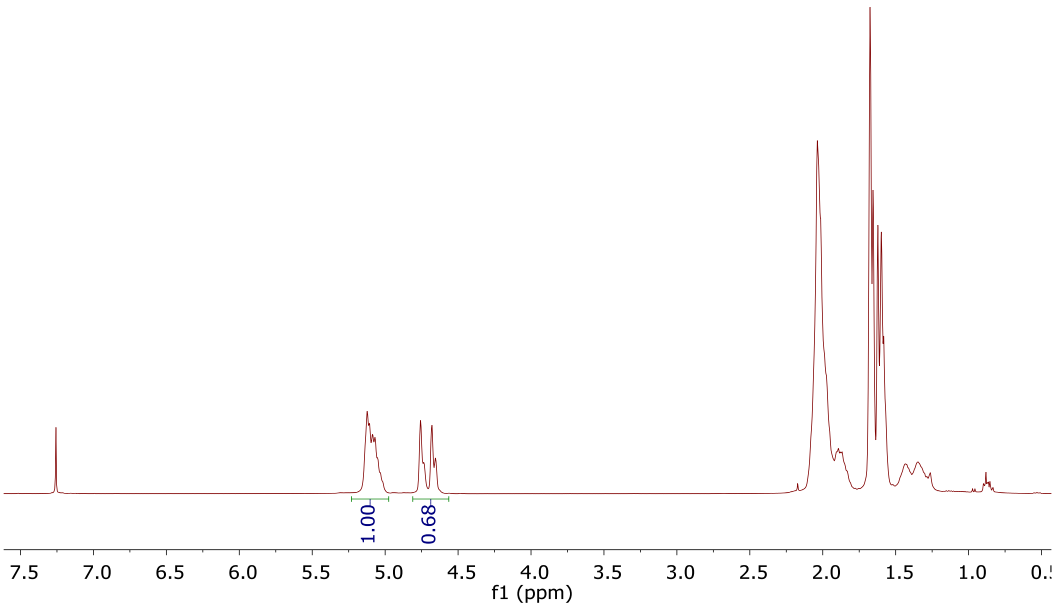

Fig. S96 ^{1}H NMR spectrum of PIP 500 equivalents generated by **$Y(CH_2SiMe_3)_3(THF)_2$**, 2 equivalents $[Ph_3C][B(C_6F_5)_4]$, and 1 equivalent PPh_3 from **Table 8**, entry 1 (Step 1: 60 min).

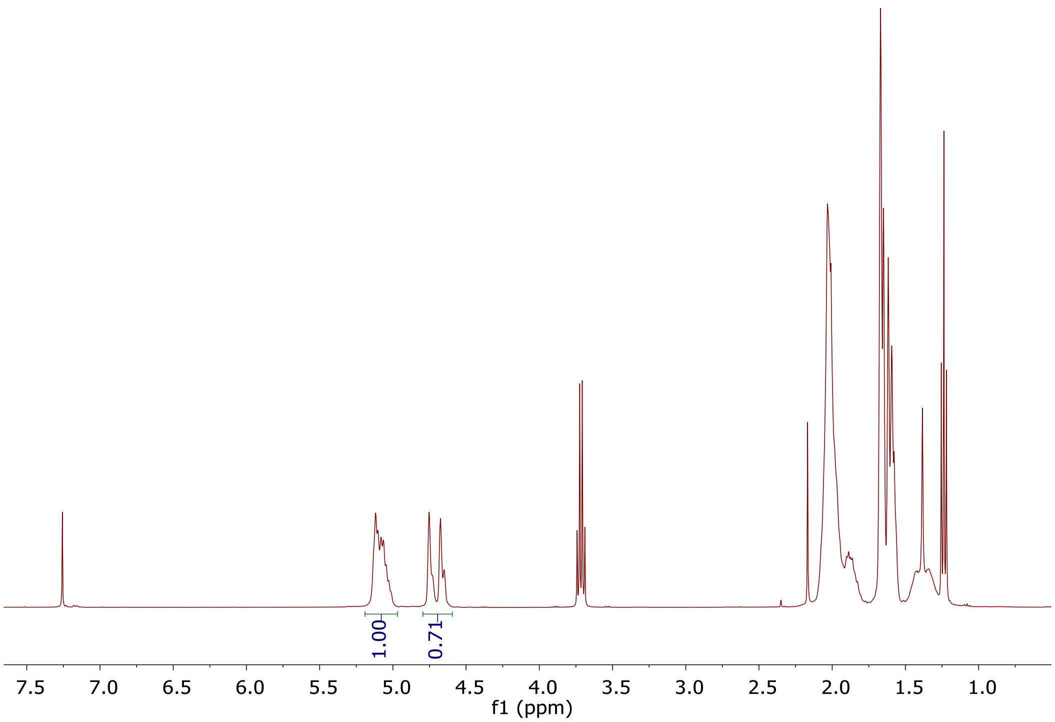

Fig. S97 ^{1}H NMR spectrum of PIP 500 equivalents generated by **$Y(CH_2SiMe_3)_3(THF)_2$**, 2 equivalents $[Ph_3C][B(C_6F_5)_4]$, and 1 equivalent PPh_3 from **Table 8**, entry 2 (Step 2: 60 min).

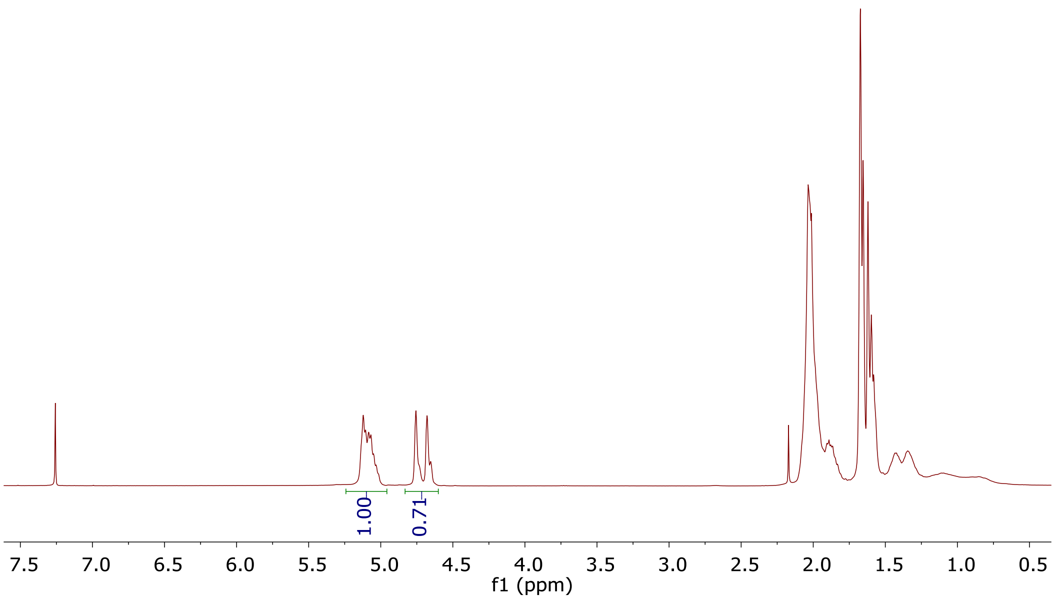

Fig. S98 ^{1}H NMR spectrum of PIP 500 equivalents generated by **$Y(CH_2SiMe_3)_3(THF)_2$**, 2 equivalents $[Ph_3C][B(C_6F_5)_4]$, and 1 equivalent PPh_3 from **Table 8**, entry 3 (Step 3: 60 min).

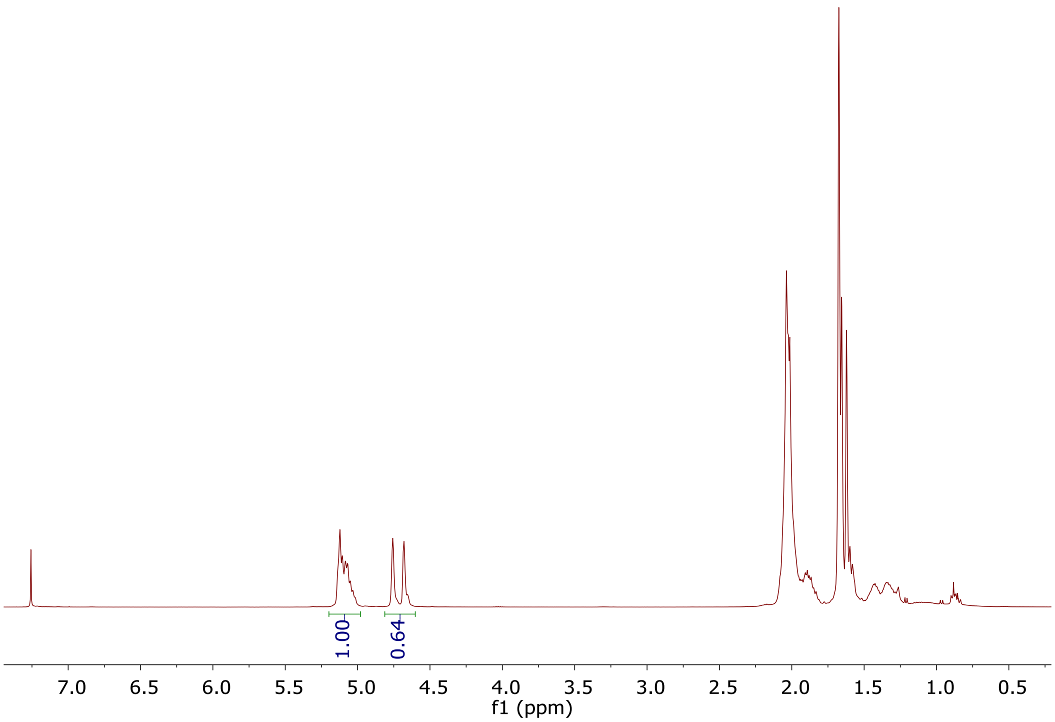

Fig. S99 ^{1}H NMR spectrum of PIP 500 equivalents generated by **$Y(CH_2SiMe_3)_3(THF)_2$** and 2 equivalents $[Ph_3C][B(C_6F_5)_4]$ from **Table 8**, entry 4 (Step 1: 60 min).

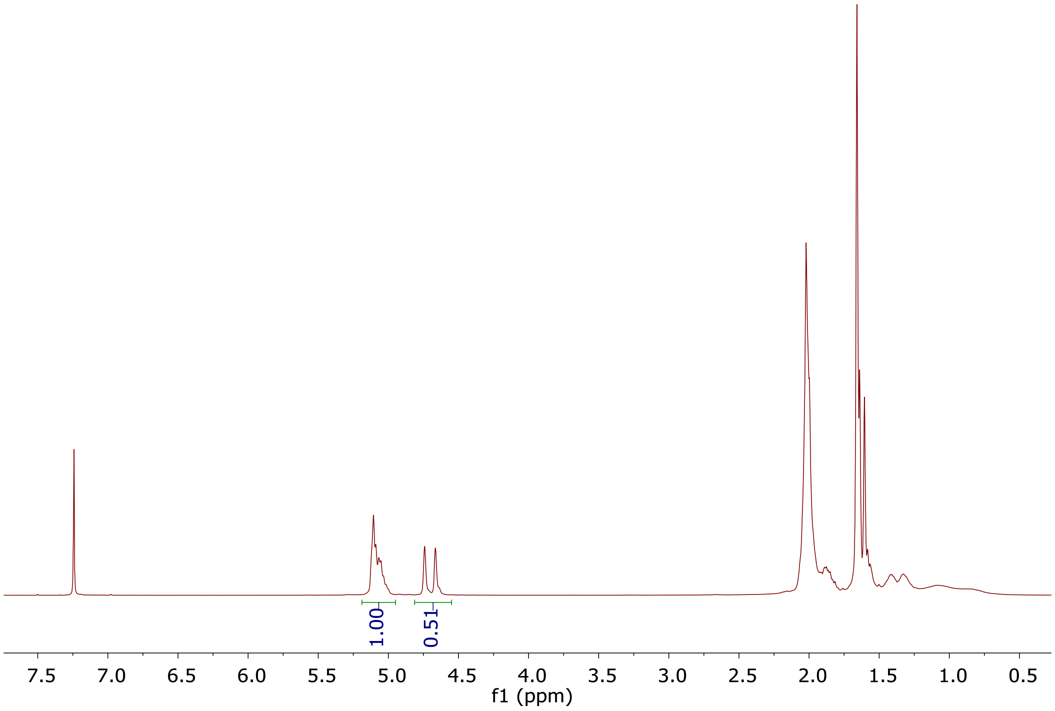

Fig. S100 ^{1}H NMR spectrum of PIP 500 equivalents generated by **$Y(CH_2SiMe_3)_3(THF)_2$** and 2 equivalents $[Ph_3C][B(C_6F_5)_4]$ from **Table 8**, entry 5 (Step 2: 60 min).

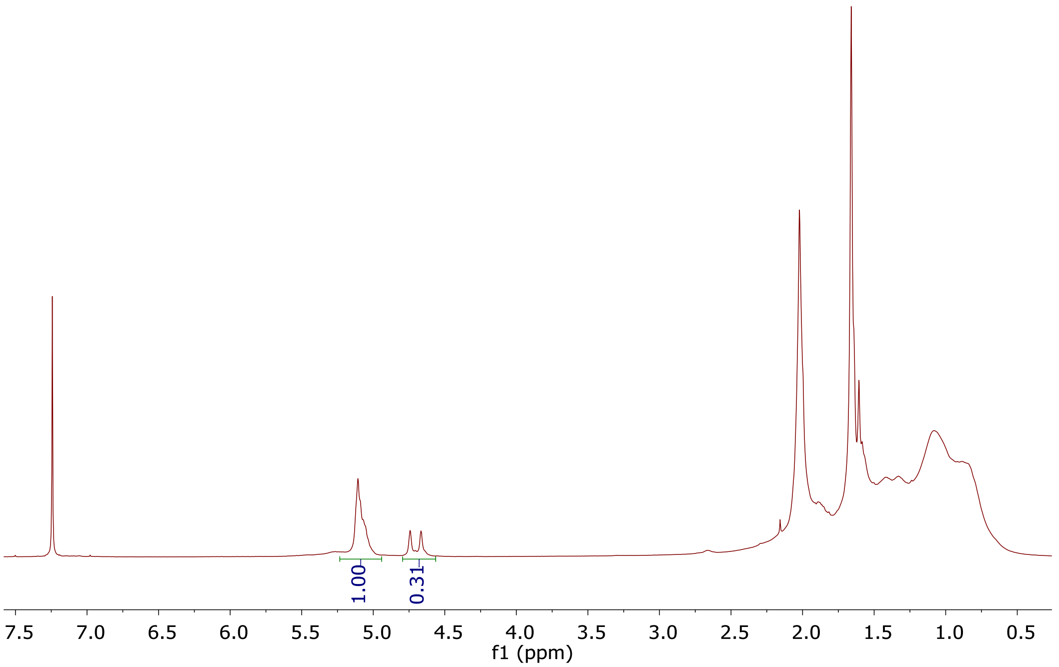

Fig. S101 ^{1}H NMR spectrum of PIP 500 equivalents generated by **$Y(CH_2SiMe_3)_3(THF)_2$** and 2 equivalents $[Ph_3C][B(C_6F_5)_4]$ from **Table 8**, entry 6 (Step 3: 60 min).

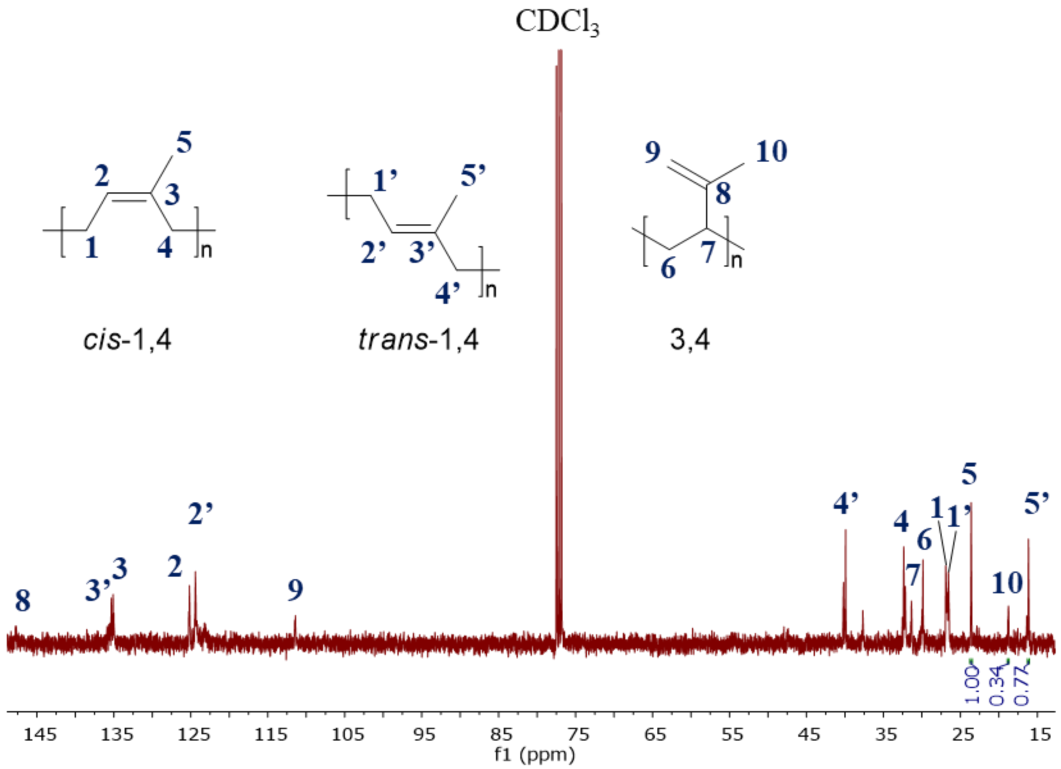

Fig. S102 ^{13}C NMR spectrum of PIP 500 equivalents generated by **$Y(CH_2SiMe_3)_3(THF)_2$** 1 equivalent $[Ph_3C][B(C_6F_5)_4]$ from **Table 1**, entry 1 in $CDCl_3$ at 298 K (30 min). Peak 5 (23.8 ppm) was used to calculate *cis*-1,4 content and peak 5' (16.2) was used to calculate *trans*-1,4 content.

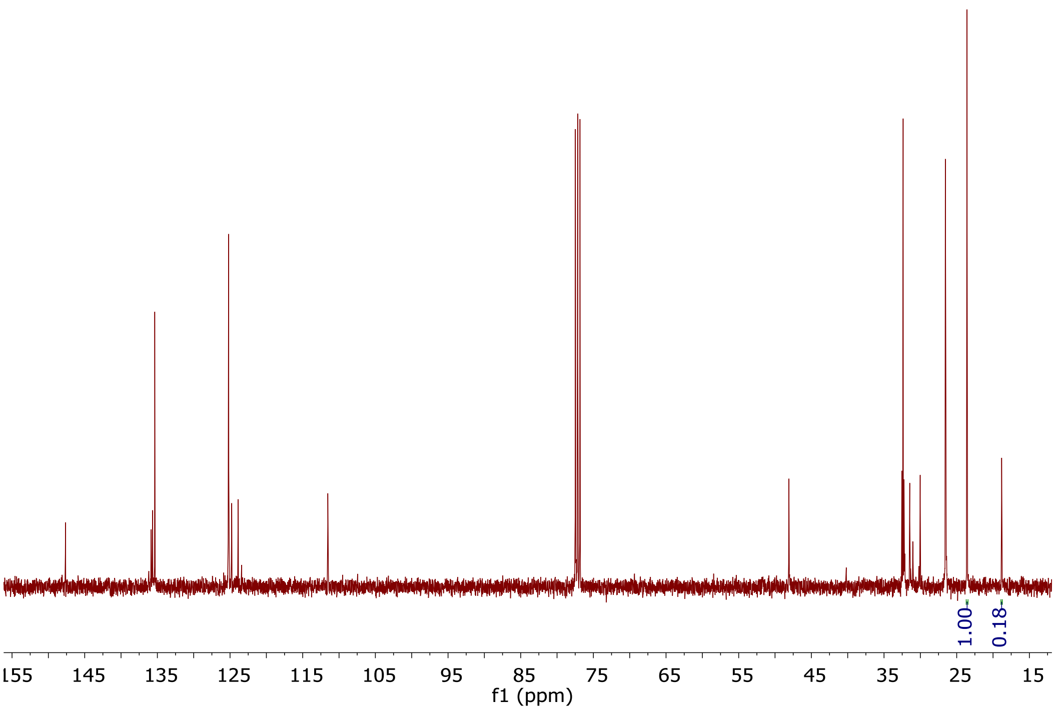

Fig. S103 ^{13}C NMR spectrum of PIP 500 equivalents generated by **$Y(CH_2SiMe_3)_3(THF)_2$** and 2 equivalents $[Ph_3C][B(C_6F_5)_4]$ from **Table 1**, entry 2 in $CDCl_3$ at 298 K (30 min).

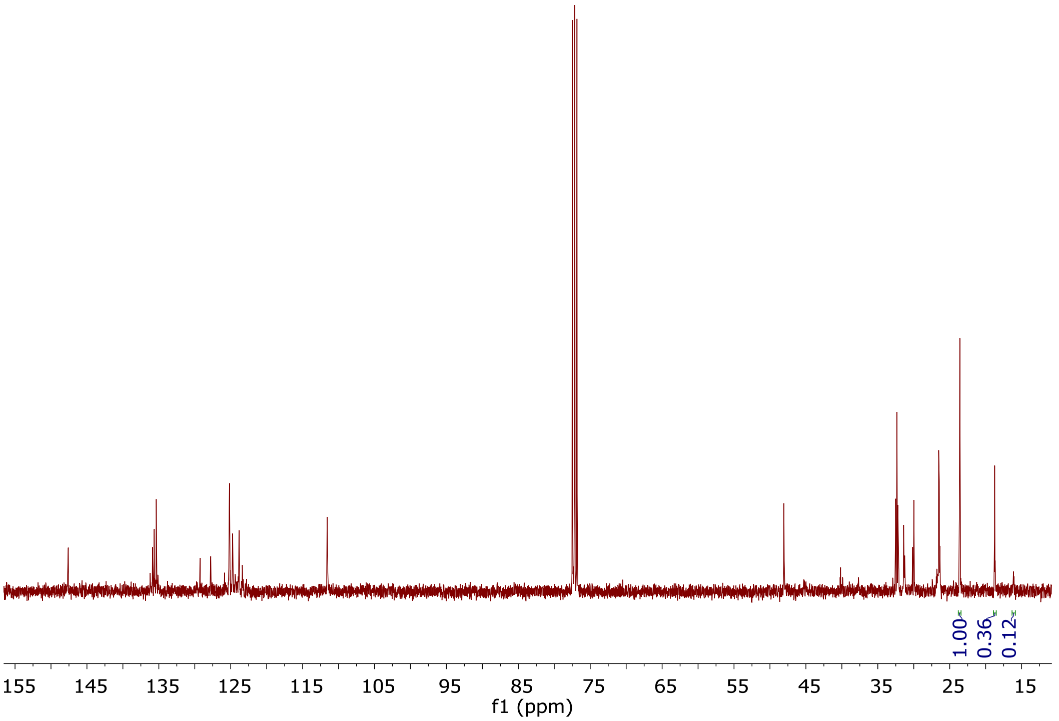

Fig. S104 ^{13}C NMR spectrum of PIP 500 equivalents generated by **$Y(CH_2SiMe_3)_3(THF)_2$**, 2 equivalents $[Ph_3C][B(C_6F_5)_4]$, and 1 equivalent Bipy from **Table 2**, entry 3 in $CDCl_3$ at 298 K (30 min).

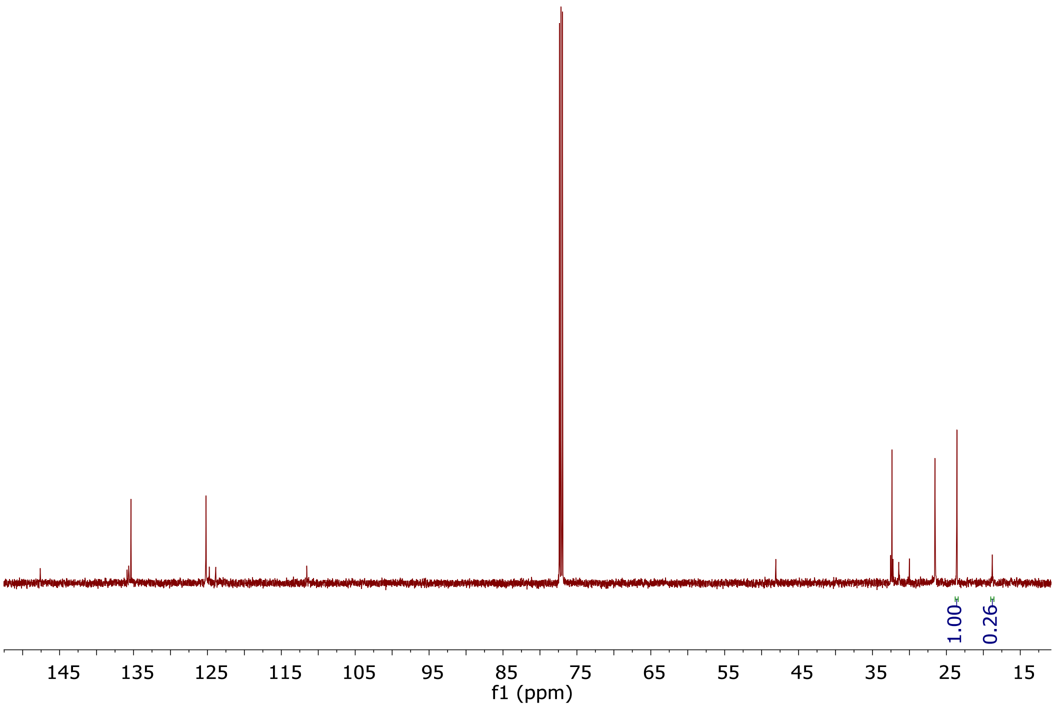

Fig. S105 ^{13}C NMR spectrum of PIP 500 equivalents generated by **$Y(CH_2SiMe_3)_3(THF)_2$**, 2 equivalents $[Ph_3C][B(C_6F_5)_4]$, and 1 equivalent MeCN from **Table 2**, entry 5 in $CDCl_3$ at 298 K (30 min).

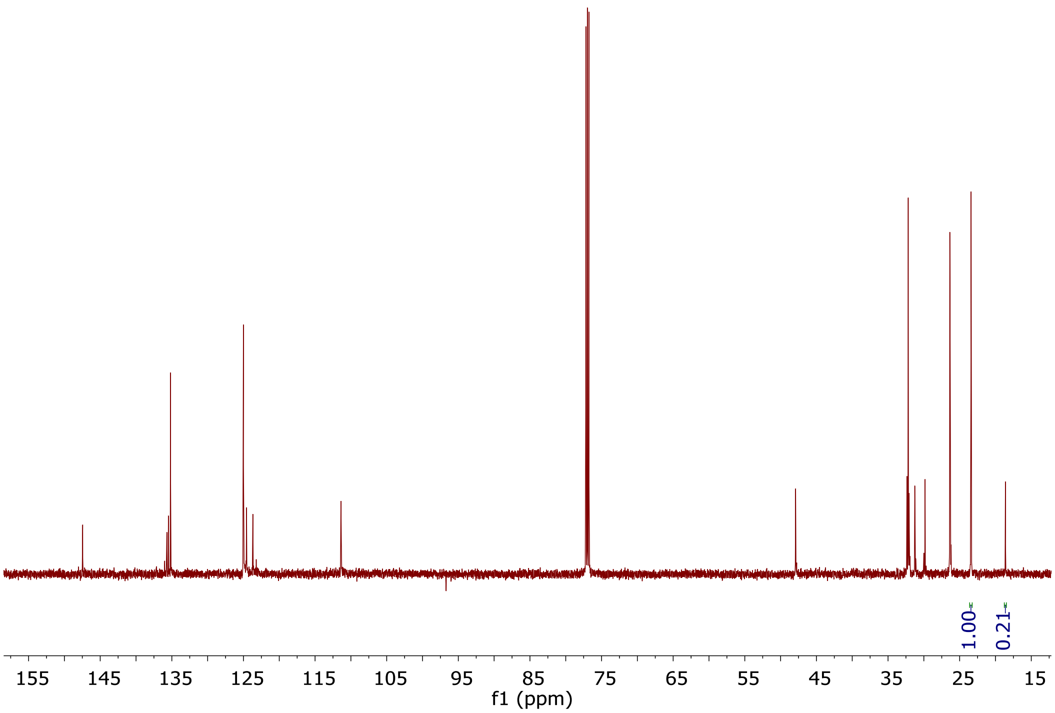

Fig. S106 ^{13}C NMR spectrum of PIP 500 equivalents generated by **$Y(CH_2SiMe_3)_3(THF)_2$**, 2 equivalents $[Ph_3C][B(C_6F_5)_4]$, and 1 equivalent P(*o*-tolyl)$_3$ from **Table 2**, entry 6 in $CDCl_3$ at 298 K (30 min).

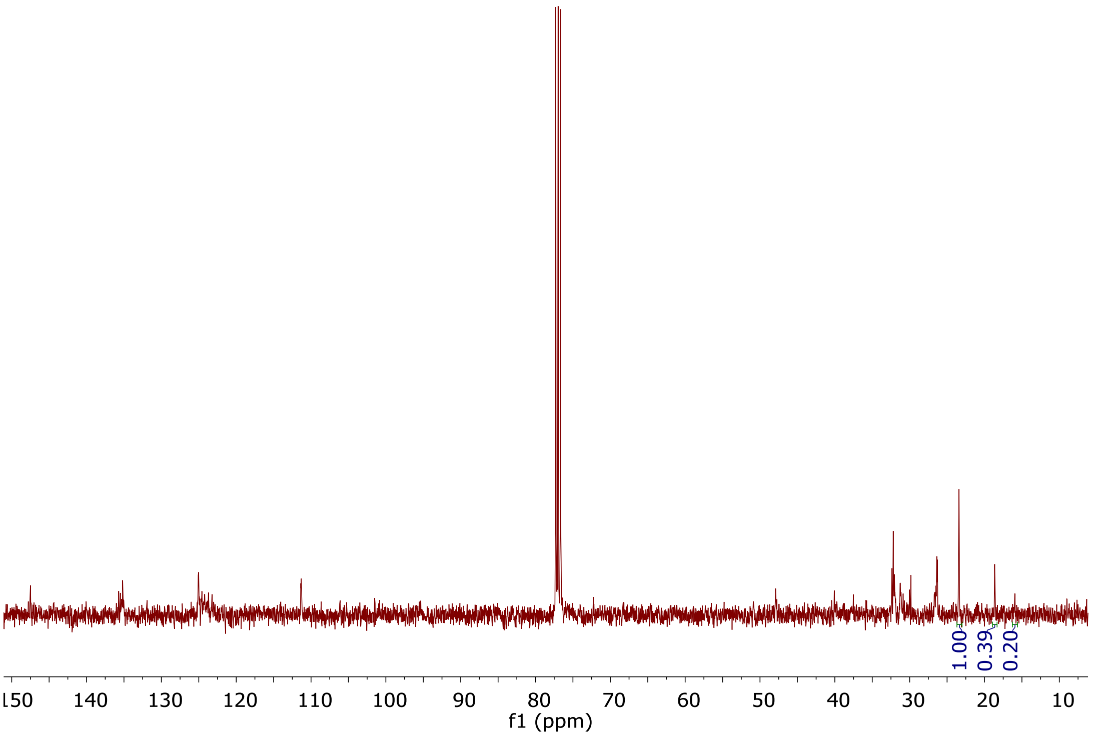

Fig. S107 ^{13}C NMR spectrum of PIP 500 equivalents generated by **Y(CH$_2$SiMe$_3$)$_3$(THF)$_2$**, 2 equivalents [Ph$_3$C][B(C$_6$F$_5$)$_4$], and 1 equivalent PCy$_3$ from **Table 2**, entry 7 in CDCl$_3$ at 298 K (30 min).

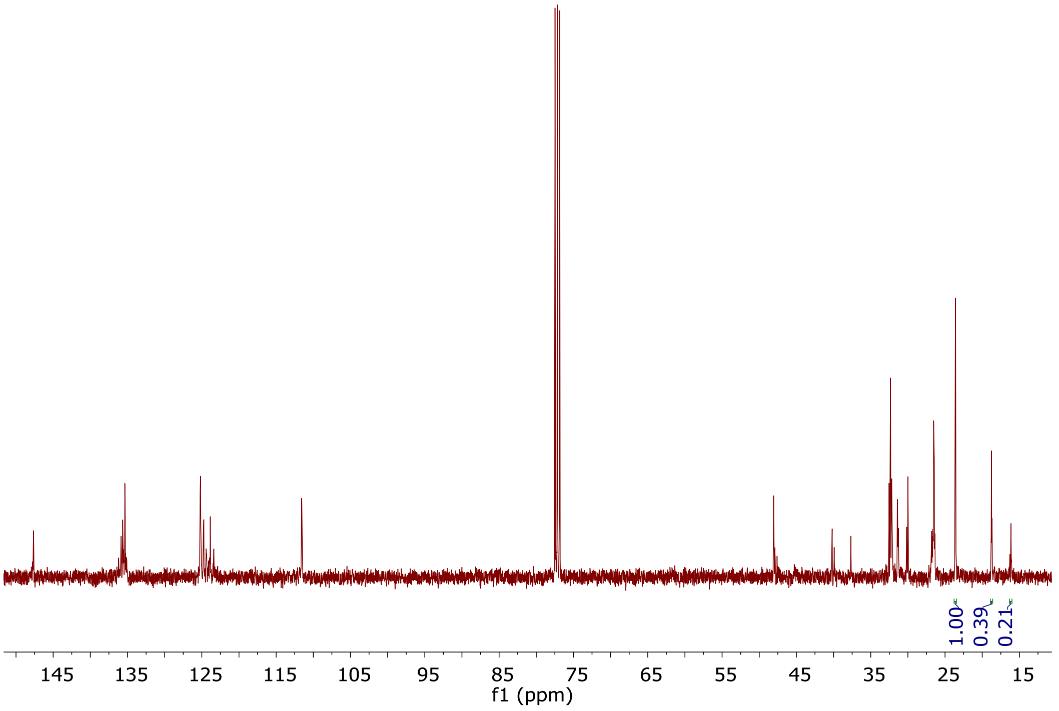

Fig. S108 ^{13}C NMR spectrum of PIP 500 equivalents generated by **Y(CH$_2$SiMe$_3$)$_3$(THF)$_2$**, 2 equivalents [Ph$_3$C][B(C$_6$F$_5$)$_4$], and 1 PPh$_3$ from **Table 2**, entry 8 in CDCl$_3$ at 298 K (30 min).

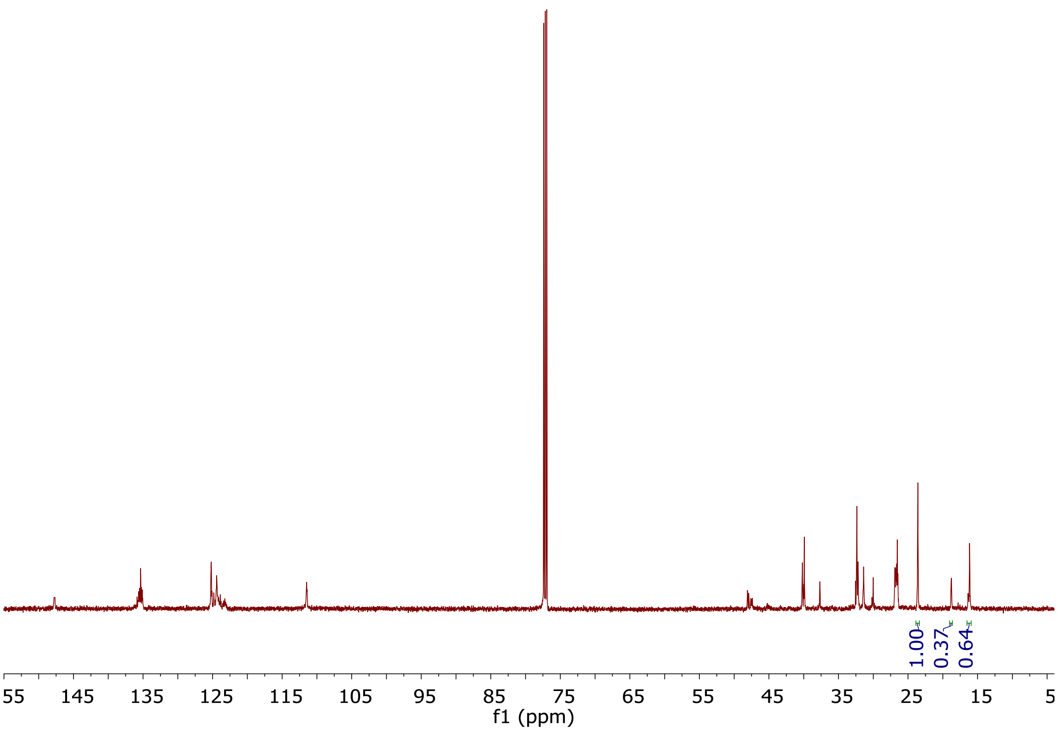

Fig. S109 ^{13}C NMR spectrum of PIP 500 equivalents generated by $\mathbf{Y(CH_2SiMe_3)_3(THF)_2}$, 2 equivalents $[Ph_3C][B(C_6F_5)_4]$, and 1 equivalent P(Ph-*p*-OMe)$_3$ from **Table S1**, entry 1 in $CDCl_3$ at 298 K (30 min).

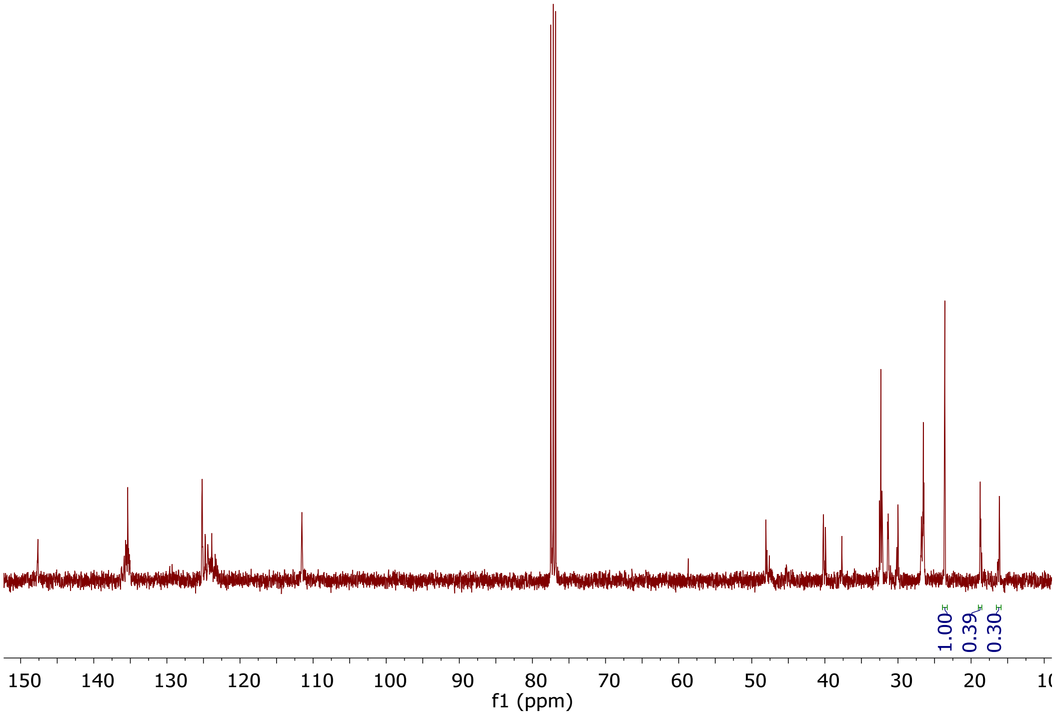

Fig. S110 ^{13}C NMR spectrum of PIP 500 equivalents generated by $\mathbf{Y(CH_2SiMe_3)_3(THF)_2}$, 2 equivalents $[Ph_3C][B(C_6F_5)_4]$, and 1 equivalent P(*p*-tolyl)$_3$ from **Table S1**, entry 2 in $CDCl_3$ at 298 K (30 min).

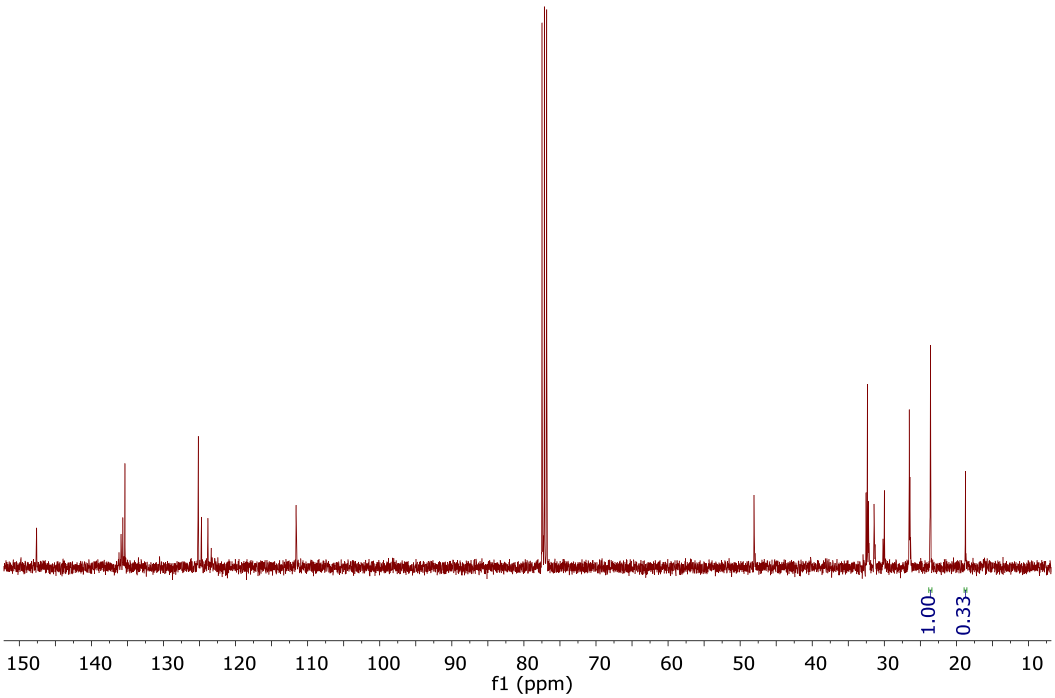

Fig. S111 ^{13}C NMR spectrum of PIP 500 equivalents generated by **$Y(CH_2SiMe_3)_3(THF)_2$**, 2 equivalents $[Ph_3C][B(C_6F_5)_4]$, and 1 equivalent P(Ph-*p*-F)$_3$ from **Table S1**, entry 4 in $CDCl_3$ at 298 K (10 min).

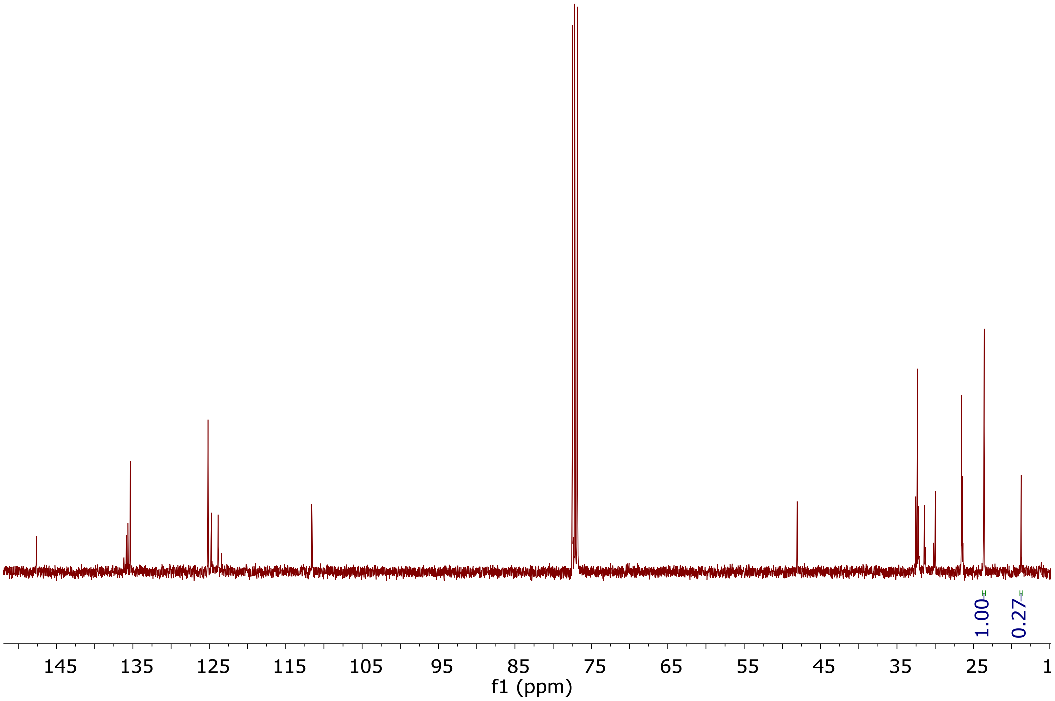

Fig. S112 ^{13}C NMR spectrum of PIP 500 equivalents generated by **$Y(CH_2SiMe_3)_3(THF)_2$** and 2 equivalents $[Ph_3C][B(C_6F_5)_4]$ from **Table S2**, entry 1 in $CDCl_3$ at 298 K (5 min).

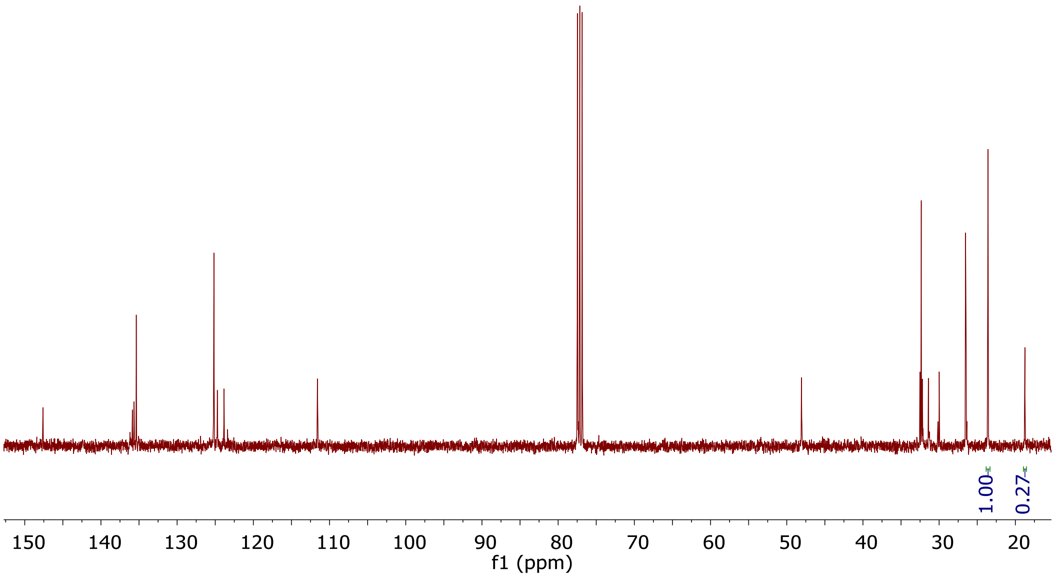

Fig. S113 ^{13}C NMR spectrum of PIP 500 equivalents generated by **Y(CH$_2$SiMe$_3$)$_3$(THF)$_2$** and 2 equivalents [Ph$_3$C][B(C$_6$F$_5$)$_4$] from **Table S2**, entry 2 in CDCl$_3$ at 298 K (12 min).

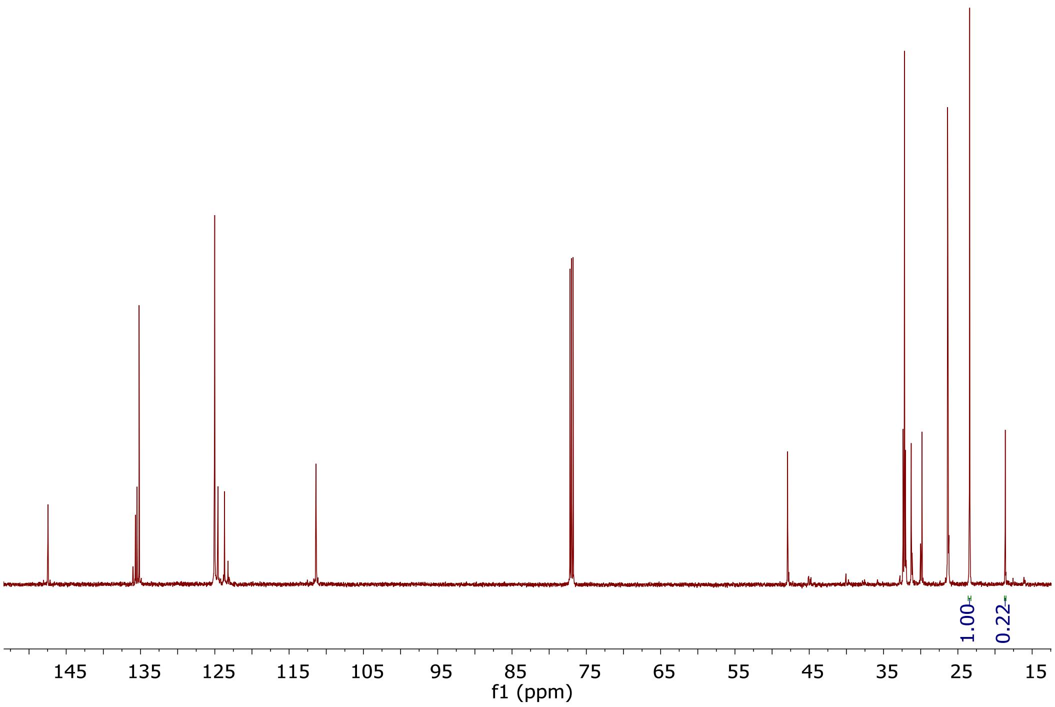

Fig. S114 ^{13}C NMR spectrum of PIP 500 equivalents generated by **Y(CH$_2$SiMe$_3$)$_3$(THF)$_2$** and 2 equivalents [Ph$_3$C][B(C$_6$F$_5$)$_4$] from **Table S2**, entry 3 in CDCl$_3$ at 298 K (18 min).

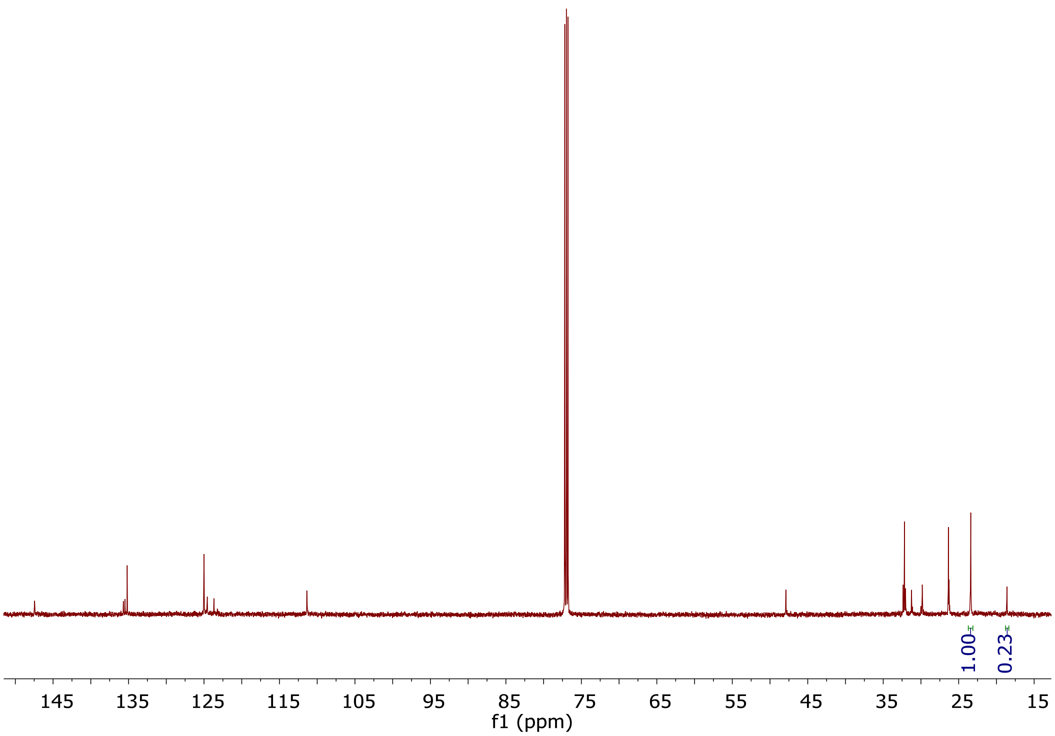

Fig. S115 ^{13}C NMR spectrum of PIP 500 equivalents generated by **Y(CH$_2$SiMe$_3$)$_3$(THF)$_2$** and 2 equivalents [Ph$_3$C][B(C$_6$F$_5$)$_4$] from **Table S2**, entry 4 in CDCl$_3$ at 298 K (24 min).

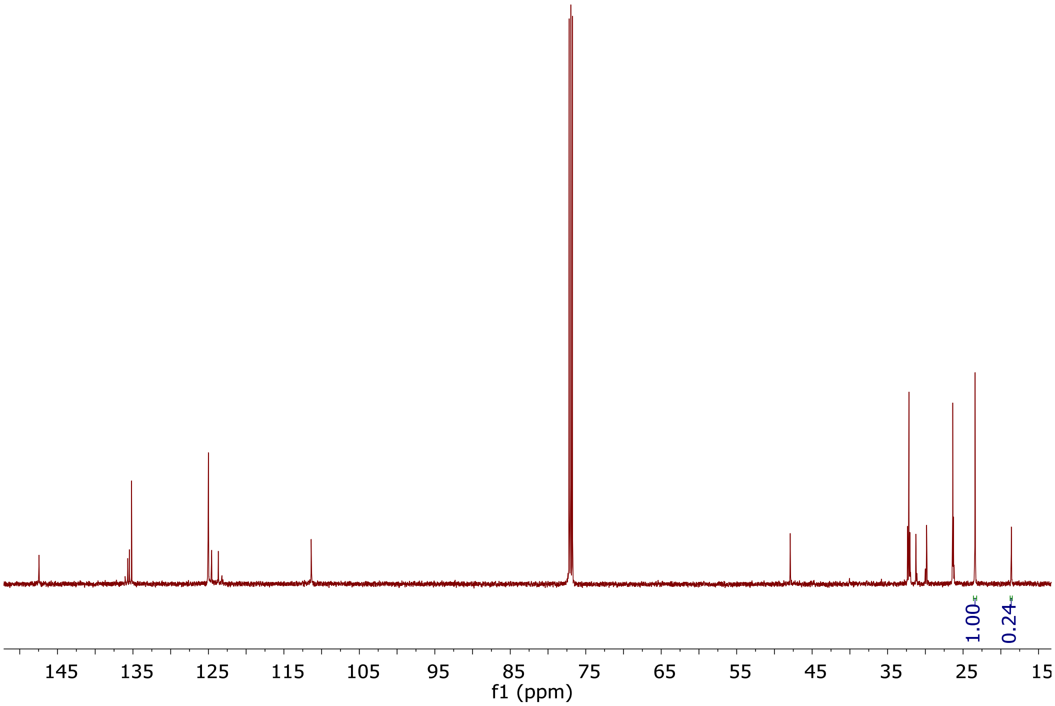

Fig. S116 ^{13}C NMR spectrum of PIP 500 equivalents generated by **Y(CH$_2$SiMe$_3$)$_3$(THF)$_2$** and 2 equivalents [Ph$_3$C][B(C$_6$F$_5$)$_4$] from **Table S2**, entry 5 in CDCl$_3$ at 298 K (30 min).

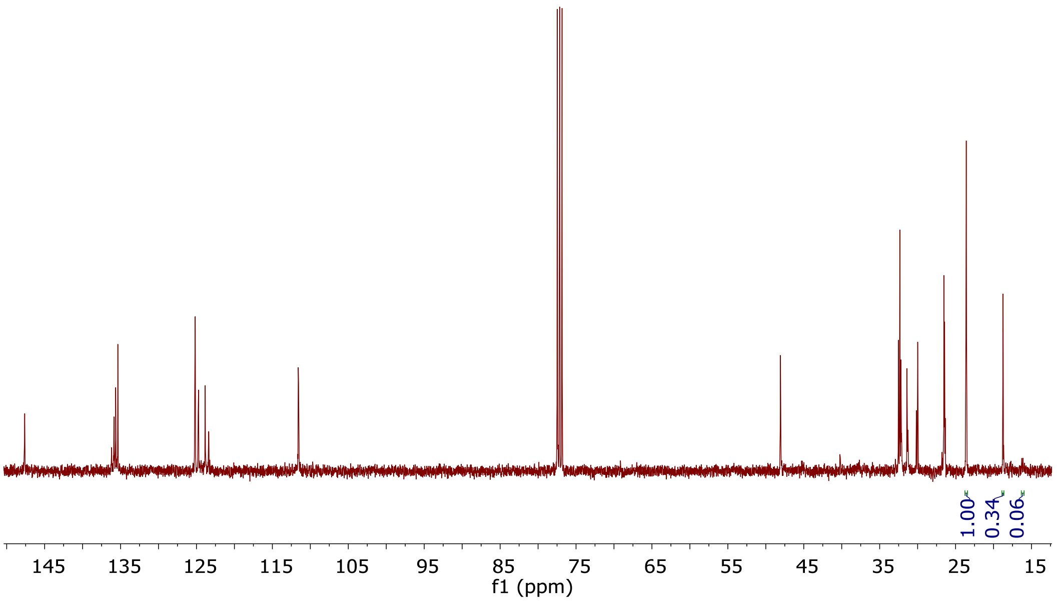

Fig. S117 ^{13}C NMR spectrum of PIP 500 equivalents generated by **$Y(CH_2SiMe_3)_3(THF)_2$**, 2 equivalents $[Ph_3C][B(C_6F_5)_4]$, and 1 equivalent PPh_3 from **Table S3**, entry 1 in $CDCl_3$ at 298 K (10 min).

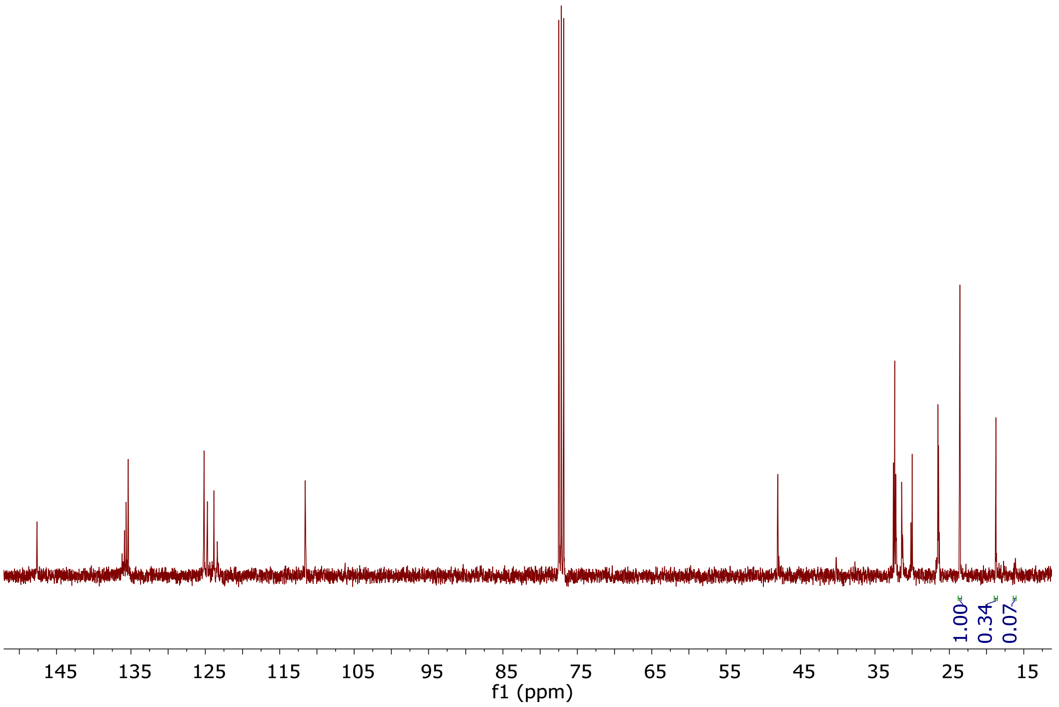

Fig. S118 ^{13}C NMR spectrum of PIP 500 equivalents generated by **$Y(CH_2SiMe_3)_3(THF)_2$**, 2 equivalents $[Ph_3C][B(C_6F_5)_4]$, and 1 equivalent PPh_3 from **Table S3**, entry 2 in $CDCl_3$ at 298 K (21 min).

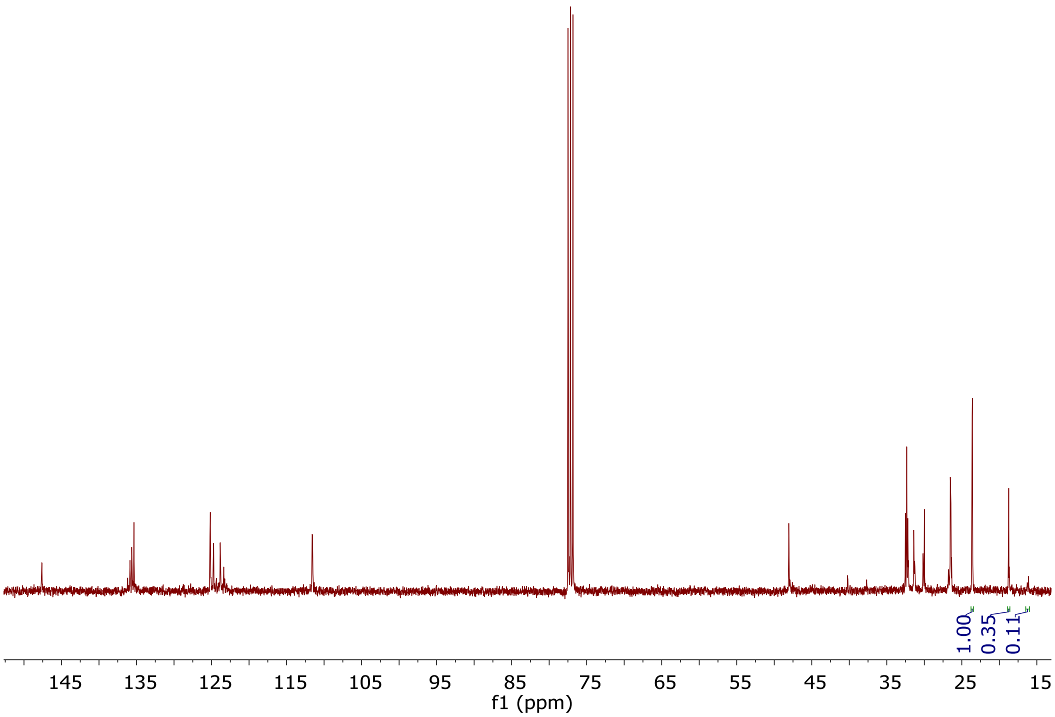

Fig. S119 ^{13}C NMR spectrum of PIP 500 equivalents generated by **Y(CH$_2$SiMe$_3$)$_3$(THF)$_2$**, 2 equivalents [Ph$_3$C][B(C$_6$F$_5$)$_4$], and 1 equivalent PPh$_3$ from **Table S3**, entry 3 in CDCl$_3$ at 298 K (31 min).

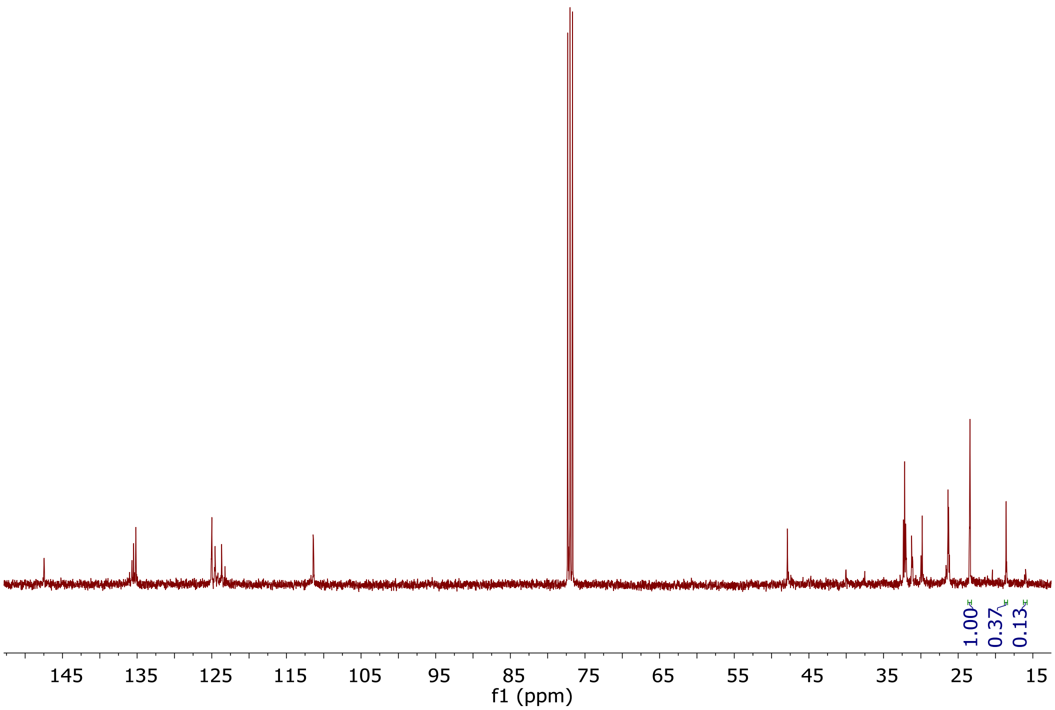

Fig. S120 ^{13}C NMR spectrum of PIP 500 equivalents generated by **Y(CH$_2$SiMe$_3$)$_3$(THF)$_2$**, 2 equivalents [Ph$_3$C][B(C$_6$F$_5$)$_4$], and 1 equivalent PPh$_3$ from **Table S3**, entry 4 in CDCl$_3$ at 298 K (41 min).

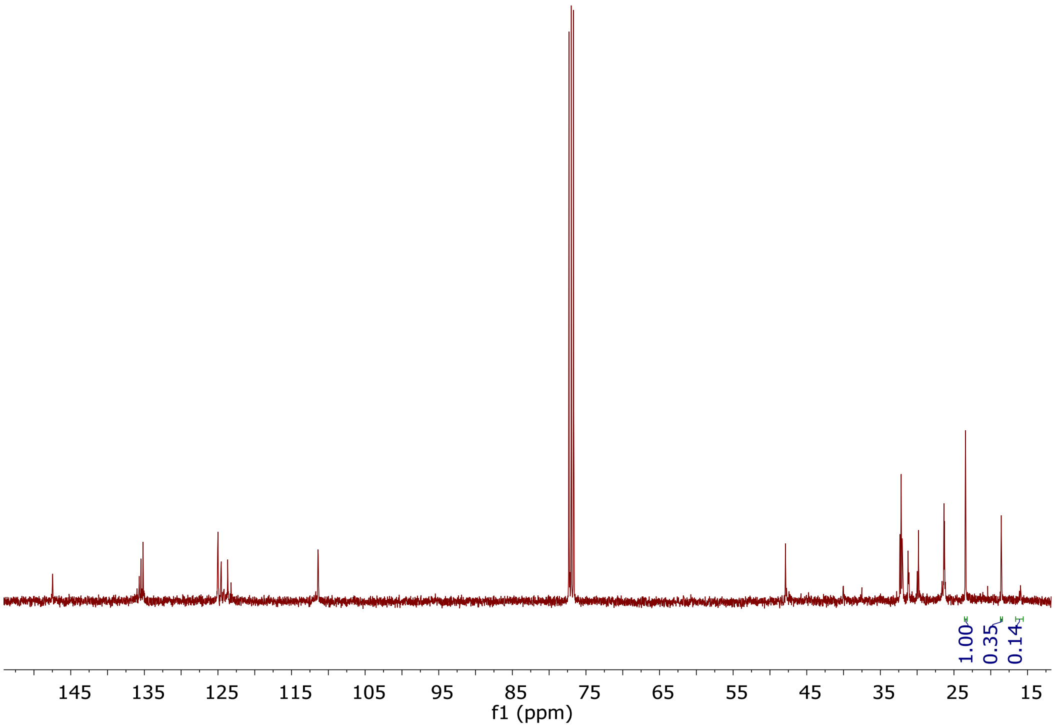

Fig. S121 ^{13}C NMR spectrum of PIP 500 equivalents generated by **$Y(CH_2SiMe_3)_3(THF)_2$**, 2 equivalents $[Ph_3C][B(C_6F_5)_4]$, and 1 equivalent PPh_3 from **Table S3**, entry 5 in $CDCl_3$ at 298 K (51 min).

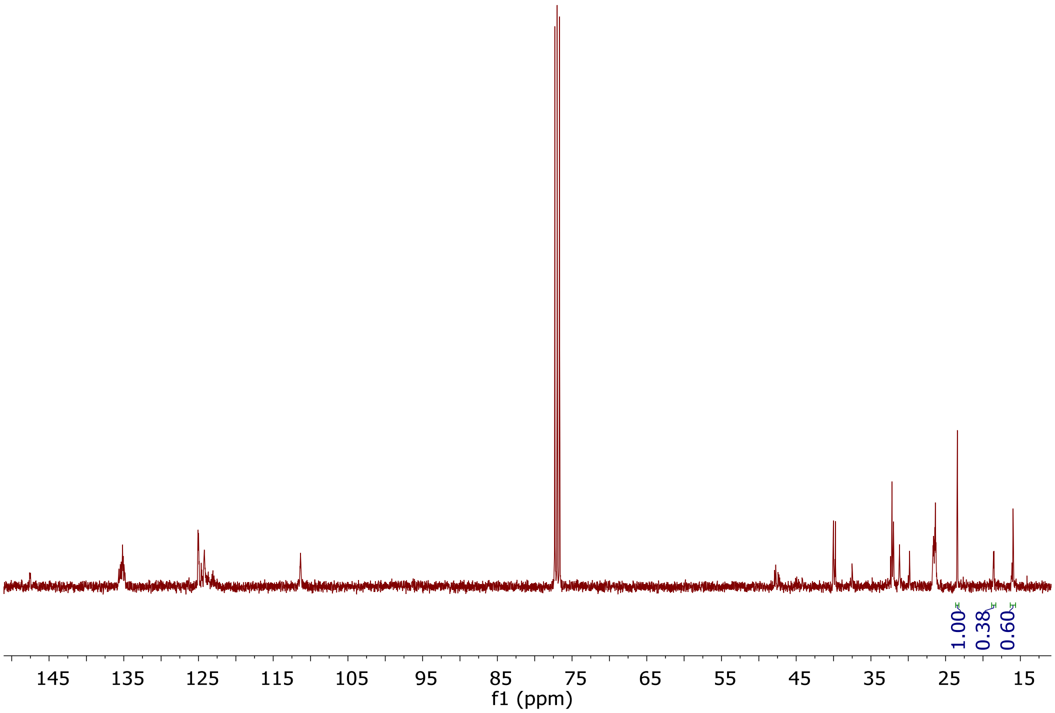

Fig. S122 ^{13}C NMR spectrum of PIP 500 equivalents generated by **$Sm(CH_2SiMe_3)_3(THF)_3$** and 1 equivalent $[Ph_3C][B(C_6F_5)_4]$ from **Table S4**, entry 1 in $CDCl_3$ at 298 K (7 h).

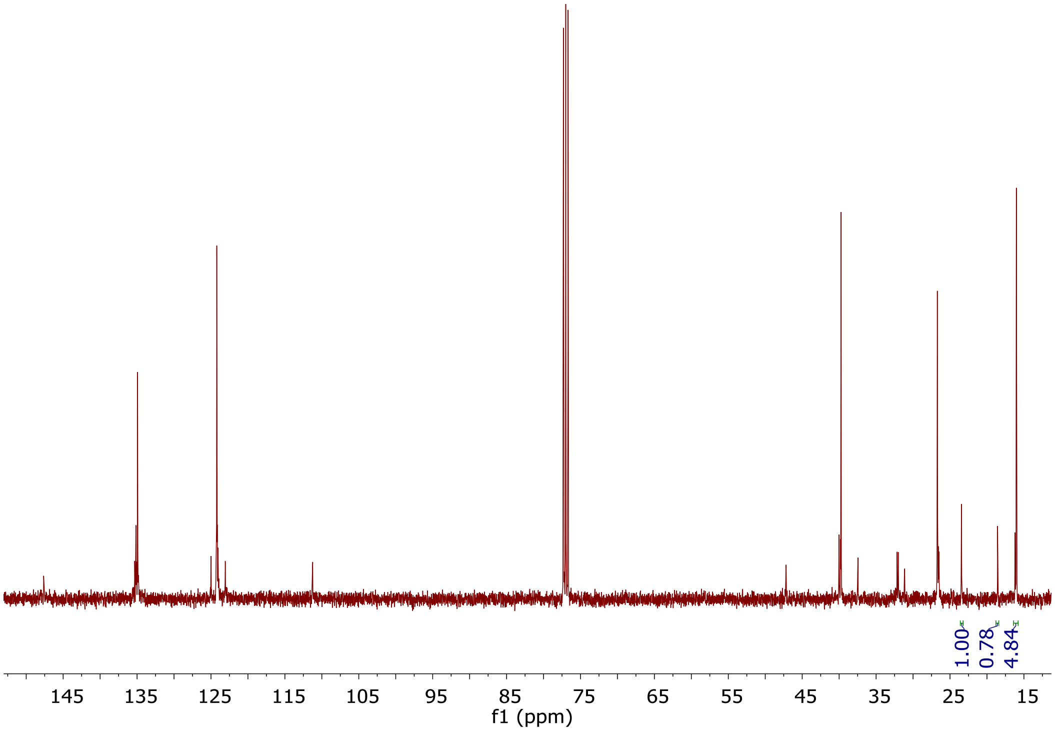

Fig. S123 ^{13}C NMR spectrum of PIP 500 equivalents generated by **Gd(CH$_2$SiMe$_3$)$_3$(THF)$_2$** and 1 equivalent [Ph$_3$C][B(C$_6$F$_5$)$_4$] from **Table S4**, entry 2 in CDCl$_3$ at 298 K (7 h).

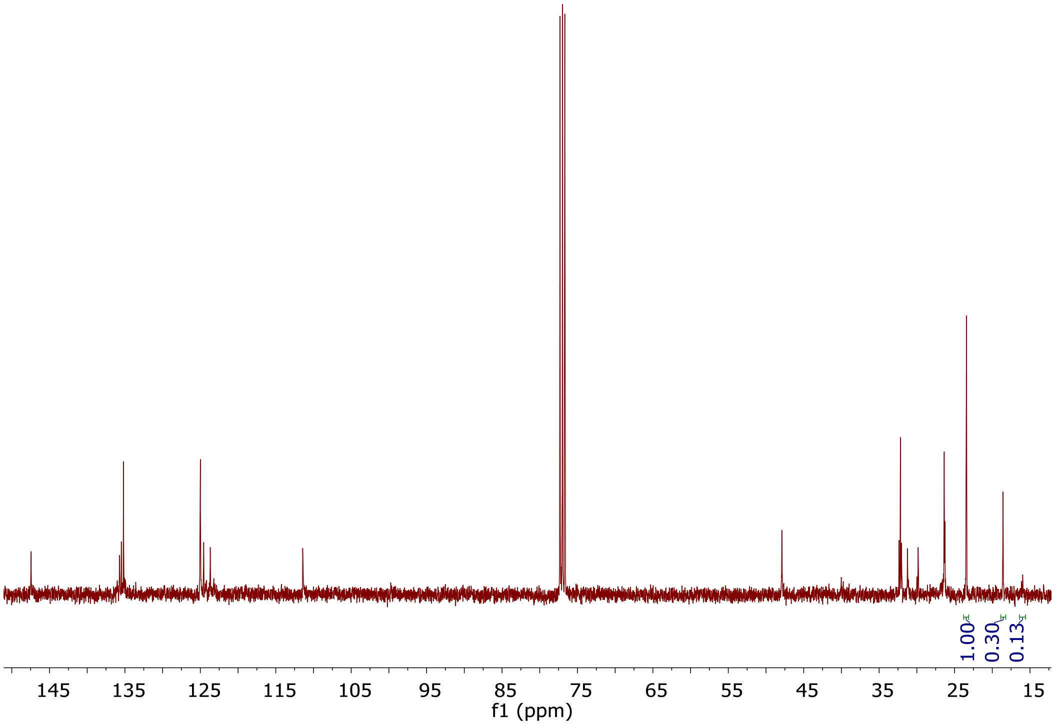

Fig. S124 ^{13}C NMR spectrum of PIP 500 equivalents generated by **Gd(CH$_2$SiMe$_3$)$_3$(THF)$_2$** and 2 equivalents [Ph$_3$C][B(C$_6$F$_5$)$_4$] from **Table S4**, entry 3 in CDCl$_3$ at 298 K (7 h).

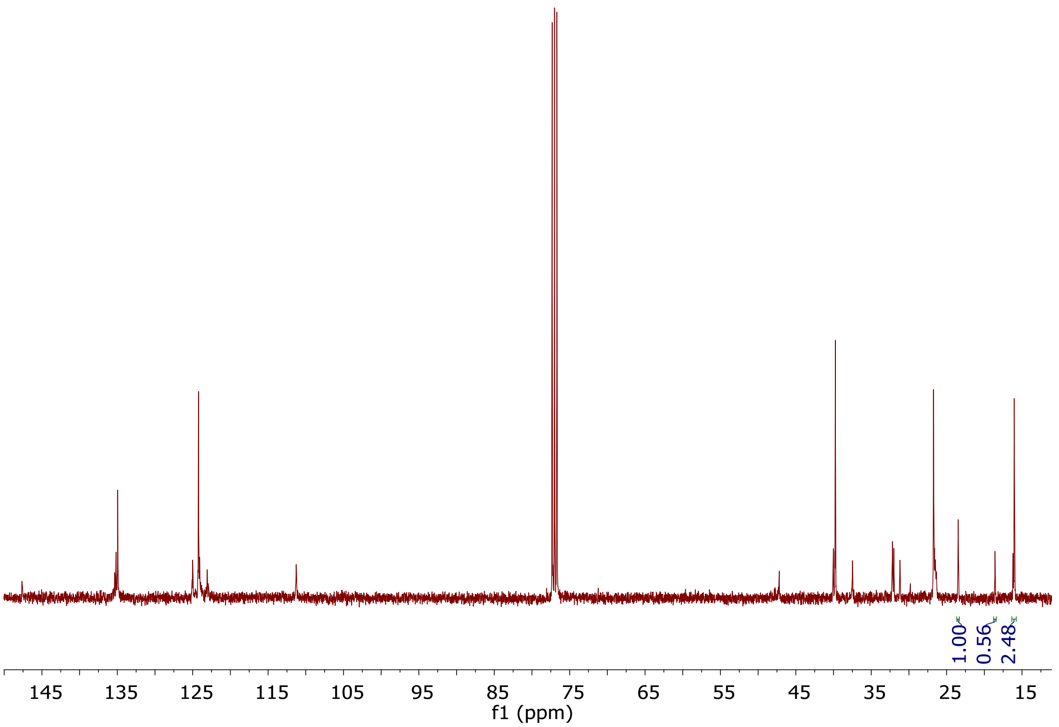

Fig. S125 ^{13}C NMR spectrum of PIP 500 equivalents generated by **$Tm(CH_2SiMe_3)_3(THF)_2$** and 1 equivalent $[Ph_3C][B(C_6F_5)_4]$ from **Table S4**, entry 4 in $CDCl_3$ at 298 K (7 h).

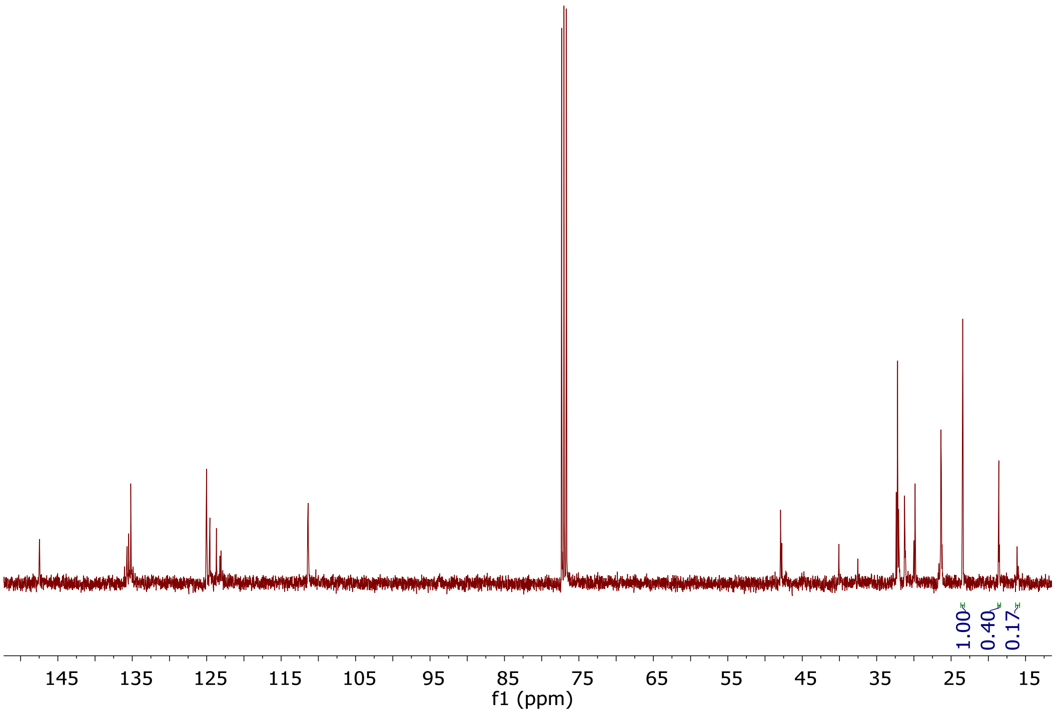

Fig. S126 ^{13}C NMR spectrum of PIP 500 equivalents generated by **$Tm(CH_2SiMe_3)_3(THF)_2$** and 2 equivalents $[Ph_3C][B(C_6F_5)_4]$ from **Table S4**, entry 5 in $CDCl_3$ at 298 K (7 h).

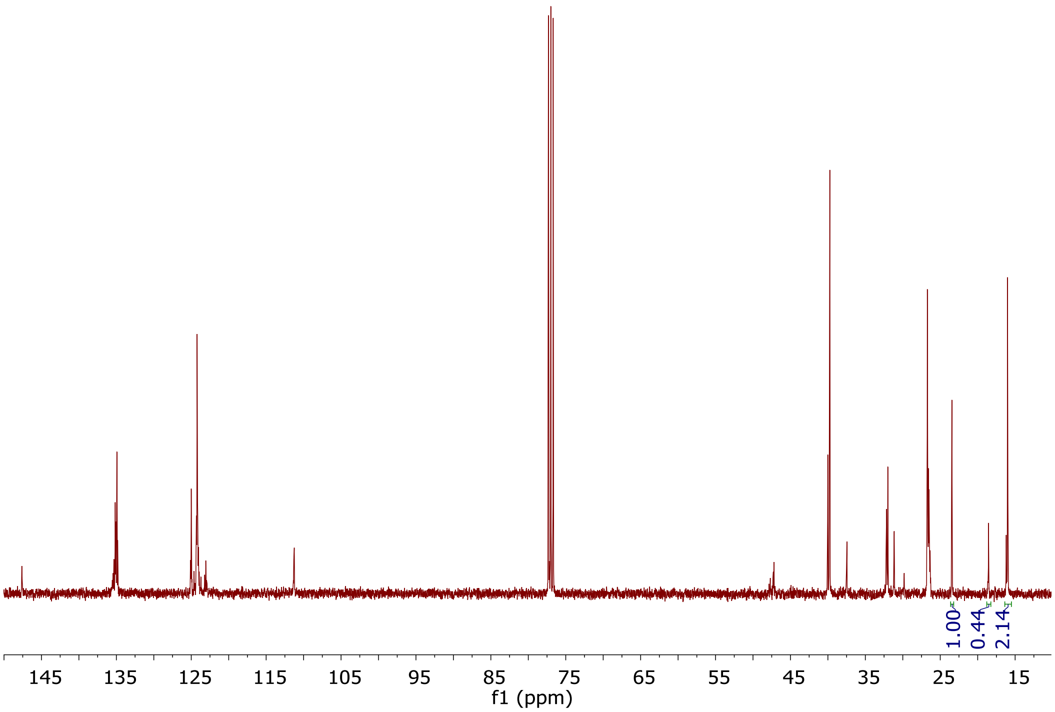

Fig. S127 ^{13}C NMR spectrum of PIP 500 equivalents generated by **$Sm(CH_2SiMe_3)_3(THF)_3$**, 1 equivalent $[Ph_3C][B(C_6F_5)_4]$, and 1 equivalent PPh_3 from **Table S4**, entry 6 in $CDCl_3$ at 298 K (7 h).

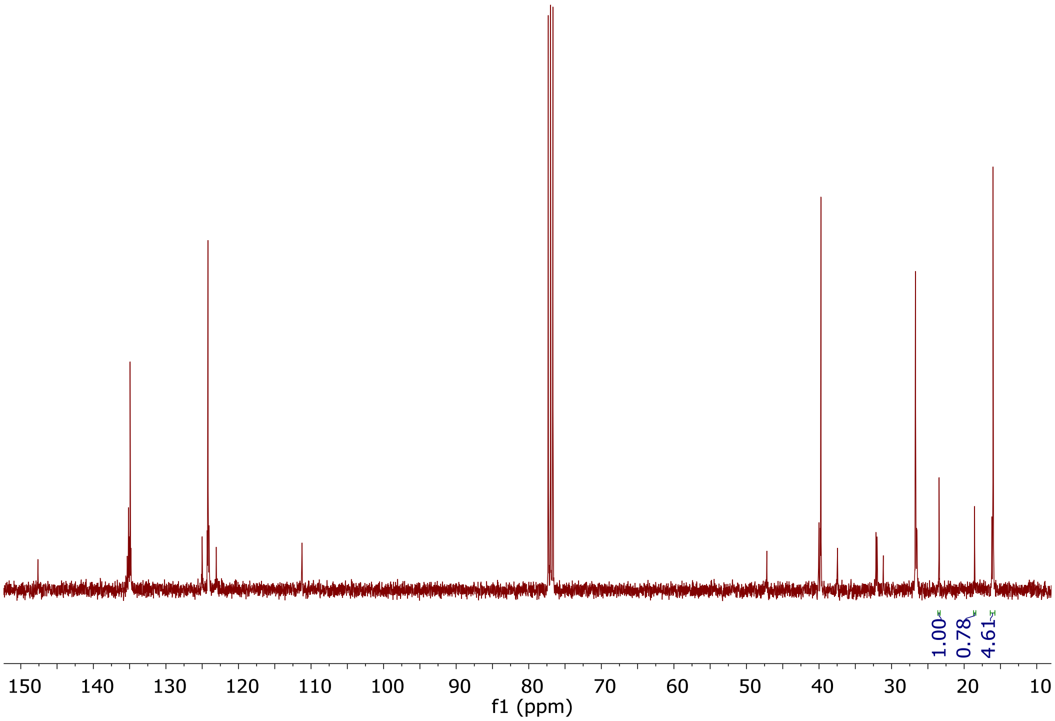

Fig. S128 ^{13}C NMR spectrum of PIP 500 equivalents generated by **$Gd(CH_2SiMe_3)_3(THF)_2$**, 1 equivalent $[Ph_3C][B(C_6F_5)_4]$, and 1 equivalent PPh_3 from **Table S4**, entry 7 in $CDCl_3$ at 298 K (7 h).

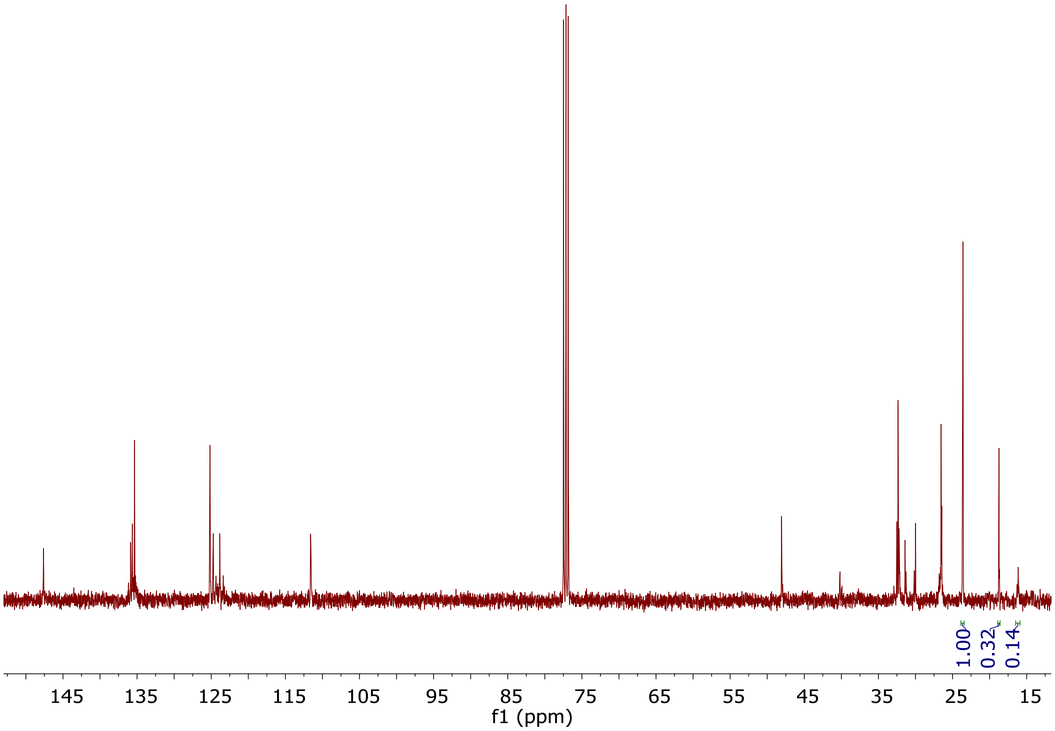

Fig. S129 ^{13}C NMR spectrum of PIP 500 equivalents generated by **$Gd(CH_2SiMe_3)_3(THF)_2$**, 2 equivalents $[Ph_3C][B(C_6F_5)_4]$, and 1 equivalent PPh_3 from **Table S4**, entry 8 in $CDCl_3$ at 298 K (7 h).

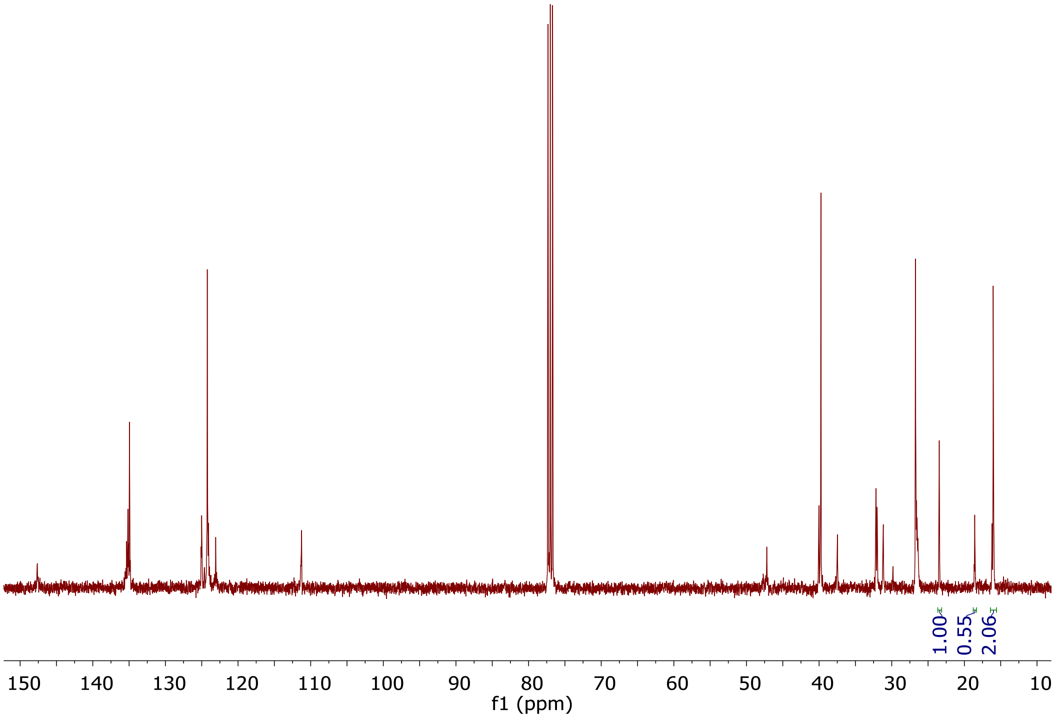

Fig. S130 ^{13}C NMR spectrum of PIP 500 equivalents generated by **$Tm(CH_2SiMe_3)_3(THF)_2$**, 1 equivalent $[Ph_3C][B(C_6F_5)_4]$, and 1 equivalent PPh_3 from **Table S4**, entry 9 in $CDCl_3$ at 298 K (7 h).

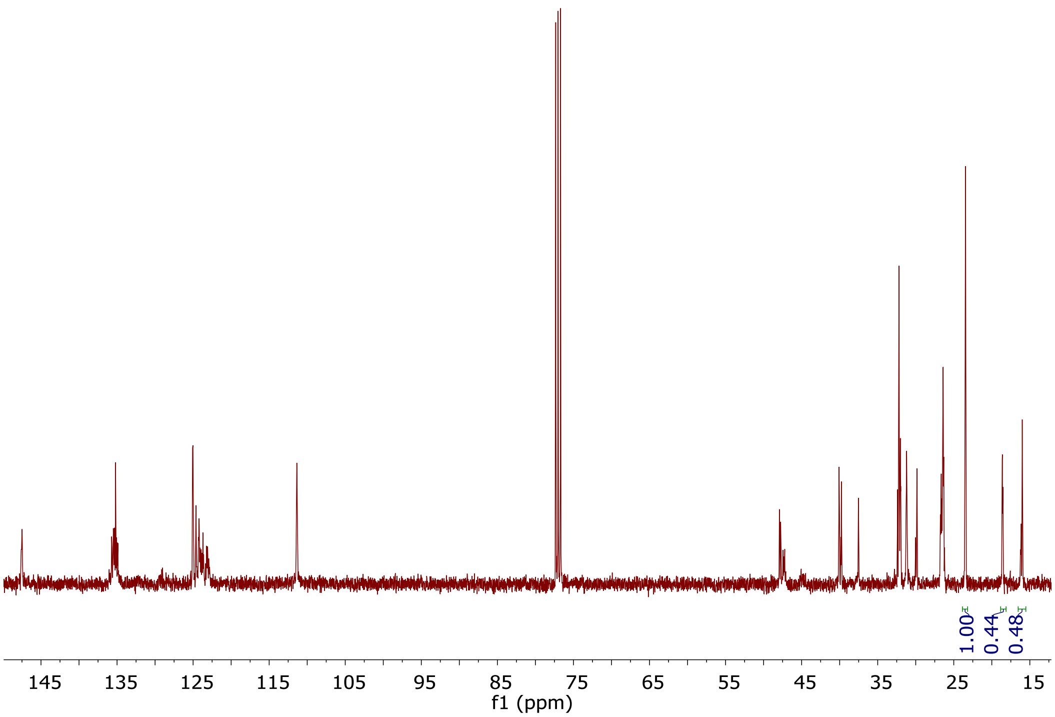

Fig. S131 ^{13}C NMR spectrum of PIP 500 equivalents generated by **Tm(CH$_2$SiMe$_3$)$_3$(THF)$_2$**, 2 equivalents [Ph$_3$C][B(C$_6$F$_5$)$_4$], and 1 equivalent PPh$_3$ from **Table S4**, entry 10 in CDCl$_3$ at 298 K (7 h).

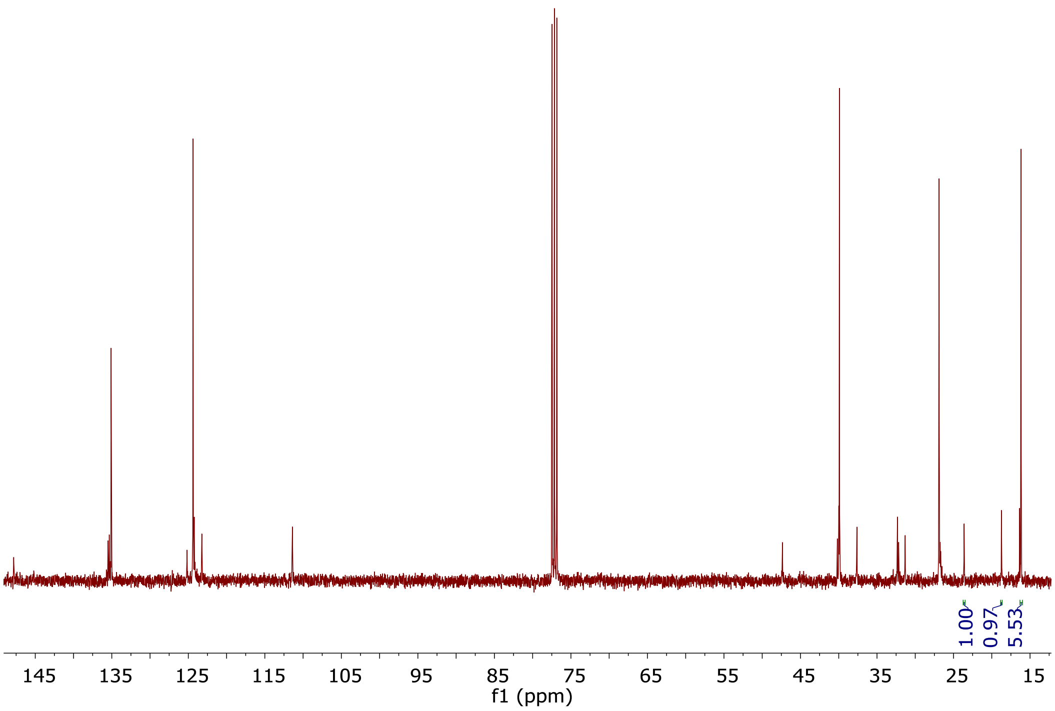

Fig. S132 ^{13}C NMR spectrum of PIP 500 equivalents generated by **Y(CH$_2$SiMe$_3$)$_3$(THF)$_2$** and 1 equivalent [Ph$_3$C][B(C$_6$F$_5$)$_4$] from **Table 3**, entry 1 in CDCl$_3$ at 298 K (7 h).

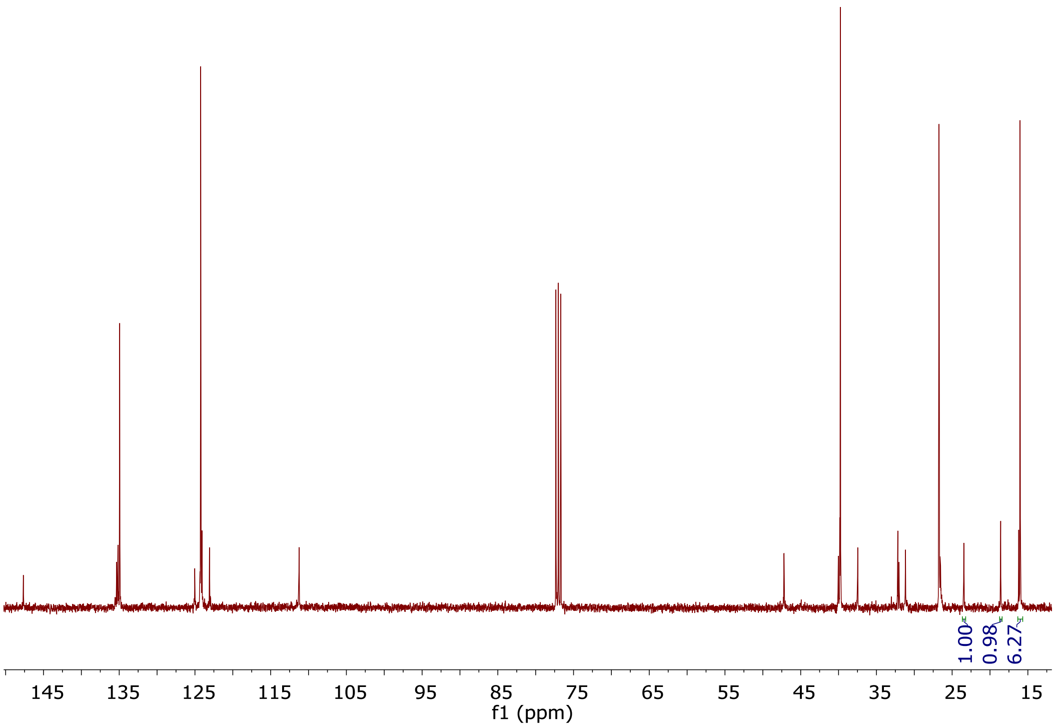

Fig. S133 ^{13}C NMR spectrum of PIP 500 equivalents generated by **$Y(CH_2SiMe_3)_3(THF)_2$**, 1 equivalent $[Ph_3C][B(C_6F_5)_4]$, and 1 equivalent PPh_3 from **Table 3**, entry 2 in $CDCl_3$ at 298 K (7 h).

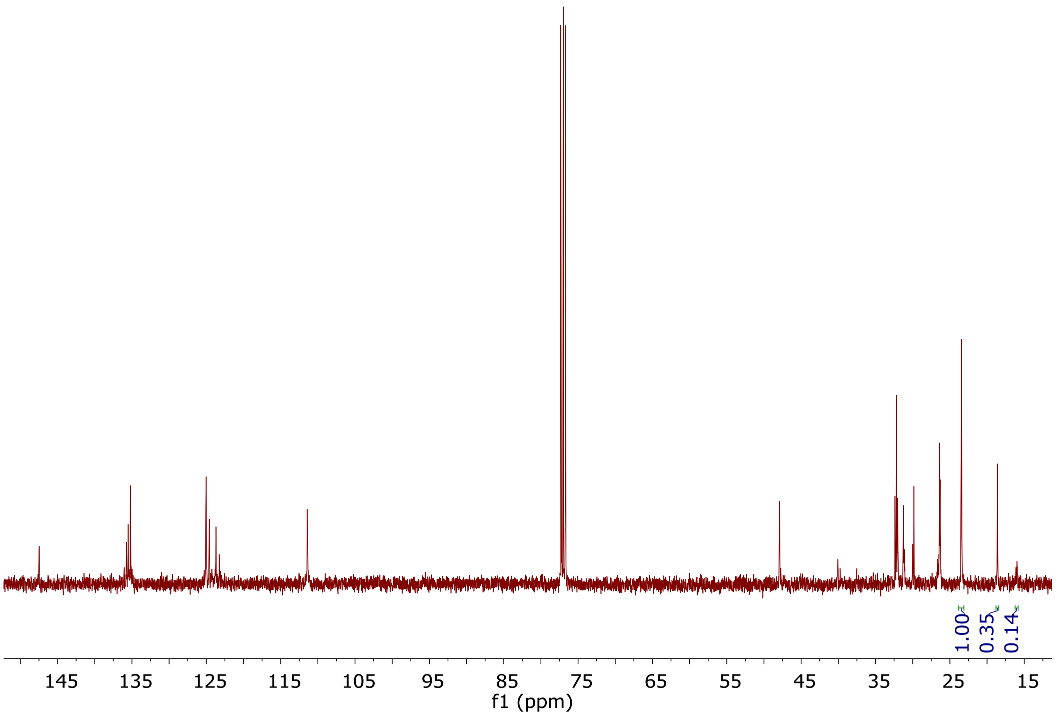

Fig. S134 ^{13}C NMR spectrum of PIP 500 equivalents generated by **$Y(CH_2SiMe_3)_3(THF)_2$**, 1.5 equivalents $[Ph_3C][B(C_6F_5)_4]$ from **Table 3**, entry 3 in $CDCl_3$ at 298 K (7 h).

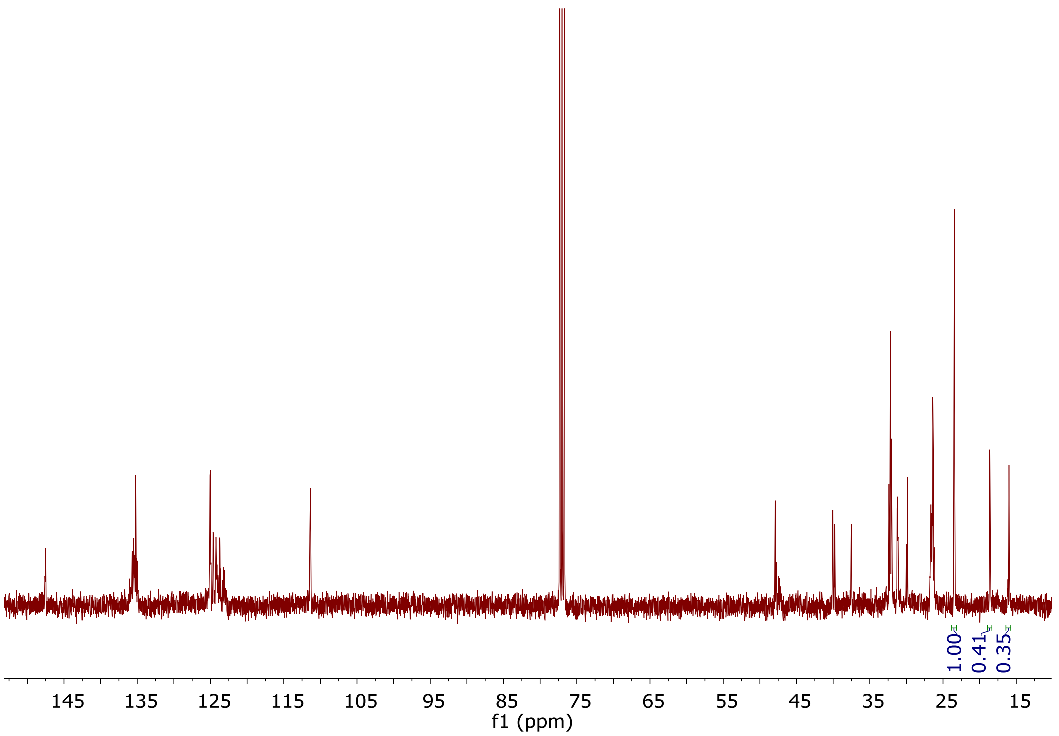

Fig. S135 ^{13}C NMR spectrum of PIP 500 equivalents generated by **$Y(CH_2SiMe_3)_3(THF)_2$**, 1.5 equivalents $[Ph_3C][B(C_6F_5)_4]$, and 1 equivalent PPh_3 from **Table 3**, entry 4 in $CDCl_3$ at 298 K (7 h).

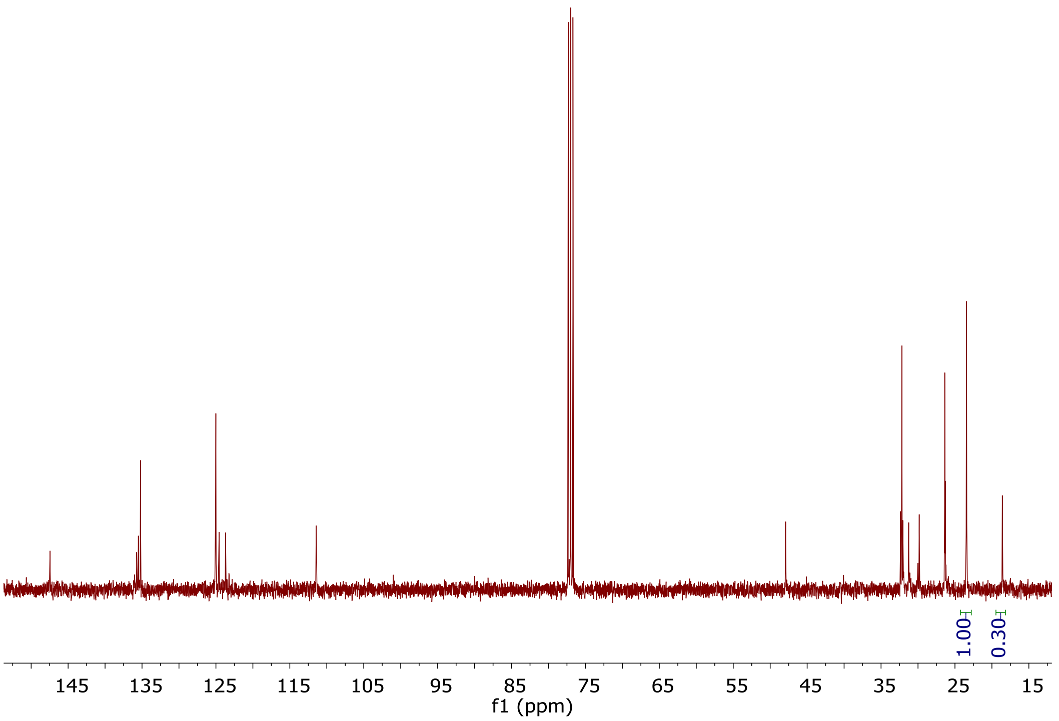

Fig. S136 ^{13}C NMR spectrum of PIP 500 equivalents generated by **$Y(CH_2SiMe_3)_3(THF)_2$** and 2 equivalents $[Ph_3C][B(C_6F_5)_4]$ from **Table 3**, entry 5 in $CDCl_3$ at 298 K (7 h).

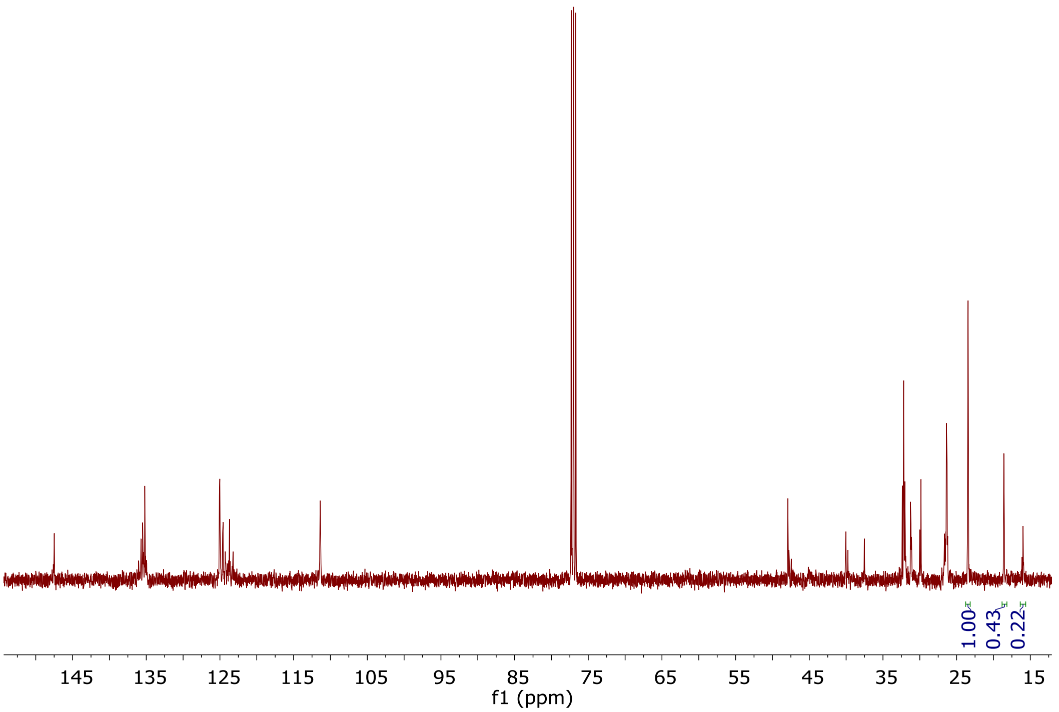

Fig. S137 ^{13}C NMR spectrum of PIP 500 equivalents generated by **$Y(CH_2SiMe_3)_3(THF)_2$**, 2 equivalents $[Ph_3C][B(C_6F_5)_4]$, and 1 equivalent PPh_3 from **Table 3**, entry 6 in $CDCl_3$ at 298 K (7 h).

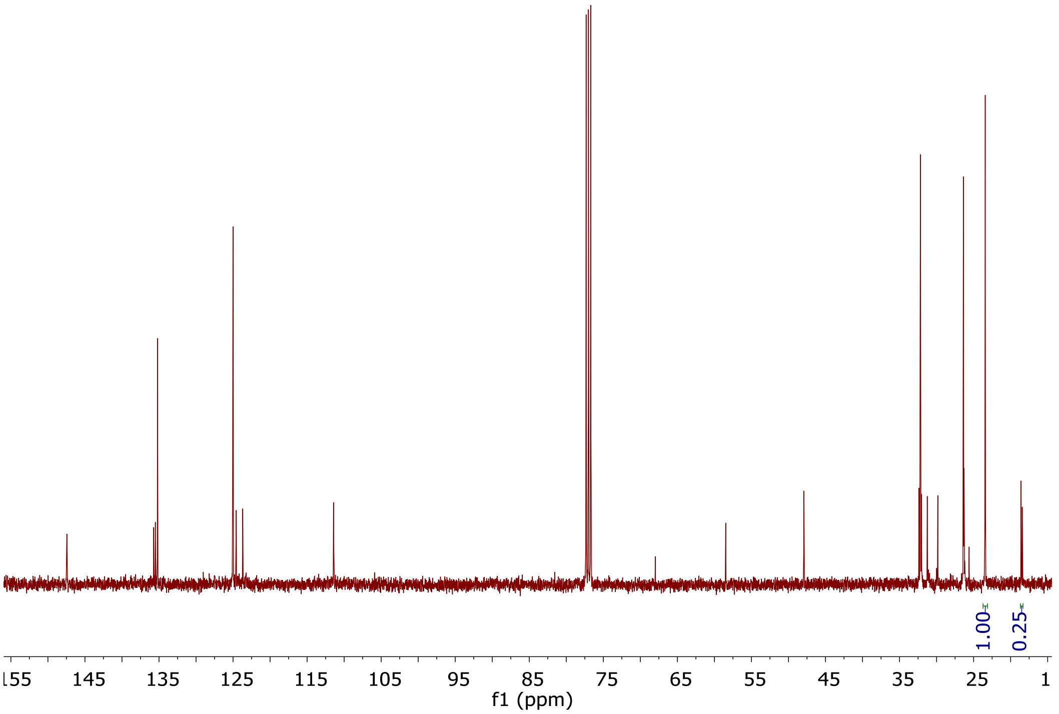

Fig. S138 ^{13}C NMR spectrum of PIP 500 equivalents generated by **$Y(CH_2SiMe_3)_3(THF)_2$** and 2.5 equivalents $[Ph_3C][B(C_6F_5)_4]$ from **Table 3**, entry 7 in $CDCl_3$ at 298 K (7 h).

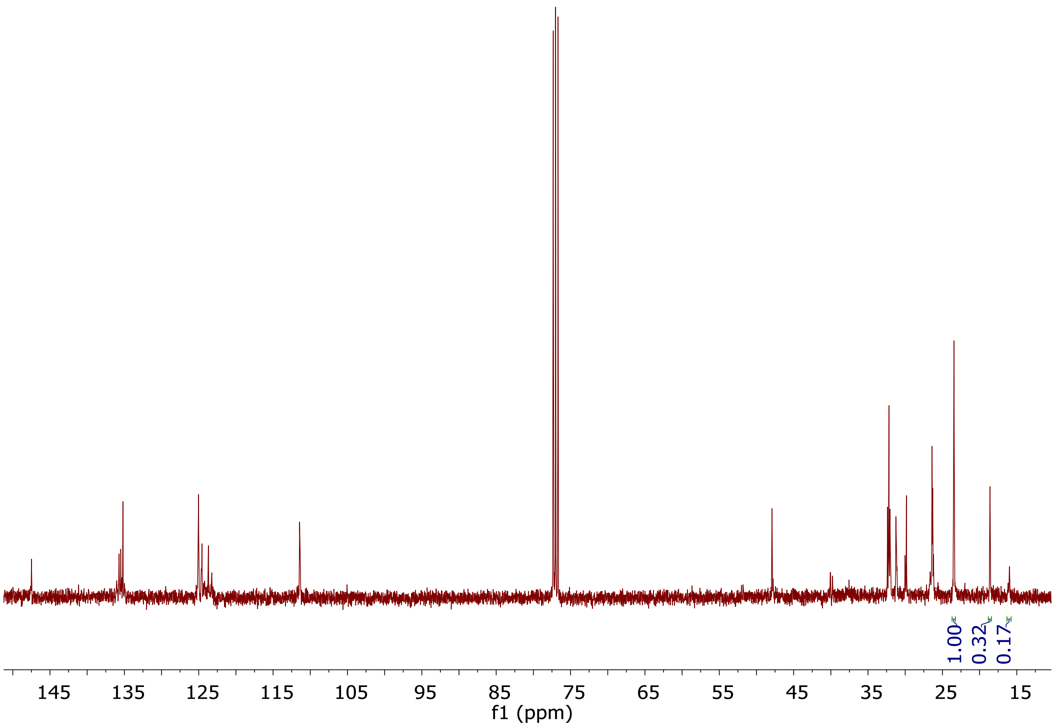

Fig. S139 ^{13}C NMR spectrum of PIP 500 equivalents generated by **$Y(CH_2SiMe_3)_3(THF)_2$**, 2.5 equivalents $[Ph_3C][B(C_6F_5)_4]$, and 1 equivalent PPh_3 from **Table 3**, entry 8 in $CDCl_3$ at 298 K (7 h).

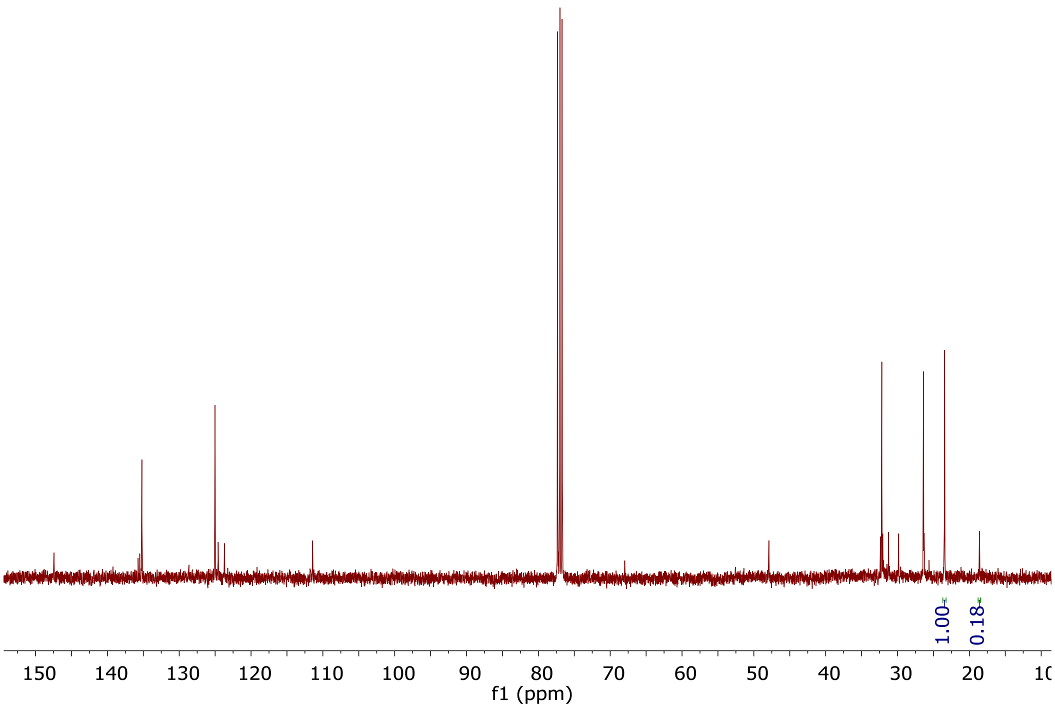

Fig. S140 ^{13}C NMR spectrum of PIP 500 equivalents generated by **$Y(CH_2SiMe_3)_3(THF)_2$** and 3 equivalents $[Ph_3C][B(C_6F_5)_4]$ from **Table 3**, entry 9 in $CDCl_3$ at 298 K (7 h).

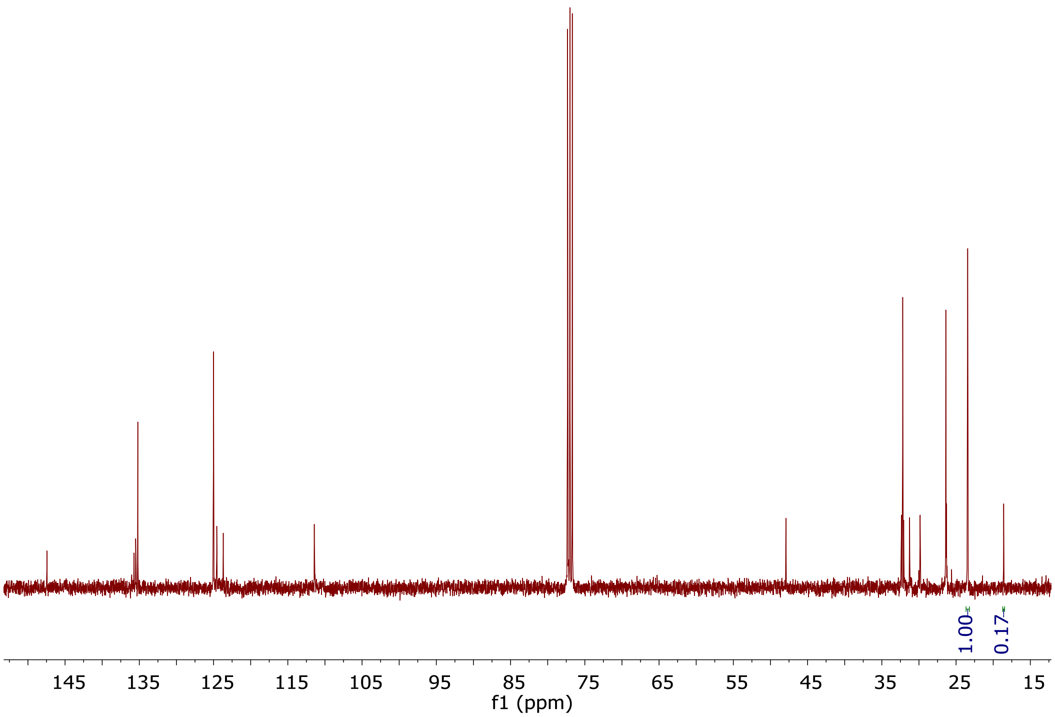

Fig. S141 ^{13}C NMR spectrum of PIP 500 equivalents generated by **$Y(CH_2SiMe_3)_3(THF)_2$**, 3 equivalents $[Ph_3C][B(C_6F_5)_4]$, and 1 equivalent PPh_3 from **Table 3**, entry 10 in $CDCl_3$ at 298 K (7 h).

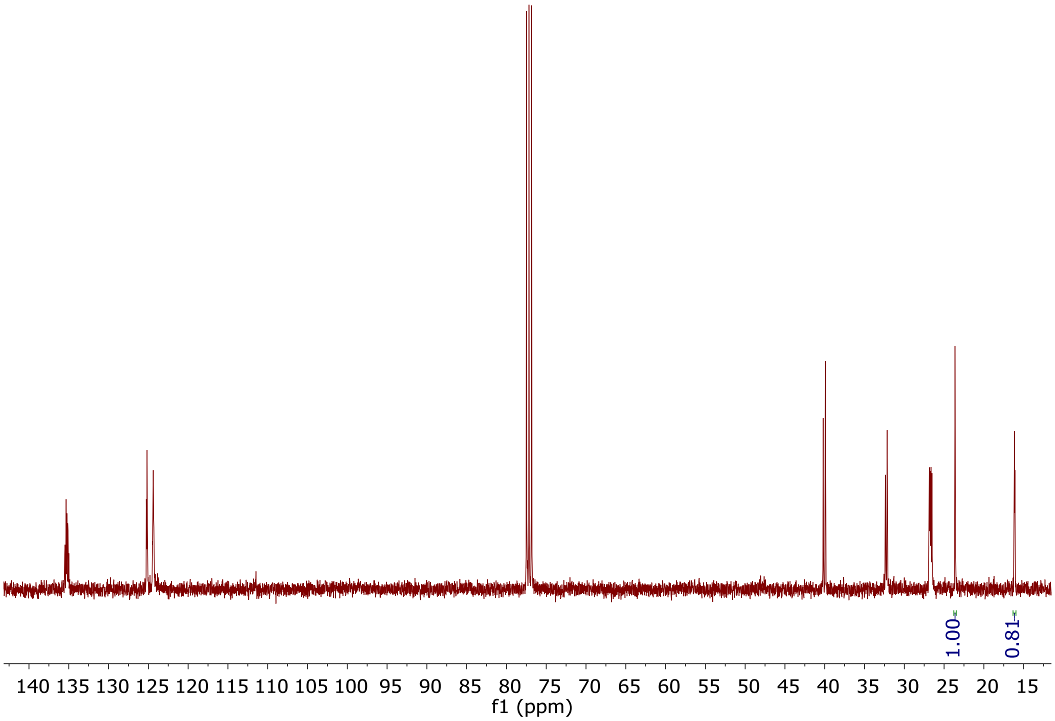

Fig. S142 ^{13}C NMR spectrum of PIP 500 equivalents generated by **$Y(CH_2SiMe_3)_3(THF)_2$**, 2 equivalents $[Ph_3C][B(C_6F_5)_4]$, and 5 equivalents $AlMe_3$ from **Table 4**, entry 1 in $CDCl_3$ at 298 K (30 min).

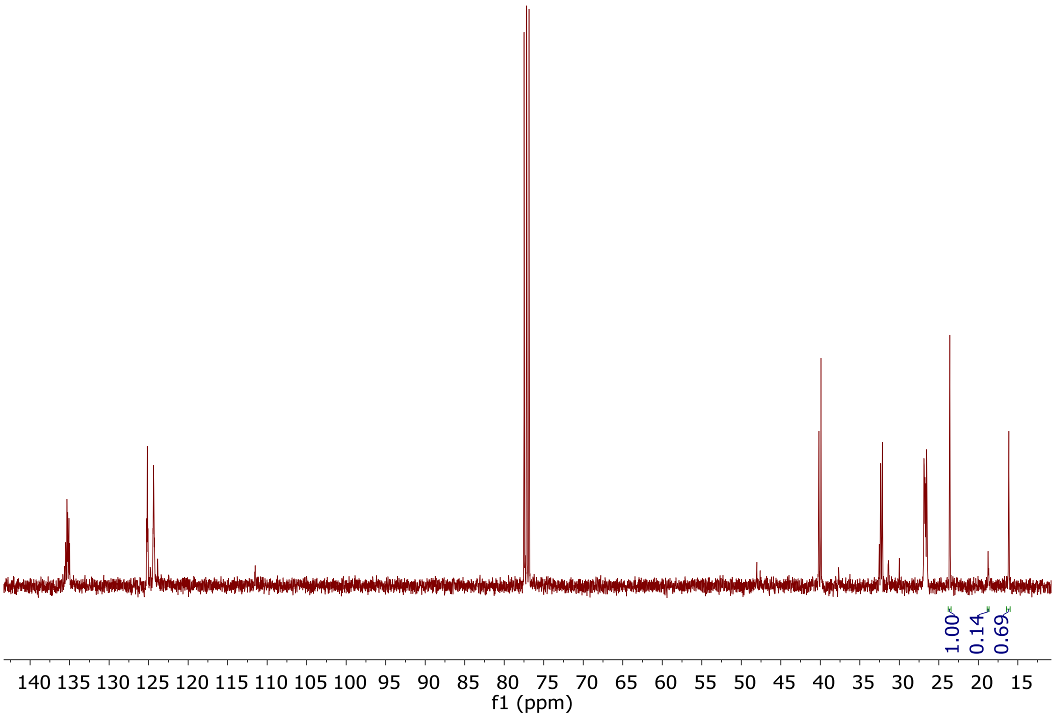

Fig. S143 ^{13}C NMR spectrum of PIP 500 equivalents generated by **$Y(CH_2SiMe_3)_3(THF)_2$**, 2 equivalents $[Ph_3C][B(C_6F_5)_4]$, and 10 equivalents $AlMe_3$ from **Table 4**, entry 2 in $CDCl_3$ at 298 K (30 min).

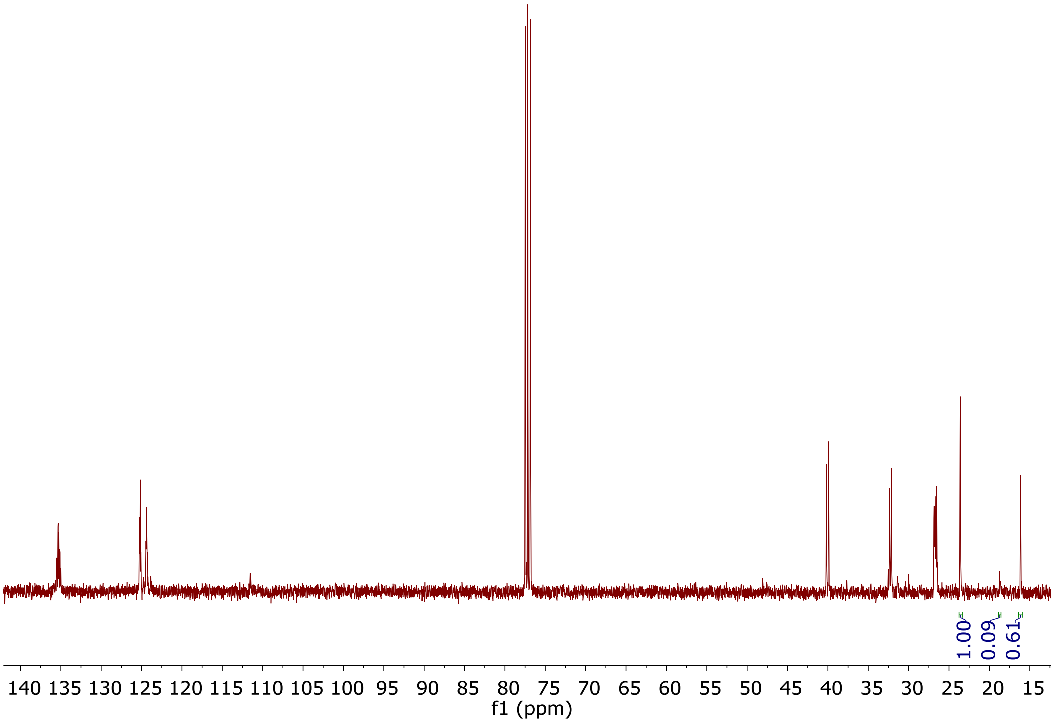

Fig. S144 ^{13}C NMR spectrum of PIP 500 equivalents generated by **$Y(CH_2SiMe_3)_3(THF)_2$**, 2 equivalents $[Ph_3C][B(C_6F_5)_4]$, and 15 equivalents $AlMe_3$ from **Table 4**, entry 3 in $CDCl_3$ at 298 K (30 min).

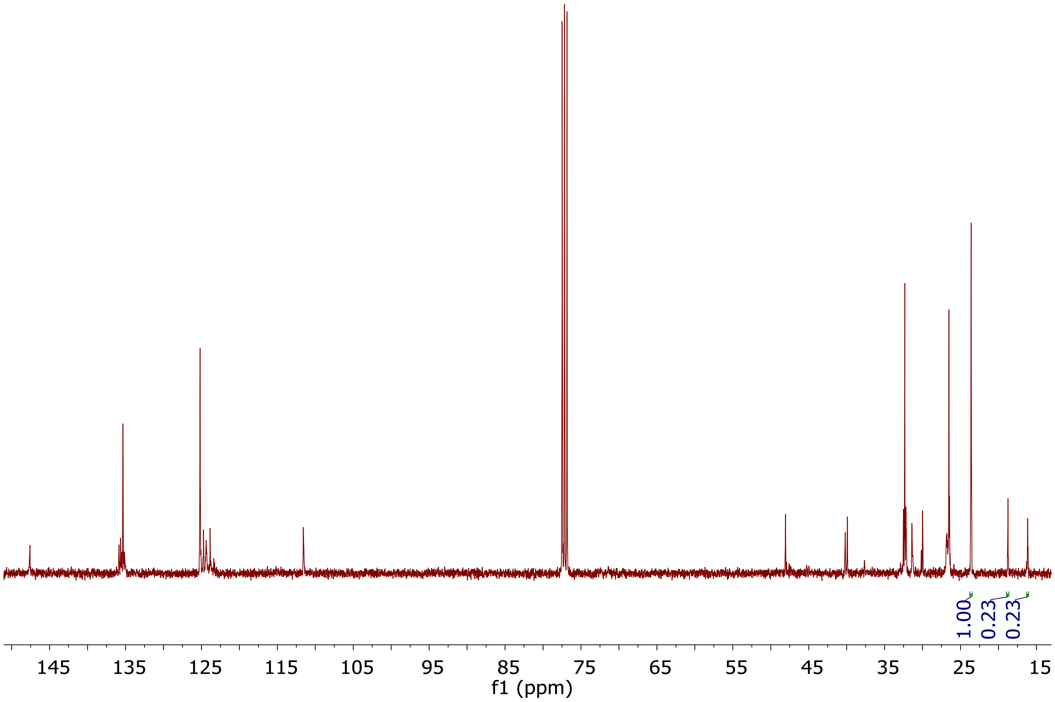

Fig. S145 ^{13}C NMR spectrum of PIP 500 equivalents generated by **$Y(CH_2SiMe_3)_3(THF)_2$**, 2 equivalents $[Ph_3C][B(C_6F_5)_4]$, and 5 equivalents $AlEt_3$ from **Table 4**, entry 4 in $CDCl_3$ at 298 K (30 min).

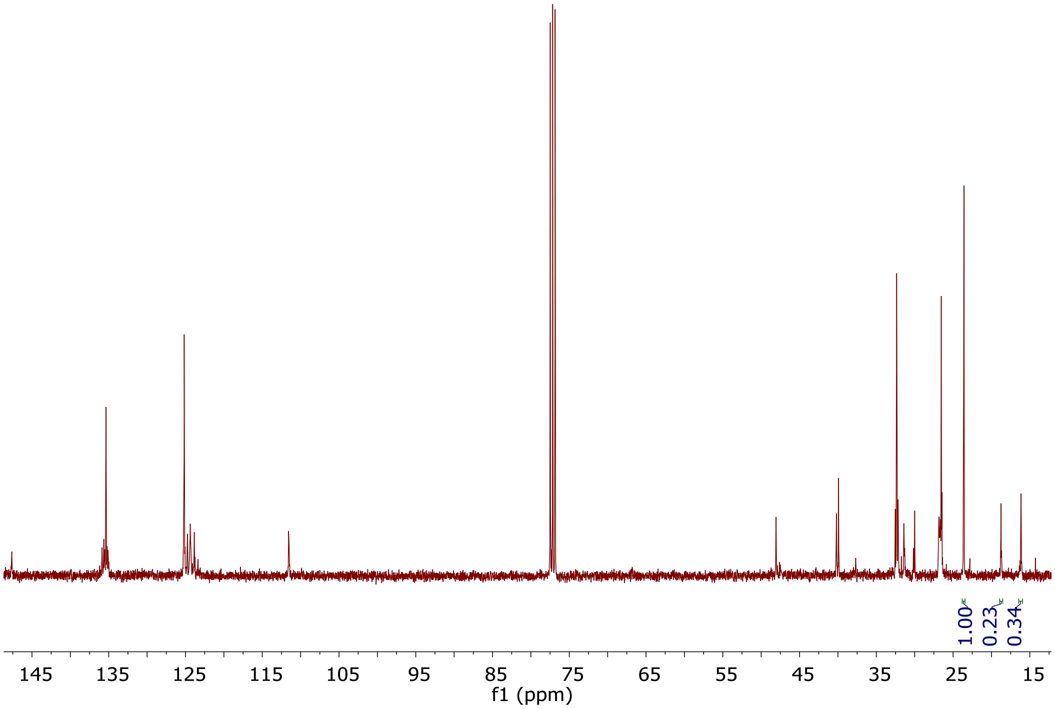

Fig. S146 ^{13}C NMR spectrum of PIP 500 equivalents generated by **$Y(CH_2SiMe_3)_3(THF)_2$**, 2 equivalents $[Ph_3C][B(C_6F_5)_4]$, and 10 equivalents $AlEt_3$ from **Table 4**, entry 5 in $CDCl_3$ at 298 K (30 min).

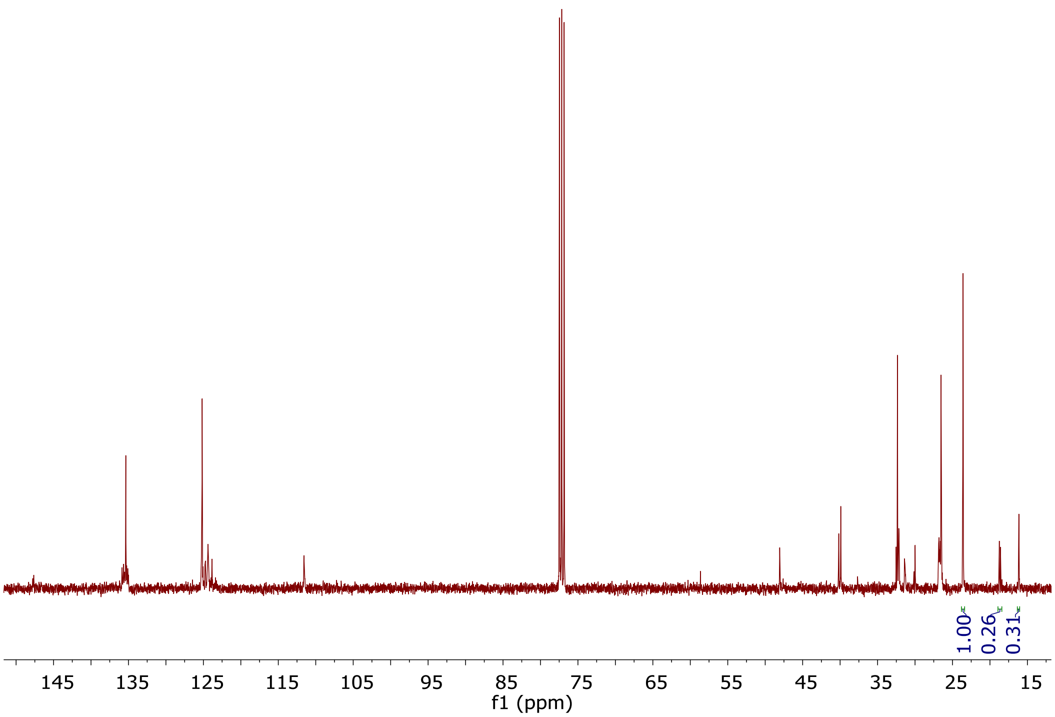

Fig. S147 ^{13}C NMR spectrum of PIP 500 equivalents generated by **$Y(CH_2SiMe_3)_3(THF)_2$**, 2 equivalents $[Ph_3C][B(C_6F_5)_4]$, and 15 equivalents $AlEt_3$ from **Table 4**, entry 6 in $CDCl_3$ at 298 K (30 min).

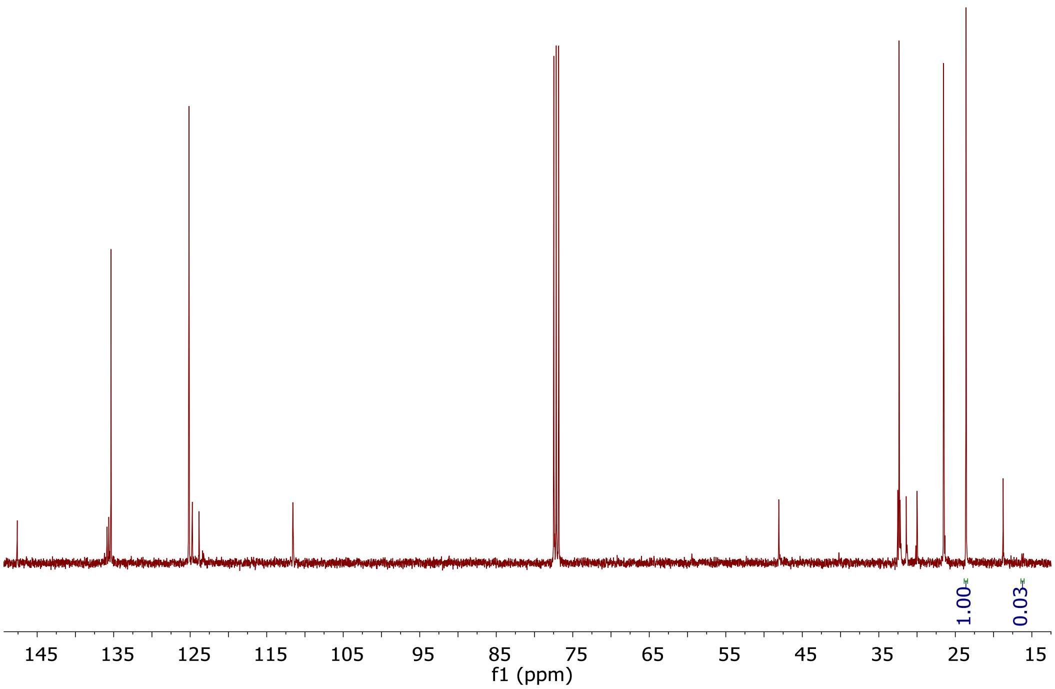

Fig. S148 ^{13}C NMR spectrum of PIP 500 equivalents generated by **$Y(CH_2SiMe_3)_3(THF)_2$**, 2 equivalents $[Ph_3C][B(C_6F_5)_4]$, and 5 equivalents Al^iBu_3 from **Table 4**, entry 7 in $CDCl_3$ at 298 K (30 min).

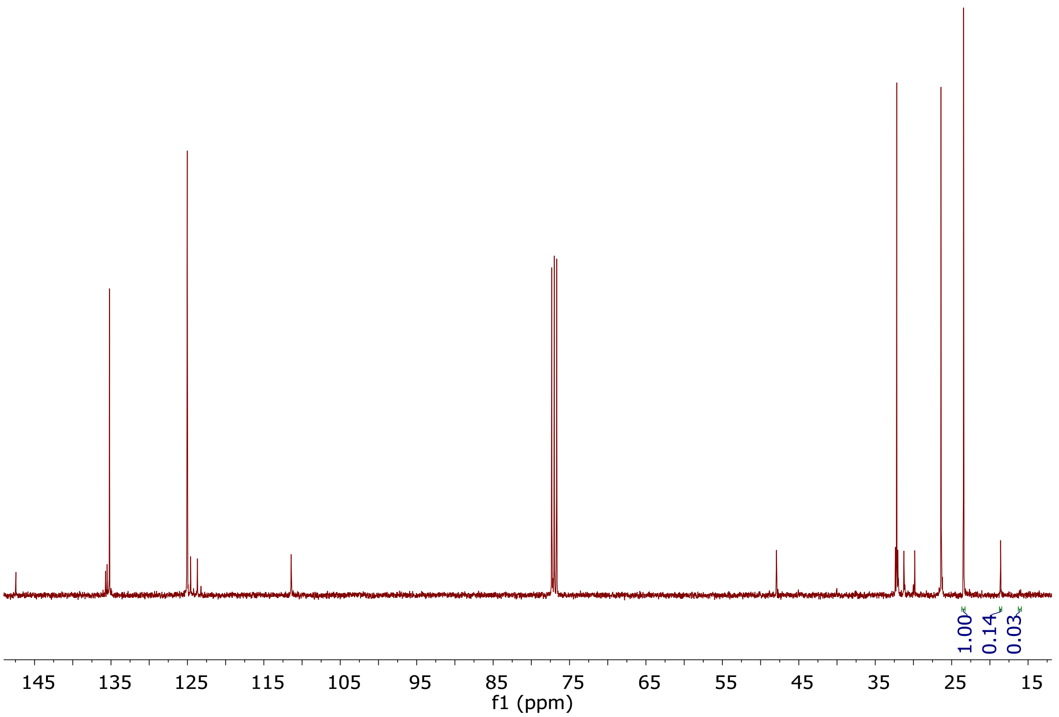

Fig. S149 ^{13}C NMR spectrum of PIP 500 equivalents generated by **$Y(CH_2SiMe_3)_3(THF)_2$**, 2 equivalents $[Ph_3C][B(C_6F_5)_4]$, and 10 equivalents Al $^{i}Bu_3$ from **Table 4**, entry 8 in $CDCl_3$ at 298 K (30 min).

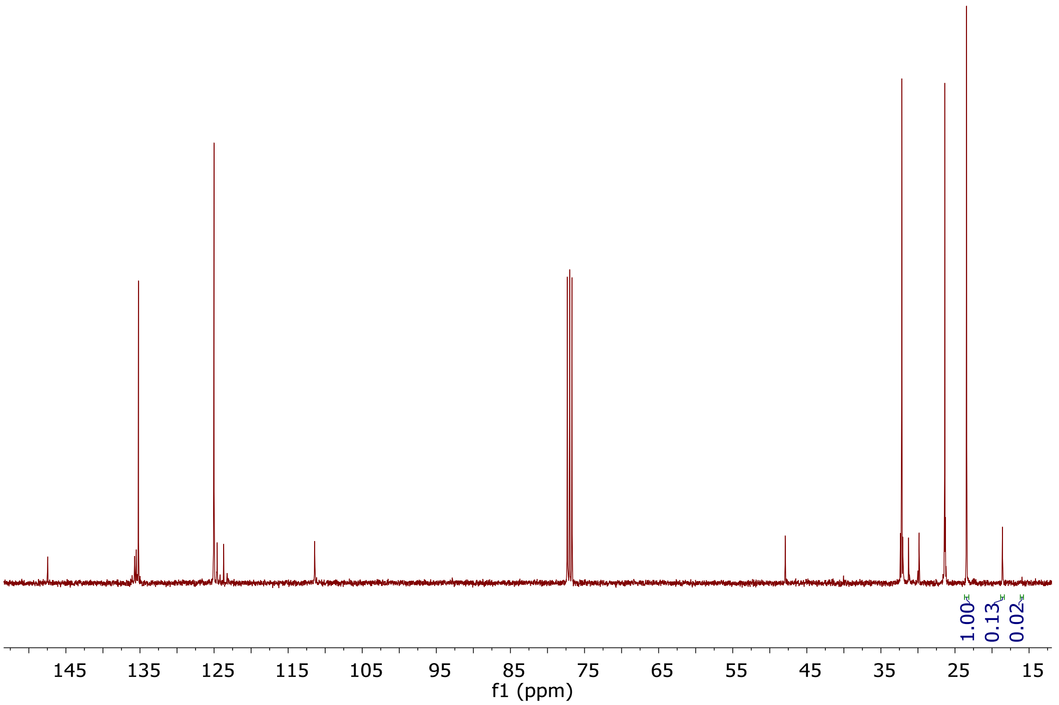

Fig. S150 ^{13}C NMR spectrum of PIP 500 equivalents generated by **$Y(CH_2SiMe_3)_3(THF)_2$**, 2 equivalents $[Ph_3C][B(C_6F_5)_4]$, and 15 equivalents Al $^{i}Bu_3$ from **Table 4**, entry 9 in $CDCl_3$ at 298 K (30 min).

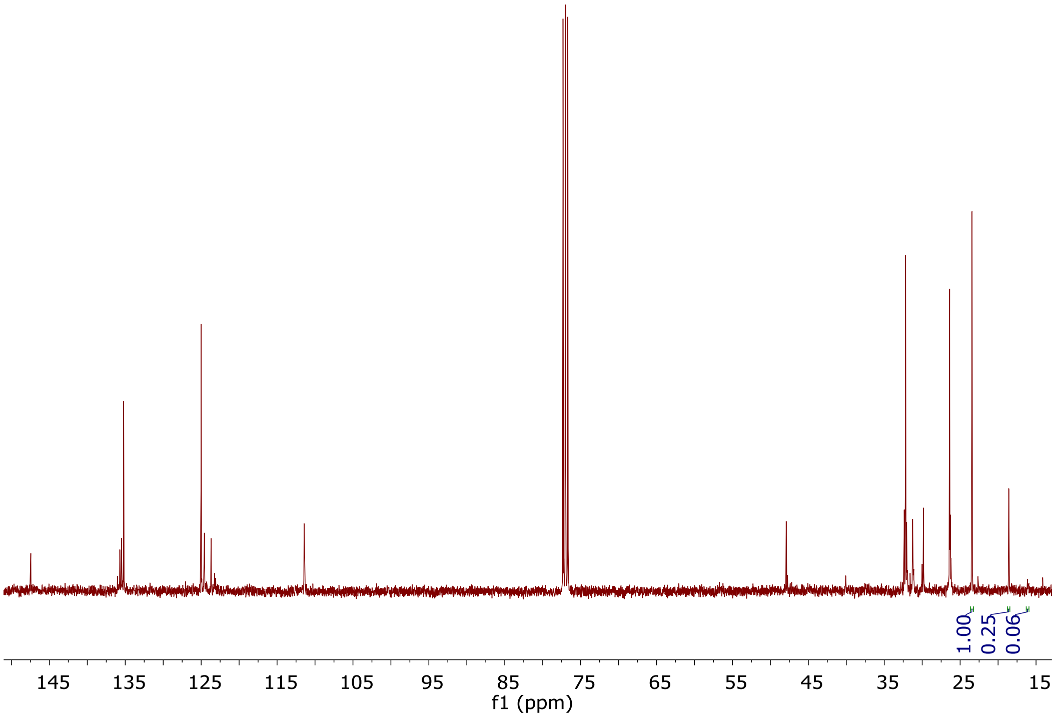

Fig. S151 ^{13}C NMR spectrum of PIP 500 equivalents generated by **Y(CH$_2$SiMe$_3$)$_3$(THF)$_2$**, 2 equivalents [Ph$_3$C][B(C$_6$F$_5$)$_4$], 1 equivalent PPh$_3$, and 5 equivalents Al ^{i}Bu$_3$ from **Table 4**, entry 10 in CDCl$_3$ at 298 K (30 min).

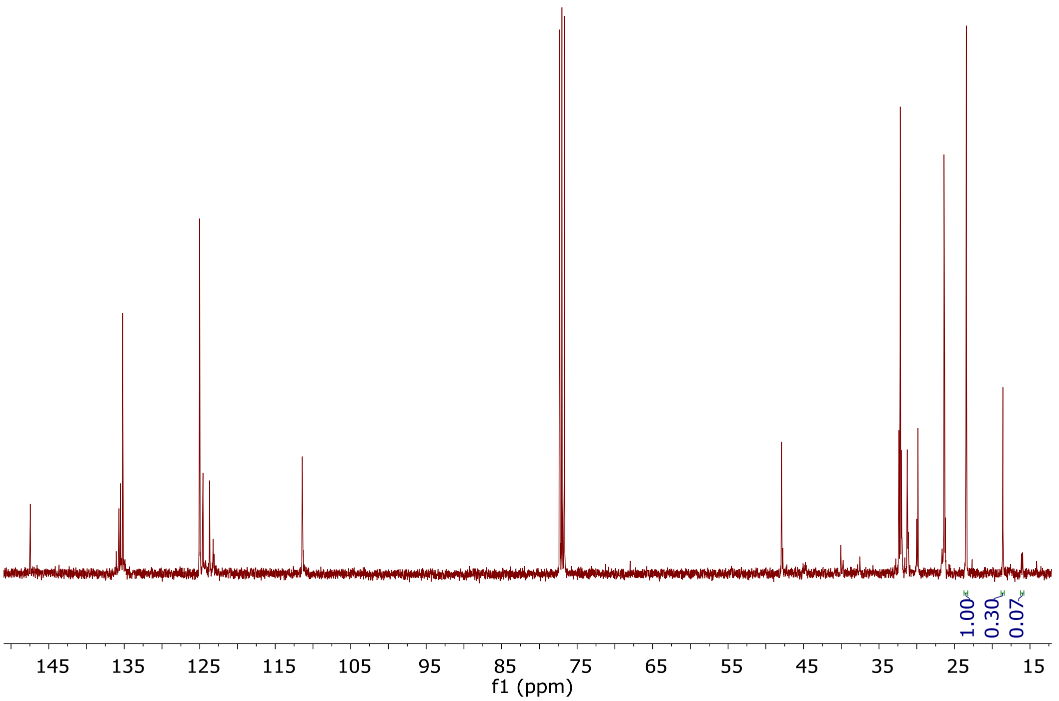

Fig. S152 ^{13}C NMR spectrum of PIP 500 equivalents generated by **Y(CH$_2$SiMe$_3$)$_3$(THF)$_2$**, 2 equivalents [Ph$_3$C][B(C$_6$F$_5$)$_4$], 1 equivalent PPh$_3$, and 10 equivalents Al ^{i}Bu$_3$ from **Table 4**, entry 11 in CDCl$_3$ at 298 K (30 min).

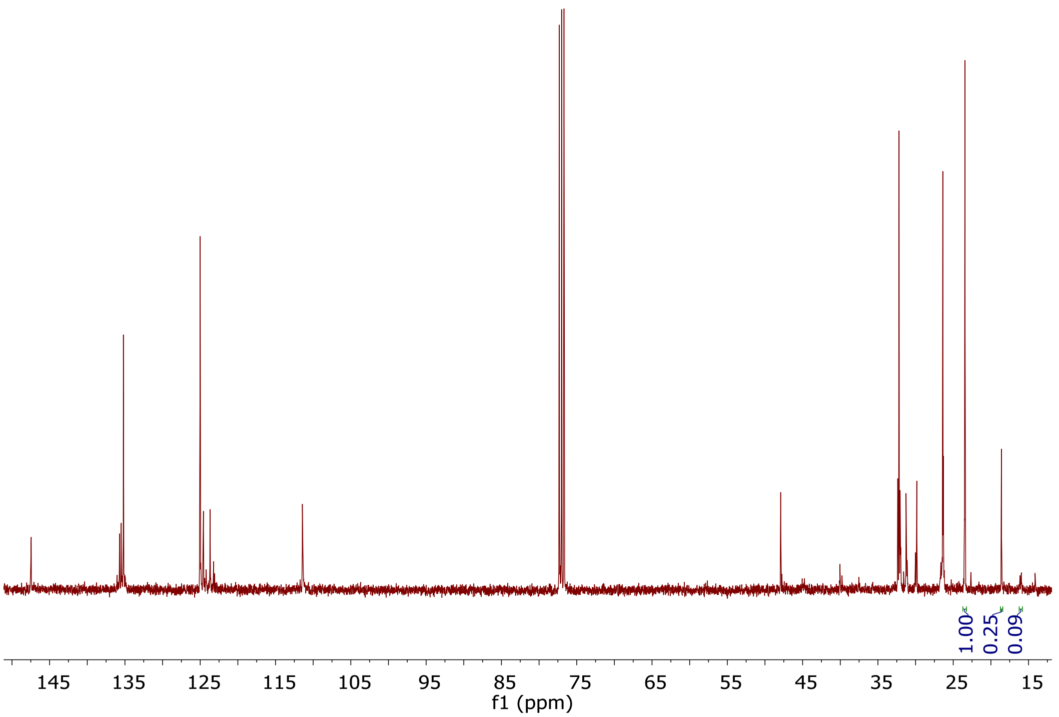

Fig. S153 ^{13}C NMR spectrum of PIP 500 equivalents generated by **Y(CH$_2$SiMe$_3$)$_3$(THF)$_2$**, 2 equivalents [Ph$_3$C][B(C$_6$F$_5$)$_4$], 1 equivalent PPh$_3$, and 15 equivalents Al^{i}Bu$_3$ from **Table 4**, entry 12 in CDCl$_3$ at 298 K (30 min).

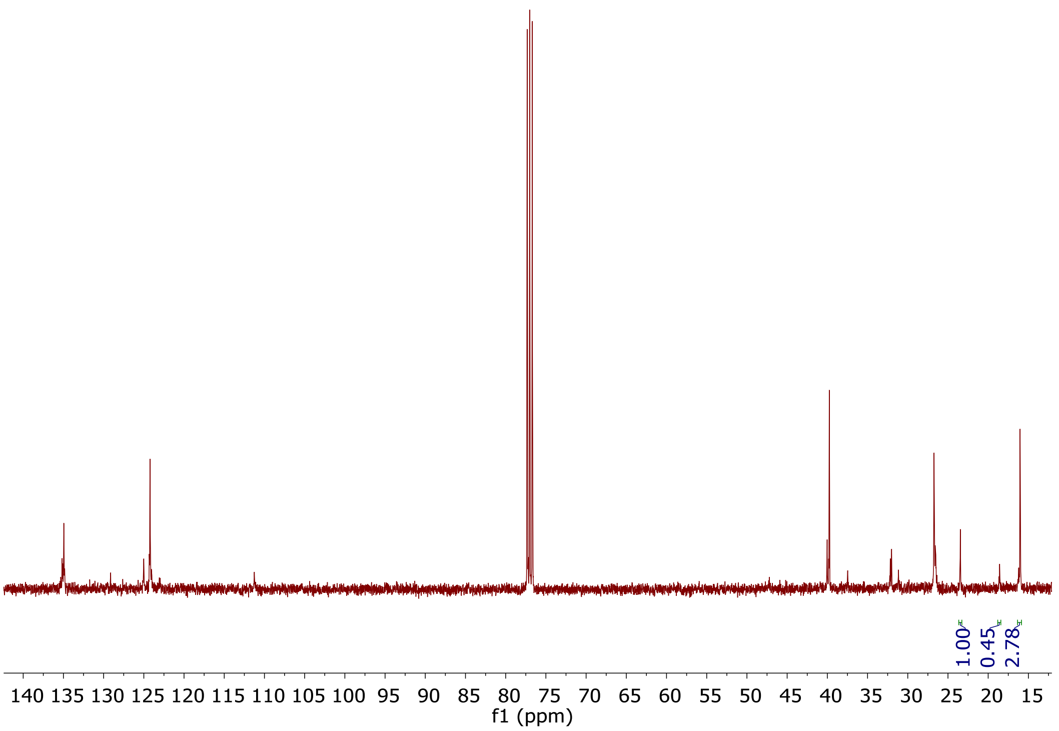

Fig. S154 ^{13}C NMR spectrum of PIP 500 equivalents generated by **Sm(CH$_2$SiMe$_3$)$_3$(THF)$_3$** and 1 equivalent [Ph$_3$C][B(C$_6$F$_5$)$_4$] from **Table 5**, entry 1 in CDCl$_3$ at 298 K (30 min).

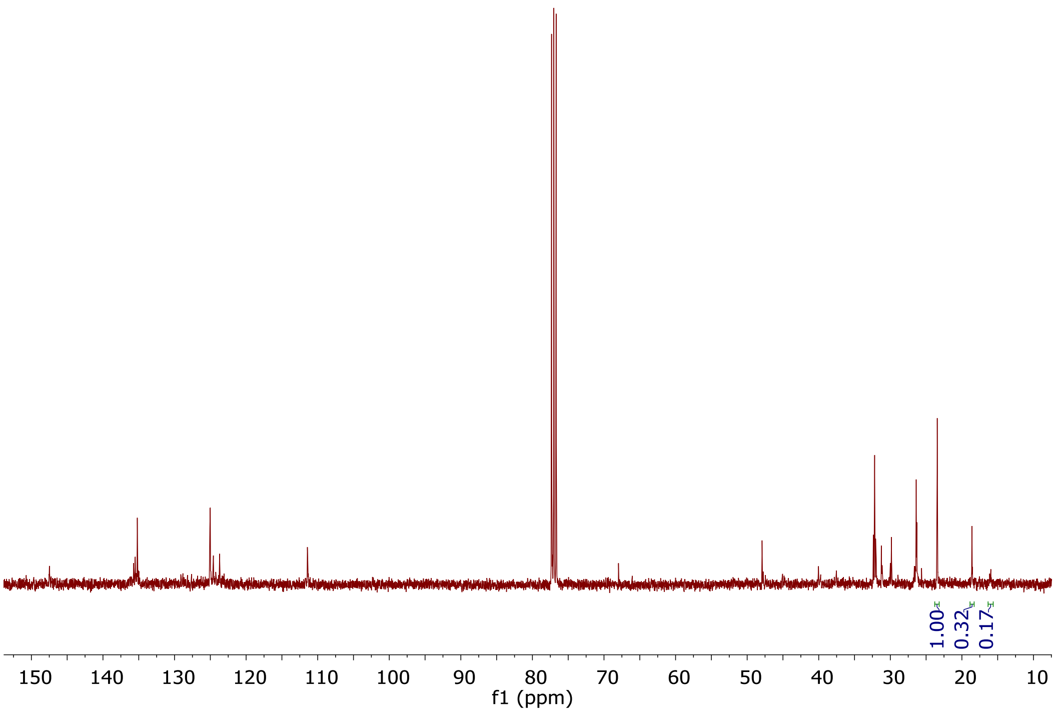

Fig. S155 ^{13}C NMR spectrum of PIP 500 equivalents generated by **$Sm(CH_2SiMe_3)_3(THF)_3$** and 2 equivalents $[Ph_3C][B(C_6F_5)_4]$ from **Table 5**, entry 2 in $CDCl_3$ at 298 K (30 min).

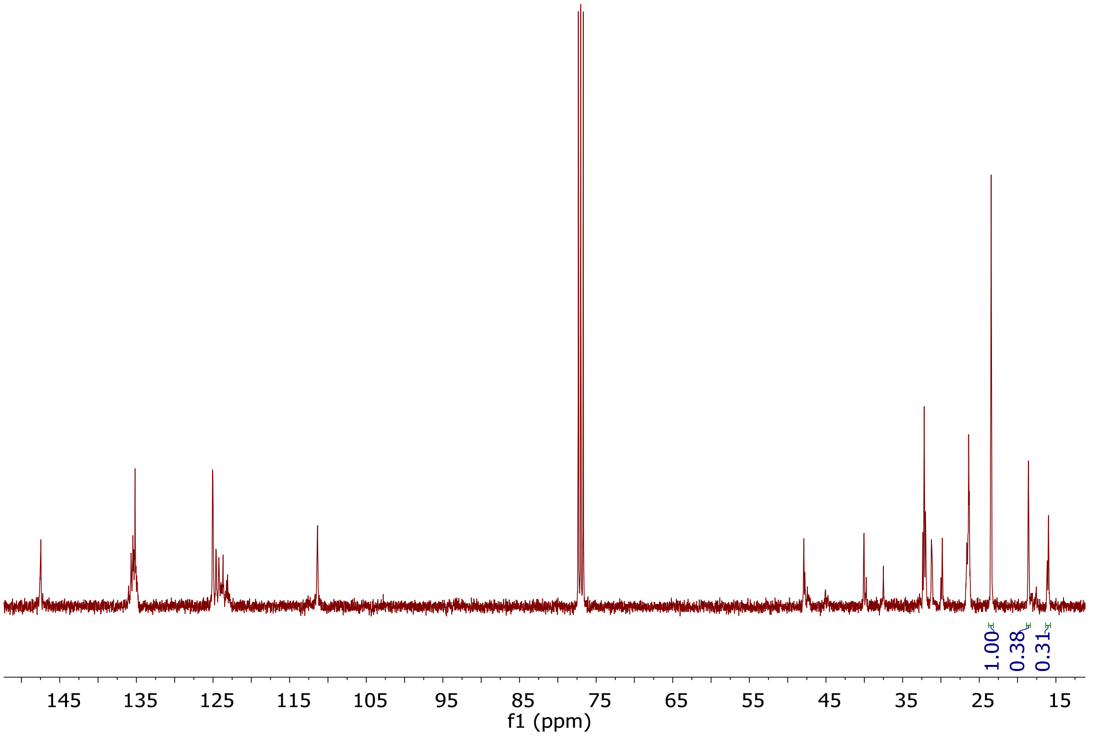

Fig. S156 ^{13}C NMR spectrum of PIP 500 equivalents generated by **$Gd(CH_2SiMe_3)_3(THF)_2$** and 1 equivalent $[Ph_3C][B(C_6F_5)_4]$ from **Table 5**, entry 3 in $CDCl_3$ at 298 K (30 min).

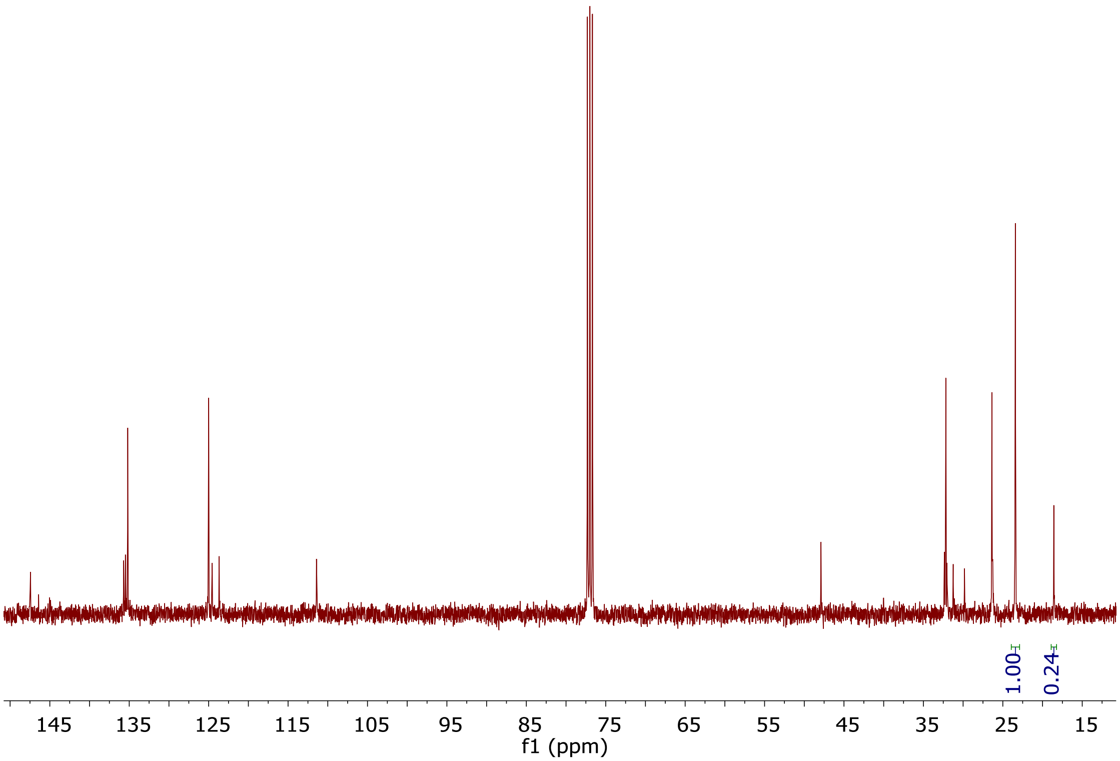

Fig. S157 ^{13}C NMR spectrum of PIP 500 equivalents generated by **Gd(CH$_2$SiMe$_3$)$_3$(THF)$_2$** and 2 equivalents [Ph$_3$C][B(C$_6$F$_5$)$_4$] from **Table 5**, entry 4 in CDCl$_3$ at 298 K (30 min).

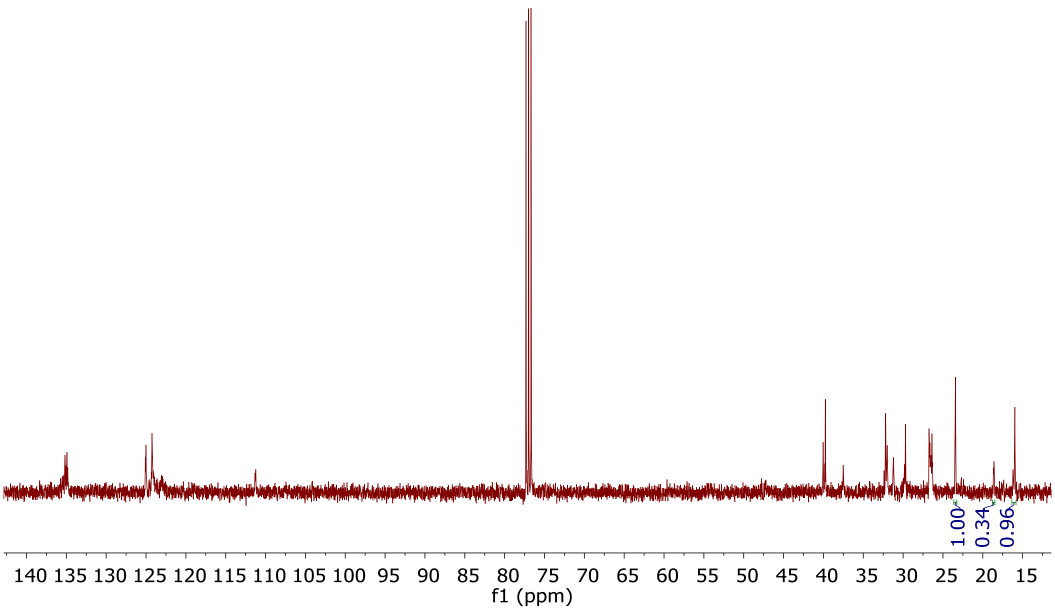

Fig. S158 ^{13}C NMR spectrum of PIP 500 equivalents generated by **Y(CH$_2$SiMe$_3$)$_3$(THF)$_2$** and 1 equivalent [Ph$_3$C][B(C$_6$F$_5$)$_4$] from **Table 5**, entry 5 in CDCl$_3$ at 298 K (30 min).

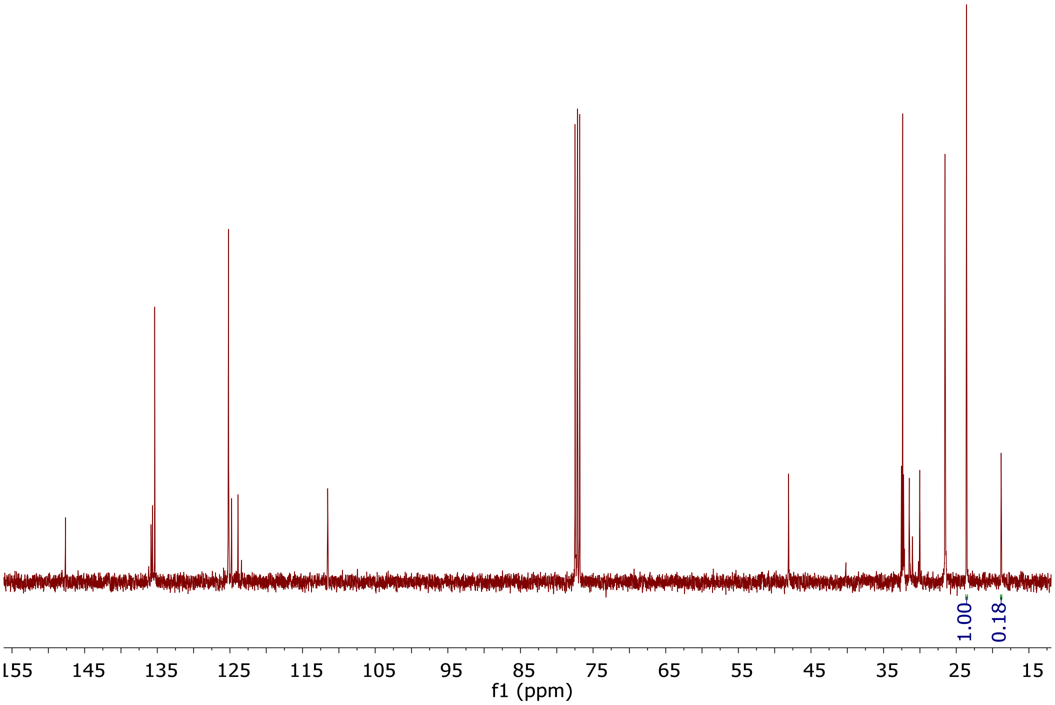

Fig. S159 ^{13}C NMR spectrum of PIP 500 equivalents generated by **$Y(CH_2SiMe_3)_3(THF)_2$** and 2 equivalents $[Ph_3C][B(C_6F_5)_4]$ from **Table 5**, entry 6 in $CDCl_3$ at 298 K (30 min).

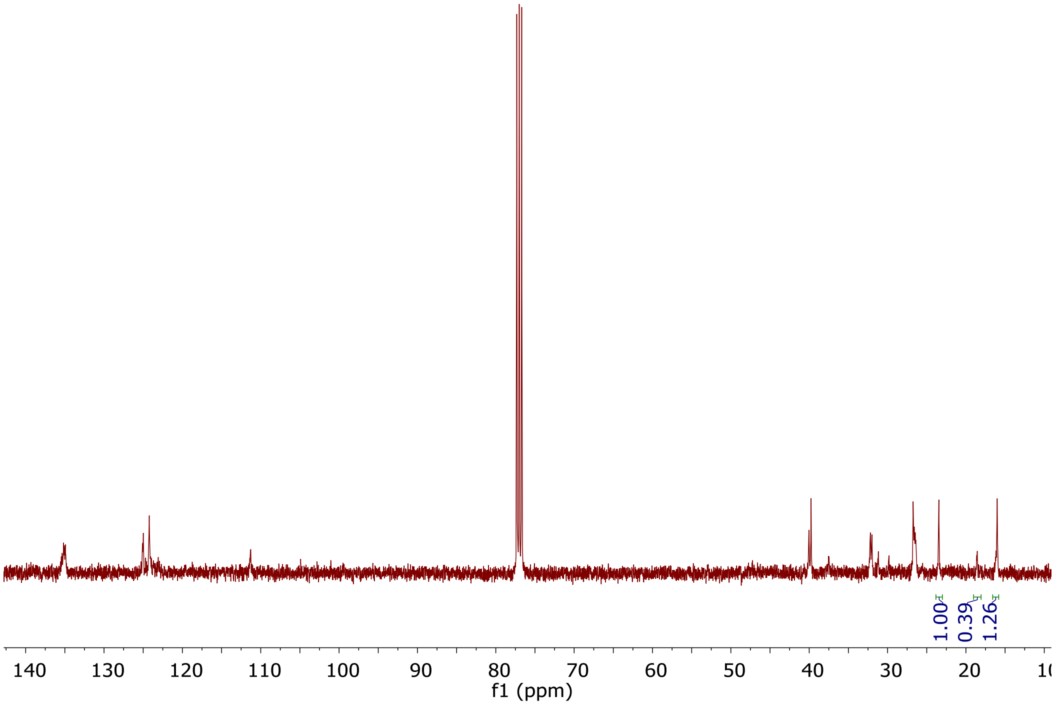

Fig. S160 ^{13}C NMR spectrum of PIP 500 equivalents generated by **$Tm(CH_2SiMe_3)_3(THF)_2$** and 1 equivalent $[Ph_3C][B(C_6F_5)_4]$ from **Table 5**, entry 7 in $CDCl_3$ at 298 K (30 min).

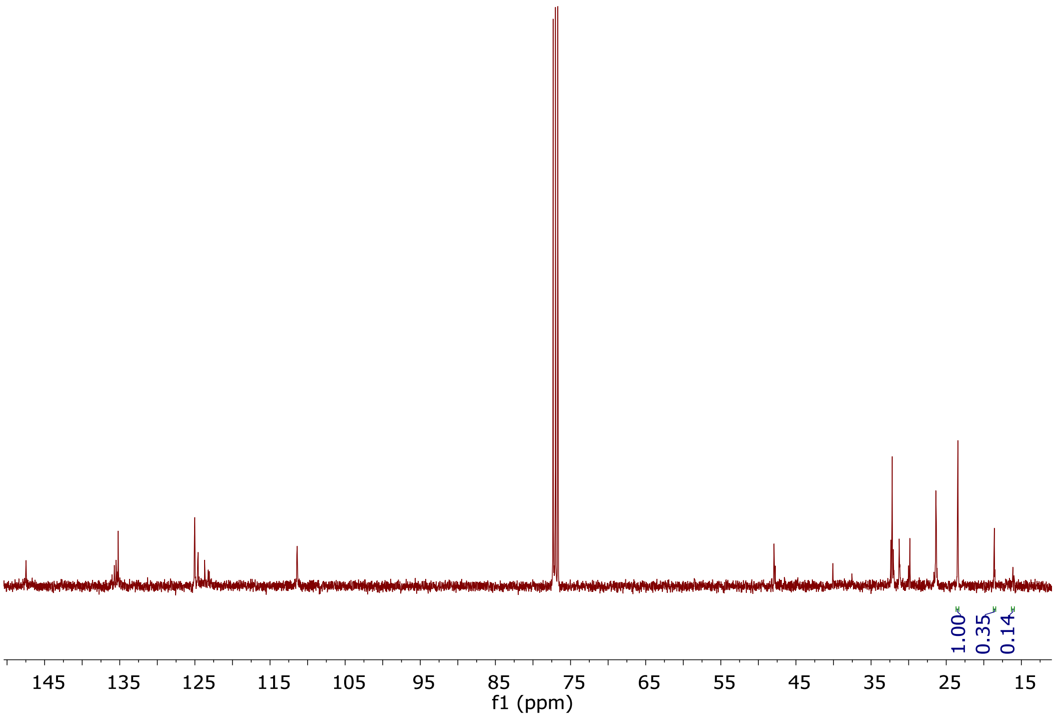

Fig. S161 ^{13}C NMR spectrum of PIP 500 equivalents generated by **$Tm(CH_2SiMe_3)_3(THF)_2$** and 2 equivalents $[Ph_3C][B(C_6F_5)_4]$ from **Table 5**, entry 8 in $CDCl_3$ at 298 K (30 min).

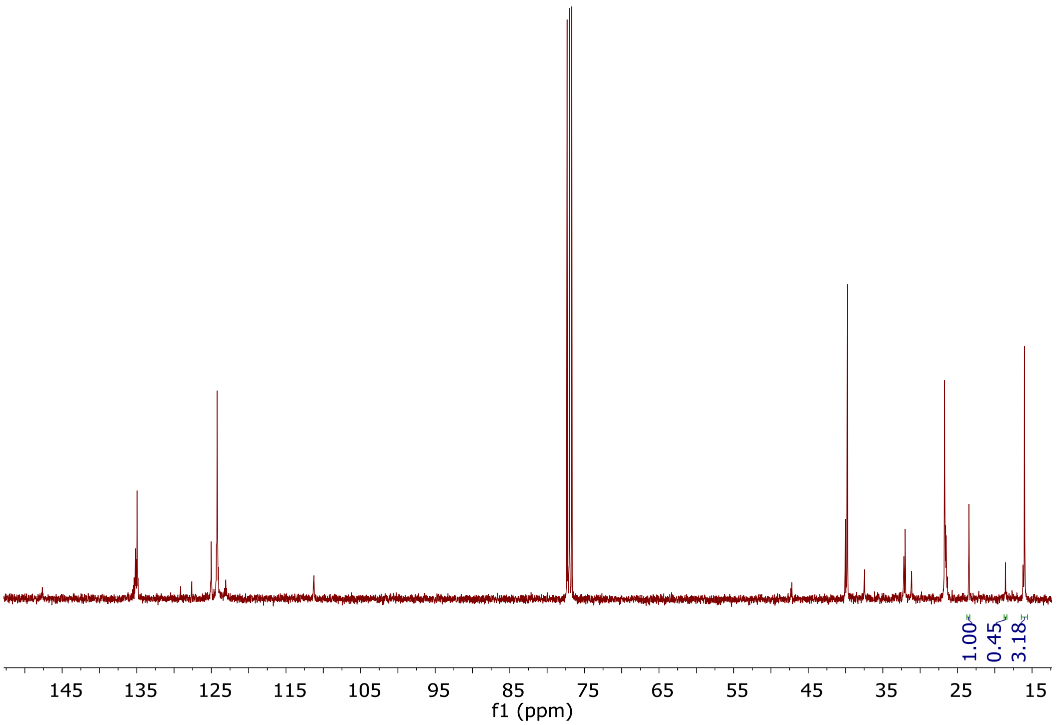

Fig. S162 ^{13}C NMR spectrum of PIP 500 equivalents generated by **$Sm(CH_2SiMe_3)_3(THF)_3$**, 1 equivalent $[Ph_3C][B(C_6F_5)_4]$, and 1 equivalent PPh_3 from **Table 5**, entry 9 in $CDCl_3$ at 298 K (30 min).

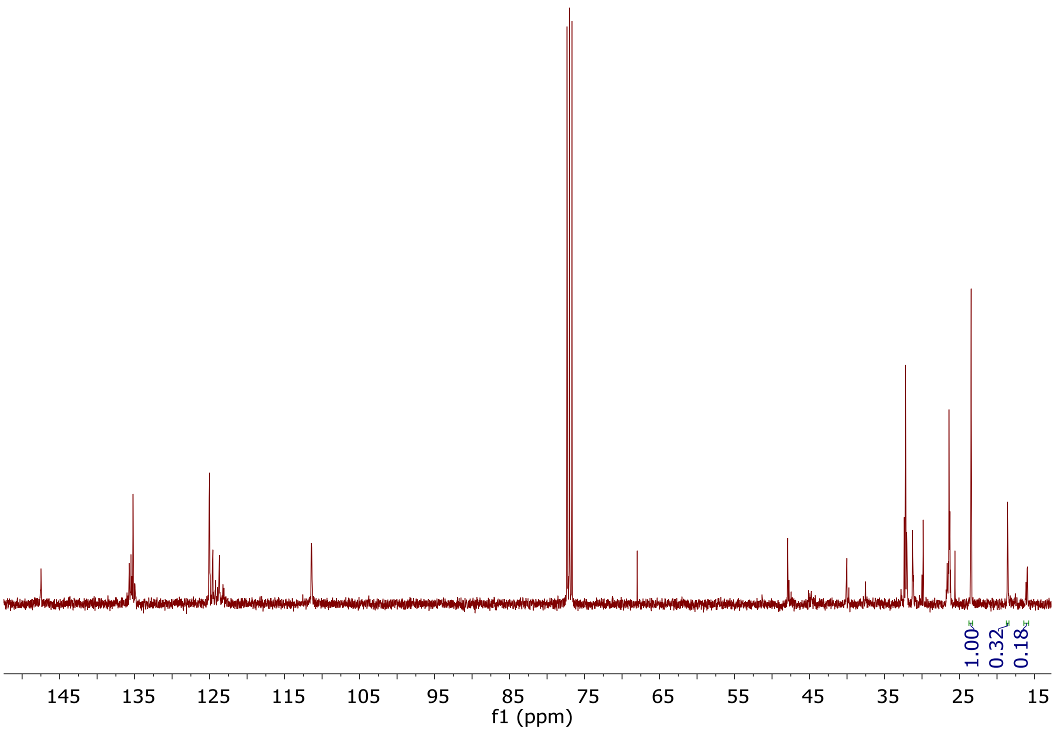

Fig. S163 ^{13}C NMR spectrum of PIP 500 equivalents generated by **$Sm(CH_2SiMe_3)_3(THF)_3$**, 2 equivalents $[Ph_3C][B(C_6F_5)_4]$, and 1 equivalent PPh_3 from **Table 5**, entry 10 in $CDCl_3$ at 298 K (30 min).

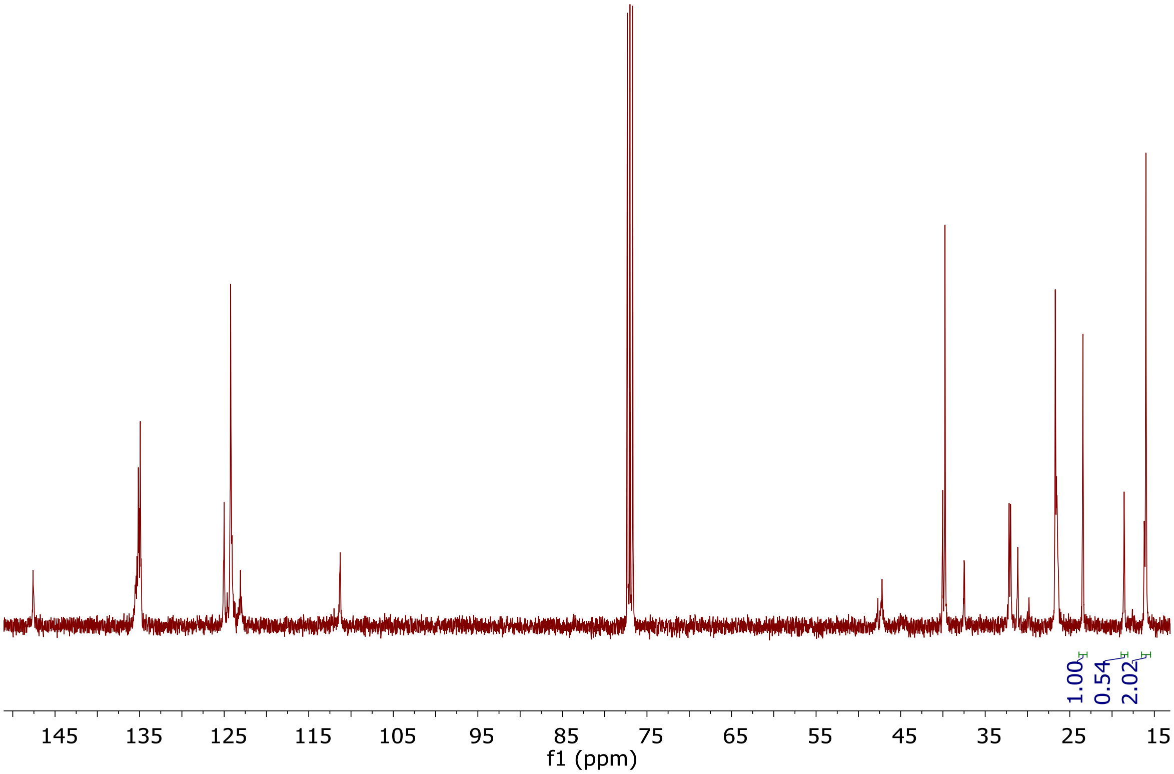

Fig. S164 ^{13}C NMR spectrum of PIP 500 equivalents generated by **$Gd(CH_2SiMe_3)_3(THF)_2$**, 1 equivalent $[Ph_3C][B(C_6F_5)_4]$, and 1 equivalent PPh_3 from **Table 5**, entry 11 in $CDCl_3$ at 298 K (30 min).

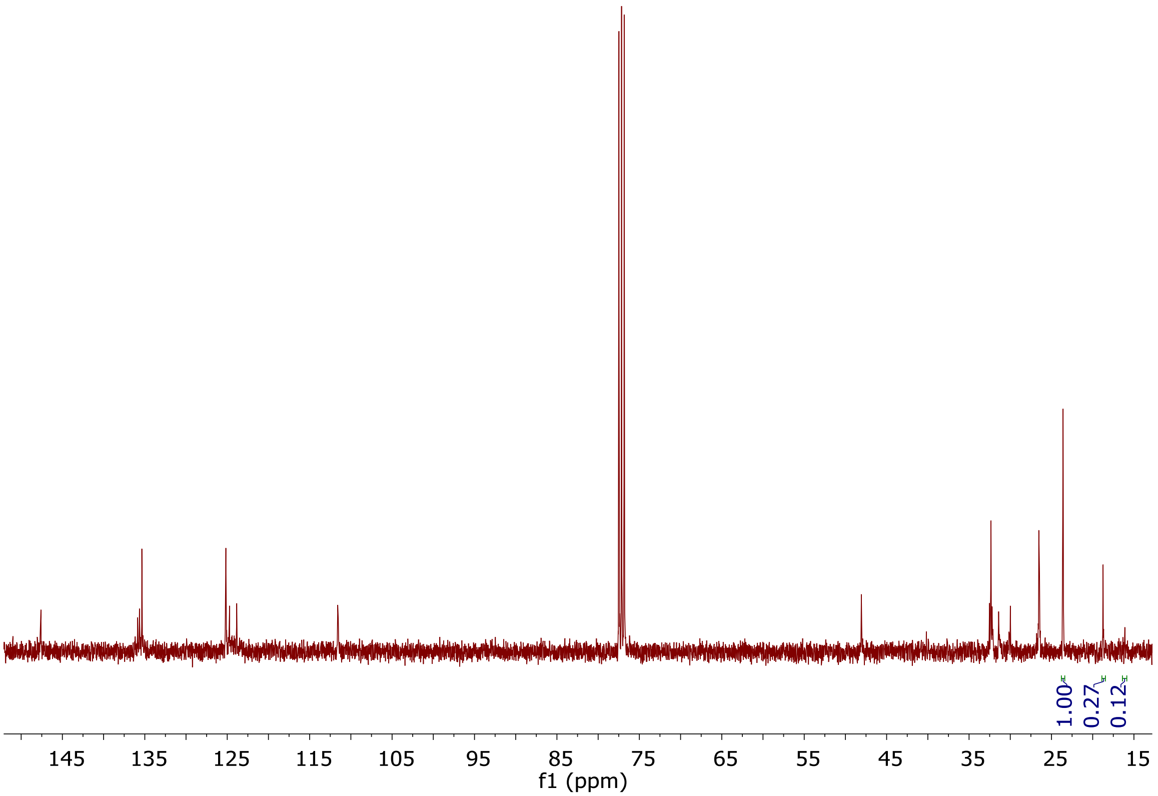

Fig. S165 ^{13}C NMR spectrum of PIP 500 equivalents generated by **$Gd(CH_2SiMe_3)_3(THF)_2$**, 2 equivalents $[Ph_3C][B(C_6F_5)_4]$, and 1 equivalent PPh_3 from **Table 5**, entry 12 in $CDCl_3$ at 298 K (30 min).

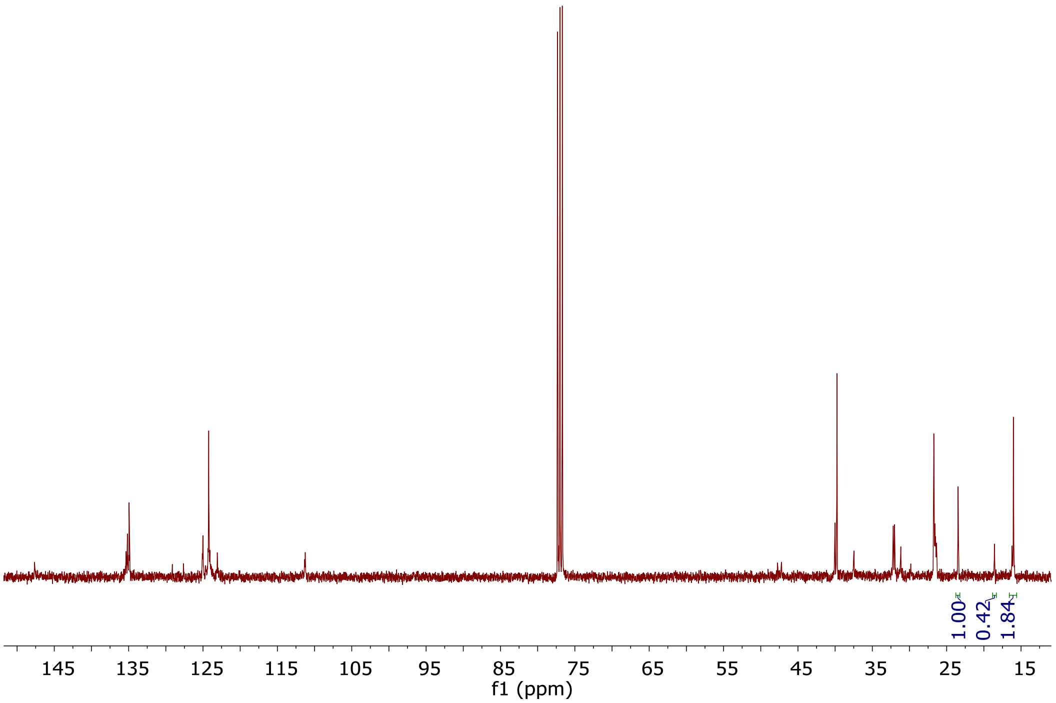

Fig. S166 ^{13}C NMR spectrum of PIP 500 equivalents generated by **$Y(CH_2SiMe_3)_3(THF)_2$**, 1 equivalent $[Ph_3C][B(C_6F_5)_4]$, and 1 equivalent PPh_3 from **Table 5**, entry 13 in $CDCl_3$ at 298 K (30 min).

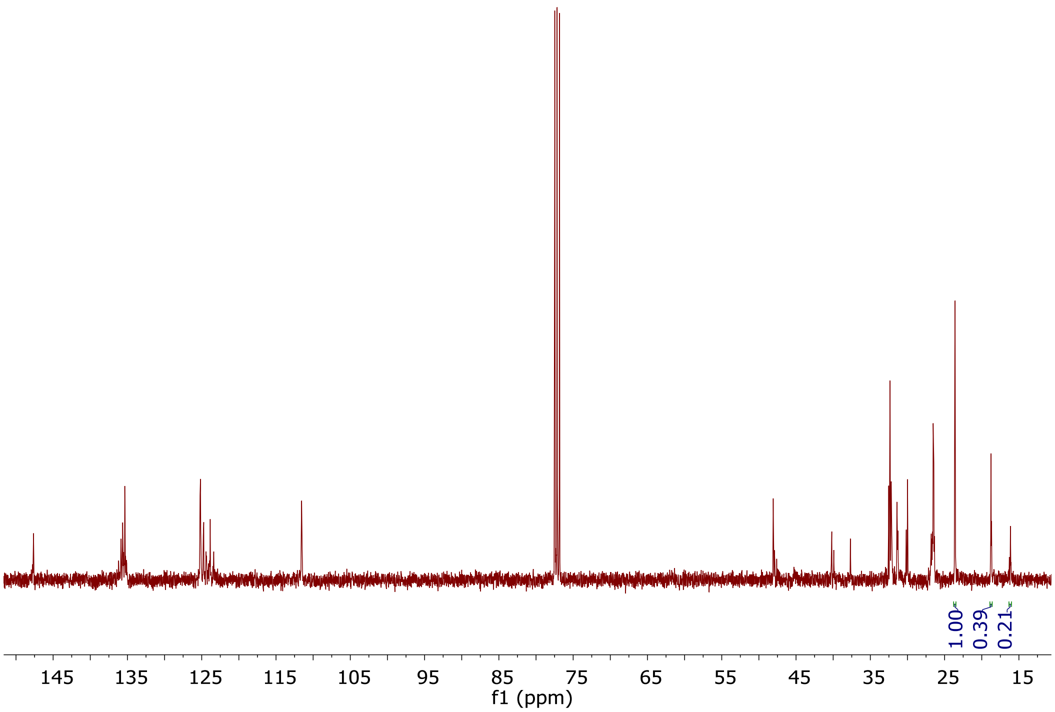

Fig. S167 ^{13}C NMR spectrum of PIP 500 equivalents generated by **$Y(CH_2SiMe_3)_3(THF)_2$**, 2 equivalents $[Ph_3C][B(C_6F_5)_4]$, and 1 equivalent PPh_3 from **Table 5**, entry 14 in $CDCl_3$ at 298 K (30 min).

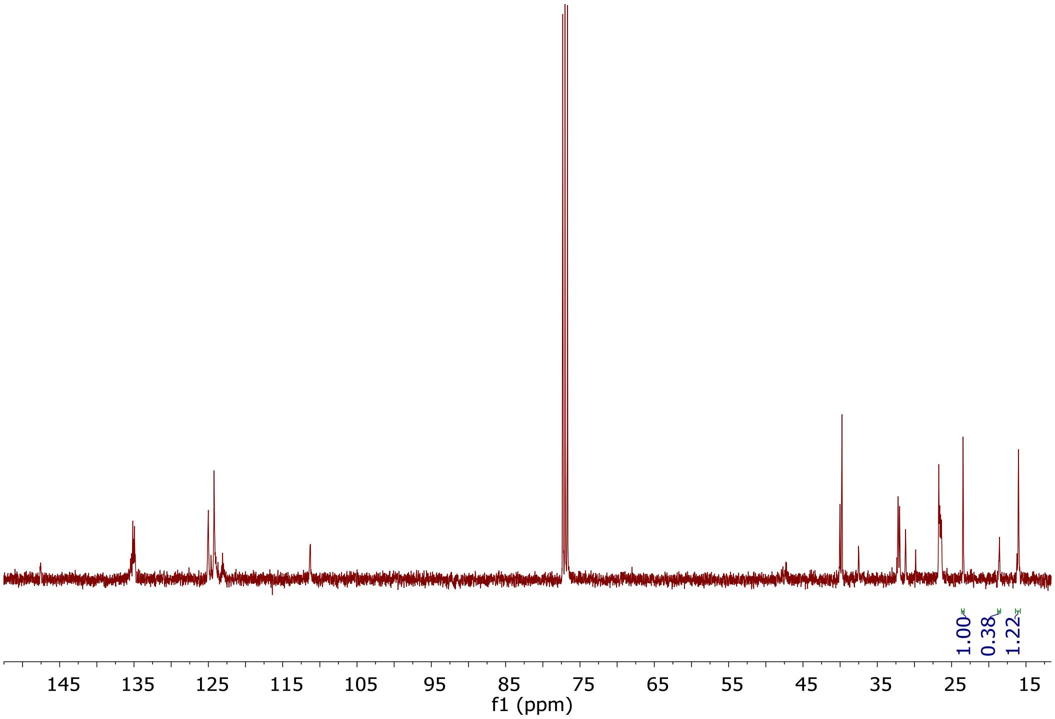

Fig. S168 ^{13}C NMR spectrum of PIP 500 equivalents generated by **$Tm(CH_2SiMe_3)_3(THF)_2$**, 1 equivalent $[Ph_3C][B(C_6F_5)_4]$, and 1 equivalent PPh_3 from **Table 5**, entry 15 in $CDCl_3$ at 298 K (30 min).

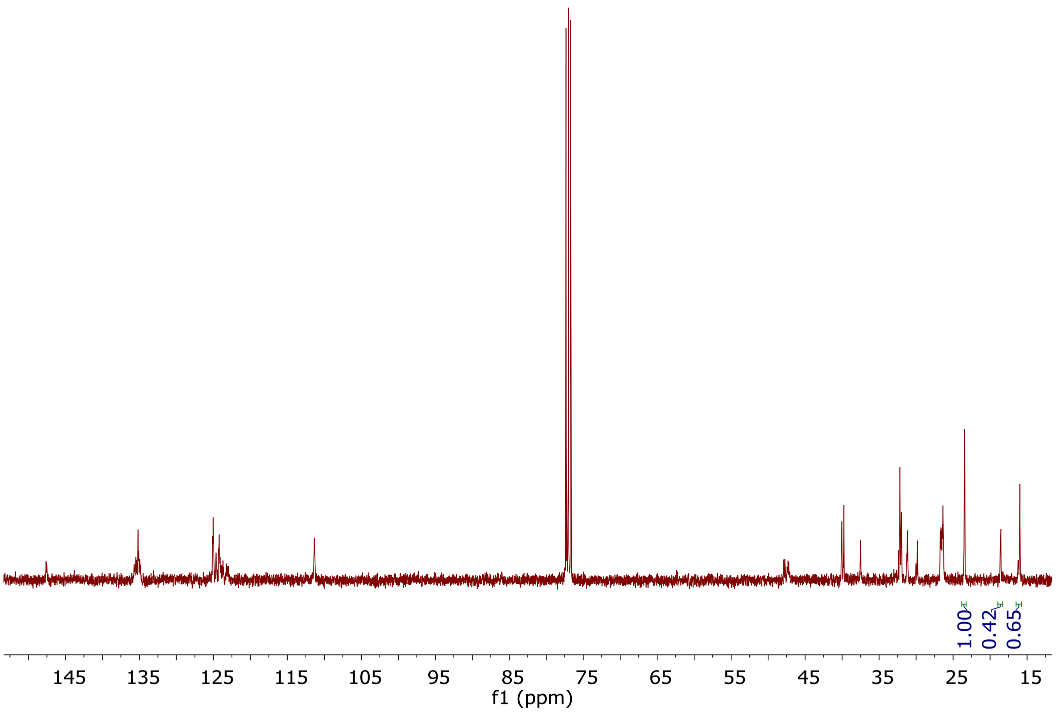

Fig. S169 ^{13}C NMR spectrum of PIP 500 equivalents generated by **$Tm(CH_2SiMe_3)_3(THF)_2$**, 2 equivalents $[Ph_3C][B(C_6F_5)_4]$, and 1 equivalent PPh_3 from **Table 5**, entry 16 in $CDCl_3$ at 298 K (30 min).

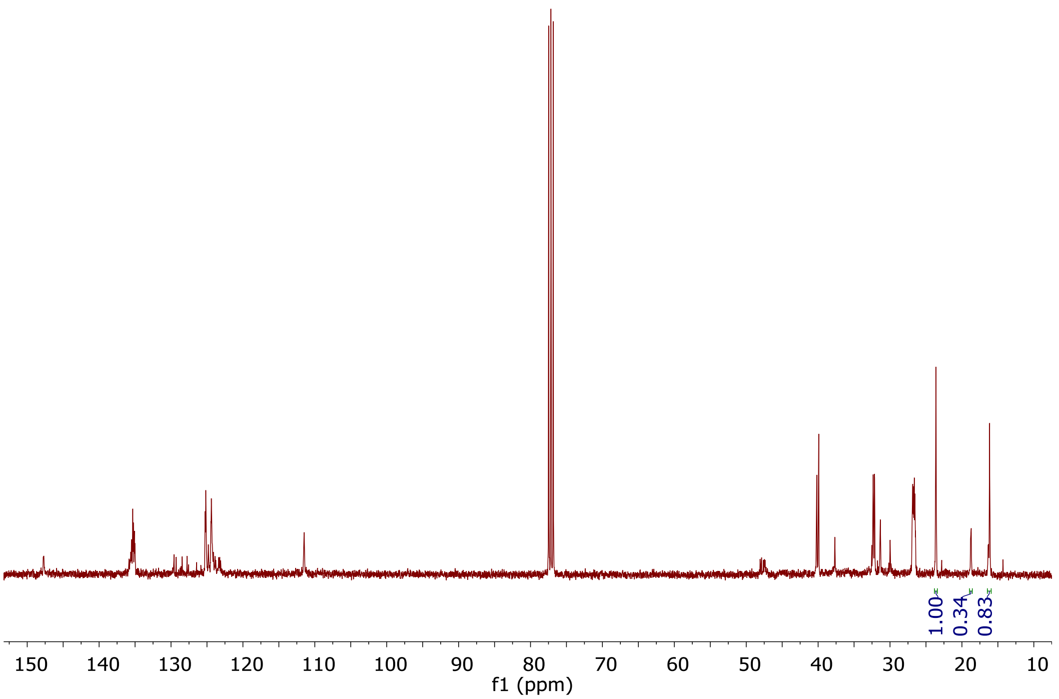

Fig. S170 ^{13}C NMR spectrum of PIP 500 equivalents generated by **$Sm(CH_2SiMe_3)_3(THF)_3$**, 1 equivalent PPh_3, and 2 equivalents $[Ph_3C][B(C_6F_5)_4]$ and from **Table 6**, entry 1 in $CDCl_3$ at 298 K ($[Ph_3C][B(C_6F_5)_4]$ addition time 0 min).

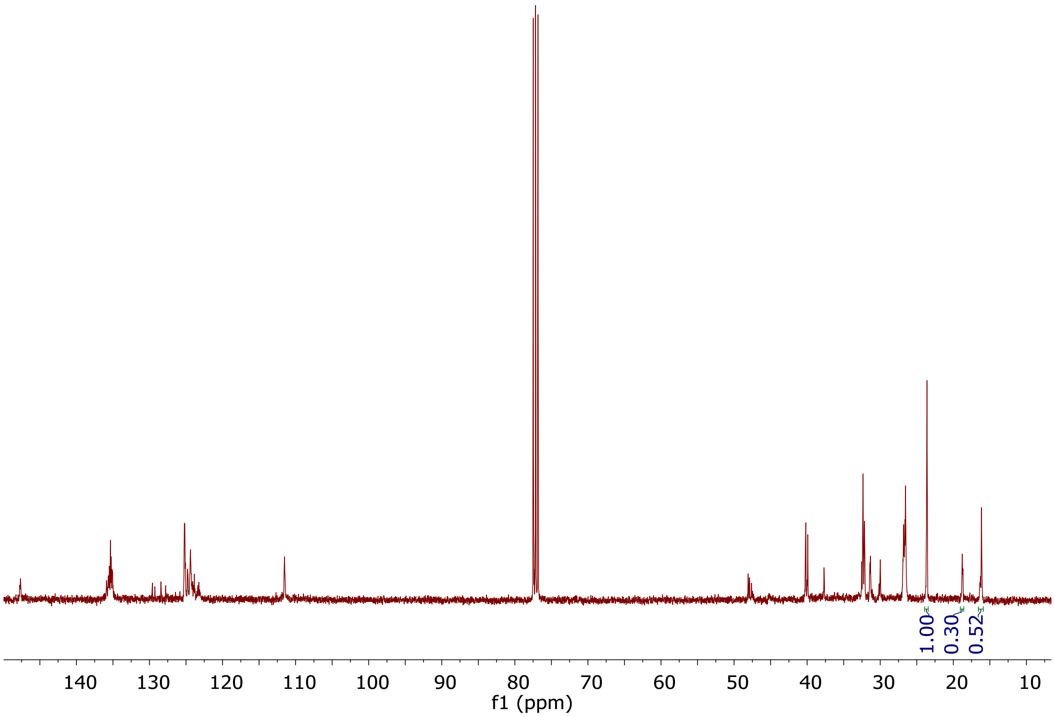

Fig. S171 ^{13}C NMR spectrum of PIP 500 equivalents generated by **$Sm(CH_2SiMe_3)_3(THF)_3$**, 1 equivalent PPh_3, and 2 equivalents $[Ph_3C][B(C_6F_5)_4]$ and from **Table 6**, entry 2 in $CDCl_3$ at 298 K ($[Ph_3C][B(C_6F_5)_4]$ addition time 10 min).

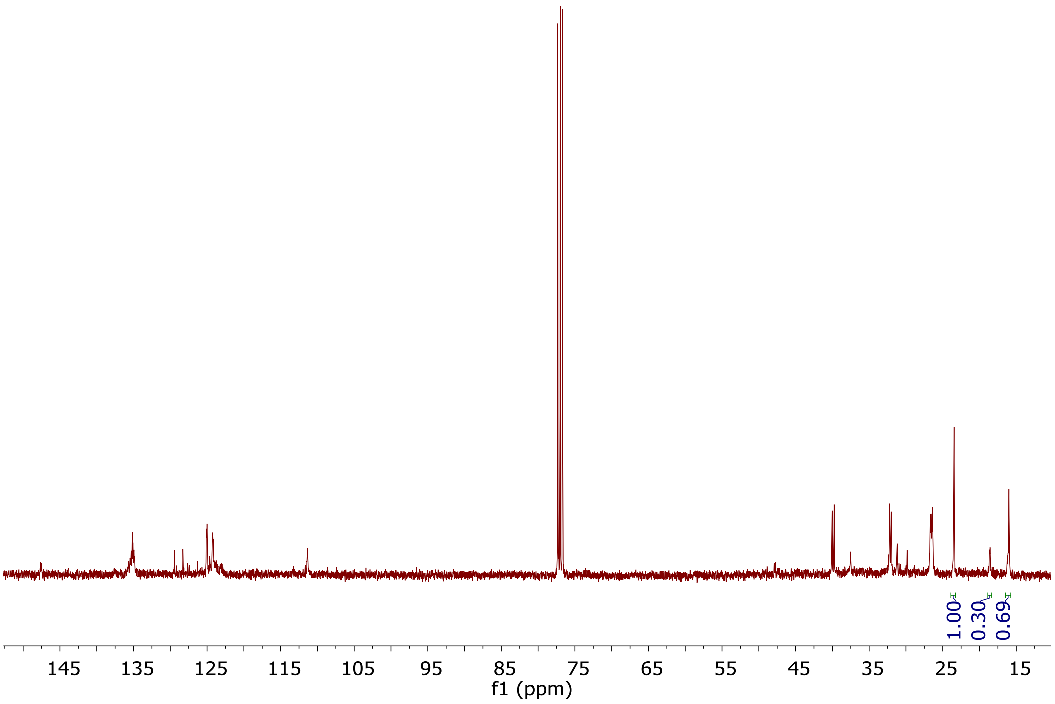

Fig. S172 ^{13}C NMR spectrum of PIP 500 equivalents generated by **$Sm(CH_2SiMe_3)_3(THF)_3$**, 1 equivalent PPh_3, and 2 equivalents $[Ph_3C][B(C_6F_5)_4]$ from **Table 6**, entry 3 in $CDCl_3$ at 298 K ($[Ph_3C][B(C_6F_5)_4]$ addition time 30 min).

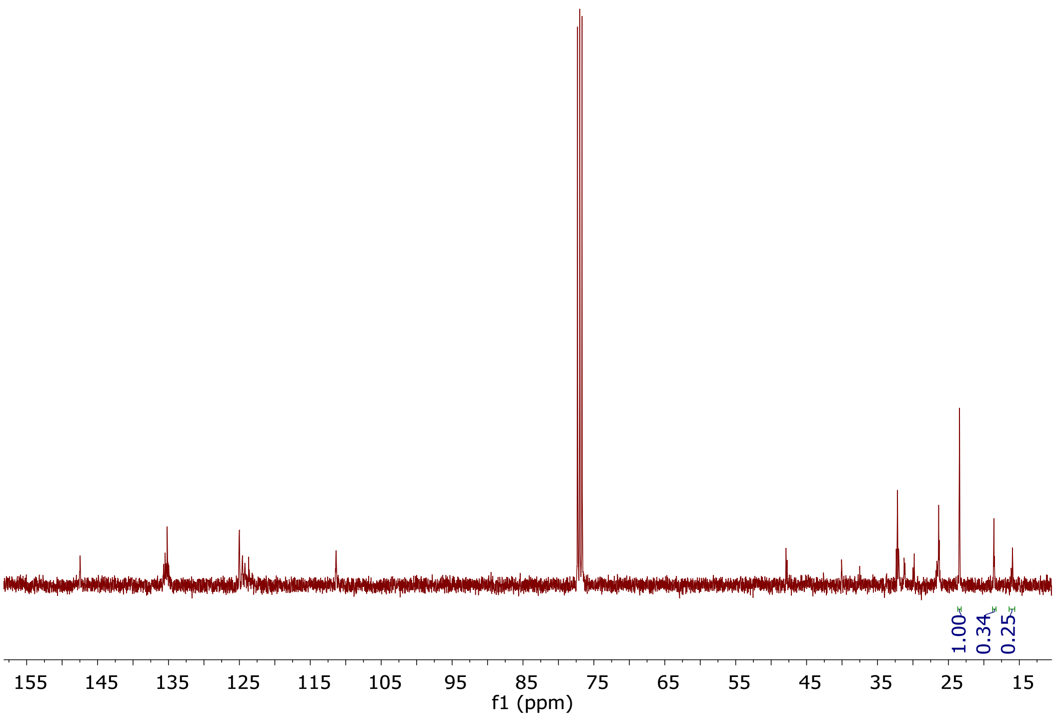

Fig. S173 ^{13}C NMR spectrum of PIP 500 equivalents generated by **Gd(CH$_2$SiMe$_3$)$_3$(THF)$_2$**, 1 equivalent PPh$_3$, and 2 equivalents [Ph$_3$C][B(C$_6$F$_5$)$_4$] from **Table 6**, entry 4 in CDCl$_3$ at 298 K ([Ph$_3$C][B(C$_6$F$_5$)$_4$] addition time 0 min).

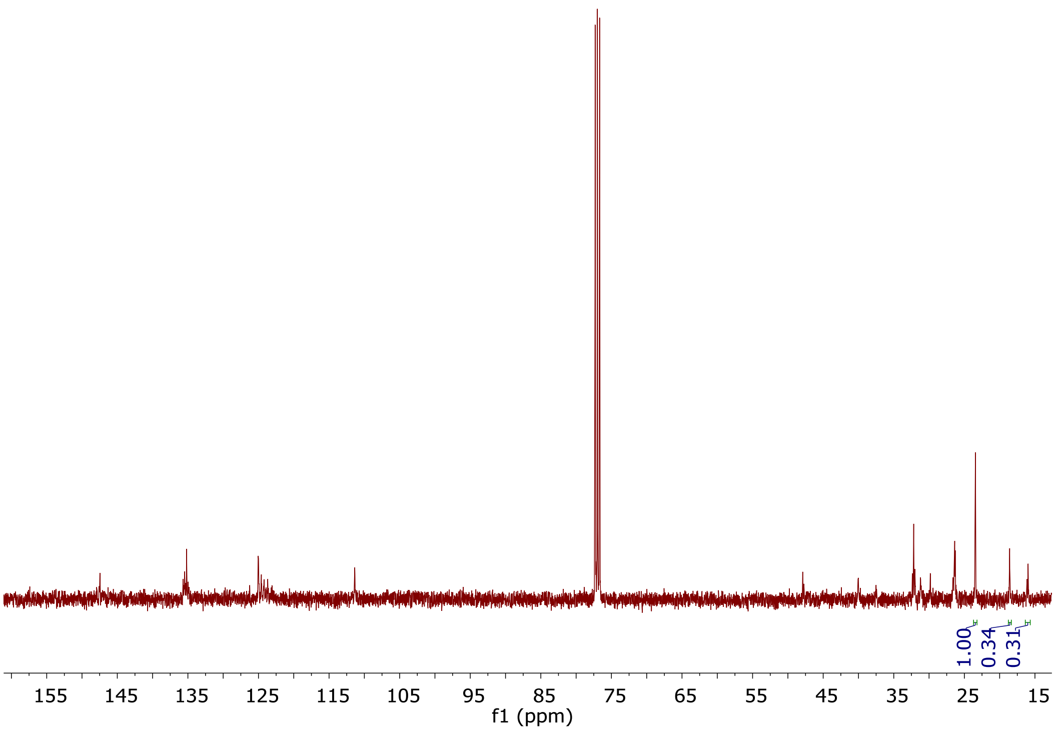

Fig. S174 ^{13}C NMR spectrum of PIP 500 equivalents generated by **Gd(CH$_2$SiMe$_3$)$_3$(THF)$_2$**, 1 equivalent PPh$_3$, and 2 equivalents [Ph$_3$C][B(C$_6$F$_5$)$_4$] from **Table 6**, entry 5 in CDCl$_3$ at 298 K ([Ph$_3$C][B(C$_6$F$_5$)$_4$] addition time 10 min).

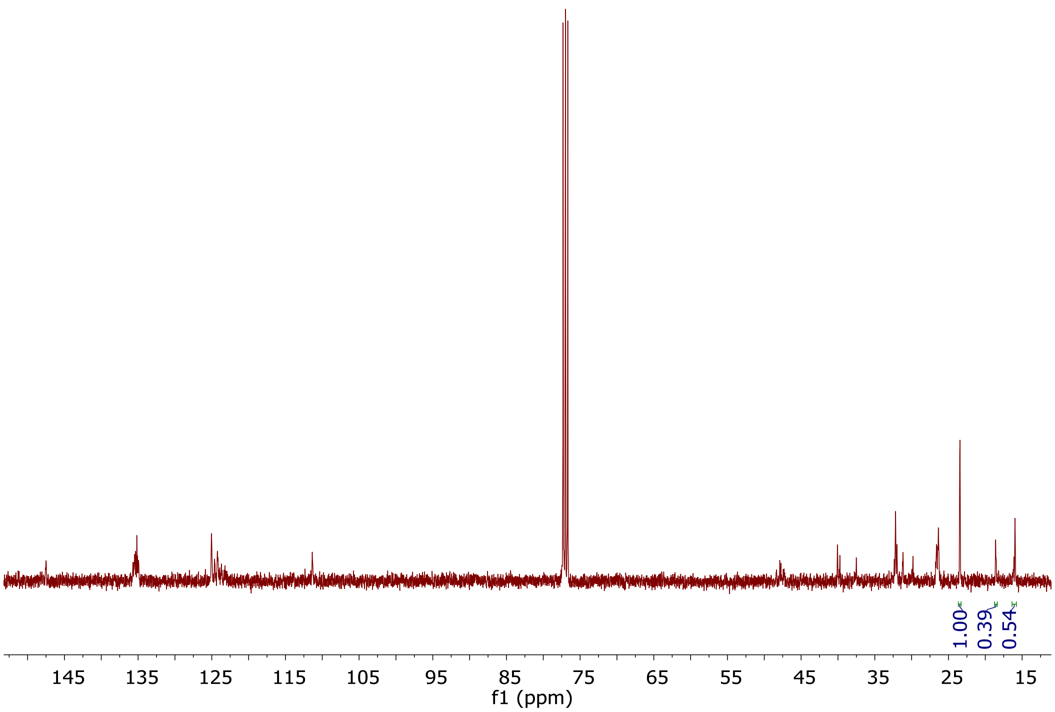

Fig. S175 ^{13}C NMR spectrum of PIP 500 equivalents generated by **Gd(CH$_2$SiMe$_3$)$_3$(THF)$_2$**, 1 equivalent PPh$_3$, and 2 equivalents [Ph$_3$C][B(C$_6$F$_5$)$_4$] from **Table 6**, entry 6 in CDCl$_3$ at 298 K ([Ph$_3$C][B(C$_6$F$_5$)$_4$] addition time 30 min).

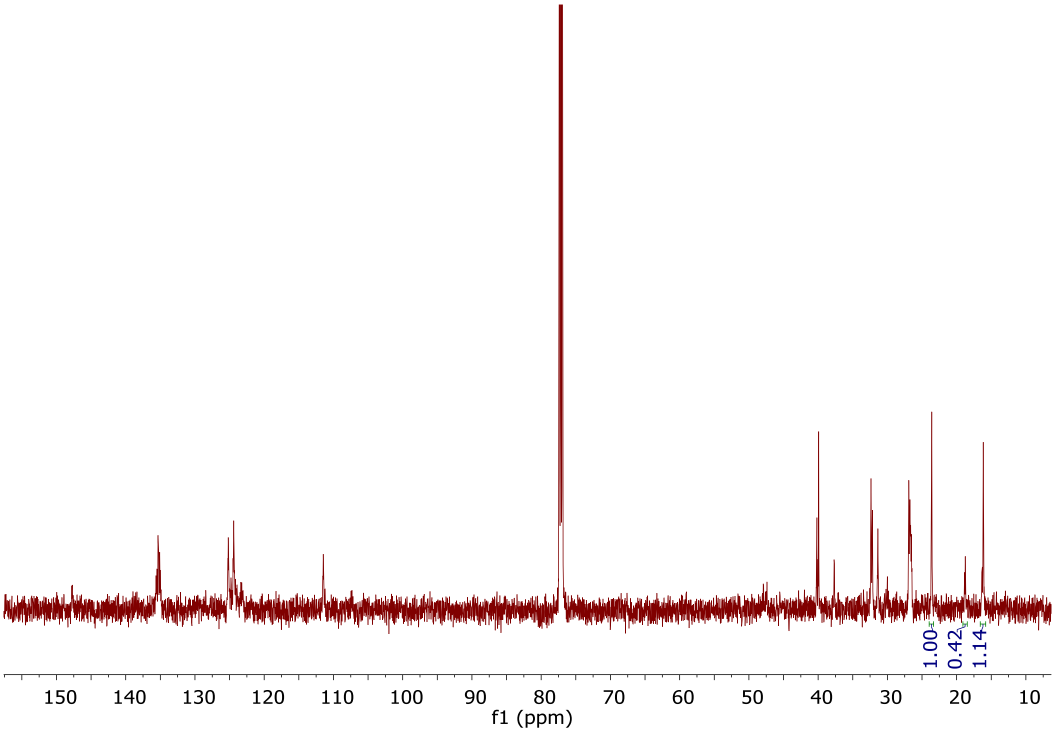

Fig. S176 ^{13}C NMR spectrum of PIP 500 equivalents generated by **Y(CH$_2$SiMe$_3$)$_3$(THF)$_2$**, 1 equivalent PPh$_3$, and 2 equivalents [Ph$_3$C][B(C$_6$F$_5$)$_4$] from **Table 6**, entry 7 in CDCl$_3$ at 298 K ([Ph$_3$C][B(C$_6$F$_5$)$_4$] addition time 0 min).

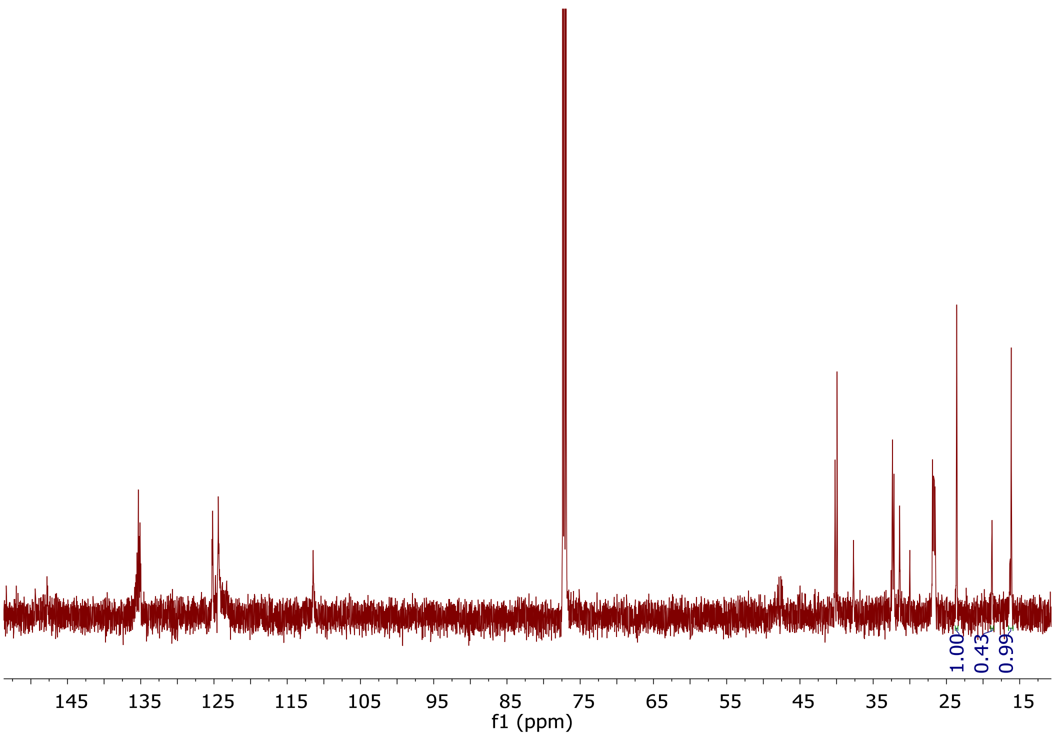

Fig. S177 ^{13}C NMR spectrum of PIP 500 equivalents generated by **$Y(CH_2SiMe_3)_3(THF)_2$**, 1 equivalent PPh_3, and 2 equivalents $[Ph_3C][B(C_6F_5)_4]$ from **Table 6**, entry 8 in $CDCl_3$ at 298 K ($[Ph_3C][B(C_6F_5)_4]$ addition time 10 min).

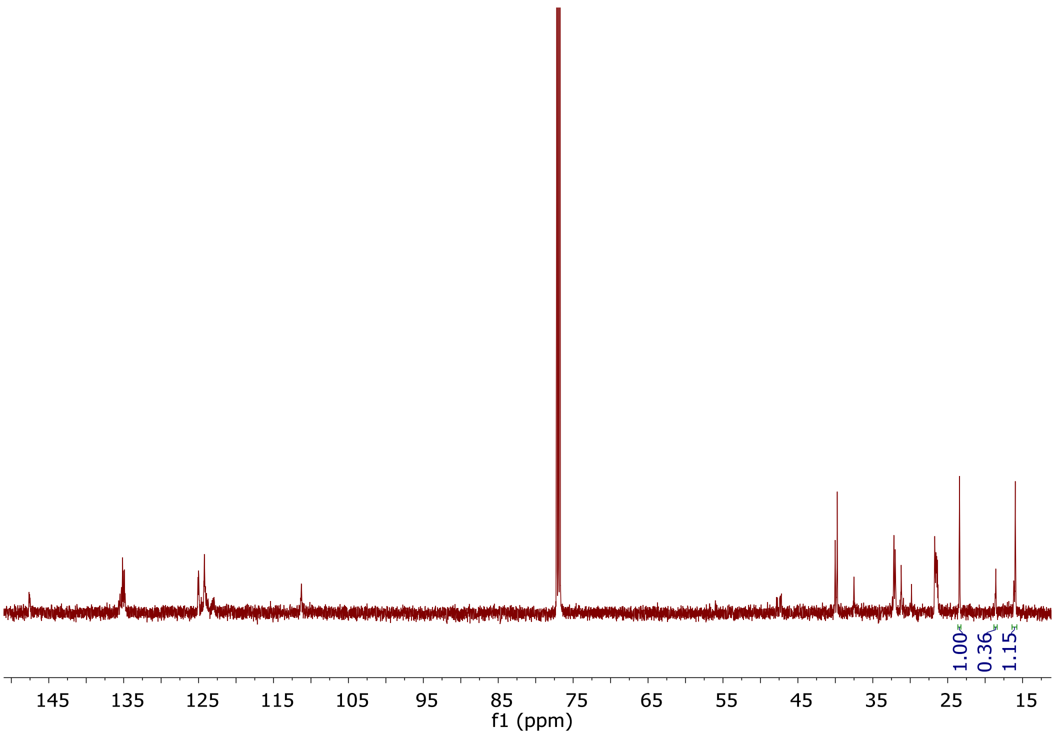

Fig. S178 ^{13}C NMR spectrum of PIP 500 equivalents generated by **$Y(CH_2SiMe_3)_3(THF)_2$**, 1 equivalent PPh_3, and 2 equivalents $[Ph_3C][B(C_6F_5)_4]$ from **Table 6**, entry 9 in $CDCl_3$ at 298 K ($[Ph_3C][B(C_6F_5)_4]$ addition time 30 min).

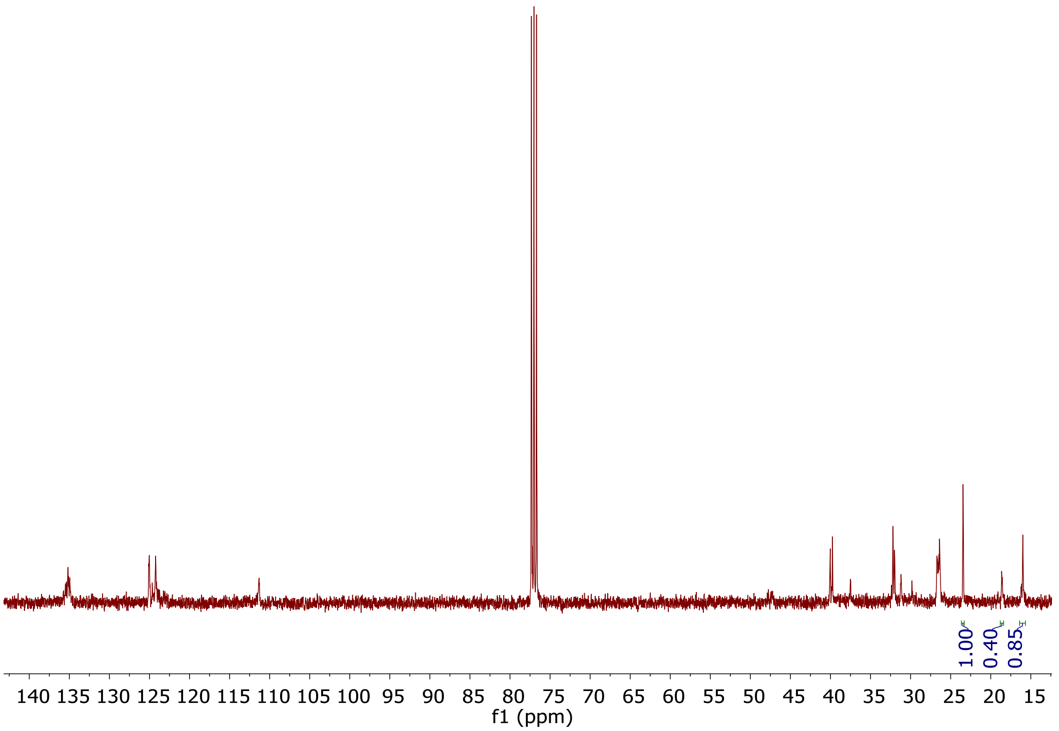

Fig. S179 ^{13}C NMR spectrum of PIP 500 equivalents generated by **$Tm(CH_2SiMe_3)_3(THF)_2$**, 1 equivalent PPh_3, and 2 equivalents $[Ph_3C][B(C_6F_5)_4]$ from **Table 6**, entry 10 in $CDCl_3$ at 298 K ($[Ph_3C][B(C_6F_5)_4]$ addition time 0 min).

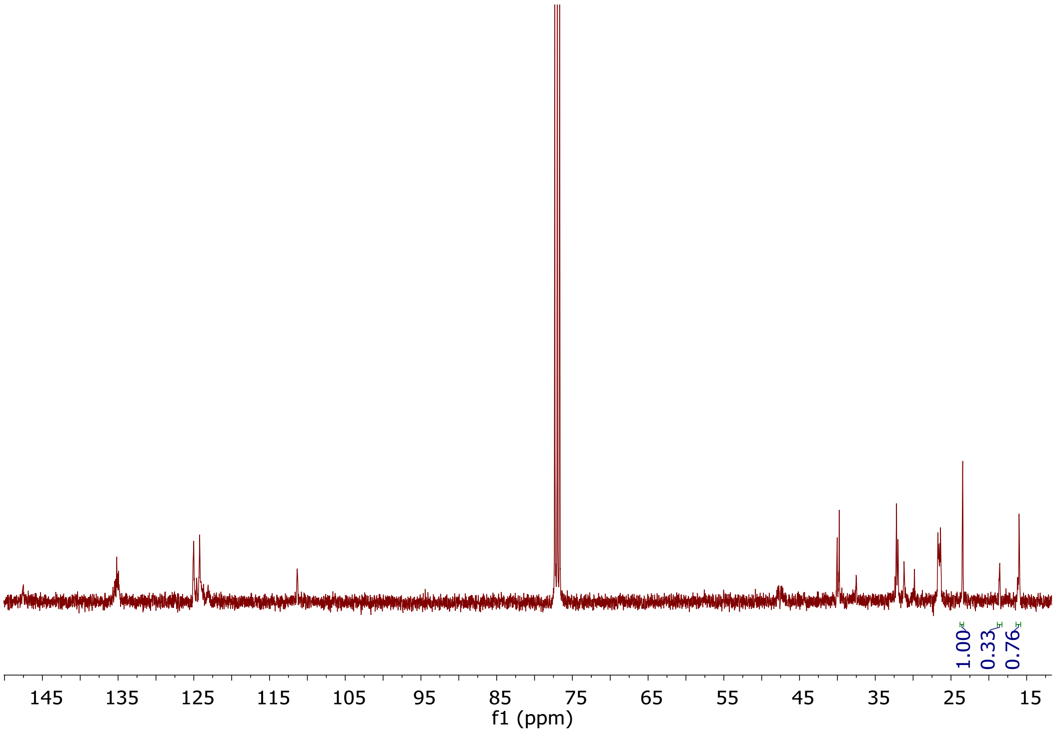

Fig. S180 ^{13}C NMR spectrum of PIP 500 equivalents generated by **$Tm(CH_2SiMe_3)_3(THF)_2$**, 1 equivalent PPh_3, and 2 equivalents $[Ph_3C][B(C_6F_5)_4]$ from **Table 6**, entry 11 in $CDCl_3$ at 298 K ($[Ph_3C][B(C_6F_5)_4]$ addition time 10 min).

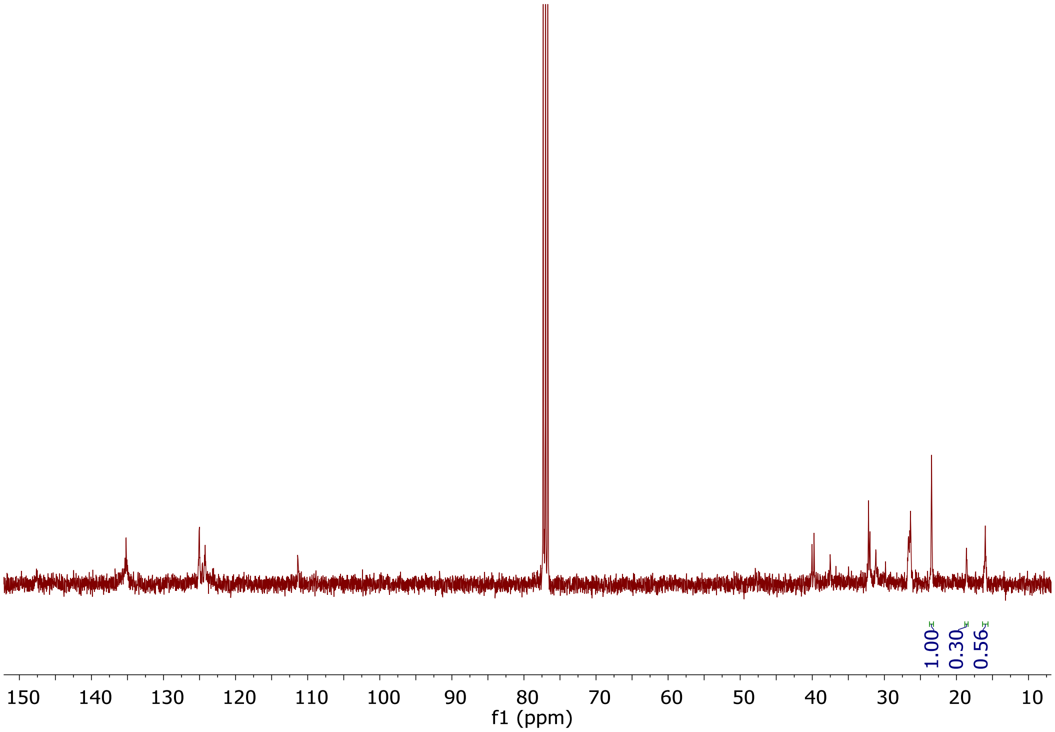

Fig. S181 ^{13}C NMR spectrum of PIP 500 equivalents generated by **$Tm(CH_2SiMe_3)_3(THF)_2$**, 1 equivalent PPh_3, and 2 equivalents $[Ph_3C][B(C_6F_5)_4]$ from **Table 6**, entry 12 in $CDCl_3$ at 298 K ($[Ph_3C][B(C_6F_5)_4]$ addition time 30 min).

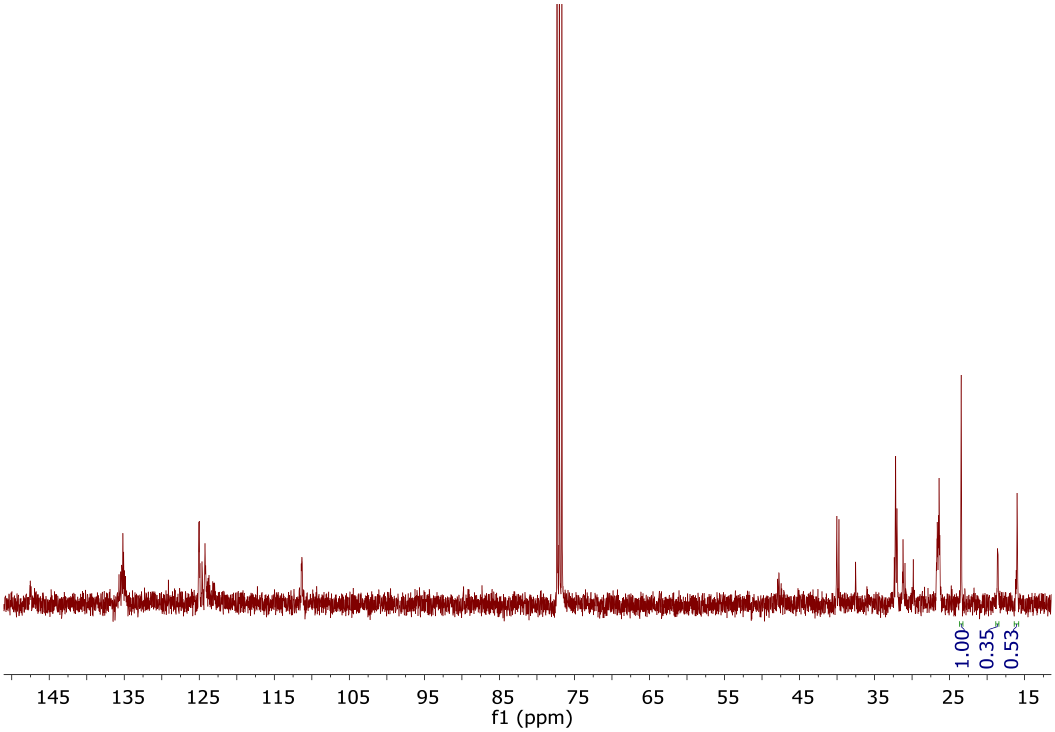

Fig. S182 ^{13}C NMR spectrum of PIP 500 equivalents generated by **$Sm(CH_2SiMe_3)_3(THF)_3$**, 2 equivalents $[Ph_3C][B(C_6F_5)_4]$, and 1 equivalent PPh_3 from **Table 7**, entry 1 in $CDCl_3$ at 298 K (PPh_3 addition time 0 min).

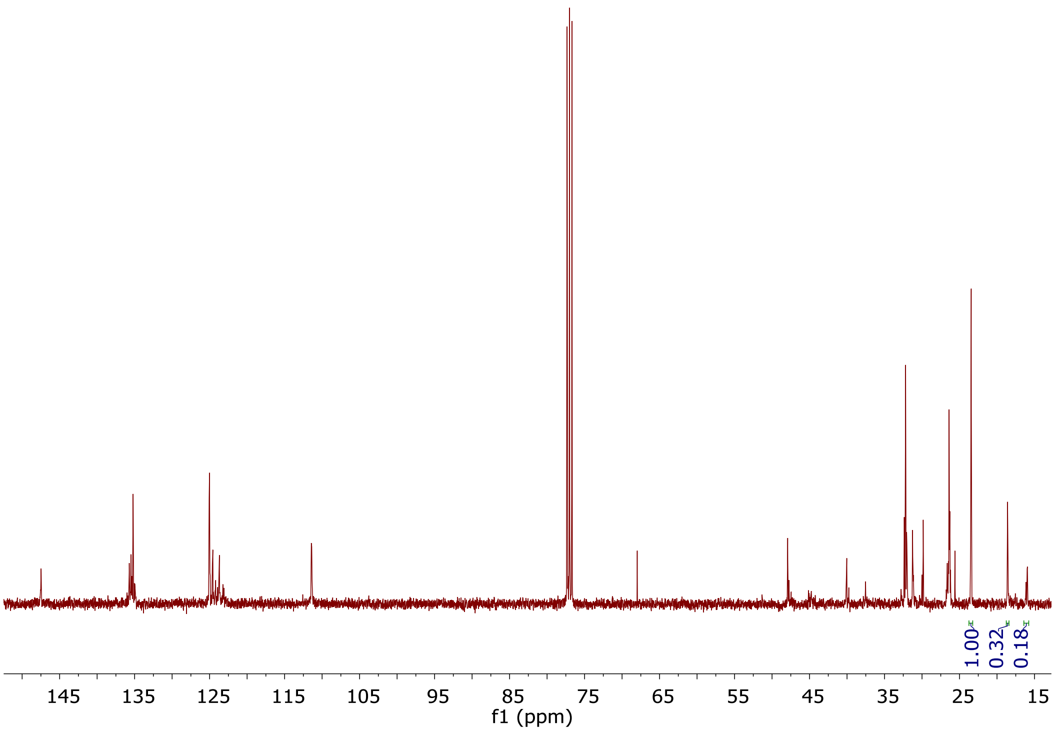

Fig. S183 ^{13}C NMR spectrum of PIP 500 equivalents generated by **$Sm(CH_2SiMe_3)_3(THF)_3$**, 2 equivalents $[Ph_3C][B(C_6F_5)_4]$, and 1 equivalent PPh_3 from **Table 7**, entry 2 in $CDCl_3$ at 298 K (PPh_3 addition time 10 min).

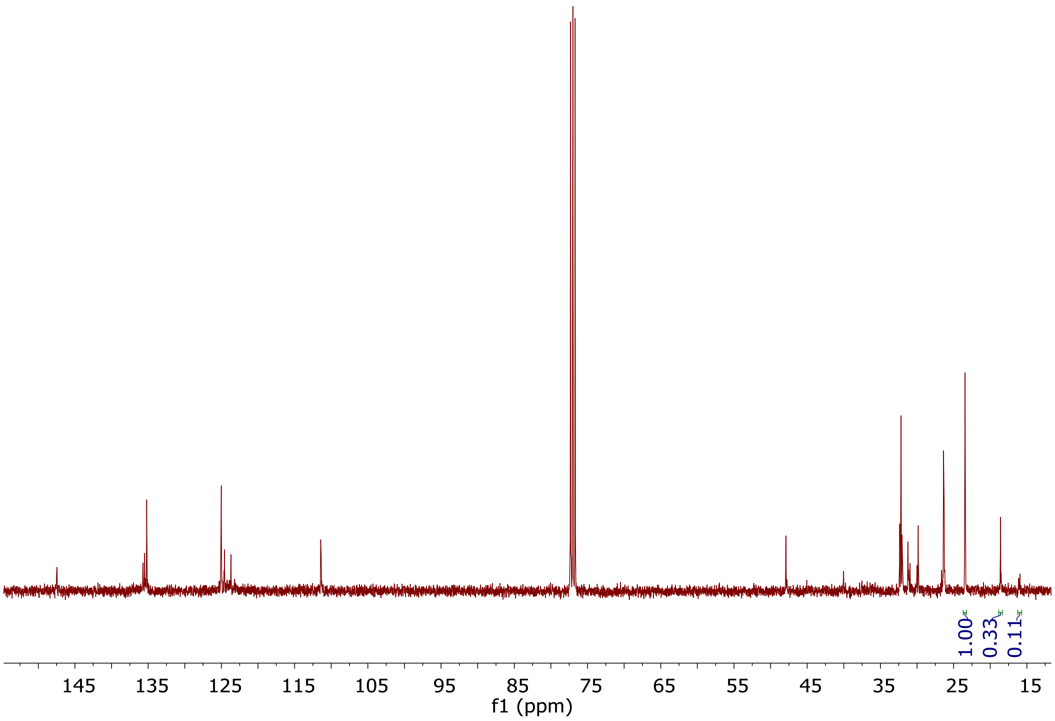

Fig. S184 ^{13}C NMR spectrum of PIP 500 equivalents generated by **$Sm(CH_2SiMe_3)_3(THF)_3$**, 2 equivalents $[Ph_3C][B(C_6F_5)_4]$, and 1 equivalent PPh_3 from **Table 7**, entry 3 in $CDCl_3$ at 298 K (PPh_3 addition time 30 min).

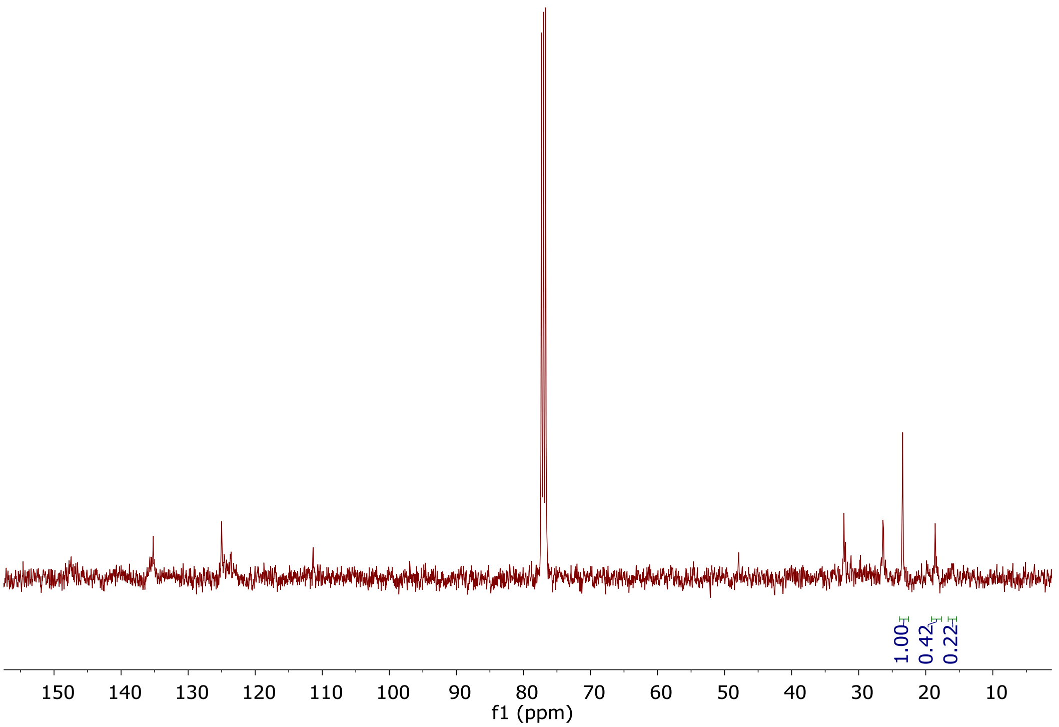

Fig. S185 ^{13}C NMR spectrum of PIP 500 equivalents generated by **$Gd(CH_2SiMe_3)_3(THF)_2$**, 2 equivalents $[Ph_3C][B(C_6F_5)_4]$, and 1 equivalent PPh_3 from **Table 7**, entry 4 in $CDCl_3$ at 298 K (PPh_3 addition time 0 min).

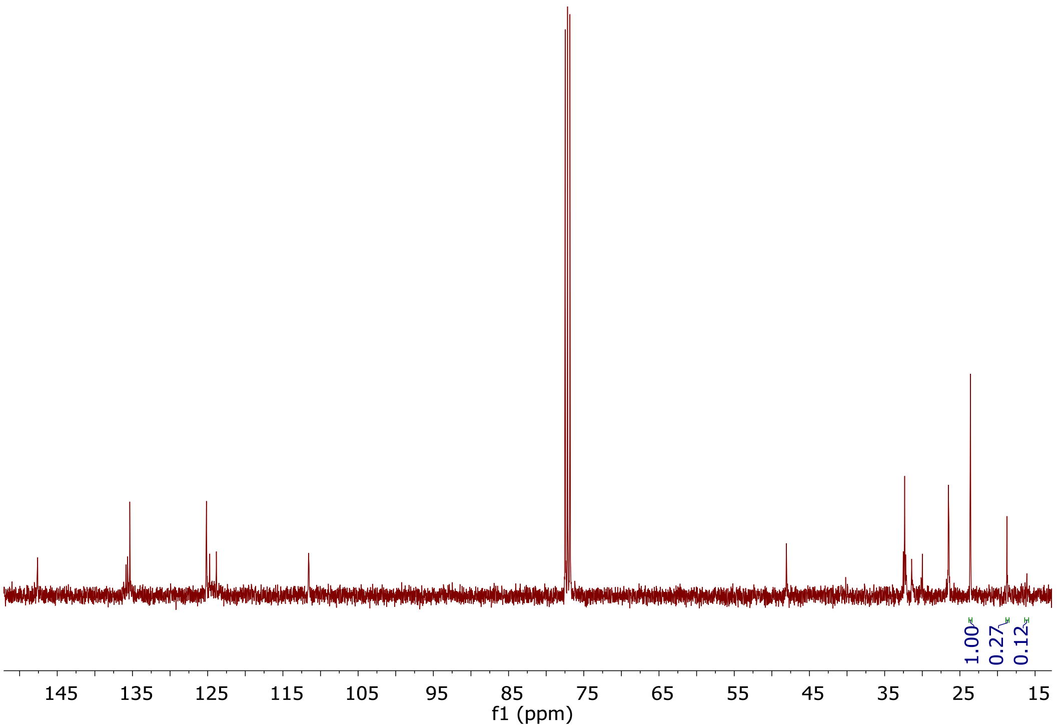

Fig. S186 ^{13}C NMR spectrum of PIP 500 equivalents generated by **$Gd(CH_2SiMe_3)_3(THF)_2$**, 2 equivalents $[Ph_3C][B(C_6F_5)_4]$, and 1 equivalent PPh_3 from **Table 7**, entry 5 in $CDCl_3$ at 298 K (PPh_3 addition time 10 min).

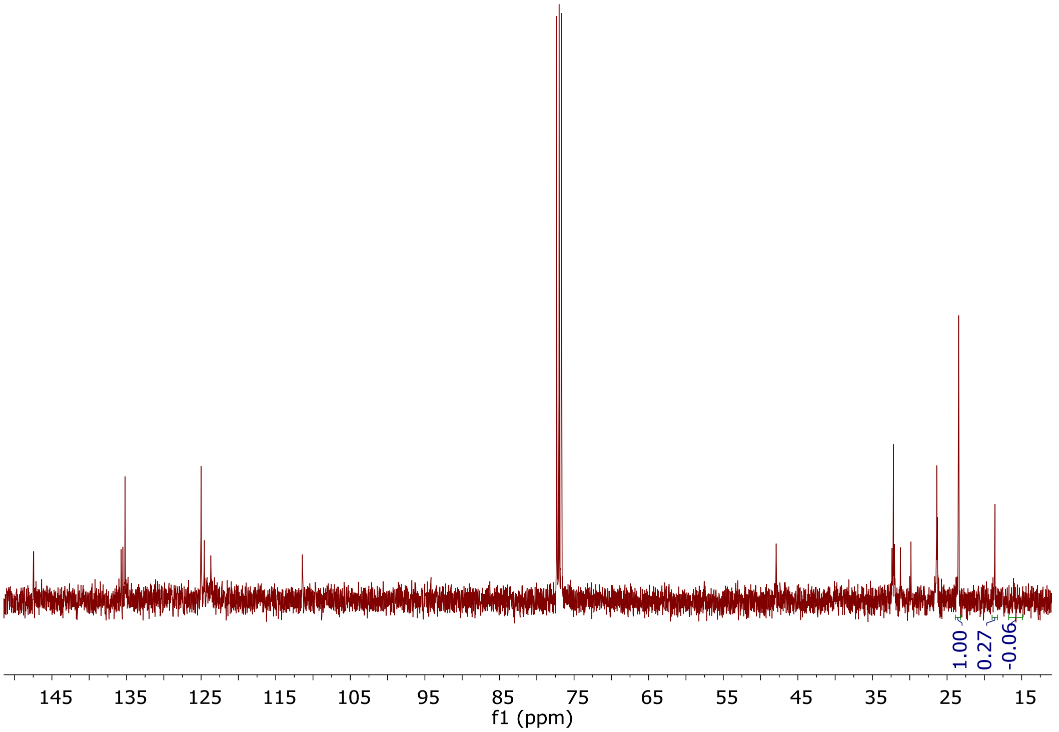

Fig. S187 ^{13}C NMR spectrum of PIP 500 equivalents generated by **Gd(CH$_2$SiMe$_3$)$_3$(THF)$_2$**, 2 equivalents [Ph$_3$C][B(C$_6$F$_5$)$_4$], and 1 equivalent PPh$_3$ from **Table 7**, entry 6 in CDCl$_3$ at 298 K (PPh$_3$ addition time 30 min).

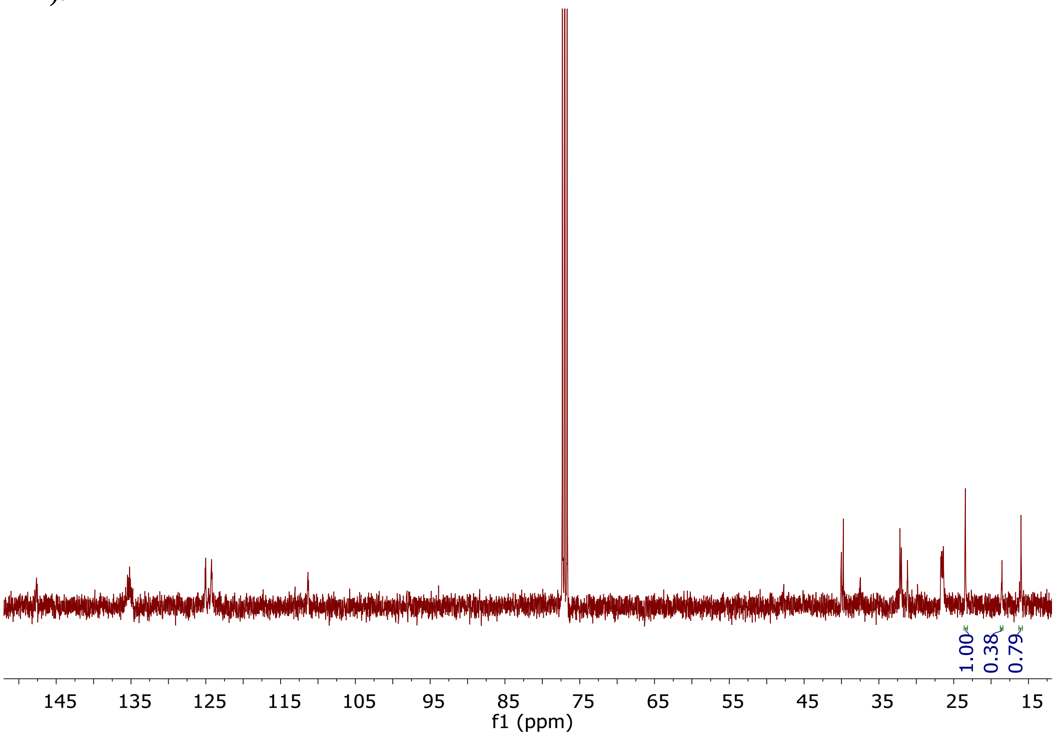

Fig. S188 ^{13}C NMR spectrum of PIP 500 equivalents generated by **Y(CH$_2$SiMe$_3$)$_3$(THF)$_2$**, 2 equivalents [Ph$_3$C][B(C$_6$F$_5$)$_4$], and 1 equivalent PPh$_3$ from **Table 7**, entry 7 in CDCl$_3$ at 298 K (PPh$_3$ addition time 0 min).

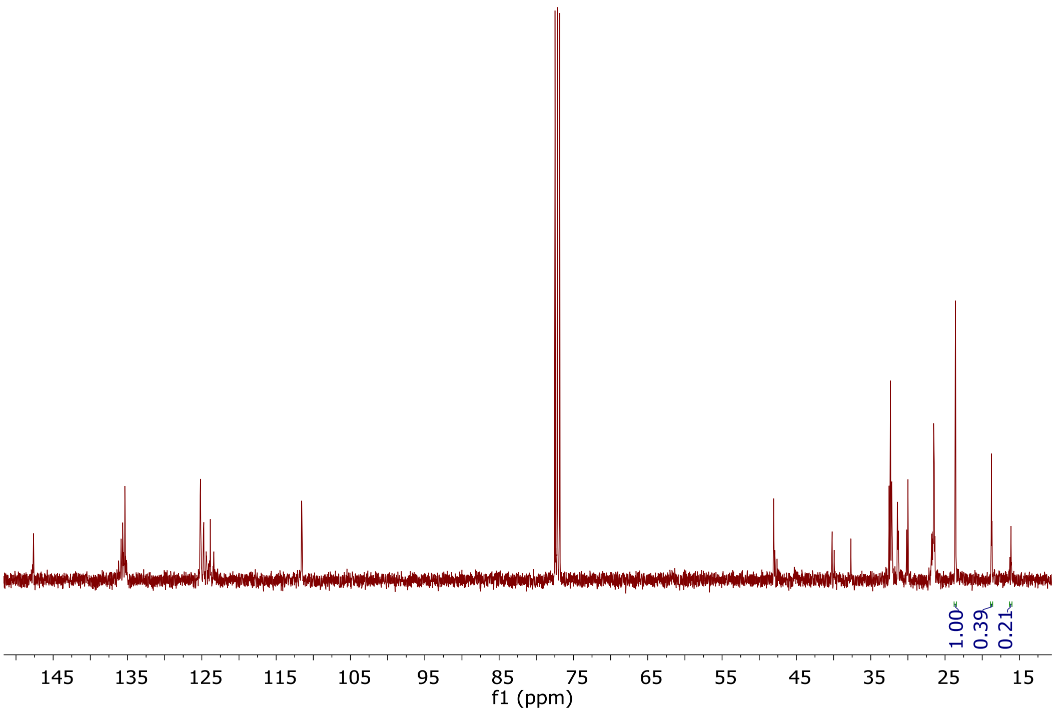

Fig. S189 ^{13}C NMR spectrum of PIP 500 equivalents generated by **$Y(CH_2SiMe_3)_3(THF)_2$**, 2 equivalents $[Ph_3C][B(C_6F_5)_4]$, and 1 equivalent PPh_3 from **Table 7**, entry 8 in $CDCl_3$ at 298 K (PPh_3 addition time 10 min).

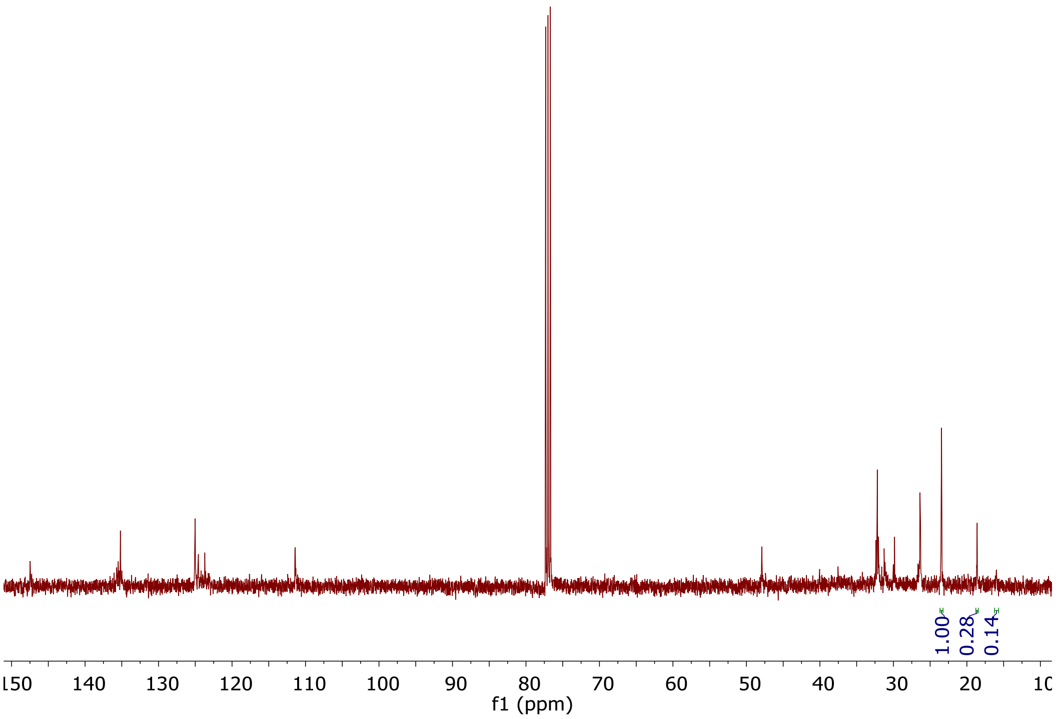

Fig. S190 ^{13}C NMR spectrum of PIP 500 equivalents generated by **$Y(CH_2SiMe_3)_3(THF)_2$**, 2 equivalents $[Ph_3C][B(C_6F_5)_4]$, and 1 equivalent PPh_3 from **Table 7**, entry 9 in $CDCl_3$ at 298 K (PPh_3 addition time 30 min).

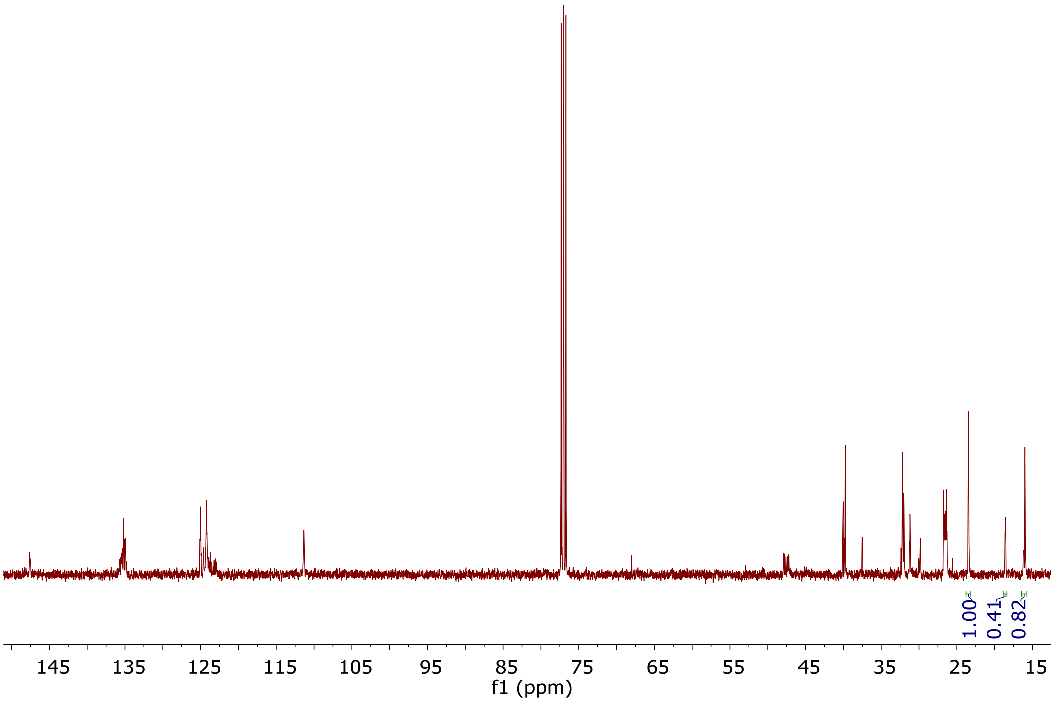

Fig. S191 ^{13}C NMR spectrum of PIP 500 equivalents generated by **Tm(CH$_2$SiMe$_3$)$_3$(THF)$_2$**, 2 equivalents [Ph$_3$C][B(C$_6$F$_5$)$_4$], and 1 equivalent PPh$_3$ from **Table 7**, entry 10 in CDCl$_3$ at 298 K (PPh$_3$ addition time 0 min).

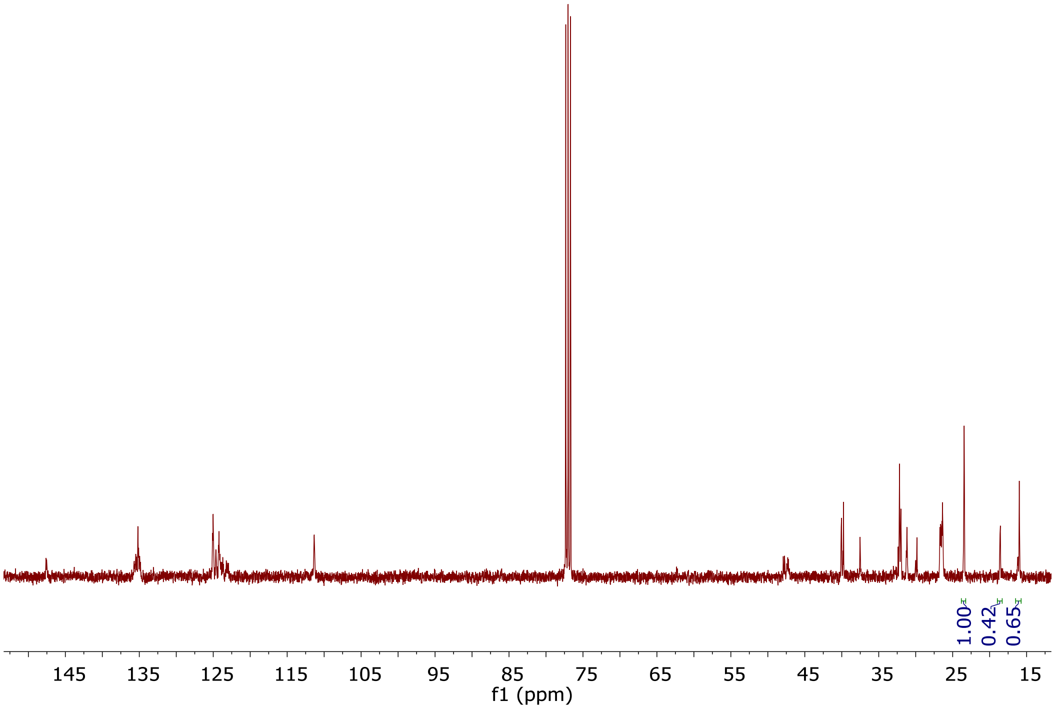

Fig. S192 ^{13}C NMR spectrum of PIP 500 equivalents generated by **Tm(CH$_2$SiMe$_3$)$_3$(THF)$_2$**, 2 equivalents [Ph$_3$C][B(C$_6$F$_5$)$_4$], and 1 equivalent PPh$_3$ from **Table 7**, entry 11 in CDCl$_3$ at 298 K (PPh$_3$ addition time 10 min).

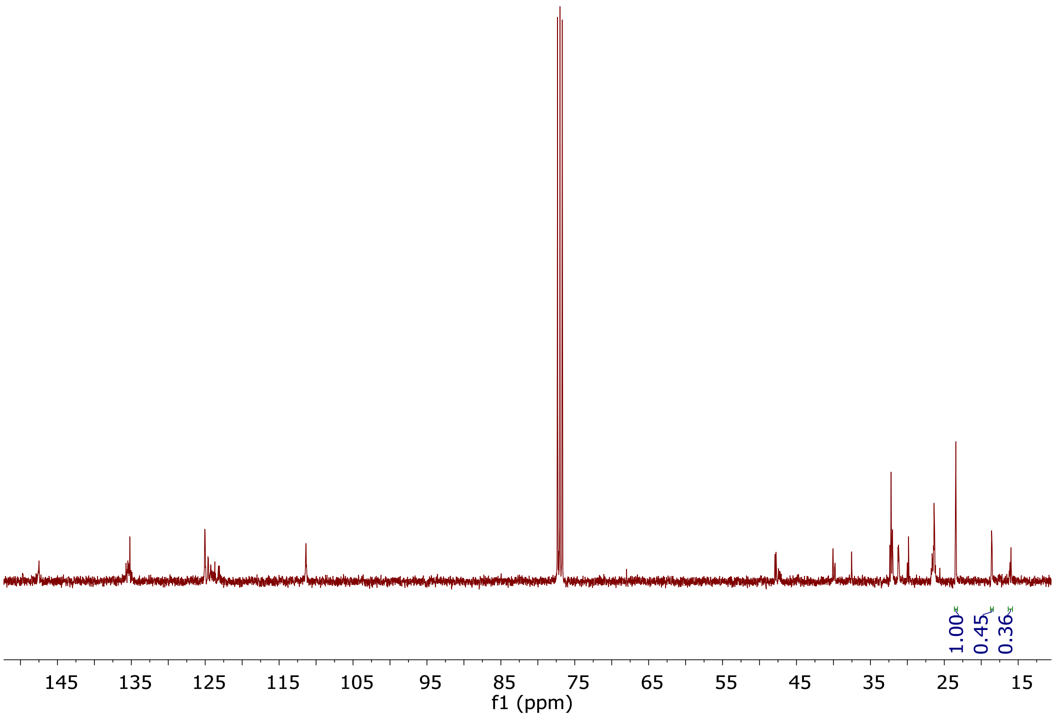

Fig. S193 ^{13}C NMR spectrum of PIP 500 equivalents generated by **$Tm(CH_2SiMe_3)_3(THF)_2$**, 2 equivalents $[Ph_3C][B(C_6F_5)_4]$, and 1 equivalent PPh_3 from **Table 7**, entry 12 in $CDCl_3$ at 298 K (PPh_3 addition time 30 min).

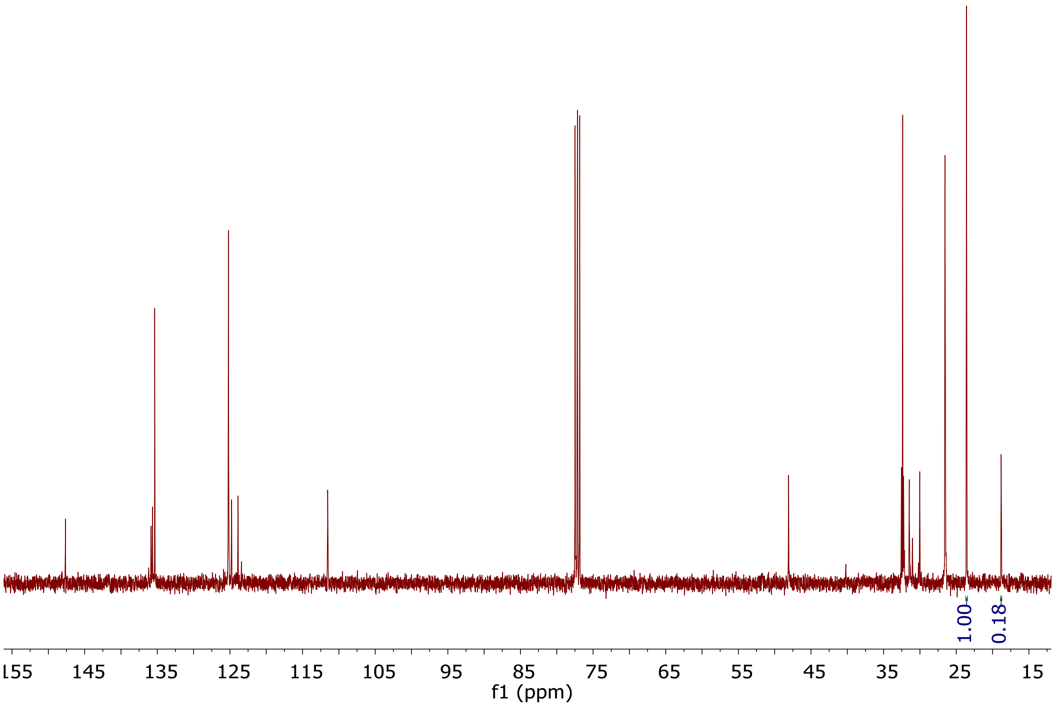

Fig. S194 ^{13}C NMR spectrum of PIP 500 equivalents generated by **$Y(CH_2SiMe_3)_3(THF)_2$** and 2 equivalents $[Ph_3C][B(C_6F_5)_4]$ from **Table 7**, entry 13 in $CDCl_3$ at 298 K (IP addition time 10 min).

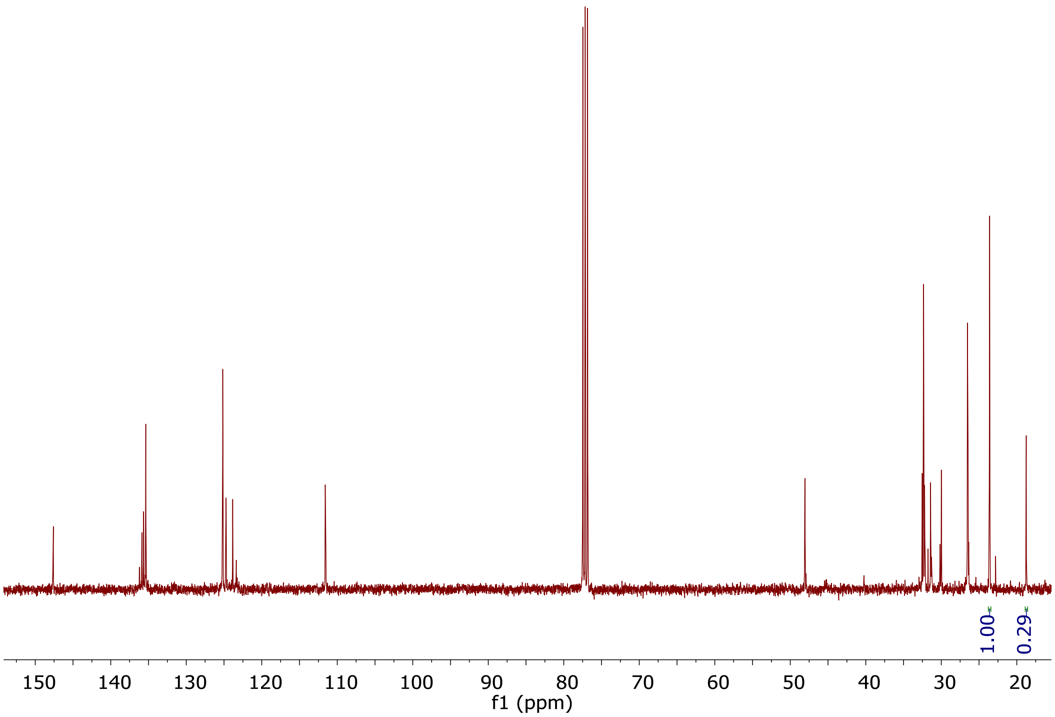

Fig. S195 ^{13}C NMR spectrum of PIP 500 equivalents generated by **$Y(CH_2SiMe_3)_3(THF)_2$** and 2 equivalents $[Ph_3C][B(C_6F_5)_4]$ from **Table 7**, entry 14 in $CDCl_3$ at 298 K (IP addition time 20 min).

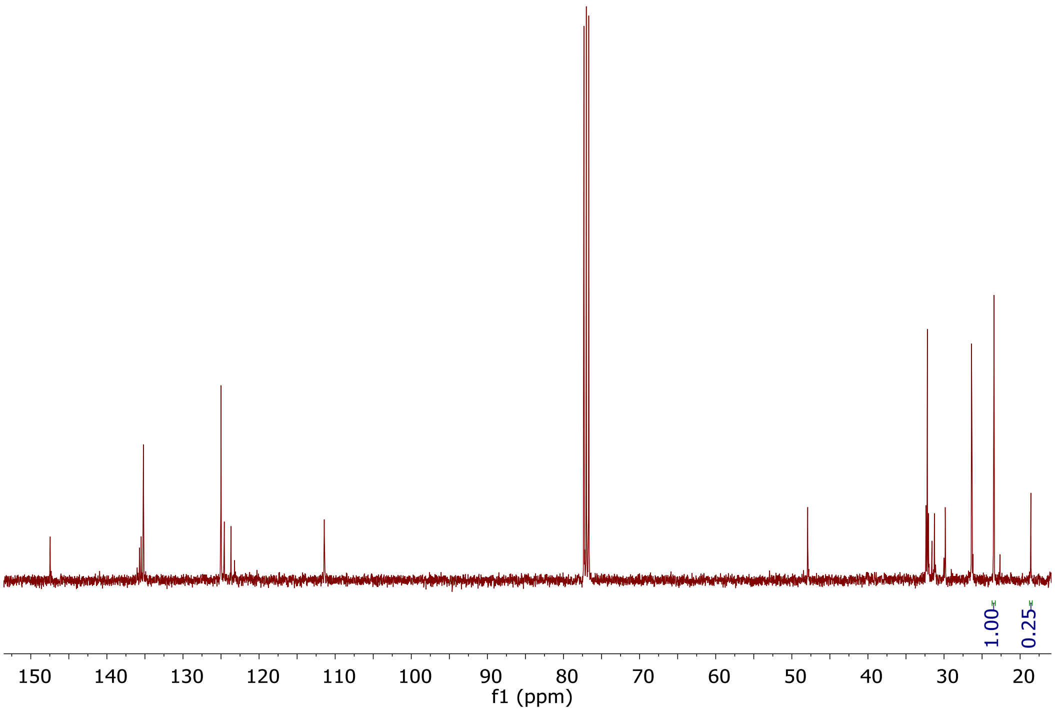

Fig. S196 ^{13}C NMR spectrum of PIP 500 equivalents generated by **$Y(CH_2SiMe_3)_3(THF)_2$** and 2 equivalents $[Ph_3C][B(C_6F_5)_4]$ from **Table 7**, entry 15 in $CDCl_3$ at 298 K (IP addition time 40 min).

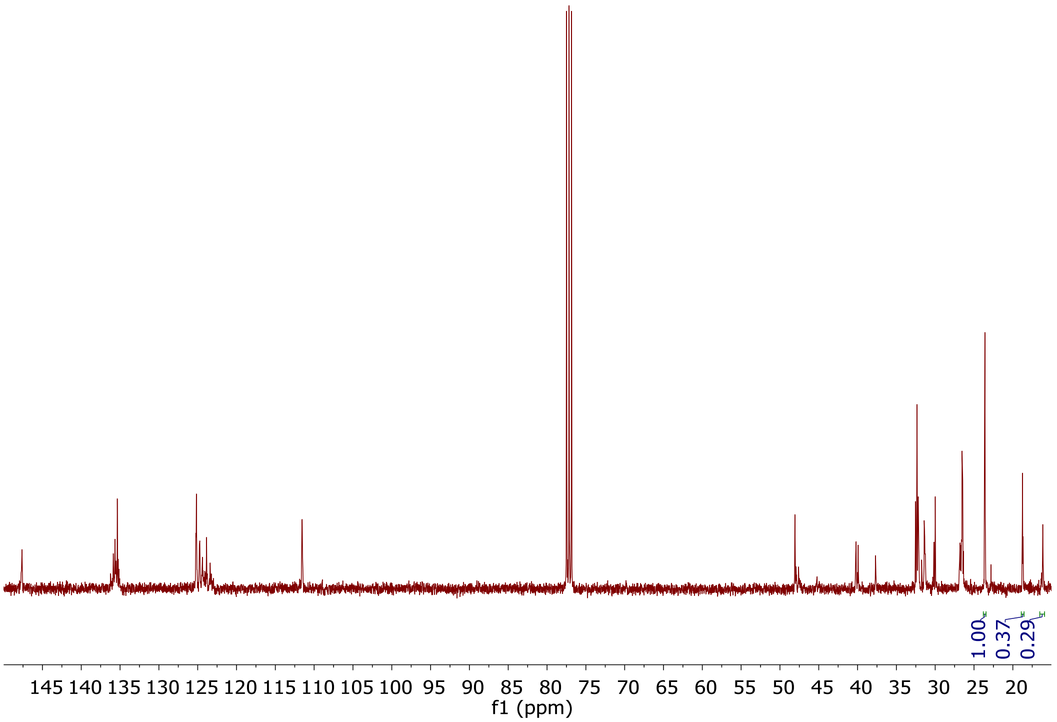

Fig. S197 ^{13}C NMR spectrum of PIP 500 equivalents generated by **$Y(CH_2SiMe_3)_3(THF)_2$**, and 2 equivalents $[Ph_3C][B(C_6F_5)_4]$, and 1 equivalent PPh_3 from **Table 8**, entry 1 (Step 1: 60 min).

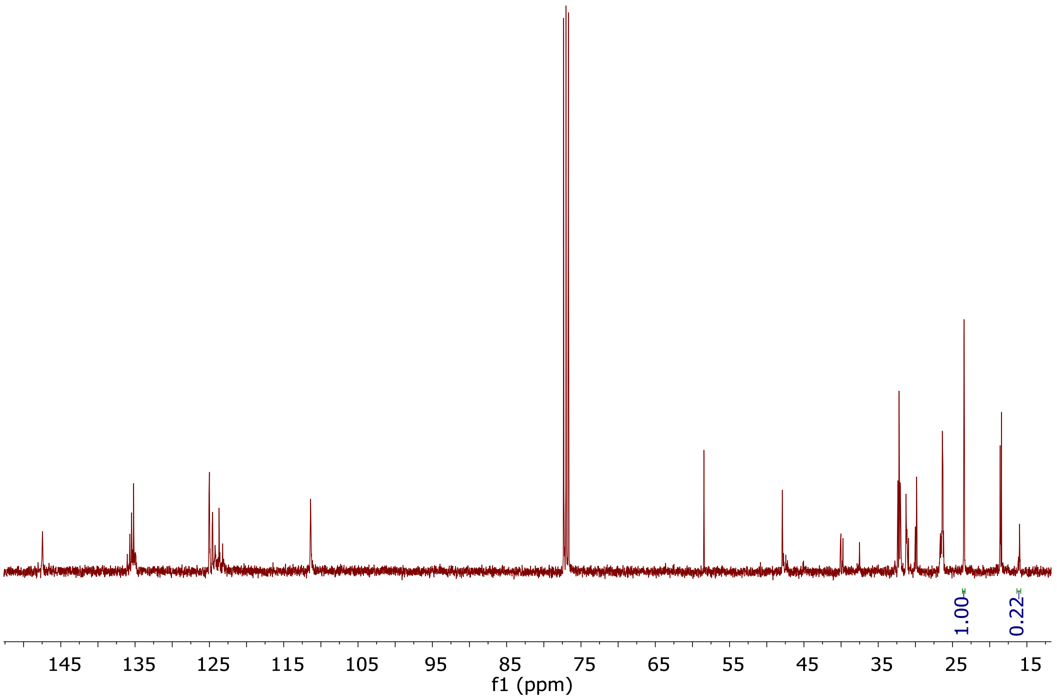

Fig. S198 ^{13}C NMR spectrum of PIP 500 equivalents generated by **$Y(CH_2SiMe_3)_3(THF)_2$**, and 2 equivalents $[Ph_3C][B(C_6F_5)_4]$, and 1 equivalent PPh_3 from **Table 8**, entry 2 (Step 2: 60 min).

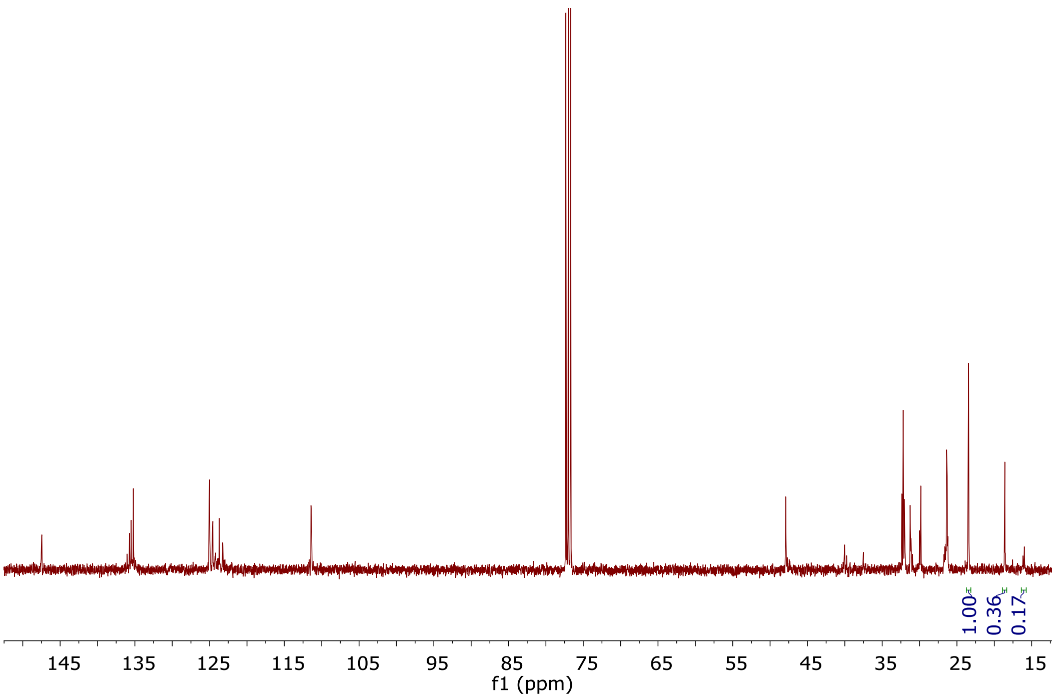

Fig. S199 ^{13}C NMR spectrum of PIP 500 equivalents generated by **$Y(CH_2SiMe_3)_3(THF)_2$**, and 2 equivalents $[Ph_3C][B(C_6F_5)_4]$, and 1 equivalent PPh_3 from **Table 8**, entry 3 (Step 3: 60 min).

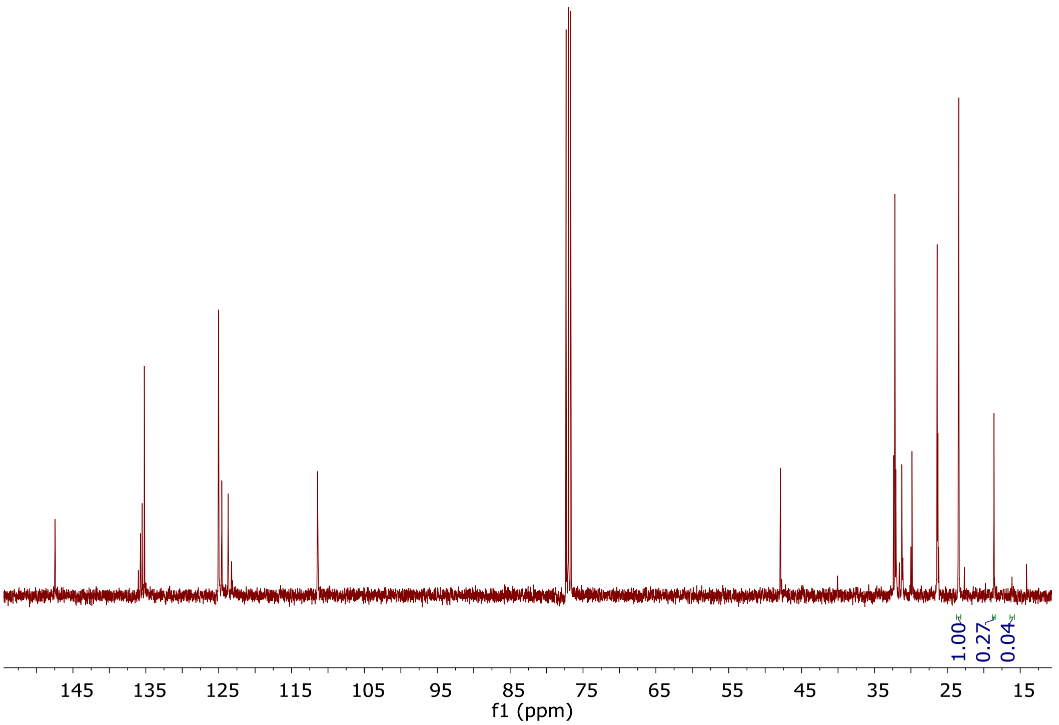

Fig. S200 ^{13}C NMR spectrum of PIP 500 equivalents generated by **$Y(CH_2SiMe_3)_3(THF)_2$** and 2 equivalents $[Ph_3C][B(C_6F_5)_4]$ from **Table 8**, entry 4 (Step 1: 60 min).

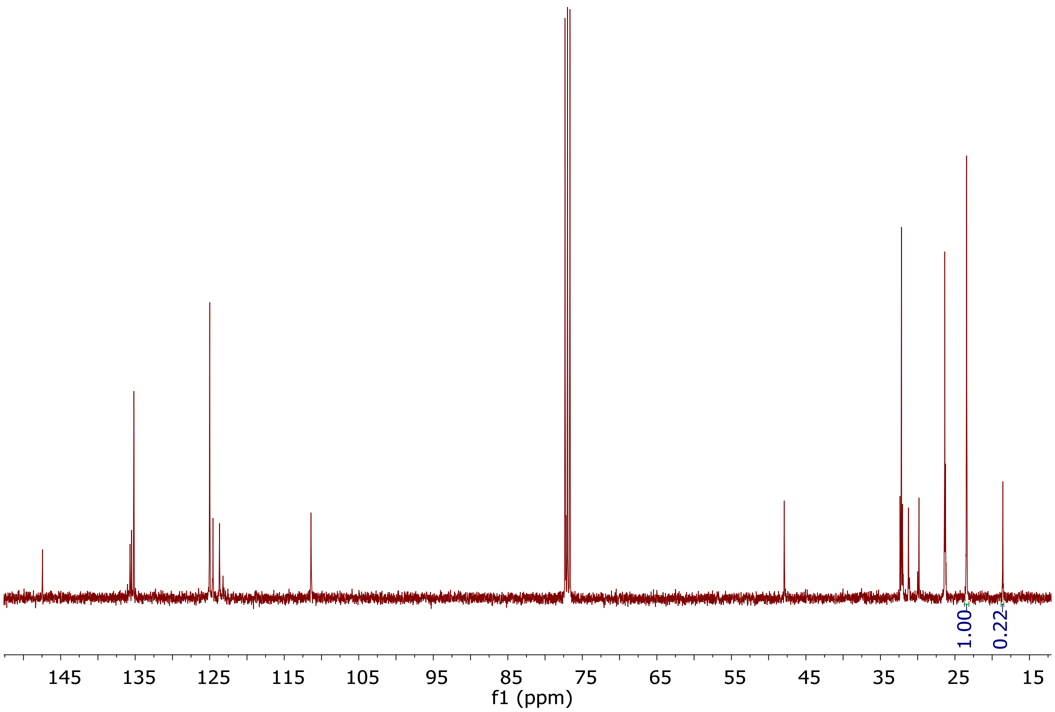

Fig. S201 ^{13}C NMR spectrum of PIP 500 equivalents generated by **$Y(CH_2SiMe_3)_3(THF)_2$** and 2 equivalents $[Ph_3C][B(C_6F_5)_4]$ from **Table 8**, entry 5 (Step 2: 60 min).

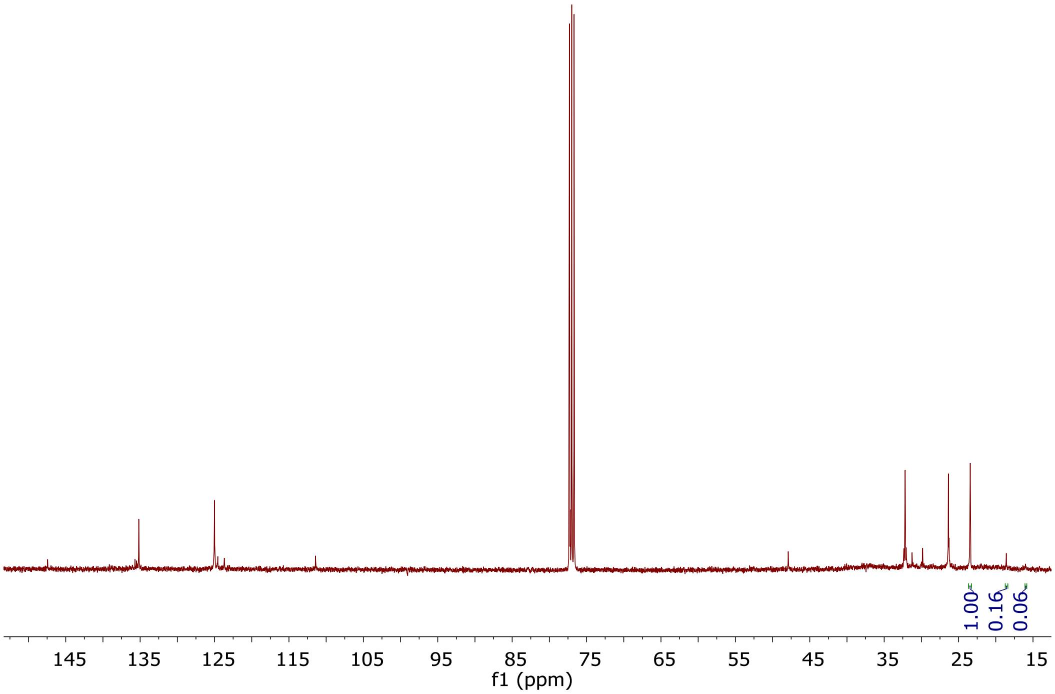

Fig. S202 ^{13}C NMR spectrum of PIP 500 equivalents generated by **$Y(CH_2SiMe_3)_3(THF)_2$** and 2 equivalents $[Ph_3C][B(C_6F_5)_4]$ from **Table 8**, entry 6 (Step 3: 60 min).
