# Supplementary material for: Enhanced Control of Isoprene Polymerization with Trialkyl Rare Earth Metal Complexes through Neutral Donor Support
Source: Inorg Chem. 2023 Dec 8;63(21):9464–77. doi: 10.1021/acs.inorgchem.3c03161 (PMC11134520; doi:10.1021/acs.inorgchem.3c03161)
Supplement: Supplementary file 3 — ic3c03161_si_003.pdf [file ic3c03161_si_003.pdf]

4.0 Fourier-Transform Infrared (FT-IR) Spectroscopy Characterization of Isolated Polymers.

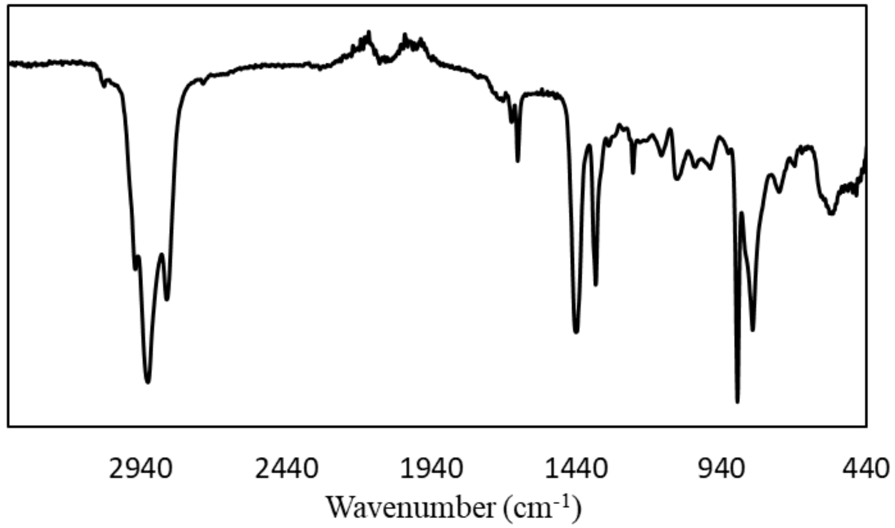

Fig. S203 FT-IR spectrum of PIP 500 equivalents generated by **$Y(CH_2SiMe_3)_3(THF)_2$** and 1 equivalent $[Ph_3C][B(C_6F_5)_4]$ from **Table 1**, entry 1 (30 min).

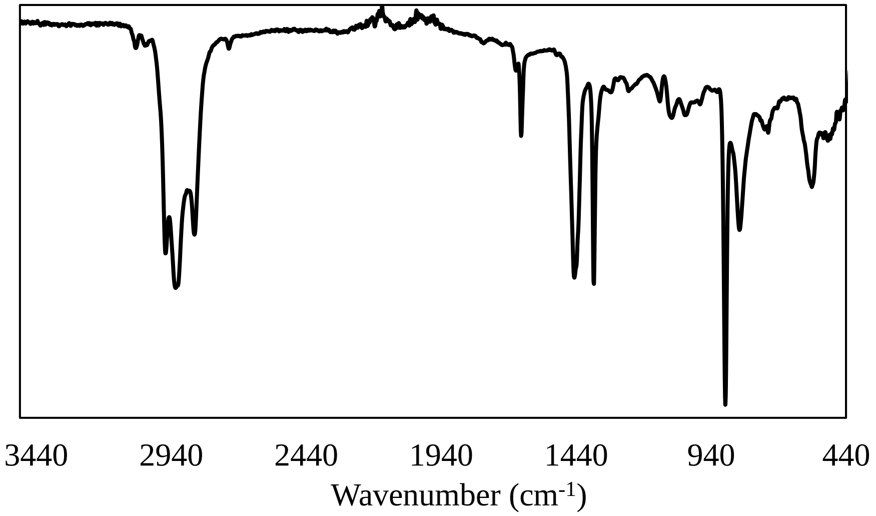

Fig. S204 FT-IR spectrum of PIP 500 equivalents generated by **$Y(CH_2SiMe_3)_3(THF)_2$** and 2 equivalents $[Ph_3C][B(C_6F_5)_4]$ from **Table 1**, entry 2 (30 min).

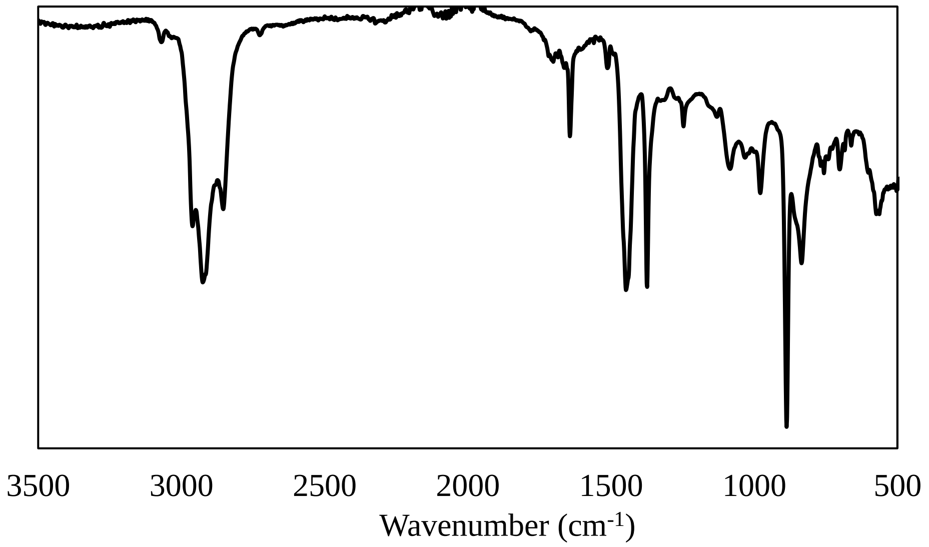

Fig. S205 FT-IR spectrum of PIP 500 equivalents generated by $\mathbf{Y(CH_2SiMe_3)_3(THF)_2}$, 2 equivalents $[Ph_3C][B(C_6F_5)_4]$, and 1 equivalent Bipy from **Table 2**, entry 3 (30 min).

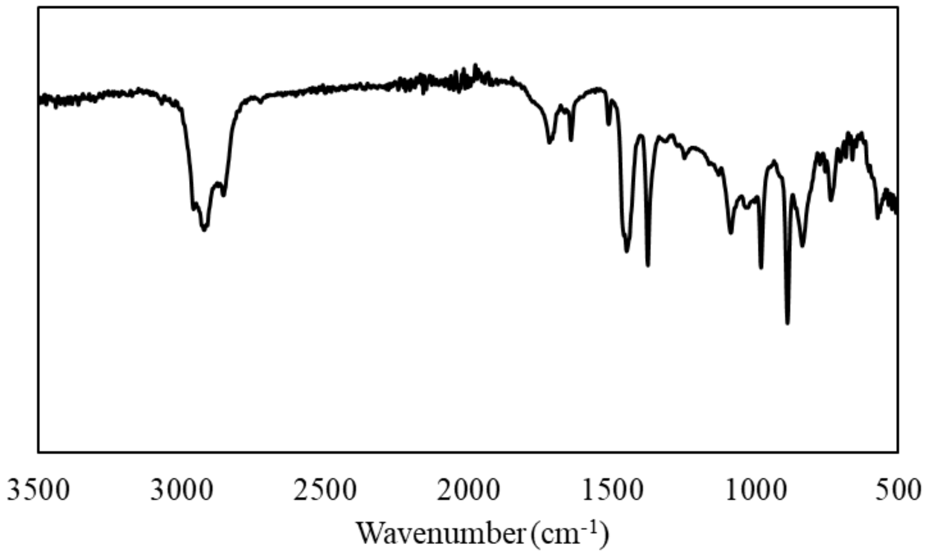

Fig. S206 FT-IR spectrum of PIP 500 equivalents generated by $\mathbf{Y(CH_2SiMe_3)_3(THF)_2}$, 2 equivalents $[Ph_3C][B(C_6F_5)_4]$, and 1 equivalent MeCN from **Table 2**, entry 5 (30 min).

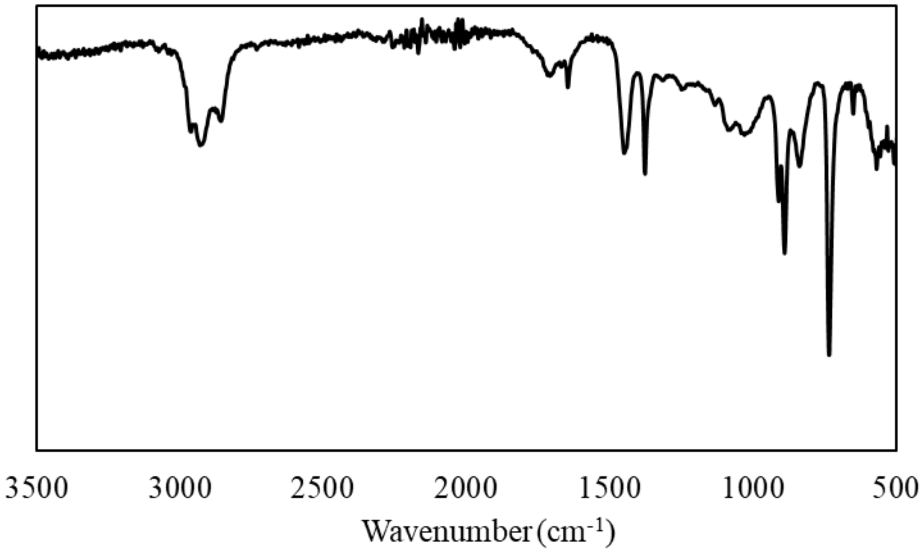

Fig. S207 FT-IR spectrum of PIP 500 equivalents generated by **$Y(CH_2SiMe_3)_3(THF)_2$**, 2 equivalents $[Ph_3C][B(C_6F_5)_4]$, and 1 equivalent P(*o*-tolyl)$_3$ from **Table 2**, entry 6 (30 min).

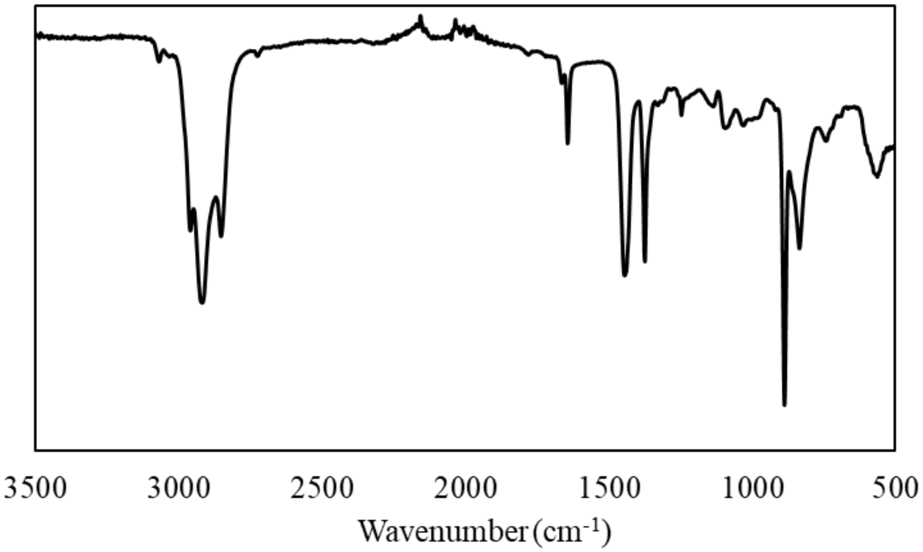

Fig. S208 FT-IR spectrum of PIP 500 equivalents generated by **$Y(CH_2SiMe_3)_3(THF)_2$**, 2 equivalents $[Ph_3C][B(C_6F_5)_4]$, and 1 equivalent PCy_3 from **Table 2**, entry 7 (30 min).

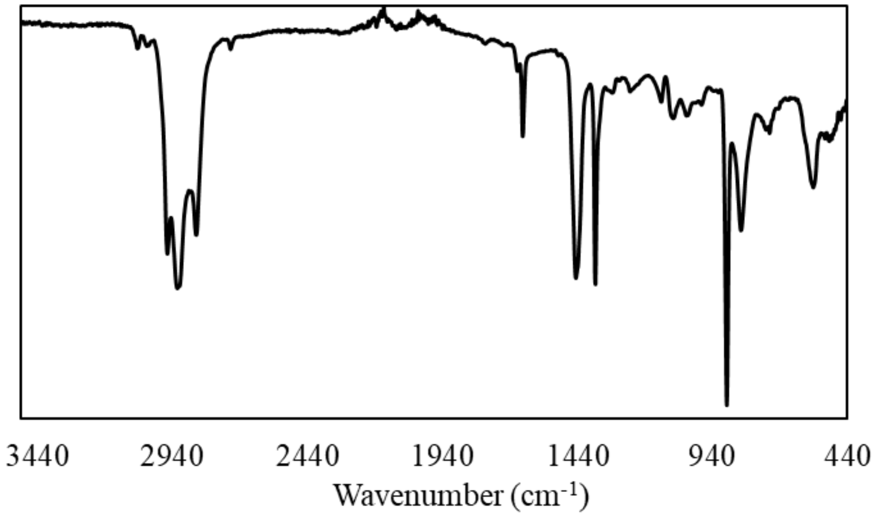

Fig. S209 FT-IR spectrum of PIP 500 equivalents generated by **$Y(CH_2SiMe_3)_3(THF)_2$**, 2 equivalents $[Ph_3C][B(C_6F_5)_4]$, and 1 equivalent PPh_3 from **Table 2**, entry 8 (30 min).

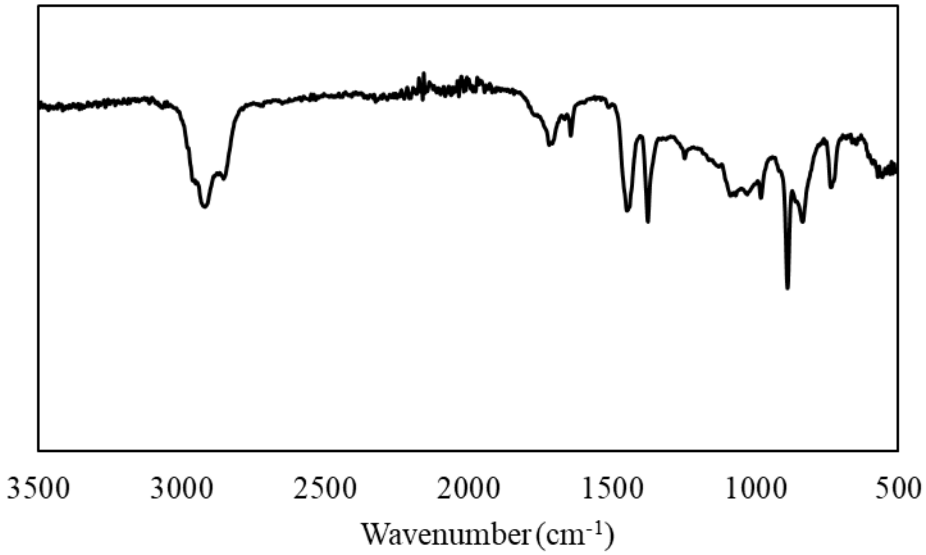

Fig. S210 FT-IR spectrum of PIP 500 equivalents generated by **$Y(CH_2SiMe_3)_3(THF)_2$**, 2 equivalents $[Ph_3C][B(C_6F_5)_4]$, and 1 equivalent P(Ph-*p*-OMe)$_3$ from **Table S1**, entry 1 (30 min).

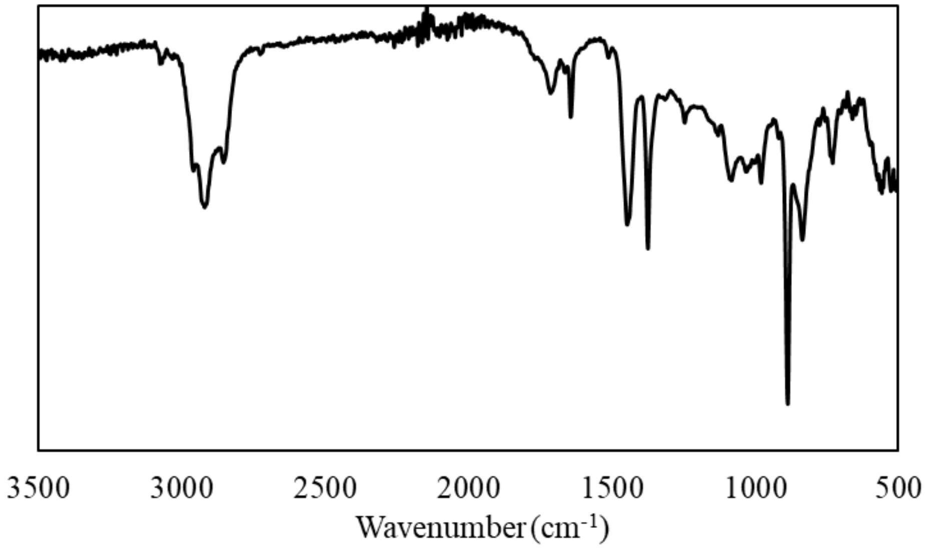

Fig. S211 FT-IR spectrum of PIP 500 equivalents generated by **$Y(CH_2SiMe_3)_3(THF)_2$**, 2 equivalents $[Ph_3C][B(C_6F_5)_4]$, and 1 equivalent P(*p*-tolyl)$_3$ from **Table S1**, entry 2 (30 min).

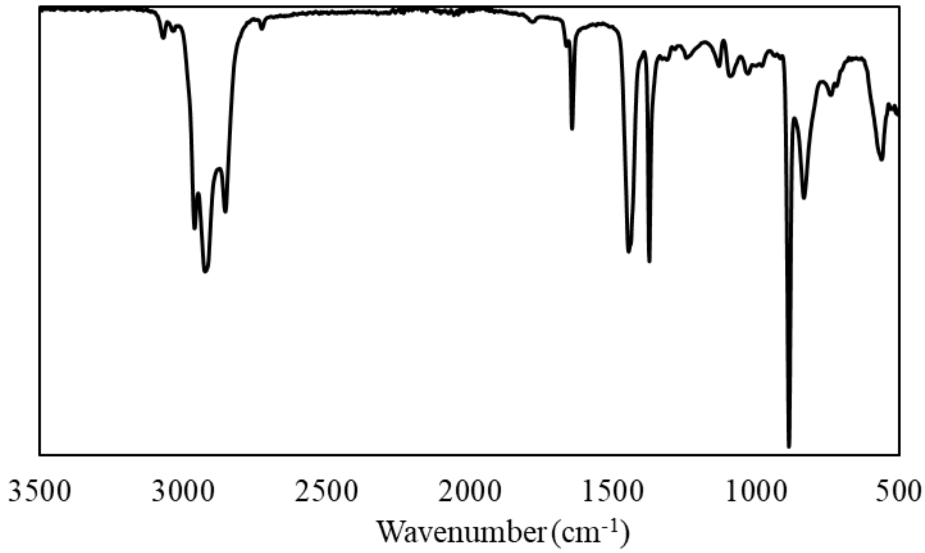

Fig. S212 FT-IR spectrum of PIP 500 equivalents generated by **$Y(CH_2SiMe_3)_3(THF)_2$**, 2 equivalents $[Ph_3C][B(C_6F_5)_4]$, and 1 equivalent P(Ph-*p*-F)$_3$ from **Table S1**, entry 4 (30 min).

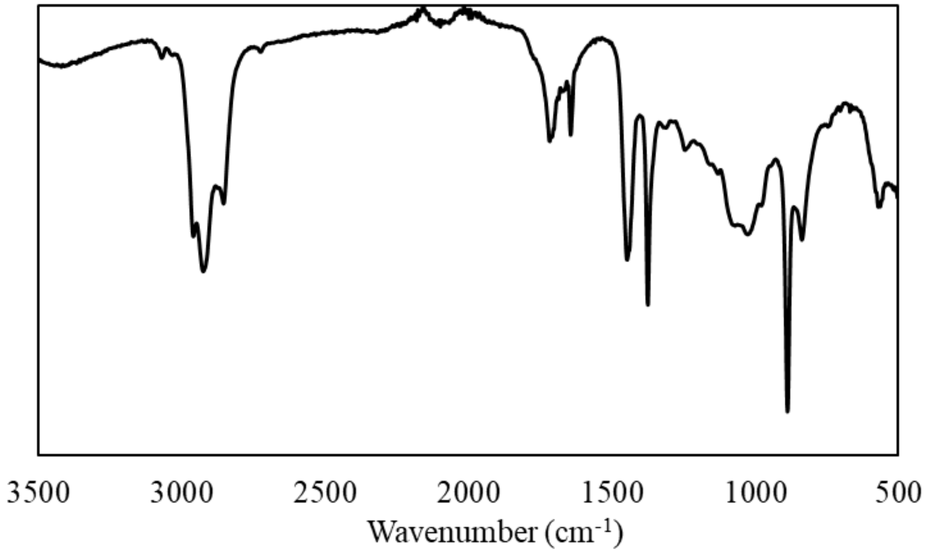

Fig. S213 FT-IR spectrum of PIP 500 equivalents generated by **$Y(CH_2SiMe_3)_3(THF)_2$** and 2 equivalents $[Ph_3C][B(C_6F_5)_4]$ from **Table S2**, entry 1 (5 min).

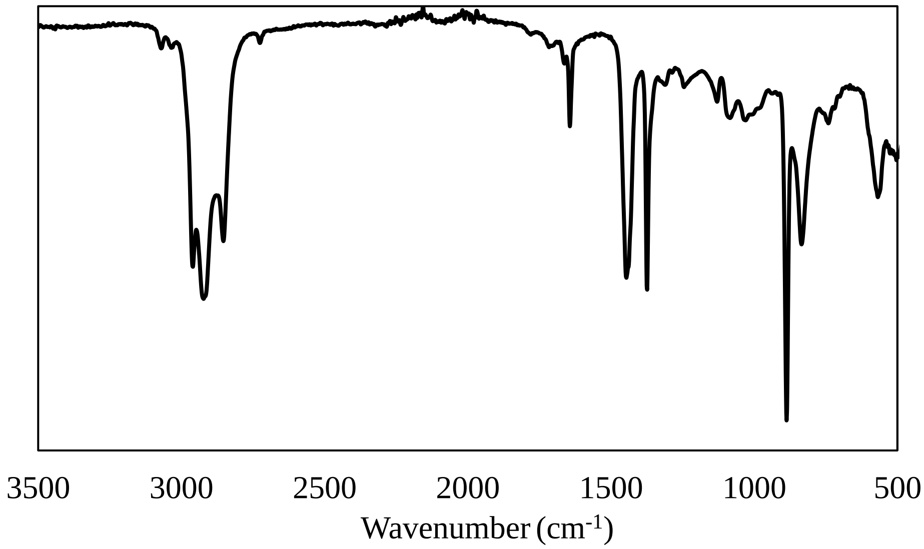

Fig. S214 FT-IR spectrum of PIP 500 equivalents generated by **$Y(CH_2SiMe_3)_3(THF)_2$** and 2 equivalents $[Ph_3C][B(C_6F_5)_4]$ from **Table S2**, entry 2 (12 min).

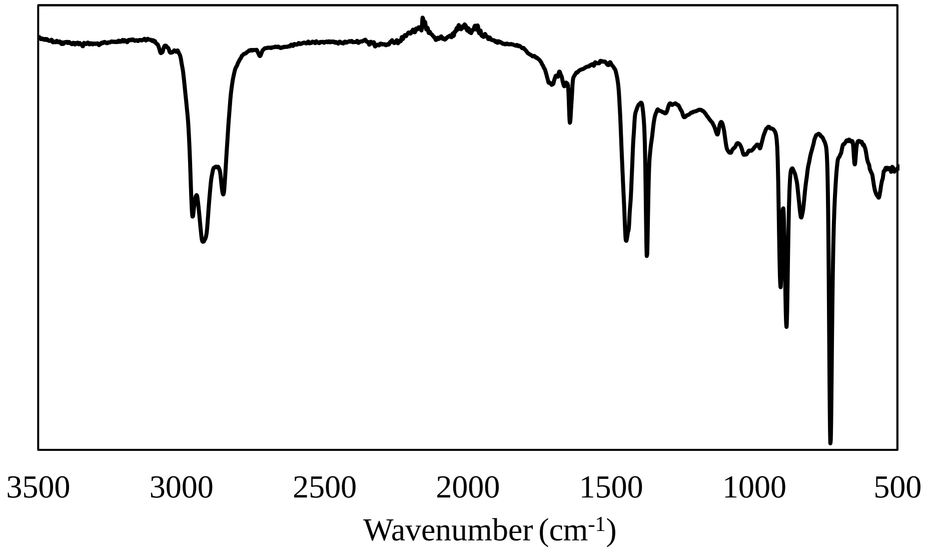

Fig. S215 FT-IR spectrum of PIP 500 equivalents generated by **$Y(CH_2SiMe_3)_3(THF)_2$** and 2 equivalents $[Ph_3C][B(C_6F_5)_4]$ from **Table S2**, entry 3 (18 min).

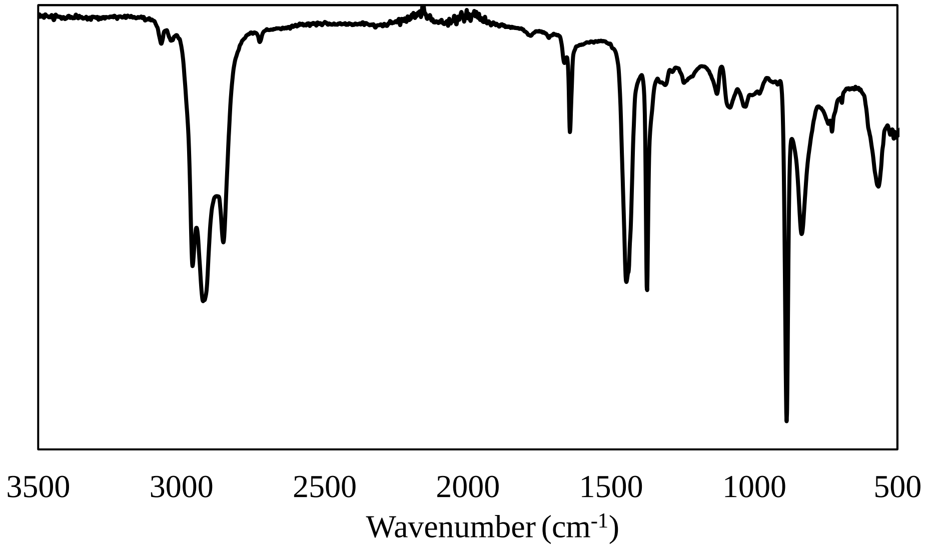

Fig. S216 FT-IR spectrum of PIP 500 equivalents generated by **$Y(CH_2SiMe_3)_3(THF)_2$** and 2 equivalents $[Ph_3C][B(C_6F_5)_4]$ from **Table S2**, entry 4 (24 min).

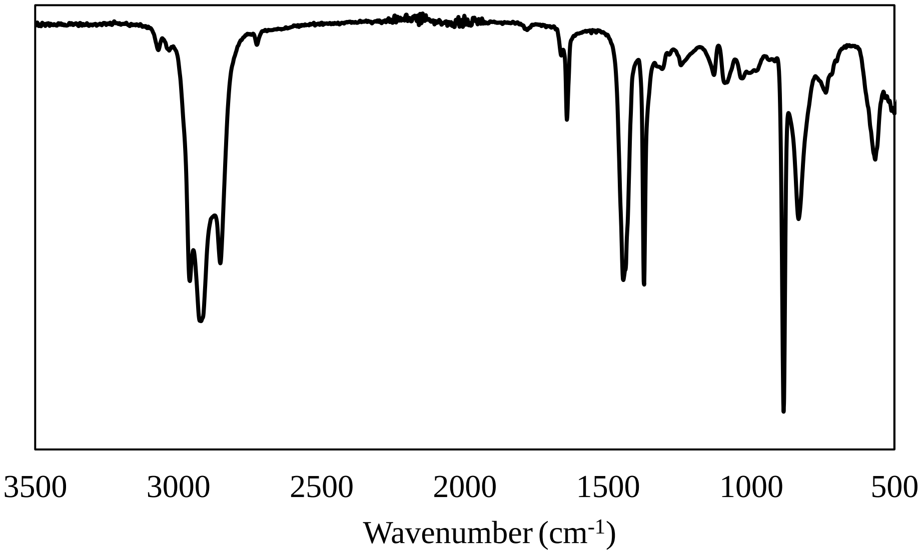

Fig. S217 FT-IR spectrum of PIP 500 equivalents generated by **$Y(CH_2SiMe_3)_3(THF)_2$** and 2 equivalents $[Ph_3C][B(C_6F_5)_4]$ from **Table S2**, entry 5 (30 min).

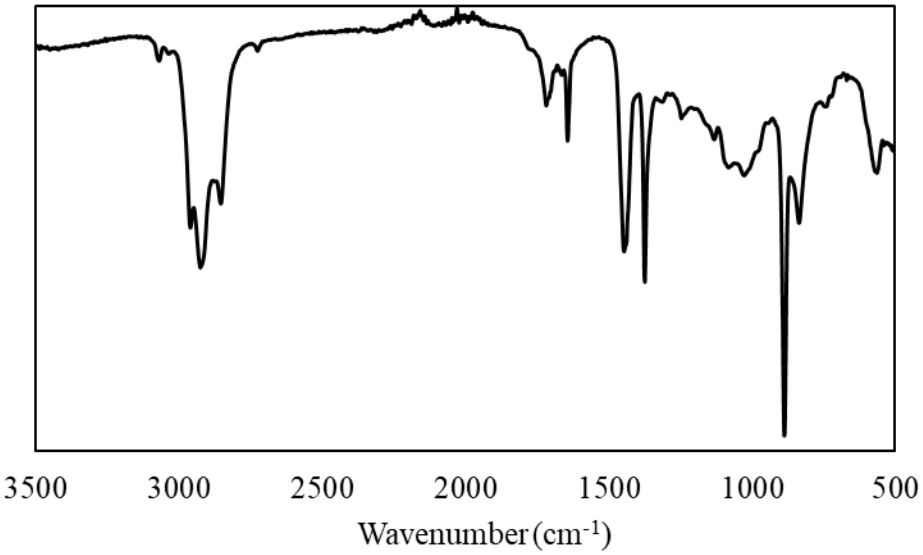

Fig. S218 FT-IR spectrum of PIP 500 equivalents generated by **$Y(CH_2SiMe_3)_3(THF)_2$**, 2 equivalents $[Ph_3C][B(C_6F_5)_4]$, and 1 equivalent PPh_3 from **Table S3**, entry 1 (10 min).

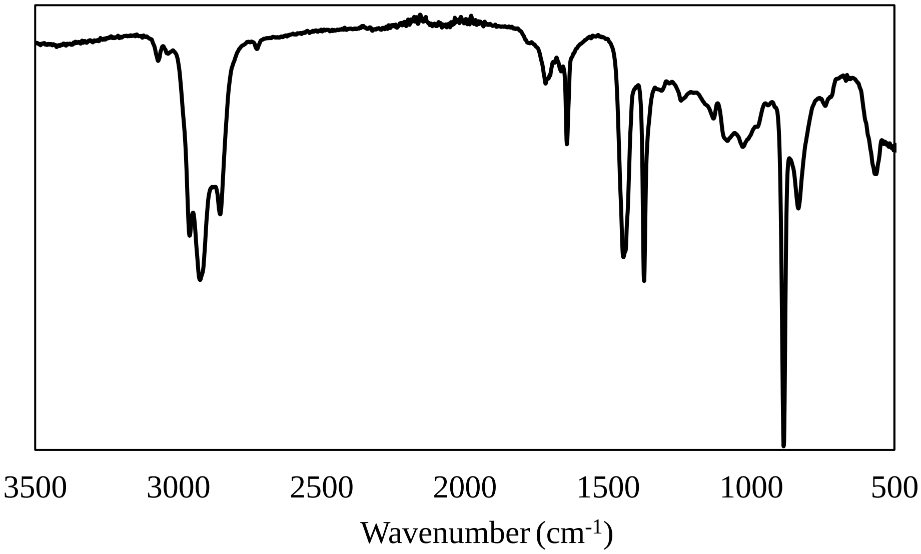

Fig. S219 FT-IR spectrum of PIP 500 equivalents generated by **$Y(CH_2SiMe_3)_3(THF)_2$**, 2 equivalents $[Ph_3C][B(C_6F_5)_4]$, and 1 equivalent PPh_3 from **Table S3**, entry 2 (21 min).

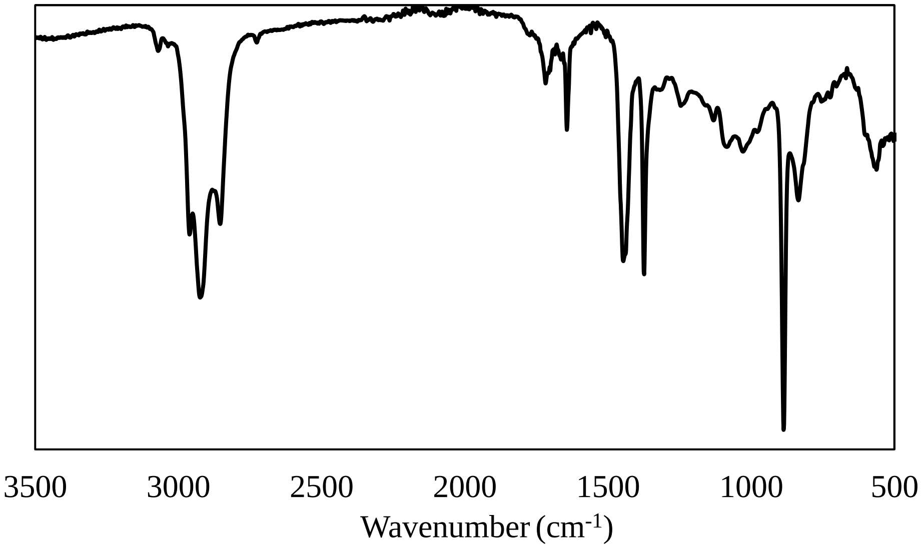

Fig. S220 FT-IR spectrum of PIP 500 equivalents generated by **$Y(CH_2SiMe_3)_3(THF)_2$**, 2 equivalents $[Ph_3C][B(C_6F_5)_4]$, and 1 equivalent PPh_3 from **Table S3**, entry 3 (31 min).

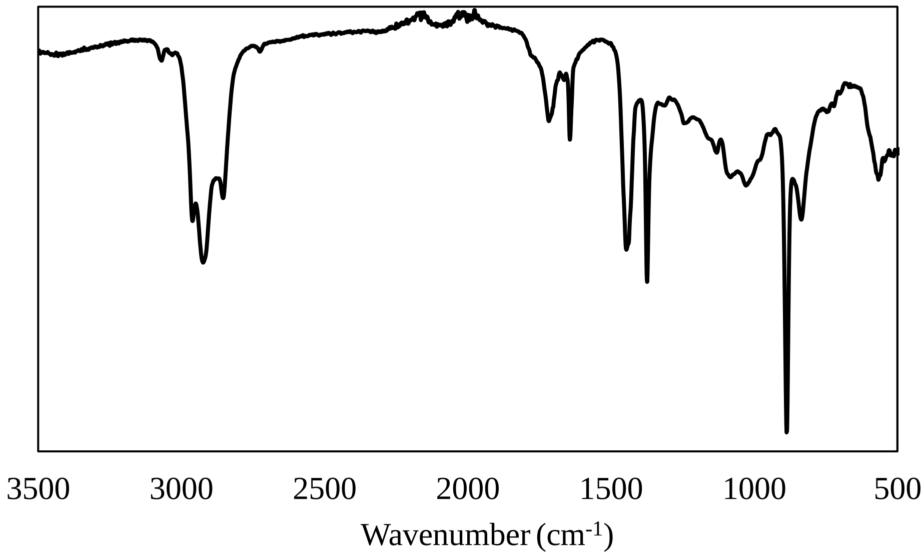

Fig. S221 FT-IR spectrum of PIP 500 equivalents generated by **$Y(CH_2SiMe_3)_3(THF)_2$**, 2 equivalents $[Ph_3C][B(C_6F_5)_4]$, and 1 equivalent PPh_3 from **Table S3**, entry 4 (41 min).

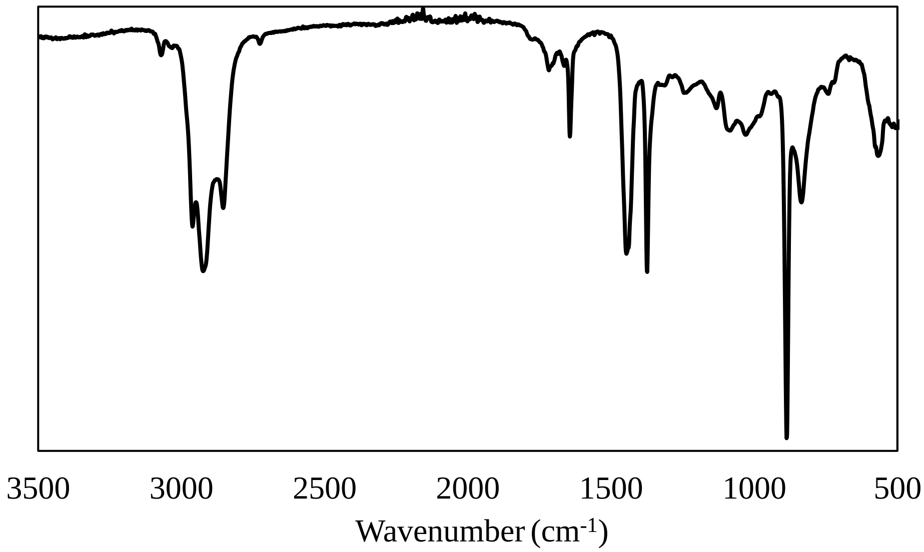

Fig. S222 FT-IR spectrum of PIP 500 equivalents generated by **$Y(CH_2SiMe_3)_3(THF)_2$**, 2 equivalents $[Ph_3C][B(C_6F_5)_4]$, and 1 equivalent PPh_3 from **Table S3**, entry 5 (51 min).

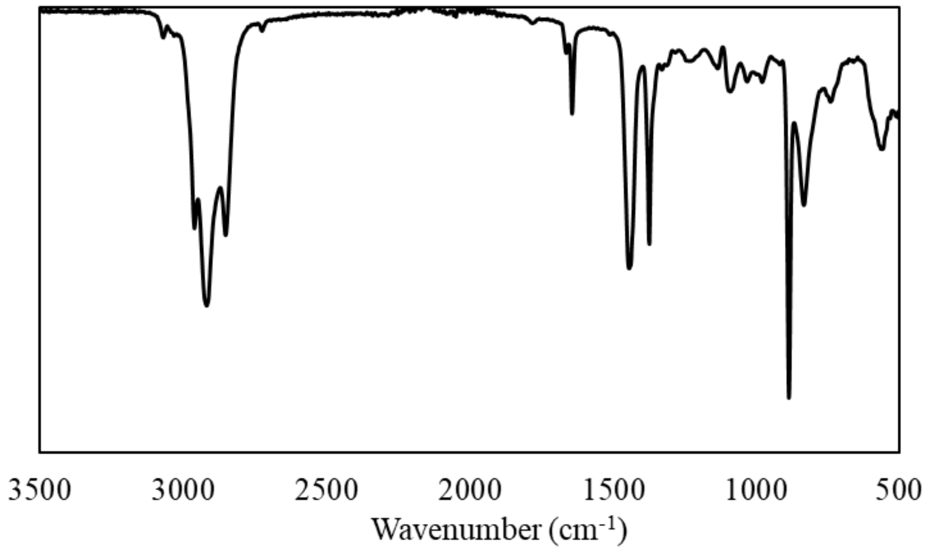

Fig. S223 FT-IR spectrum of PIP 500 equivalents generated by **$Sm(CH_2SiMe_3)_3(THF)_3$** and 1 equivalent $[Ph_3C][B(C_6F_5)_4]$ from **Table S4**, entry 1 (7 h).

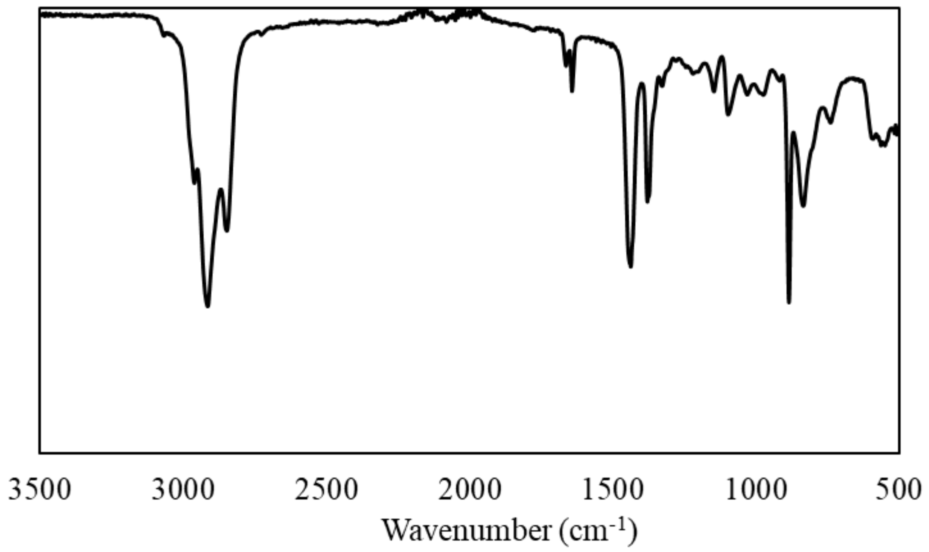

Fig. S224 FT-IR spectrum of PIP 500 equivalents generated by **$Gd(CH_2SiMe_3)_3(THF)_2$** and 1 equivalent $[Ph_3C][B(C_6F_5)_4]$ from **Table S4**, entry 2 (7 h).

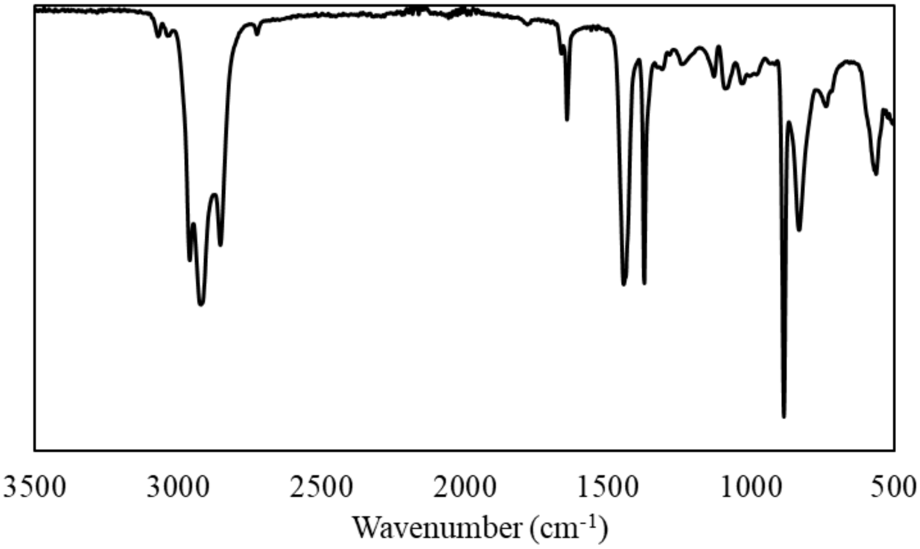

Fig. S225 FT-IR spectrum of PIP 500 equivalents generated by **$Gd(CH_2SiMe_3)_3(THF)_2$** and 2 equivalents $[Ph_3C][B(C_6F_5)_4]$ from **Table S4**, entry 3 (7 h).

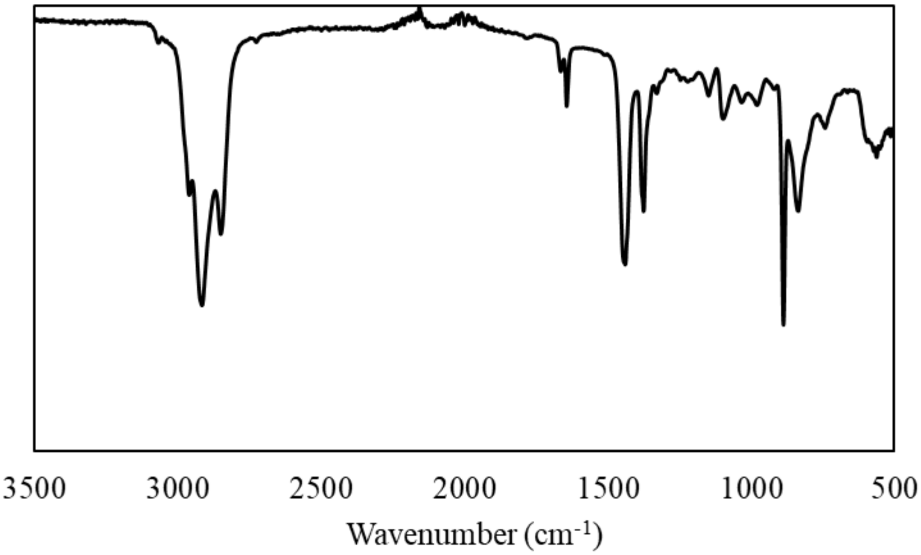

Fig. S226 FT-IR spectrum of PIP 500 equivalents generated by **$Tm(CH_2SiMe_3)_3(THF)_2$** and 1 equivalent $[Ph_3C][B(C_6F_5)_4]$ from **Table S4**, entry 4 (7 h).

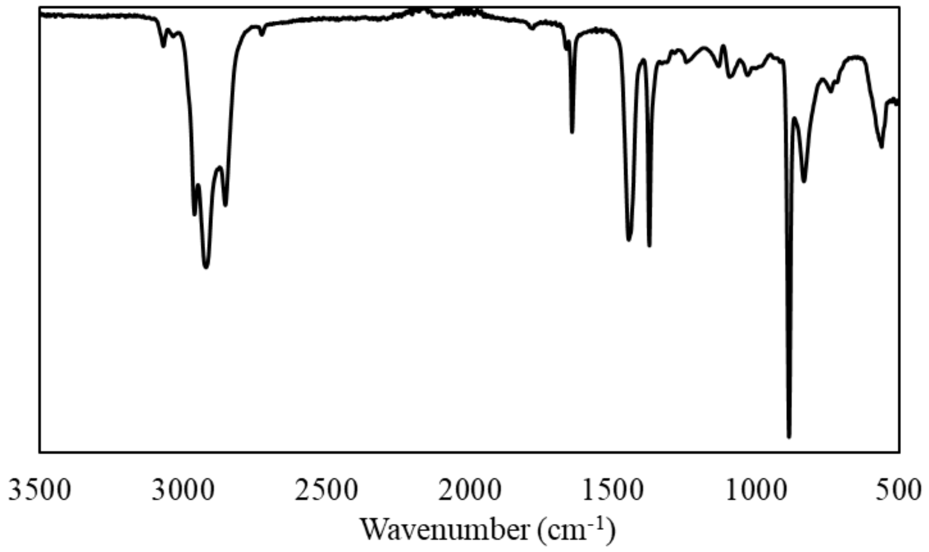

Fig. S227 FT-IR spectrum of PIP 500 equivalents generated by **$Tm(CH_2SiMe_3)_3(THF)_2$** and 2 equivalents $[Ph_3C][B(C_6F_5)_4]$ from **Table S4**, entry 5 (7 h).

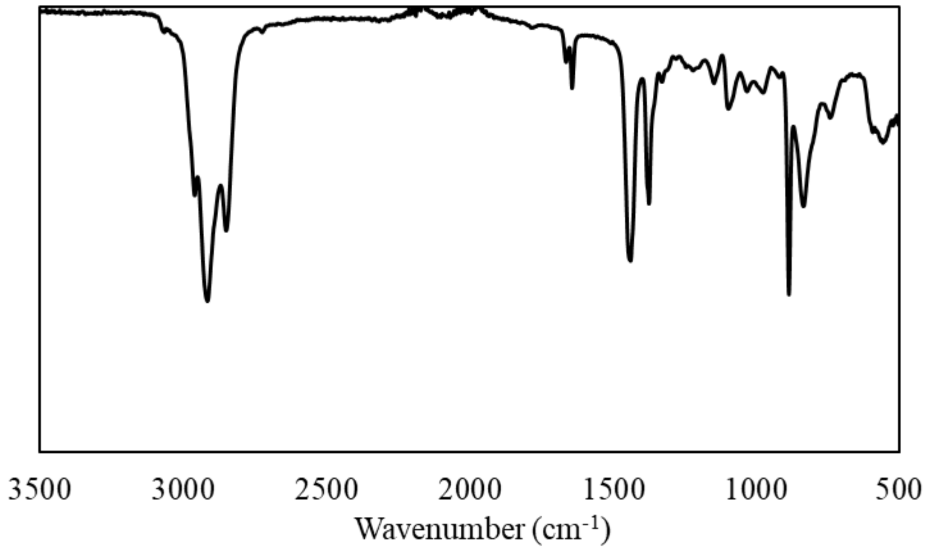

Fig. S228 FT-IR spectrum of PIP 500 equivalents generated by **$Sm(CH_2SiMe_3)_3(THF)_3$**, 1 equivalent $[Ph_3C][B(C_6F_5)_4]$, and 1 equivalent PPh_3 from **Table S4**, entry 6 (7 h).

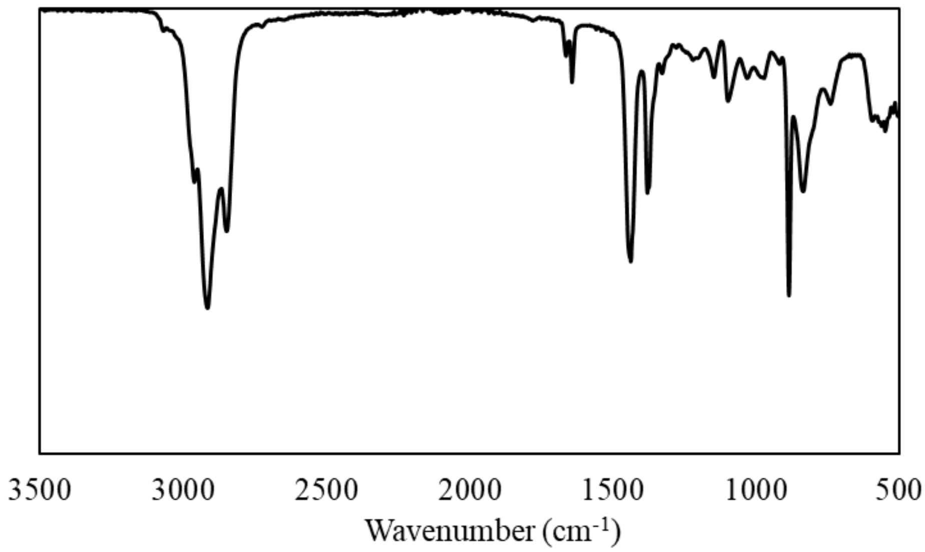

Fig. S229 FT-IR spectrum of PIP 500 equivalents generated by **$Gd(CH_2SiMe_3)_3(THF)_2$**, 1 equivalent $[Ph_3C][B(C_6F_5)_4]$, and 1 equivalent PPh_3 from **Table S4**, entry 7 (7 h).

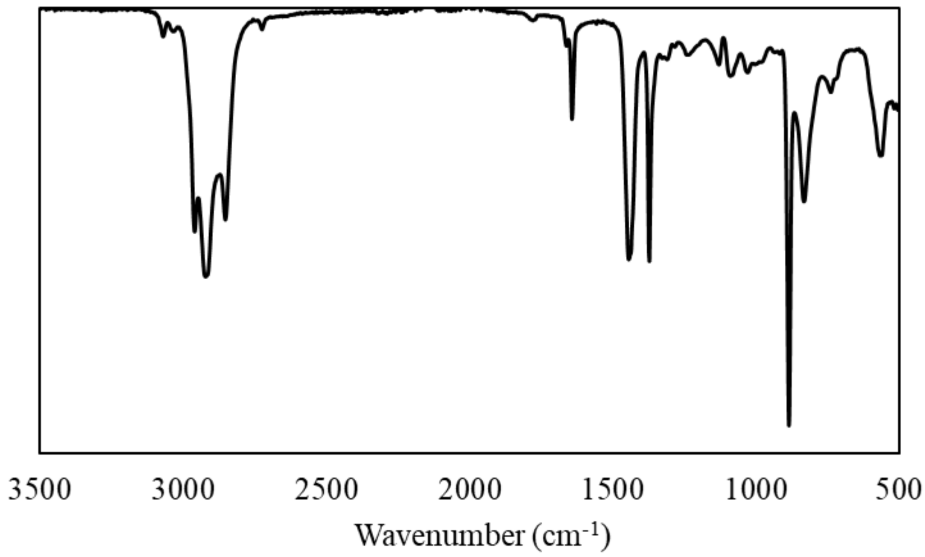

Fig. S230 FT-IR spectrum of PIP 500 equivalents generated by **$Gd(CH_2SiMe_3)_3(THF)_2$**, 2 equivalents $[Ph_3C][B(C_6F_5)_4]$, and 1 equivalent PPh_3 from **Table S4**, entry 8 (7 h).

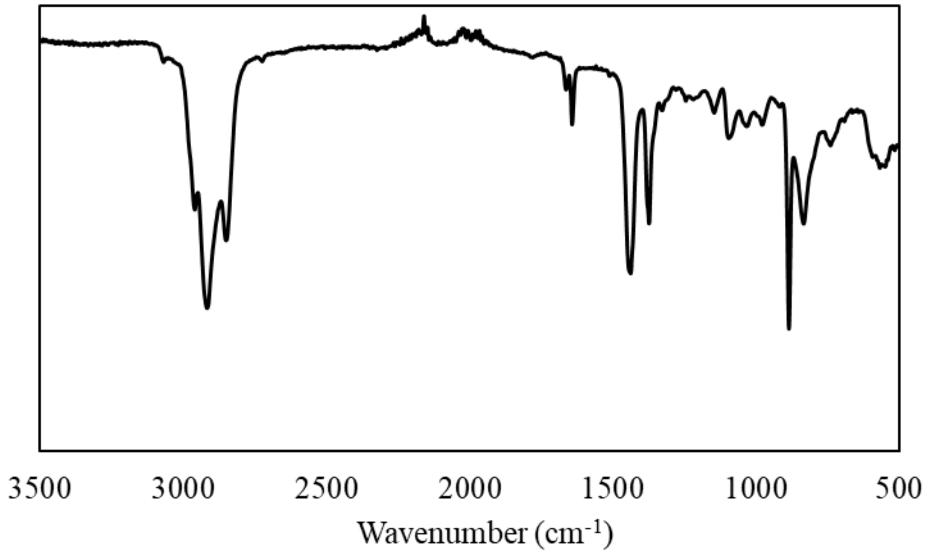

Fig. S231 FT-IR spectrum of PIP 500 equivalents generated by **$Tm(CH_2SiMe_3)_3(THF)_2$**, 1 equivalent $[Ph_3C][B(C_6F_5)_4]$, and 1 equivalent PPh_3 from **Table S4**, entry 9 (7 h).

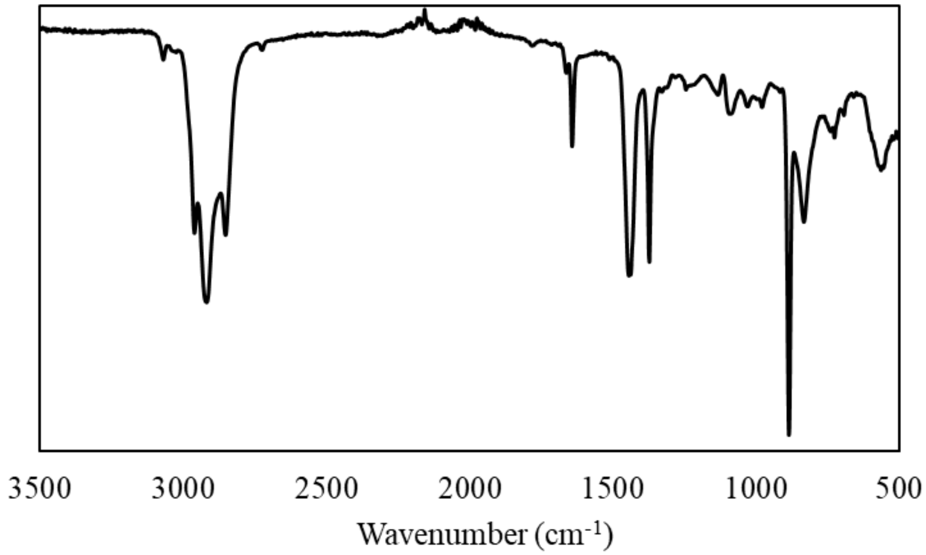

Fig. S232 FT-IR spectrum of PIP 500 equivalents generated by **$Tm(CH_2SiMe_3)_3(THF)_2$**, 2 equivalents $[Ph_3C][B(C_6F_5)_4]$, and 1 equivalent PPh_3 from **Table S4**, entry 10 (7 h).

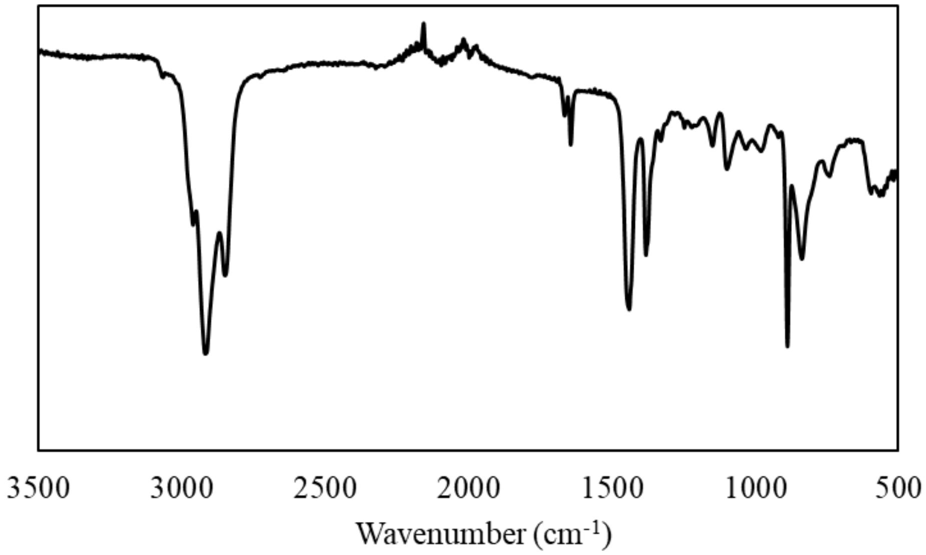

Fig. S233 FT-IR spectrum of PIP 500 equivalents generated by **$Y(CH_2SiMe_3)_3(THF)_2$** and 1 equivalent $[Ph_3C][B(C_6F_5)_4]$ from **Table 3**, entry 1 (7 h).

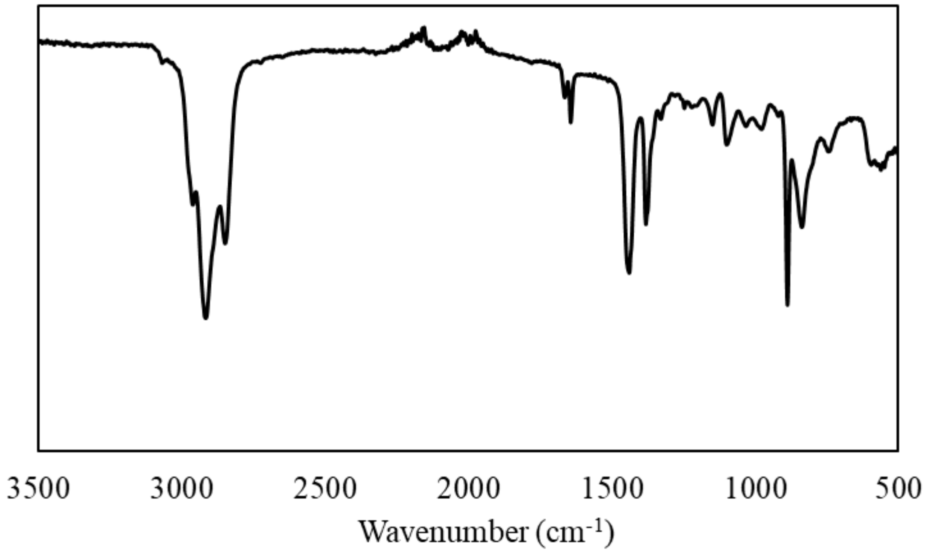

Fig. S234 FT-IR spectrum of PIP 500 equivalents generated by **$Y(CH_2SiMe_3)_3(THF)_2$**, 1 equivalent $[Ph_3C][B(C_6F_5)_4]$, and 1 equivalent PPh_3 from **Table 3**, entry 2 (7 h).

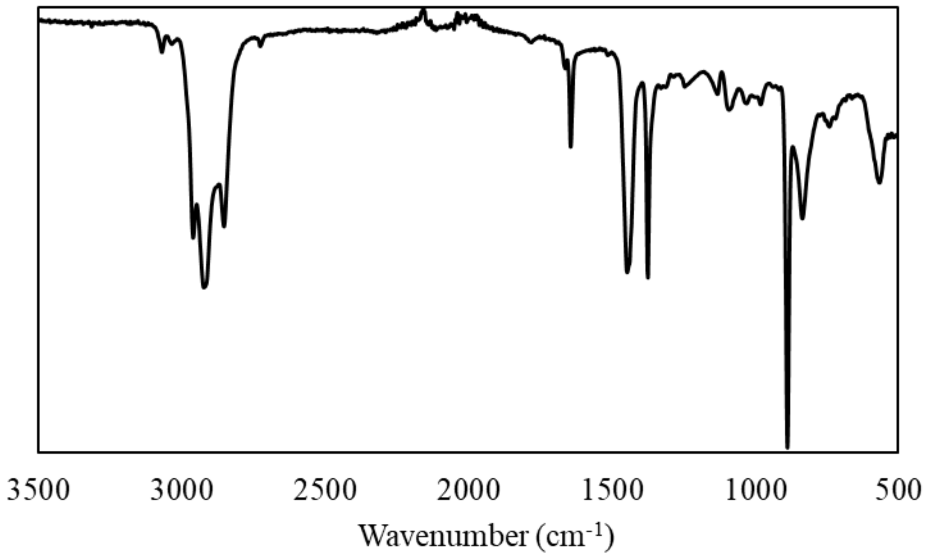

Fig. S235 FT-IR spectrum of PIP 500 equivalents generated by **$Y(CH_2SiMe_3)_3(THF)_2$** and 1.5 equivalents $[Ph_3C][B(C_6F_5)_4]$ from **Table 3**, entry 3 (7 h).

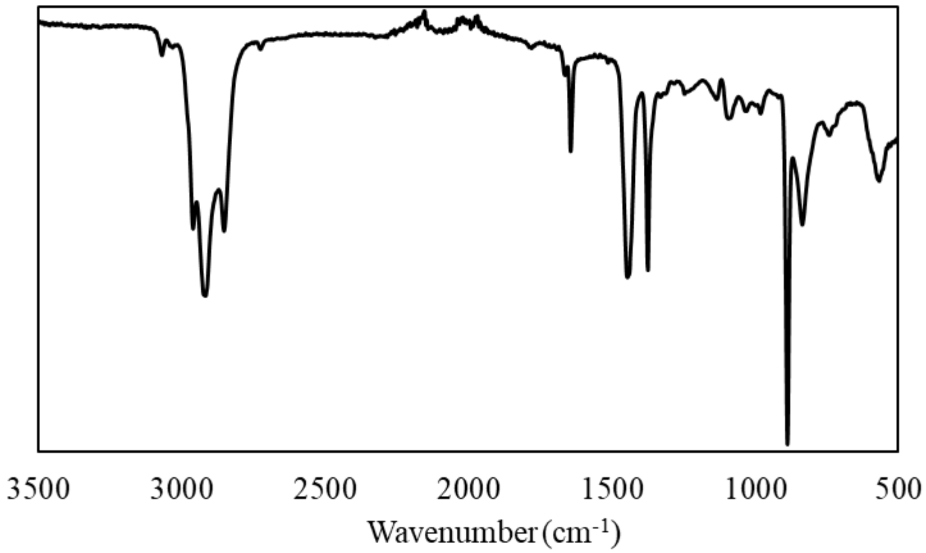

Fig. S236 FT-IR spectrum of PIP 500 equivalents generated by **$Y(CH_2SiMe_3)_3(THF)_2$**, 1.5 equivalents $[Ph_3C][B(C_6F_5)_4]$, and 1 equivalent PPh_3 from **Table 3**, entry 4 (7 h).

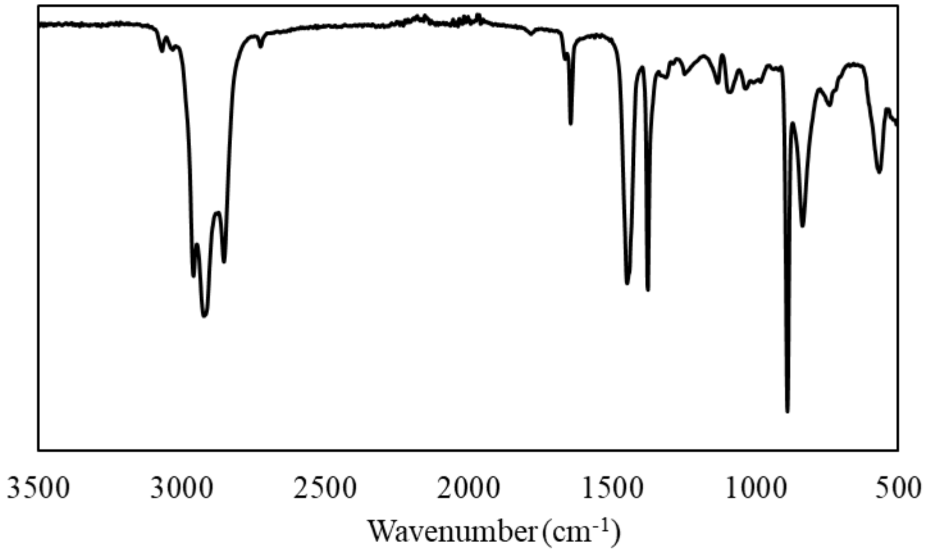

Fig. S237 FT-IR spectrum of PIP 500 equivalents generated by **$Y(CH_2SiMe_3)_3(THF)_2$** and 2 equivalents $[Ph_3C][B(C_6F_5)_4]$ from **Table 3**, entry 5 (7 h).

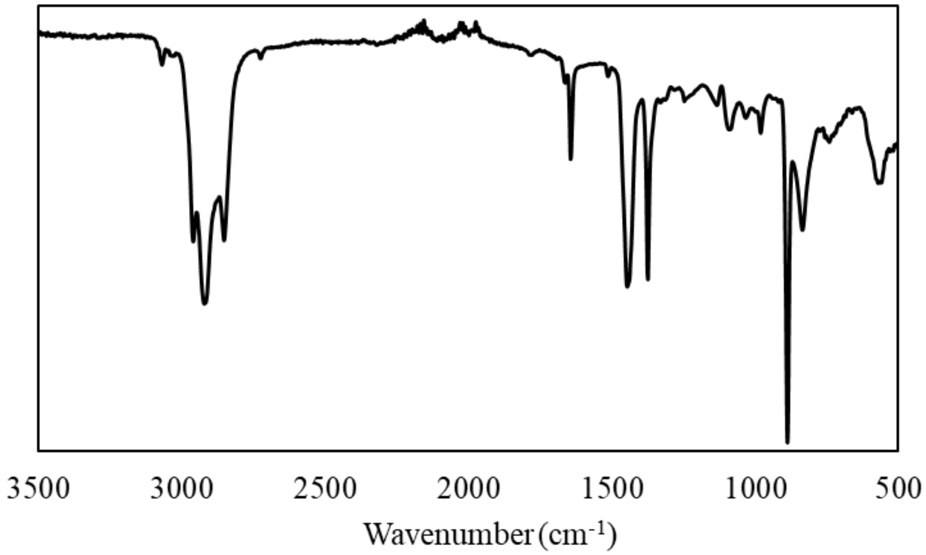

Fig. S238 FT-IR spectrum of PIP 500 equivalents generated by **$Y(CH_2SiMe_3)_3(THF)_2$**, 2 equivalents $[Ph_3C][B(C_6F_5)_4]$, and 1 equivalent PPh_3 from **Table 3**, entry 6 (7 h).

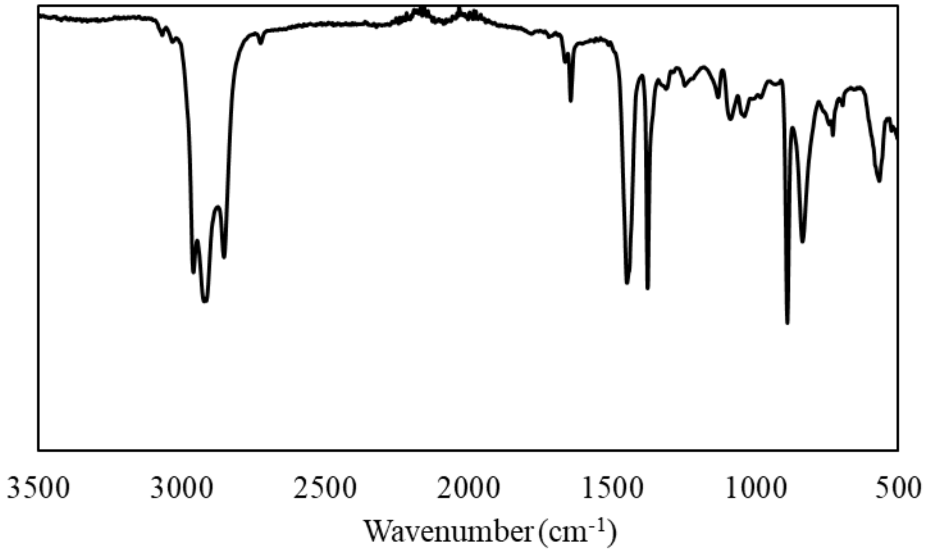

Fig. S239 FT-IR spectrum of PIP 500 equivalents generated by **$Y(CH_2SiMe_3)_3(THF)_2$** and 2.5 equivalents $[Ph_3C][B(C_6F_5)_4]$ from **Table 3**, entry 7 (7 h).

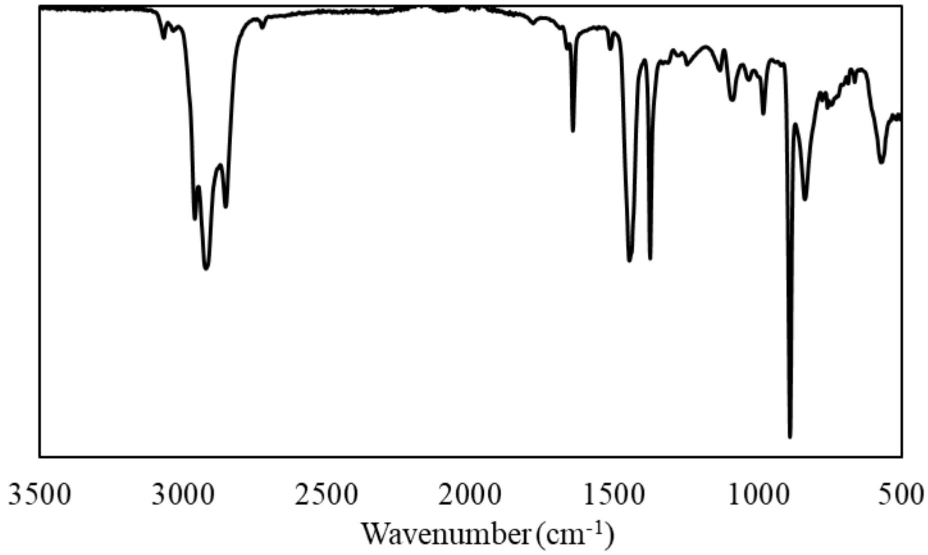

Fig. S240 FT-IR spectrum of PIP 500 equivalents generated by **$Y(CH_2SiMe_3)_3(THF)_2$**, 2.5 equivalents $[Ph_3C][B(C_6F_5)_4]$, and 1 equivalent PPh_3 from **Table 3**, entry 8 (7 h).

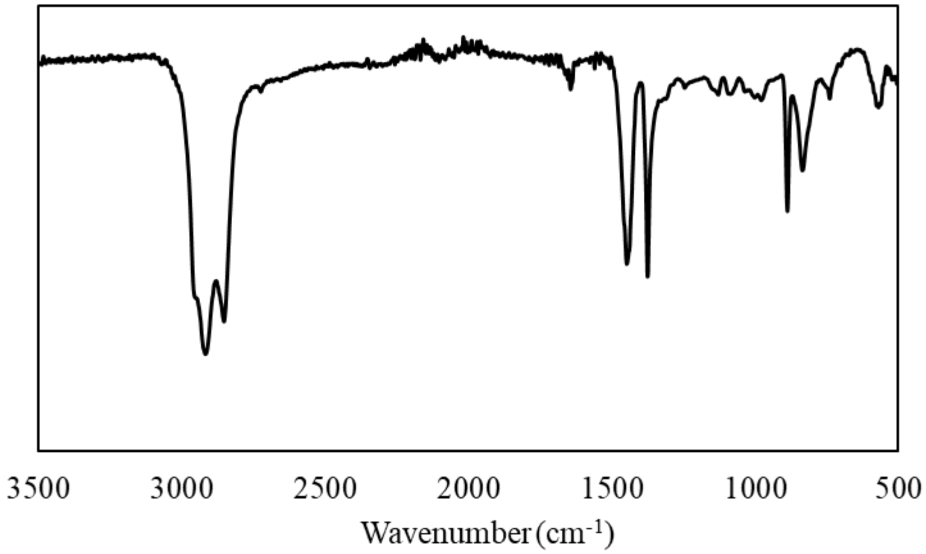

Fig. S241 FT-IR spectrum of PIP 500 equivalents generated by **$Y(CH_2SiMe_3)_3(THF)_2$** and 3 equivalents $[Ph_3C][B(C_6F_5)_4]$ from **Table 3**, entry 9 (7 h).

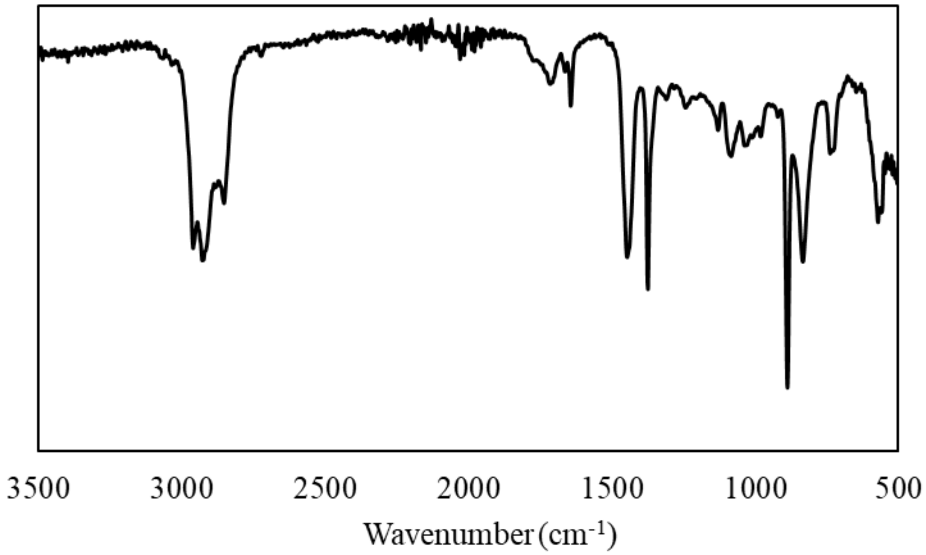

Fig. S242 FT-IR spectrum of PIP 500 equivalents generated by **$Y(CH_2SiMe_3)_3(THF)_2$**, 3 equivalents $[Ph_3C][B(C_6F_5)_4]$, and 1 equivalent PPh_3 from **Table 3**, entry 10 (7 h).

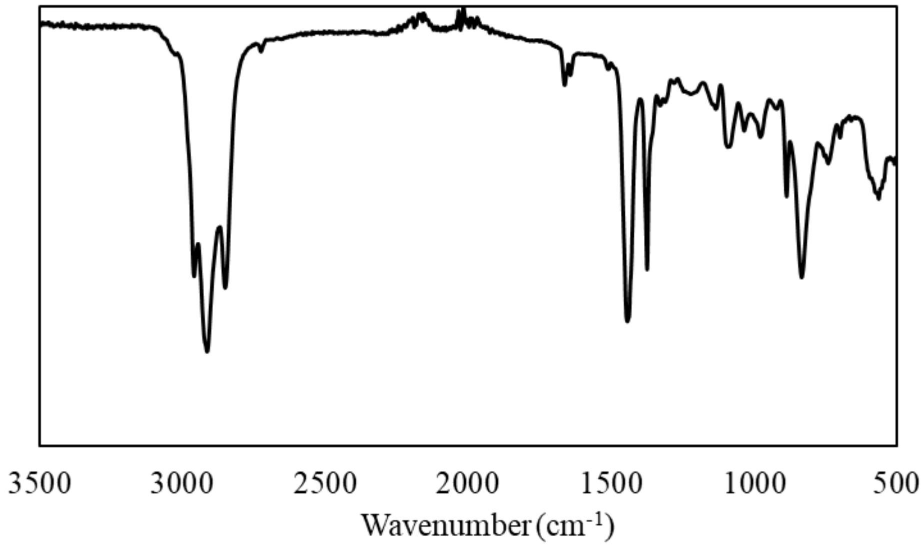

Fig. S243 FT-IR spectrum of PIP 500 equivalents generated by **$Y(CH_2SiMe_3)_3(THF)_2$**, 2 equivalents $[Ph_3C][B(C_6F_5)_4]$, and 5 equivalents $AlMe_3$ from **Table 4**, entry 1 (30 min).

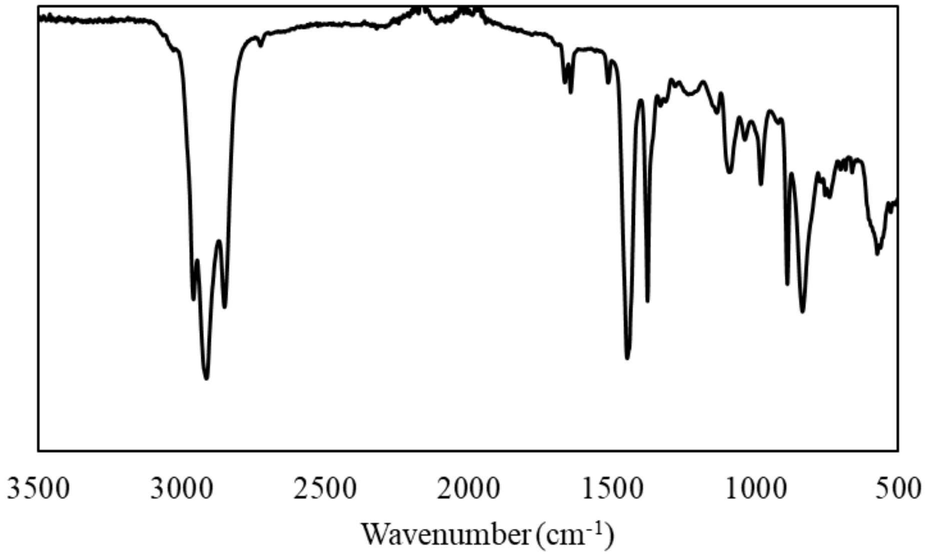

Fig. S244 FT-IR spectrum of PIP 500 equivalents generated by **$Y(CH_2SiMe_3)_3(THF)_2$**, 2 equivalents $[Ph_3C][B(C_6F_5)_4]$, and 10 equivalents $AlMe_3$ from **Table 4**, entry 2 (30 min).

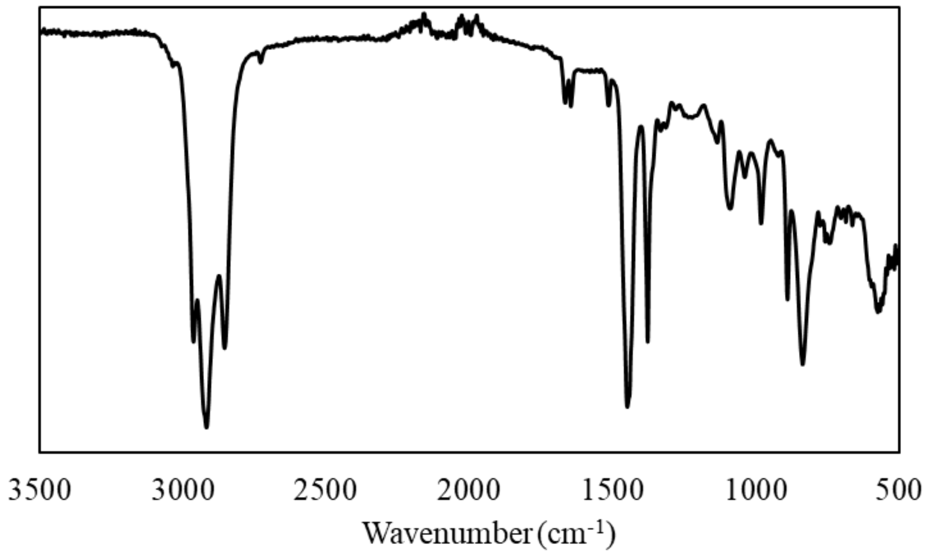

Fig. S245 FT-IR spectrum of PIP 500 equivalents generated by **$Y(CH_2SiMe_3)_3(THF)_2$**, 2 equivalents $[Ph_3C][B(C_6F_5)_4]$, and 15 equivalents $AlMe_3$ from **Table 4**, entry 3 (30 min).

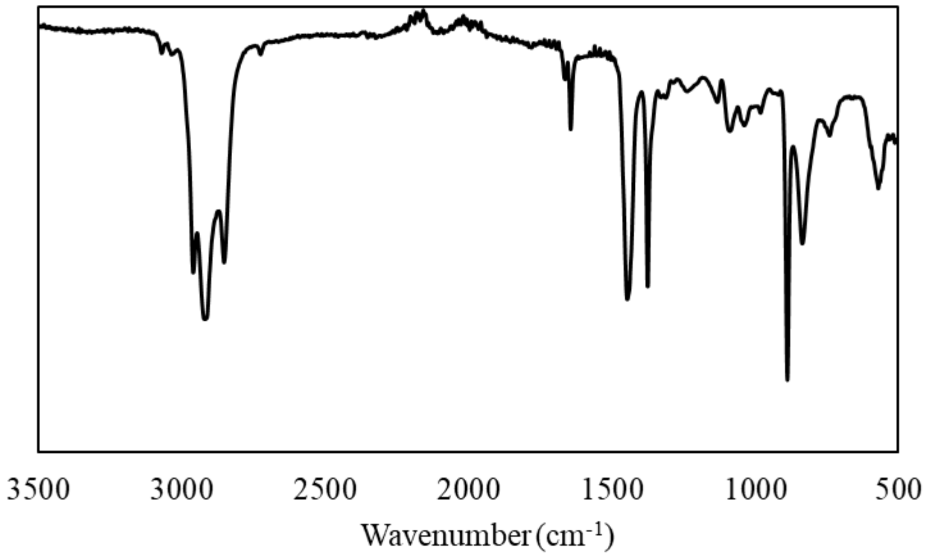

Fig. S246 FT-IR spectrum of PIP 500 equivalents generated by **$Y(CH_2SiMe_3)_3(THF)_2$**, 2 equivalents $[Ph_3C][B(C_6F_5)_4]$, and 5 equivalents $AlEt_3$ from **Table 4**, entry 4 (30 min).

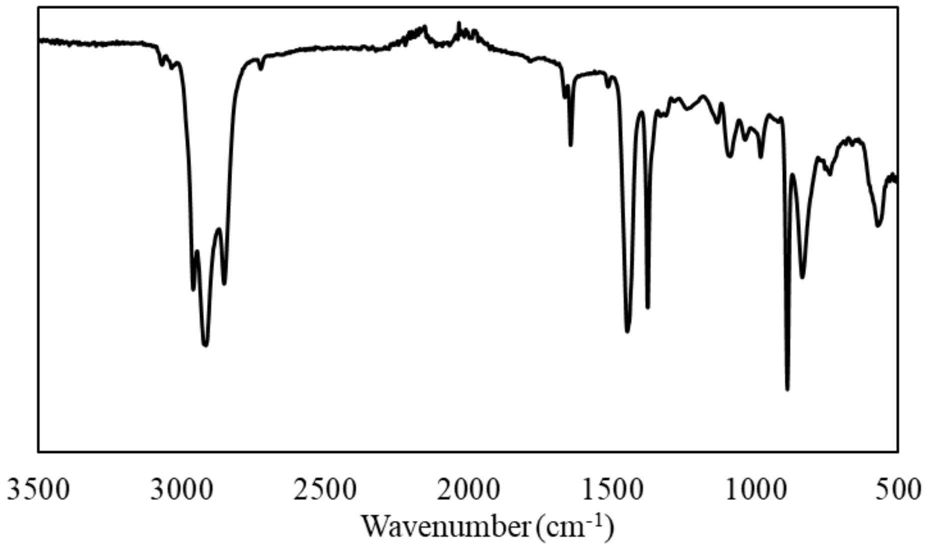

Fig. S247 FT-IR spectrum of PIP 500 equivalents generated by **$Y(CH_2SiMe_3)_3(THF)_2$**, 2 equivalents $[Ph_3C][B(C_6F_5)_4]$, and 10 equivalents $AlEt_3$ from **Table 4**, entry 5 (30 min).

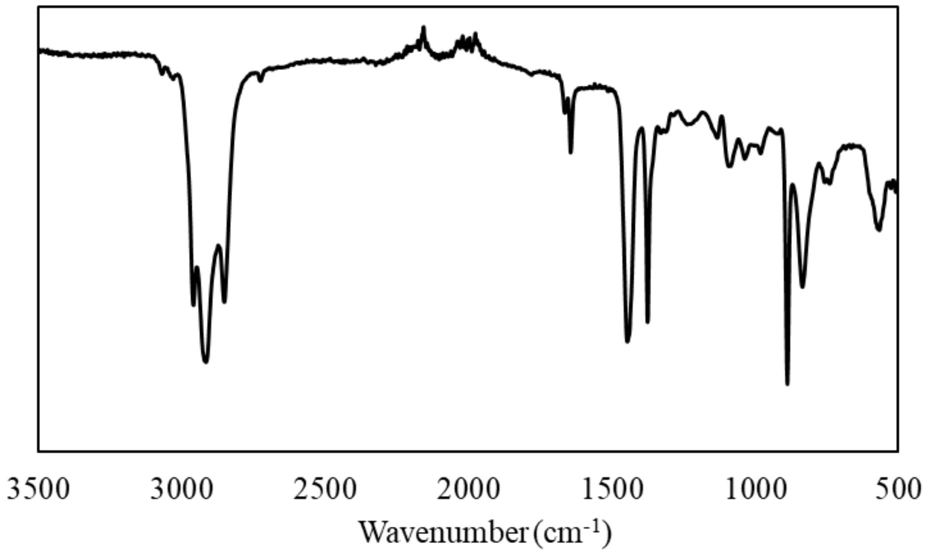

Fig. S248 FT-IR spectrum of PIP 500 equivalents generated by **$Y(CH_2SiMe_3)_3(THF)_2$**, 2 equivalents $[Ph_3C][B(C_6F_5)_4]$, and 15 equivalents $AlEt_3$ from **Table 4**, entry 6 (30 min).

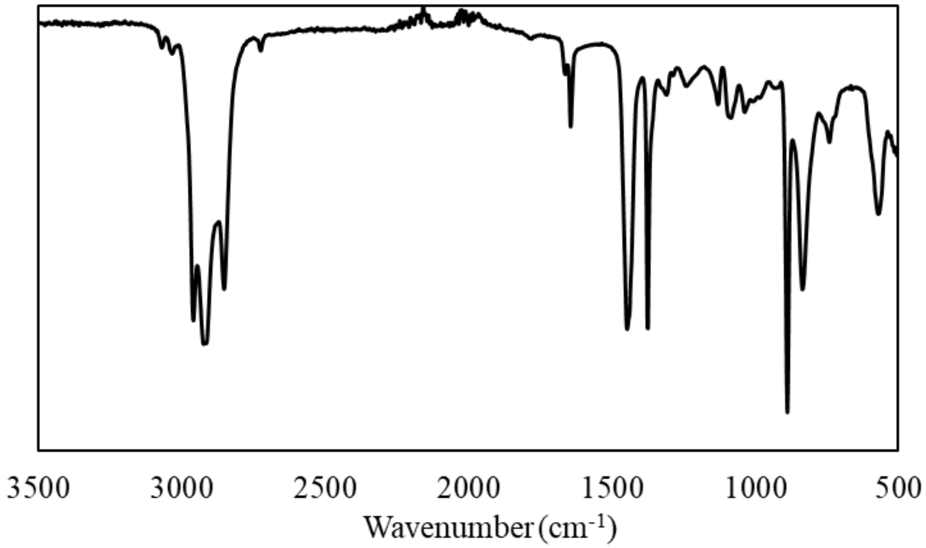

Fig. S249 FT-IR spectrum of PIP 500 equivalents generated by **$Y(CH_2SiMe_3)_3(THF)_2$**, 2 equivalents $[Ph_3C][B(C_6F_5)_4]$, and 5 equivalents Al^iBu_3 from **Table 4**, entry 7 (30 min).

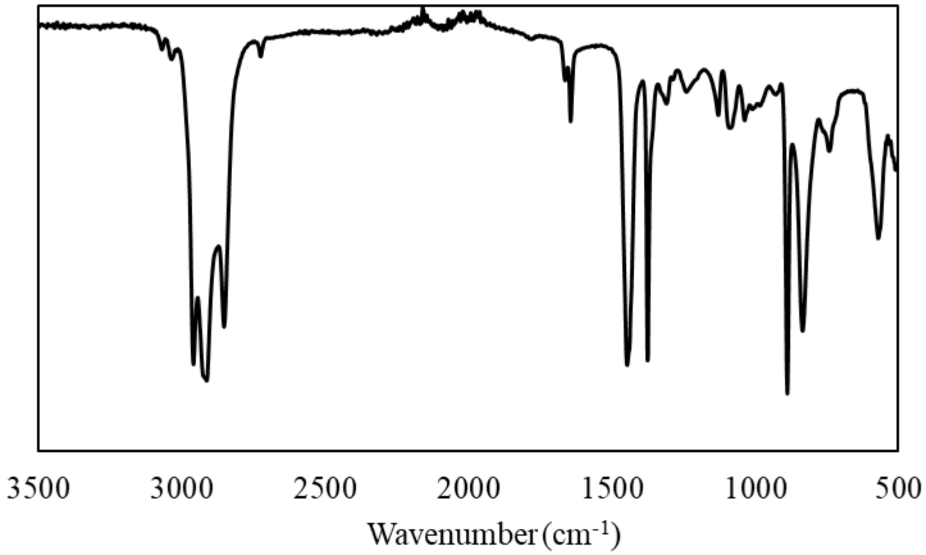

Fig. S250 FT-IR spectrum of PIP 500 equivalents generated by **$Y(CH_2SiMe_3)_3(THF)_2$**, 2 equivalents $[Ph_3C][B(C_6F_5)_4]$, and 10 equivalents Al^iBu_3 from **Table 4**, entry 8 (30 min).

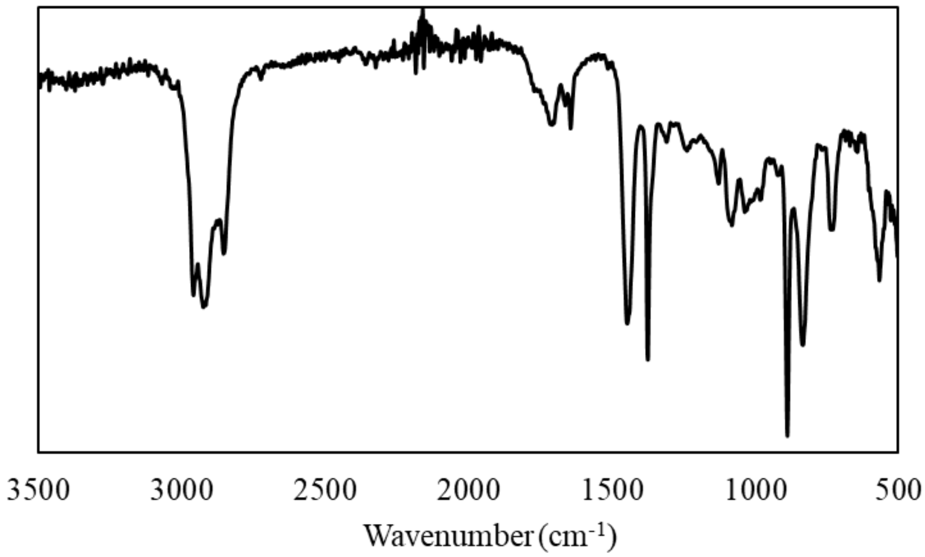

Fig. S251 FT-IR spectrum of PIP 500 equivalents generated by **Y(CH$_2$SiMe$_3$)$_3$(THF)$_2$**, 2 equivalents [Ph$_3$C][B(C$_6$F$_5$)$_4$], and 15 equivalents Al^{i}Bu$_3$ from **Table 4**, entry 9 (30 min).

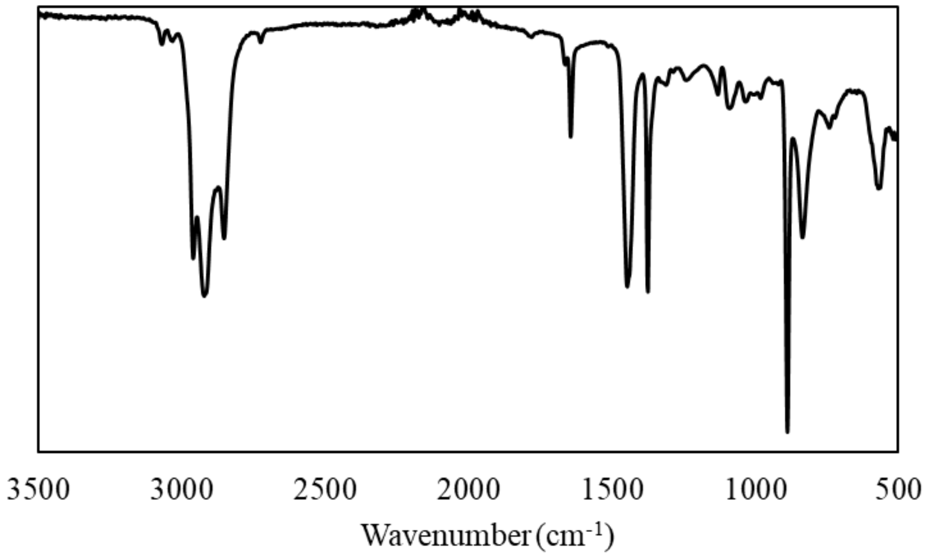

Fig. S252 FT-IR spectrum of PIP 500 equivalents generated by **Y(CH$_2$SiMe$_3$)$_3$(THF)$_2$**, 2 equivalents [Ph$_3$C][B(C$_6$F$_5$)$_4$], 1 equivalent PPh$_3$, and 5 equivalents Al^{i}Bu$_3$ from **Table 4**, entry 10 (30 min).

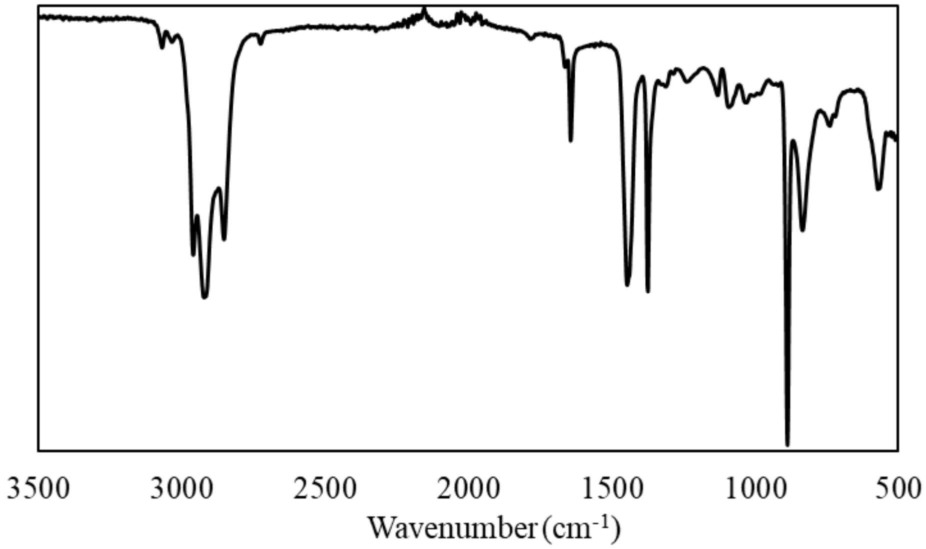

Fig. S253 FT-IR spectrum of PIP 500 equivalents generated by **$Y(CH_2SiMe_3)_3(THF)_2$**, 2 equivalents $[Ph_3C][B(C_6F_5)_4]$, 1 equivalent PPh_3, and 10 equivalents Al^iBu_3 from **Table 4**, entry 11 (30 min).

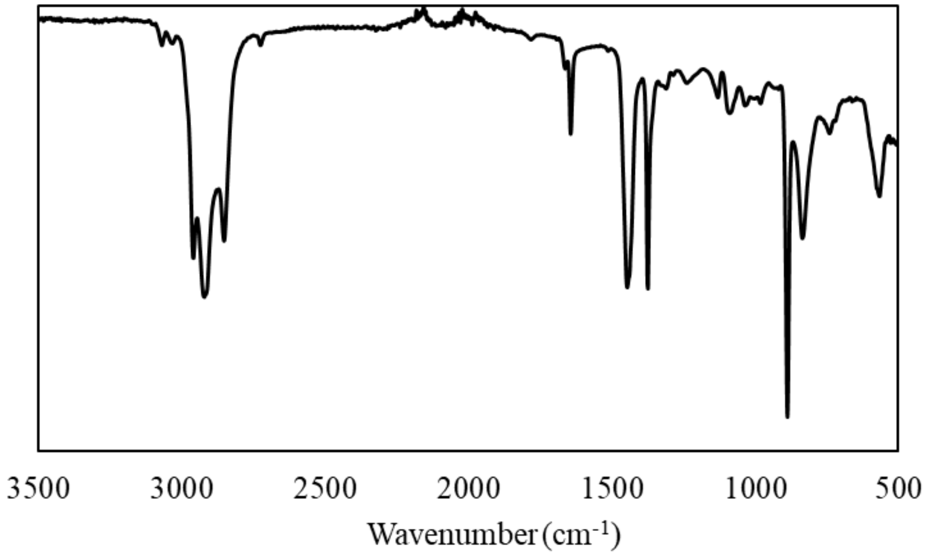

Fig. S254 FT-IR spectrum of PIP 500 equivalents generated by **$Y(CH_2SiMe_3)_3(THF)_2$**, 2 equivalents $[Ph_3C][B(C_6F_5)_4]$, 1 equivalent PPh_3, and 15 equivalents Al^iBu_3 from **Table 4**, entry 12 (30 min).

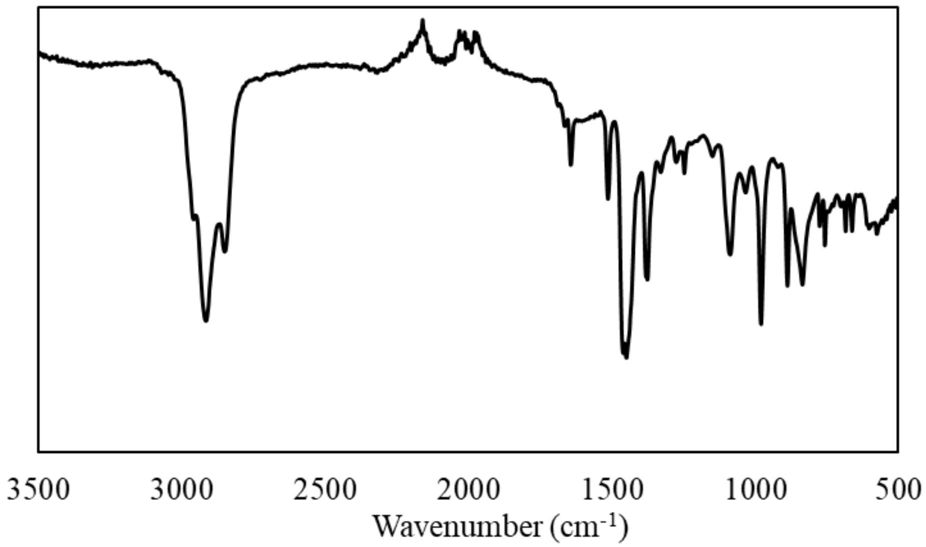

Fig. S255 FT-IR spectrum of PIP 500 equivalents generated by **$Sm(CH_2SiMe_3)_3(THF)_3$** and 1 equivalent $[Ph_3C][B(C_6F_5)_4]$ from **Table 5**, entry 1 (30 min).

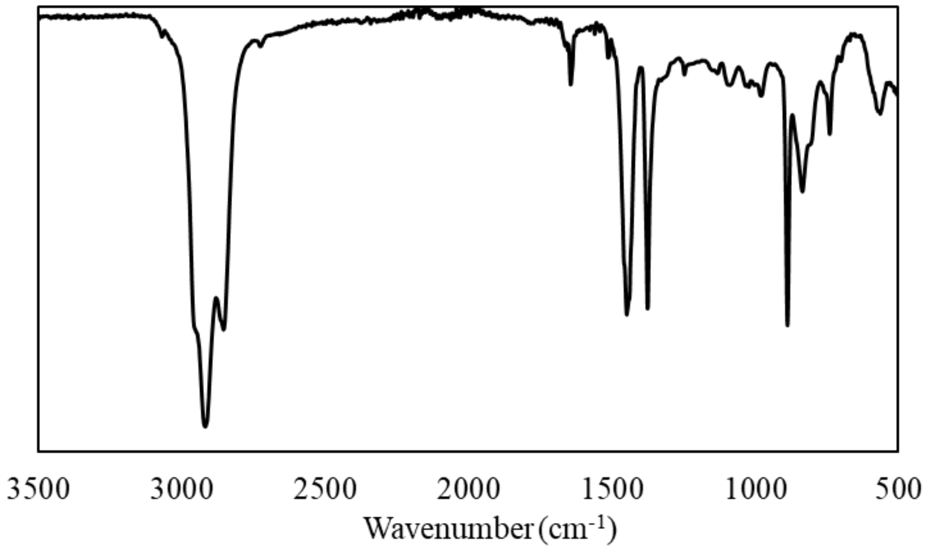

Fig. S256 FT-IR spectrum of PIP 500 equivalents generated by **$Sm(CH_2SiMe_3)_3(THF)_3$** and 2 equivalents $[Ph_3C][B(C_6F_5)_4]$ from **Table 5**, entry 2 (30 min).

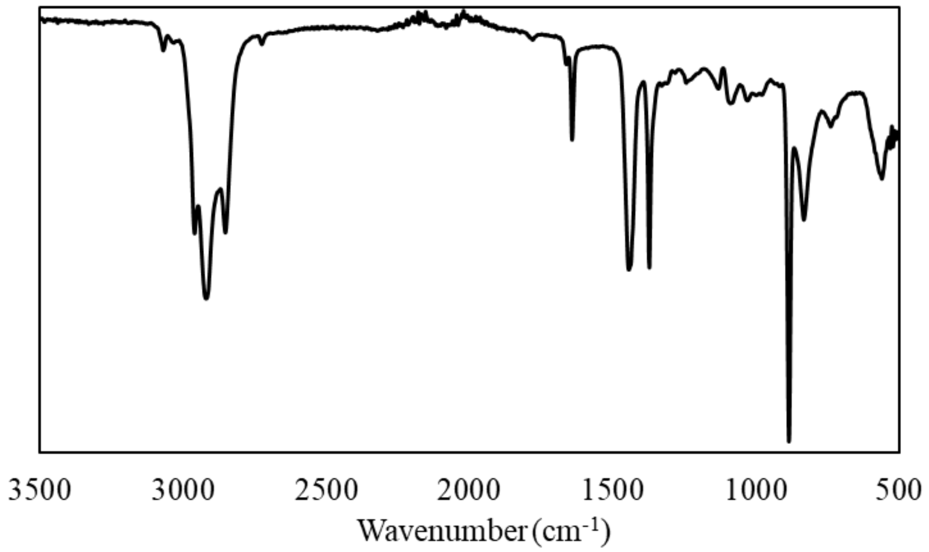

Fig. S257 FT-IR spectrum of PIP 500 equivalents generated by **Gd(CH_2SiMe_3)$_3$(THF)$_2$** and 1 equivalent [Ph_3C][B(C_6F_5)$_4$] from **Table 5**, entry 3 (30 min).

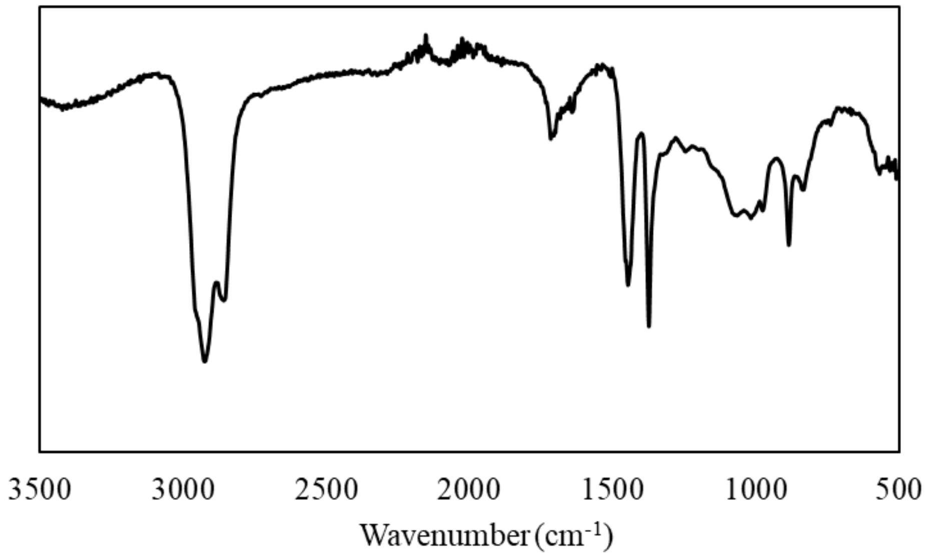

Fig. S258 FT-IR spectrum of PIP 500 equivalents generated by **Gd(CH_2SiMe_3)$_3$(THF)$_2$** and 2 equivalents [Ph_3C][B(C_6F_5)$_4$] from **Table 5**, entry 4 (30 min).

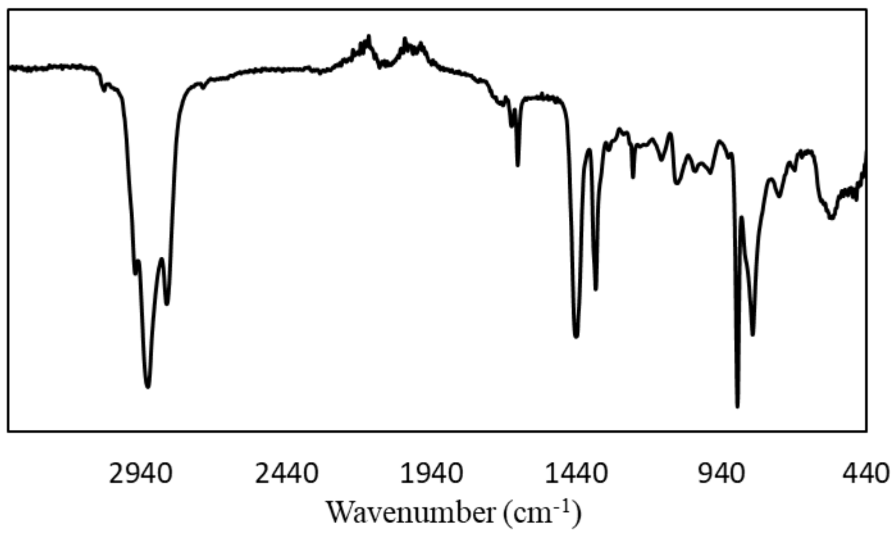

Fig. S259 FT-IR spectrum of PIP 500 equivalents generated by **$Y(CH_2SiMe_3)_3(THF)_2$** and 1 equivalent $[Ph_3C][B(C_6F_5)_4]$ from **Table 5**, entry 5 (30 min).

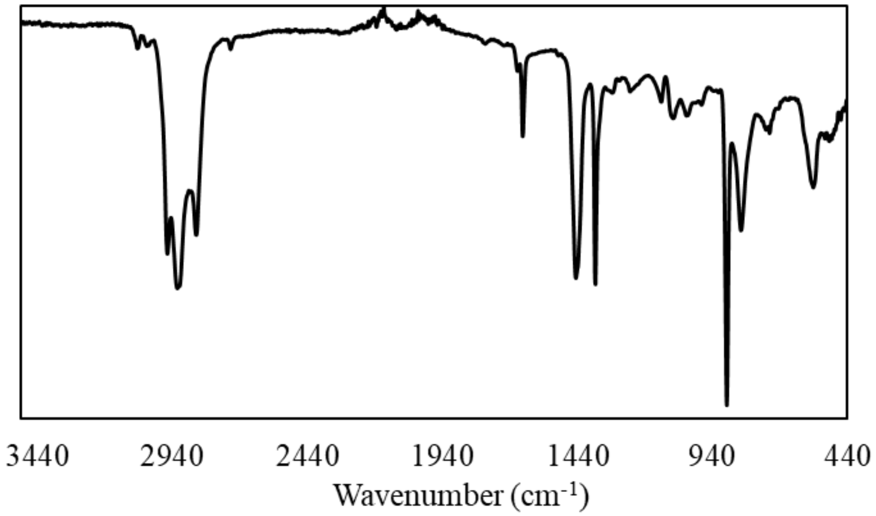

Fig. S260 FT-IR spectrum of PIP 500 equivalents generated by **$Y(CH_2SiMe_3)_3(THF)_2$** and 2 equivalents $[Ph_3C][B(C_6F_5)_4]$ from **Table 5**, entry 6 (30 min).

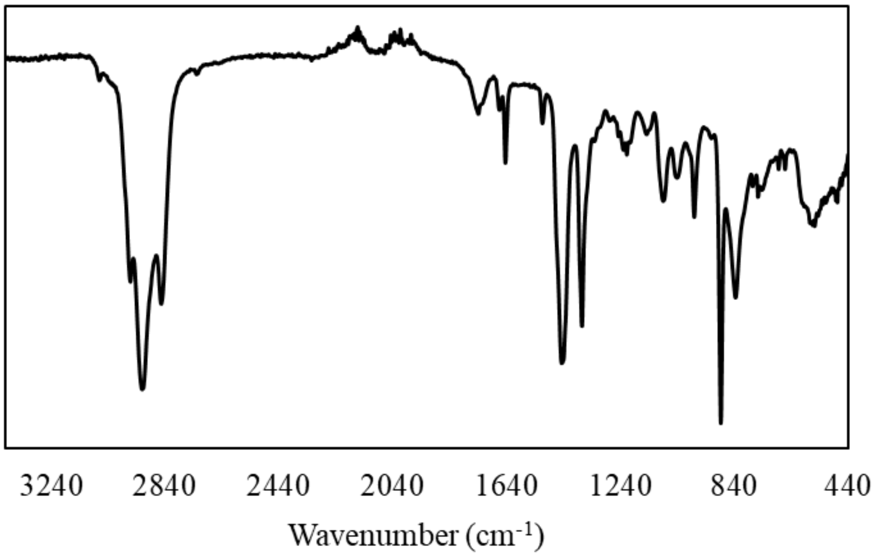

Fig. S261 FT-IR spectrum of PIP 500 equivalents generated by **Tm(CH_2SiMe_3)$_3$(THF)$_2$** and 1 equivalent [Ph_3C][B(C_6F_5)$_4$] from **Table 5**, entry 7 (30 min).

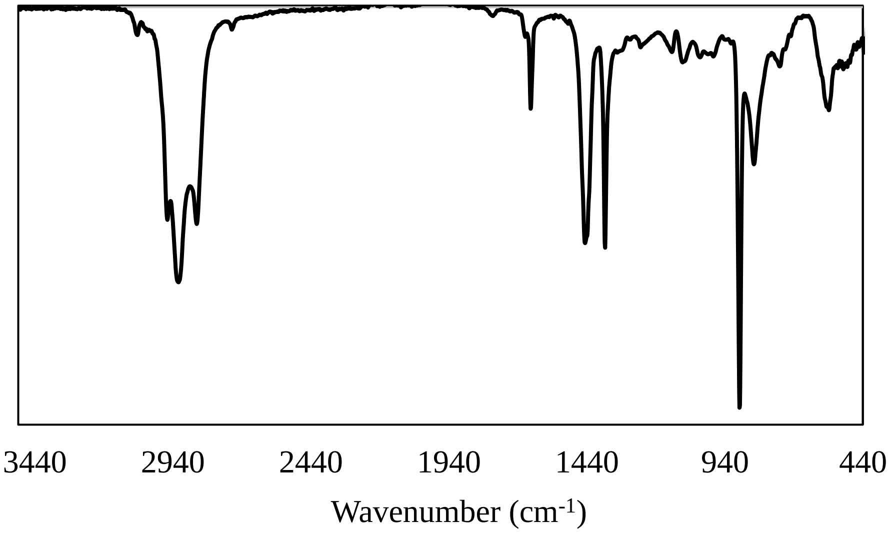

Fig. S262 FT-IR spectrum of PIP 500 equivalents generated by **Tm(CH_2SiMe_3)$_3$(THF)$_2$** and 2 equivalents [Ph_3C][B(C_6F_5)$_4$] from **Table 5**, entry 8 (30 min).

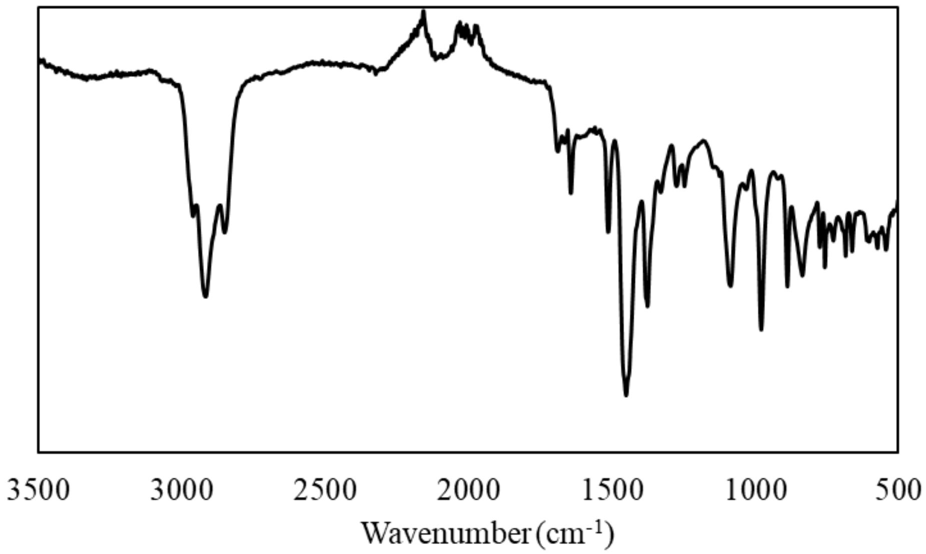

Fig. S263 FT-IR spectrum of PIP 500 equivalents generated by **$Sm(CH_2SiMe_3)_3(THF)_3$**, 1 equivalent $[Ph_3C][B(C_6F_5)_4]$, and 1 equivalent PPh_3 from **Table 5**, entry 9 (30 min).

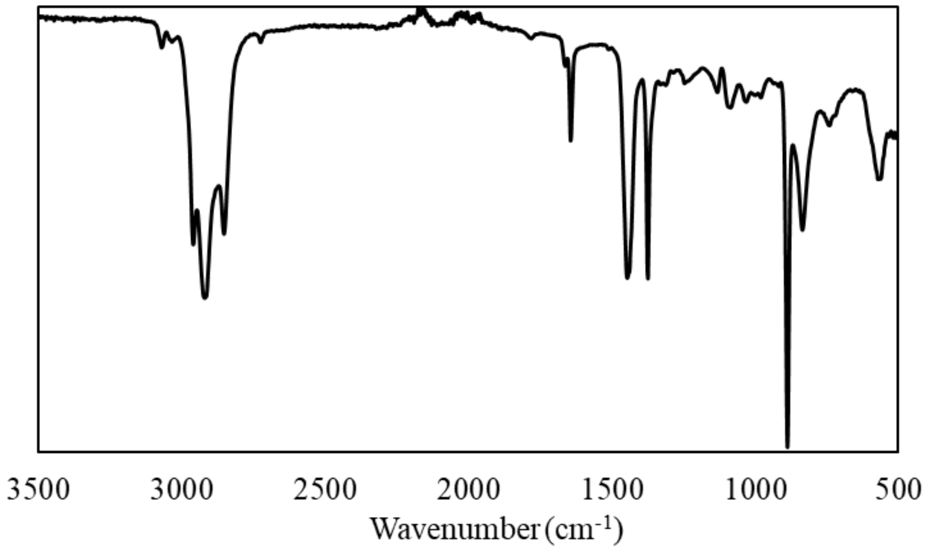

Fig. S264 FT-IR spectrum of PIP 500 equivalents generated by **$Sm(CH_2SiMe_3)_3(THF)_3$**, 2 equivalents $[Ph_3C][B(C_6F_5)_4]$, and 1 equivalent PPh_3 from **Table 5**, entry 10 (30 min).

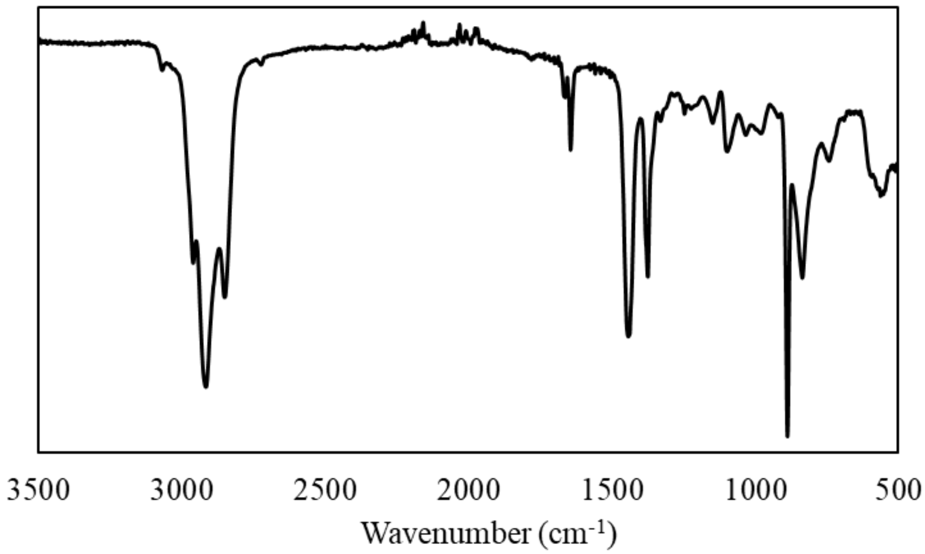

Fig. S265 FT-IR spectrum of PIP 500 equivalents generated by **$Gd(CH_2SiMe_3)_3(THF)_2$**, 1 equivalent $[Ph_3C][B(C_6F_5)_4]$, and 1 equivalent PPh_3 from **Table 5**, entry 11 (30 min).

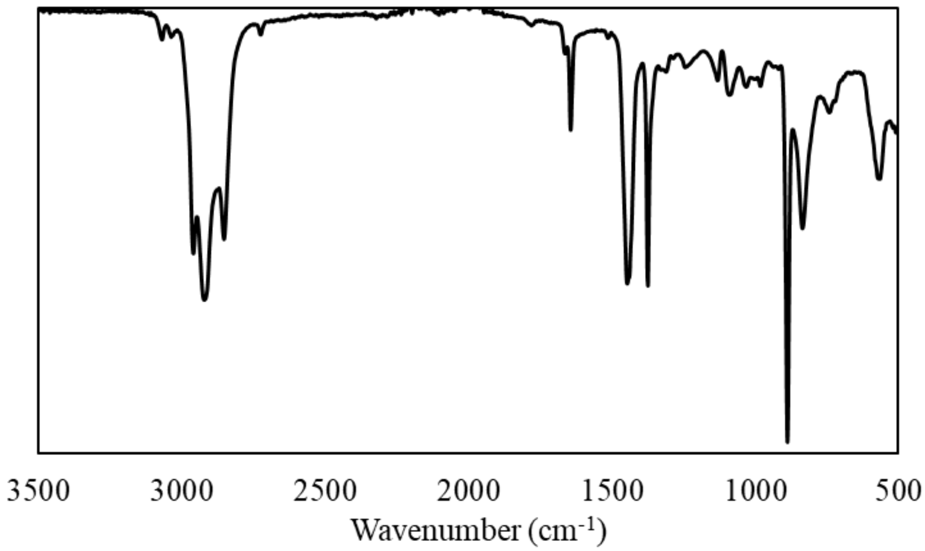

Fig. S266 FT-IR spectrum of PIP 500 equivalents generated by **$Gd(CH_2SiMe_3)_3(THF)_2$**, 2 equivalents $[Ph_3C][B(C_6F_5)_4]$, and 1 equivalent PPh_3 from **Table 5**, entry 12 (30 min).

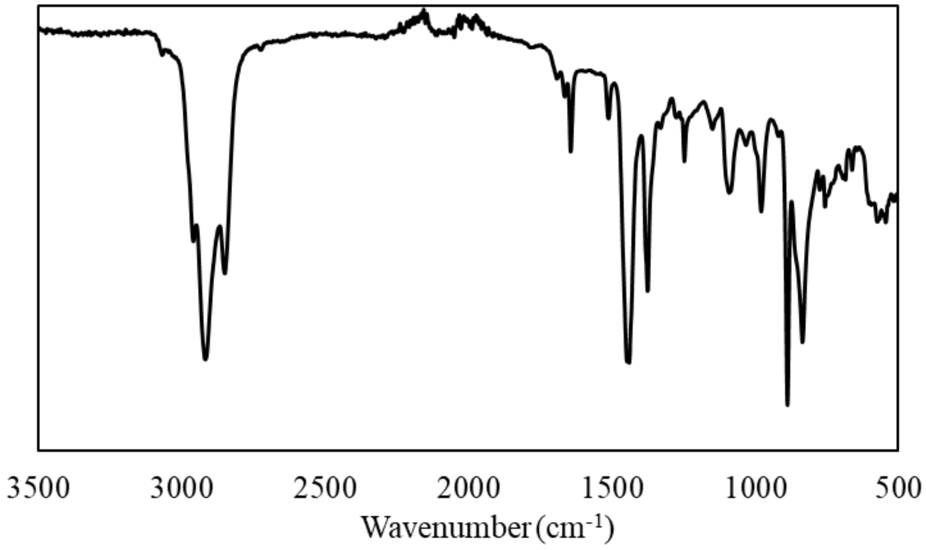

Fig. S267 FT-IR spectrum of PIP 500 equivalents generated by **$Y(CH_2SiMe_3)_3(THF)_2$**, 1 equivalent $[Ph_3C][B(C_6F_5)_4]$, and 1 equivalent PPh_3 from **Table 5**, entry 13 (30 min).

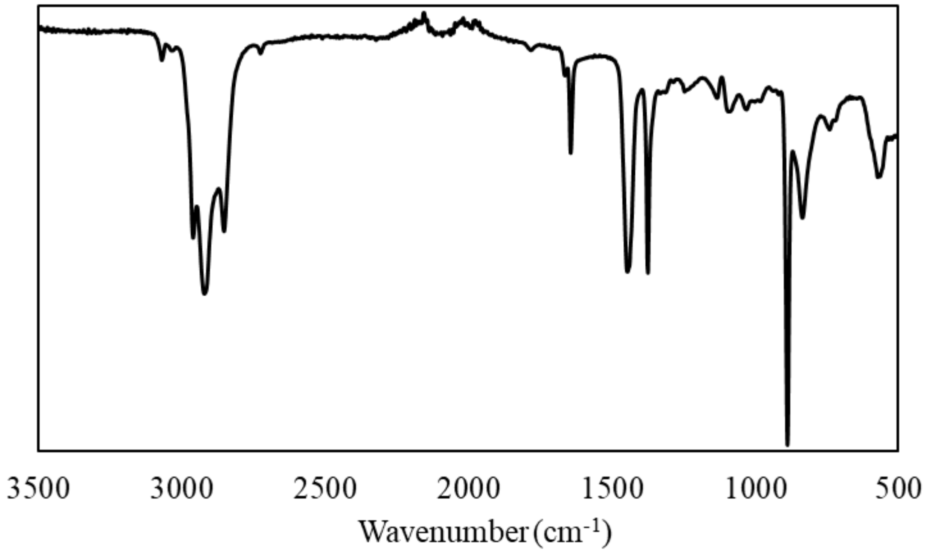

Fig. S268 FT-IR spectrum of PIP 500 equivalents generated by **$Y(CH_2SiMe_3)_3(THF)_2$**, 2 equivalents $[Ph_3C][B(C_6F_5)_4]$, and 1 equivalent PPh_3 from **Table 5**, entry 14 (30 min).

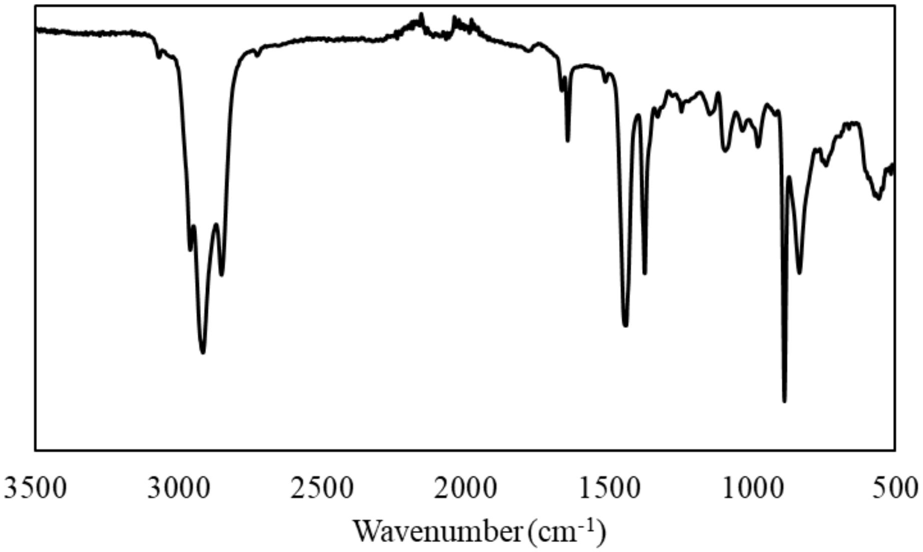

Fig. S269 FT-IR spectrum of PIP 500 equivalents generated by **$Tm(CH_2SiMe_3)_3(THF)_2$**, 1 equivalent $[Ph_3C][B(C_6F_5)_4]$, and 1 equivalent PPh_3 from **Table 5**, entry 15 (30 min).

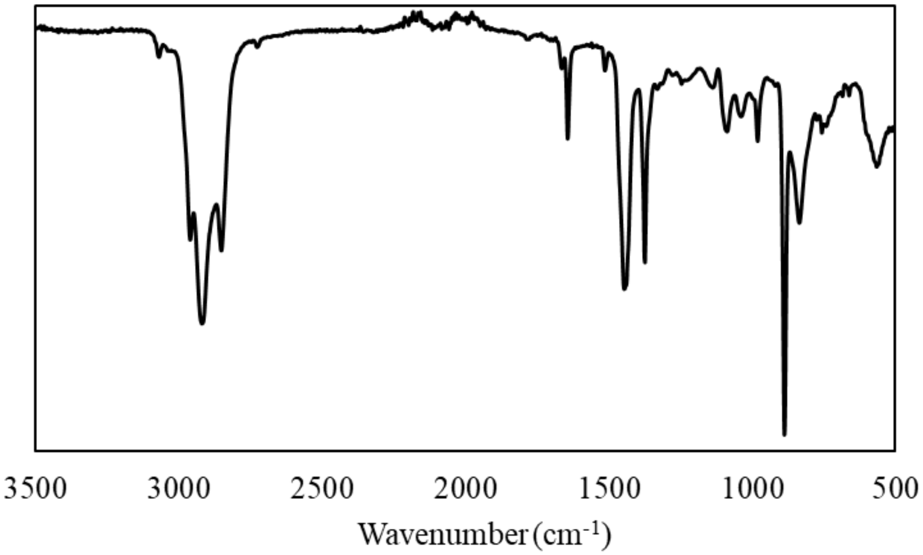

Fig. S270 FT-IR spectrum of PIP 500 equivalents generated by **$Tm(CH_2SiMe_3)_3(THF)_2$**, 2 equivalents $[Ph_3C][B(C_6F_5)_4]$, and 1 equivalent PPh_3 from **Table 5**, entry 16 (30 min).

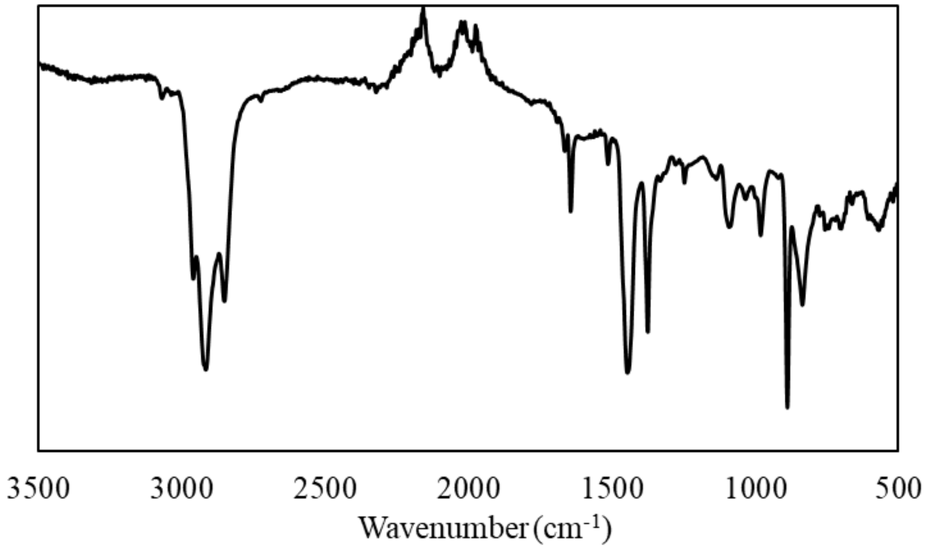

Fig. S271 FT-IR spectrum of PIP 500 equivalents generated by **$Sm(CH_2SiMe_3)_3(THF)_3$,** 1 equivalent PPh_3, and 2 equivalents $[Ph_3C][B(C_6F_5)_4]$ from **Table 6**, entry 1 ($[Ph_3C][B(C_6F_5)_4]$ addition time 0 min).

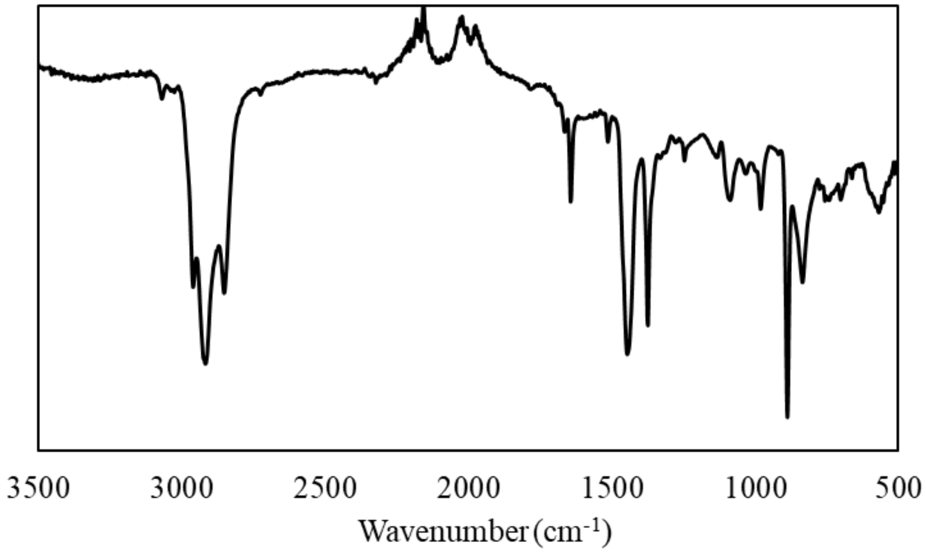

Fig. S272 FT-IR spectrum of PIP 500 equivalents generated by **$Sm(CH_2SiMe_3)_3(THF)_3$**, 1 equivalent PPh_3, and 2 equivalents $[Ph_3C][B(C_6F_5)_4]$ from **Table 6**, entry 2 ($[Ph_3C][B(C_6F_5)_4]$ addition time 10 min).

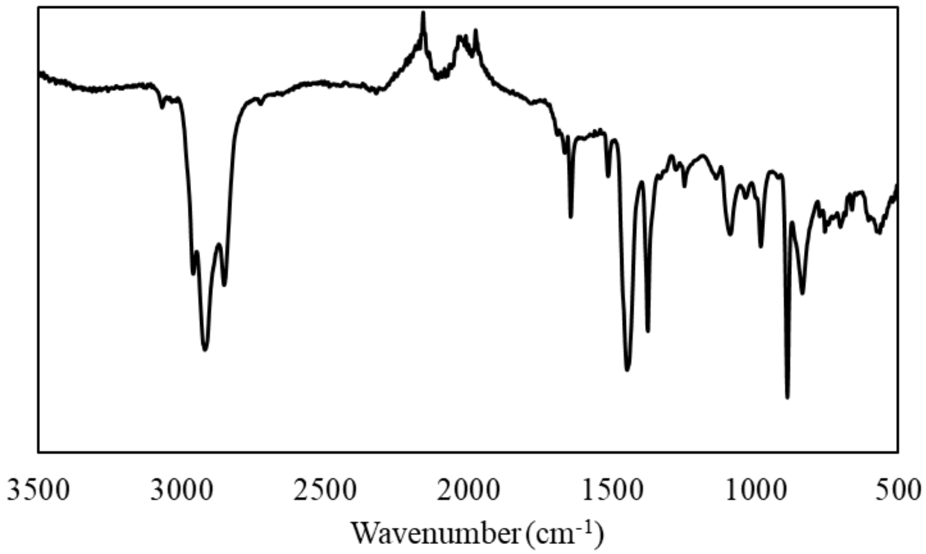

Fig. S273 FT-IR spectrum of PIP 500 equivalents generated by **$Sm(CH_2SiMe_3)_3(THF)_3$**, 1 equivalent PPh_3, and 2 equivalents $[Ph_3C][B(C_6F_5)_4]$ from **Table 6**, entry 3 ($[Ph_3C][B(C_6F_5)_4]$ addition time 30 min).

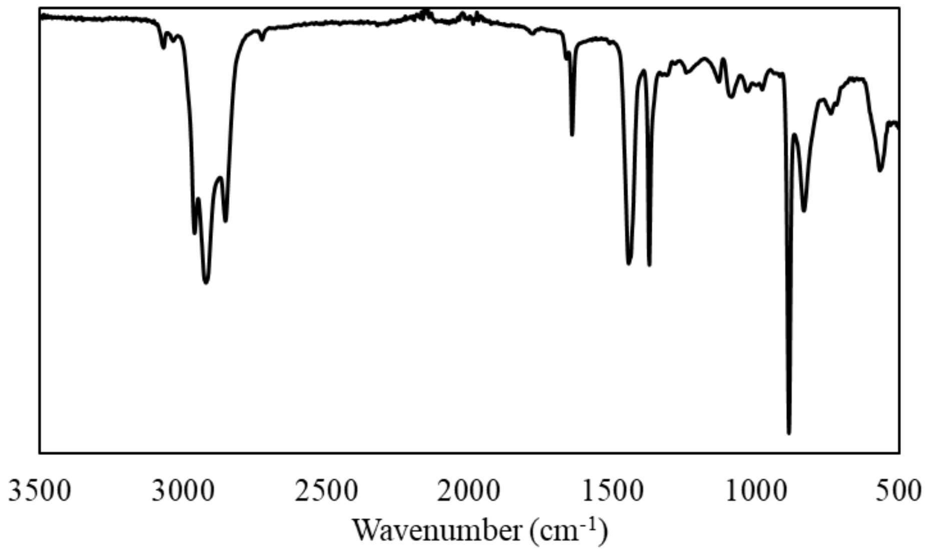

Fig. S274 FT-IR spectrum of PIP 500 equivalents generated by **$Gd(CH_2SiMe_3)_3(THF)_2$**, 1 equivalent PPh_3, and 2 equivalents $[Ph_3C][B(C_6F_5)_4]$ from **Table 6**, entry 4 ($[Ph_3C][B(C_6F_5)_4]$ addition time 0 min).

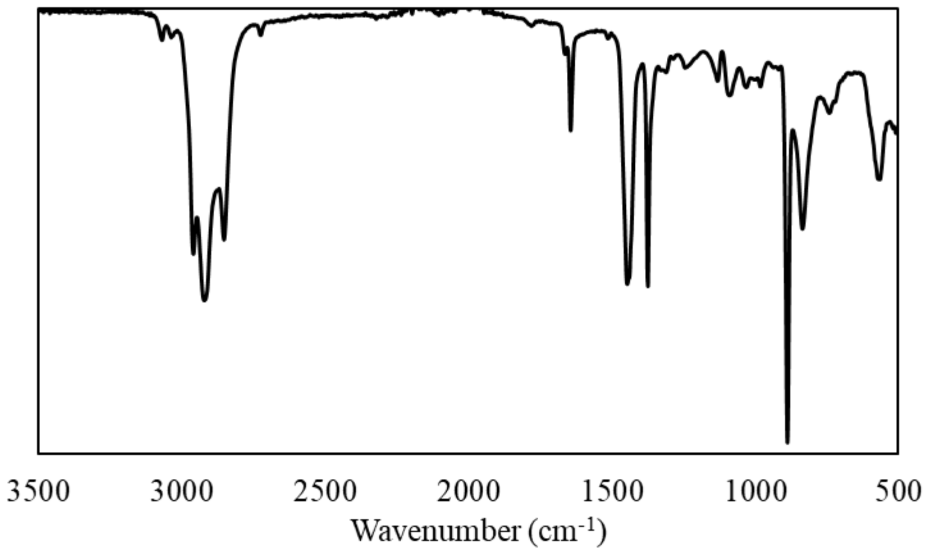

Fig. S275 FT-IR spectrum of PIP 500 equivalents generated by **$Gd(CH_2SiMe_3)_3(THF)_2$**, 1 equivalent PPh_3, and 2 equivalents $[Ph_3C][B(C_6F_5)_4]$ from **Table 6**, entry 5 ($[Ph_3C][B(C_6F_5)_4]$ addition time 10 min).

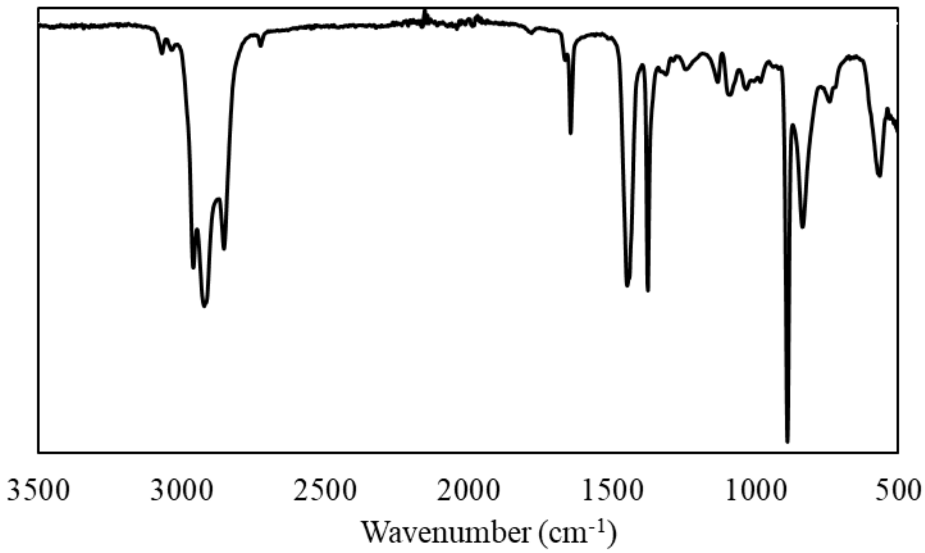

Fig. S276 FT-IR spectrum of PIP 500 equivalents generated by **$Gd(CH_2SiMe_3)_3(THF)_2$**, 1 equivalent PPh_3, and 2 equivalents $[Ph_3C][B(C_6F_5)_4]$ from **Table 6**, entry 6 ($[Ph_3C][B(C_6F_5)_4]$ addition time 30 min).

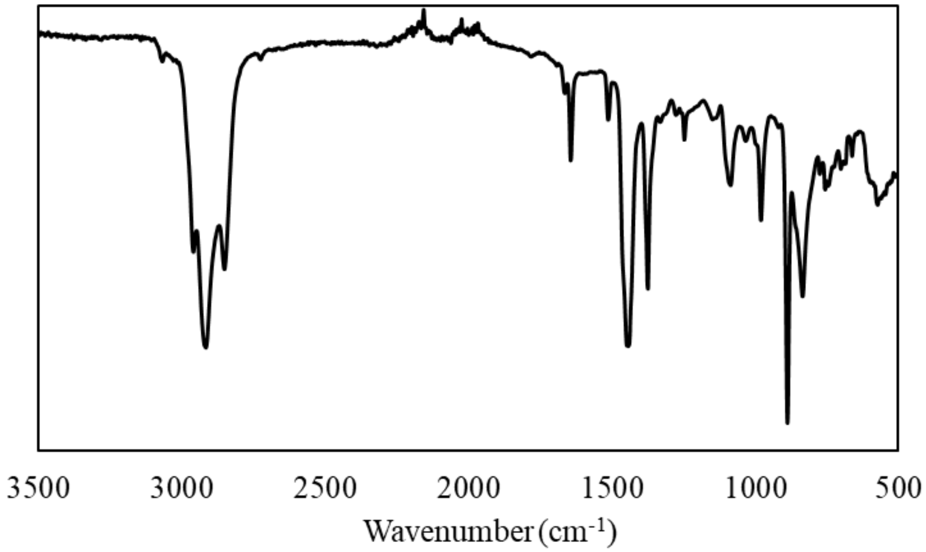

Fig. S277 FT-IR spectrum of PIP 500 equivalents generated by **$Y(CH_2SiMe_3)_3(THF)_2$**, 1 equivalent PPh_3, and 2 equivalents $[Ph_3C][B(C_6F_5)_4]$ from **Table 6**, entry 7 ($[Ph_3C][B(C_6F_5)_4]$ addition time 0 min).

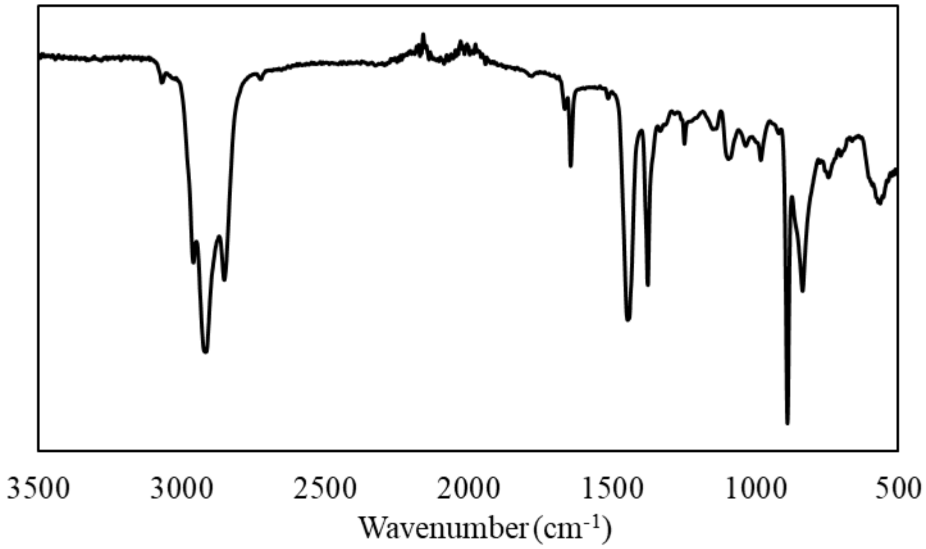

Fig. S278 FT-IR spectrum of PIP 500 equivalents generated by **$Y(CH_2SiMe_3)_3(THF)_2$**, 1 equivalent PPh_3, and 2 equivalents $[Ph_3C][B(C_6F_5)_4]$ from **Table 6**, entry 8 ($[Ph_3C][B(C_6F_5)_4]$ addition time 10 min).

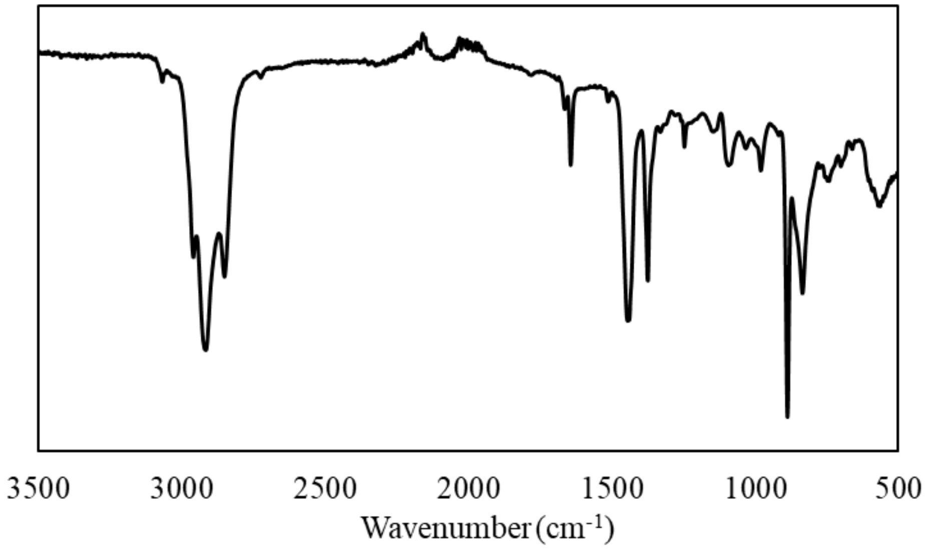

Fig. S279 FT-IR spectrum of PIP 500 equivalents generated by **$Y(CH_2SiMe_3)_3(THF)_2$**, 1 equivalent PPh_3, and 2 equivalents $[Ph_3C][B(C_6F_5)_4]$ from **Table 6**, entry 9 ($[Ph_3C][B(C_6F_5)_4]$ addition time 30 min).

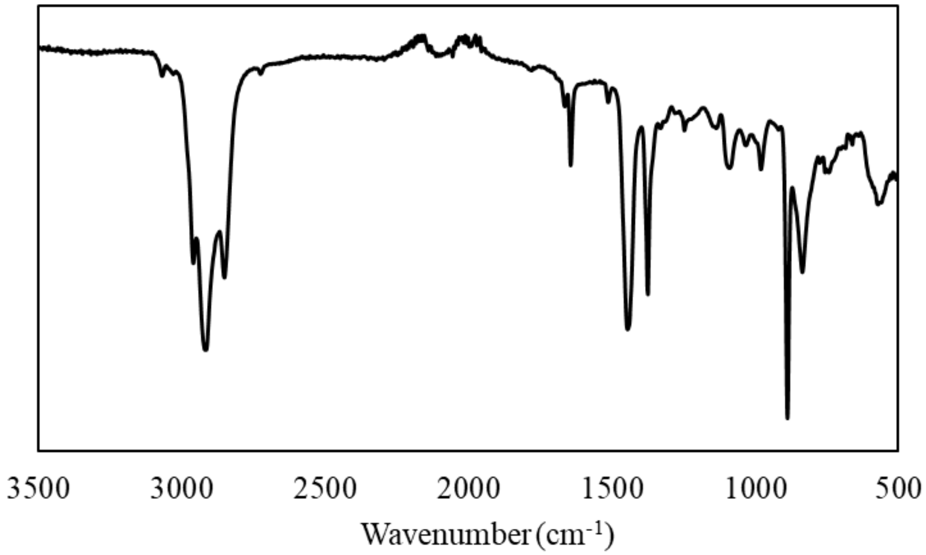

Fig. S280 FT-IR spectrum of PIP 500 equivalents generated by **$Tm(CH_2SiMe_3)_3(THF)_2$**, 1 equivalent PPh_3, and 2 equivalents $[Ph_3C][B(C_6F_5)_4]$ from **Table 6**, entry 10 ($[Ph_3C][B(C_6F_5)_4]$ addition time 0 min).

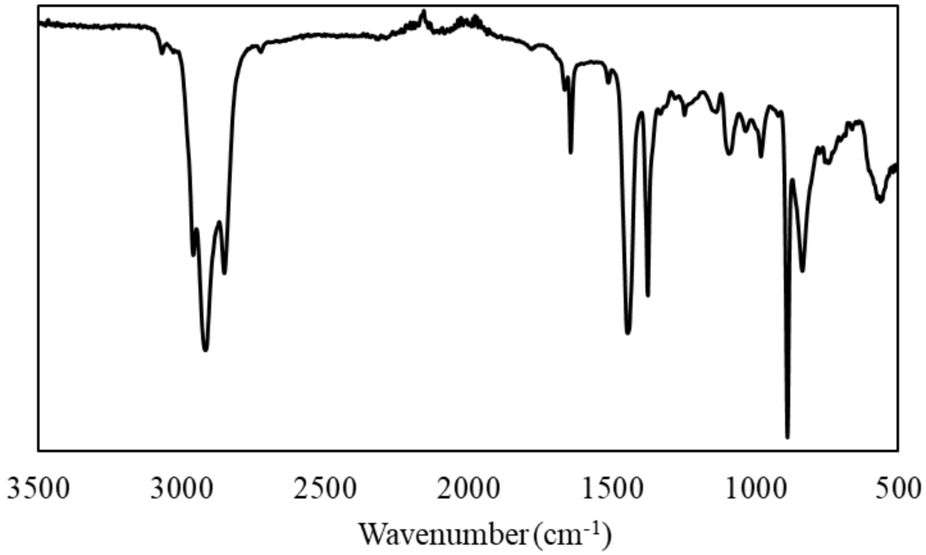

Fig. S281 FT-IR spectrum of PIP 500 equivalents generated by **Tm(CH$_2$SiMe$_3$)$_3$(THF)$_2$**, 1 equivalent PPh$_3$, and 2 equivalents [Ph$_3$C][B(C$_6$F$_5$)$_4$] from **Table 6**, entry 11 ([Ph$_3$C][B(C$_6$F$_5$)$_4$] addition time 10 min).

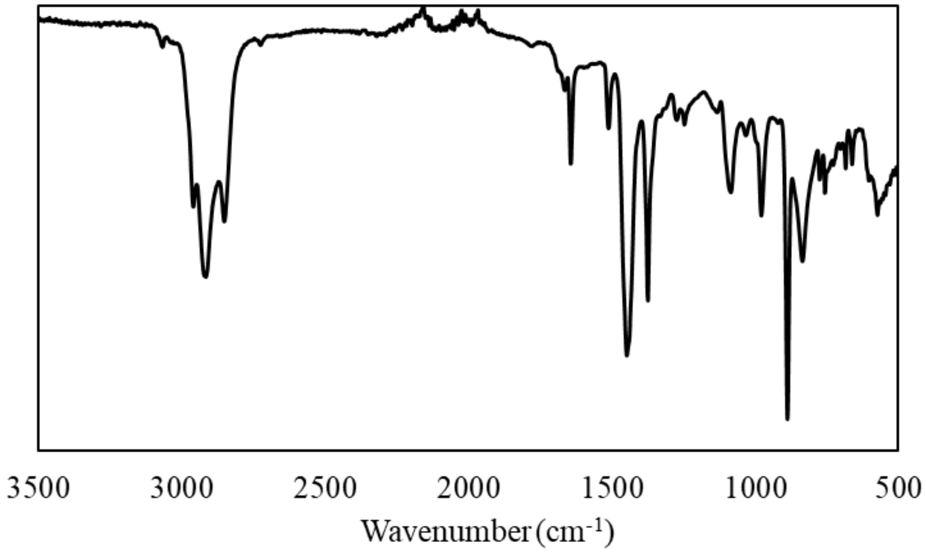

Fig. S282 FT-IR spectrum of PIP 500 equivalents generated by **Tm(CH$_2$SiMe$_3$)$_3$(THF)$_2$**, 1 equivalent PPh$_3$, and 2 equivalents [Ph$_3$C][B(C$_6$F$_5$)$_4$] from **Table 6**, entry 12 ([Ph$_3$C][B(C$_6$F$_5$)$_4$] addition time 30 min).

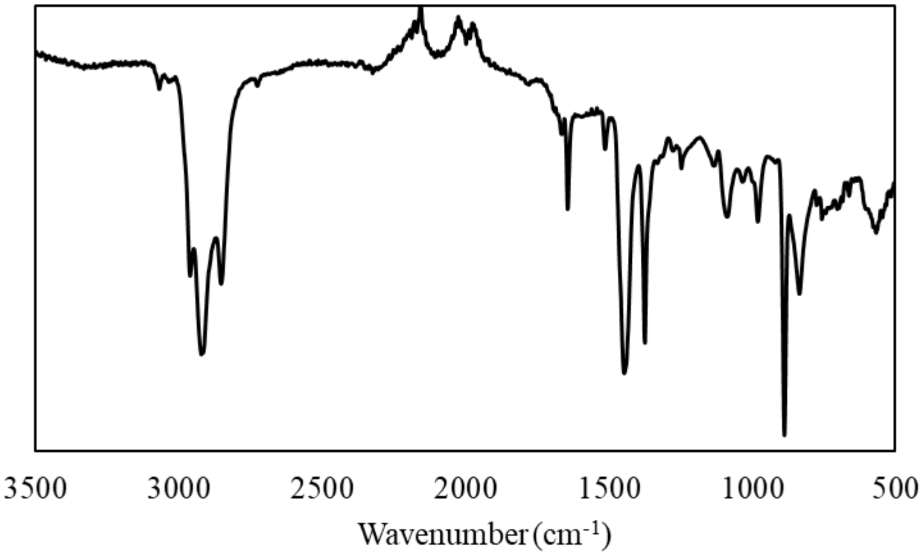

Fig. S283 FT-IR spectrum of PIP 500 equivalents generated by **$Sm(CH_2SiMe_3)_3(THF)_3$**, 2 equivalents $[Ph_3C][B(C_6F_5)_4]$, and 1 equivalent PPh_3 from **Table 7**, entry 1 (PPh_3 addition time 0 min).

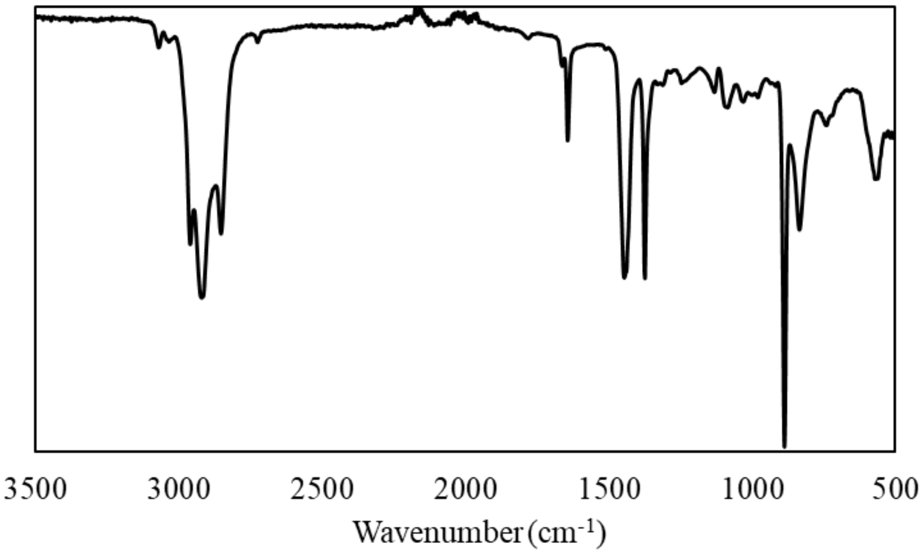

Fig. S284 FT-IR spectrum of PIP 500 equivalents generated by **$Sm(CH_2SiMe_3)_3(THF)_3$**, 2 equivalents $[Ph_3C][B(C_6F_5)_4]$, and 1 equivalent PPh_3 from **Table 7**, entry 2 (PPh_3 addition time 10 min).

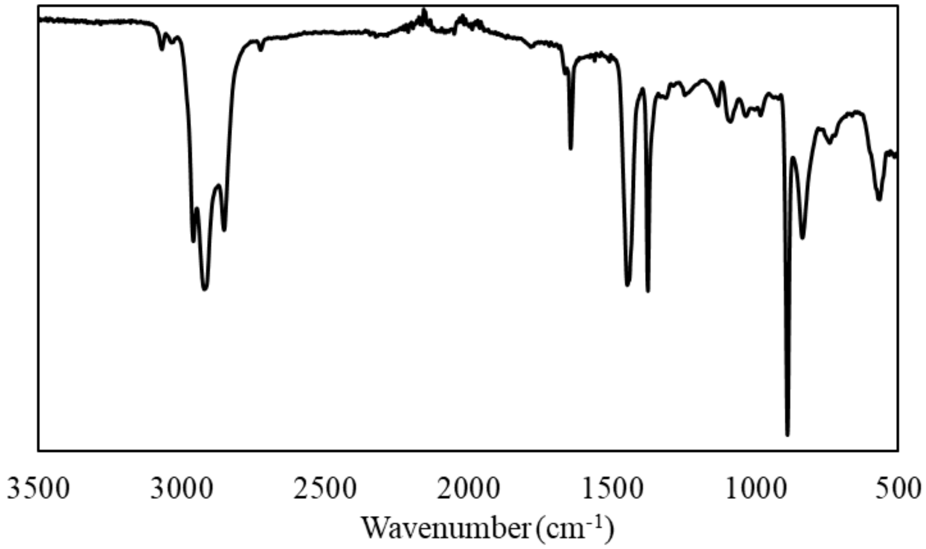

Fig. S285 FT-IR spectrum of PIP 500 equivalents generated by **$Sm(CH_2SiMe_3)_3(THF)_3$**, 2 equivalents $[Ph_3C][B(C_6F_5)_4]$, and 1 equivalent PPh_3 from **Table 7**, entry 3 (PPh_3 addition time 30 min).

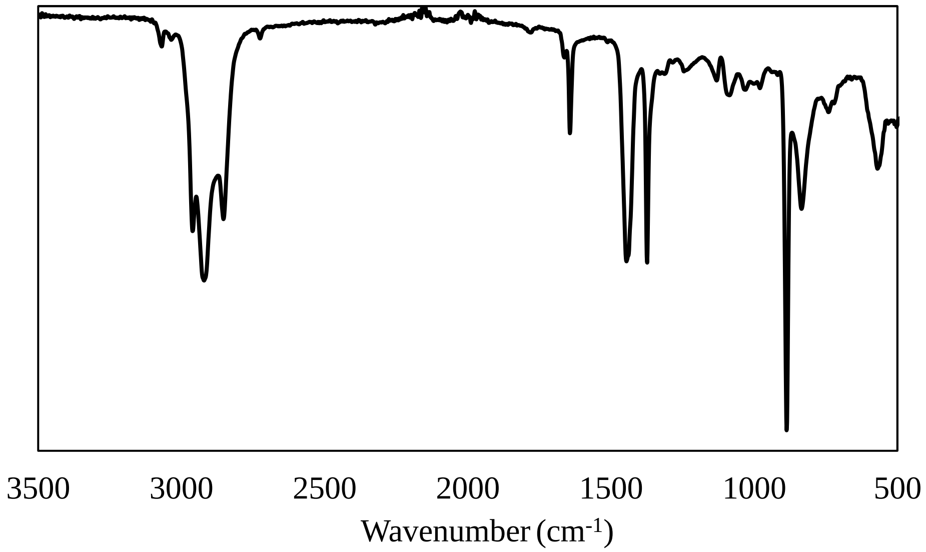

Fig. S286 FT-IR spectrum of PIP 500 equivalents generated by **$Gd(CH_2SiMe_3)_3(THF)_2$**, 2 equivalents $[Ph_3C][B(C_6F_5)_4]$, and 1 equivalent PPh_3 from **Table 7**, entry 4 (PPh_3 addition time 0 min).

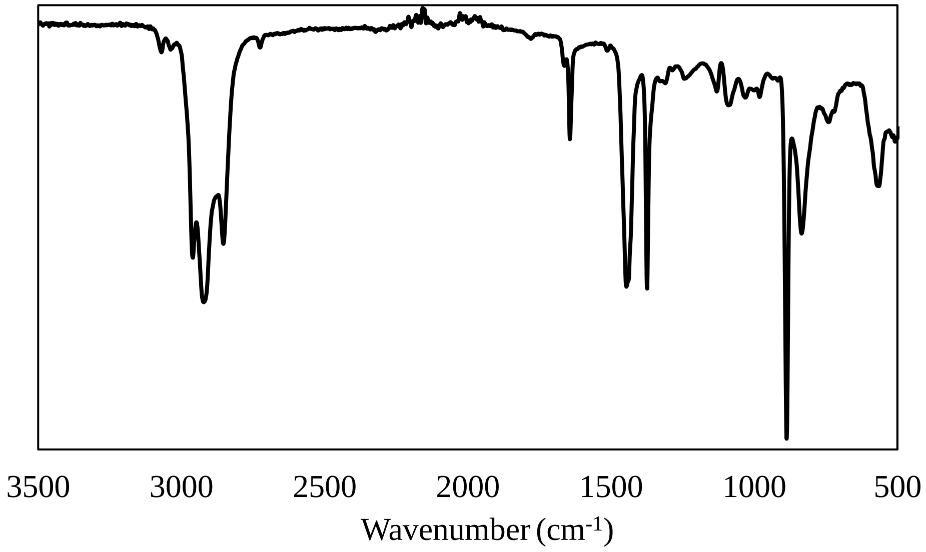

Fig. S287 FT-IR spectrum of PIP 500 equivalents generated by **$Gd(CH_2SiMe_3)_3(THF)_2$**, 2 equivalents $[Ph_3C][B(C_6F_5)_4]$, and 1 equivalent PPh_3 from **Table 7**, entry 5 (PPh_3 addition time 10 min).

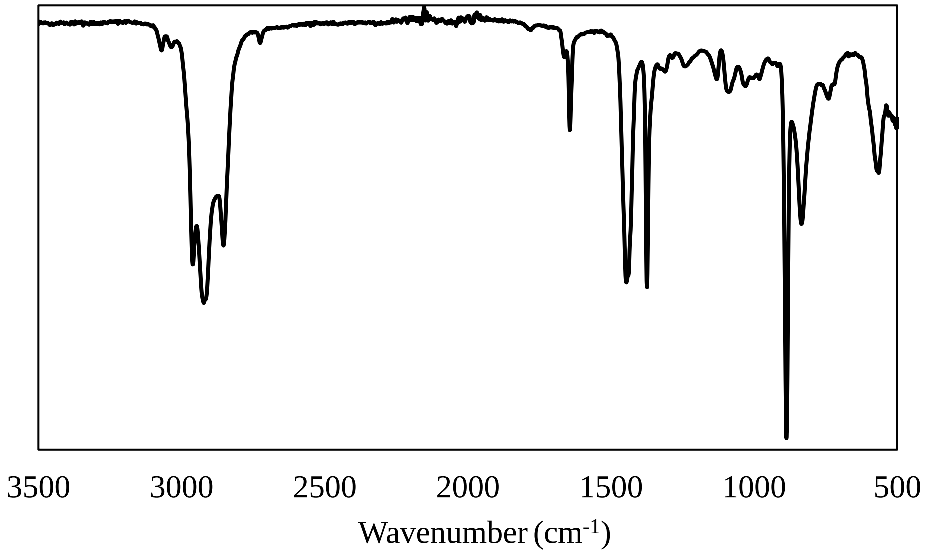

Fig. S288 FT-IR spectrum of PIP 500 equivalents generated by **$Gd(CH_2SiMe_3)_3(THF)_2$**, 2 equivalents $[Ph_3C][B(C_6F_5)_4]$, and 1 equivalent PPh_3 from **Table 7**, entry 6 (PPh_3 addition time 30 min).

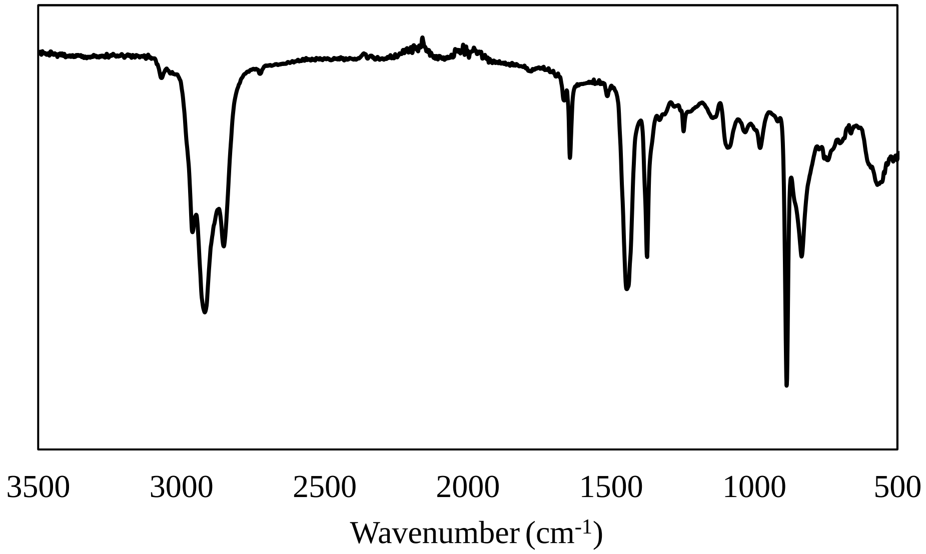

Fig. S289 FT-IR spectrum of PIP 500 equivalents generated by **$Y(CH_2SiMe_3)_3(THF)_2$**, 2 equivalents $[Ph_3C][B(C_6F_5)_4]$, and 1 equivalent PPh_3 from **Table 7**, entry 7 (PPh_3 addition time 0 min).

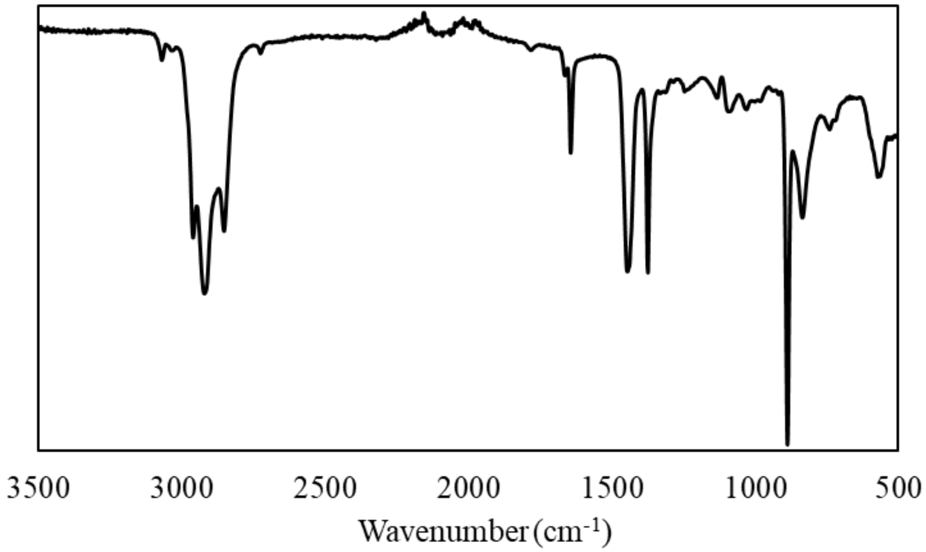

Fig. S290 FT-IR spectrum of PIP 500 equivalents generated by **$Y(CH_2SiMe_3)_3(THF)_2$**, 2 equivalents $[Ph_3C][B(C_6F_5)_4]$, and 1 equivalent PPh_3 from **Table 7**, entry 8 (PPh_3 addition time 10 min).

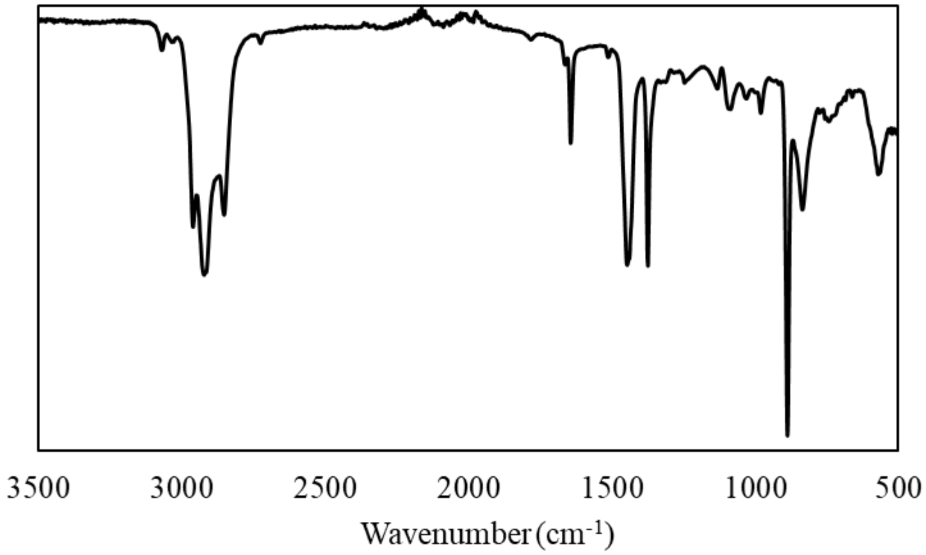

Fig. S291 FT-IR spectrum of PIP 500 equivalents generated by **$Y(CH_2SiMe_3)_3(THF)_2$**, 2 equivalents $[Ph_3C][B(C_6F_5)_4]$, and 1 equivalent PPh_3 from **Table 7**, entry 9 (PPh_3 addition time 30 min).

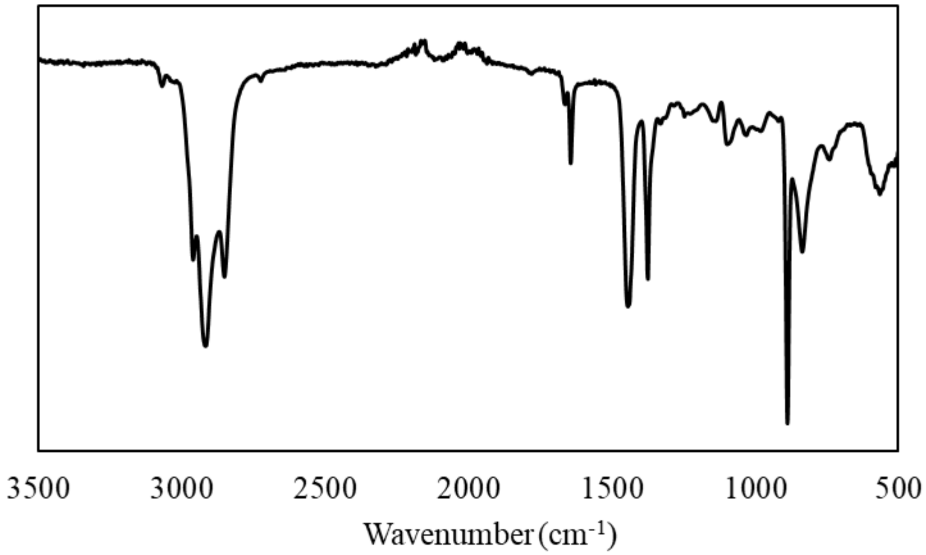

Fig. S292 FT-IR spectrum of PIP 500 equivalents generated by **$Tm(CH_2SiMe_3)_3(THF)_2$**, 2 equivalents $[Ph_3C][B(C_6F_5)_4]$, and 1 equivalent PPh_3 from **Table 7**, entry 10 (PPh_3 addition time 0 min).

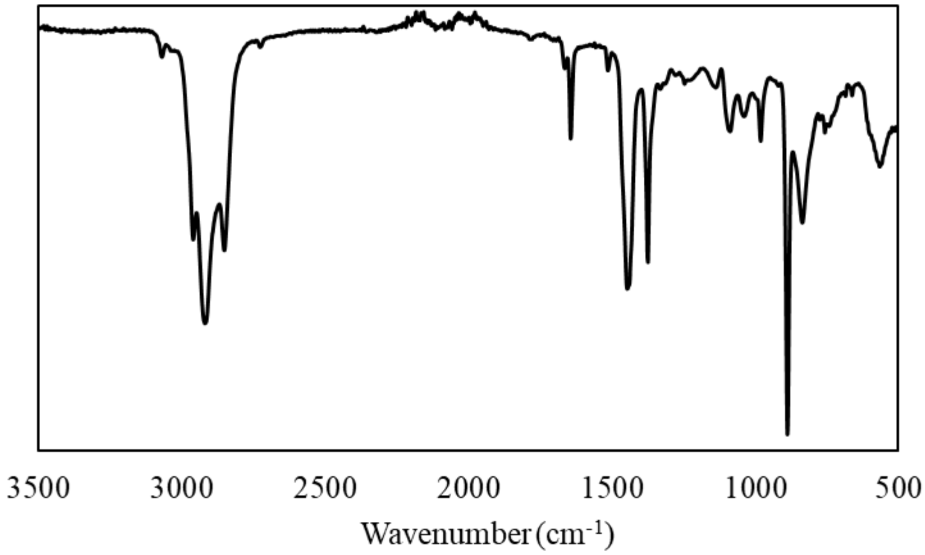

Fig. S293 FT-IR spectrum of PIP 500 equivalents generated by **$Tm(CH_2SiMe_3)_3(THF)_2$**, 2 equivalents $[Ph_3C][B(C_6F_5)_4]$, and 1 equivalent PPh_3 from **Table 7**, entry 11 (PPh_3 addition time 10 min).

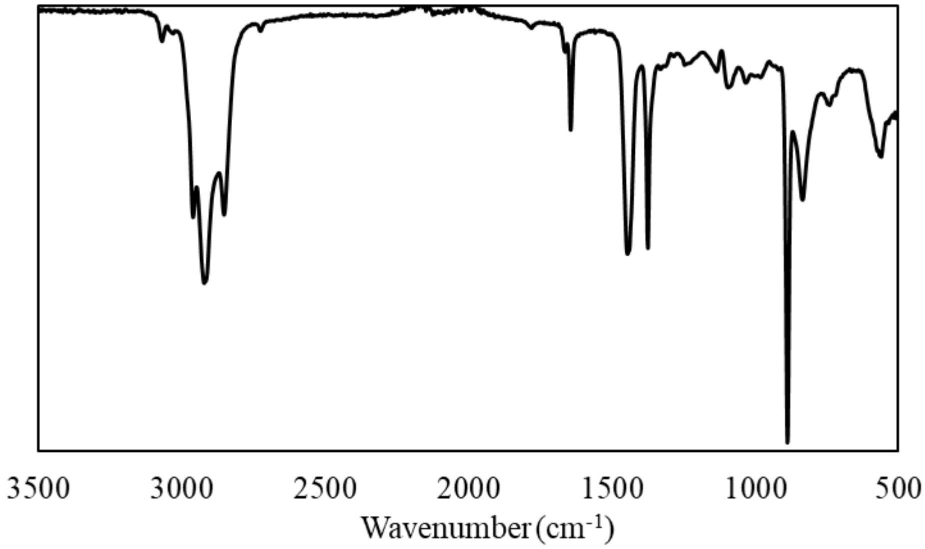

Fig. S294 FT-IR spectrum of PIP 500 equivalents generated by **$Tm(CH_2SiMe_3)_3(THF)_2$**, 2 equivalents $[Ph_3C][B(C_6F_5)_4]$, and 1 equivalent PPh_3 from **Table 7**, entry 12 (PPh_3 addition time 30 min).

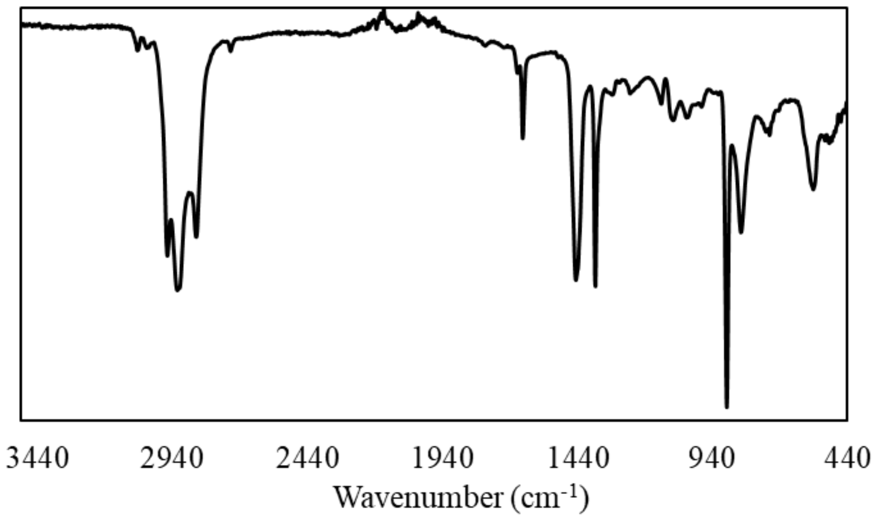

Fig. S295 FT-IR spectrum of PIP 500 equivalents generated by **$Y(CH_2SiMe_3)_3(THF)_2$** and 2 equivalents $[Ph_3C][B(C_6F_5)_4]$ from **Table 7**, entry 13 (IP addition time 10 min).

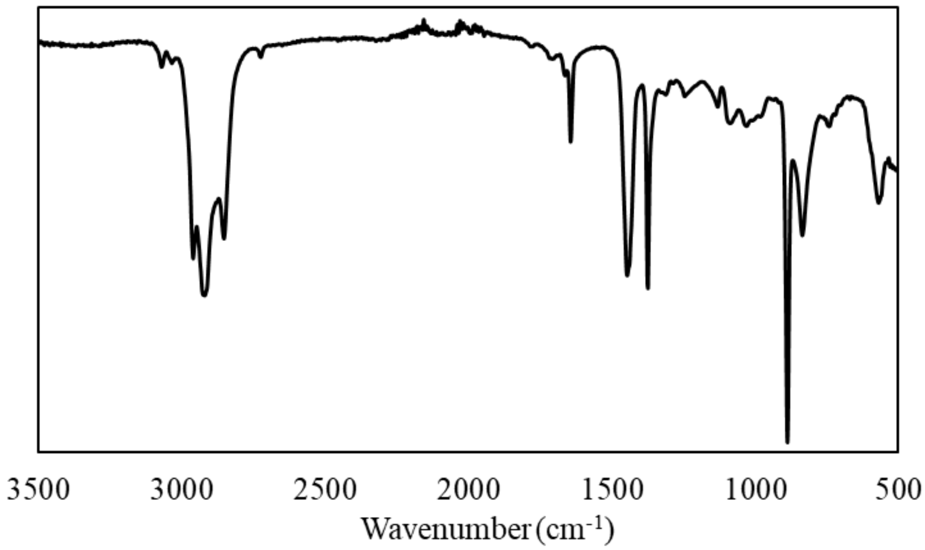

Fig. S296 FT-IR spectrum of PIP 500 equivalents generated by **$Y(CH_2SiMe_3)_3(THF)_2$** and 2 equivalents $[Ph_3C][B(C_6F_5)_4]$ from **Table 6**, entry 14 (IP addition time 20 min).

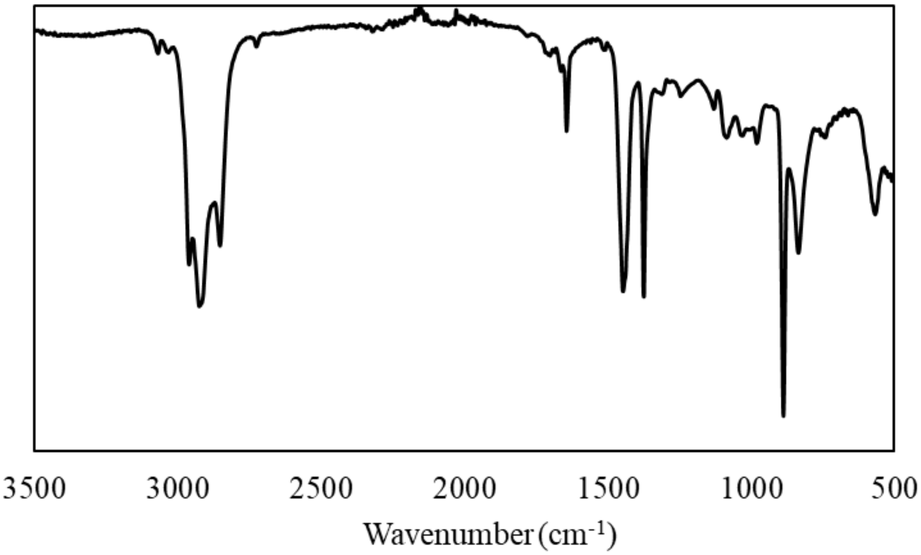

Fig. S297 FT-IR spectrum of PIP 500 equivalents generated by **$Y(CH_2SiMe_3)_3(THF)_2$** and 2 equivalents $[Ph_3C][B(C_6F_5)_4]$ from **Table 7**, entry 15 (IP addition time 40 min).

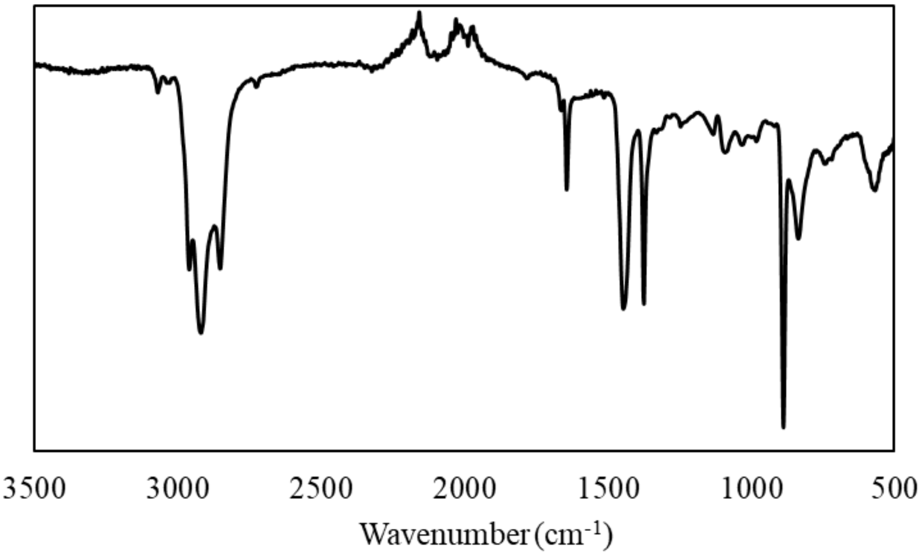

Fig. S298 FT-IR spectrum of PIP 500 equivalents generated by **$Y(CH_2SiMe_3)_3(THF)_2$**, 2 equivalents $[Ph_3C][B(C_6F_5)_4]$, and 1 equivalent PPh_3 from **Table 8**, entry 1 (Step 1: 60 min).

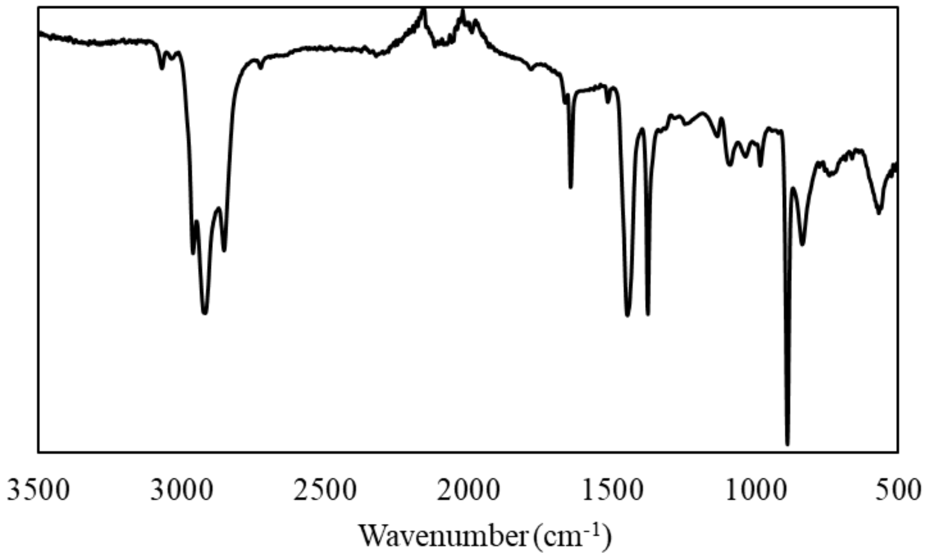

Fig. S299 FT-IR spectrum of PIP 250 equivalents generated by **$Y(CH_2SiMe_3)_3(THF)_2$**, 2 equivalents $[Ph_3C][B(C_6F_5)_4]$, and 1 equivalent PPh_3 from **Table 8**, entry 2 (Step 2: 60 min).

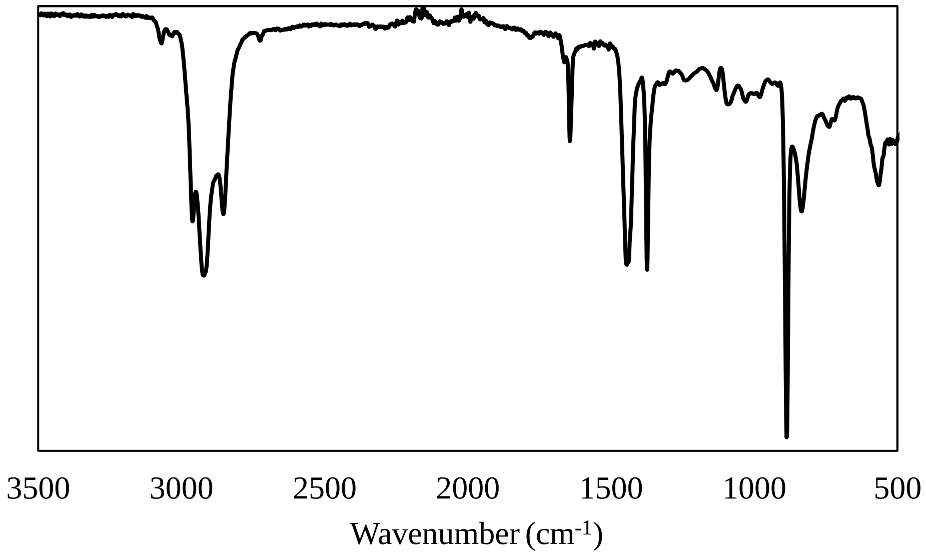

Fig. S300 FT-IR spectrum of PIP 125 equivalents generated by **$Y(CH_2SiMe_3)_3(THF)_2$**, 2 equivalents $[Ph_3C][B(C_6F_5)_4]$, and 1 equivalent PPh_3 from **Table 8**, entry 3 (Step 3: 60 min).

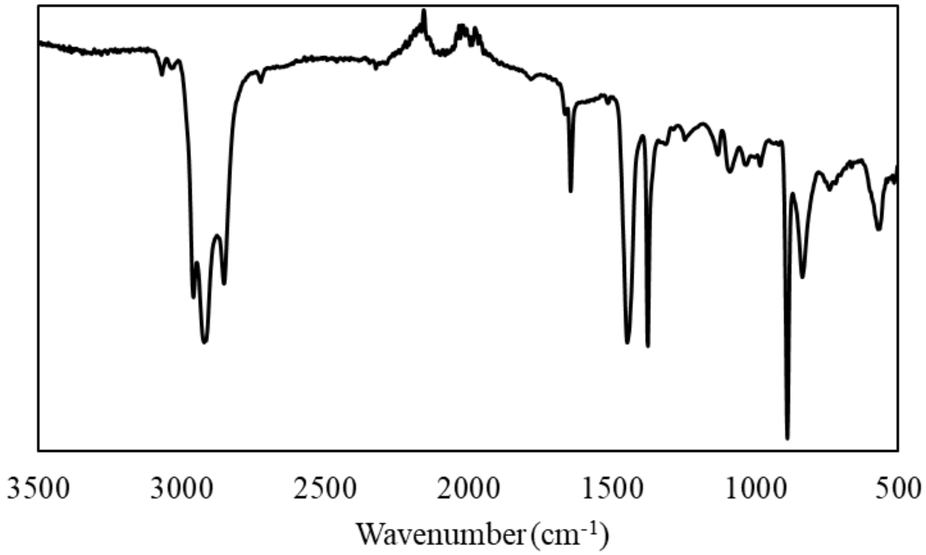

Fig. S301 FT-IR spectrum of PIP 500 equivalents generated by **Y(CH$_2$SiMe$_3$)$_3$(THF)$_2$** and 2 equivalents [Ph$_3$C][B(C$_6$F$_5$)$_4$] from **Table 8**, entry 4 (Step 1: 60 min).

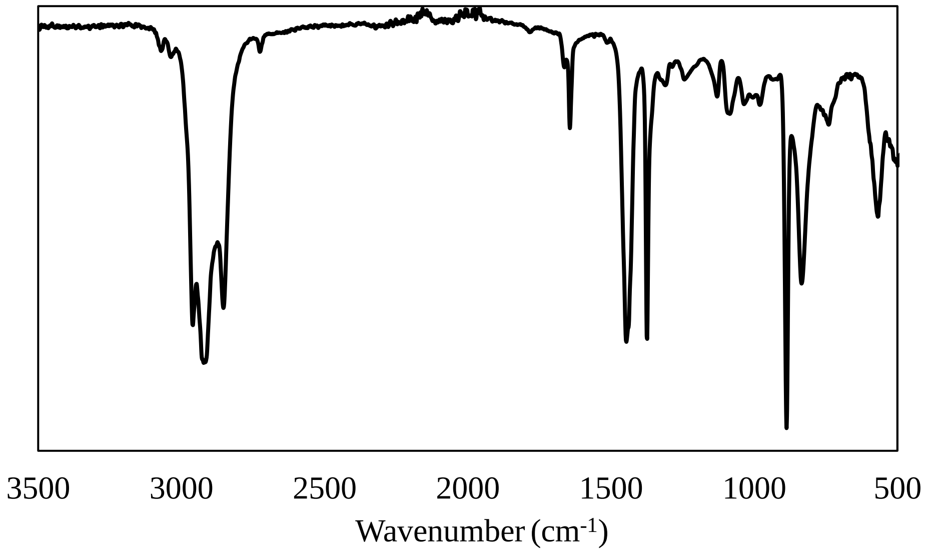

Fig. S302 FT-IR spectrum of PIP 250 equivalents generated by **Y(CH$_2$SiMe$_3$)$_3$(THF)$_2$** and 2 equivalents [Ph$_3$C][B(C$_6$F$_5$)$_4$] from **Table 8**, entry 5 (Step 2: 60 min).

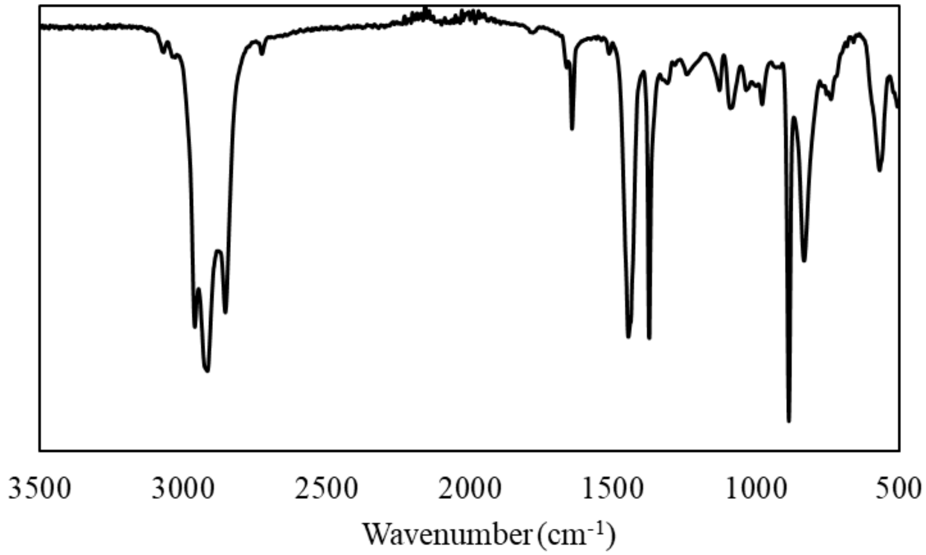

Fig. S303 FT-IR spectrum of PIP 125 equivalents generated by **$Y(CH_2SiMe_3)_3(THF)_2$** and 2 equivalents $[Ph_3C][B(C_6F_5)_4]$ from **Table 8**, entry 6 (Step 3: 60 min).
