# Supplementary material for: Enhanced Control of Isoprene Polymerization with Trialkyl Rare Earth Metal Complexes through Neutral Donor Support
Source: Inorg Chem. 2023 Dec 8;63(21):9464–77. doi: 10.1021/acs.inorgchem.3c03161 (PMC11134520; doi:10.1021/acs.inorgchem.3c03161)
Supplement: Supplementary file 4 — ic3c03161_si_004.pdf [file ic3c03161_si_004.pdf]

5.0 Gel Permeation Chromatography (GPC) Characterization of Isolated Polymers

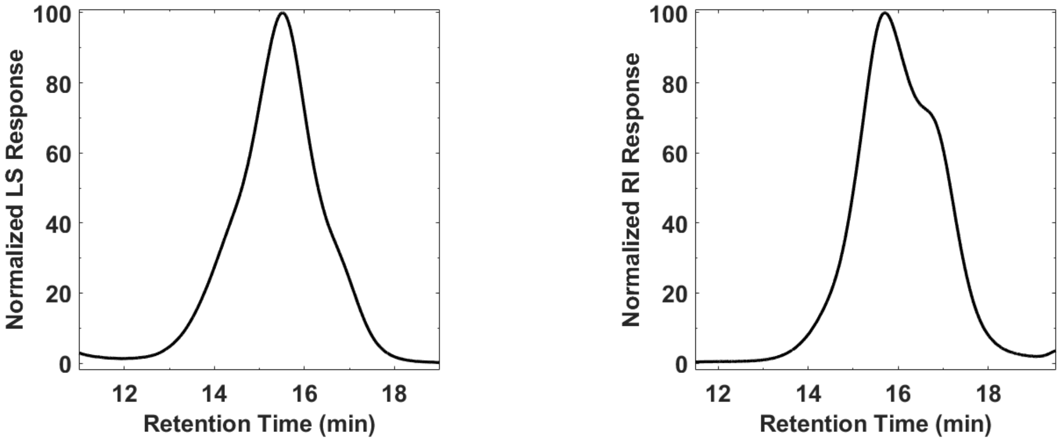

Fig. S304 GPC spectrum of PIP 500 equivalents generated by $\mathbf{Y(CH_2SiMe_3)_3(THF)_2}$ and 1 equivalent $[Ph_3C][B(C_6F_5)_4]$ from **Table 1**, entry 1 (30 min): (left) LS; (right) RI.

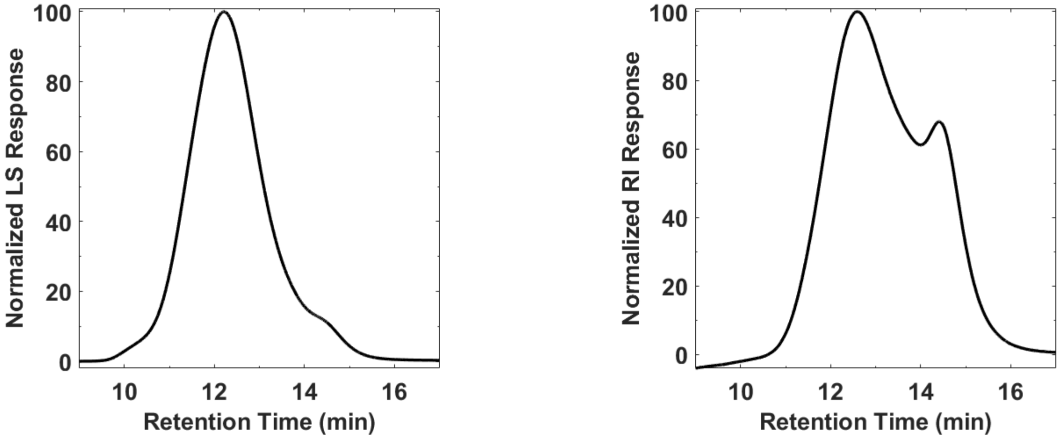

Fig. S305 GPC spectrum of PIP 500 equivalents generated by $\mathbf{Y(CH_2SiMe_3)_3(THF)_2}$ and 2 equivalents $[Ph_3C][B(C_6F_5)_4]$ from **Table 1**, entry 2 (30 min): (left) LS; (right) RI.

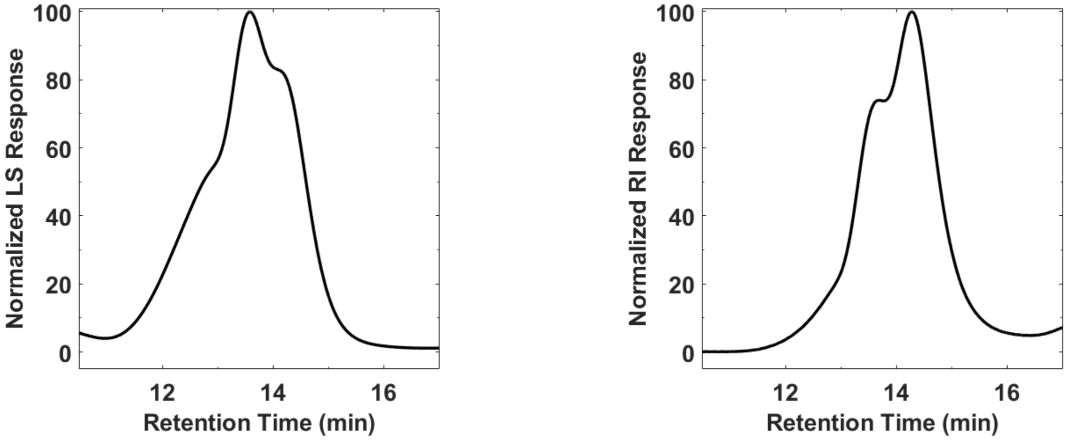

Fig. S306 GPC spectrum of PIP 500 equivalents generated by $\mathbf{Y(CH_2SiMe_3)_3(THF)_2}$, 2 equivalents $[Ph_3C][B(C_6F_5)_4]$, and 1 equivalent Bipy from **Table 2**, entry 3 (30 min): (left) LS; (right) RI.

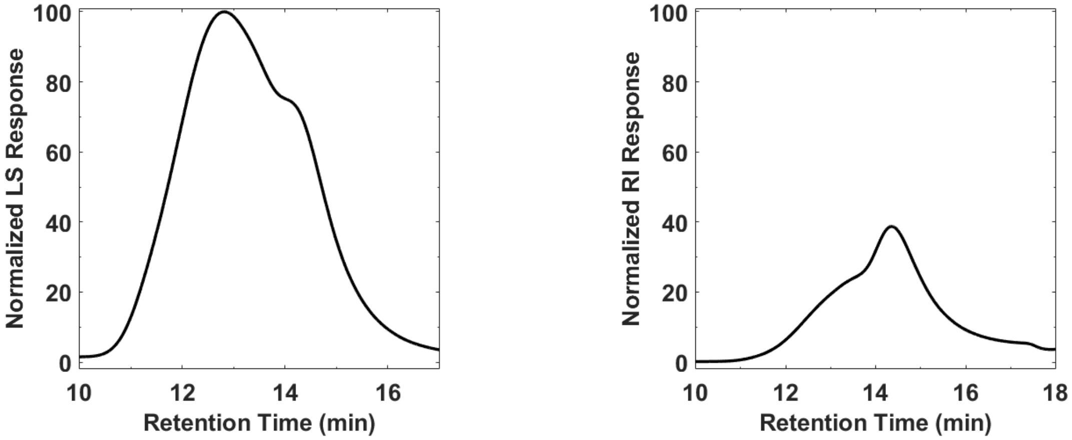

Fig. S307 GPC spectrum of PIP 500 equivalents generated by **$Y(CH_2SiMe_3)_3(THF)_2$**, 2 equivalents $[Ph_3C][B(C_6F_5)_4]$, and 1 equivalent MeCN from **Table 2**, entry 5 (30 min): (left) LS; (right) RI.

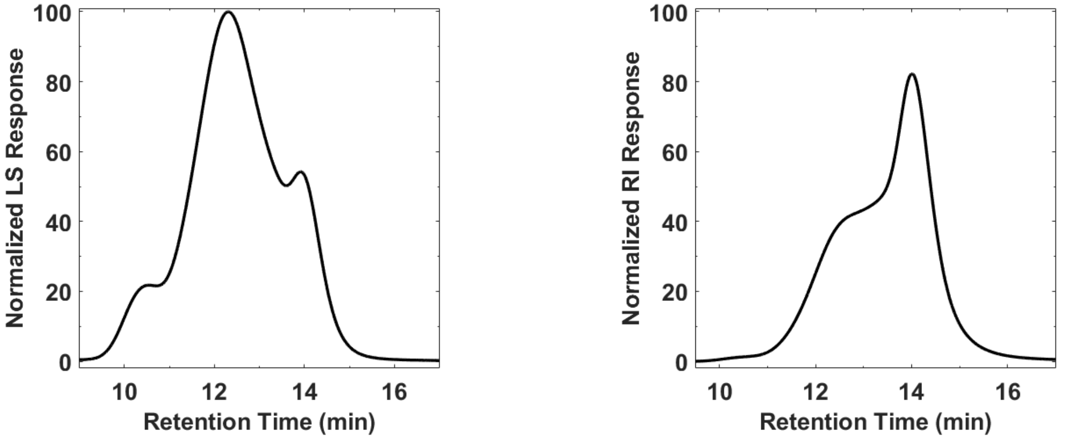

Fig. S308 GPC spectrum of PIP 500 equivalents generated by **$Y(CH_2SiMe_3)_3(THF)_2$**, 2 equivalents $[Ph_3C][B(C_6F_5)_4]$, and 1 equivalent P(*o*-tolyl)$_3$ from **Table 2**, entry 6 (30 min): (left) LS; (right) RI.

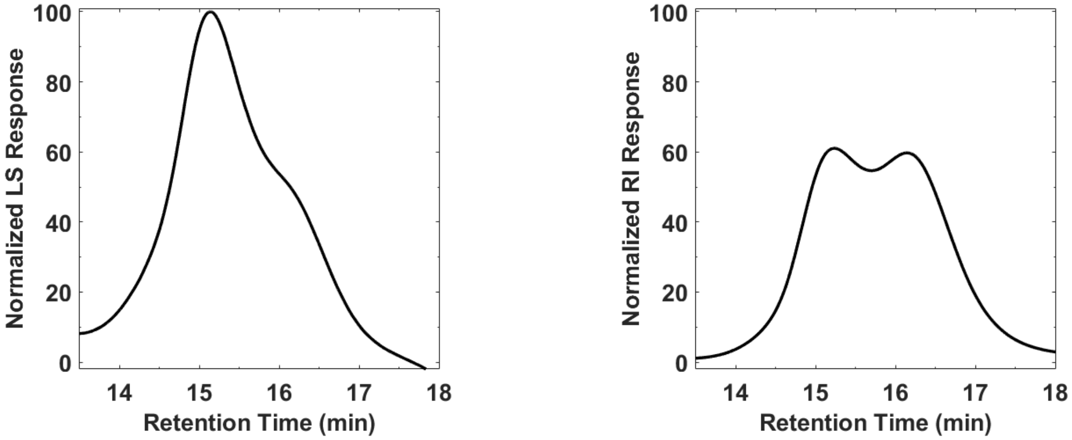

Fig. S309 GPC spectrum of PIP 500 equivalents generated by **$Y(CH_2SiMe_3)_3(THF)_2$**, 2 equivalents $[Ph_3C][B(C_6F_5)_4]$, and 1 equivalent PCy_3 from **Table 2**, entry 7 (30 min): (left) LS; (right) RI.

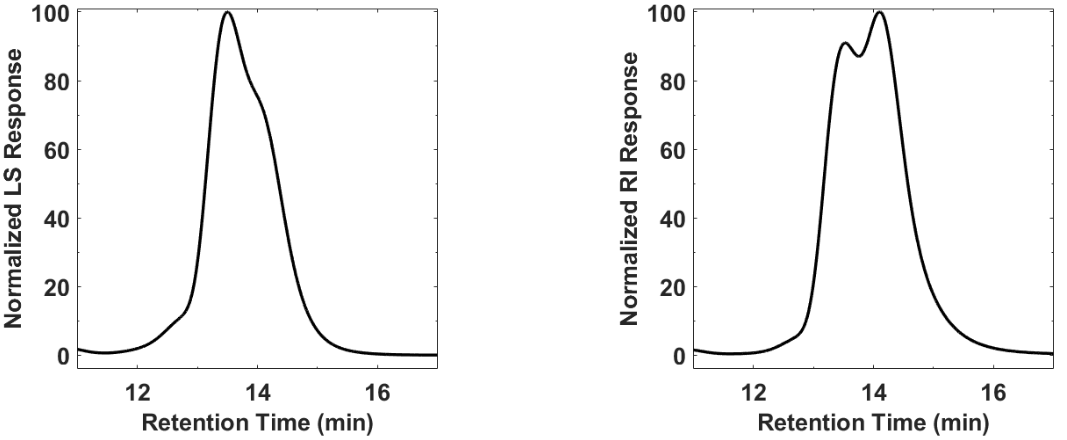

Fig. S310 GPC spectrum of PIP 500 equivalents generated by **$Y(CH_2SiMe_3)_3(THF)_2$**, 2 equivalents $[Ph_3C][B(C_6F_5)_4]$, and 1 equivalent PPh_3 from **Table 2**, entry 8 (30 min): (left) LS; (right) RI.

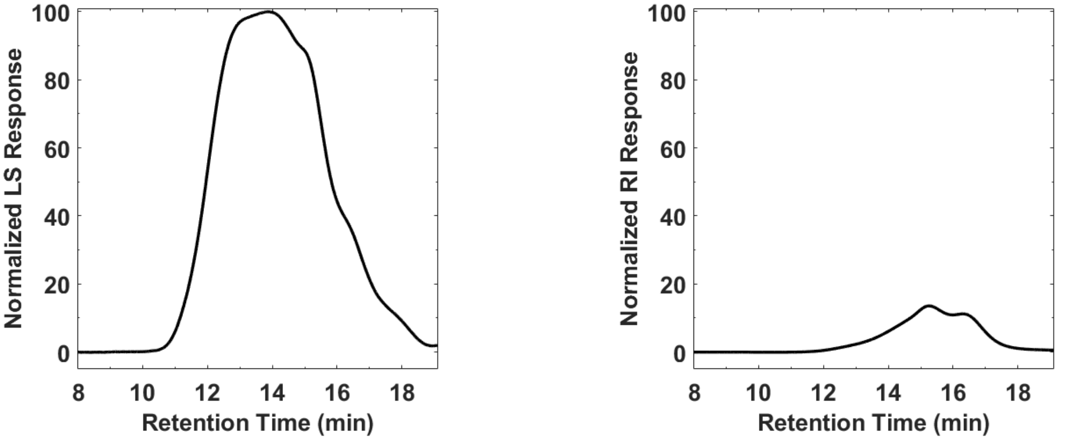

Fig. S311 GPC spectrum of PIP 500 equivalents generated by **$Y(CH_2SiMe_3)_3(THF)_2$**, 2 equivalents $[Ph_3C][B(C_6F_5)_4]$, and 1 equivalent P(Ph-*p*-OMe)$_3$ from **Table S1**, entry 1 (30 min): (left) LS; (right) RI.

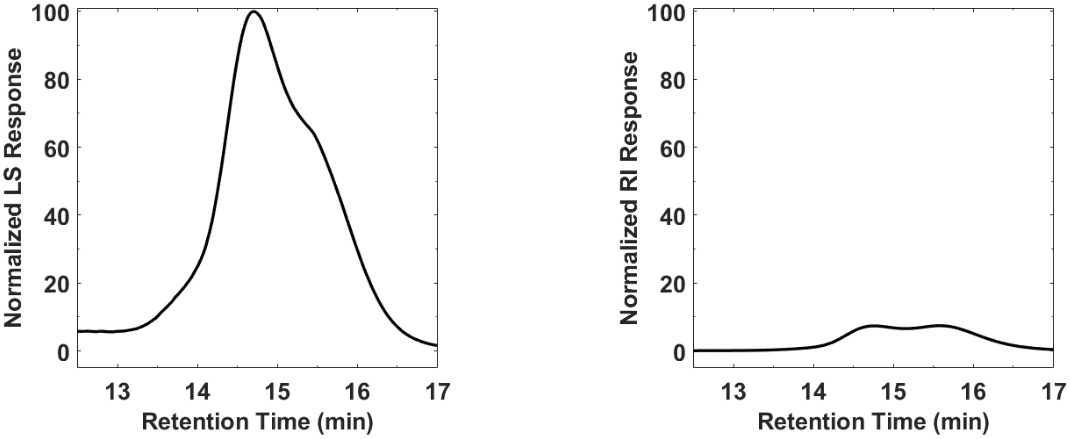

Fig. S312 GPC spectrum of PIP 500 equivalents generated by **$Y(CH_2SiMe_3)_3(THF)_2$**, 2 equivalents $[Ph_3C][B(C_6F_5)_4]$, and 1 equivalent P(*p*-tolyl)$_3$ from **Table S1**, entry 2 (30 min): (left) LS; (right) RI.

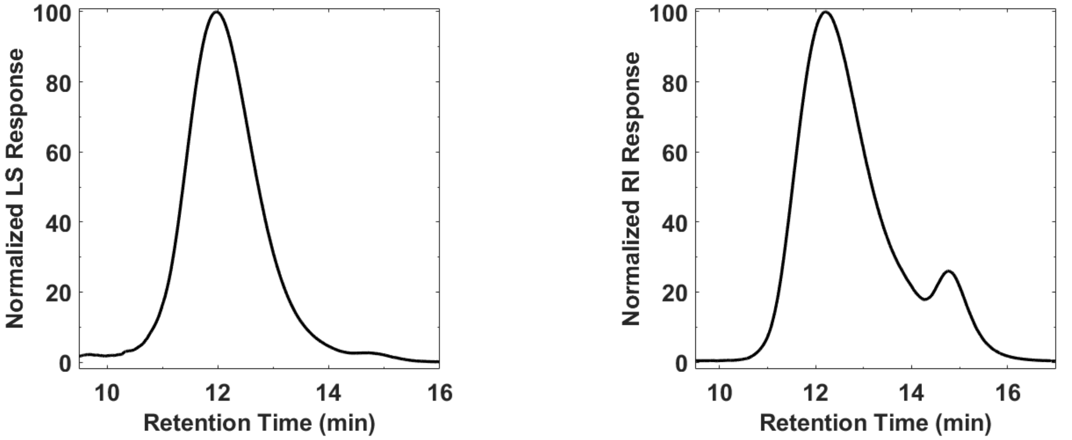

Fig. S313 GPC spectrum of PIP 500 equivalents generated by **$Y(CH_2SiMe_3)_3(THF)_2$**, 2 equivalents $[Ph_3C][B(C_6F_5)_4]$, and 1 equivalent P(Ph-*p*-F)$_3$ from **Table S1**, entry 4 (30 min): (left) LS; (right) RI.

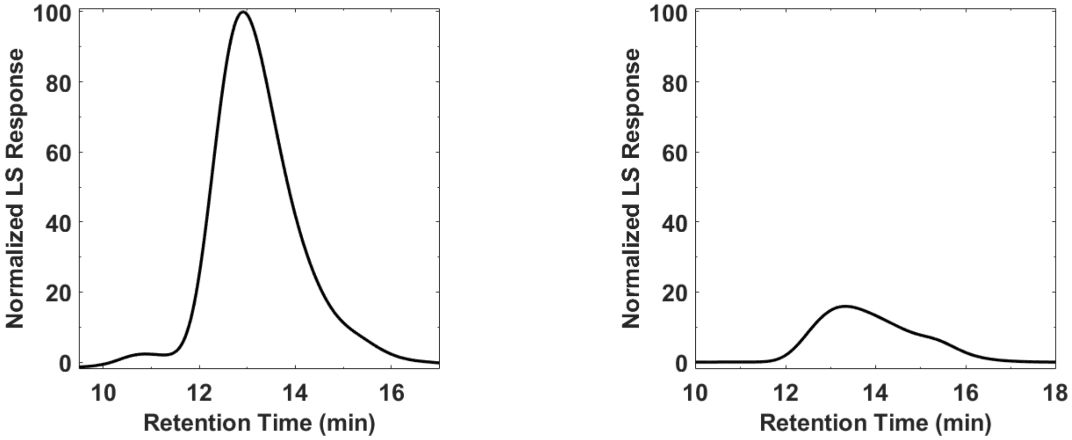

Fig. S314 GPC spectrum of PIP 500 equivalents generated by **$Y(CH_2SiMe_3)_3(THF)_2$** and 2 equivalents $[Ph_3C][B(C_6F_5)_4]$ from **Table S2**, entry 1 (5 min): (left) LS; (right) RI.

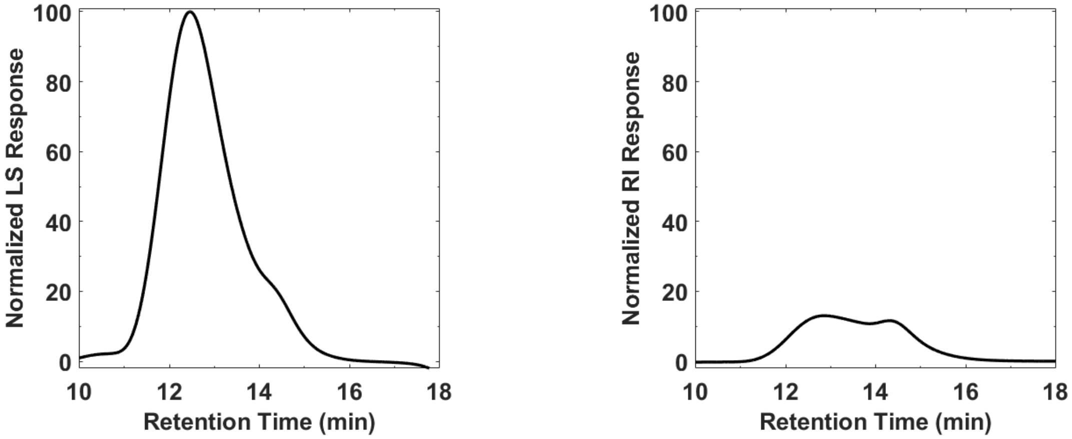

Fig. S315 GPC spectrum of PIP 500 equivalents generated by **$Y(CH_2SiMe_3)_3(THF)_2$** and 2 equivalents $[Ph_3C][B(C_6F_5)_4]$ from **Table S2**, entry 2 (12 min): (left) LS; (right) RI.

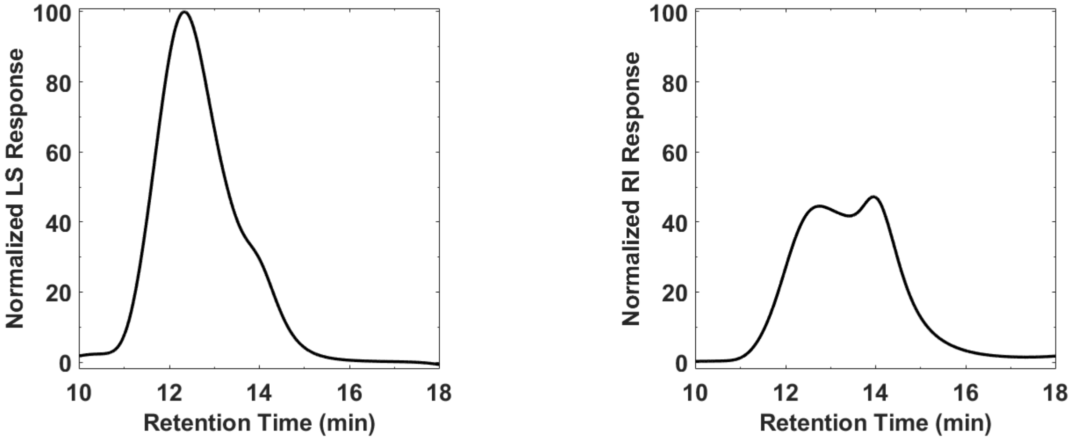

Fig. S316 GPC spectrum of PIP 500 equivalents generated by **$Y(CH_2SiMe_3)_3(THF)_2$** and 2 equivalents $[Ph_3C][B(C_6F_5)_4]$ from **Table S2**, entry 3 (18 min): (left) LS; (right) RI.

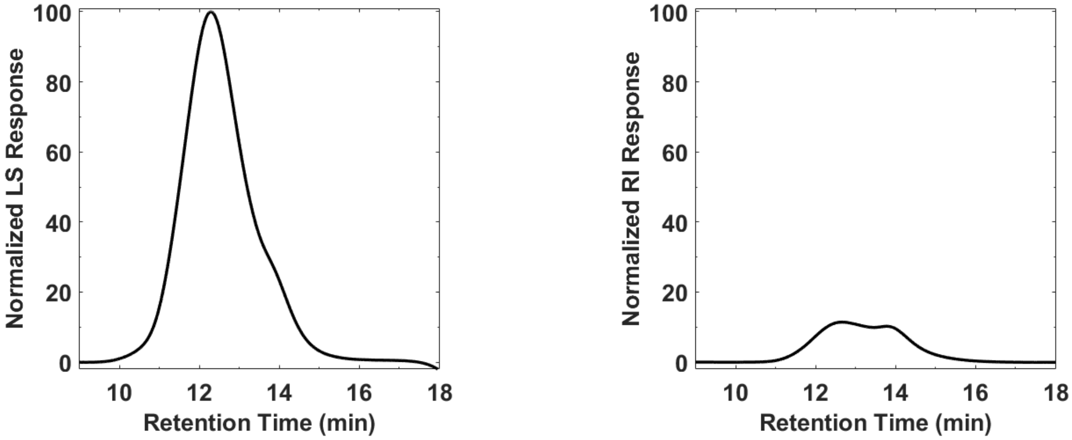

Fig. S317 GPC spectrum of PIP 500 equivalents generated by **$Y(CH_2SiMe_3)_3(THF)_2$** and 2 equivalents $[Ph_3C][B(C_6F_5)_4]$ from **Table S2**, entry 4 (24 min): (left) LS; (right) RI.

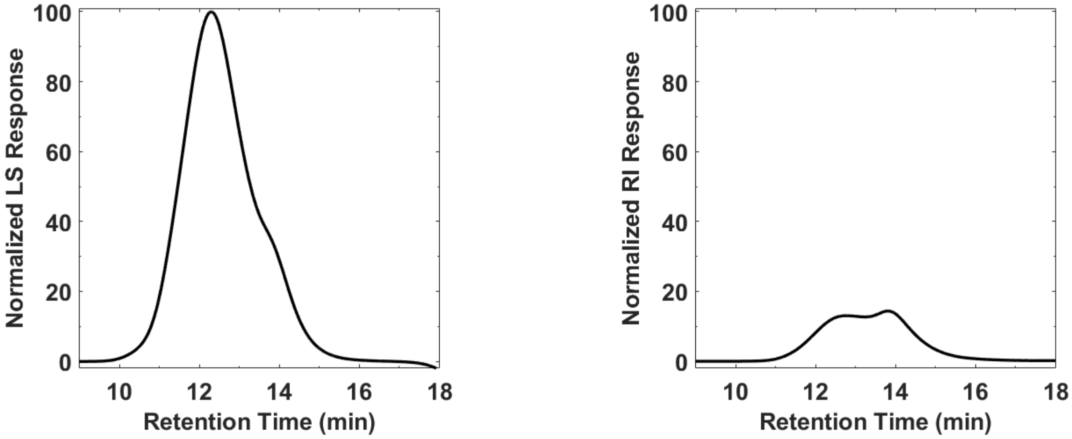

Fig. S318 GPC spectrum of PIP 500 equivalents generated by **$Y(CH_2SiMe_3)_3(THF)_2$** and 2 equivalents $[Ph_3C][B(C_6F_5)_4]$ from **Table S2**, entry 5 (30 min): (left) LS; (right) RI.

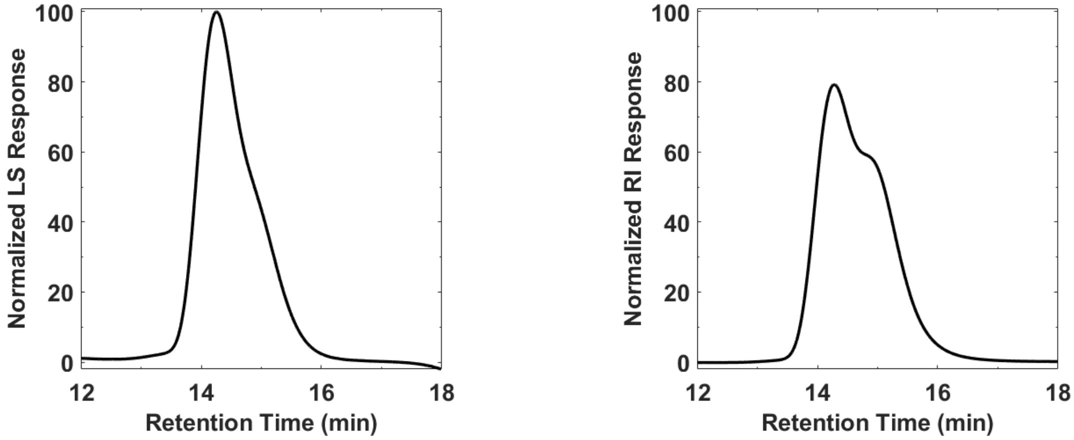

Fig. S319 GPC spectrum of PIP 500 equivalents generated by **$Y(CH_2SiMe_3)_3(THF)_2$**, 2 equivalents $[Ph_3C][B(C_6F_5)_4]$, and 1 equivalent PPh_3 from **Table S3**, entry 1 (10 min): (left) LS; (right) RI.

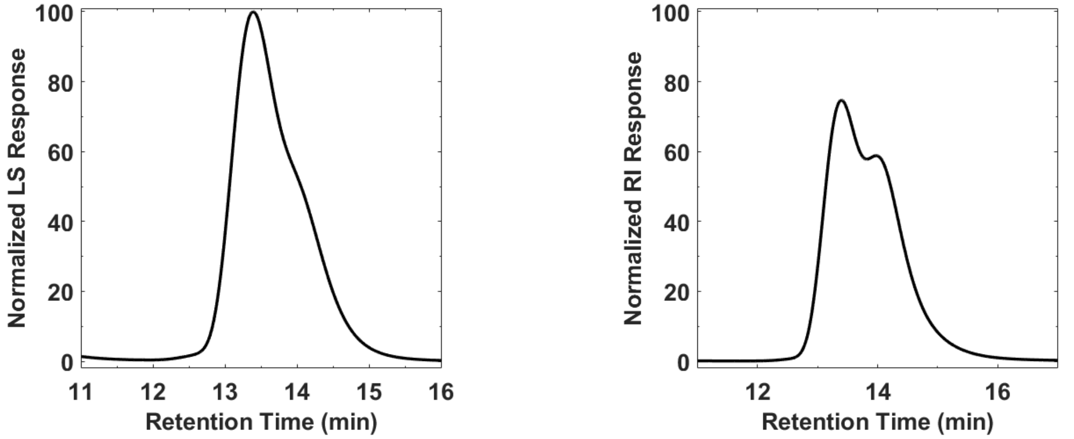

Fig. S320 GPC spectrum of PIP 500 equivalents generated by **$Y(CH_2SiMe_3)_3(THF)_2$**, 2 equivalents $[Ph_3C][B(C_6F_5)_4]$, and 1 equivalent PPh_3 from **Table S3**, entry 2 (21 min): (left) LS; (right) RI.

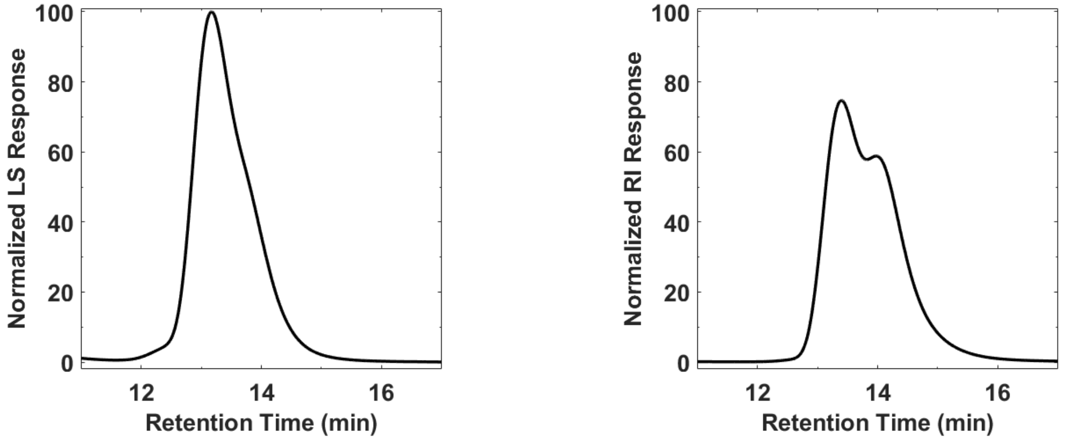

Fig. S321 GPC spectrum of PIP 500 equivalents generated by **$Y(CH_2SiMe_3)_3(THF)_2$**, 2 equivalents $[Ph_3C][B(C_6F_5)_4]$, and 1 equivalent PPh_3 from **Table S3**, entry 3 (31 min): (left) LS; (right) RI.

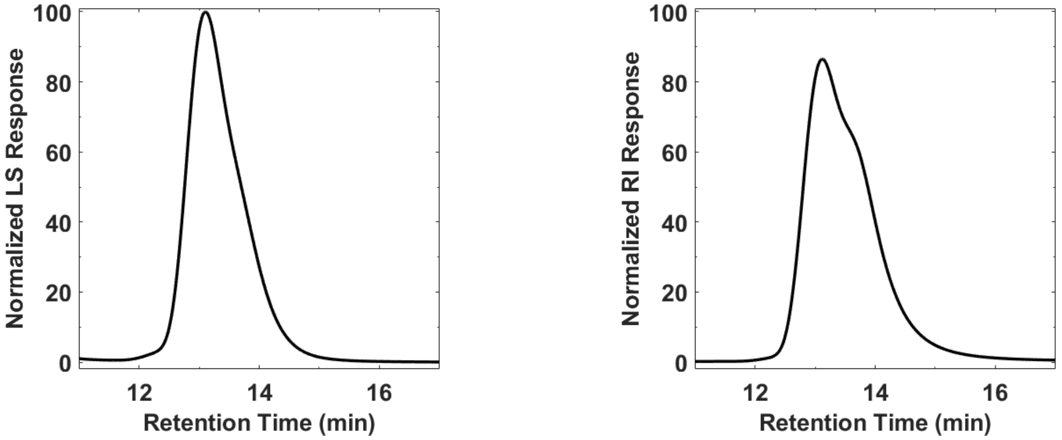

Fig. S322 GPC spectrum of PIP 500 equivalents generated by **Y(CH_2SiMe_3)$_3$(THF)$_2$**, 2 equivalents [Ph_3C][B(C_6F_5)$_4$], and 1 equivalent PPh_3 from **Table S3**, entry 4 (41 min): (left) LS; (right) RI.

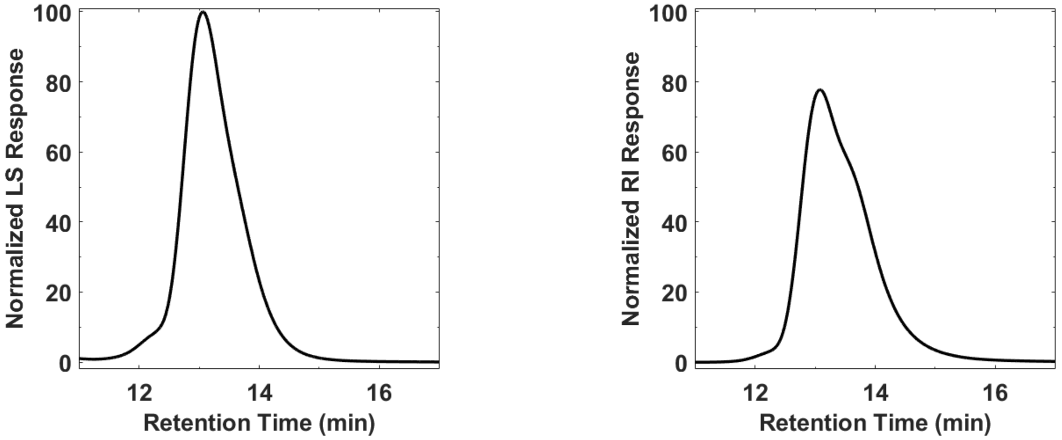

Fig. S323 GPC spectrum of PIP 500 equivalents generated by **Y(CH_2SiMe_3)$_3$(THF)$_2$**, 2 equivalents [Ph_3C][B(C_6F_5)$_4$], and 1 equivalent PPh_3 from **Table S3**, entry 5 (51 min): (left) LS; (right) RI.

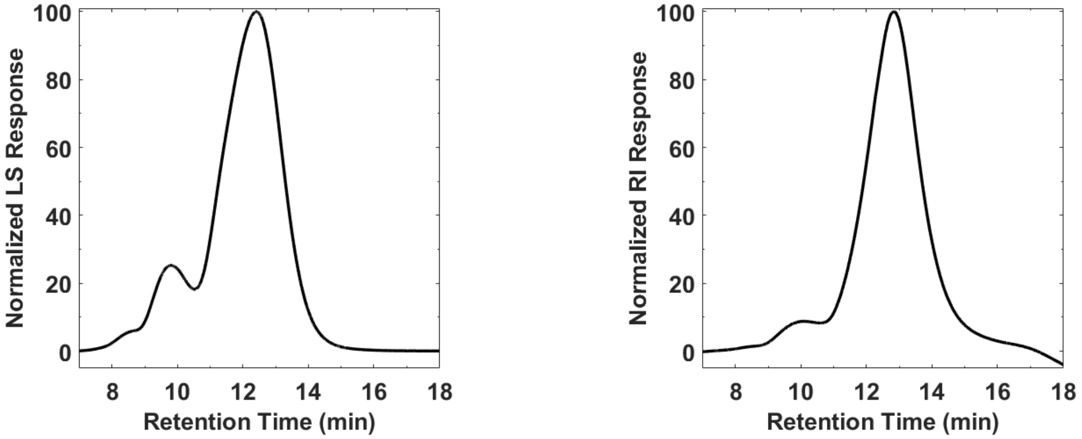

Fig. S324 GPC spectrum of PIP 500 equivalents generated by **Sm(CH_2SiMe_3)$_3$(THF)$_3$** and 1 equivalent [Ph_3C][B(C_6F_5)$_4$] from **Table S4**, entry 1 (7 h): (left) LS; (right) RI.

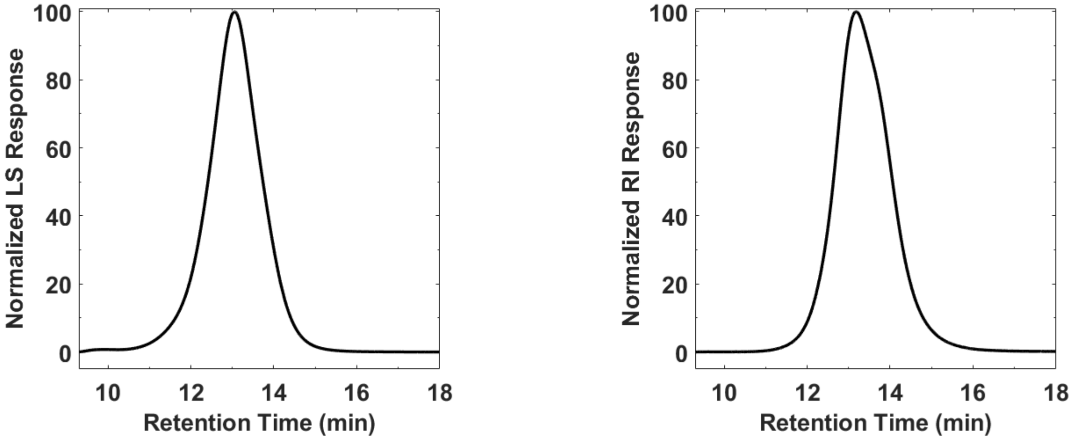

Fig. S325 GPC spectrum of PIP 500 equivalents generated by **Gd(CH_2SiMe_3)$_3$(THF)$_2$** and 1 equivalent [Ph_3C][$B(C_6F_5)_4$] from **Table S4**, entry 2 (7 h): (left) LS; (right) RI.

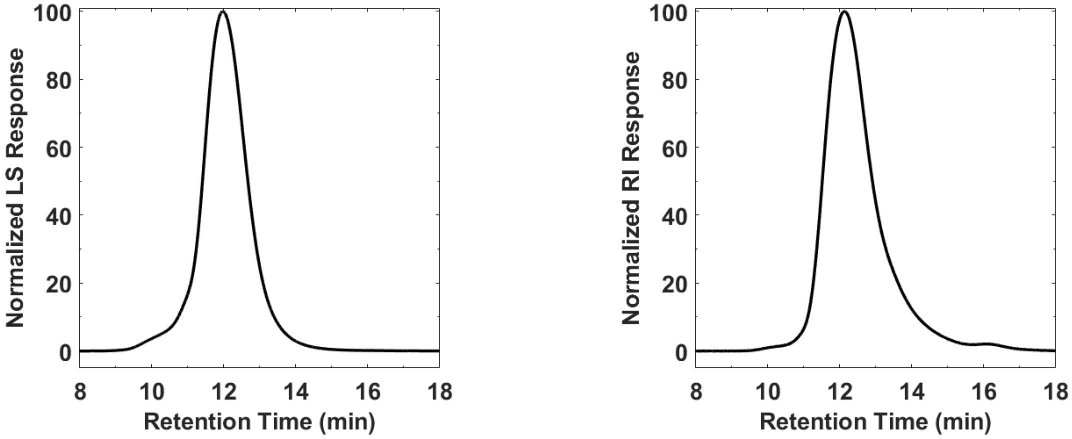

Fig. S326 GPC spectrum of PIP 500 equivalents generated by **Gd(CH_2SiMe_3)$_3$(THF)$_2$** and 2 equivalents [Ph_3C][$B(C_6F_5)_4$] from **Table S4**, entry 3 (7 h): (left) LS; (right) RI.

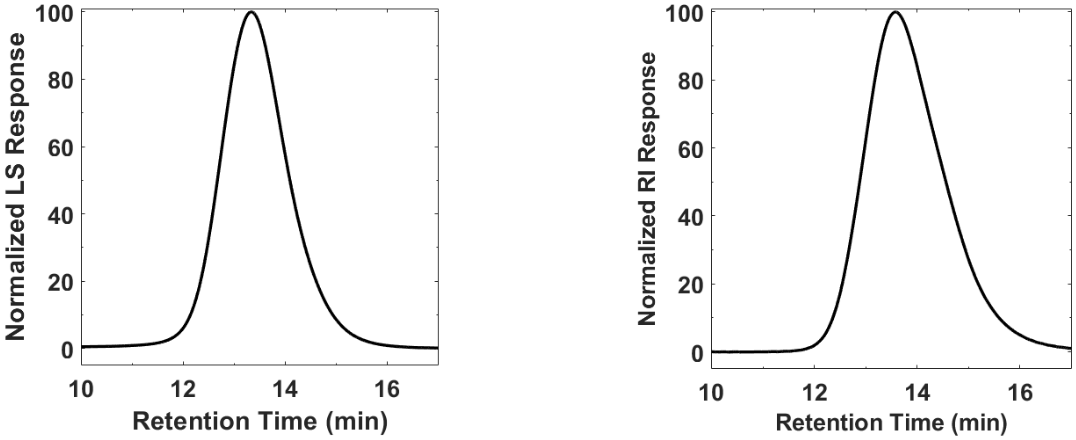

Fig. S327 GPC spectrum of PIP 500 equivalents generated by **Tm(CH_2SiMe_3)$_3$(THF)$_2$** and 1 equivalent [Ph_3C][$B(C_6F_5)_4$] from **Table S4**, entry 4 (7 h): (left) LS; (right) RI.

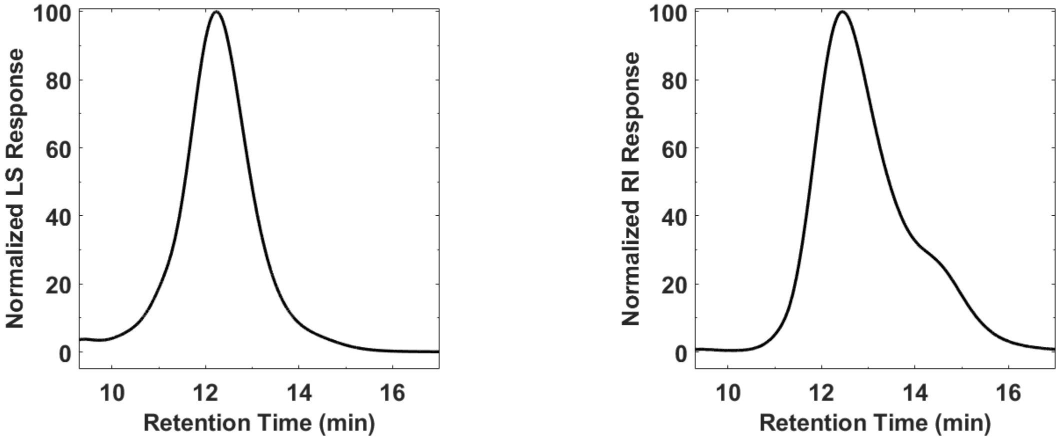

Fig. S328 GPC spectrum of PIP 500 equivalents generated by **$Tm(CH_2SiMe_3)_3(THF)_2$** and 2 equivalents $[Ph_3C][B(C_6F_5)_4]$ from **Table S4**, entry 5 (7 h): (left) LS; (right) RI.

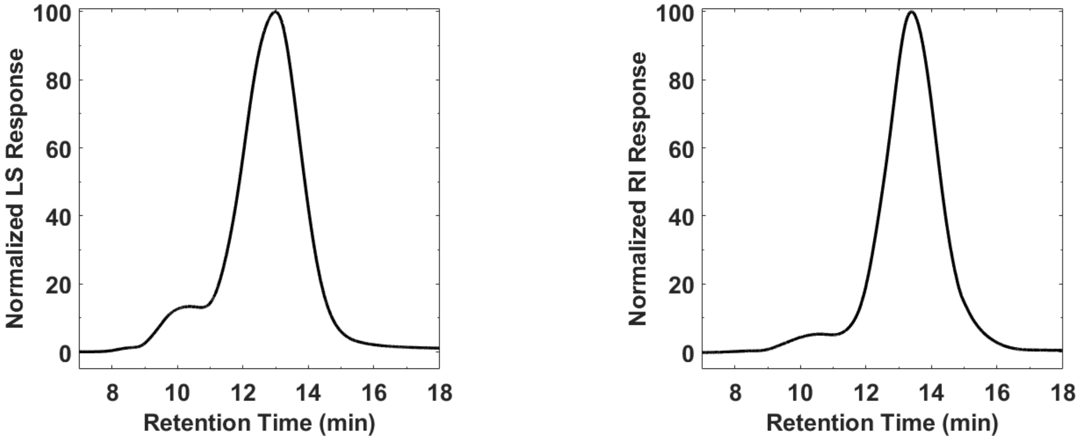

Fig. S329 GPC spectrum of PIP 500 equivalents generated by **$Sm(CH_2SiMe_3)_3(THF)_3$**, 1 equivalent $[Ph_3C][B(C_6F_5)_4]$, and 1 equivalent PPh_3 from **Table S4**, entry 6 (7 h): (left) LS; (right) RI.

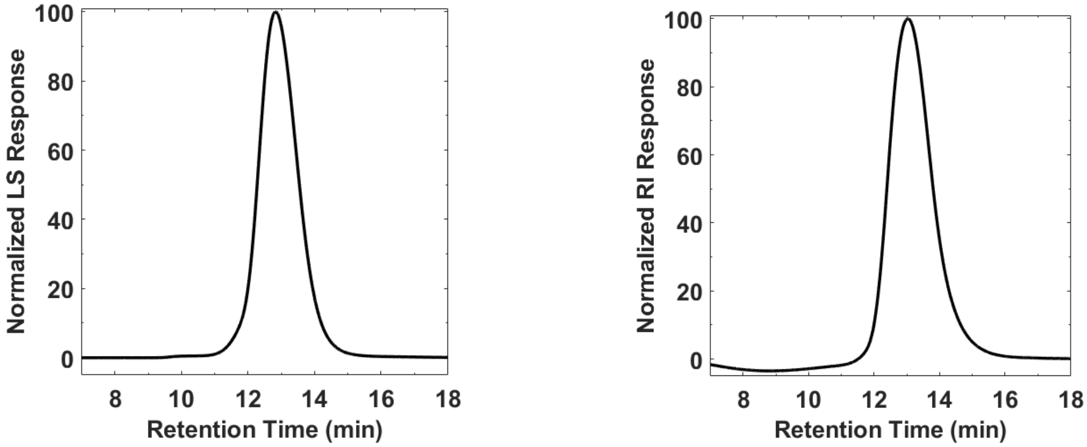

Fig. S330 GPC spectrum of PIP 500 equivalents generated by **$Gd(CH_2SiMe_3)_3(THF)_2$**, 1 equivalent $[Ph_3C][B(C_6F_5)_4]$, and 1 equivalent PPh_3 from **Table S4**, entry 7 (7 h): (left) LS; (right) RI.

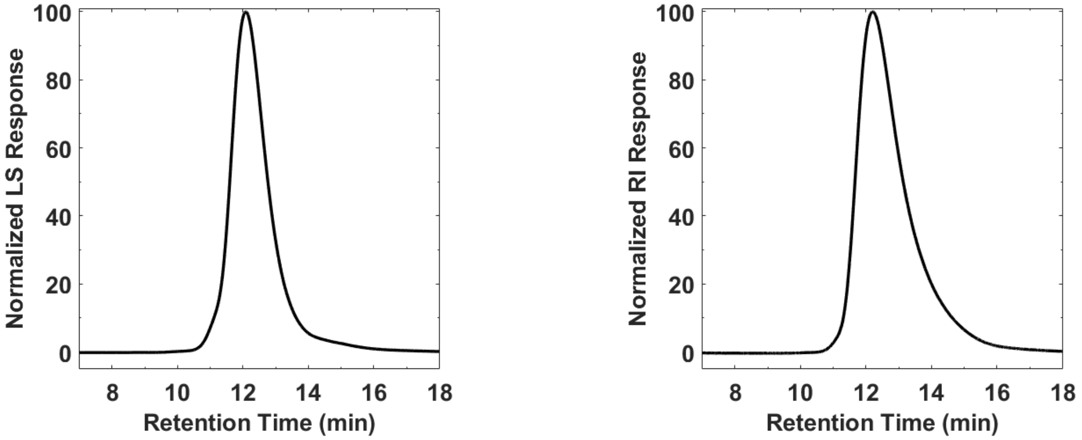

Fig. S331 GPC spectrum of PIP 500 equivalents generated by **Gd(CH$_2$SiMe$_3$)$_3$(THF)$_2$**, 2 equivalents [Ph$_3$C][B(C$_6$F$_5$)$_4$], and 1 equivalent PPh$_3$ from **Table S4**, entry 8 (7 h): (left) LS; (right) RI.

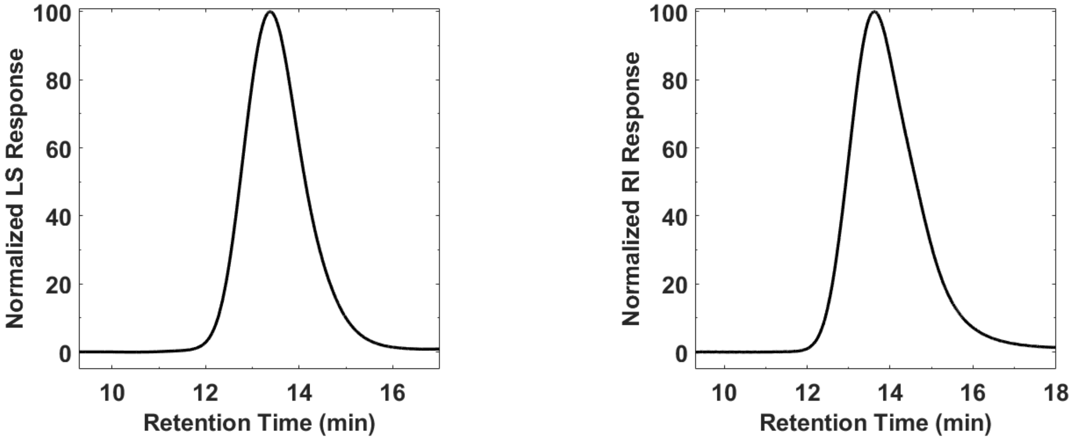

Fig. S332 GPC spectrum of PIP 500 equivalents generated by **Tm(CH$_2$SiMe$_3$)$_3$(THF)$_2$**, 1 equivalent [Ph$_3$C][B(C$_6$F$_5$)$_4$], and 1 equivalent PPh$_3$ from **Table S4**, entry 9 (7 h): (left) LS; (right) RI.

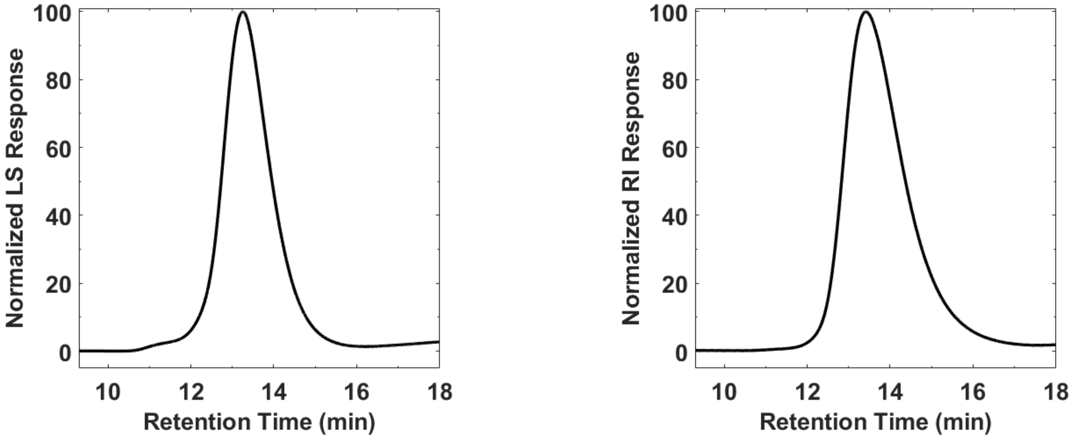

Fig. S333 GPC spectrum of PIP 500 equivalents generated by **Tm(CH$_2$SiMe$_3$)$_3$(THF)$_2$**, 2 equivalents [Ph$_3$C][B(C$_6$F$_5$)$_4$], and 1 equivalent PPh$_3$ from **Table S4**, entry 10 (7 h): (left) LS; (right) RI.

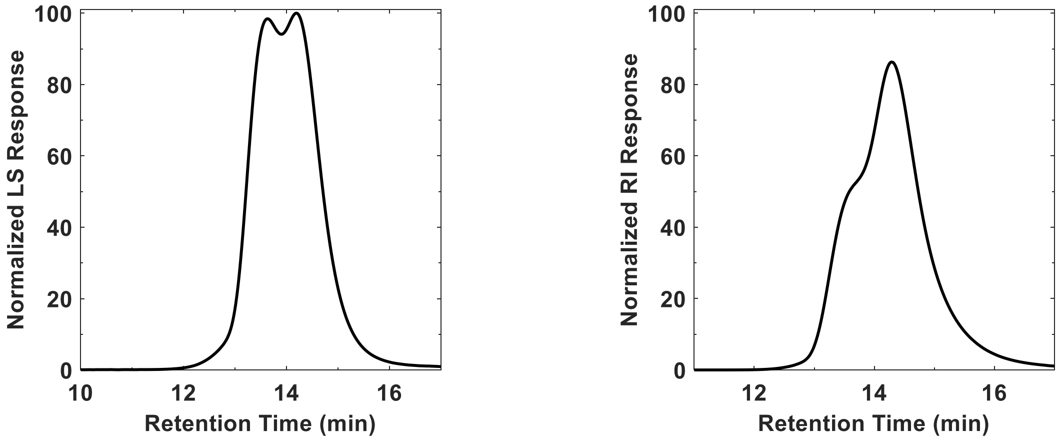

Fig. S334 GPC spectrum of PIP 500 equivalents generated by $\mathbf{Y(CH_2SiMe_3)_3(THF)_2}$ and 1 equivalent $[Ph_3C][B(C_6F_5)_4]$ from **Table 3**, entry 1 (7 h): (left) LS; (right) RI.

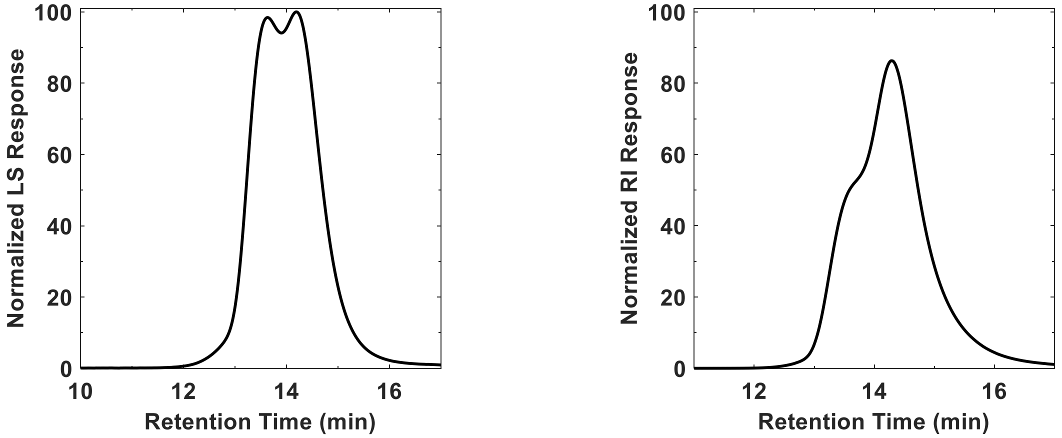

Fig. S335 GPC spectrum of PIP 500 equivalents generated by $\mathbf{Y(CH_2SiMe_3)_3(THF)_2}$, 1 equivalent $[Ph_3C][B(C_6F_5)_4]$, and 1 equivalent PPh_3 from **Table 3**, entry 2 (7 h): (left) LS; (right) RI.

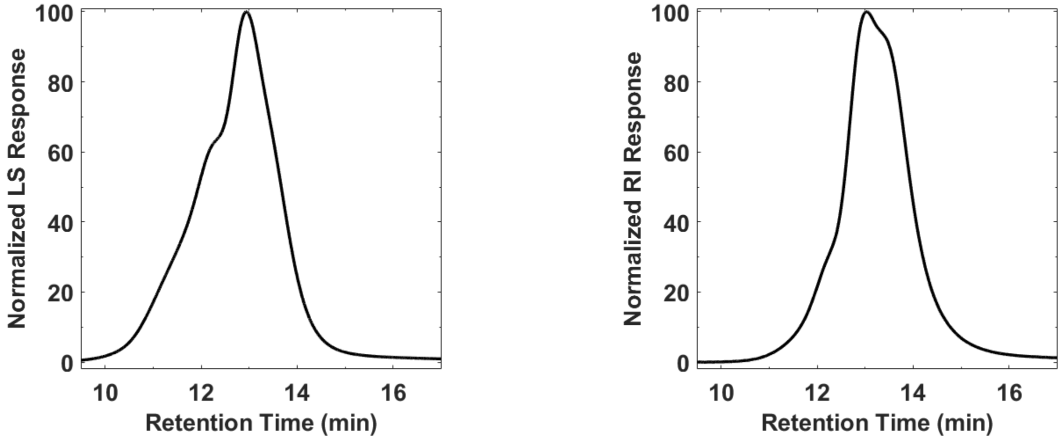

Fig. S336 GPC spectrum of PIP 500 equivalents generated by $\mathbf{Y(CH_2SiMe_3)_3(THF)_2}$ and 1.5 equivalents $[Ph_3C][B(C_6F_5)_4]$ from **Table 3**, entry 3 (7 h): (left) LS; (right) RI.

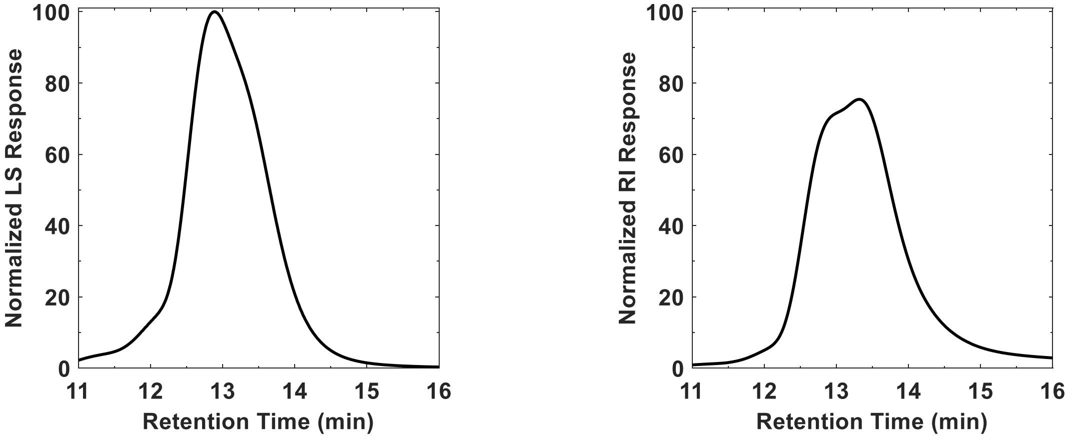

Fig. S337 GPC spectrum of PIP 500 equivalents generated by **$Y(CH_2SiMe_3)_3(THF)_2$**, 1.5 equivalents $[Ph_3C][B(C_6F_5)_4]$, and 1 equivalent PPh_3 from **Table 3**, entry 4 (7 h): (left) LS; (right) RI.

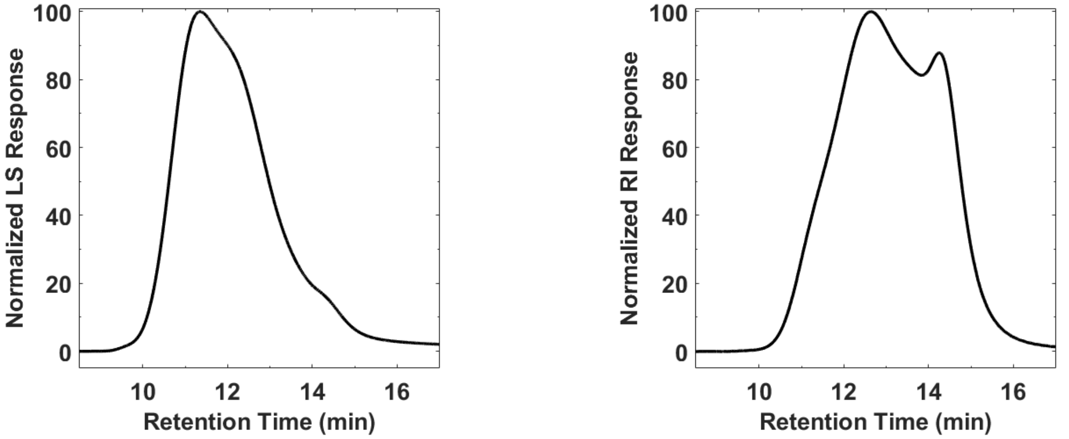

Fig. S338 GPC spectrum of PIP 500 equivalents generated by **$Y(CH_2SiMe_3)_3(THF)_2$** and 2 equivalents $[Ph_3C][B(C_6F_5)_4]$ from **Table 3**, entry 5 (7 h): (left) LS; (right) RI.

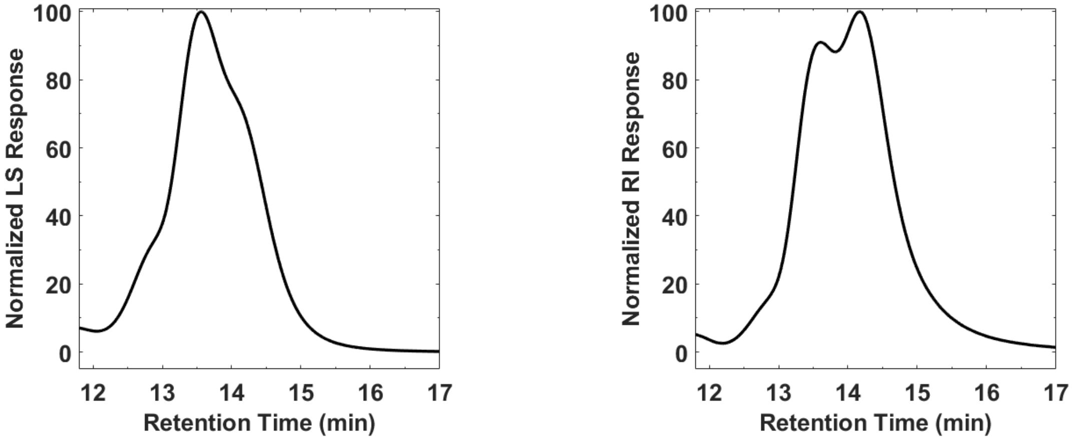

Fig. S339 GPC spectrum of PIP 500 equivalents generated by **$Y(CH_2SiMe_3)_3(THF)_2$**, 2 equivalents $[Ph_3C][B(C_6F_5)_4]$, and 1 equivalent PPh_3 from **Table 3**, entry 6 (7 h): (left) LS; (right) RI.

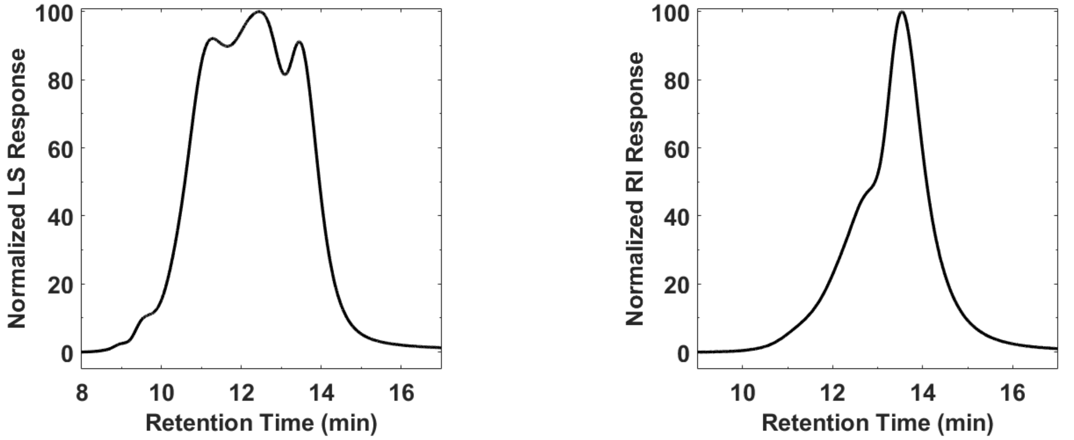

Fig. S340 GPC spectrum of PIP 500 equivalents generated by **$Y(CH_2SiMe_3)_3(THF)_2$** and 2.5 equivalents $[Ph_3C][B(C_6F_5)_4]$ from **Table 3**, entry 7 (7 h): (left) LS; (right) RI.

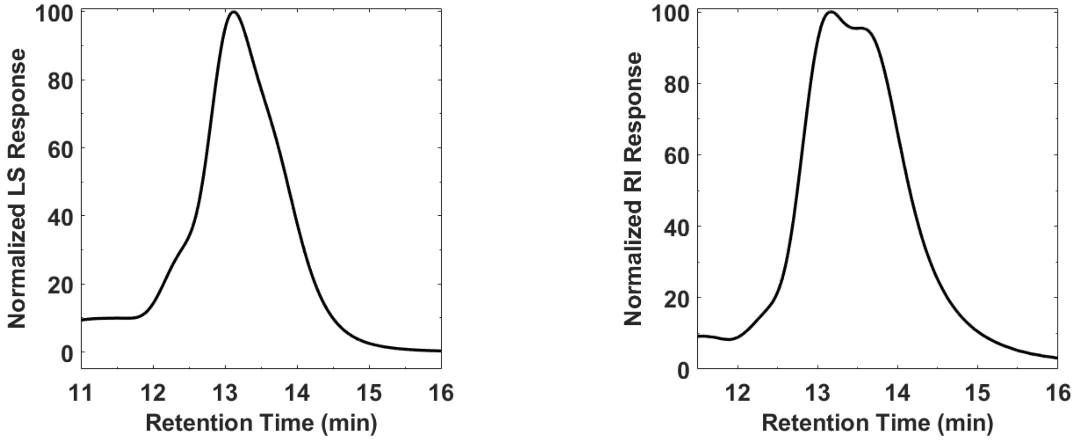

Fig. S341 GPC spectrum of PIP 500 equivalents generated by **$Y(CH_2SiMe_3)_3(THF)_2$**, 2.5 equivalents $[Ph_3C][B(C_6F_5)_4]$, and 1 equivalent PPh_3 from **Table 3**, entry 8 (7 h): (left) LS; (right) RI.

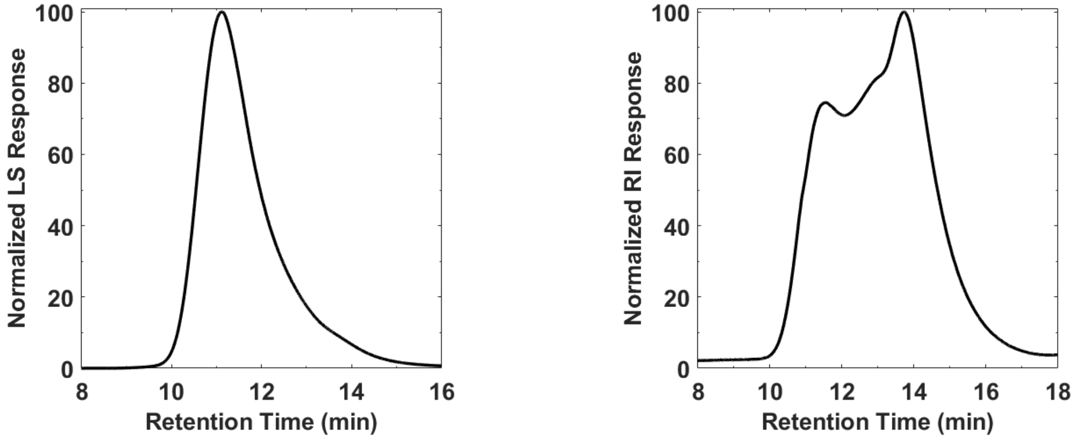

Fig. S342 GPC spectrum of PIP 500 equivalents generated by **$Y(CH_2SiMe_3)_3(THF)_2$** and 3 equivalents $[Ph_3C][B(C_6F_5)_4]$ from **Table 3**, entry 9 (7 h): (left) LS; (right) RI.

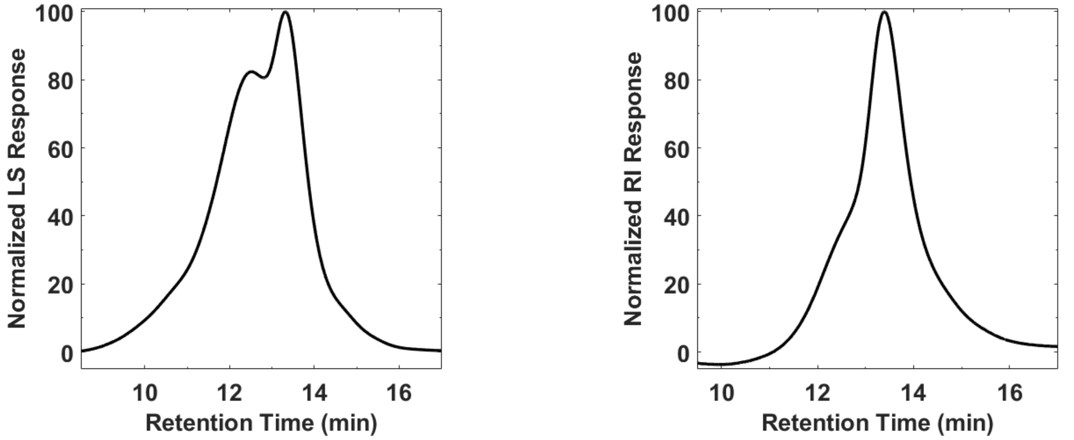

Fig. S343 GPC spectrum of PIP 500 equivalents generated by **Y(CH$_2$SiMe$_3$)$_3$(THF)$_2$**, 3 equivalents [Ph$_3$C][B(C$_6$F$_5$)$_4$], and 1 equivalent PPh$_3$ from **Table 3**, entry 10 (7 h): (left) LS; (right) RI.

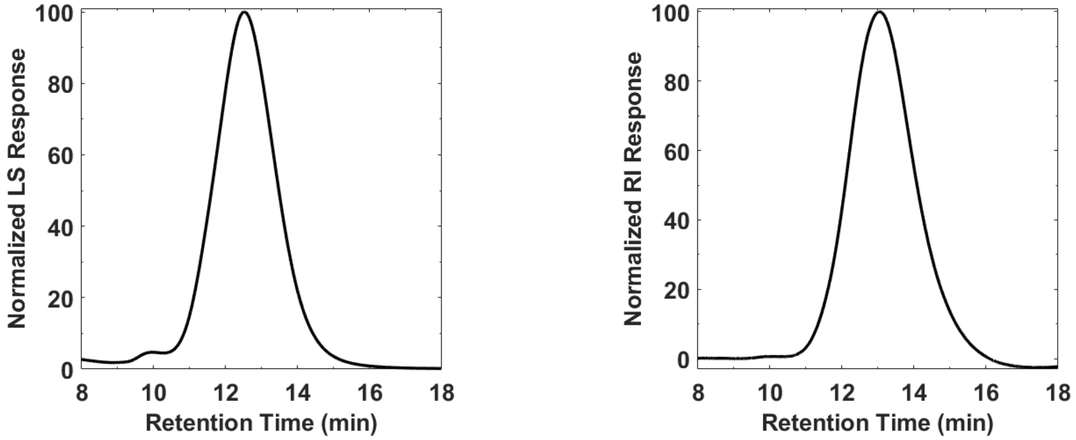

Fig. S344 GPC spectrum of PIP 500 equivalents generated by **Y(CH$_2$SiMe$_3$)$_3$(THF)$_2$**, 2 equivalents [Ph$_3$C][B(C$_6$F$_5$)$_4$], and 5 equivalents AlMe$_3$ from **Table 4**, entry 1 (30 min): (left) LS; (right) RI.

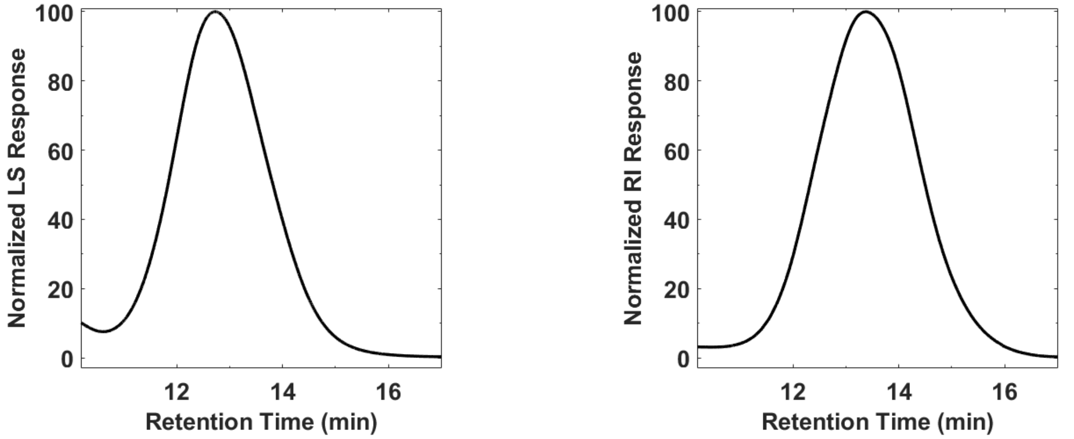

Fig. S345 GPC spectrum of PIP 500 equivalents generated by **Y(CH$_2$SiMe$_3$)$_3$(THF)$_2$**, 2 equivalents [Ph$_3$C][B(C$_6$F$_5$)$_4$], and 10 equivalents AlMe$_3$ from **Table 4**, entry 2 (30 min): (left) LS; (right) RI.

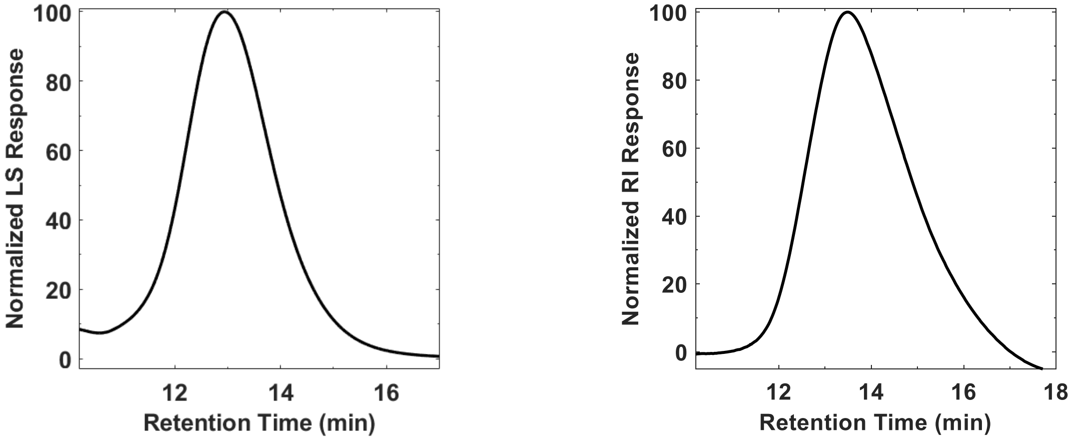

Fig. S346 GPC spectrum of PIP 500 equivalents generated by **$Y(CH_2SiMe_3)_3(THF)_2$**, 2 equivalents $[Ph_3C][B(C_6F_5)_4]$, and 15 equivalents $AlMe_3$ from **Table 4**, entry 3 (30 min): (left) LS; (right) RI.

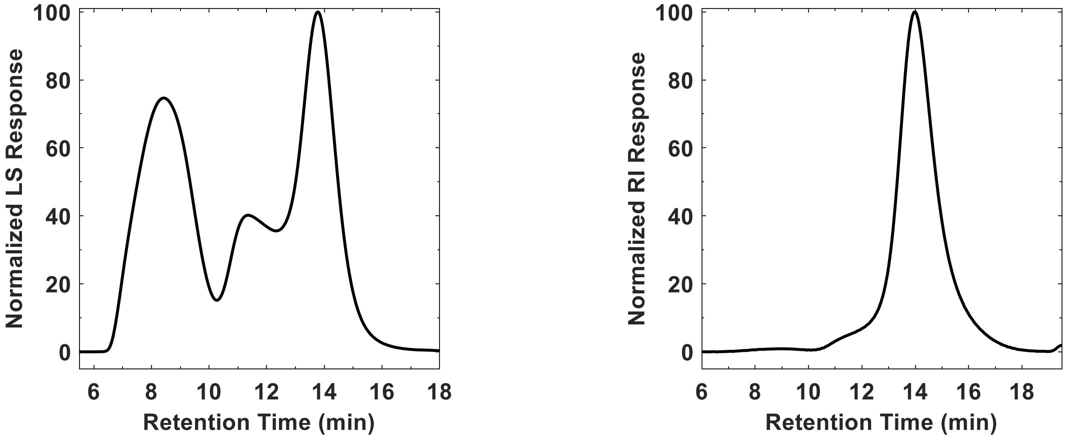

Fig. S347 GPC spectrum of PIP 500 equivalents generated by **$Y(CH_2SiMe_3)_3(THF)_2$**, 2 equivalents $[Ph_3C][B(C_6F_5)_4]$, and 5 equivalents $AlEt_3$ from **Table 4**, entry 4 (30 min): (left) LS; (right) RI.

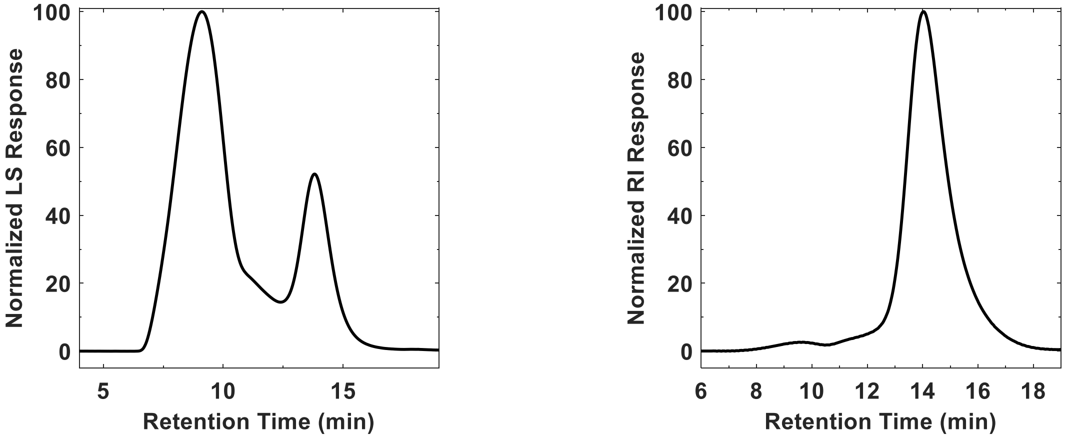

Fig. S348 GPC spectrum of PIP 500 equivalents generated by **$Y(CH_2SiMe_3)_3(THF)_2$**, 2 equivalents $[Ph_3C][B(C_6F_5)_4]$, and 10 equivalents $AlEt_3$ from **Table 4**, entry 5 (30 min): (left) LS; (right) RI.

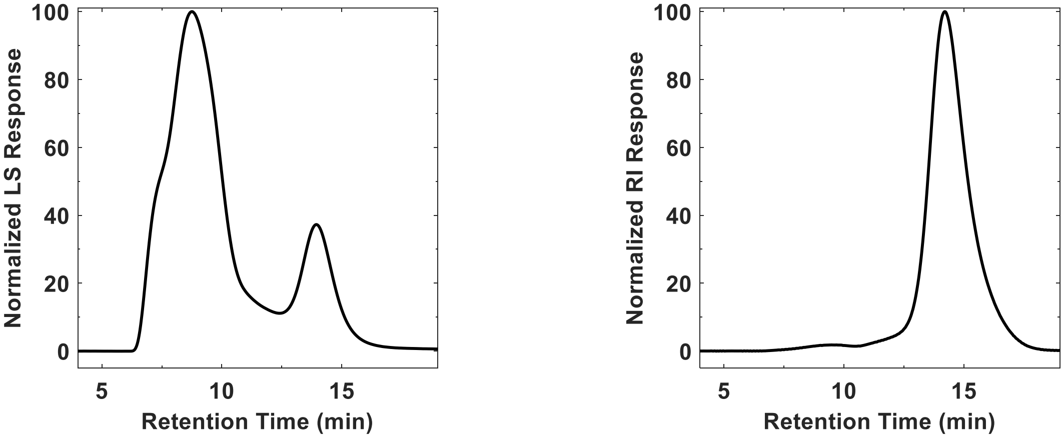

Fig. S349 GPC spectrum of PIP 500 equivalents generated by **$Y(CH_2SiMe_3)_3(THF)_2$**, 2 equivalents $[Ph_3C][B(C_6F_5)_4]$, and 15 equivalents $AlEt_3$ from **Table 4**, entry 6 (30 min): (left) LS; (right) RI.

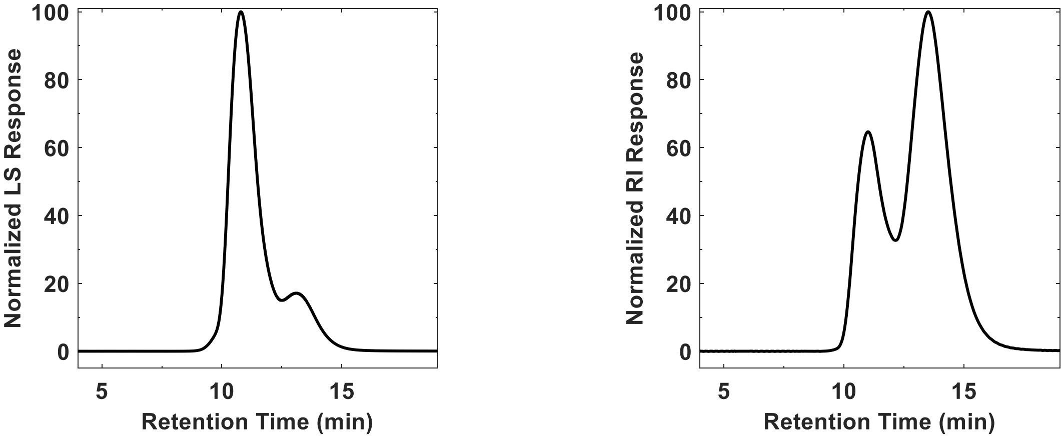

Fig. S350 GPC spectrum of PIP 500 equivalents generated by **$Y(CH_2SiMe_3)_3(THF)_2$**, 2 equivalents $[Ph_3C][B(C_6F_5)_4]$, and 5 equivalents Al^iBu_3 from **Table 4**, entry 7 (30 min): (left) LS; (right) RI.

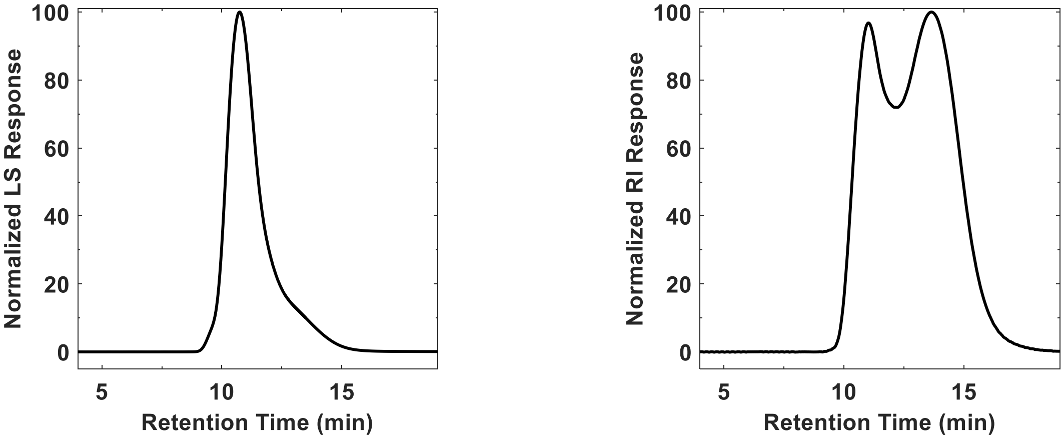

Fig. S351 GPC spectrum of PIP 500 equivalents generated by **$Y(CH_2SiMe_3)_3(THF)_2$**, 2 equivalents $[Ph_3C][B(C_6F_5)_4]$, and 10 equivalents Al^iBu_3 from **Table 4**, entry 8 (30 min): (left) LS; (right) RI.

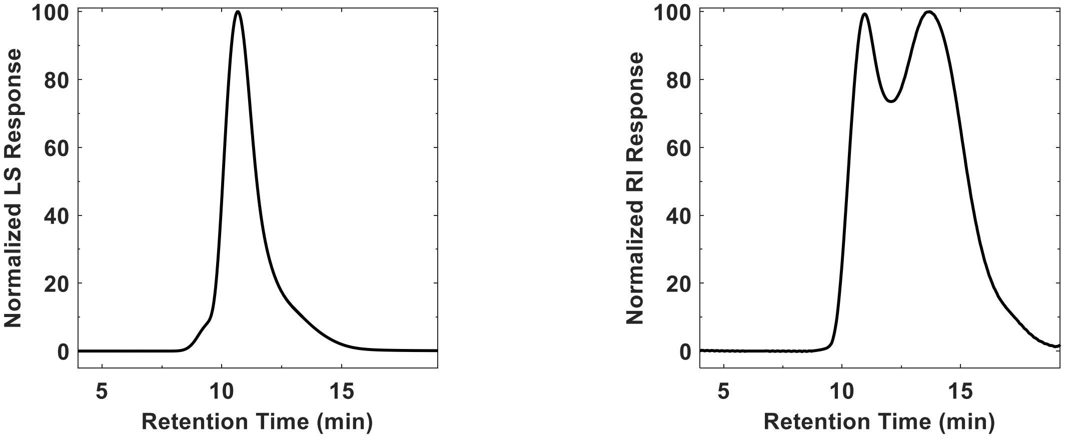

Fig. S352 GPC spectrum of PIP 500 equivalents generated by **$Y(CH_2SiMe_3)_3(THF)_2$**, 2 equivalents $[Ph_3C][B(C_6F_5)_4]$, and 15 equivalents Al^iBu_3 from **Table 4**, entry 9 (30 min): (left) LS; (right) RI.

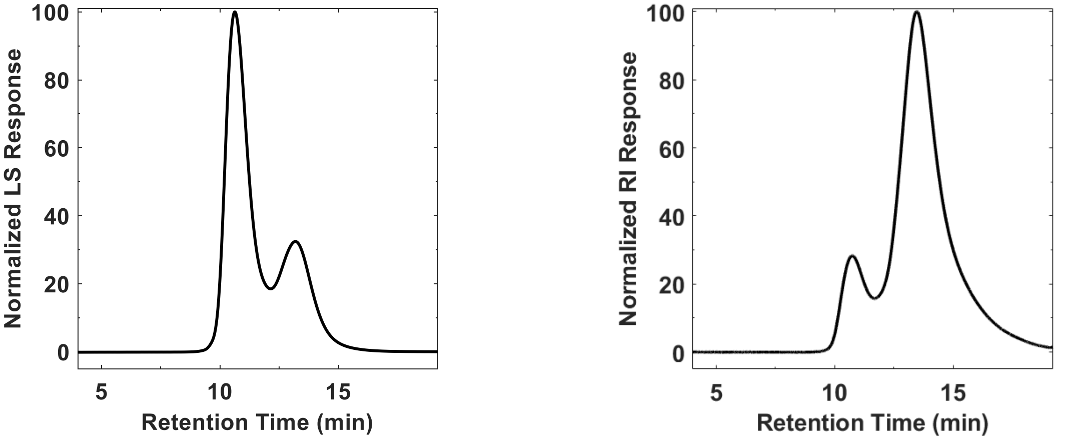

Fig. S353 GPC spectrum of PIP 500 equivalents generated by **$Y(CH_2SiMe_3)_3(THF)_2$**, 2 equivalents $[Ph_3C][B(C_6F_5)_4]$, 1 equivalent PPh_3, and 5 equivalents Al^iBu_3 from **Table 4**, entry 10 (30 min): (left) LS; (right) RI.

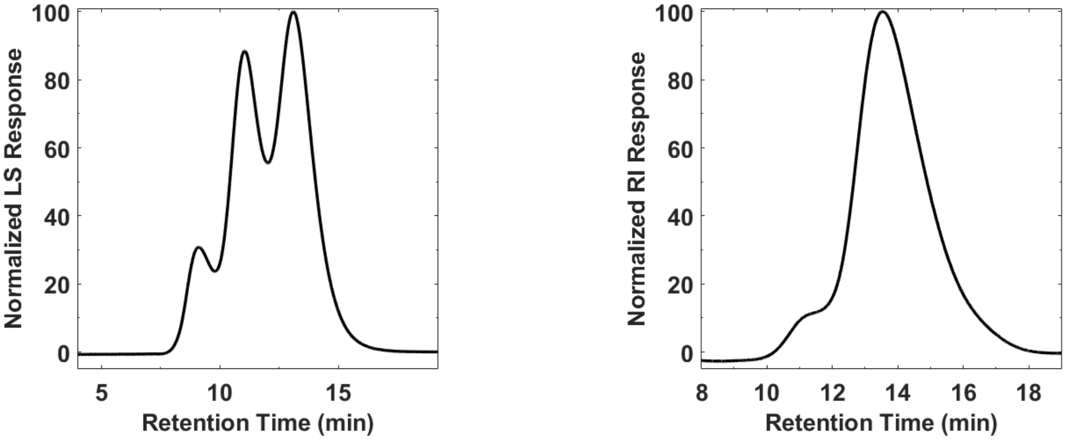

Fig. S354 GPC spectrum of PIP 500 equivalents generated by **$Y(CH_2SiMe_3)_3(THF)_2$**, 2 equivalents $[Ph_3C][B(C_6F_5)_4]$, 1 equivalent PPh_3, and 10 equivalents Al^iBu_3 from **Table 4**, entry 11 (30 min): (left) LS; (right) RI.

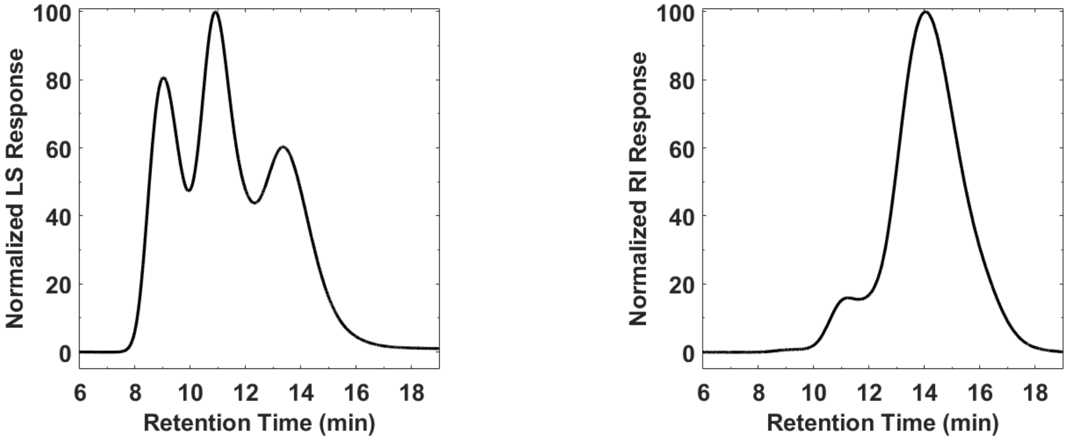

Fig. S355 GPC spectrum of PIP 500 equivalents generated by **$Y(CH_2SiMe_3)_3(THF)_2$**, 2 equivalents $[Ph_3C][B(C_6F_5)_4]$, 1 equivalent PPh_3, and 15 equivalents Al^iBu_3 from **Table 4**, entry 12 (30 min): (left) LS; (right) RI.

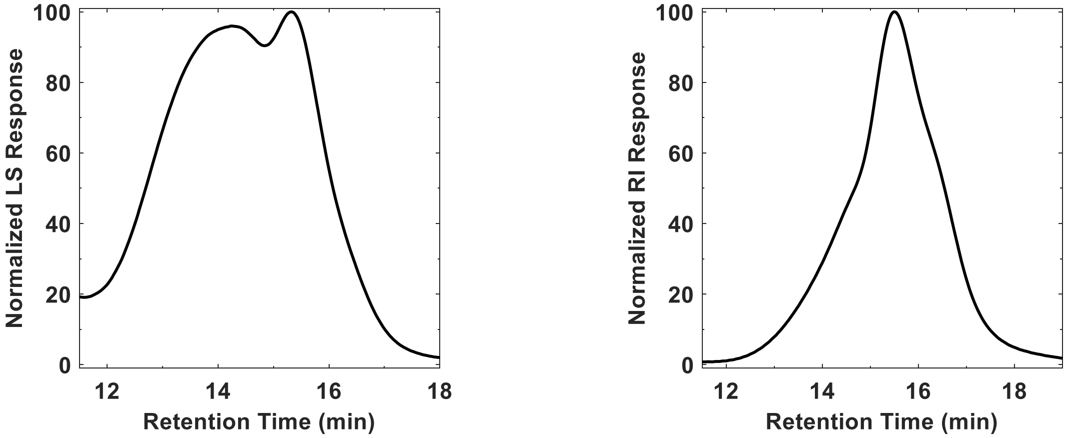

Fig. S356 GPC spectrum of PIP 500 equivalents generated by **$Sm(CH_2SiMe_3)_3(THF)_3$** and 1 equivalent $[Ph_3C][B(C_6F_5)_4]$ from **Table 5**, entry 1 (30 min): (left) LS; (right) RI.

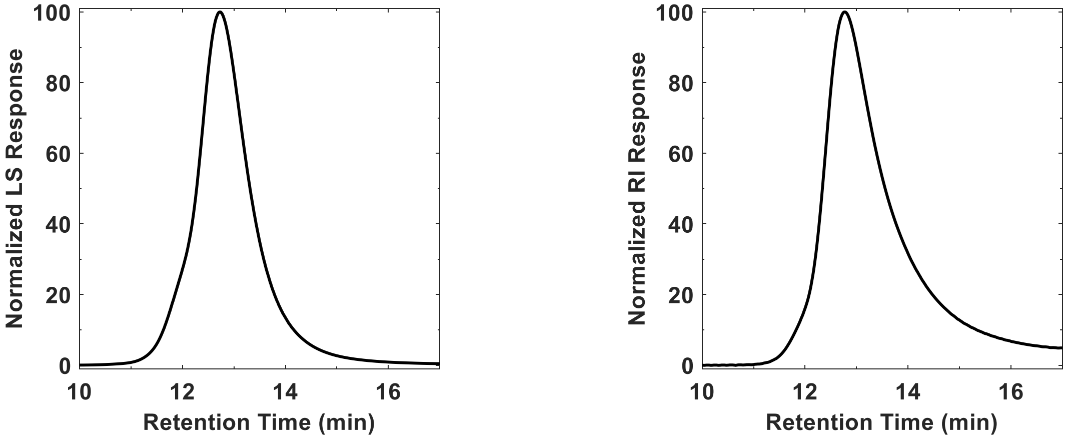

Fig. S357 GPC spectrum of PIP 500 equivalents generated by **$Sm(CH_2SiMe_3)_3(THF)_3$** and 2 equivalents $[Ph_3C][B(C_6F_5)_4]$ from **Table 5**, entry 2 (30 min): (left) LS; (right) RI.

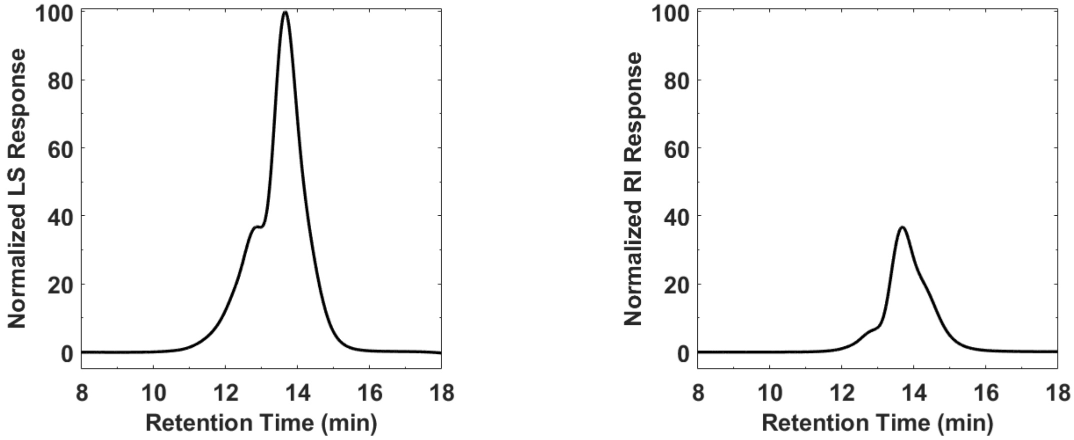

Fig. S358 GPC spectrum of PIP 500 equivalents generated by **$Gd(CH_2SiMe_3)_3(THF)_2$** and 1 equivalent $[Ph_3C][B(C_6F_5)_4]$ from **Table 5**, entry 3 (30 min): (left) LS; (right) RI.

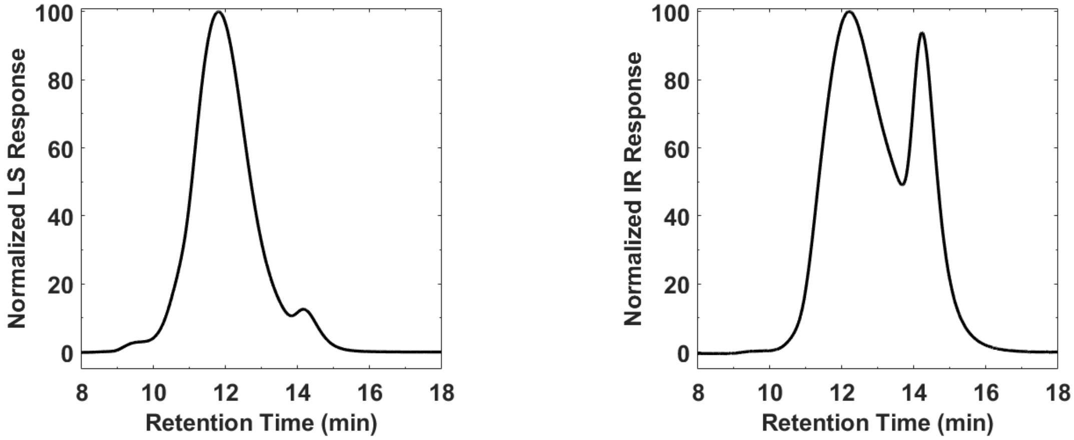

Fig. S359 GPC spectrum of PIP 500 equivalents generated by **$Gd(CH_2SiMe_3)_3(THF)_2$** and 2 equivalents $[Ph_3C][B(C_6F_5)_4]$ from **Table 5**, entry 4 (30 min): (left) LS; (right) RI.

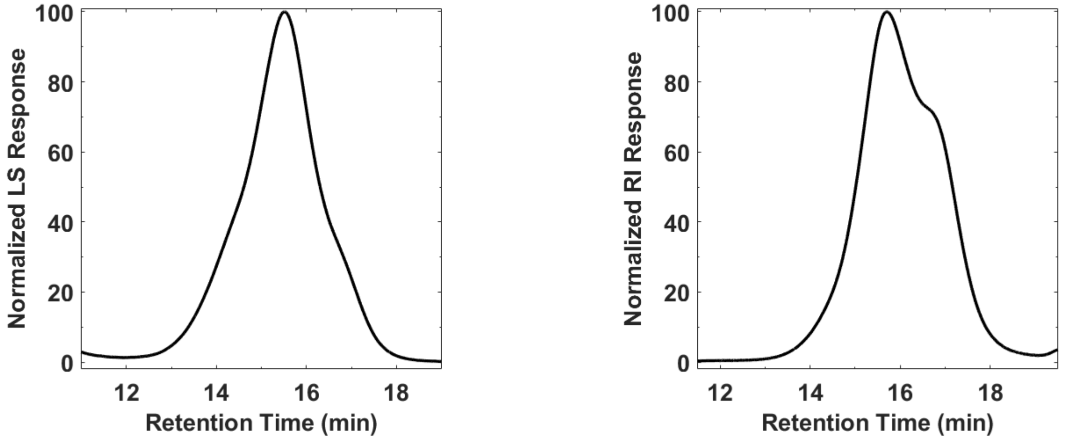

Fig. S360 GPC spectrum of PIP 500 equivalents generated by **$Y(CH_2SiMe_3)_3(THF)_2$** and 1 equivalent $[Ph_3C][B(C_6F_5)_4]$ from **Table 5**, entry 5 (30 min): (left) LS; (right) RI.

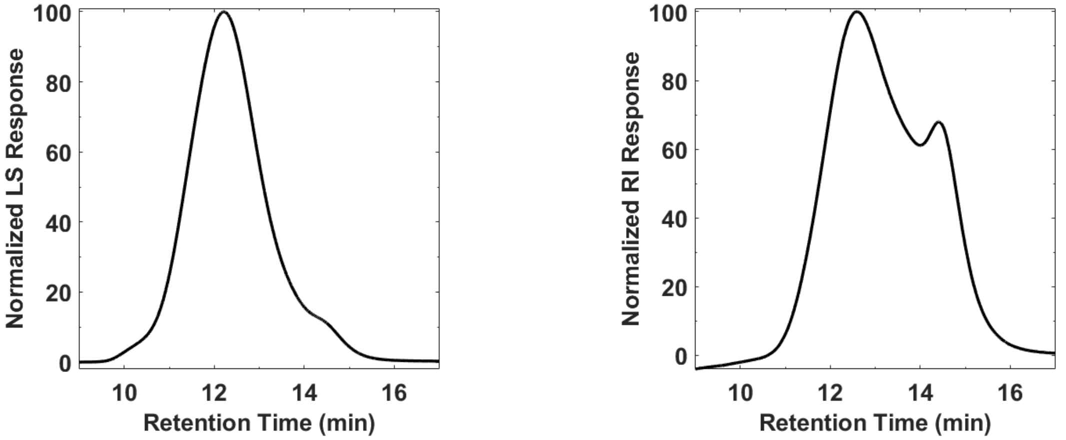

Fig. S361 GPC spectrum of PIP 500 equivalents generated by **$Y(CH_2SiMe_3)_3(THF)_2$** and 2 equivalents $[Ph_3C][B(C_6F_5)_4]$ from **Table 5**, entry 6 (30 min): (left) LS; (right) RI.

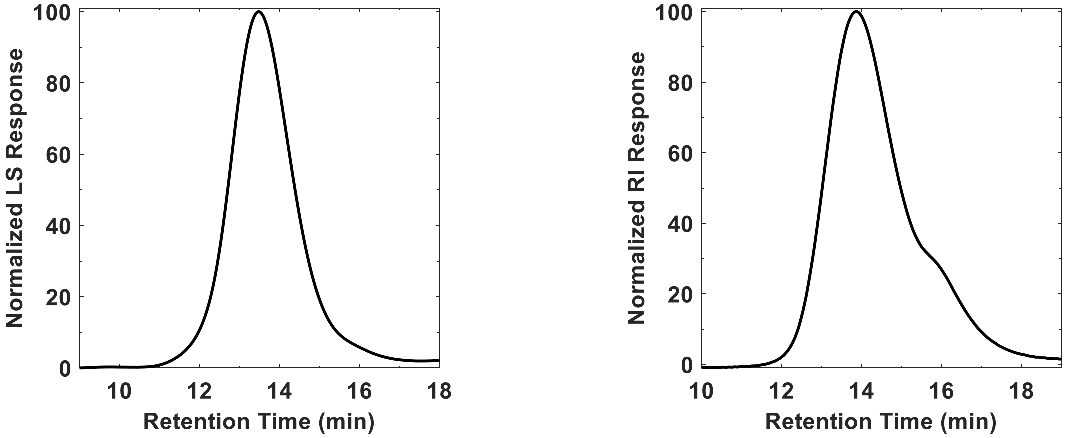

Fig. S362 GPC spectrum of PIP 500 equivalents generated by **$Tm(CH_2SiMe_3)_3(THF)_2$** and 1 equivalent $[Ph_3C][B(C_6F_5)_4]$ from **Table 5**, entry 7 (30 min): (left) LS; (right) RI.

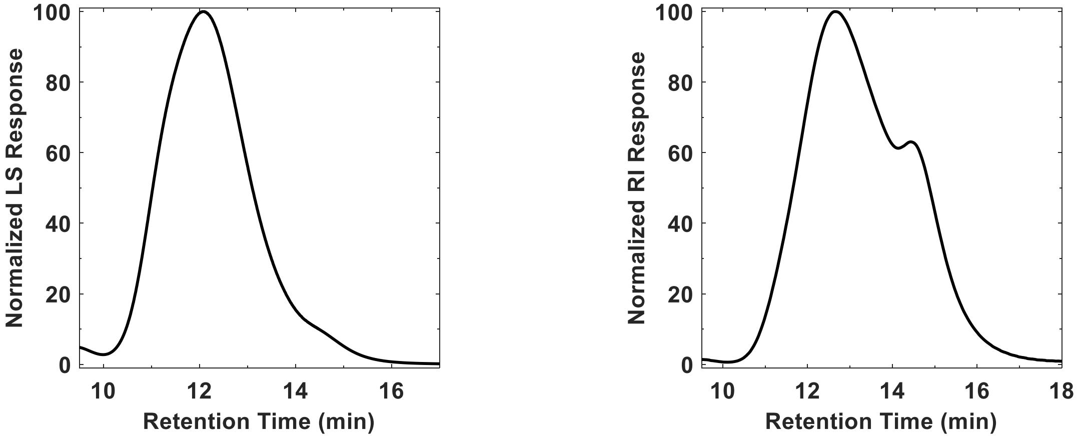

Fig. S363 GPC spectrum of PIP 500 equivalents generated by **$Tm(CH_2SiMe_3)_3(THF)_2$** and 2 equivalents $[Ph_3C][B(C_6F_5)_4]$ from **Table 5**, entry 8 (30 min): (left) LS; (right) RI.

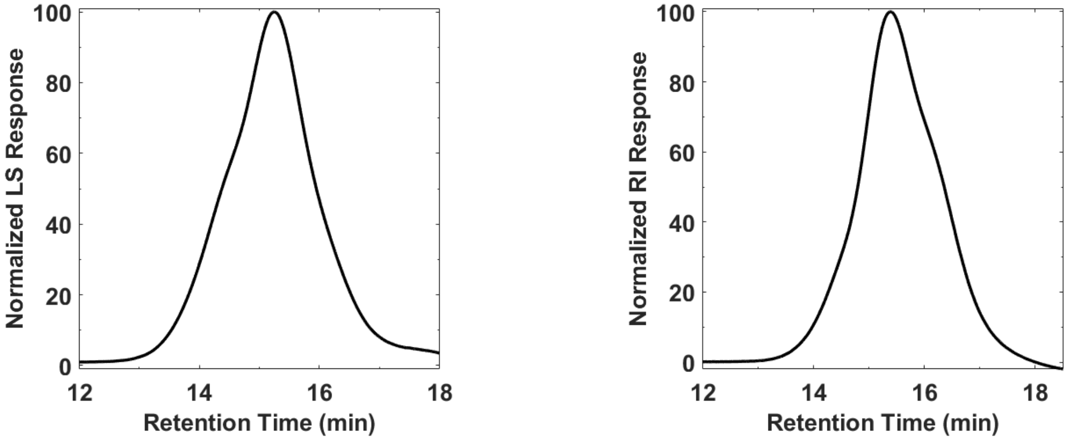

Fig. S364 GPC spectrum of PIP 500 equivalents generated by **Sm(CH_2SiMe_3)$_3$(THF)$_3$**, 1 equivalent [Ph_3C][B(C_6F_5)$_4$], and 1 equivalent PPh_3 from **Table 5**, entry 9 (30 min): (left) LS; (right) RI.

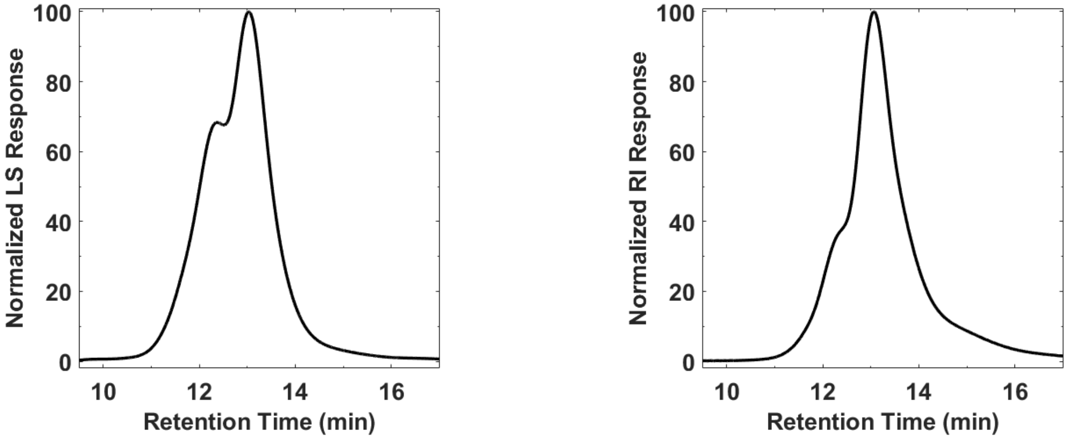

Fig. S365 GPC spectrum of PIP 500 equivalents generated by **Sm(CH_2SiMe_3)$_3$(THF)$_3$**, 2 equivalents [Ph_3C][B(C_6F_5)$_4$], and 1 equivalent PPh_3 from **Table 5**, entry 10 (30 min): (left) LS; (right) RI.

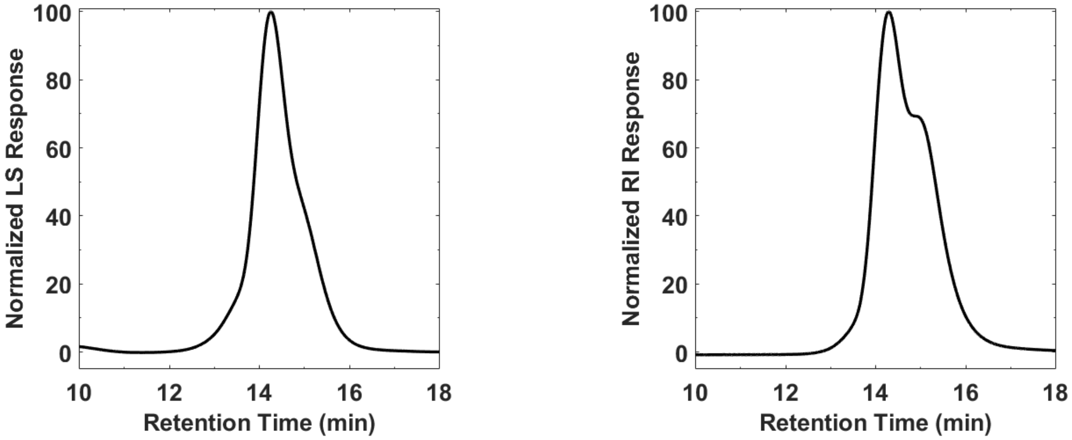

Fig. S366 GPC spectrum of PIP 500 equivalents generated by **Gd(CH_2SiMe_3)$_3$(THF)$_2$**, 1 equivalent [Ph_3C][B(C_6F_5)$_4$], and 1 equivalent PPh_3 from **Table 5**, entry 11 (30 min): (left) LS; (right) RI.

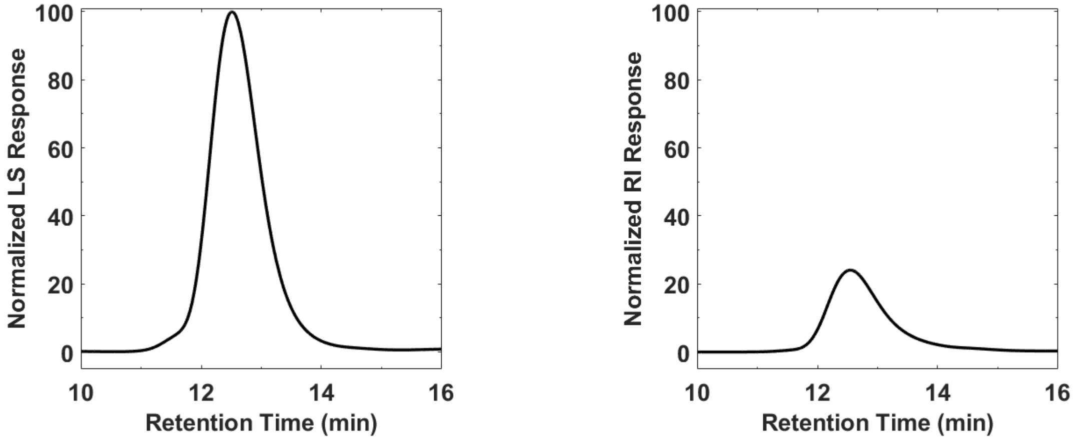

Fig. S367 GPC spectrum of PIP 500 equivalents generated by **$Gd(CH_2SiMe_3)_3(THF)_2$**, 2 equivalents $[Ph_3C][B(C_6F_5)_4]$, and 1 equivalent PPh_3 from **Table 5**, entry 12 (30 min): (left) LS; (right) RI.

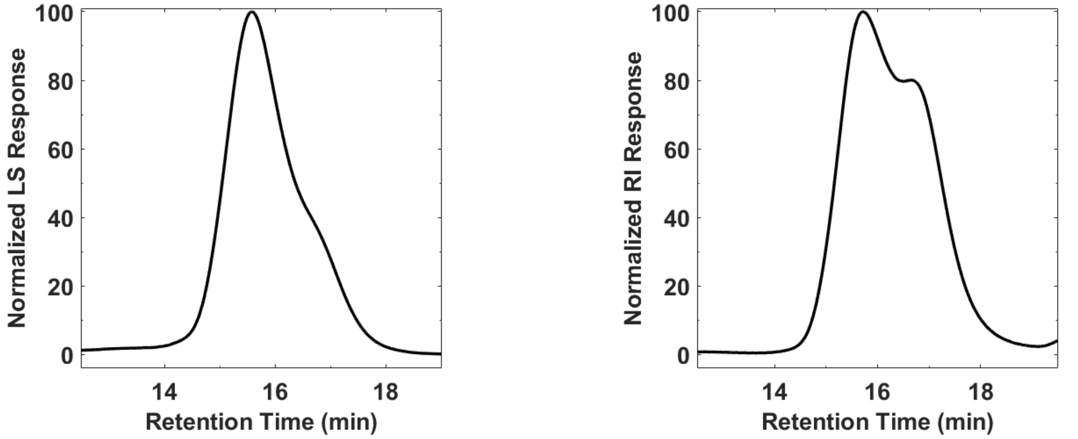

Fig. S368 GPC spectrum of PIP 500 equivalents generated by **$Y(CH_2SiMe_3)_3(THF)_2$**, 1 equivalent $[Ph_3C][B(C_6F_5)_4]$, and 1 equivalent PPh_3 from **Table 5**, entry 13 (30 min): (left) LS; (right) RI.

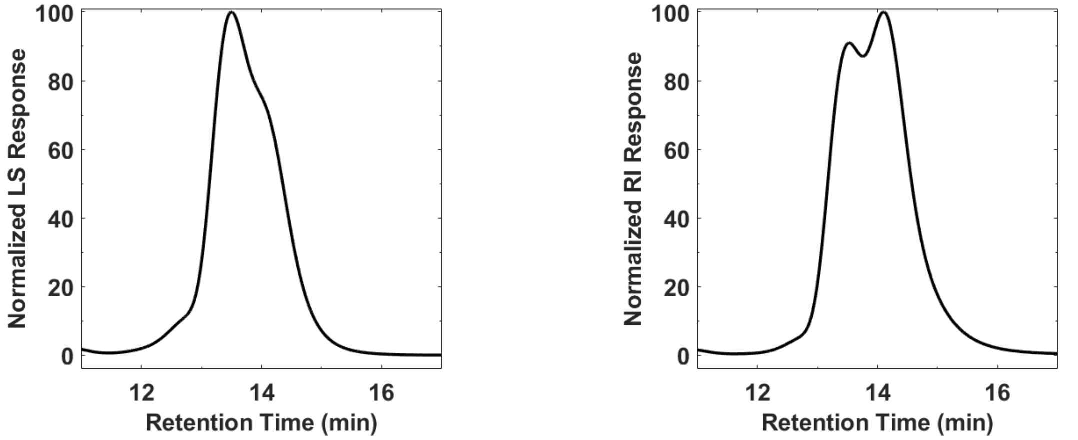

Fig. S369 GPC spectrum of PIP 500 equivalents generated by **$Y(CH_2SiMe_3)_3(THF)_2$**, 2 equivalents $[Ph_3C][B(C_6F_5)_4]$, and 1 equivalent PPh_3 from **Table 5**, entry 14 (30 min): (left) LS; (right) RI.

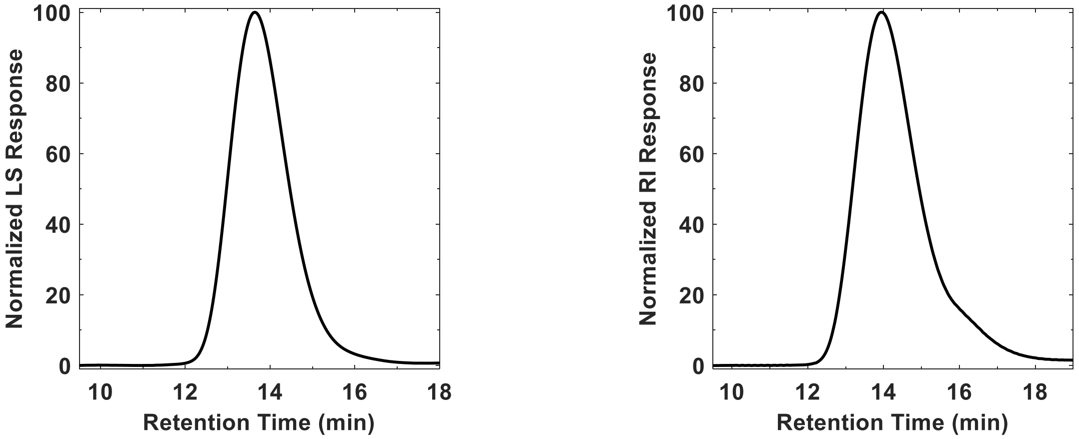

Fig. S370 GPC spectrum of PIP 500 equivalents generated by **$Tm(CH_2SiMe_3)_3(THF)_2$**, 1 equivalent $[Ph_3C][B(C_6F_5)_4]$, and 1 equivalent PPh_3 from **Table 5**, entry 15 (30 min): (left) LS; (right) RI.

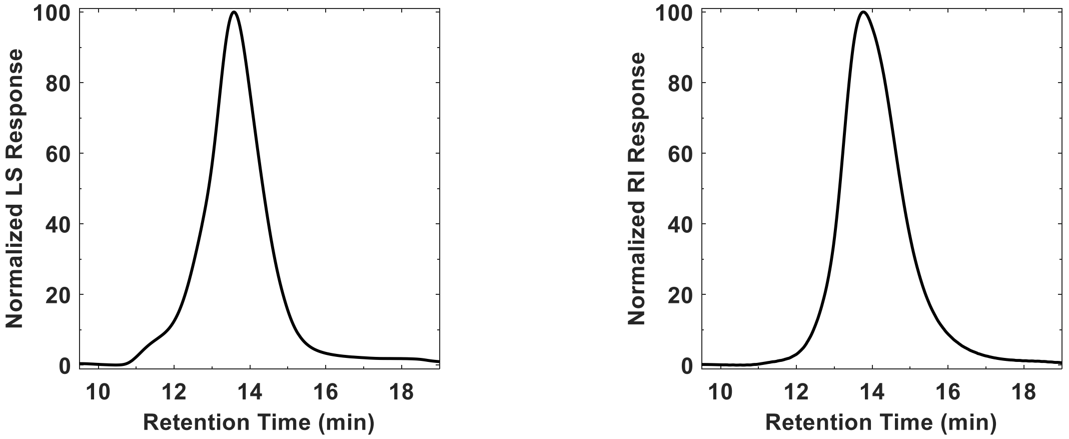

Fig. S371 GPC spectrum of PIP 500 equivalents generated by **$Tm(CH_2SiMe_3)_3(THF)_2$**, 2 equivalents $[Ph_3C][B(C_6F_5)_4]$, and 1 equivalent PPh_3 from **Table 5**, entry 16 (30 min): (left) LS; (right) RI.

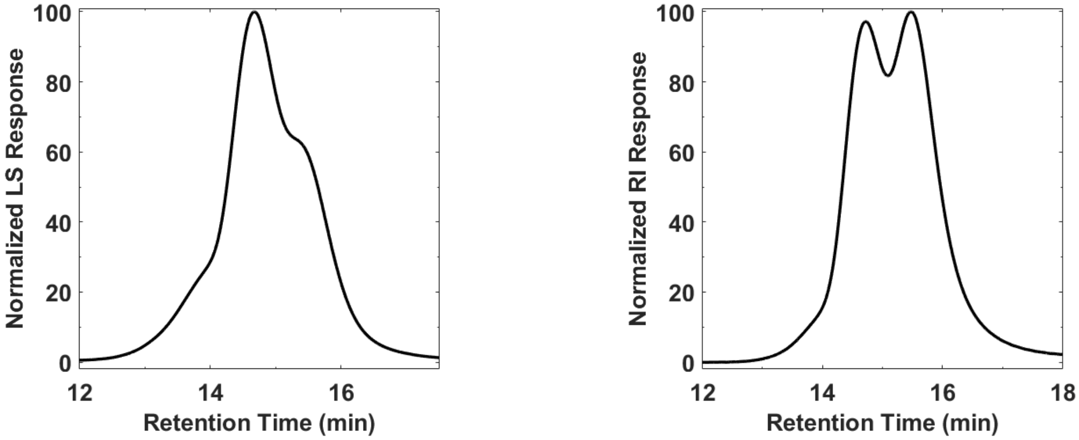

Fig. S372 GPC spectrum of PIP 500 equivalents generated by **$Sm(CH_2SiMe_3)_3(THF)_3$,** 1 equivalent PPh_3, and 2 equivalents $[Ph_3C][B(C_6F_5)_4]$ from **Table 6**, entry 1 ($[Ph_3C][B(C_6F_5)_4]$ addition time 0 min): (left) LS; (right) RI.

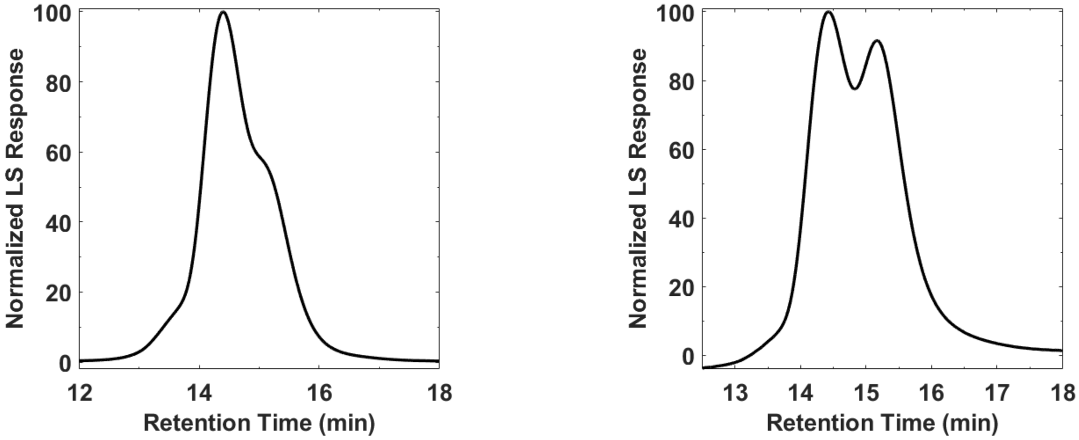

Fig. S373 GPC spectrum of PIP 500 equivalents generated by **$Sm(CH_2SiMe_3)_3(THF)_3$**, 1 equivalent PPh_3, and 2 equivalents $[Ph_3C][B(C_6F_5)_4]$ from **Table 6**, entry 2 ($[Ph_3C][B(C_6F_5)_4]$ addition time 10 min): (left) LS; (right) RI.

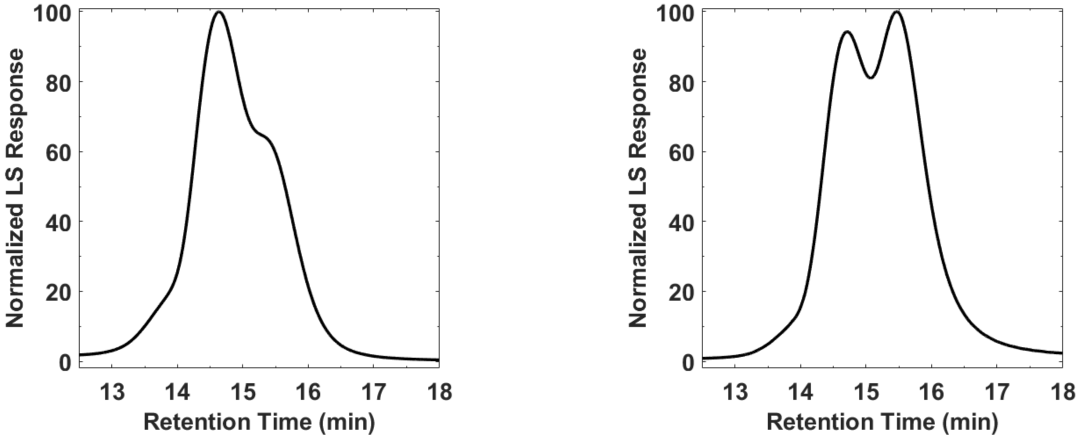

Fig. S374 GPC spectrum of PIP 500 equivalents generated by **$Sm(CH_2SiMe_3)_3(THF)_3$**, 1 equivalent PPh_3 and 2 equivalents $[Ph_3C][B(C_6F_5)_4]$ from **Table 6**, entry 3 ($[Ph_3C][B(C_6F_5)_4]$ addition time 30 min): (left) LS; (right) RI.

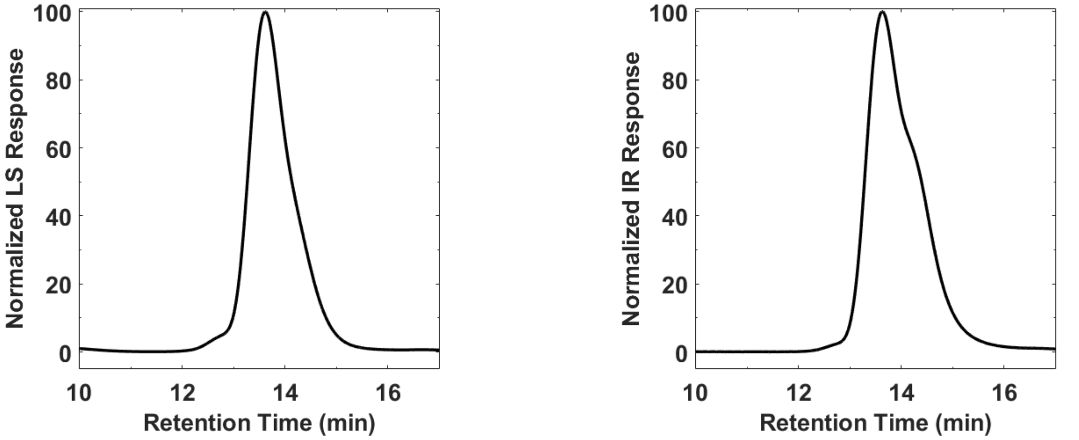

Fig. S375 GPC spectrum of PIP 500 equivalents generated by **$Gd(CH_2SiMe_3)_3(THF)_2$**, 1 equivalent PPh_3, and 2 equivalents $[Ph_3C][B(C_6F_5)_4]$ from **Table 6**, entry 4 ($[Ph_3C][B(C_6F_5)_4]$ addition time 0 min): (left) LS; (right) RI.

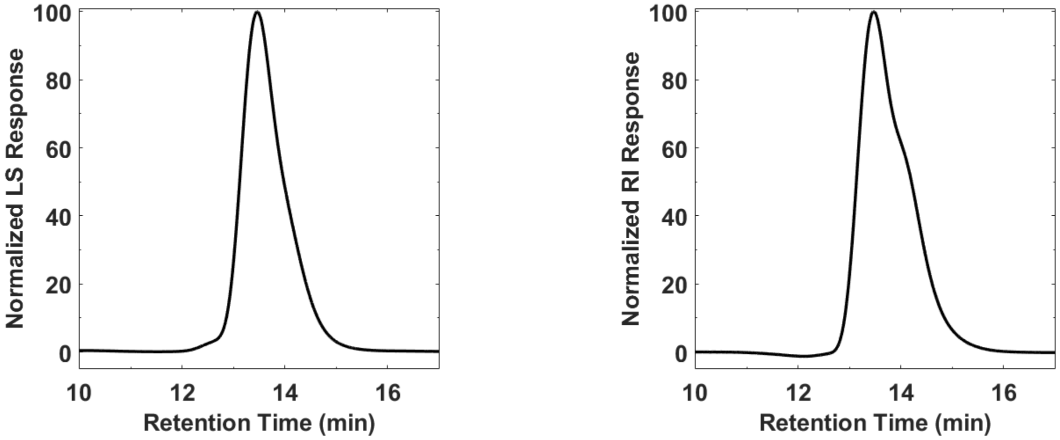

Fig. S376 GPC spectrum of PIP 500 equivalents generated by **$Gd(CH_2SiMe_3)_3(THF)_2$**, 1 equivalent PPh_3, and 2 equivalents $[Ph_3C][B(C_6F_5)_4]$ from **Table 6**, entry 5 ($[Ph_3C][B(C_6F_5)_4]$ addition time 10 min): (left) LS; (right) RI.

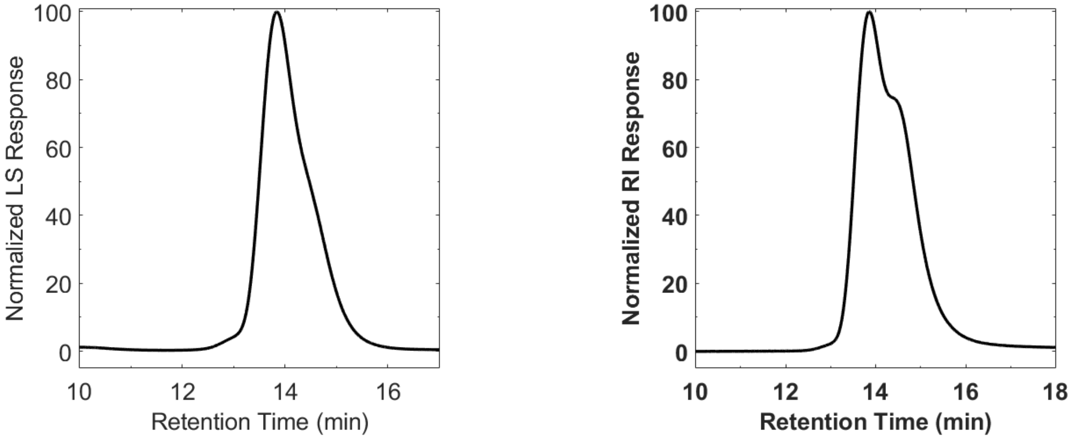

Fig. S377 GPC spectrum of PIP 500 equivalents generated by **$Gd(CH_2SiMe_3)_3(THF)_2$**, 1 equivalent PPh_3, and 2 equivalents $[Ph_3C][B(C_6F_5)_4]$ from **Table 6**, entry 6 ($[Ph_3C][B(C_6F_5)_4]$ addition time 30 min): (left) LS; (right) RI.

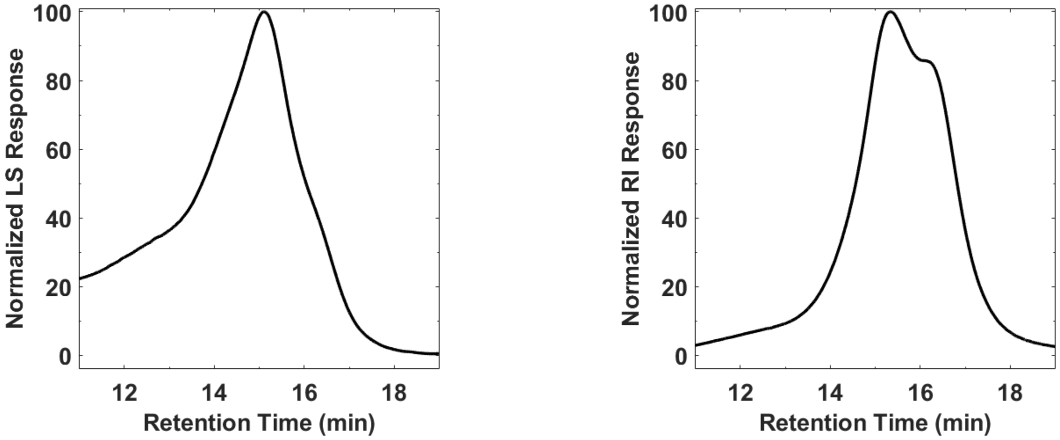

Fig. S378 GPC spectrum of PIP 500 equivalents generated by **$Y(CH_2SiMe_3)_3(THF)_2$**, 1 equivalent PPh_3, and 2 equivalents $[Ph_3C][B(C_6F_5)_4]$ from **Table 6**, entry 7 ($[Ph_3C][B(C_6F_5)_4]$ addition time 0 min): (left) LS; (right) RI.

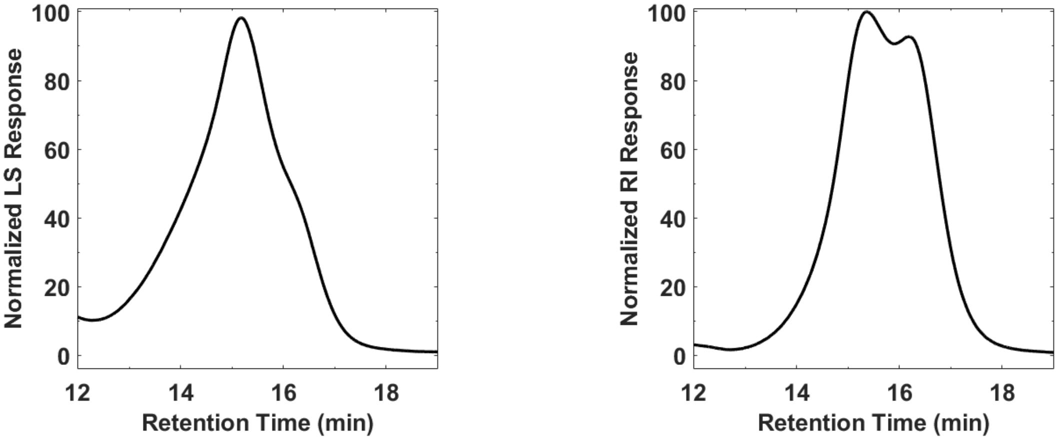

Fig. S379 GPC spectrum of PIP 500 equivalents generated by **$Y(CH_2SiMe_3)_3(THF)_2$**, 1 equivalent PPh_3, and 2 equivalents $[Ph_3C][B(C_6F_5)_4]$ from **Table 6**, entry 8 ($[Ph_3C][B(C_6F_5)_4]$ addition time 10 min): (left) LS; (right) RI.

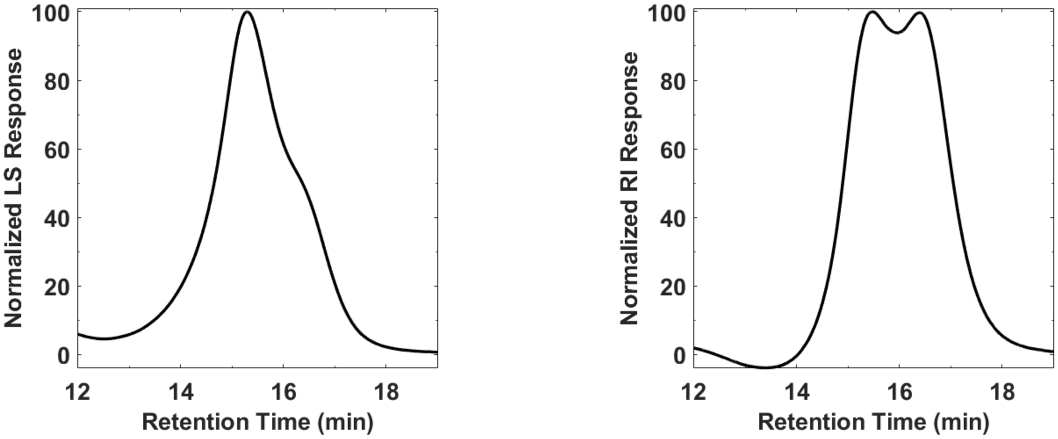

Fig. S380 GPC spectrum of PIP 500 equivalents generated by **$Y(CH_2SiMe_3)_3(THF)_2$**, 1 equivalent PPh_3, and 2 equivalents $[Ph_3C][B(C_6F_5)_4]$ from **Table 6**, entry 9 ($[Ph_3C][B(C_6F_5)_4]$ addition time 30 min): (left) LS; (right) RI.

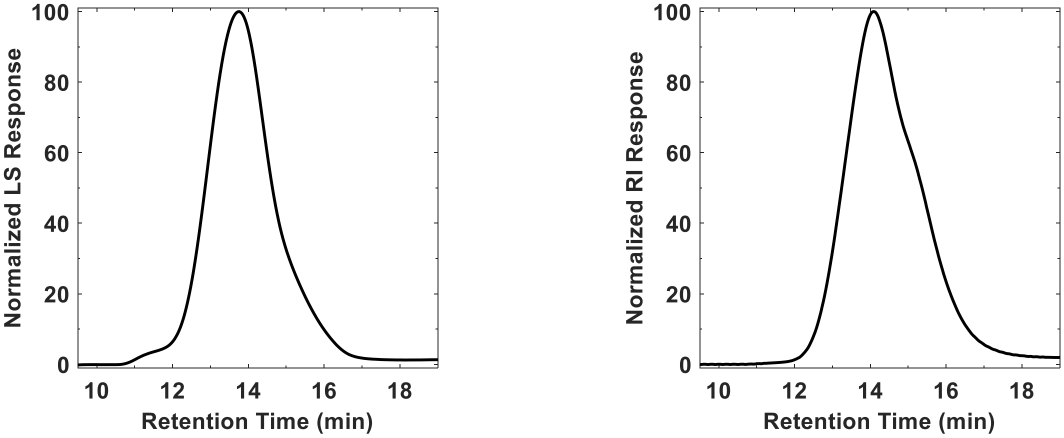

Fig. S381 GPC spectrum of PIP 500 equivalents generated by **$Tm(CH_2SiMe_3)_3(THF)_2$**, 1 equivalent PPh_3, and 2 equivalents $[Ph_3C][B(C_6F_5)_4]$ from **Table 6**, entry 10 ($[Ph_3C][B(C_6F_5)_4]$ addition time 0 min): (left) LS; (right) RI.

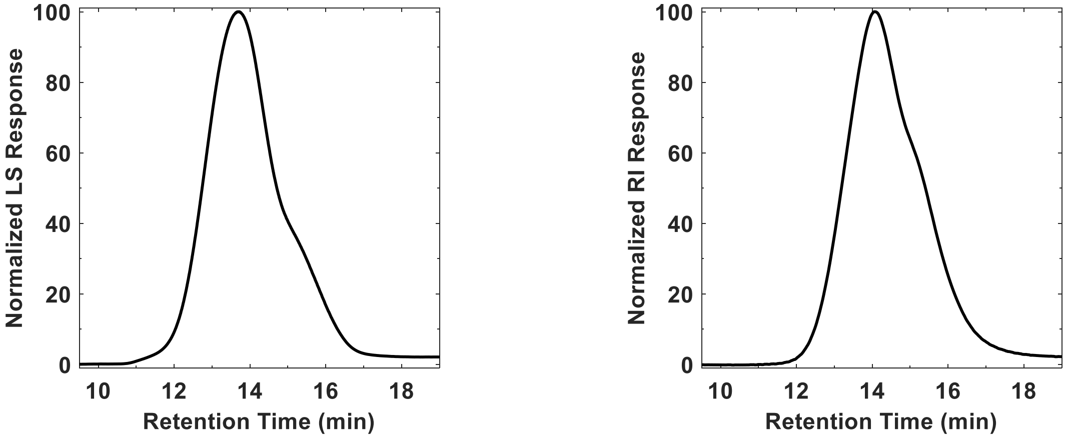

Fig. S382 GPC spectrum of PIP 500 equivalents generated by **$Tm(CH_2SiMe_3)_3(THF)_2$**, 1 equivalent PPh_3, and 2 equivalents $[Ph_3C][B(C_6F_5)_4]$ from **Table 6**, entry 11 ($[Ph_3C][B(C_6F_5)_4]$ addition time 10 min): (left) LS; (right) RI.

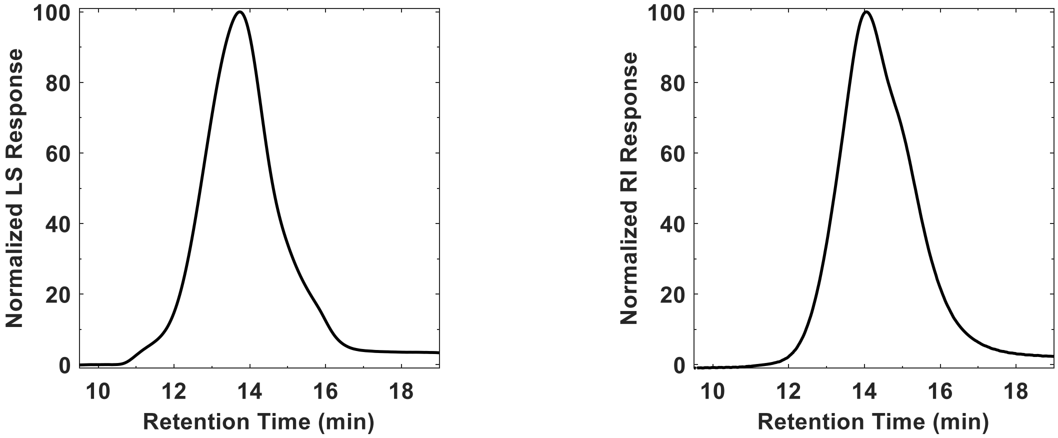

Fig. S383 GPC spectrum of PIP 500 equivalents generated by **$Tm(CH_2SiMe_3)_3(THF)_2$**, 1 equivalent PPh_3 and 2 equivalents $[Ph_3C][B(C_6F_5)_4]$ from **Table 6**, entry 12 ($[Ph_3C][B(C_6F_5)_4]$ addition time 30 min): (left) LS; (right) RI.

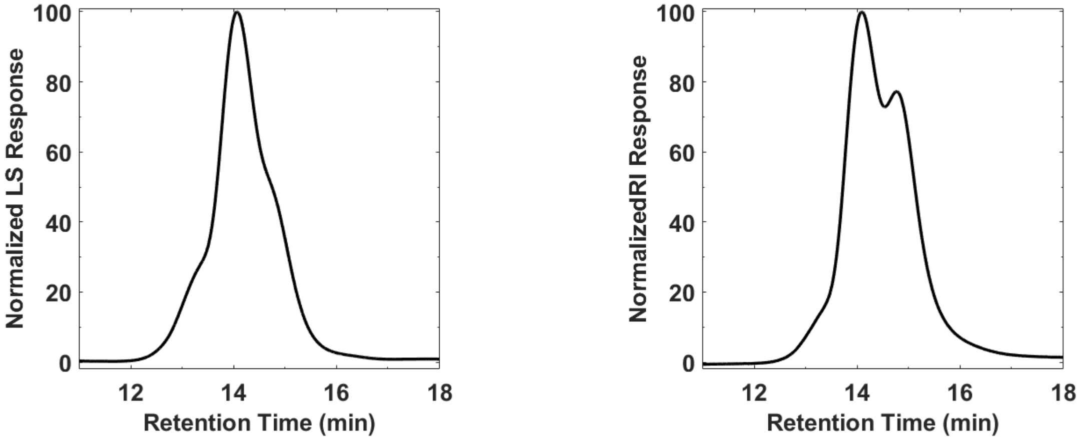

Fig. S384 GPC spectrum of PIP 500 equivalents generated by **$Sm(CH_2SiMe_3)_3(THF)_3$**, 2 equivalents $[Ph_3C][B(C_6F_5)_4]$, and 1 equivalent PPh_3 from **Table 7**, entry 1 (PPh_3 addition time 0 min): (left) LS; (right) RI.

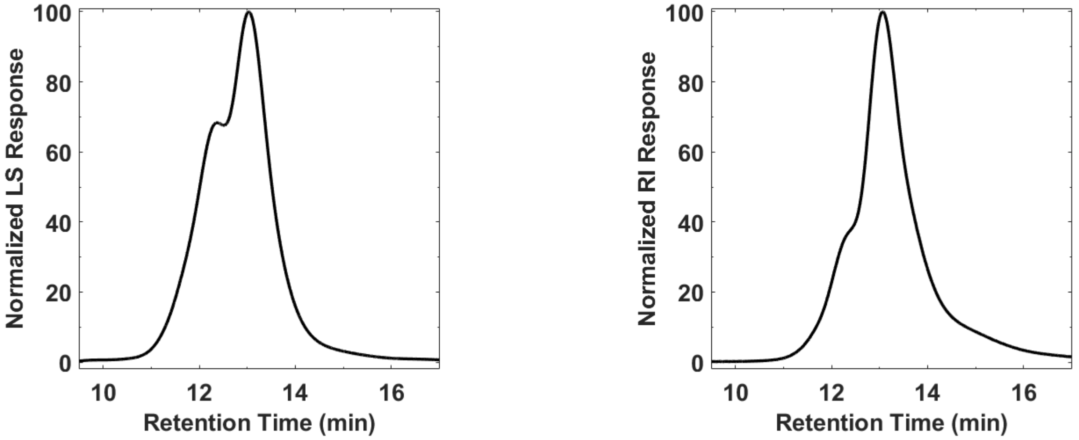

Fig. S385 GPC spectrum of PIP 500 equivalents generated by **$Sm(CH_2SiMe_3)_3(THF)_3$**, 2 equivalents $[Ph_3C][B(C_6F_5)_4]$, and 1 equivalent PPh_3 from **Table 7**, entry 2 (PPh_3 addition time 10 min): (left) LS; (right) RI.

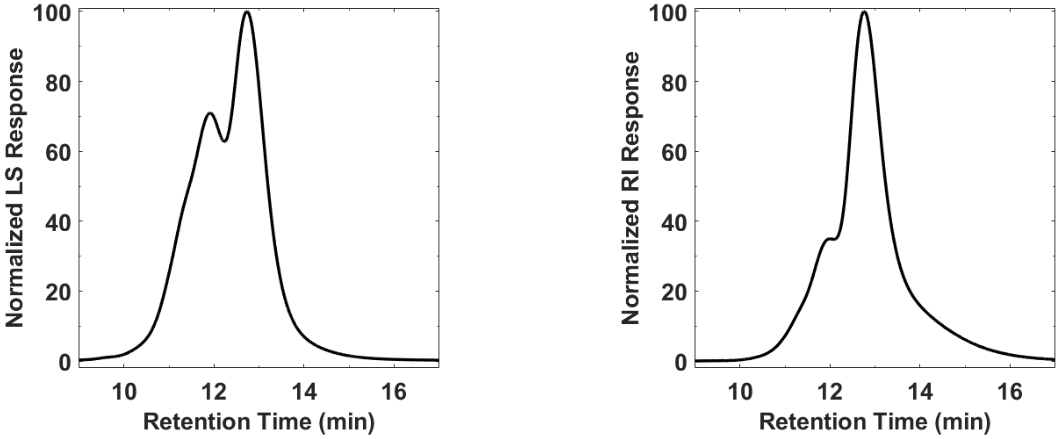

Fig. S386 GPC spectrum of PIP 500 equivalents generated by **$Sm(CH_2SiMe_3)_3(THF)_3$**, 2 equivalents $[Ph_3C][B(C_6F_5)_4]$, and 1 equivalent PPh_3 from **Table 7**, entry 3 (PPh_3 addition time 30 min): (left) LS; (right) RI.

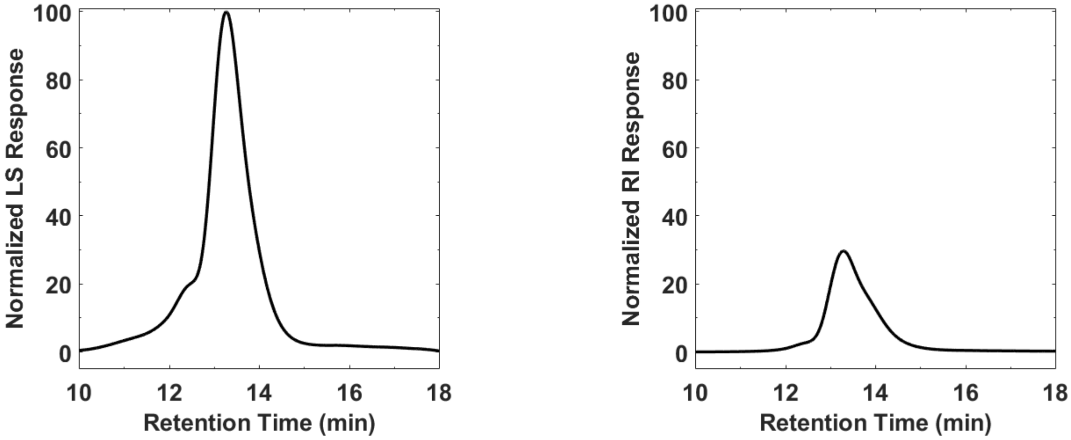

Fig. S387 GPC spectrum of PIP 500 equivalents generated by **$Gd(CH_2SiMe_3)_3(THF)_2$**, 2 equivalents $[Ph_3C][B(C_6F_5)_4]$, and 1 equivalent PPh_3 from **Table 7**, entry 4 (PPh_3 addition time 0 min): (left) LS; (right) RI.

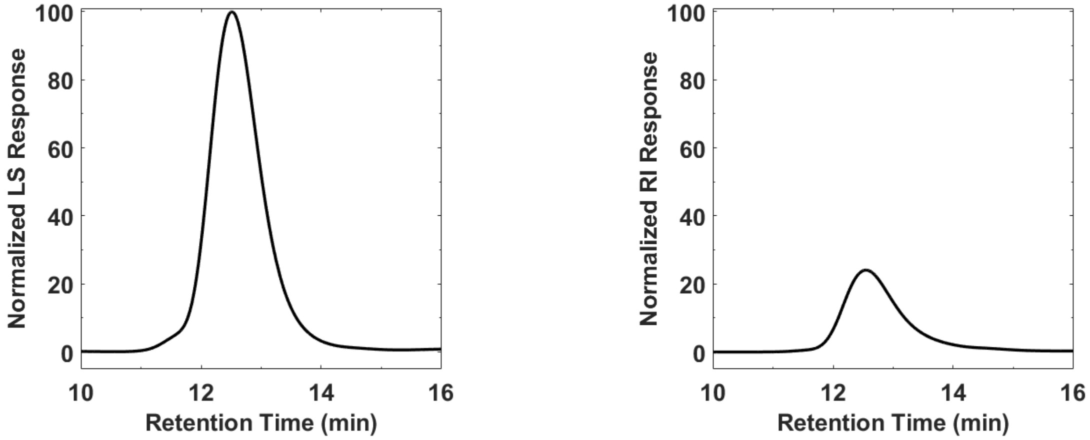

Fig. S388 GPC spectrum of PIP 500 equivalents generated by **$Gd(CH_2SiMe_3)_3(THF)_2$**, 2 equivalents $[Ph_3C][B(C_6F_5)_4]$, and 1 equivalent PPh_3 from **Table 7**, entry 5 (PPh_3 addition time 10 min): (left) LS; (right) RI.

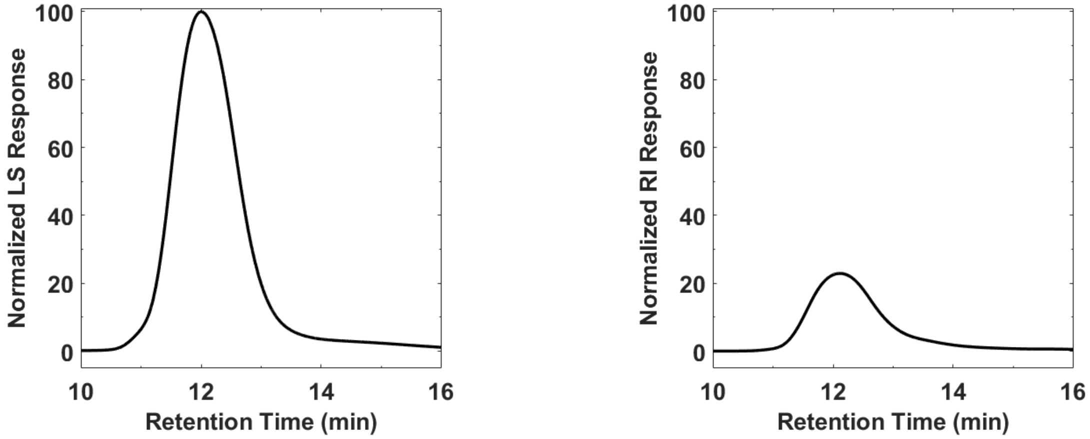

Fig. S389 GPC spectrum of PIP 500 equivalents generated by **$Gd(CH_2SiMe_3)_3(THF)_2$**, 2 equivalents $[Ph_3C][B(C_6F_5)_4]$, and 1 equivalent PPh_3 from **Table 7**, entry 6 (PPh_3 addition time 30 min): (left) LS; (right) RI.

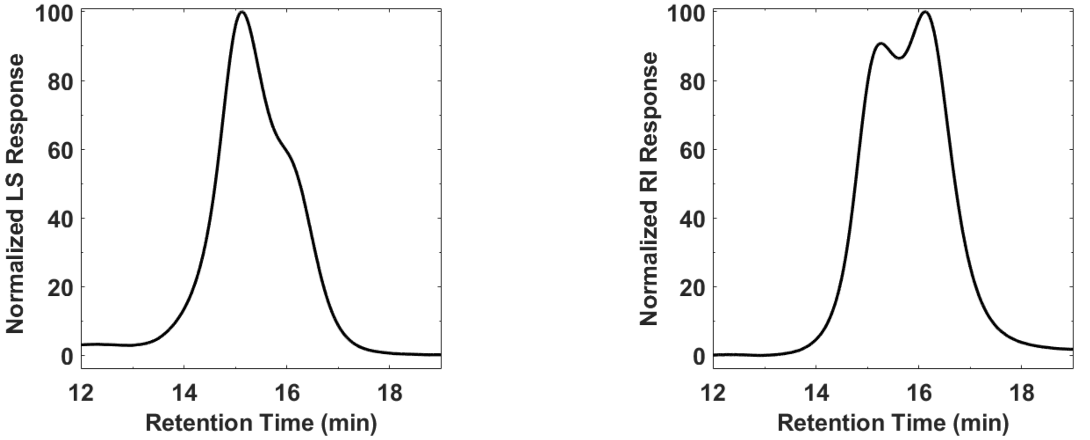

Fig. S390 GPC spectrum of PIP 500 equivalents generated by **$Y(CH_2SiMe_3)_3(THF)_2$**, 2 equivalents $[Ph_3C][B(C_6F_5)_4]$, and 1 equivalent PPh_3 from **Table 7**, entry 7 (PPh_3 addition time 0 min): (left) LS; (right) RI.

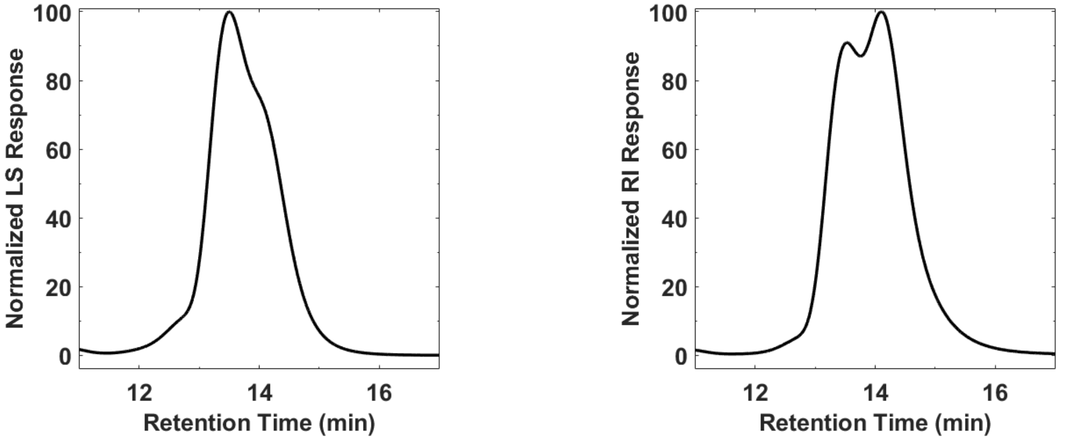

Fig. S391 GPC spectrum of PIP 500 equivalents generated by **$Y(CH_2SiMe_3)_3(THF)_2$**, 2 equivalents $[Ph_3C][B(C_6F_5)_4]$, and 1 equivalent PPh_3 from **Table 7**, entry 8 (PPh_3 addition time 10 min): (left) LS; (right) RI.

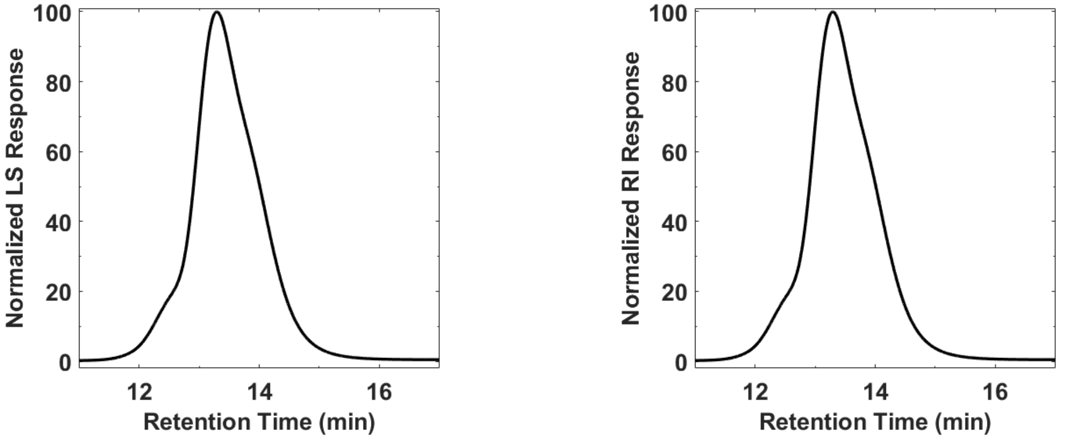

Fig. S392 GPC spectrum of PIP 500 equivalents generated by **$Y(CH_2SiMe_3)_3(THF)_2$**, 2 equivalents $[Ph_3C][B(C_6F_5)_4]$, and 1 equivalent PPh_3 from **Table 7**, entry 9 (PPh_3 addition time 30 min): (left) LS; (right) RI.

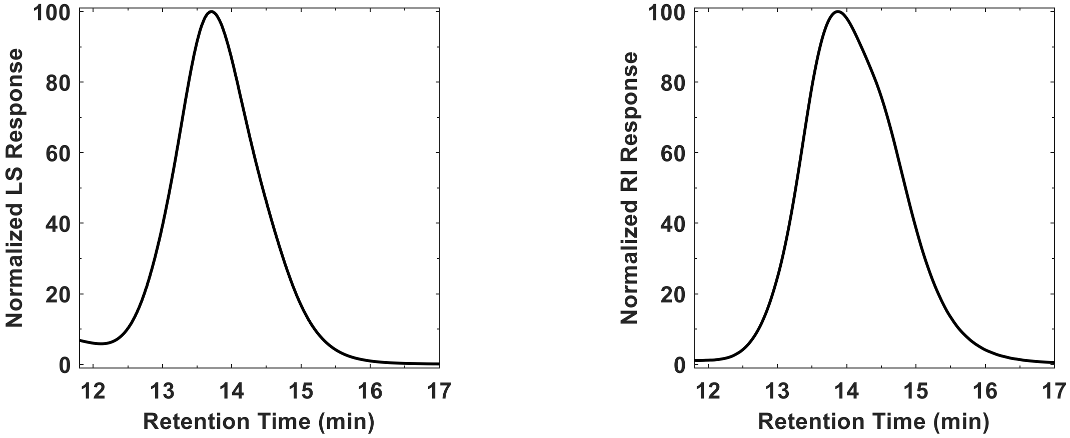

Fig. S393 GPC spectrum of PIP 500 equivalents generated by **$Tm(CH_2SiMe_3)_3(THF)_2$**, 2 equivalents $[Ph_3C][B(C_6F_5)_4]$, and 1 equivalent PPh_3 from **Table 7**, entry 10 (PPh_3 addition time 0 min): (left) LS; (right) RI.

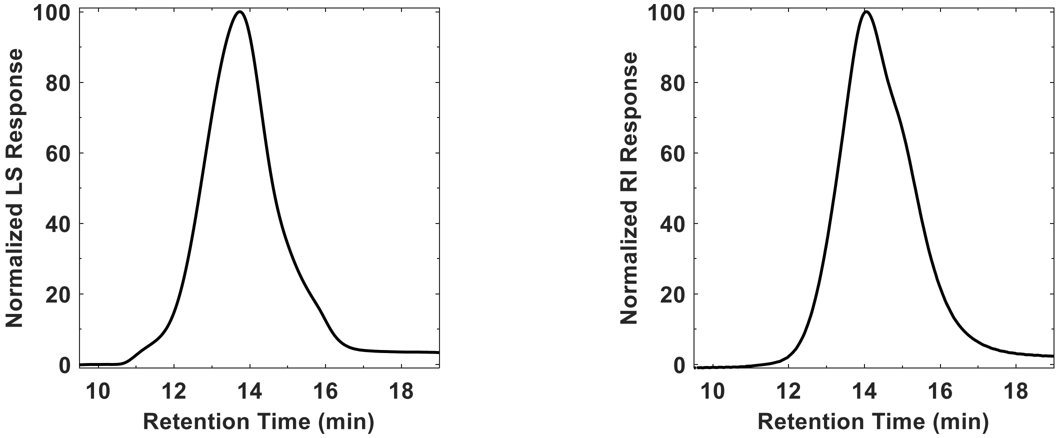

Fig. S394 GPC spectrum of PIP 500 equivalents generated by **$Tm(CH_2SiMe_3)_3(THF)_2$**, 2 equivalents $[Ph_3C][B(C_6F_5)_4]$, and 1 equivalent PPh_3 from **Table 7**, entry 11 (PPh_3 addition time 10 min): (left) LS; (right) RI.

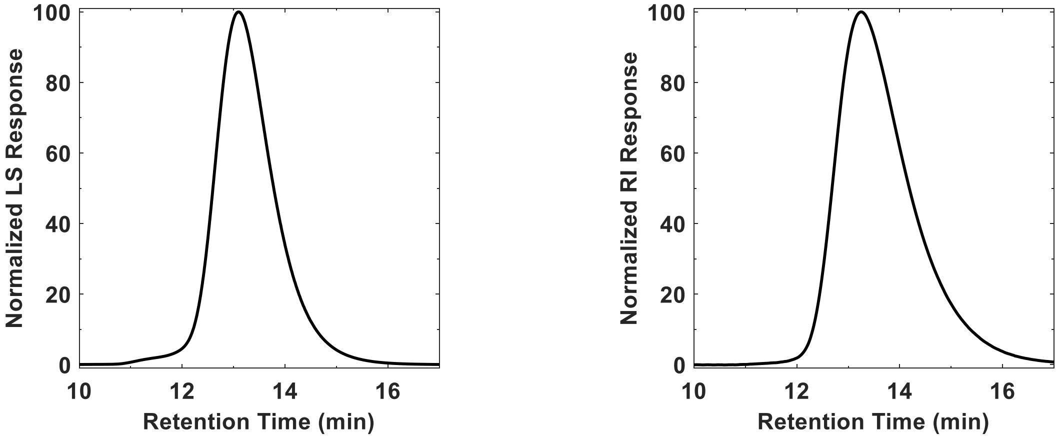

Fig. S395 GPC spectrum of PIP 500 equivalents generated by **$Tm(CH_2SiMe_3)_3(THF)_2$**, 2 equivalents $[Ph_3C][B(C_6F_5)_4]$, and 1 equivalent PPh_3 from **Table 7**, entry 12 (PPh_3 addition time 30 min): (left) LS; (right) RI.

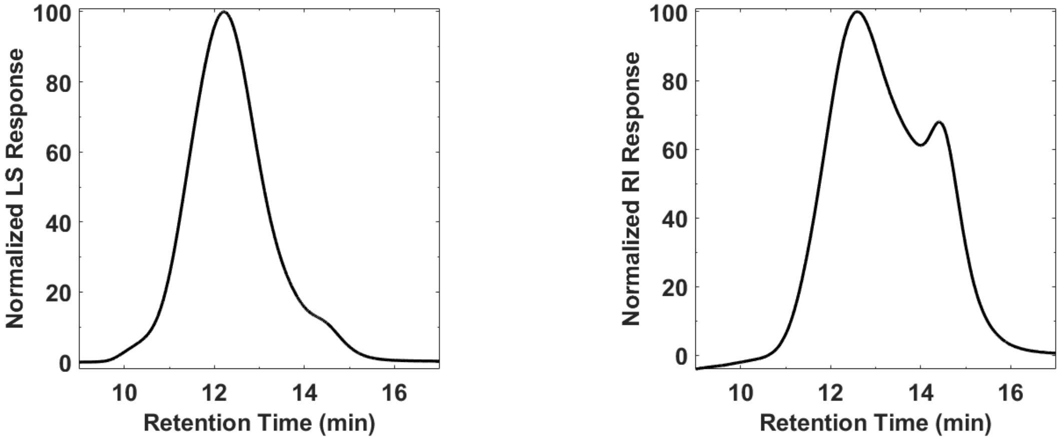

Fig. S396 GPC spectrum of PIP 500 equivalents generated by **$Y(CH_2SiMe_3)_3(THF)_2$** and 2 equivalents $[Ph_3C][B(C_6F_5)_4]$ from **Table 7**, entry 13 (IP addition time 10 min): (left) LS; (right) RI.

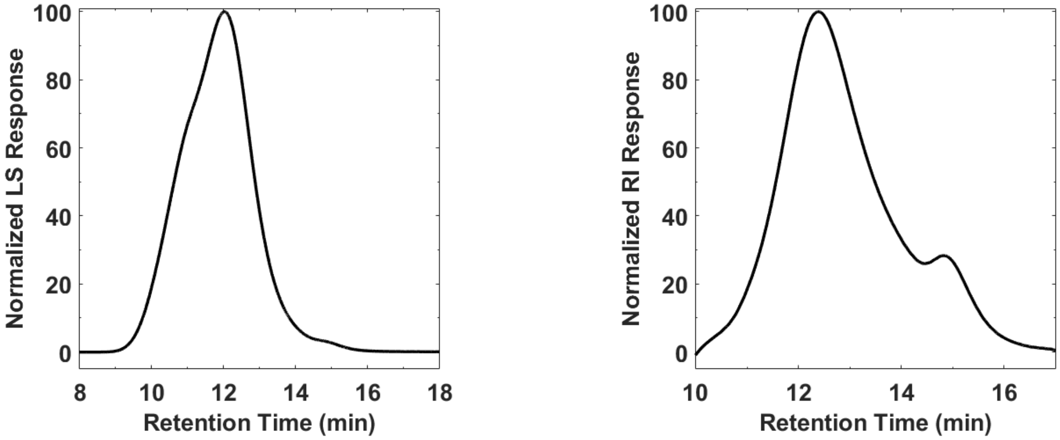

Fig. S397 GPC spectrum of PIP 500 equivalents generated by **$Y(CH_2SiMe_3)_3(THF)_2$** and 2 equivalents $[Ph_3C][B(C_6F_5)_4]$ from **Table 7**, entry 14 (IP addition time 20 min): (left) LS; (right) RI.

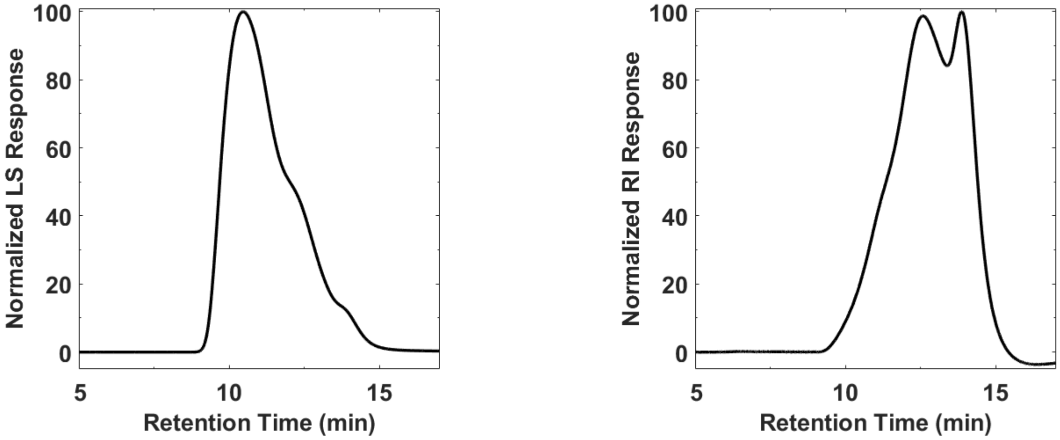

Fig. S398 GPC spectrum of PIP 500 equivalents generated by **$Y(CH_2SiMe_3)_3(THF)_2$** and 2 equivalents $[Ph_3C][B(C_6F_5)_4]$ from **Table 7**, entry 15 (IP addition time 40 min): (left) LS; (right) RI.

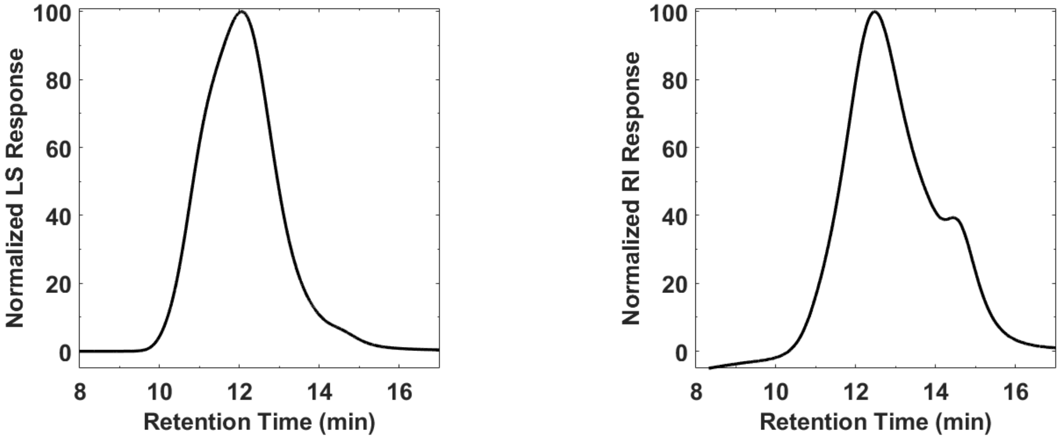

Fig. S399 GPC spectrum of PIP 500 equivalents generated by **$Y(CH_2SiMe_3)_3(THF)_2$**, 2 equivalents $[Ph_3C][B(C_6F_5)_4]$, and 1 equivalent PPh_3 from **Table 8**, entry 1 (Step 1: 60 min): (left) LS; (right) RI.

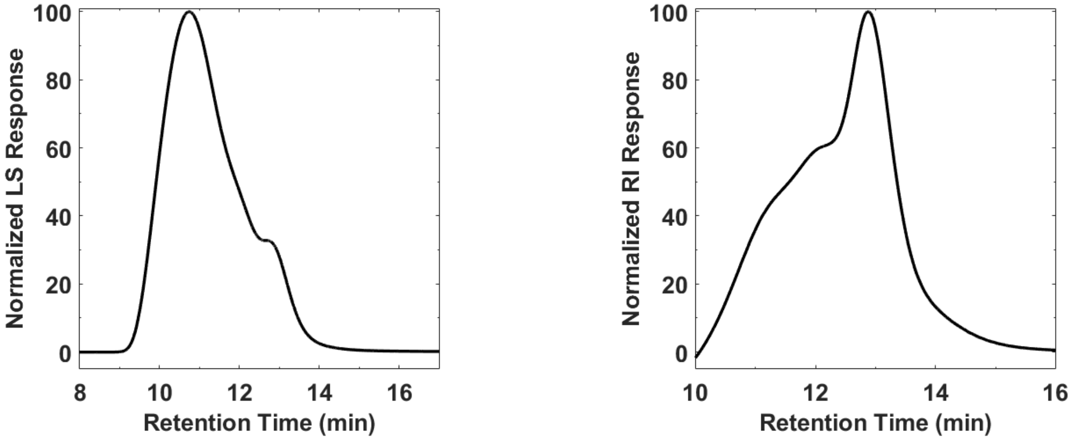

Fig. S400 GPC spectrum of PIP 250 equivalents generated by **$Y(CH_2SiMe_3)_3(THF)_2$**, 2 equivalents $[Ph_3C][B(C_6F_5)_4]$, and 1 equivalent PPh_3 from **Table 8**, entry 2 (Step 2: 60 min): (left) LS; (right) RI.

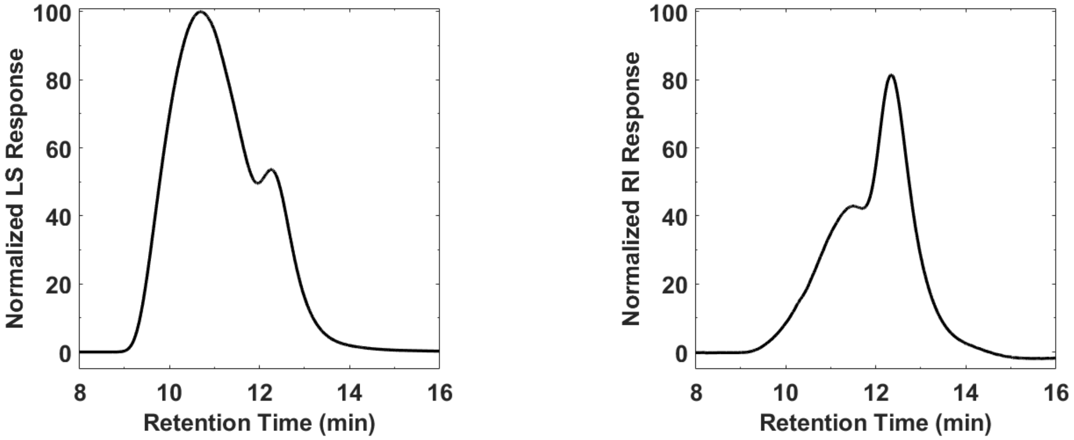

Fig. S401 GPC spectrum of PIP 125 equivalents generated by **$Y(CH_2SiMe_3)_3(THF)_2$**, 2 equivalents $[Ph_3C][B(C_6F_5)_4]$, and 1 equivalent PPh_3 from **Table 8**, entry 3 (Step 3: 60 min): (left) LS; (right) RI.

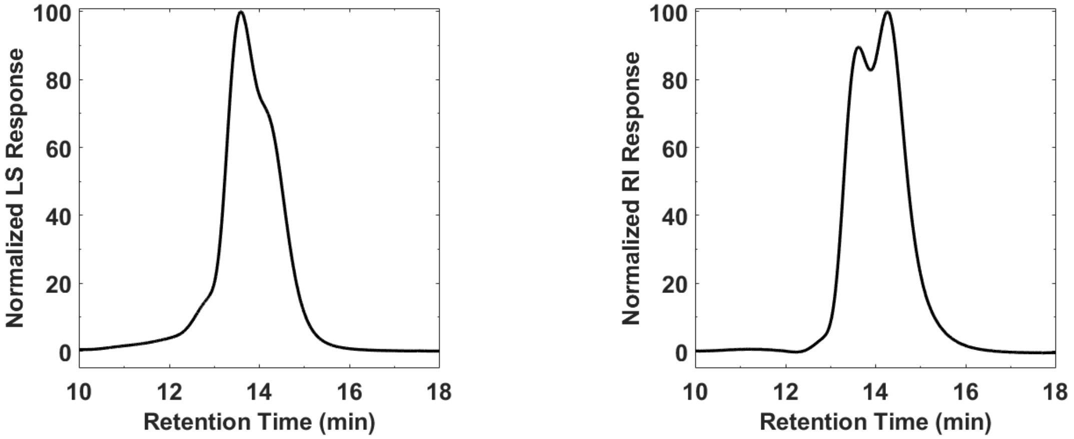

Fig. S402 GPC spectrum of PIP 500 equivalents generated by **$Y(CH_2SiMe_3)_3(THF)_2$** and 2 equivalents $[Ph_3C][B(C_6F_5)_4]$ from **Table 8**, entry 4 (Step 1: 60 min): (left) LS; (right) RI.

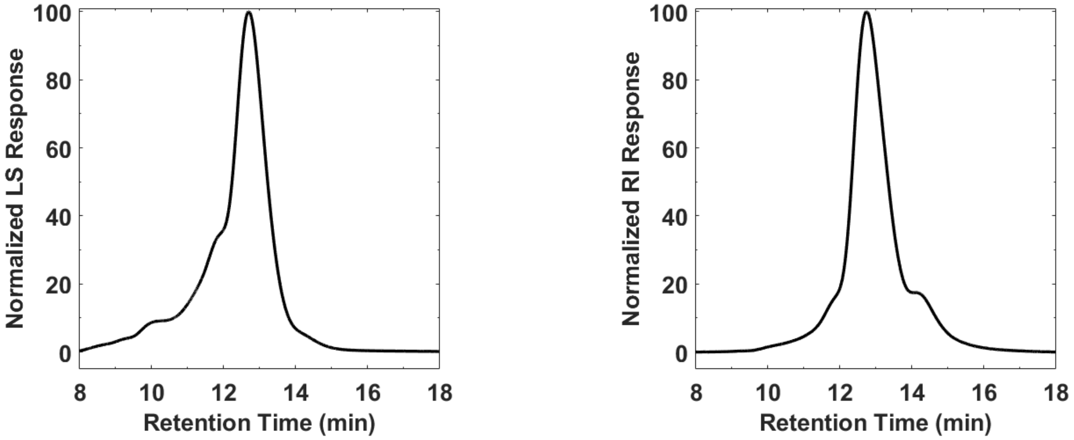

Fig. S403 GPC spectrum of PIP 250 equivalents generated by **$Y(CH_2SiMe_3)_3(THF)_2$** and 2 equivalents $[Ph_3C][B(C_6F_5)_4]$ from **Table 8**, entry 5 (Step 2: 60 min): (left) LS; (right) RI.

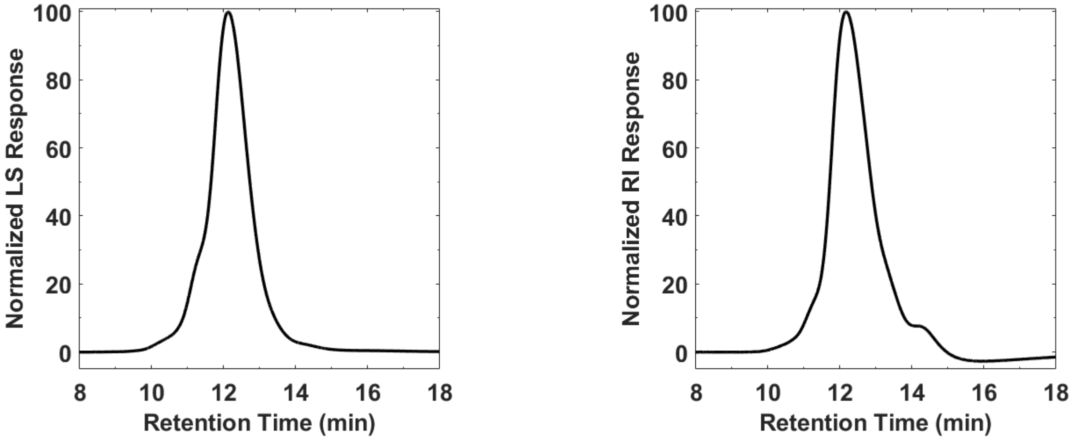

Fig. S404 GPC spectrum of PIP 125 equivalents generated by **$Y(CH_2SiMe_3)_3(THF)_2$** and 2 equivalents $[Ph_3C][B(C_6F_5)_4]$ from **Table 8**, entry 6 (Step 3: 60 min): (left) LS; (right) RI.

6.0 Thermogravimetric Analysis (TGA) of $Y(CH_2SiMe_3)_3(THF)_2$

TGA curve was recorded using Mettler-Toledo STARe System TGA/DSC 3+ equipped with STARe software, a TA SDTA Sensor LF, XP1 Balance, and a sample robot. Sample weight of crystallized $Y(CH_2SiMe_3)_3(THF)_2$ was 8.91 mg sealed in 40 μL aluminum crucible fitted with a pierceable lid. General method involves heating from 25 ºC to 500 ºC at a scan rate of 10 ºC/min under a constant flow of N_2 (40 mL/min).

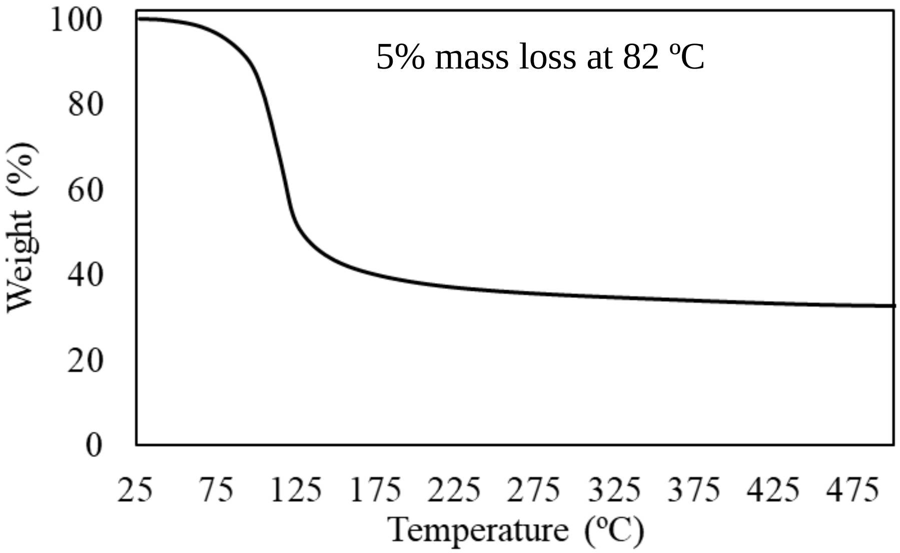

Fig. S405 TGA curve of $Y(CH_2SiMe_3)_3(THF)_2$.

7.0 *In situ* NMR studies

7.1 *In situ* synthesis of $[Y(CH_2SiMe_3)_2(THF)_2]^+[B(C_6F_5)_4]^-$ and $[Y(CH_2SiMe_3)(THF)_2]^{2+}[B(C_6F_5)_4]^{2-}$

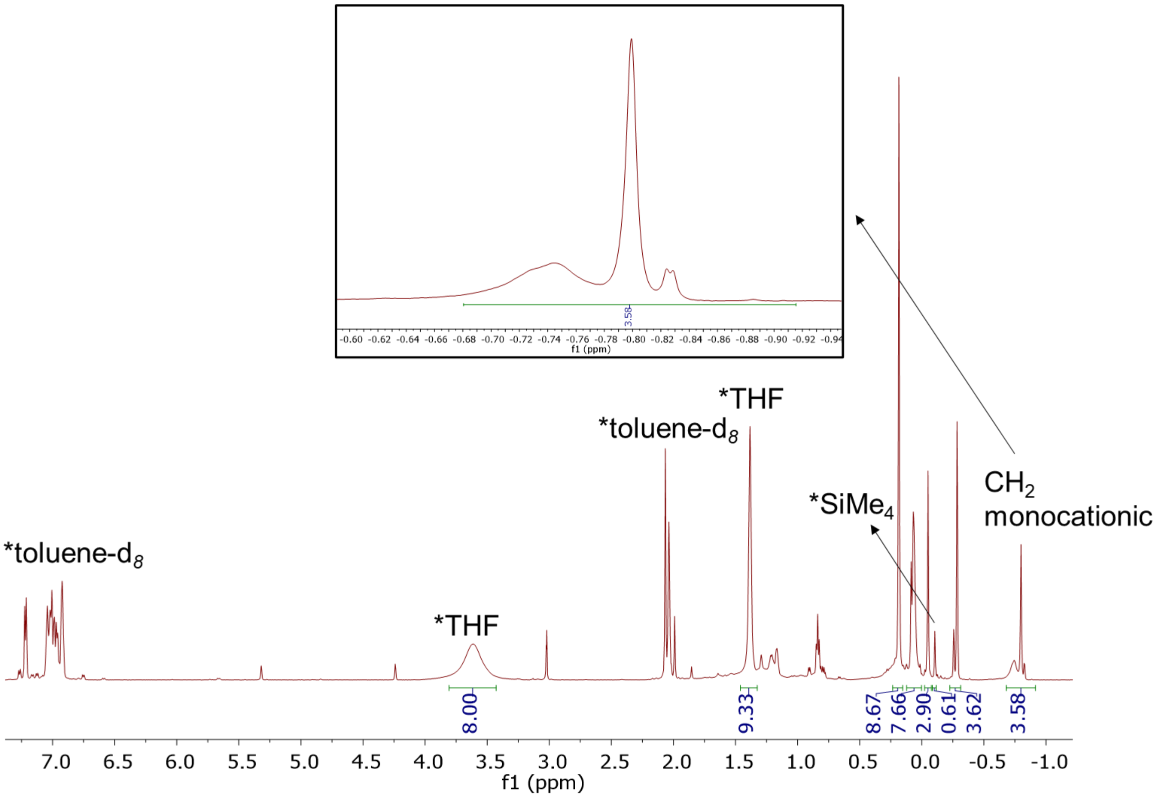

Fig. S406 *In situ* ^{1}H NMR spectrum of the monocationic active species $[Y(CH_2SiMe_3)_2(THF)_2]^+[B(C_6F_5)_4]^-$ from the reaction of complex $Y(CH_2SiMe_3)_3(THF)_2$ with 1 equiv. $[Ph_3C][B(C_6F_5)_4]$ in toluene-d_8 at room temperature.

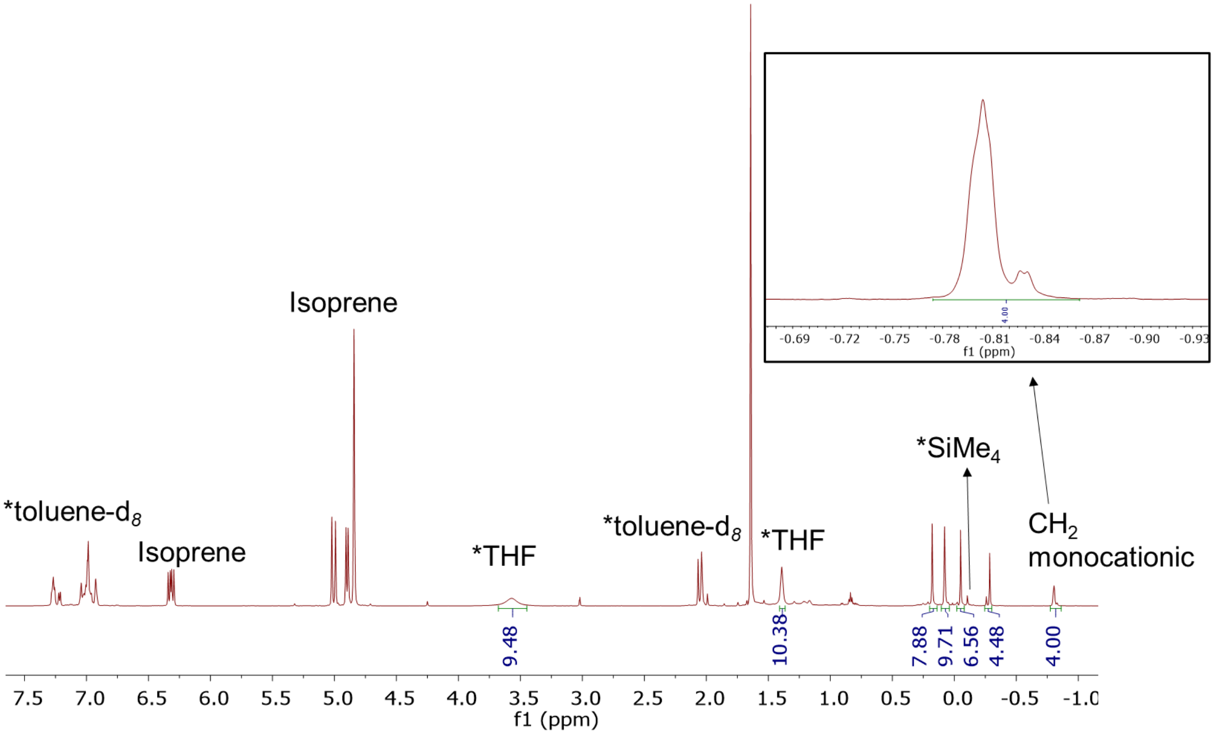

Fig. S407 *In situ* ^{1}H NMR spectrum of the monocationic active species $[Y(CH_2SiMe_3)_2(THF)_2]^+[B(C_6F_5)_4]^-$ from the reaction of complex $Y(CH_2SiMe_3)_3(THF)_2$ with 1 equiv. $[Ph_3C][B(C_6F_5)_4]$ after IP addition in toluene-d_8 at room temperature.

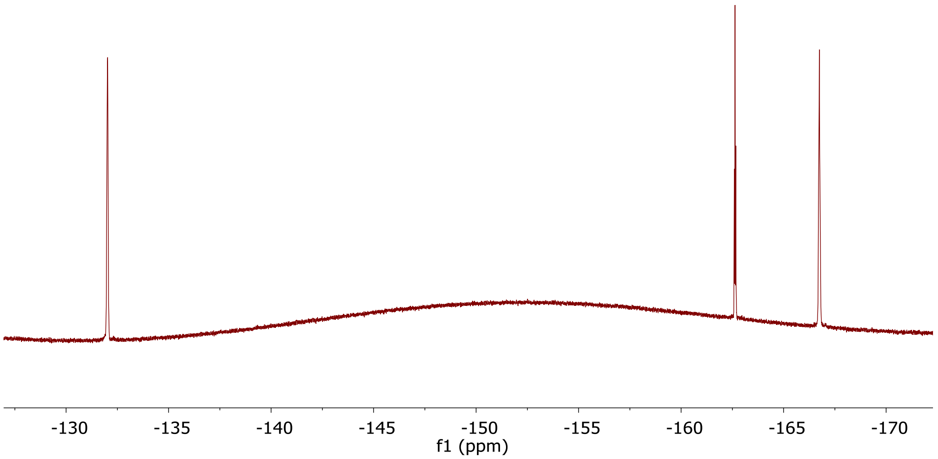

Fig. S408 *In situ* ^{19}F NMR spectrum of the monocationic active species $[Y(CH_2SiMe_3)_2(THF)_2]^+[B(C_6F_5)_4]^-$ from the reaction of complex $Y(CH_2SiMe_3)_3(THF)_2$ with 1 equiv. $[Ph_3C][B(C_6F_5)_4]$ in toluene-d_8 at room temperature.

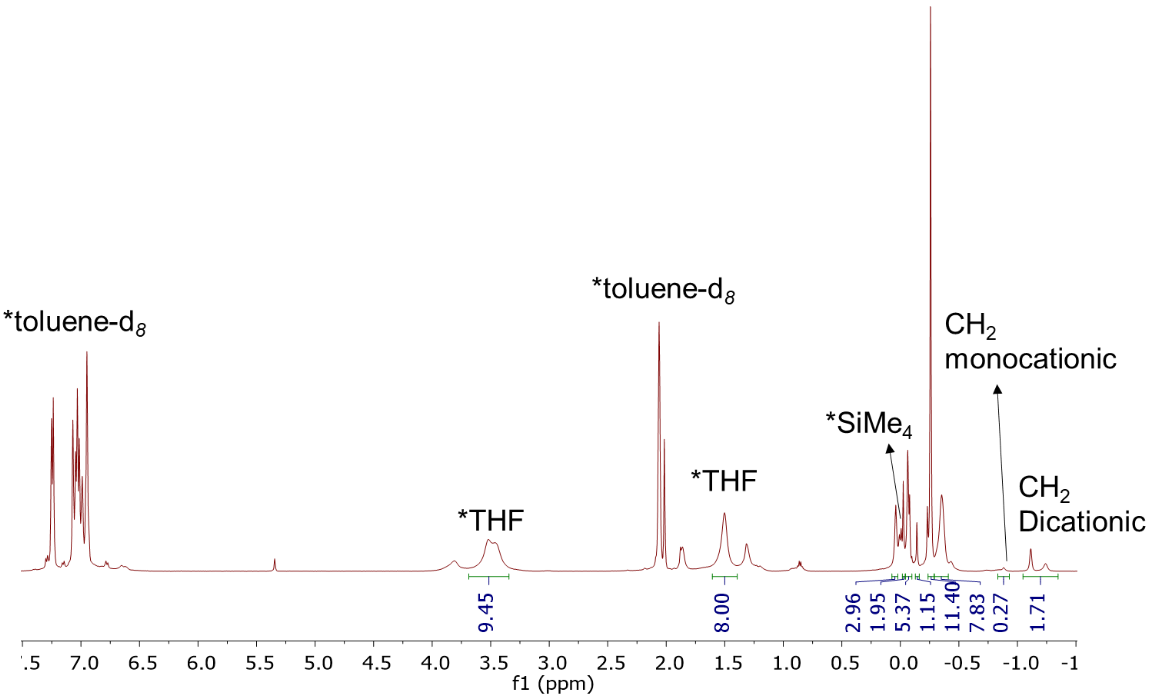

Fig. S409 *In situ* ^{1}H NMR spectrum of the dicationic active species $[Y(CH_2SiMe_3)_2(THF)_2]^{2+}[B(C_6F_5)_4]^{2-}$ from the reaction of complex $Y(CH_2SiMe_3)_3(THF)_2$ with 2 equiv. $[Ph_3C][B(C_6F_5)_4]$ in toluene-d_8 at room temperature.

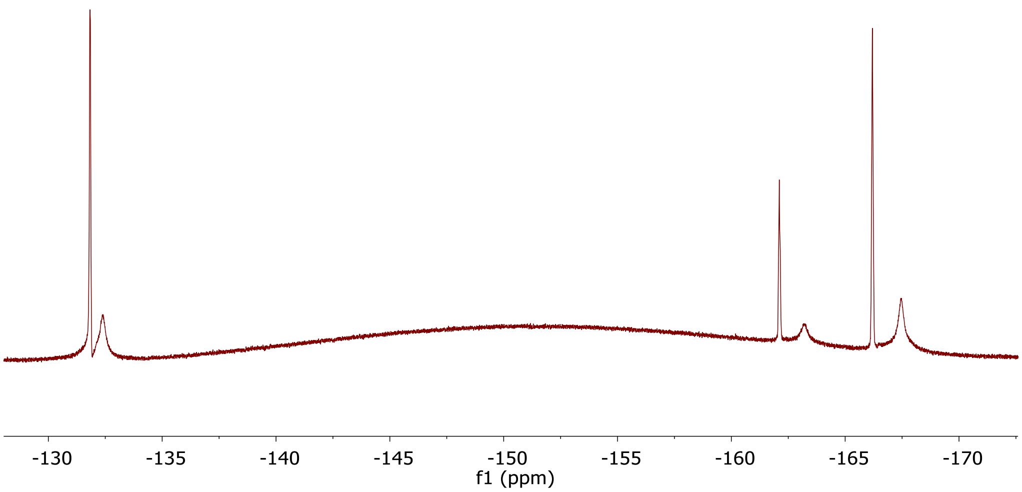

Fig. S410 *In situ* ^{19}F NMR spectrum of the dicationic active species $[Y(CH_2SiMe_3)(THF)_2]^{2+}[B(C_6F_5)_4]^{2-}$ from the reaction of complex $Y(CH_2SiMe_3)_3(THF)_2$ with 1 equiv. $[Ph_3C][B(C_6F_5)_4]$ in toluene-d_8 at room temperature.

7.2 *In situ* NMR Studies with PPh_3

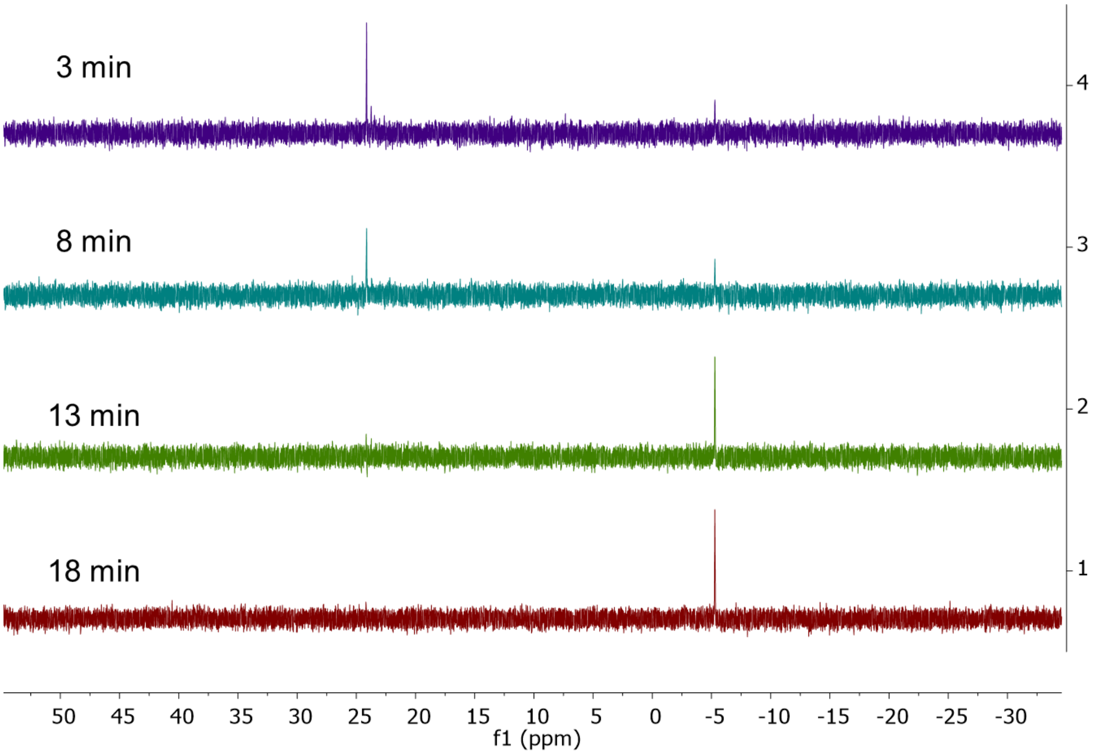

Fig. S411 *In situ* ^{31}P NMR spectrum of the dicationic active species $[Y(CH_2SiMe_3)(THF)_2]^{2+}[B(C_6F_5)_4]^{2-}$ from the reaction of complex $Y(CH_2SiMe_3)_3(THF)_2$ with 2 equiv. $[Ph_3C][B(C_6F_5)_4]$ and 1 equiv. PPh_3 added 10 min after activation in toluene-d_8 at room temperature. Reaction monitored overtime, ^{31}P NMR taken in 5 minute intervals.

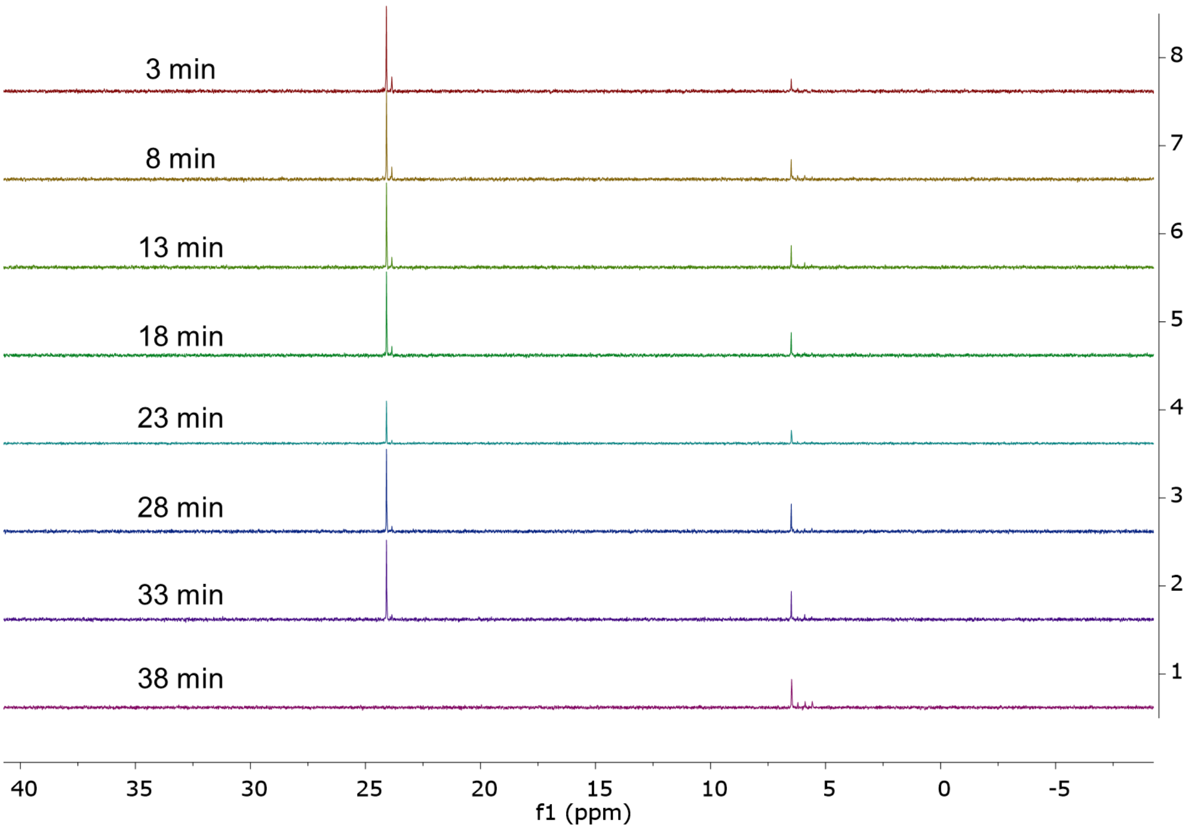

Fig. S412 *In situ* ^{31}P NMR spectrum of 1 equiv. PPh_3 with 2 equiv. $[Ph_3C][B(C_6F_5)_4]$ in toluene-d_8 at room temperature. Reaction monitored overtime, ^{31}P NMR taken in 5 minute intervals.

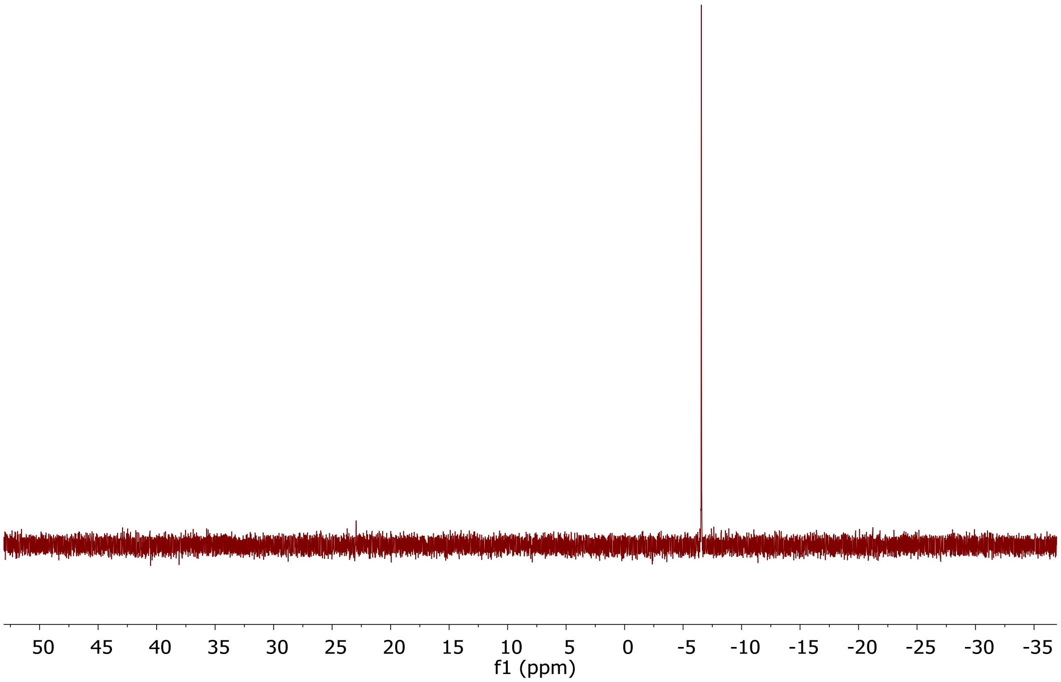

Fig. S413 *In situ* ^{31}P NMR spectrum of the dicationic active species $[Y(CH_2SiMe_3)(THF)_2]^{2+}[B(C_6F_5)_4]^{2-}$ from the reaction of complex $Y(CH_2SiMe_3)_3(THF)_2$ with 2 equiv. $[Ph_3C][B(C_6F_5)_4]$ and 1 equiv. PPh_3 in toluene-d_8 at -80 ºC.

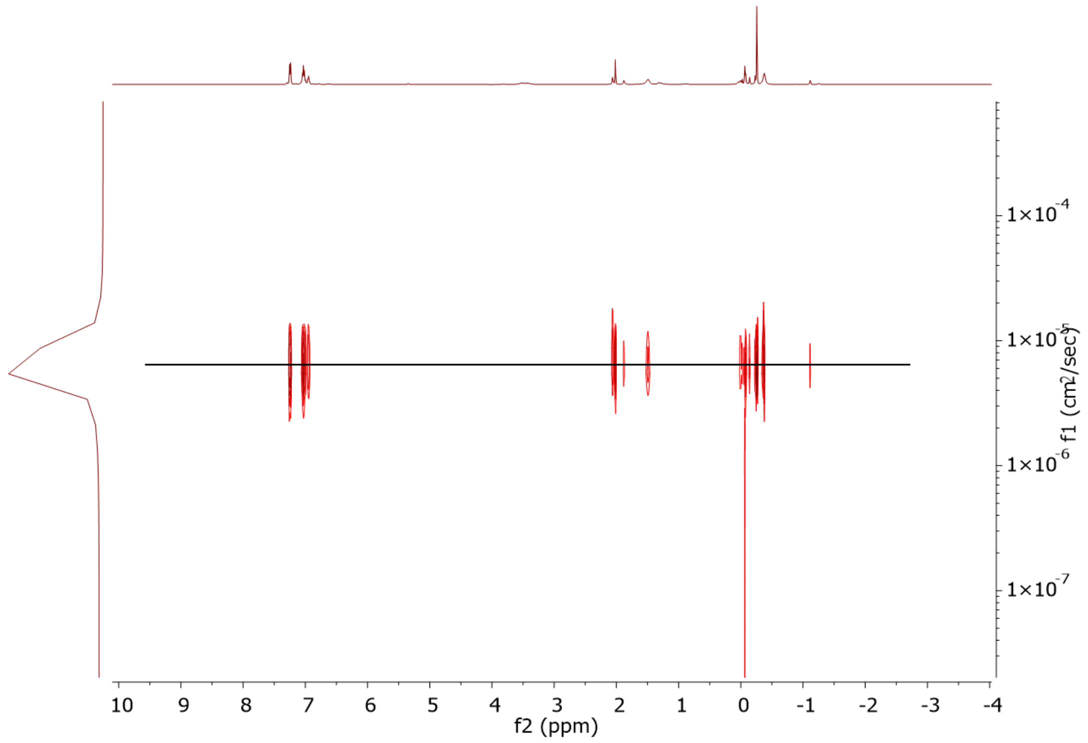

Fig. S414 DOSY NMR spectrum of the dicationic active species $[Y(CH_2SiMe_3)(THF)_2]^{2+}[B(C_6F_5)_4]^{2-}$ from the reaction of complex $Y(CH_2SiMe_3)_3(THF)_2$ with 2 equiv. $[Ph_3C][B(C_6F_5)_4]$ in toluene-d_8 at room temperature.

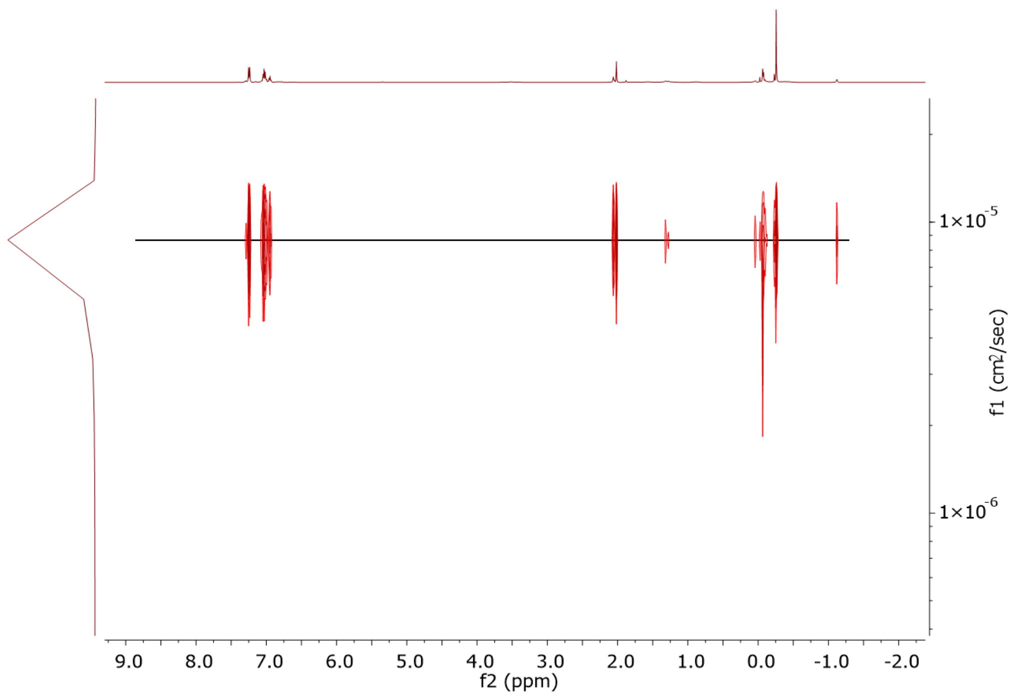

Fig. S415 DOSY NMR spectrum of the dicationic active species $[Y(CH_2SiMe_3)(THF)_2]^{2+}[B(C_6F_5)_4]^{2-}$ from the reaction of complex $Y(CH_2SiMe_3)_3(THF)_2$ with 2 equiv. $[Ph_3C][B(C_6F_5)_4]$ and 1 equiv. PPh_3 in toluene-d_8 at room temperature.

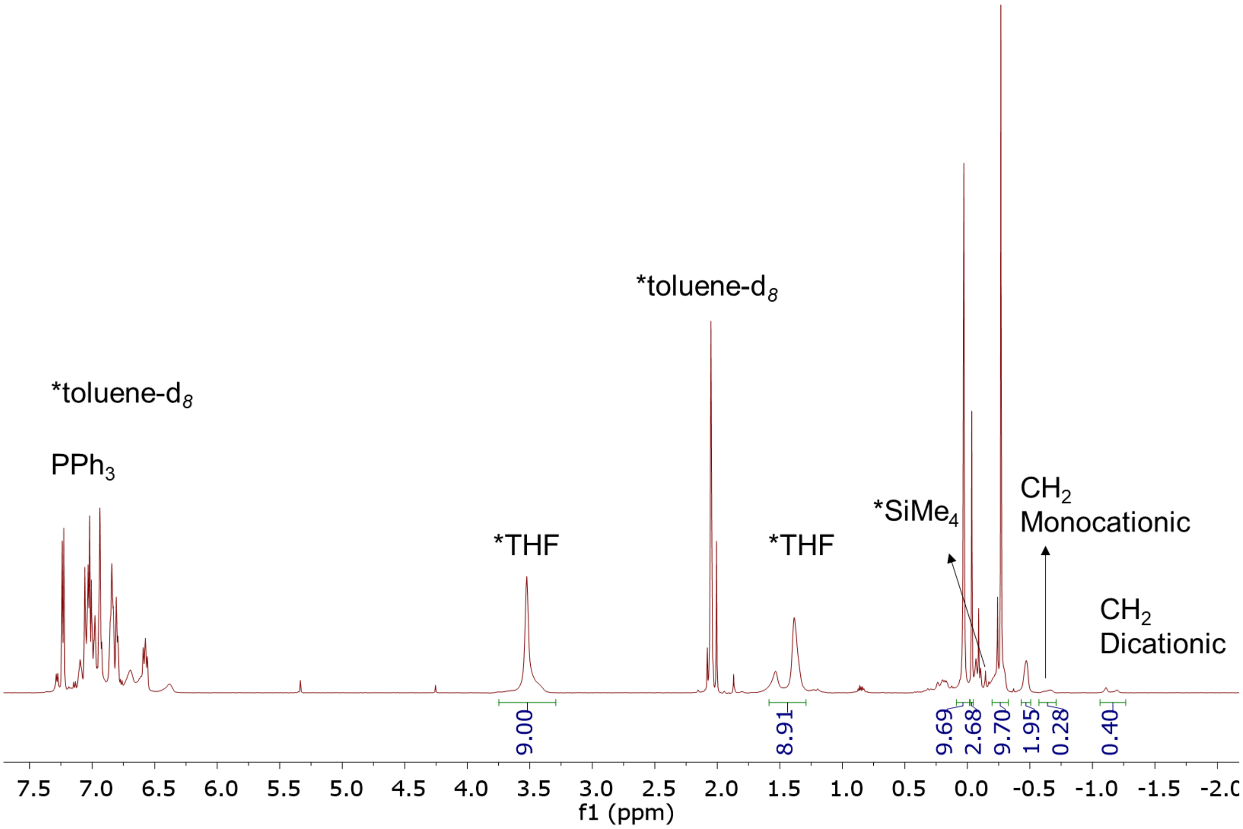

Fig. S416 *In situ* ^{1}H NMR spectrum of the dicationic active species $[Y(CH_2SiMe_3)(THF)_2]^{2+}[B(C_6F_5)_4]^{2-}$ from the reaction of complex $Y(CH_2SiMe_3)_3(THF)_2$ with 2 equiv. $[Ph_3C][B(C_6F_5)_4]$ and PPh_3 added at time 0 min in toluene-d_8 at room temperature.

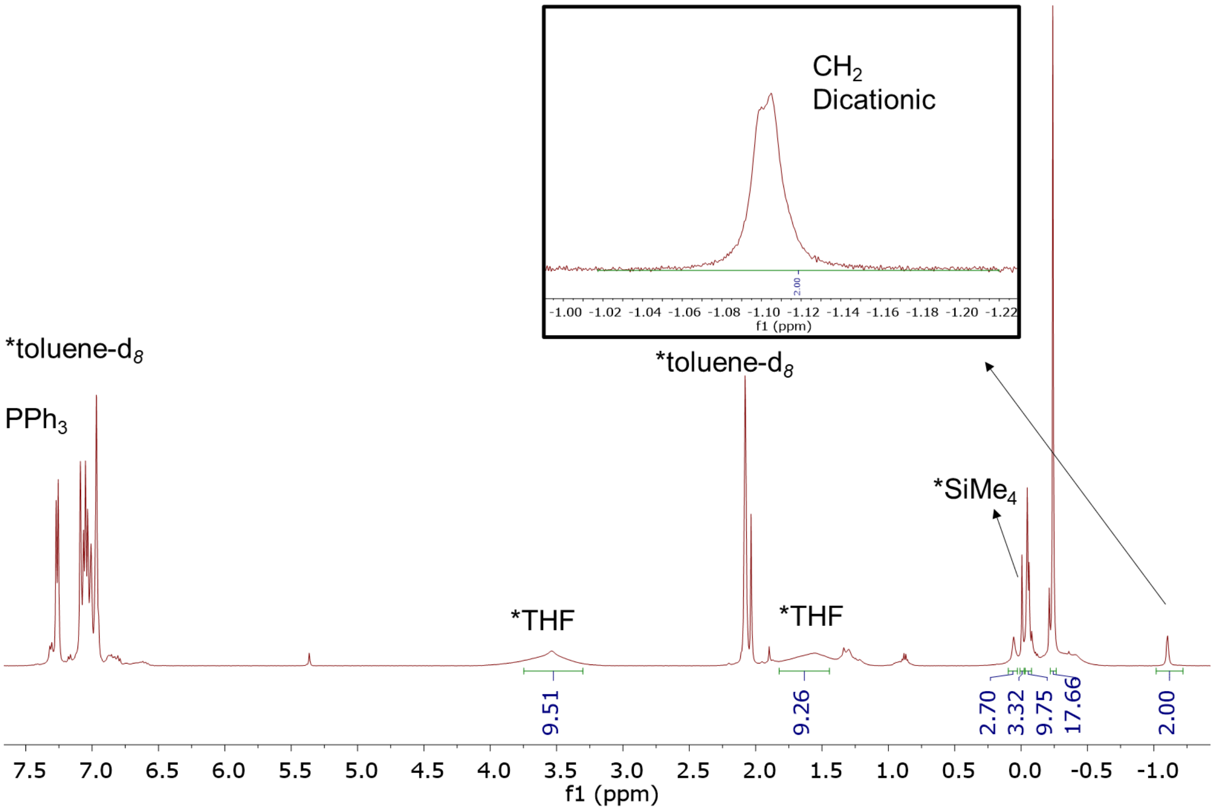

Fig. S417 *In situ* ^{1}H NMR spectrum of the dicationic active species $[Y(CH_2SiMe_3)(THF)_2]^{2+}[B(C_6F_5)_4]^{2-}$ from the reaction of complex $Y(CH_2SiMe_3)_3(THF)_2$ with 2 equiv. $[Ph_3C][B(C_6F_5)_4]$ and PPh_3 added at time 10 min in toluene-d_8 at room temperature.

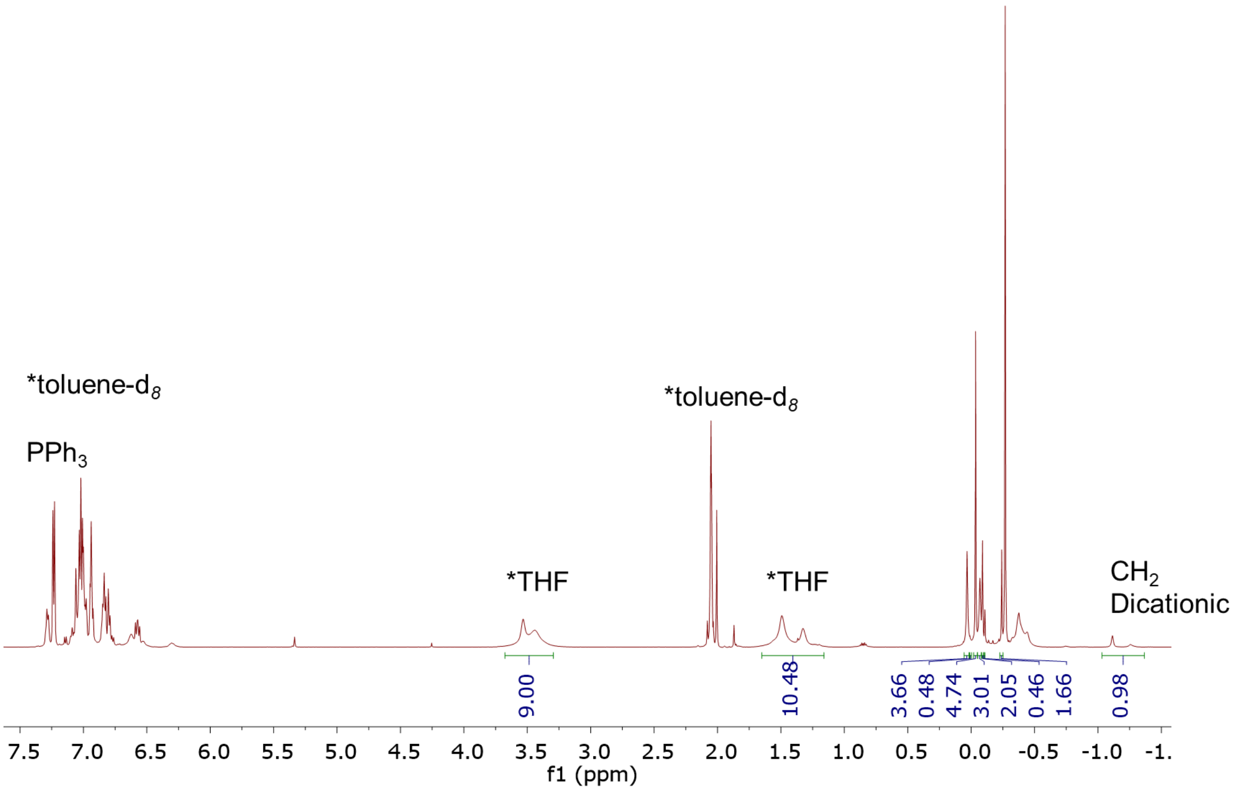

Fig. S418 *In situ* ^{1}H NMR spectrum of the dicationic active species $[Y(CH_2SiMe_3)(THF)_2]^{2+}[B(C_6F_5)_4]^{2-}$ from the reaction of complex $Y(CH_2SiMe_3)_3(THF)_2$ with 2 equiv. $[Ph_3C][B(C_6F_5)_4]$ and PPh_3 added at time 30 min in toluene-d_8 at room temperature.

7.3 *In situ* reaction of $Y(CH_2SiMe_3)_3(THF)_2$ with 3 equiv. $[Ph_3C][B(C_6F_5)_4]$.

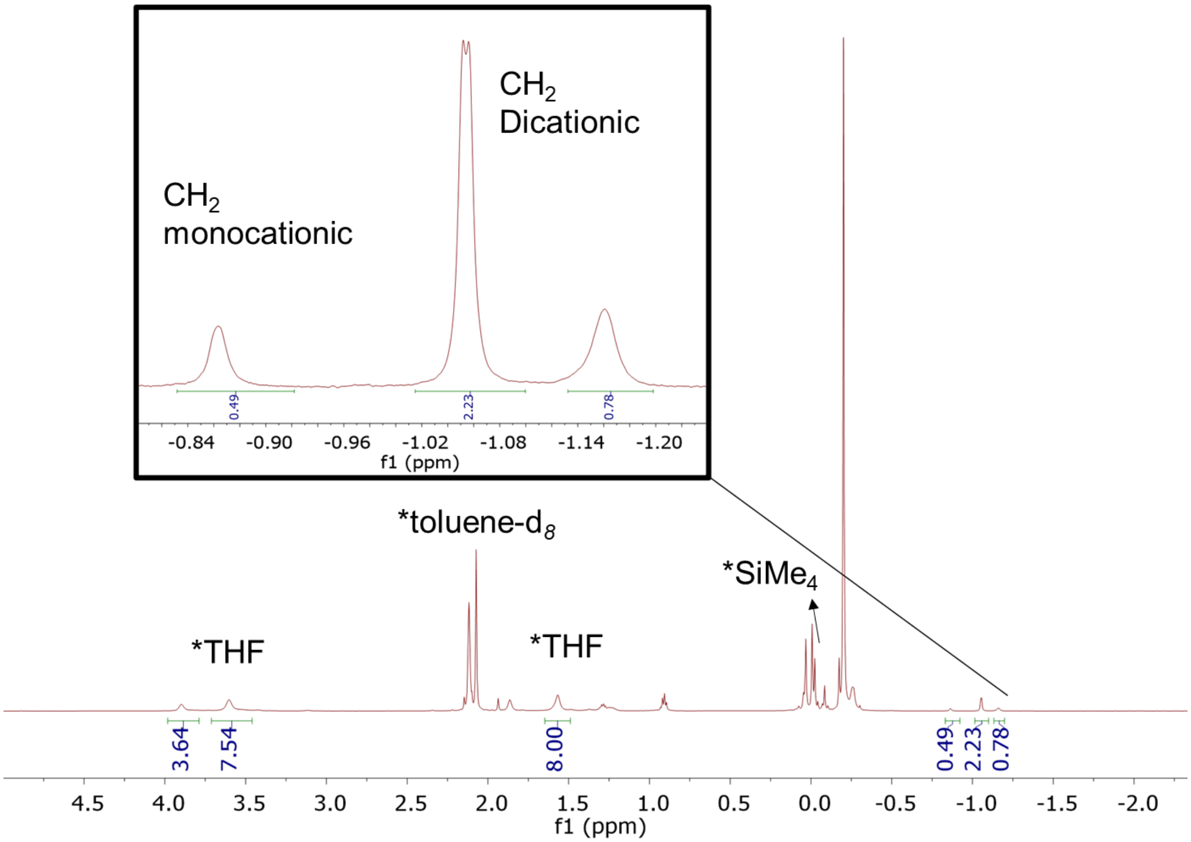

Fig. S419 *In situ* ^{1}H NMR spectrum of the reaction of complex $Y(CH_2SiMe_3)_3(THF)_2$ with 3 equiv. $[Ph_3C][B(C_6F_5)_4]$ in toluene-d_8 at room temperature. NMR taken 10 minutes after catalyst addition.

7.4 *In situ* reaction of $Y(CH_2SiMe_3)_3(THF)_2$ with 2 equiv. $[Ph_3C][B(C_6F_5)_4]$ and 5 equiv. $AlMe_3$.

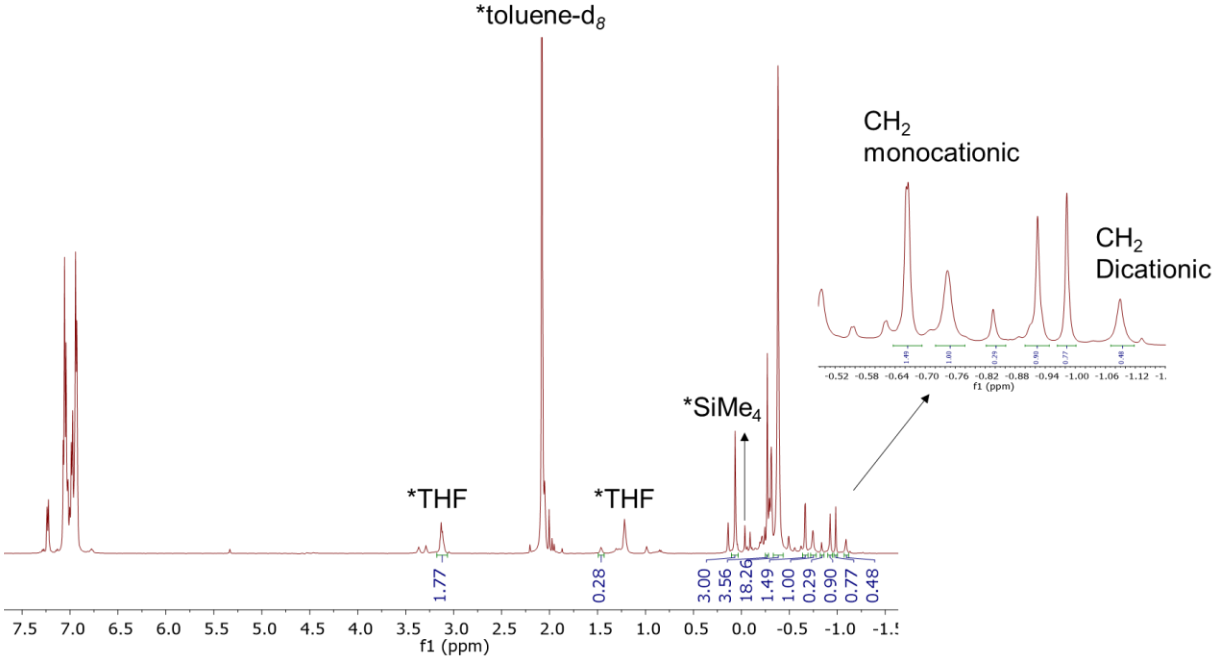

Fig. S420 *In situ* ^1H NMR spectrum of the reaction of complex $Y(CH_2SiMe_3)_3(THF)_2$ with 2 equiv. $[Ph_3C][B(C_6F_5)_4]$ and 5 equiv. $AlMe_3$ in toluene-d_8 at room temperature.

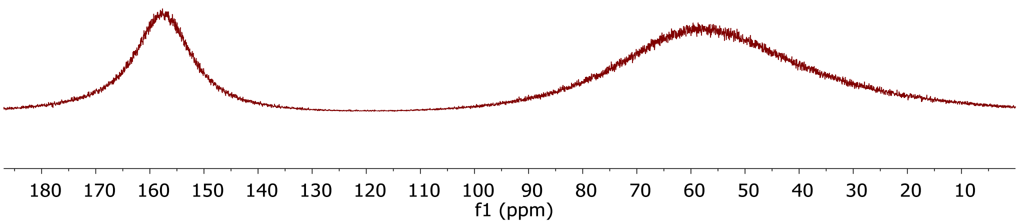

Fig. S421 *In situ* ^{27}Al NMR spectrum of the reaction of complex $Y(CH_2SiMe_3)_3(THF)_2$ with 2 equiv. $[Ph_3C][B(C_6F_5)_4]$ and 5 equiv. $AlMe_3$ in toluene-d_8 at room temperature.
